# Spirostrain-Accelerated Chemiexcitation of Dioxetanes Yields Unprecedented Detection Sensitivity in Chemiluminescence Bioassays

**DOI:** 10.1021/acscentsci.3c01141

**Published:** 2023-10-24

**Authors:** Rozan Tannous, Omri Shelef, Sara Gutkin, Maya David, Thomas Leirikh, Liang Ge, Qais Jaber, Qingyang Zhou, Pengchen Ma, Micha Fridman, Urs Spitz, Kendall N. Houk, Doron Shabat

**Affiliations:** † School of Chemistry, Raymond and Beverly Sackler Faculty of Exact Sciences, Tel-Aviv University, Tel Aviv 69978, Israel; ‡ Department of Chemistry and Biochemistry, University of California, Los Angeles, California 90095, United States; § Department of Chemistry, School of Chemistry, Xi’an Key Laboratory of Sustainable Energy Material Chemistry and Engineering Research Center of Energy Storage Materials and Devices, Ministry of Education, Xi’an Jiaotong University, Xi’an 710049, People’s Republic of China; ∥ BIOSYNTH, Rietlistr. 4 Postfach 125 9422 Staad, Switzerland

## Abstract

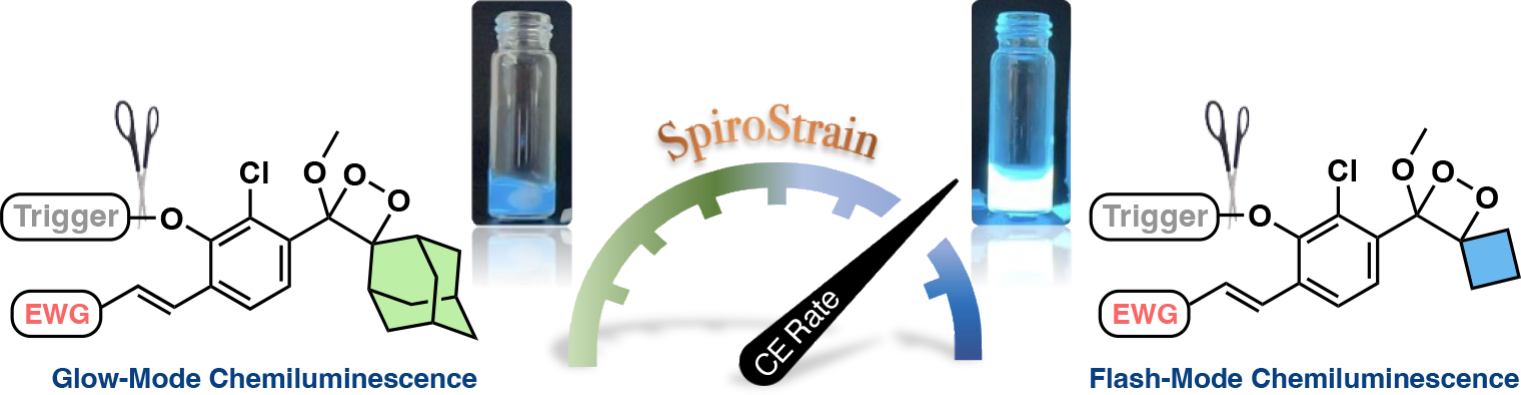

Chemiluminescence is a fascinating phenomenon that involves the generation of light through chemical reactions. The light emission from adamantyl-phenoxy-1,2-dioxetanes can glow from minutes to hours depending on the specific substituent present on the dioxetane molecule. In order to improve the light emission properties produced by these chemiluminescent luminophores, it is necessary to induce the chemiexcitation rate to a flash mode, wherein the bulk of light is emitted instantly rather than slowly over time. We report the realization of this goal through the incorporation of spirostrain release into the decomposition of 1,2-dioxetane luminophores. DFT computational simulations provided support for the hypothesis that the spiro-cyclobutyl substituent accelerates chemiexcitation as compared to the unstrained adamantyl substituent. Spiro-linking of cyclobutane and oxetane units led to greater than 100-fold and 1000-fold emission enhancement, respectively. This accelerated chemiexcitation rate increases the detection sensitivity for known chemiluminescent probes to the highest signal-to-noise ratio documented to date. A turn-ON probe, containing a spiro-cyclobutyl unit, for detecting the enzyme β-galactosidase exhibited a limit of detection value that is 125-fold more sensitive than that for the previously described adamantyl analogue. This probe was also able to instantly detect and image β-gal activity with enhanced sensitivity in *E. coli* bacterial assays.

## Abstract 2

Chemiexcitation acceleration was attained by incorporating a cyclobutyl unit as a spirostrain motif into 1,2-dioxetane luminophores, resulting in significantly enhanced detection sensitivity.

## Introduction

Chemiluminescence is a light-producing phenomenon generated by a chemical reaction.^1^ Molecules that produce chemiluminescence can emit light by either a stable glow-type or fast flash-type reaction. The glow-type chemiluminescent reaction generates a stable long light emission profile with relatively low intensity, but the flash mode of the chemiluminescent reaction generates a short light emission profile with a highly intense signal. Flash luminescent reactions occur quickly, in a matter of seconds or minutes, giving off a very bright signal. Consequently, assays utilizing flash-type chemistry offer superior detection sensitivity compared to those employing glow-type chemistry.^2^

**Figure 1:**
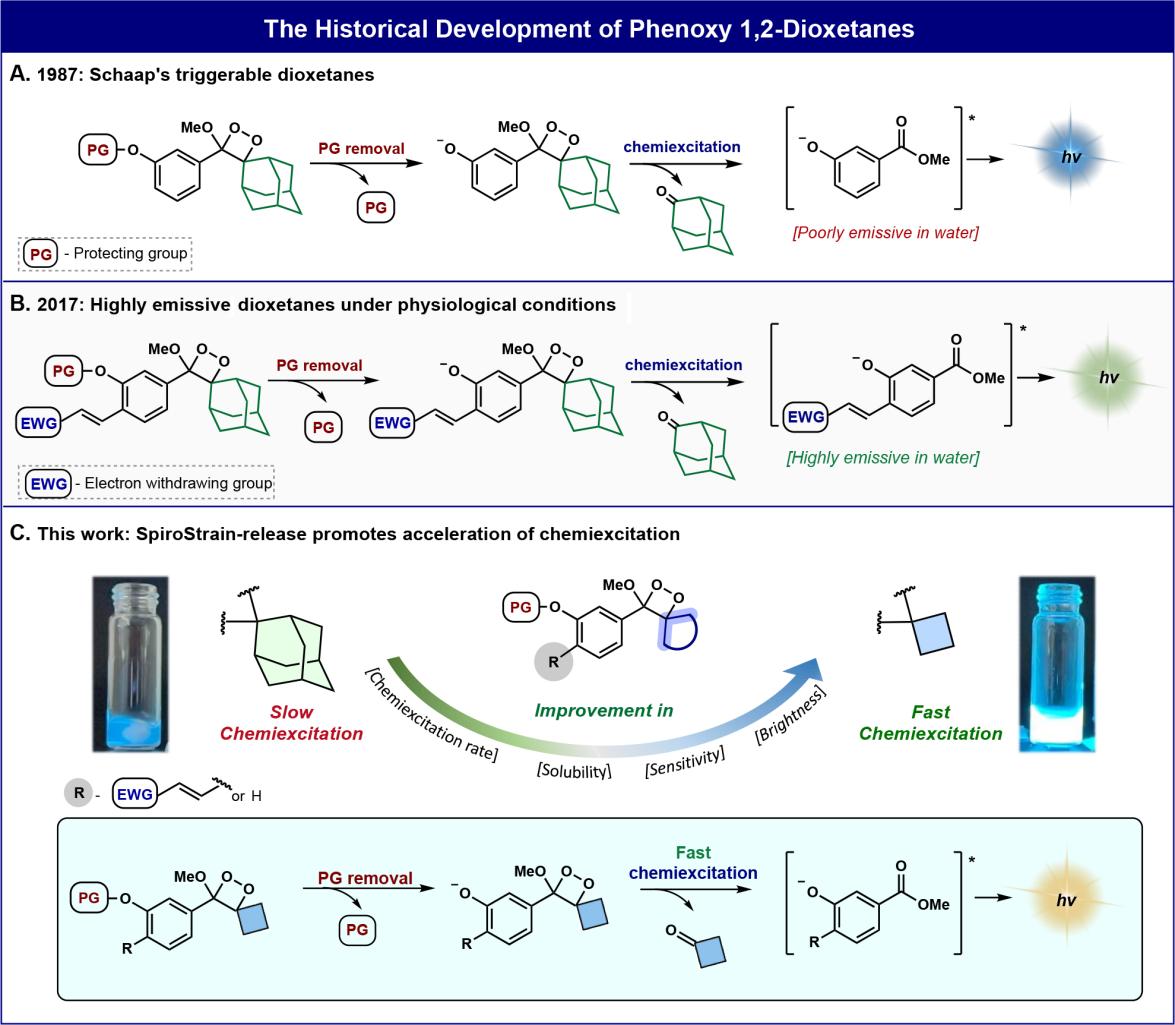
(A) Activation and chemiexcitation pathway of Schaap’s dioxetane. (B) Chemiluminescence amplification under aqueous conditions by the *ortho*-substituent effect. (C) This work: spirostrain-release effect on the chemiexcitation rate.

Adamantyl-phenoxy-1,2-dioxetane chemiluminescent luminophores undergo efficient chemiexcitation upon generation of their phenolate ion (Figure 1A).^3^ This chemiexcitation occurs through electron transfer from the phenoxide to the dioxetane accompanying O–O cleavage of the dioxetane, followed by C–C cleavage and formation of an excited benzoate, which emits visible light. While the light emission of phenoxy-1,2-dioxetanes is highly efficient in polar organic solvents, the presence of water results in nearly complete quenching of the light emission. Several years ago, we discovered that the incorporation of an electron-withdrawing group (EWG) as a substituent at the *ortho* position of a phenoxy-adamantyl-1,2-dioxetane prevents water-mediated quenching of the excited intermediate and increases the light-emission intensity of the chemiluminescent luminophore by up to 3000-fold (Figure 1B).^4^ This groundbreaking development enabled the use of our chemiluminescent luminophores as single-component probes with no required additives.^5−9^ Numerous research groups worldwide,^10−16^ including ours, took advantage of the *ortho*-substituted phenoxy-adamantyl-1,2-dioxetane luminophore to develop useful chemiluminescent probes for applications in cells and *in vivo* assays.^17−33^

The light emission from adamantyl-phenoxy-1,2-dioxetanes can glow from minutes to hours, depending on the solvent and the specific substituent present on the dioxetane molecule. Previous efforts by our group to enhance the chemiexcitation rate of chemiluminescent luminophores focused on modifications of the substituent at the *ortho* position of phenoxy-1,2-dioxetanes.^34^ Although these modifications yielded an increase in the chemiexcitation rate, they caused a substantial reduction in the chemiluminescent quantum yields. In order to improve the light emission properties, the rate of chemiexcitation should be significantly enhanced to a flash mode without a decrease of the quantum yield. We now report a substantial acceleration of chemiexcitation rate for phenoxy 1,2-dioxetane luminophores by introduction of the spirofused-cyclobutane-dioxetane unit (Figure 1C). The acceleration results from an angular strain release that is generated by spirostrain. This term is used to emphasize that the spirofusion of the two strained rings causes a spring-loading effect on the O–O stretching, facilitates charge transfer to the developing diradical, and leads to dioxetane decomposition into an excited state.

## Results and Discussion

The rate-determining step of phenoxy-1,2-dioxetane chemiexcitation is the O–O cleavage of the dioxetane that is accompanied by electron transfer from the phenolate to the dioxetane to generate a biradical species.^35^ We hypothesized that by exchanging the spiro-adamantyl-dioxetane unit with a spiro-cyclobutyl-dioxetane, the additional strain at the spiro fusion of two small rings (spirostrain) could lead to faster O–O cleavage and accelerated chemiexcitation. We explored this spirostrain-release hypothesis and performed quantum mechanical studies with reliable DFT methods (see the Supporting Information for details).

**Figure 2:**
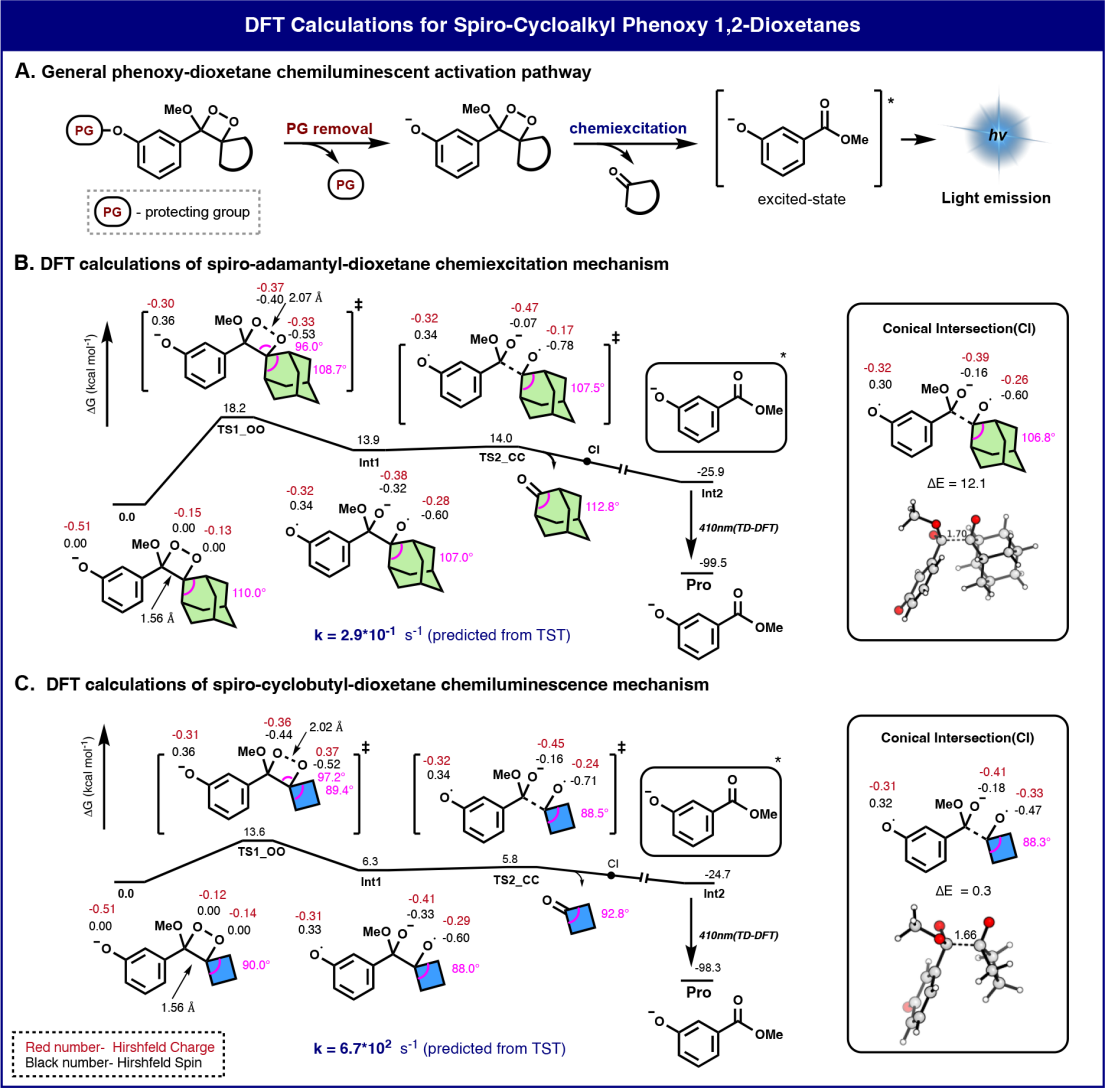
(A) The chemiexcitation pathway of a general spiro-cycloalkyl phenoxy-1,2-dioxetane. Computed Gibbs free energy profiles of (B) spiro-adamantyl phenoxy-1,2-dioxetane chemiexcitation and (C) spiro-cyclobutyl phenoxy-1,2-dioxetane chemiexcitation. Additional DFT calculations are presented in Figures S1–S7 in the Supporting Information.

There have been several previous computational studies of “charge-transfer-induced luminescence” (CTIL) and variations in timing of charge transfer, O–O, and C–C cleavage.^36−38^ The extensive and elegant results by Yue and Liu for the adamantyl compound, studied by us as shown in Figure 2B, showed that the mechanism of dioxetane ring opening involves charge transfer by state crossing during the O–O cleavage transition state.^39^ Our activation barrier is very similar to their results with the CAM-B3LYP/6-31G(d) method. Subsequent C–C cleavage leads in part to the luminescent excited state of the aryl ester. We have previously studied a related chemiluminescent reaction of a heterocycle that forms a dioxetane-oxide and decomposes to generate an excited state aryl ester.^40^

The computational results in Figure 2 indicate that the adamantyl-phenoxy-1,2-dioxetane exhibits a comparatively slow chemiexcitation rate, predicted by the relatively high barrier of 18.2 kcal/mol (Figure 2B) for a transition state with O–O cleavage and partial electron transfer. This becomes more significant in metastable Int1. Very rapid C–C cleavage leads to the formation of the excited state of the metaphenoxide of methyl benzoate. By contrast, the cyclobutyl-phenoxy-1,2-dioxetane shows a chemiexcitation rate with a barrier of only 13.6 kcal/mol (Figure 2C). Ring-opening is again accompanied by electron transfer. Figure 2B,C shows the energetics, essential geometric features along the reaction path, charges, and spin densities on important atoms involved in the reaction. Transition state theory predictions of rate constants from computed activation-free energies are also shown.

The striking predictions by DFT of more than 10^3^ rate acceleration encouraged us to synthesize spirocyclobutyl-phenoxy-1,2-dioxetane along with other cycloalkyl derivatives and evaluate their light emission properties. We initially studied the synthesis and thermal stabilities of dioxetanes, where *tert*-butyldimethyl-silyl (TBS) is the triggering substrate used as a protecting group for the phenol function that can be removed by fluoride ions.

**Figure 3:**
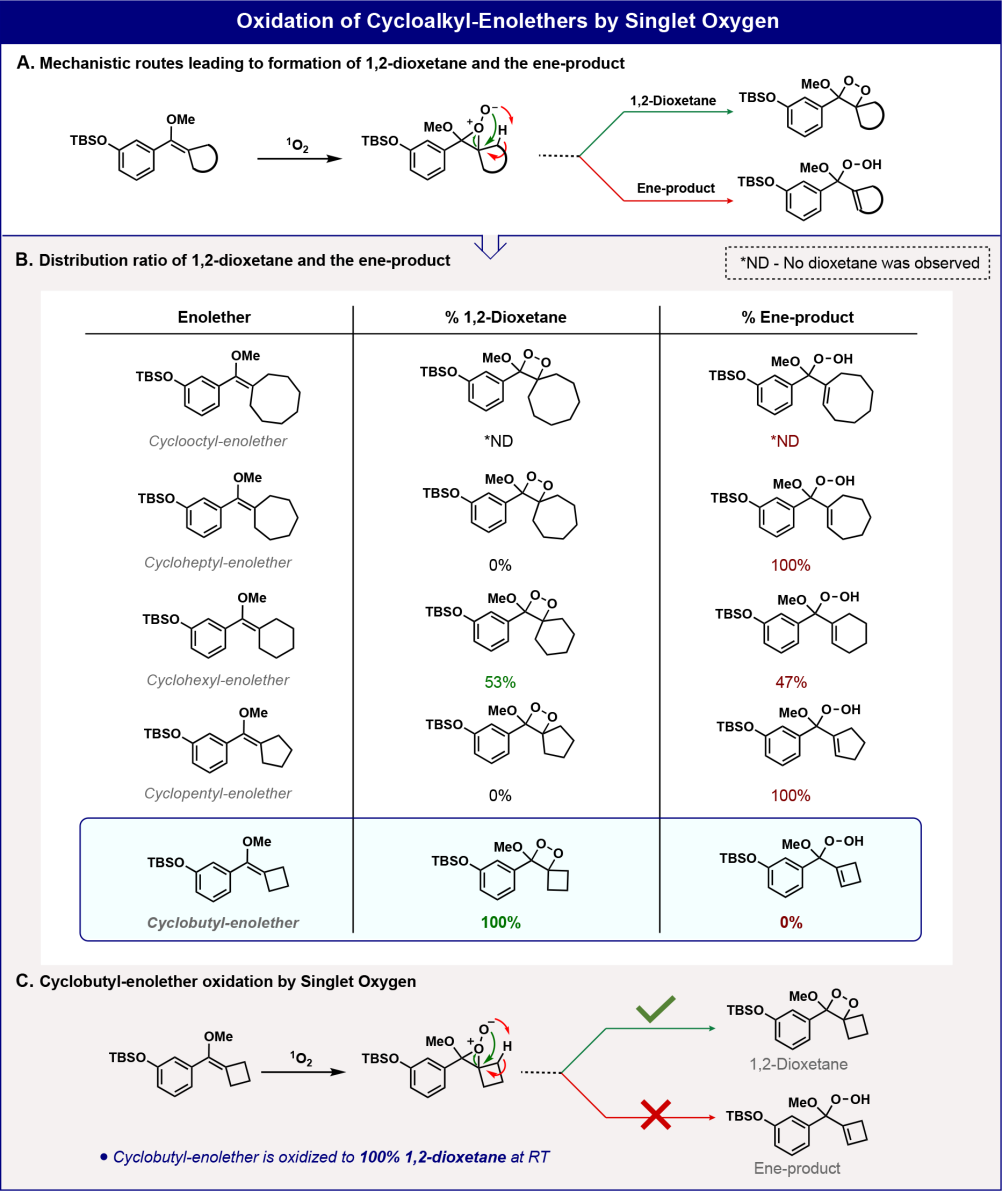
(A) Oxidation of enolethers by singlet oxygen: the ene product vs the 1,2-dioxetane product. (B) Oxidation of different spiro-cycloalkyl phenoxy-1,2-dioxetanes. The product distribution was determined by RP-HPLC. (C) Oxidation of cyclobutyl-enolether occurring exclusively to generate the 1,2-dioxetane product. Measurements were performed in triplicate using independent samples.

To examine the spirostrain release effect, five enol ether dioxetane precursors with cycloalkyl rings of various sizes were synthesized. The last step in the synthesis of phenoxy-1,2-dioxetanes involves oxidation by singlet oxygen of an enolether precursor to a dioxetane. The side product of this oxidation is an ene product, which is obtained through the elimination of a proton positioned at the allylic position of the enolether (Figure 3A).^41^ Interestingly, the oxidation of the cyclopentyl- and cycloheptyl-enolethers resulted in a full formation of the undesired ene product while the cyclohexyl derivative gave about 1:1 ratio of ene and dioxetane products (Figure 3B and Figure S8). The oxidation of the cyclooctyl-enol ether resulted in the formation of a mixture of decomposition products. On the other hand, the oxidation of the cyclobutyl-enolether resulted in full conversion to the dioxetane with no formation of the ene product (Figure 3C). The oxidation of adamantyl-enolether also leads to full conversion to its dioxetane product. Understandably, for both cyclobutyl-enolether and adamantyl-enolether, the formation of the side product is disfavored because the elimination reaction leads to the generation of a highly constrained cyclic alkene.

The chemiexcitation of the cyclobutyl- and cyclohexyl-dioxetanes was then evaluated by the addition of the dioxetanes to a solution of tetrabutylammonium fluoride (TBAF) in DMSO. Remarkably, and in agreement with theoretical predictions, *the spiro-cyclobutyl-dioxetane exhibited a significantly enhanced chemiexcitation rate (more than 100-fold)* in comparison to that of the spiro-adamantyl-dioxetane. The chemiexcitation rate of spiro-cyclohexyl-dioxetane was slightly faster than that of the spiro-adamantyl-dioxetane. The acceleration is about 20-fold less than predicted computationally, which could reflect inaccuracies in the DFT methods or, more likely, the solvation models used in the computations.

Following this encouraging experimental observation, we synthesized a series of spiro-cyclobutyl-dioxetanes and measured their chemiluminescence and stability properties. The molecular structures of 15 different spiro-cycloalkyl-dioxetanes, their relative stability (PBS 7.4, RT), total light emission half-life value (*T*_1/2_ in DMSO or acetone), and relative chemiexcitation rate are presented in Table 1 (rate constants are presented in Figures S17–S19). Adamantyl dioxetane Diox 1 was used as a reference compound for the 13 spirocyclobutyl-dioxetanes (Diox 3–Diox 15). The chemiexcitation of dioxetanes is much faster in polar organic solvents like DMSO. Therefore, the *T*_1/2_ values of total light emission for dioxetanes with a relatively slow chemiexcitation rate were determined in DMSO as a solvent, and those for dioxetanes with a fast chemiexcitation rate measurements were determined in acetone.

**Table 1:**
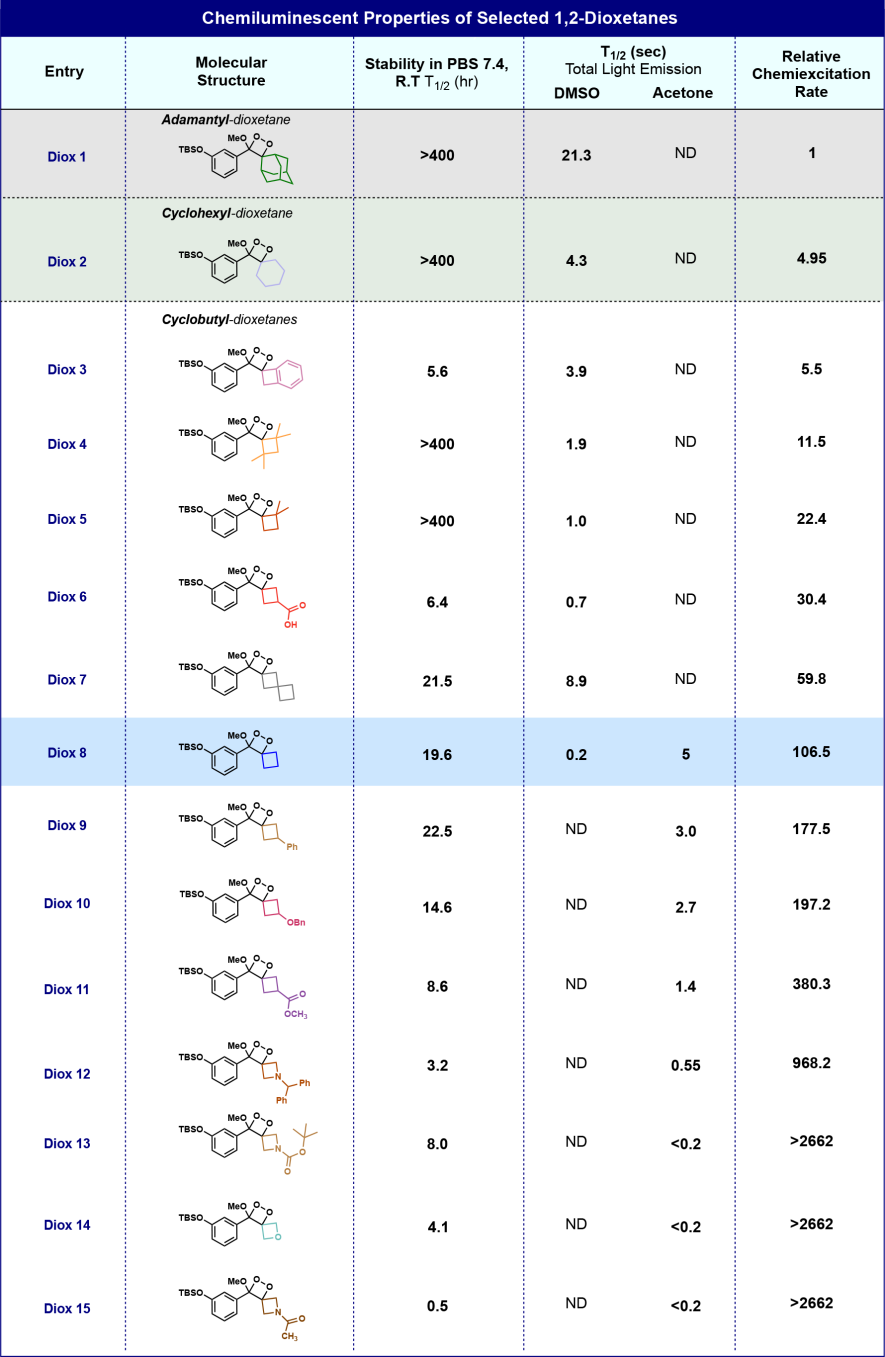
Molecular Structures and Chemiluminescent Properties of Different Spiro-Cycloalkyl Phenoxy-1,2-dioxetanes^a^

a The stability of Diox 1-Diox 15 (500 μM) was measured in PBS (100 mM), pH 7.4, 10% ACN at room temperature; product distribution was determined using RP-HPLC (90-100% ACN in water with 0.1% TFA) (see Figures S9–S12). Chemiexcitation properties of Diox 1–Diox 15 (10 nM) were measured in DMSO or acetone, with TBAF (10 mM) (see Figures S13–S22). The half-life value (*T*_1/2_) is defined as the time point at which half of the total light emission was observed. The relative chemiexcitation rate is defined as the ratio between the *T*_1/2_ values of Diox 1–Diox 15. The *T*_1/2_ value of Diox 1 in DMSO was used as a reference. All measurements were conducted using a SpectraMax iD3 instrument , with injector settings fixed on an integration time of 50 ms. Measurements were performed in triplicate using independent samples.

In general, all cyclobutyl-dioxetanes exhibited significantly faster chemiexcitation rates compared to those of the parent adamantyl-dioxetane. The chemiexcitation rate of the unsubstituted spiro-cyclobutyl-dioxetane, Diox 8, was 106-fold faster than that of the adamantyl-dioxetane, Diox 1. Spiro-dioxetanes with oxetane and azetidine rings exhibited the fastest chemiexcitation with more than 2662-fold rate enhancement (Diox 12–Diox 15), likely the result of further stabilization of the radical anion formed in O–O cleavage and charge transfer (Figure S3). The chemical stabilities of most of the spiro-cyclobutyl-dioxetanes were lower than that of the parent adamantyl derivative. This phenomenon is likely caused by the steric hindrance decrease in the vicinity of the dioxetane unit. Dimethyl- and tetramethyl-substituted cyclobutyl-dioxetanes (Diox 4 and Diox 5), which have larger steric hindrance near the dioxetane, exhibit chemical stability similar to that of the adamantyl derivative but 22-fold and 11-fold higher chemiexcitation rates, respectively.

The slower rate of acceleration of the substituted cyclobutanes than the unsubstituted cyclobutane case is likely due to the reduced electronegativity of the alkyl spirocycle, while greater stability toward decomposition arises from steric hindrance to external attack. Dioxetanes with cyclobutyl units substituted with electron-withdrawing functional groups (Diox 10 and Diox 11) also exhibited higher chemiexcitation rates but reduced chemical stability. Diox 3 and Diox 6 exhibited slower chemiexcitation rates, although their cyclobutane unit is equipped with electron-withdrawing functional groups (benzene ring for Diox 3 and carboxylic acid group for Diox 6). For Diox 6, the negative charge of the carboxylate probably serves as an electron donor. However, the rationale behind this behavior in the case of Diox 6 remains unclear. Since spiro-cyclobutyl-dioxetane, Diox 8, showed a remarkable chemiexcitation acceleration effect (over 100-fold) with moderate but sufficient chemical stability at room temperature (*T*_1/2_ of 20 h), it was therefore selected to further demonstrate the advantage that can be achieved by the acceleration of chemiexcitation.

**Figure 4:**
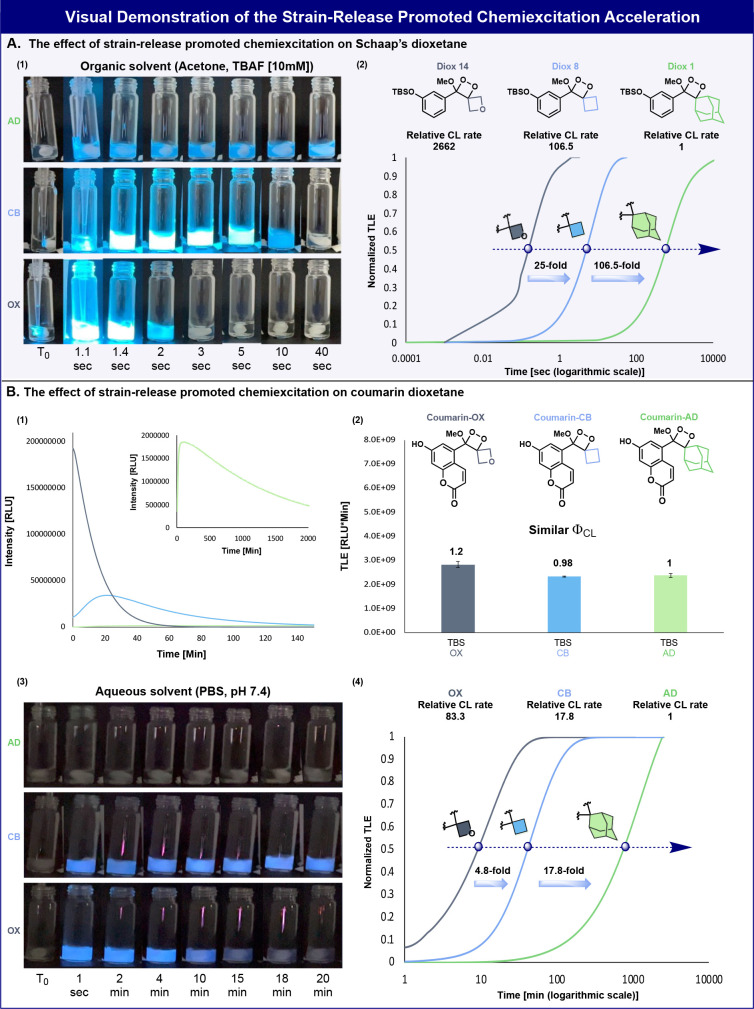
(A) (1) Visual demonstration of the light emitted by Diox 1, Diox 8, and Diox 14 (500 μM) during 40 s in the presence of TBAF [10 mM] in Acetone. (2) Normalized total light emission kinetic profile (time is represented on a logarithmic scale) of Diox 1, Diox 8, and Diox 14. The relative calculated chemiexcitation rates are taken from Table 1. (B) (1) Chemiluminescent kinetic profiles of Coumarin-OX, Coumarin-CB, and Coumarin-AD (1 μM) in PBS, pH 7.4, 10% ACN. (2) Total light emission measured for Coumarin-OX, Coumarin-CB, and Coumarin-AD. (3) Visual demonstration of the light emitted by Coumarin-OX, Coumarin-CB, and Coumarin-AD (1 mM) during 20 min in PBS, pH 7.4, 10% ACN. (4) Normalized total light emission kinetic profiles (time is represented on a logarithmic scale) of Coumarin-OX, Coumarin-CB, and Coumarin-AD (1 μM) in PBS, pH 7.4, 10% ACN. The relative chemiexcitation rate is defined as the ratio between the *T*_1/2_ values of Coumarin-OX, Coumarin-CB, and Coumarin-AD. The *T*_1/2_ value of Coumarin-AD was used as a reference. Measurements were performed in triplicate using independent samples.

A visual validation for the chemiexcitation acceleration effect obtained by the replacement of the adamantyl unit with cyclobutane and oxetane four-membered-ring units is presented in Figure 4. The light emission activation of the three TBS-protected dioxetanes was initiated by addition of the dioxetane to a solution of TBAF in acetone. Figure 4A1 shows images of vials taken at selected time intervals for a period of 40 s. Adamantyl-dioxetane emitted light with a relatively slow chemiexcitation rate that lasted beyond 40 s. Cyclobutyl- and oxetanyl-dioxetanes emitted light with a significantly higher chemiexcitation rate that lasted up to 10 s (*T*_1/2_ = 5 s) and 2 s (*T*_1/2_ = 0.25 s), respectively. The relative chemiexcitation rates of the three dioxetanes were calculated by measuring their total light emission *T*_1/2_ values according to the plots presented in Figure 4A2. Cyclobutyl- and oxetanyl-dioxetanes chemiexcitation rates were respectively 107-fold and 2662-fold faster than that of the adamantyl-dioxetane. Videos of these reactions happening are given in the Supporting Information.

We next sought to demonstrate the chemiexcitation acceleration effect of the adamantyl-dioxetane luminophore with a very slow chemiexcitation rate and high chemiluminescent quantum yield under physiological conditions. Several years ago, we reported such a chemiluminescent dioxetane chemiluminophore composed of a coumarin scaffold.^42^ Coumarin-AD undergoes extremely slow chemiexcitation with a light emission profile that lasts over 50 h. This luminophore exhibited a very high chemiluminescent quantum yield, with a value of 55% under physiological conditions. The analogous derivatives of coumarin-AD were prepared with cyclobutane and oxetane units (coumarin-CB and coumarin-OX; Figure 4B), and their light emission properties were evaluated in PBS 7.4. The chemiexcitation of the coumarin dioxetane luminophores was initiated by the deprotonation of their phenol functional group. Expectedly, coumarin-CB and coumarin-OX exhibited significantly faster light emission profiles compared to coumarin-AD (Figure 4B1). The chemiluminescent quantum yields of all three coumarin dioxetane luminophores were similar (Figure 4B2). Figure 4B3 shows images of vials taken for the three luminophores at selected time intervals for 20 min. The light intensity produced by coumarin-AD is very low (*T*_1/2_ = 750 min) and thus cannot be visualized in the presented images. However, since the chemiexcitation rate of coumarin-CB and coumarin-OX is substantially higher, their light emission is clearly visualized in the images. Coumarin-CB emitted light that was visualized over a time period of more than 20 min, and coumarin-OX emitted similar total light emission within a time slot of only 10 min. The relative chemiexcitation rates of the three coumarin dioxetanes were calculated by their total light emission *T*_1/2_ values according to the plots presented in Figure 4B4. Coumarin-CB and coumarin-OX chemiexcitation rates were respectively 18-fold and 83-fold faster than that of the coumarin-AD. This example classically demonstrates how to transform a glow mode of a chemiluminescent luminophore to a flash mode by the replacement of the adamantyl unit with a four-membered-ring unit.

**Figure 5:**
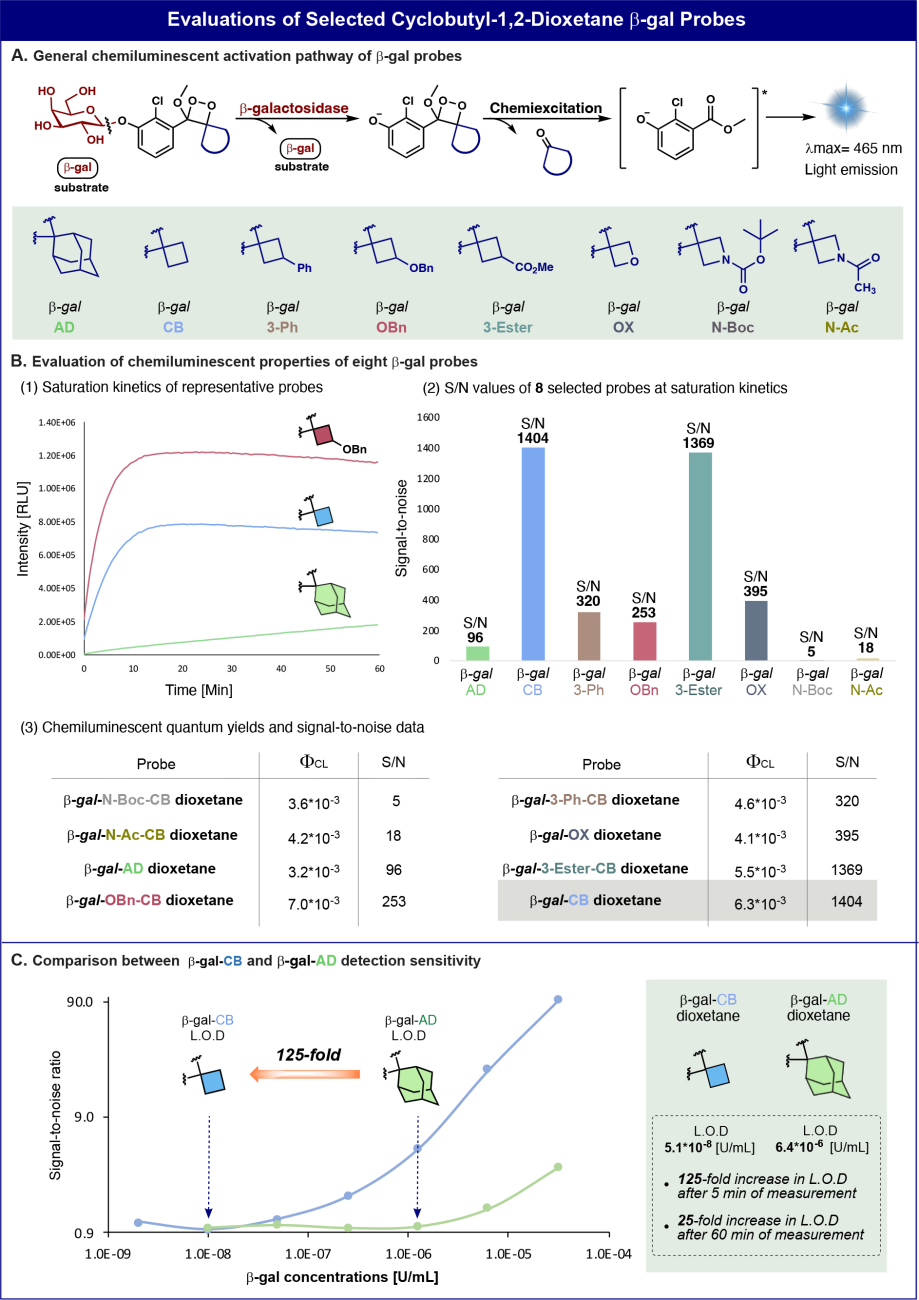
(A) Chemiluminescent activation pathway and molecular structures of eight analogues of spiro-cycloalkyl-1,2-dioxetanes bearing a β-galactosidase-responsive trigger. (B) (1) Chemiluminescent kinetic profiles obtained by probes β-gal-OBn, β-gal-CB, and β-gal-AD. (2) Signal-to-noise ratio of the eight β-gal probes (100 μM) with and without β-galactosidase (4.0 × 10^–3^ U/mL), PB pH 7.4, 1% ACN, 5% Emerald-II enhancer (see Figures S23–S32). The signal-to-noise value is defined as the ratio between the total emitted light during 5 min in the presence and absence of β-galactosidase. (3) Table of the chemiluminescent quantum yields and signal-to-noise values of the eight β-gal dioxetane probes. (C) Determination of the limit of detection for probe β-gal-CB and probe β-gal-AD (100 μM). Measurements were taken with various β-galactosidase concentrations (3.2 × 10^–5^ to 2.0 × 10^–9^ U/mL), 5 min after the addition of the enzyme (see Figures S33 and S34). Measurements were performed in triplicate using independent samples.

Chemiluminescent luminophores with accelerated chemiexcitation produce a larger number of photons per time interval. Therefore, turn-ON probes composed of such luminophores are expected to exhibit a higher detection sensitivity. To evaluate this hypothesis, we synthesized several turn-ON chemiluminescent probes for the detection of β-galactosidase (β-gal) activity. The probes were equipped with β-galactose as a triggering substrate and various four-membered-ring molecular units taken from Table 1. The chemical structures of the probes and their chemiexcitation activation pathway by β-gal are presented in Figure 5A. Since chemiluminescent probes based on the Schaap phenoxy-1,2-dioxetane luminophores exhibit extremely low quantum yields in the presence of water, an Emerald-II enhancer additive is required to amplify their light emission intensity. This enhancer is composed of a polymeric amphiphile detergent and a fluorescein dye additive that can form micelles, which prevent water-induced quenching effects. The probes were incubated with β-gal in PB 7.4 with 5% Emerald-II enhancer, and their light emission signals were recorded under saturation kinetics conditions (low enzyme concentration). Under such conditions, the light emission signal reaches a long-lasting plateau. Probes utilizing chemiluminophores with superior chemiexcitation rates can rapidly reach the plateau stage within a shorter time frame. The light emission profiles over 60 min of two probes with four-membered-ring units are presented in comparison to that of the adamantyl probe (Figure 5B1). The probe β-gal-CB is composed of cyclobutyl-dioxetane, and probe β-gal-OBn has a benzyloxy substituent on the cyclobutyl ring. The S/N values obtained by all probes for the detection of β-gal are presented in Figure 5B2.

Expectedly, the probe β-gal-CB exhibited a light emission signal with a S/N value of 1404, which is significantly higher than the signal produced by the adamantyl-dioxetane, probe β-gal-AD (S/N = 96). Although the absolute signal obtained by probe β-gal-OBn was higher than that of probe β-gal-CB, its S/N value was inferior, with a value of only 253-fold, as a result of a higher background signal (Figure S30). The chemiluminescence quantum yields and the S/N values obtained for all β-gal probes are summarized in the table presented in Figure 5B3. Since probe β-gal-CB exhibited the highest S/N value, its sensitivity for detection of β-gal activity was compared with that of probe β-gal-AD. Remarkably, the LOD (limit of detection) value obtained by probe β-gal-CB was 125-fold more sensitive than the LOD value obtained by its adamantyl analogue, probe β-gal-AD (Figure 5C).

The β-gal probes described above are effective and useful for assays performed in a test tube setup but cannot be applied for *in vitro* and *in vivo* measurements. These probes have an extremely low quantum yield in an aqueous environment and thus must be used in the presence of Emerald-II enhancer, which is highly toxic to live cells. Our *ortho*-substituted acrylate phenoxy-1,2-dioxetanes have a chemiluminescent quantum yield in water, which is up to 3000-fold higher than that of their parent nonsubstituted dioxetanes. This advantage enables the use of such chemiluminescent luminophores as single-component probes with no required additives. Therefore, we next evaluated the chemiexcitation acceleration obtained by the cyclobutyl unit on ortho-acrylate-substituted phenoxy-1,2-dioxetanes.

**Figure 6:**
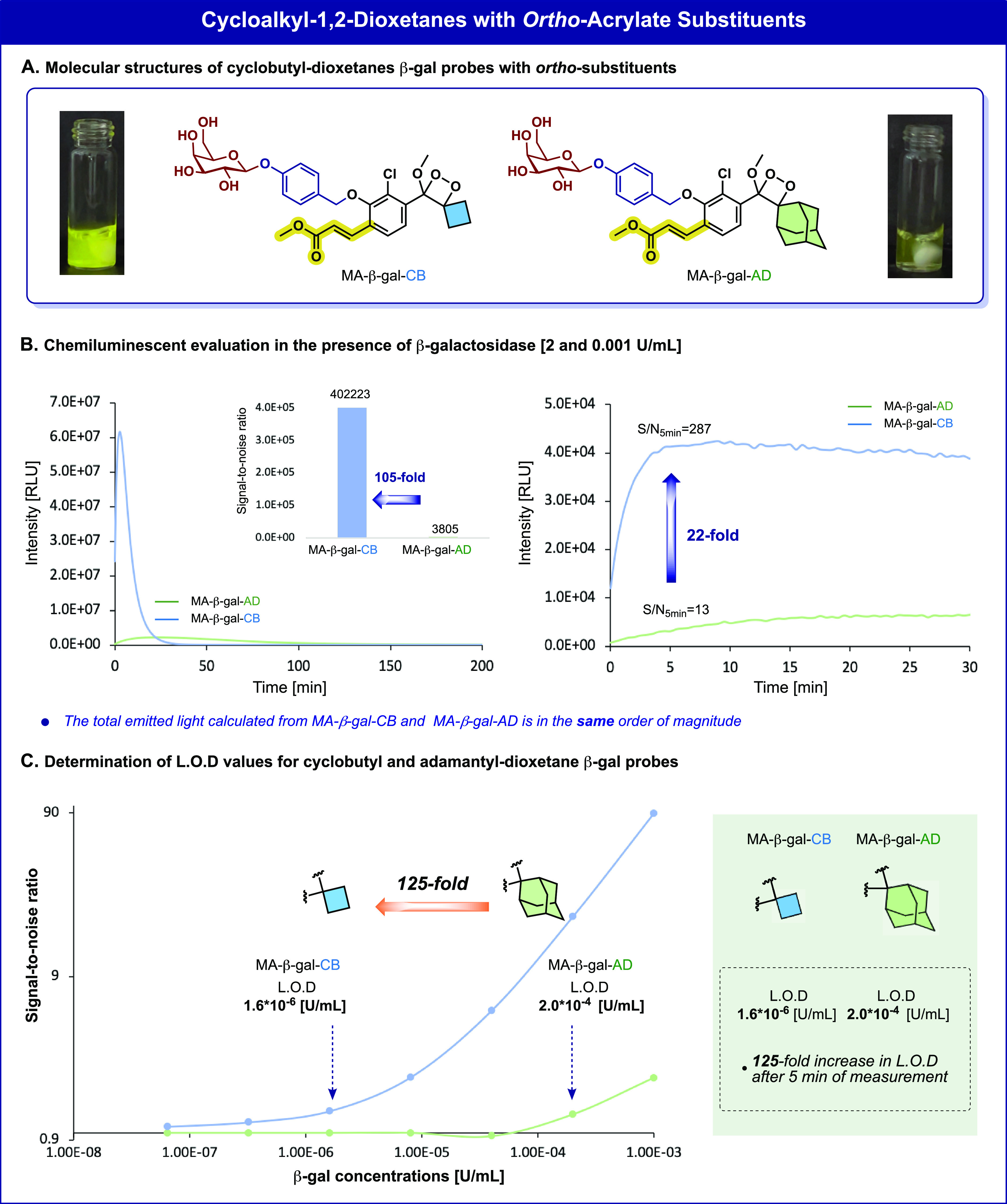
(A) Molecular structures of methyl-acrylate cyclobutyl and adamantyl-phenoxy-1,2-dioxetane-β-gal probes and images of visual demonstration of the light emission obtained by phenoxy methyl-acrylate cyclobutyl-1,2-dioxetane and phenoxy methyl-acrylate adamantyl-1,2-dioxetane (100 μM) in carbonate buffer. (B) Chemiluminescent kinetic profile and signal-to-noise ratio (left) of the total emitted light measured after 5 min by probes MA-β-gal-CB and MA-β-gal-AD (10 μM), with β-gal (2 U/mL), in PBS at pH 7.4, 10% ACN at room temperature. Chemiluminescent kinetic profiles (right) of MA-β-gal-CB and MA-β-gal-AD (10 uM) with and without β-galactosidase (1.0 × 10^–3^ U/mL), PBS pH 7.4, 10% ACN at room temperature. (C) Determination of the limit of detection values of probes MA-β-gal-CB and MA-β-gal-AD (10 μM). Measurements were taken with various β-gal concentrations (1.0 × 10^–3^ to 6.4 × 10^–8^ U/mL), 5 min after the addition of the enzyme in PBS 7.4, 1% ACN at room temperature. See Figures S35–S54 for further results. Measurements were performed in triplicate using independent samples.

A probe for detection of β-gal activity with a methyl acrylate substituent and cyclobutyl unit (probe MA-β-gal-CB) was synthesized and evaluated in comparison to its adamantyl analogue, probe MA-β-gal-CB (Figure 6A). The probes were initially incubated with high-concentration β-gal (2U/mL) in PBS 7.4, and their light emission profiles were measured. Predictably, probe MA-β-gal-CB generates a light emission profile with high intensity that completely decayed after 20 min. On the other hand, the light emission profile of probe MA-β-gal-AD was significantly less intense and lasted over more than 120 min. The S/N value measured for probe MA-β-gal-CB after 5 min was over 400000-fold and about 105-fold higher than the S/N measured for probe MA-β-gal-AD in the same time interval (Figure 6B, left). To our knowledge, this is the highest S/N ratio achieved by a chemiluminescent probe.

The light emission signal of the two probes was then measured under saturation kinetic conditions (low enzyme concentration). Under such conditions, the generated signal is gradually increased to a plateau level (Figure 6B, right). The intensity of the plateau signal produced by probe MA-β-gal-CB was substantially higher than that produced by probe MA-β-gal-AD. An increase of 22-fold was determined for the S/N values obtained for probe MA-β-gal-CB and probe MA-β-gal-AD. The detection sensitivity of probe MA-β-gal-CB for β-gal activity was compared with that of probe MA-β-gal-AD. Similarly, as observed for probes without the acrylate substituent, the LOD (limit of detection) value obtained by probe MA-β-gal-CB was 125-fold more sensitive than the LOD value obtained by its adamantyl analogue, probe MA-β-gal-AD (Figure 6C).

**Figure 7:**
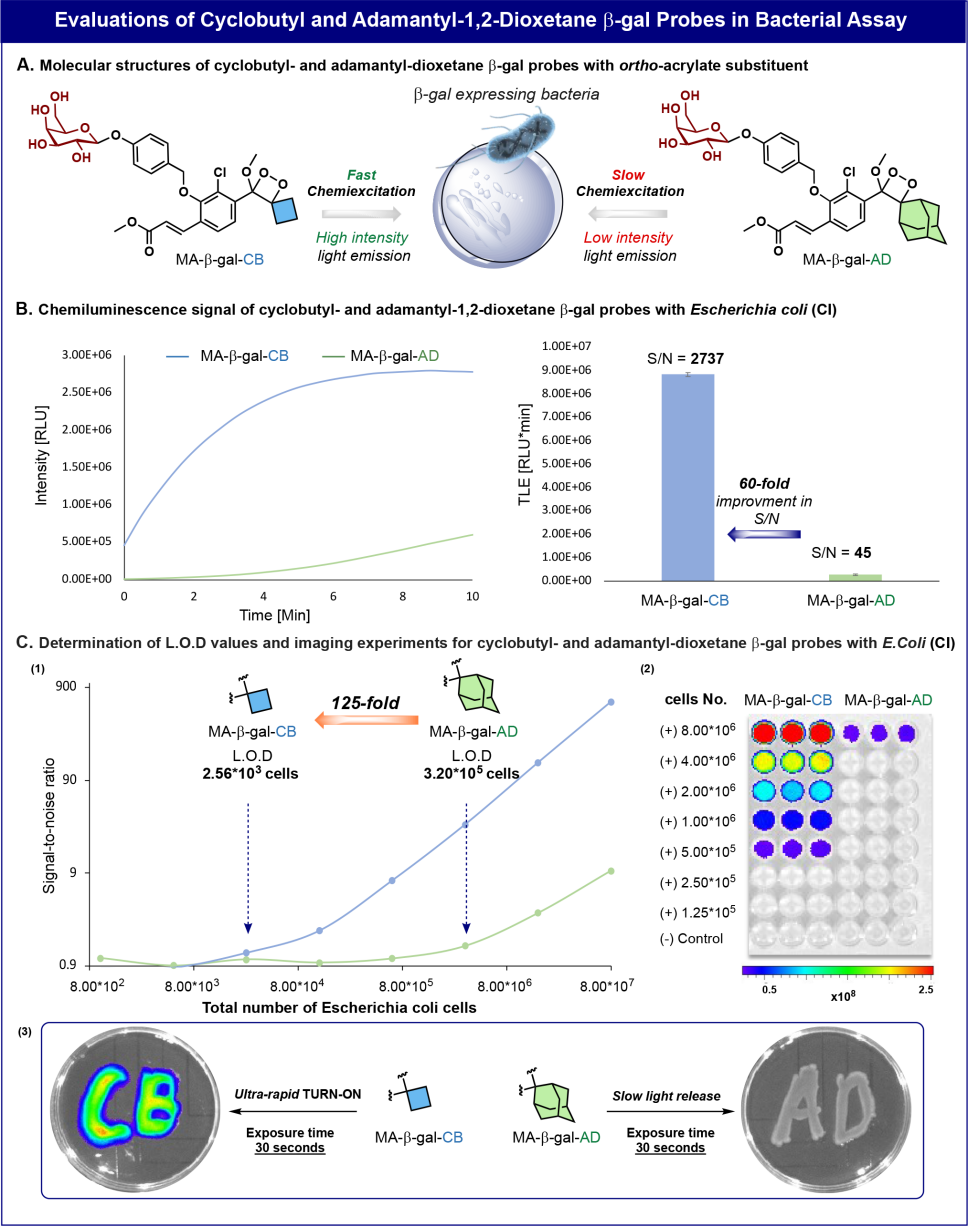
(A) Chemical structures of probes MA-β-gal AD and MA-β-gal CB for evaluation in the bacterial assay. (B) Chemiluminescent kinetic profiles (left) and the total light emitted over 5 min (right) by probes MA-β-gal-CB and MA−β-gal-AD (10 μM), in the presence of *E. coli* (clinical isolate) (4.0 × 10^8^ cells/mL) in PBS, pH 7.4, 1% ACN, at 37 °C. (C) (1) Determination of the limit of detection values for probes MA-β-Gal-CB and MA-β-Gal-AD (10 μM). Measurements were taken with various concentrations of *E. coli* (clinical isolate) (1.0 × 10^3^ to 8.0 × 10^7^ cells/mL), in PBS pH 7.4, 1% ACN at 37 °C, after 5 min using SpectraMax iD3. (2) Images of a dilution series of *E. coli* (1.25 × 10^6^ to 8.00 × 10^7^ cells/mL) taken after 1 min with an IVIS Lumina imager. (3) Chemiluminescent images of *E. coli* colonies in an agar plate sprayed with MA β-Gal-CB or MA β-Gal-AD (100 μM) in PBS pH 7.4, 1% ACN. Images were taken with an IVIS Lumina imager. See Figures S57 and S69 for further results. Measurements were performed in triplicate using independent samples.

The high sensitivity exhibited by probe MA-β-gal-CB toward the detection of β-gal activity stimulated us to evaluate this probe’s ability to detect and image β-gal, in comparison to that of probe MA-β-gal-AD, in live bacterial assays (Figure 7A). Probes MA-β-gal-CB and MA-β-gal-AD were incubated in the presence of *Escherichia coli* (clinical isolate) in PBS, pH 7.4, and the light emission signal was monitored over 10 min. Noticeably, the signal produced by probe MA-β-gal-CB was significantly more intense than the signal produced by probe MA-β-gal-AD (Figure 7B, left). The S/N value obtained by probe MA-β-gal-CB was 60-fold higher than that of probe MA-β-gal-AD (Figure 7B, right). These data clearly demonstrate the superior ability of probe MA-β-gal-CB, over that of probe MA-β-gal-AD, to detect β-gal-expressing bacteria such as *E. coli*.

Probes MA-β-gal-CB and MA-β-gal-AD were then evaluated for their imaging ability to provide bacterial cell images based on their β-gal detection mode. Various concentrations per well of *E. coli* bacteria were incubated in a 96-well plate with probes MA-β-gal-CB and MA-β-gal-AD. After 60 s incubation time, the plate was imaged using an IVIS Lumina imager (Figure 7C2). Under these conditions, probe MA-β-gal-AD was able to image a minimum amount of 8 × 10^6^ bacterial cells, while probe MA-β-gal-CB could image more than a 16-fold lesser number of bacterial cells (5 × 10^5^). Next, we used the two probes to determine the LOD values that could be achieved for the detection of *E. coli* bacterial cells. The LOD value obtained by MA-β-gal-CB was 125-fold more sensitive compared to that of probe MA-β-gal-AD (Figure 7C1). Notably, probe MA-β-gal-CB exhibited an ultrahigh detection sensitivity, capable of detecting as low as 2560 bacterial cells.

The intriguing capability of probe MA-β-gal-CB to rapidly provide images of bacterial cells prompted us to evaluate whether a premade solution of the probe could be potentially used to detect the presence of bacteria by spraying the solution on a contaminated surface. *E. coli* colonies were inoculated on two separated agar plates to shape the letters CB on one plate and AD on the other plate. Solutions of probes MA-β-Gal-CB and MA-β-Gal-AD, in PBS 7.4, were sprayed on the first and the second plates, respectively. After 30 s, the plates were imaged by the IVIS Lumina imager (Figure 7C3). The bacterial colonies sprayed with the probe MA-β-Gal-CB could be clearly visualized by the imager, while the colonies sprayed with the probe MA-β-Gal-AD remained faintly visible. Remarkably, the light emission signal produced by the *E. coli* colonies exposed to a solution of probe MA-β-Gal-CB was 380-fold stronger than the signal obtained from probe MA-β-Gal-AD (Figure S69). This observation effectively demonstrates the significance of the accelerated chemiexcitation rate exhibited by probe MA-β-Gal-CB, compared to the relatively slower rate of probe MA-β-Gal-AD.

Chemiluminescence assays can reach detection sensitivities equivalent to those achieved with radioactive assays because of the extremely low background luminescence of the samples and the reagents.^43^ As previously mentioned, flash-type chemiluminescence assays generate more intense light emission signals in comparison to glow-type assays, primarily because they produce a higher number of photons within a given time interval. Flash assays with rapid kinetics typically necessitate the injection of samples. Indeed, this is the case for assays aimed at the detection of analytes that react with a probe in a stoichiometric manner. However, in our study, we used enzymatic assays under saturation kinetics conditions. Under such conditions, the light emission signal reaches a long-lasting plateau. The signal lasts for several hours, and thus, no sample injection is required.

Previous studies aimed at substituting the adamantyl group of phenoxy-1,2-dioxetanes with alternative groups primarily emphasized enhancing thermal stability, without considering the potential impact on the chemiexcitation rate.^44−47^ The introduction of a spirocyclobutane instead of a spiroadamantane generates a spirostrain effect, causing the corresponding dioxetane to transition from a glow-type light emission mode to a flash-type mode. This effect is directly translated into substantial enhancement of the detection time and the detection sensitivity of the chemiluminescence assay. Given that bioassays involving dioxetane probes are conducted in aqueous environments, the use of probes with improved water solubility offers an obvious advantage. In this context, it has been observed that cyclobutyl-phenoxy-1,2-dioxetanes demonstrate enhanced solubility in water compared to their adamantyl counterparts. This difference in solubility can be attributed to the higher hydrophobicity associated with the adamantane unit (Figures S70–S72).

Even though spiro-cyclobutyl-dioxetanes exhibited lower thermal stability compared to their adamantyl counterparts, their relative chemical stability is adequate for prolonged storage at low temperatures and several hours of usage at room temperature. Structure–activity relationships indicated that the chemical stability of phenoxy-1,2-dioxetanes is compromised in order to gain an enhancement of the chemiexcitation rate. In other words, phenoxy-1,2-dioxetanes with faster chemiexcitation rates are, in general, chemically less stable than dioxetanes with slower chemiexcitation rates. Hence, the quest for a chemiluminophore with both fast chemiexcitation capabilities and exceptional chemical stability continues to pose a noteworthy challenge.

Ever since their discovery, chemists have been cautious about replacing the traditional adamantyl unit of phenoxy-1,2-dioxetanes with alternative substituents, primarily because of concerns related to the chemical stability of the dioxetane and to a mechanistic pathway leading to the formation of an ene product during the oxidation reaction. Our findings revealed that employing a cyclobutyl substituent completely prevents the formation of an ene product, while the obtained dioxetane exhibits satisfactory chemical stability.

The substitution of the spiro-adamantyl dioxetane with either spiro-cyclobutyl- or spiro-oxetanyl-dioxetane led to minor alterations in the chemiluminescent quantum yield of the dioxetane luminophores. As is evident from Figures 4B and 6B, the spiro-cyclobutyl-phenoxy-1,2-dioxetane presented about a 2-fold increase in total light emission intensity compared to the spiro-adamantyl counterpart. In general, turn-ON probes composed of a phenoxy-1,2-dioxetane chemiluminophore with a cyclobutyl unit, instead of an adamantyl unit, exhibited a substantial enhancement in detection sensitivity, with an observed increase ranging from 10- to 100-fold. The detection sensitivity enhancement effect was demonstrated by both Schaap’s dioxetanes and by dioxetanes with an *ortho*-acrylate substituent under physiological conditions. Therefore, they currently hold the record for the most sensitive chemiluminescent probes in terms of the signal-to-noise ratio. The remarkable acceleration effect observed in the chemiexcitation rate of phenoxy-1,2-dioxetanes, achieved through the incorporation of a cyclobutyl substituent, presents a fascinating opportunity to explore new avenues in the design of even brighter 1,2-dioxetane chemiluminophores.

## Conclusions

In summary, we discovered a distinct spirostrained molecular motif that enables efficient enhancement of the chemiexcitation rate of phenoxy-1,2-dioxetane luminophores. The chemiexcitation rate of dioxetane luminophores was significantly accelerated through a spiro-strain-release effect generated by a spiro-cyclobutane-dioxetane fusion. Computations provided support for the hypothesis, and experimental results confirmed the accelerated chemiexcitation rate of the spirocyclobutyl-dioxetanes compared to its parent adamantyl-dioxetane. Remarkably, spiro-dioxetanes containing cyclobutyl and oxetanyl moieties exhibited chemiexcitation rates that were 107-fold and 2662-fold faster, respectively, when compared to spiro-adamantyl-dioxetane. The accelerated chemiexcitation rate of the new dioxetane luminophores enables us to substantially increase the detection sensitivity of known chemiluminescent probes. A turn-ON probe for the detection of the enzyme β-gal, containing a spiro-cyclobutyl unit, exhibited a limit of detection value that is 125-fold more sensitive than that obtained by previously described adamantyl analogues. This probe was also able to instantly detect and image β-gal activity with enhanced sensitivity in *E. coli* bacterial assays. The findings described in this study contribute to the development of improved chemiluminescent probes and highlight the importance of strain-release strategies in enhancing the detection sensitivity of chemiluminescence assays. Overall, the discovery of the spiro-cyclobutyl-phenoxy-1,2-dioxetane luminophore enabled the preparation of chemiluminescent probes, which exhibit the highest signal-to-noise ratio documented so far, with unprecedented detection sensitivity.

## Supporting Information Available

Experimental procedures, compound characterization data, and bioassay information (PDF)
Visual demonstration of the light emission effects observed through chemiluminescent luminophores with accelerated chemiexcitation (MP4)
Visual demonstration of the light emission effects observed through chemiluminescent luminophores with accelerated chemiexcitation (MP4)
