# Supplementary material for: Spirostrain-Accelerated Chemiexcitation of Dioxetanes Yields Unprecedented Detection Sensitivity in Chemiluminescence Bioassays
Source: ACS Cent Sci. 2023 Oct 24;10(1):28–42. doi: 10.1021/acscentsci.3c01141 (PMC10823517; doi:10.1021/acscentsci.3c01141)
Supplement: Supplementary file 1 — oc3c01141_si_001.pdf [file oc3c01141_si_001.pdf]

Supporting Information

Spirostrain-Accelerated Chemiexcitation of Dioxetanes Yields Unprecedented Detection Sensitivity in Chemiluminescence Bioassays

Rozan Tannous[a#], Omri Shelef[a#], Sara Gutkin[a#], Maya David[a], Thomas Leirikh[a], Liang Ge[a], Qais Jaber[a], Qingyang Zhou[b], Pengchen Ma[b,c], Micha Fridman[a], Urs Spitz[d], Kendall N. Houk[b]*, and Doron Shabat[a]*

[a]School of Chemistry, Raymond and Beverly Sackler Faculty of Exact Sciences, Tel-Aviv University, Tel Aviv 69978 Israel. [b]Department of Chemistry and Biochemistry, University of California, Los Angeles, California 90095, United States. [c]Department of Chemistry, School of Chemistry, Xi'an Key Laboratory of Sustainable Energy Material Chemistry and Engineering Research Center of Energy Storage Materials and Devices, Ministry of Education, Xi'an Jiaotong University, Xi'an 710049, China. [d]BIOSYNTH, Rietlistr. 4 Postfach 125 9422 Staad, Switzerland.

[#] These authors contributed equally

***Corresponding Authors:**

Doron Shabat, Email: chdoron@tauex.tau.ac.il

Kendall N. Houk, Email: houk@chem.ucla.edu

Table of Contents

General methods S3

Synthesis and characterization S4-S47

TBS-masked 1,2-dioxetanes S5-S21

General synthetic procedures A and B S5

Synthesis of TBS-masked Diox 1,2 and Ene-product 1-3 .... S6-S9

Synthesis of Cyclobutyl derivatives: TBS-masked Diox 3-15 .... S10-S21

Coumarin 1,2-dioxetanes S22-27

General synthetic procedures C, D, and E S22-S24

Synthesis of hydroxy coumarin 1,2-dioxetane S25-S27

β-gal-masked 1,2-dioxetanes S28-S48

General synthetic procedures F, G, and H S28-S29

Synthesis of β-gal-masked 1,2-dioxetanes S30-S41

Synthesis of β-gal-masked-acrylate 1,2-dioxetanes S42-S48

Experimental protocols S49-S51

Bacterial experiments S52

Appendix I- Computational data S53-S68

Appendix II - Supplementary figures S70-S109

Appendix III – Spectral data of compounds S110-S249

References S250-S251

General methods

All reactions requiring anhydrous conditions were performed under an Argon atmosphere. All reactions were carried out at room temperature unless stated otherwise. Chemicals and solvents were either A.R. grade or purified by standard techniques. Thin-layer chromatography (TLC): silica gel plates Merck 60 F254: compounds were visualized by irradiation with UV light. Column chromatography (FC): silica gel Merck 60 (particle size 0.040-0.063 mm), eluent given in parentheses. Reverse-phase high-pressure liquid chromatography (RP-HPLC): C18 5u, 250x4.6mm, eluent given in parentheses. Preparative RP-HPLC: C18 5u, 250x21mm, eluent given in parentheses. ^{1}H-NMR spectra were measured using Bruker Avance operated at 400MHz. ^{13}C-NMR spectra were measured using Bruker Avance operated at 100 MHz. Chemical shifts were reported in ppm on the δ scale relative to a residual solvent ($CDCl_3$: δ = 7.26 for ^{1}H-NMR and 77.16 for ^{13}C-NMR, DMSO-d_6: δ = 2.50 for ^{1}H-NMR and 39.52 for ^{13}C-NMR and ACN-d_3: δ = 2.04 for ^{1}H-NMR and 29.8 for ^{13}C-NMR). Mass spectra were measured on Waters Xevo TQD. Chemiluminescence was recorded on Molecular Devices Spectramax id3. Fluorescence was recorded on Tecan Infinite 200 Pro. All general reagents, including salts and solvents, were purchased from Sigma-Aldrich. Light irradiation for photochemical reactions: LED PAR38 lamp (19W, 3000K).

Abbreviations

ACN- Acetonitrile, **Ac_2O**- Acetic anhydride, **Alloc**- N-Allyloxycarbonyl, **n-BuLi**- N-Butyllithium, **$CHCl_3$**- Chloroform, **DCM**- Dichloromethane, **DIPEA**- N,N-Diisopropylethylamine, **DMAP**- 4-Dimethylaminopyridine, **DMBA**- 1,3-Dimethylbarbituric acid, **DMF**- N,N'–Dimethylformamide, **EtOAc**- Ethyl acetate, **Et_3N**– Triethylamine, **Hex**- Hexanes, **LDA**- Lithium diisopropylamide, **MB**- Methylene blue, **MeOH**- Methanol, **$P(OMe)_3$**- Trimethyl phosphite, **TBAF**– Tetra-n-butylammonium fluoride, **TBS**– Tert-butyldimethylsilyl, **THF**- Tetrahydrofuran, **TIPS**– Triisopropylsilyl, **TMS-Cl**- Trimethylsilyl chloride, **TFA** - Trifluoroacetic acid.

Synthesis and Characterization

Synthesis and characterization of the cycloalkyl-ketone (I and II)

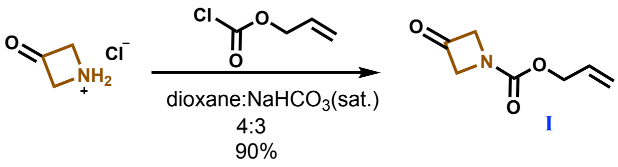

Compound I

To a solution of 3-Azetidinone hydrochloride (500 mg, 4.67 mmol) in (5 mL) of dioxane:NaHCO3 (saturated) mixture (4:3), allyl chloroformate (494 μL, 4.67 mmol) was added. The reaction was monitored by TLC (70:30, Hex:EtOAc). Upon completion, the reaction mixture was diluted with EtOAc and washed with 1M HCl followed by an additional wash with brine. The organic phase was dried over Na_2SO_4, filtered, and concentrated under reduced pressure. The crude residue was purified by column chromatography to obtain Compound **I** (651 mg, 90% yield) in the form of yellow oil.

^{1}H NMR (400 MHz, $CDCl_3$) δ 6.00 – 5.86 (m, 1H), 5.37 – 5.20 (m, 2H), 4.77 (s, 4H), 4.64 – 4.60 (m, 2H).

^{13}C NMR (101 MHz, $CDCl_3$) δ 195.67, 156.27, 132.28, 118.32, 71.28, 66.62.

MS (ES+): m/z calc. for $C_7H_9NO_3$: 155.06; found: 154.0 $[M+H]^+$.

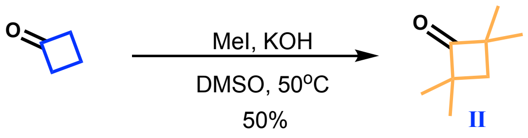

Compound II

Compound **II** was synthesized according to a known procedure from cyclobutanone.[1]

General synthetic procedures for the formation of TBS-masked Diox 1-15 and Ene-product 1-3

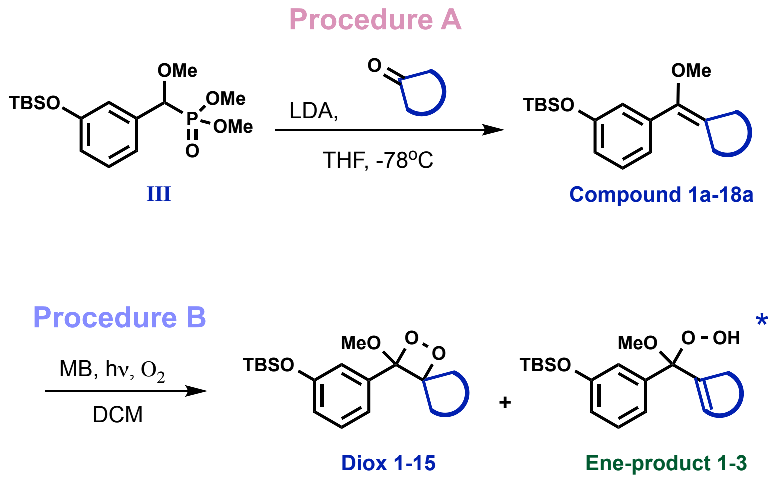

* Ene-product was **not** obseverd for adamantyl and cyclobutyl derivatives

Diox 1 was synthesized according to a known procedure.[2] All compounds were synthesized according to general **procedures A** and **B**.

***Procedure A -** Wittig-Horner reaction*

Phosphonate II[2] (1.0 equiv.) was dissolved in anhydrous THF under argon atmosphere at -78°C. LDA (1.2 equiv.) was added dropwise, and the solution was stirred for 20 minutes. Cycloalkyl ketone (1.2 equiv.) was added, and after 10 minutes of stirring at -78°C, the reaction was warmed to room temperature and stirred for additional 20 minutes. The reaction was monitored by TLC (Hex:EtOAc mixture). Upon completion, the reaction mixture was diluted with EtOAc and washed with brine. The organic phase was dried over Na_2SO_4, filtered, and concentrated under reduced pressure. Purification of the crude residues by column chromatography (Hex:EtOAc mixture) afforded the desired enolethers (compound **1a-17a**).

***Procedure B –** Oxidation reaction*

Enolethers (Compound 1a-17a) (~0.02-0.1 mmol) were dissolved in 5 mL of DCM followed by the addition of a catalytic amount of methylene blue. The solution was cooled to 0°C. Then, oxygen was bubbled through the solution while irradiating with yellow light. The reaction was monitored by TLC (Hex:EtOAc mixture) or RP-HPLC (gradient of 90-100% ACN in water, 0.1% TFA). Upon completion (about 5 min) the crude product was immediately passed through a silica gel column (Hex:EtOAc mixture) to filter out methylene blue. The solvents were removed under reduced pressure while cooling the bath to 10°C. If needed, the compounds were further purified by RP-HPLC using 100% ACN as an eluent to obtain Diox **1-15** or Ene-product **1-3** as products.

Synthesis and characterization of Diox 1,2 and ene-product 1-3

Cyclooctyl derivative

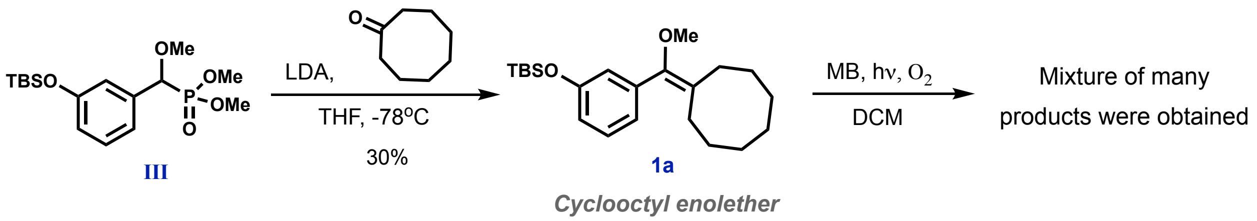

Cyclooctyl enolether (Compound 1a)

Compound **1a** was synthesized according to **Procedure A**, using phosphonate III[2] (300 mg, 0.83 mmol) in dry THF (1.5 mL), LDA (2.0M in THF, 0.5mL, 1.00 mmol) and cyclooctanone (131 μL, 126 mg, 1.00 mmol). The crude residue was purified by column chromatography (90:10, Hex:EtOAc) to afford compound **1a** in the form of a yellow oil (90 mg, 0.25 mmol, 30%).

^{1}H-NMR (400 MHz, $CDCl_3$) δ 7.20 (t, *J* = 7.9 Hz, 1H), 6.89 (d, *J* = 7.6 Hz, 1H), 6.80 – 6.75 (m, 2H), 3.27 (s, 3H), 2.38 – 2.33 (m, 2H), 2.12 – 2.08 (m, 2H), 1.78 – 1.66 (m, 2H), 1.59 – 1.51 (m, 6H), 1.51 – 1.42 (m, 2H), 0.98 (s, 9H), 0.19 (s, 6H).

^{13}C-NMR (101 MHz, $CDCl_3$) δ 155.48, 148.44, 137.41, 129.03, 123.82, 123.07, 121.55, 119.49, 56.74, 31.32, 28.51, 28.08, 27.74, 26.60, 26.01, 25.83, 24.78, 18.37, -4.30.

Oxidation of cyclooctyl enolether (Compound 1a)

Compound **1a** (10 mg, 0.03 mmol) was reacted according to **Procedure B**. The reaction was monitored by RP-HPLC (90-100% ACN in water, 0.1%TFA) and TLC (90:10, Hex:EtOAc). Several by-products were obtained in the oxidation reaction (see TLC in figure S7) without conclusive proof for the formation of spiro cyclooctyl 1,2-dioxetane.

Cycloheptyl derivative

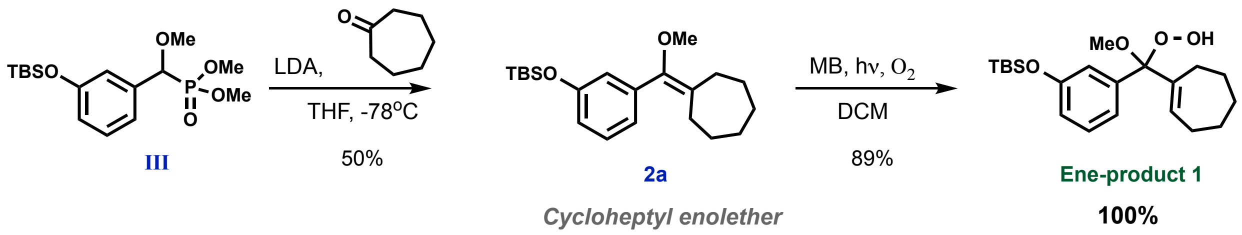

Cycloheptyl enolether (Compound 2a)

Compound **2a** was synthesized according to **Procedure A**, using phosphonate III[2] (300 mg, 0.83 mmol) in dry THF (1.5 mL), LDA (2.0M in THF, 0.5mL, 1.00 mmol), and cycloheptanone (118 μL, 112 mg, 1.00 mmol). The crude residue was purified by column chromatography (90:10, Hex:EtOAc) to afford compound **2a** in the form of a yellow oil (144 mg, 0.42 mmol, 50%).

^{1}H-NMR (400 MHz, $CDCl_3$) δ 7.20 (t, *J* = 7.8 Hz, 1H), 6.91 (dt, *J* = 7.6, 1.2 Hz, 1H), 6.81 – 6.76 (m, 2H), 3.29 (s, 3H), 2.49 – 2.44 (m, 2H), 2.19 – 2.14 (m, 2H), 1.67 (m, 2H), 1.60 – 1.48 (m, 6H), 0.99 (s, 9H), 0.20 (s, 6H).
^{13}C-NMR (101 MHz, $CDCl_3$) δ 155.41, 149.03, 137.26, 128.97, 124.55, 122.96, 121.49, 119.50, 57.13, 30.78, 30.17, 29.34, 29.15, 29.06, 27.82, 25.83, 18.36, -4.29.
MS (ES+): m/z calc. for $C_{21}H_{34}O_2Si$: 346.23; found: 347.5 $[M+H]^+$.

Ene-product 1

Compound **2a** (10 mg, 0.03 mmol) was reacted according to **Procedure B**. The oxidation reaction yielded the ene-product in 100% conversion. The crude product was purified by column chromatography (90:10, Hex:EtOAc) to afford Ene-product **1** in the form of a yellow oil (9.7 mg, 89% yield).
^{1}H-NMR (400 MHz, $CDCl_3$) δ 7.44 (s, 1H), 7.19 (dd, *J* = 7.6, 1.6 Hz, 1H), 7.07 (dd, *J* = 7.6, 1.6 Hz, 1H), 6.96 (t, *J* = 1.6 Hz, 1H), 6.77 (ddd, *J* = 8.0, 2.5, 1.0 Hz, 1H), 6.37 (t, *J* = 6.7 Hz, 1H), 3.25 (s, 3H), 2.24 – 2.17 (m, 2H), 2.03 – 1.98 (m, 2H), 1.69 – 1.59 (m, 2H), 1.48 – 1.40 (m, 2H), 1.21 – 1.11 (m, 2H), 0.97 (s, 9H), 0.18 (s, 6H).
^{13}C-NMR (101 MHz, $CDCl_3$) δ 155.59, 142.04, 139.99, 130.29, 129.07, 120.15, 120.02, 119.02, 107.71, 50.25, 32.61, 29.83, 28.62, 28.37, 26.60, 26.47, 25.85, -4.26.

Cyclohexyl derivative

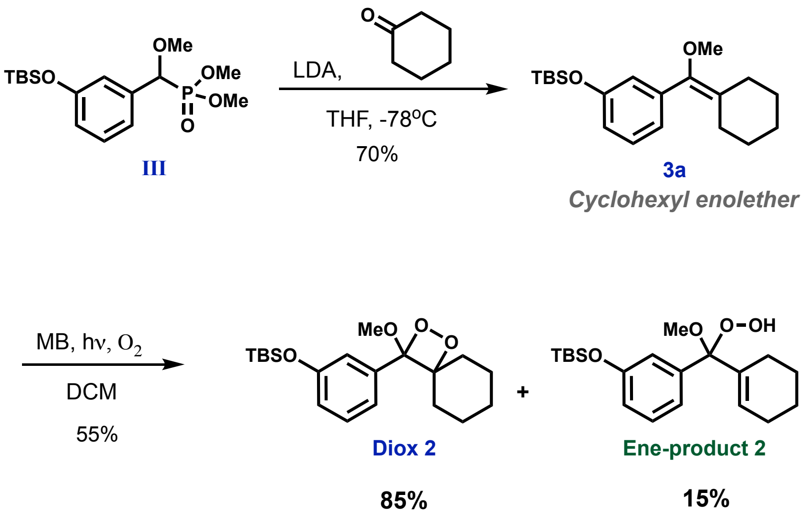

Cyclohexyl enolether (Compound 3a)

Compound **3a** was synthesized according to **Procedure A**, using phosphonate III[2] (300 mg, 0.83 mmol) in dry THF (1.5 mL), LDA (2.0M in THF, 0.5mL, 1.00 mmol) and cyclohexanone (103 µL, 98 mg, 1.00 mmol). The crude residue was purified by column chromatography (90:10, Hex:EtOAc) to afford compound **3a** in the form of a yellow oil (193 mg, 0.58 mmol, 70%).

^{1}H-NMR (400 MHz, CDCl$_3$) δ 7.20 (t, *J* = 7.7 Hz, 1H), 6.90 (d, *J* = 7.6 Hz, 1H), 6.81 – 6.75 (m, 2H), 3.28 (s, 3H), 2.39 (t, *J* = 5.5 Hz, 2H), 2.09 (t, *J* = 5.6 Hz, 2H), 1.63 – 1.52 (m, 4H), 1.52 – 1.44 (m, 2H), 0.98 (s, 9H), 0.20 (s, 6H).
^{13}C-NMR (101 MHz, CDCl$_3$) δ 155.44, 146.58, 136.91, 129.00, 123.48, 122.97, 121.51, 119.54, 57.54, 30.03, 28.28, 28.00, 27.51, 27.05, 25.83, 18.36, -4.30.
MS (ES+): m/z calc. for $C_{20}H_{32}O_2Si$: 332.22; found: 333.5 $[M+H]^+$.

Diox 2 and Ene-product 2

Compound **3a** (30 mg, 0.09 mmol) was reacted following **Procedure B**, and monitored by RP-HPLC (90-100% ACN in water, 0.1%TFA). The oxidation reaction yielded a mixture of Diox 2 and Ene-product 2 in a 85:15 ratio, respectively. The crude residue was purified by column chromatography (90:10, Hex:EtOAc) to filter out methylene blue followed by preparative RP-HPLC (100% ACN as eluent) to obtain **Diox 2** in the form of a yellow oil (18 mg, 0.03 mmol, 55%).
^{1}H-NMR (400 MHz, CDCl$_3$) (Diox 2) δ 7.28 (t, *J* = 7.9 Hz, 1H), 7.10 – 7.04 (m, 1H), 6.98 – 6.92 (m, 1H), 6.87 (ddd, *J* = 8.1, 2.5, 1.0 Hz, 1H), 3.16 (s, 2H), 2.36 – 2.25 (m, 1H), 1.97 – 1.87 (m, 1H), 1.81 – 1.72 (m, 1H), 1.55 (d, *J* = 3.8 Hz, 1H), 1.51 – 1.35 (m, 4H), 1.25 – 1.13 (m, 2H), 0.98 (s, 9H), 0.20 (s, 6H).
^{13}C-NMR (101 MHz, CDCl$_3$) (Diox 2) δ 156.03, 136.66, 129.60, 121.15, 120.76, 119.43, 112.28, 92.32, 50.16, 33.62, 30.57, 25.80, 24.88, 21.58, 21.40, 18.37, -4.27.

^{1}H NMR (400 MHz, CDCl$_3$) (Ene-product 2) δ 7.41 (s, 1H), 7.19 (t, *J* = 7.9 Hz, 1H), 7.07 (ddd, *J* = 7.8, 1.5, 1.1 Hz, 1H), 6.95 (t, *J* = 2.2 Hz, 1H), 6.78 (ddd, *J* = 8.0, 2.5, 1.0 Hz, 1H), 6.19 – 6.14 (m, 1H), 3.27 (s, 3H), 2.13 – 2.07 (m, 2H), 1.79 – 1.69 (m, 2H), 1.55 – 1.46 (m, 4H), 0.97 (s, 9H), 0.18 (s, 6H).
^{13}C NMR (101 MHz, CDCl$_3$) (Ene-product 2) δ 155.64, 140.37, 135.90, 129.12, 125.75, 120.00, 119.74, 118.70, 107.26, 50.02, 25.85, 25.14, 24.31, 22.64, 22.20, 18.40, -4.28.

Cyclopentyl derivative

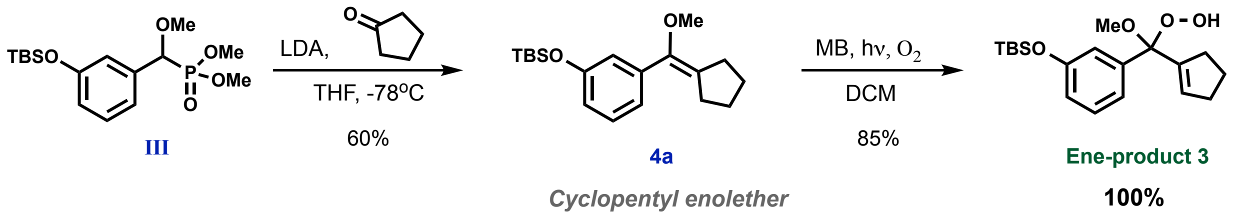

Cyclopentyl enolether (Compound 4a)

Compound **4a** was synthesized according to **Procedure A**, using phosphonate III[2] (300 mg, 0.83 mmol) in dry THF (1.5 mL), LDA (2.0M in THF, 0.5mL, 1.00 mmol) and cyclopentanone (88 μL, 84 mg, 1.00 mmol). The crude residue was purified by column chromatography (90:10, Hex:EtOAc) to afford compound **4a** in the form of a yellow oil (159 mg, 0.50 mmol, 60%).

^{1}H NMR-(400 MHz, CDCl$_3$) δ 7.20 (t, *J* = 7.9 Hz, 1H), 7.00 (d, *J* = 7.7 Hz, 1H), 6.89 (t, *J* = 1.8 Hz, 1H), 6.74 (dd, *J* = 8.0, 2.3 Hz, 1H), 3.42 (s, 3H), 2.50 (t, *J* = 6.6 Hz, 2H), 2.37 (t, *J* = 6.3 Hz, 2H), 1.74 – 1.62 (m, 4H), 0.99 (s, 9H), 0.21 (s, 6H).

^{13}C NMR-(101 MHz, CDCl$_3$) δ 155.54, 146.05, 137.74, 129.02, 128.88, 121.27, 119.67, 119.06, 57.61, 31.27, 29.64, 27.67, 26.16, 25.83, 18.35, -4.25.

MS (ES+): m/z calc. for $C_{19}H_{30}O_2Si$: 318.20; found: 319.5 $[M+H]^+$.

Ene-product 3

Compound **4a** (20 mg, 0.06 mmol) was reacted according to **Procedure B** and monitored by RP-HPLC (90-100% ACN in water, 0.1%TFA). The oxidation reaction yielded the ene-product in 100% conversion. The crude residue was purified by column chromatography (90:10, Hex:EtOAc) to afford **Ene-product 3** in the form of a yellow oil (19 mg, 85% yield).

^{1}H NMR (400 MHz, CDCl$_3$) δ 7.64 (brs, 1H), 7.20 (t, *J* = 7.9 Hz, 1H), 7.08 (dt, *J* = 7.6, 1.2 Hz, 1H), 6.97 (t, *J* = 2.2 Hz, 1H), 6.78 (ddd, *J* = 8.0, 2.5, 1.0 Hz, 1H), 6.01 – 5.95 (m, 1H), 3.31 (s, 3H), 2.38 (ddd, *J* = 9.9, 4.9, 2.4 Hz, 2H), 2.11 (dtd, *J* = 7.0, 4.9, 2.3 Hz, 2H), 1.84 – 1.74 (m, 2H), 0.97 (s, 9H), 0.18 (s, 6H).

^{13}C NMR (101 MHz, CDCl$_3$) δ 155.70, 142.06, 140.20, 129.41, 129.23, 120.04, 119.57, 118.60, 106.56, 50.21, 32.95, 32.22, 25.84, 22.92, 18.38, -4.29.

Synthesis and characterization of Diox 3-15

Cyclobutyl derivatives

Oxidation of all cyclobutyl enolether derivatives (Compounds **5a-18a**) yielded **100%** of the 1,2-dioxetane analogs, without any formation of the ene-product as a side product.

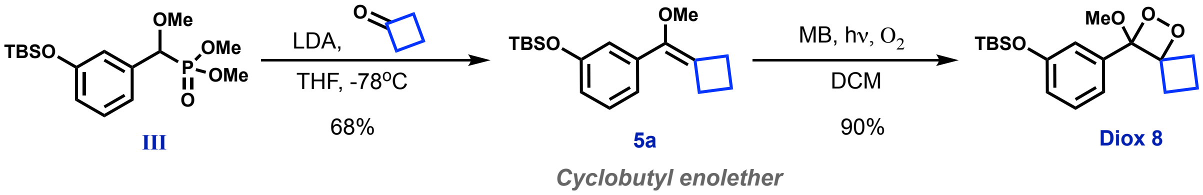

Cyclobutyl enolether (Compound 5a)

Compound **5a** was synthesized according to **Procedure A**, using phosphonate III[2] (300 mg, 0.83 mmol) in dry THF (1.5 mL), LDA (2.0M in THF, 0.5mL, 1.00 mmol) and cyclobutanone (74 μL, 70 mg, 1.00 mmol). The crude residue was purified by column chromatography (90:10, Hex:EtOAc) to afford compound **5a** in the form of a yellow oil (172 mg, 0.56 mmol, 68%).

^{1}H-NMR (400 MHz, CDCl$_3$) δ 7.18 (t, *J* = 7.9 Hz, 1H), 6.95 (d, *J* = 7.8 Hz, 1H), 6.84 (t, *J* = 1.8 Hz, 1H), 6.70 (dd, *J* = 8.0, 1.8 Hz, 1H), 3.56 (s, 3H), 2.94 (t, *J* = 7.8 Hz, 4H), 2.06 (quin, *J* = 7.8 Hz, 2H), 0.98 (s, 9H), 0.20 (s, 6H).

^{13}C-NMR (101 MHz, CDCl$_3$) δ 155.69, 146.21, 136.83, 129.21, 125.64, 119.46, 118.84, 117.84, 58.75, 30.90, 29.00, 25.82, 18.33, 18.20, -4.26.

MS (ES+): m/z calc. for $C_{18}H_{28}O_2Si$: 304.19; found: 305.5 $[M+H]^+$.

Diox 8

Compound **5a** (15 mg, 0.05 mmol) was reacted according to **Procedure B**. Reaction was monitored by RP-HPLC (90-100% ACN in water, 0.1%TFA). Upon completion (5 min), the crude product was purified by column chromatography (90:10, Hex:EtOAc) to afford **Diox 8** in the form of a yellow oil (15 mg, 90% yield).

^{1}H-NMR (400 MHz, CDCl$_3$) δ 7.34 (t, *J* = 7.9 Hz, 1H), 7.18 (ddd, *J* = 8.0, 1.6, 1.2 Hz, 1H), 7.05 (t, *J* = 1.6 Hz, 1H), 6.91 (ddd, *J* = 8.1, 2.5, 1.0 Hz, 1H), 3.35 (s, 3H), 3.08 – 2.97 (m, 1H), 2.23 (dddd, *J* = 14.2, 13.2, 9.8, 5.1 Hz, 2H), 1.87 (tddd, *J* = 8.6, 4.9, 3.6, 1.1 Hz, 1H), 1.67 – 1.55 (m, 1H), 1.34 – 1.21 (m, 1H), 1.01 (s, 9H), 0.23 (s, 6H).

^{13}C-NMR (101 MHz, CDCl$_3$) δ 156.24, 136.85, 129.91, 121.22, 119.76, 118.45, 111.90, 94.68, 50.78, 33.22, 31.09, 25.68, 18.25, 11.06, -4.42.

MS (APPI-): m/z calc. for $C_{18}H_{28}O_4Si$: 336.18; found: 335.2 $[M-H]^-$.

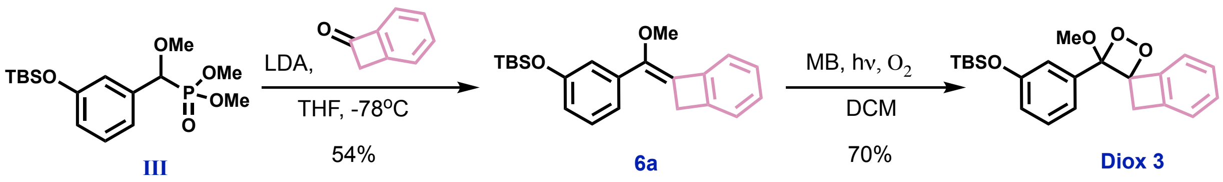

Compound 6a

Compound **6a** was synthesized according to **Procedure A**, using phosphonate III[2] (300 mg, 0.83 mmol) in dry THF (1.5 mL), LDA (2.0M in THF, 0.5mL, 1.00 mmol), and benzocyclobutenone (98 μL, 118 mg, 1.00 mmol). The crude residue was purified by column chromatography (90:10, Hex:EtOAc) to afford compound **6a** in the form of a yellow oil (158 mg, 0.45 mmol, 54%). Compound **6b** was obtained as a diastereomeric mixture of *trans* and *cis* (E:Z) in 2:1 ratio, respectively.

^{1}H-NMR (400 MHz, CDCl$_3$) (major) δ 7.38 – 6.97 (m, 7H), 6.83 (ddd, *J* = 7.9, 2.4, 1.1 Hz, 1H)], 3.88 (s, 2H), 3.84 (s, 3H), 1.05 (s, 9H), 0.28 (s, 6H).

^{1}H-NMR (400 MHz, CDCl$_3$) (minor) δ 7.38 – 6.97 (m, 6H), 6.98 (d, *J* = 6.4 Hz, 1H), 6.89 (ddd, *J* = 7.8, 2.4, 1.3 Hz, 1H), 3.92 (s, 2H), 3.81 (s, 3H), 1.04 (s, 9H), 0.26 (s, 6H).

^{13}C-NMR (101 MHz, CDCl$_3$) (Diastereomeric mixture) δ [155.99, 155.91], [147.69, 146.69], [144.39, 144.33], [144.16, 143.87], [137.38, 136.43], [129.65, 129.48], [128.00, 127.86], [127.31, 127.22], 122.29, 121.29, 120.36, [119.98, 119.95], [119.57, 118.78], 118.30, [59.22, 58.41], [38.00, 37.38], 25.89, 18.43, -4.16.

MS (ES+): m/z calc. for $C_{22}H_{28}O_2Si$: 352.19; found: 353.4 $[M+H]^+$.

Diox 3

Compound **6a** (25 mg, 0.07 mmol) was reacted following **Procedure B**. Reaction was monitored by RP-HPLC (90-100% ACN in water, 0.1%TFA). Upon completion (15 min), the crude product was purified by column chromatography (90:10, Hex:EtOAc) to afford **Diox 3** in the form of yellow oil (19 mg, 70% yield). **Diox 3** was obtained as a diastereomeric mixture in a 1.2:1 ratio.

^{1}H-NMR (400 MHz, CDCl$_3$) (major) δ 7.64 (d, *J* = 6.8 Hz, 1H), 7.40 – 6.80 (m, 7H), 3.37 (s, 6H), 3.37 (d, *J* = 14.7 Hz, 1H), 3.04 (d, *J* = 14.8 Hz, 1H), 0.96 (s, 9H), 0.17 (s, 6H).

^{1}H-NMR (400 MHz, CDCl$_3$) (minor) δ 7.40 – 7.16 (m, 7H), 6.53 (d, *J* = 7.5 Hz, 1H), 4.25 (d, *J* = 15.1 Hz, 1H), 3.54 (d, *J* = 15.1 Hz, 1H), 3.37 (s, 6H), 0.93 (s, 9H), 0.09 (s, 3H), 0.07 (s, 3H).

^{13}C-NMR (101 MHz, CDCl$_3$) (Diastereomeric mixture) δ [156.47, 156.16], [143.12, 142.32], [142.27, 142.16], [137.35, 136.81], [131.93, 131.62], [130.20, 129.83], [127.94, 127.64], 125.18, [123.20, 123.09], 123.05, [121.55, 121.39], [119.89, 119.66], [118.46, 118.29], [97.29, 96.73], [51.17, 50.98], [43.37, 41.58], 25.79, 18.36, [-4.37, -4.44].

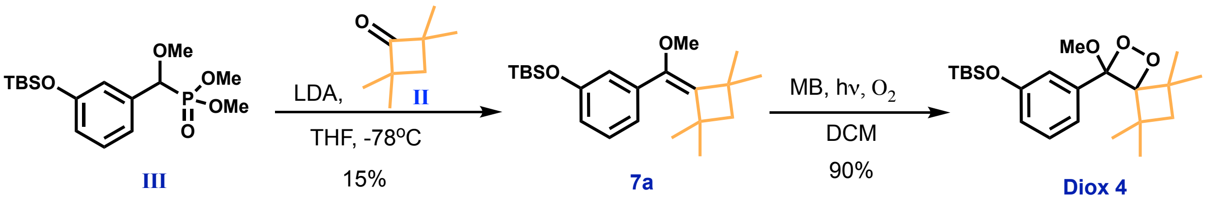

Compound 7a

Compound **7a** was synthesized according to **Procedure A**, using phosphonate III[2] (300 mg, 0.83 mmol) in dry THF (1.5 mL), LDA (2.0M in THF, 0.5mL, 1.00 mmol) and 2,2,4,4-tetramethylcyclobutanone **II** (157 mg, 1.25 mmol). Upon completion, the reaction mixture containing compound **7a** was further separated using preparative RP-HPLC (90-100% ACN in water, 0.1%TFA). Solvents were evaporated with the addition of a few drops of Et_3N to prevent the appearance of side products. Compound **7a** was obtained in the form of a yellow oil (21 mg, 0.06 mmol, 7%).

^{1}H-NMR (400 MHz, $CDCl_3$) δ 7.17 (t, *J* = 7.8 Hz, 1H), 6.93 (td, *J* = 7.6, 1.1 Hz, 1H), 6.84 – 6.82 (m, 1H), 6.77 (ddd, *J* = 8.1, 2.4, 0.9 Hz, 1H), 3.27 (s, 3H), 1.34 (s, 6H), 1.25 (s, 2H), 1.04 (s, 6H), 0.98 (s, 9H), 0.18 (s, 6H).

^{13}C-NMR (101 MHz, $CDCl_3$) δ 155.33, 149.03, 139.74, 136.99, 128.75, 122.92, 121.11, 119.74, 57.19, 48.10, 37.11, 35.66, 29.91, 28.92, 25.81, 18.32, -4.27.

Diox 4

Compound **7a** (10 mg, 0.03 mmol) was reacted following **Procedure B**. The reaction was monitored by RP-HPLC (90-100% ACN in water, 0.1%TFA). Upon completion (15 min), the crude product was purified by column chromatography (90:10, Hex:EtOAc) to afford **Diox 4** in a form of a yellow oil (10 mg, 90% yield).

^{1}H-NMR (400 MHz, $CDCl_3$) δ 7.34 – 7.26 (m, 3H), 6.88 (dd, *J* = 8.9, 2.3 Hz, 1H), 3.19 (s, 3H), 1.49 (s, 3H), 1.43 (d, *J* = 11.3 Hz, 1H), 1.33 (d, *J* = 11.3 Hz, 1H), 1.27 (s, 3H), 1.21 (s, 3H), 1.16 (s, 3H), 1.01 (s, 9H), 0.22 (s, 6H).

^{13}C-NMR (101 MHz, $CDCl_3$) δ 156.12, 137.06, 129.66, 121.41, 121.32, 114.08, 113.47, 101.03, 49.13, 46.39, 39.85, 29.71, 26.34, 25.68, 25.59, 24.96, 24.84, 18.22, -4.34.

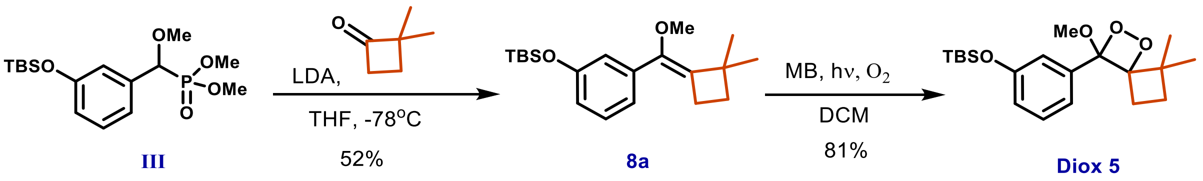

Compound 8a

Compound **8a** was synthesized according to **Procedure A**, using phosphonate III[2] (300 mg, 0.83 mmol) in dry THF (1.5 mL), LDA (2.0M in THF, 0.5mL, 1.00 mmol) and 2,2-dimethylcyclobutanone (98 mg,

1.00 mmol). The crude residue was purified by column chromatography (90:10, Hex:EtOAc) to afford compound **8a** in the form of a yellow oil (143 mg, 0.43 mmol, 52%).

^{1}H NMR (400 MHz, CDCl$_3$) δ 7.17 (t, *J* = 7.8 Hz, 1H), 6.92 (d, *J* = 7.6 Hz, 1H), 6.82 (d, *J* = 1.8 Hz, 1H), 6.77 (ddd, *J* = 8.1, 2.3, 0.6 Hz, 1H), 3.38 (s, 3H), 2.75 (t, *J* = 8.0 Hz, 2H), 1.75 (t, *J* = 8.0 Hz, 2H), 1.06 (s, 6H), 0.99 (s, 9H), 0.19 (s, 6H).

^{13}C NMR (101 MHz, CDCl$_3$) δ 155.43, 147.27, 136.99, 130.71, 128.90, 122.27, 120.49, 119.79, 57.39, 42.51, 33.06, 27.98, 25.81, 23.18, 18.33, -4.25.

Diox 5

Compound **8a** (10 mg, 0.03 mmol) was reacted following **Procedure B**. The reaction was monitored by RP-HPLC (90-100% ACN in water, 0.1%TFA). Upon completion (10 min), the crude product was purified by column chromatography (90:10, Hex:EtOAc) to afford **Diox 5** in a form of a yellow oil (9 mg, 0.02 mmol, 81%).

^{1}H NMR (400 MHz, CDCl$_3$) δ 7.31 – 7.21 (m, 2H), 7.07 (s, 1H), 6.88 (ddd, *J* = 7.8, 2.4, 1.2 Hz, 1H), 3.29 (s, 3H), 2.84 (dt, *J* = 14.1, 9.5 Hz, 1H), 2.28 (ddd, *J* = 13.8, 9.3, 4.2 Hz, 1H), 1.64 – 1.55 (m, 1H), 1.37 – 1.29 (m, 1H), 1.02 (s, 3H), 0.99 (s, 9H), 0.45 (s, 3H), 0.20 (s, 6H).

^{13}C NMR (101 MHz, CDCl$_3$) δ 156.13, 137.19, 129.65, 121.49, 121.19, 119.67, 110.60, 100.22, 50.06, 44.22, 29.39, 27.97, 25.81, 23.88, 22.83, 18.33, -4.22.

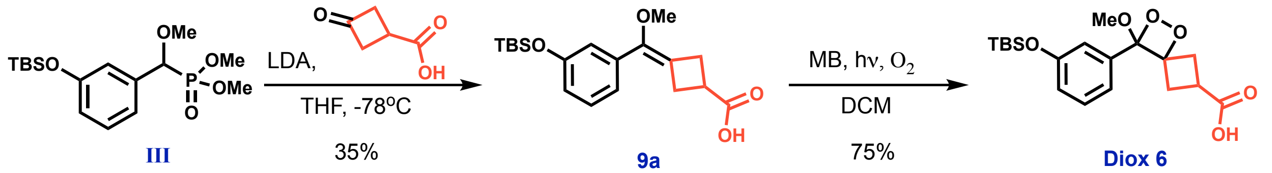

III 9a Diox 6

Compound 9a

Compound **9a** was synthesized according to **Procedure A**, using phosphonate III[2] (600 mg, 1.66 mmol) in dry THF (3 mL), LDA (2.0M in THF, 1.66 mL, 3.33 mmol), and 3-oxocyclobutanecarboxylic acid (190 mg, 1.66 mmol). The crude residue was purified by column chromatography (60:40, Hex:EtOAc) to afford compound **9a** in a form of a sticky yellow oil (202 mg, 0.58 mmol, 35%).

^{1}H-NMR (400 MHz, CDCl$_3$) δ 7.20 (t, *J* = 7.9 Hz, 1H), 6.94 (dd, *J* = 6.4, 1.2 Hz, 1H), 6.83 (t, *J* = 1.8 Hz, 1H), 6.74 (ddd, *J* = 8.1, 2.4, 0.8 Hz, 1H), 3.58 (s, 3H), 3.33 – 3.21 (m, 4H), 3.18 – 3.09 (m, 1H), 0.99 (s, 9H), 0.21 (s, 6H).

^{13}C-NMR (101 MHz, CDCl$_3$) δ 181.35, 155.80, 147.78, 136.14, 129.40, 119.69, 119.43, 118.22, 118.06, 58.72, 34.43, 33.95, 32.52, 25.83, 18.35, -4.22.

MS (ES-): m/z calc. for $C_{19}H_{28}O_4Si$: 348.18; found: 347.5 $[M-H]^-$.

Diox 6

Compound **9a** (20 mg, 0.06 mmol) was reacted following **Procedure B**. The reaction was monitored by RP-HPLC (90-100% ACN in water, 0.1%TFA). Upon completion (10 min), the crude product was

purified by column chromatography (60:40, Hex:EtOAc) to afford **Diox 6** as a diastereomeric mixture (1.3:1 ratio) in the form of a yellow oil (16 mg, 75% yield).

^{1}H-NMR (400 MHz, CDCl$_3$) (major) δ 7.30 (t, *J* = 7.9 Hz, 1H), 7.16 – 7.11 (m, 1H), 7.06 (t, *J* = 2.0 Hz, 1H), 6.93 – 6.87 (m, 1H), 3.34 (s, 3H), 3.30 – 3.22 (m, 1H), 2.71-2.63 (m, 1H), 2.54 – 2.33 (m, 2H), 2.21 (dddd, *J* = 9.5, 6.4, 3.0, 1.6 Hz, 1H), 0.98 (s, 9H), 0.20 (s, 6H).

^{1}H-NMR (400 MHz, CDCl$_3$) (minor) δ 7.35 (t, *J* = 7.9 Hz, 1H), 7.16 – 7.11 (m, 1H), 7.02 (t, *J* = 2.0 Hz, 1H), 6.93 – 6.87 (m, 1H), 3.32 (s, 3H), 3.30 – 3.22 (m, 1H), 2.97 (tt, *J* = 9.8, 6.8 Hz, 1H), 2.54 – 2.33 (m, 2H), 2.16 – 2.09 (m, 1H), 0.99 (s, 9H), 0.21 (s, 6H).

^{13}C-NMR (101 MHz, CDCl$_3$) (Diastereomeric mixture) δ [180.29, 179.47], [156.64, 156.35], [136.25, 135.94], [130.41, 130.03], [121.71, 121.59], [120.14, 119.71], [119.01, 118.60], [111.50, 111.29], [93.90, 90.58], [51.00, 50.96], [36.82, 35.89], [34.67, 33.91], [29.30, 27.67], 25.79, [18.38, 18.35], [-4.28, -4.31].

MS (ES-): m/z calc. for $C_{19}H_{28}O_6Si$: 380.17; found: 379.4 $[M-H]^-$.

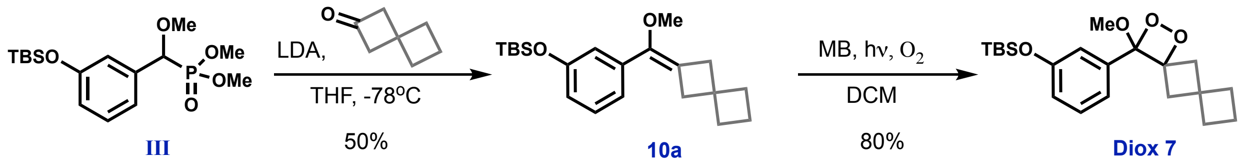

Compound 10a

Compound **10a** was synthesized according to **Procedure A**, using phosphonate III[2] (300 mg, 0.83 mmol) in dry THF (1.5mL), LDA (2.0M in THF, 0.5mL, 1.00 mmol) and spiro[3.3]heptan-2-one (104 μL, 110 mg, 1.00 mmol). The crude residue was purified by column chromatography (90:10, Hex:EtOAc) to afford compound **10a** in a form of a yellow oil (143 mg, 0.41 mmol, 50%).

^{1}H-NMR (400 MHz, CDCl$_3$) δ 7.17 (t, *J* = 7.9 Hz, 1H), 6.94 (d, *J* = 7.8 Hz, 1H), 6.83 (t, *J* = 2.0 Hz, 1H), 6.70 (dd, *J* = 7.7, 2.0 Hz, 1H), 3.55 (s, 3H), 3.10 (qd, *J* = 7.3, 4.8 Hz, 1H), 2.90 (s, 4H), 2.03 (t, *J* = 7.3 Hz, 4H), 1.86 – 1.80 (m, 1H), 0.98 (s, 9H), 0.20 (s, 6H).

^{13}C-NMR (101 MHz, CDCl$_3$) δ 155.68, 146.79, 137.02, 129.16, 120.58, 119.50, 118.82, 117.84, 58.75, 45.91, 43.95, 42.07, 40.48, 35.17, 25.84, 18.34, 16.27, -4.24

Diox 7

Compound **10a** (20 mg, 0.06 mmol) was reacted following **Procedure B**. The reaction was monitored by RP-HPLC (90-100% ACN in water, 0.1%TFA). Upon completion (5 min), the crude product was purified by column chromatography (90:10, Hex:EtOAc) to afford **Diox 7** in a form of a yellow oil (17 mg, 80% yield).

^{1}H-NMR (400 MHz, CDCl$_3$) δ 7.31 (t, *J* = 7.9 Hz, 1H), 7.11 (d, *J* = 7.6 Hz, 1H), 7.01 (s, 1H), 6.88 (d, *J* = 8.0 Hz, 1H), 3.35 (s, 3H), 3.05 (dd, *J* = 13.8, 4.8 Hz, 1H), 2.21 (d, *J* = 13.9 Hz, 1H), 2.14 (d, *J* = 13.7 Hz, 1H), 1.98 – 1.80 (m, 4H), 1.79 – 1.60 (m, 3H), 0.99 (s, 9H), 0.21 (s, 6H).

^{13}C NMR (101 MHz, CDCl$_3$) δ 156.15, 136.77, 129.73, 121.13, 120.08, 118.88, 111.70, 92.28, 50.80, 45.29, 43.17, 34.40, 34.09, 33.79, 25.66, 18.21, 16.27, -4.35.

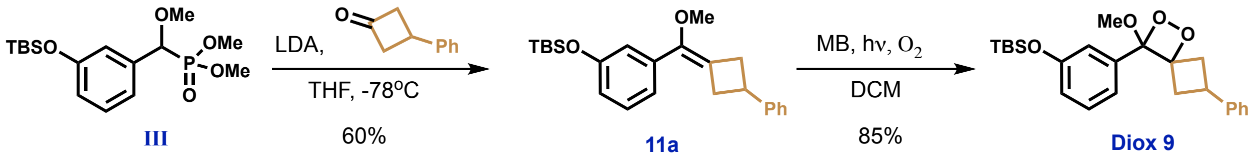

Compound 11a

Compound **11a** was synthesized according to **Procedure A**, using phosphonate III[2] (300 mg, 0.83 mmol) in dry THF (1.5mL), LDA (2.0M in THF, 0.5mL, 1.00 mmol) and 3-phenylcyclobutanone (131 μL, 146 mg, 1.00 mmol). The crude residue was purified by column chromatography (90:10, Hex:EtOAc) to afford compound **11a** in a form of a yellow oil (190 mg, 0.50 mmol, 60%).

^{1}H-NMR (400 MHz, CDCl$_3$) δ 7.42 – 7.35 (m, 4H), 7.30 – 7.23 (m, 2H), 7.08 (d, *J* = 7.8 Hz, 1H), 6.97 (t, *J* = 1.8 Hz, 1H), 6.81 (ddd, *J* = 8.0, 1.6, 0.8 Hz, 1H), 3.75 – 3.69 (m, 1H), 3.67 (s, 3H), 3.53 – 3.36 (m, 2H), 3.23 – 3.08 (m, 2H), 1.07 (s, 9H), 0.28 (s, 6H).

^{13}C-NMR (101 MHz, CDCl$_3$) δ 155.84, 147.14, 145.84, 136.84, 129.39, 128.62, 126.66, 126.32, 120.77, 119.72, 119.17, 118.07, 58.86, 38.85, 36.96, 36.41, 25.93, 18.42, -4.14.

MS (ES+): m/z calc. for $C_{24}H_{32}O_2Si$: 380.22; found: 381.5 $[M+H]^+$.

Diox 9

Compound **11a** (25 mg, 0.06 mmol) was reacted following **Procedure B**. The reaction was monitored by RP-HPLC (90-100% ACN in water, 0.1%TFA). Upon completion (5 min), the crude product was purified by column chromatography (90:10 Hex:EtOAc) to afford **Diox 9** as a diastereomeric mixture (1:1 ratio) in the form of a white solid (23 mg, 85% yield).

^{1}H-NMR (400 MHz, CDCl$_3$) (Diastereomeric mixture) δ 7.39 (t, *J* = 7.9 Hz, 1H), 7.32 – 7.23 (m, 7H), 7.21 – 7.15 (m, 2H), 7.15 – 7.03 (m, 6H), 6.95 (dd, *J* = 8.0, 2.4 Hz, 1H), 6.88 (dd, *J* = 8.0, 2.4 Hz, 1H), 3.55 (ddd, *J* = 12.9, 7.9, 5.2 Hz, 1H), 3.51 – 3.44 (m, 1H), 3.42 (s, 3H), 3.39 (s, 3H), 3.03 (dd, *J* = 14.3, 8.4 Hz, 1H), 2.92 (ddd, *J* = 14.3, 9.2, 4.8 Hz, 1H), 2.82 – 2.72 (m, 1H), 2.68 (td, *J* = 9.2, 4.6 Hz, 1H), 2.37 – 2.27 (m, 3H), 2.04 (dd, *J* = 14.1, 8.3 Hz, 1H), 1.03 (s, 9H), 1.01 (s, 9H), 0.25 (s, 6H), 0.21 (s, 6H).

^{13}C-NMR (101 MHz, CDCl$_3$) (Diastereomeric mixture) δ [156.50, 156.27], [143.85, 143.51], [136.76, 136.53], [130.24, 129.95], 128.84, [128.54, 128.46], [126.79, 126.63], [126.48, 126.43], [126.40, 126.31], [121.55, 121.34], [119.99, 119.93], [118.79, 118.62], [111.95, 111.58], [94.77, 91.01], 51.00, [40.80, 40.01], [38.92, 37.82], [30.60, 28.99], 25.80, [18.38, 18.34], [-4.25, -4.31].

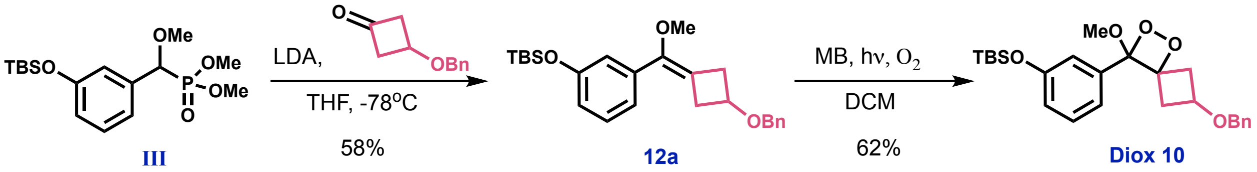

Compound 12a

Compound **12a** was synthesized according to **Procedure A**, using phosphonate III[2] (300 mg, 0.83 mmol) in dry THF (1.5mL), LDA (2.0M in THF, 0.5mL, 1.00 mmol) and 3-(benzyloxy) cyclobutanone (158 μL, 176 mg, 1.00 mmol). The crude residue was purified by column chromatography (90:10, Hex:EtOAc) to afford compound **12a** in the form of a yellow oil (198 mg, 0.48 mmol, 58%).

^{1}H NMR (400 MHz, CDCl$_3$) δ 7.40 – 7.33 (m, 4H), 7.34 – 7.28 (m, 1H), 7.20 (t, *J* = 7.9 Hz, 1H), 6.97 (d, *J* = 7.8 Hz, 1H), 6.85 (t, *J* = 2.0 Hz, 1H), 6.75 (ddd, *J* = 8.0, 2.4, 0.8 Hz, 1H), 4.49 (d, *J* = 1.3 Hz, 2H), 4.19 (quin, *J* = 6.4 Hz, 1H), 3.58 (s, 3H), 3.24 (ddt, *J* = 15.7, 6.8, 2.8 Hz, 1H), 3.12 – 2.98 (m, 2H), 2.96 – 2.88 (m, 1H), 1.01 (s, 9H), 0.22 (s, 6H).

^{13}C NMR (101 MHz, CDCl$_3$) δ 155.72, 148.34, 138.16, 136.71, 129.30, 128.59, 128.10, 127.89, 119.72, 119.28, 118.14, 115.76, 70.62, 70.15, 58.81, 39.26, 37.59, 25.86, 18.36, -4.22.

Diox 10

Compound **12a** (20 mg, 0.06 mmol) was reacted following **Procedure B**. The reaction was monitored by RP-HPLC (90-100% ACN in water, 0.1%TFA). Upon completion (5 min), the crude product was purified by column chromatography (90:10, Hex:EtOAc) to afford **Diox 10** as a diastereomeric mixture (1:1 ratio) in the form of a white solid (13 mg, 62% yield).

^{1}H-NMR (400 MHz, CDCl$_3$) (Diastereomeric mixture) δ 7.37 – 7.27 (m, 9H), 7.26 – 7.23 (m, 3H), 7.15 (d, *J* = 7.8 Hz, 1H), 7.10 – 7.07 (m, 2H), 6.95 (t, *J* = 1.8 Hz, 1H), 6.89 (ddd, *J* = 7.1, 4.7, 1.6 Hz, 2H), 4.32 (s, 2H), 4.28 (s, 2H), 4.04 – 3.96 (m, 1H), 3.46 – 3.39 (m, 2H), 3.37 (s, 3H), 3.30 (s, 3H), 2.91 – 2.84 (m, 1H), 2.73 – 2.65 (m, 1H), 2.49 – 2.41 (m, 1H), 2.29 – 2.16 (m, 3H), 1.97 – 1.89 (m, 1H), 0.99 (s, 9H), 0.99 (s, 9H), 0.20 (s, 12H).

^{13}C-NMR (101 MHz, CDCl$_3$) (Diastereomeric mixture) δ [156.52, 156.29], [137.76, 137.73], [136.64, 136.50], [130.27, 129.95], [128.56, 128.53], [128.17, 128.02], [127.95, 127.89], [121.52, 121.36], [120.08, 119.77], [118.95, 118.45], [111.73, 111.56], [93.08, 88.65], [70.68, 70.57], [66.25, 63.36], [51.01, 50.95], [41.67, 40.73], [39.70, 39.02], 25.81, 18.38, -4.26.

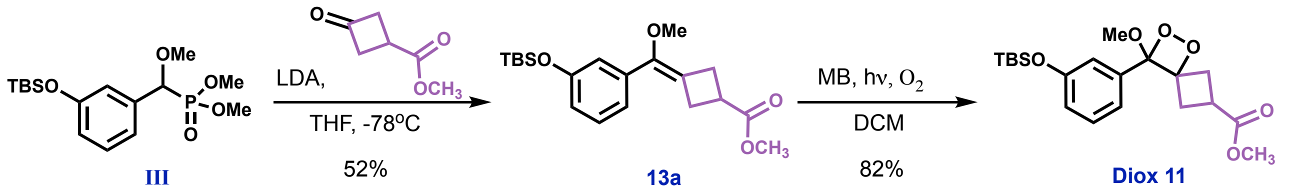

Compound 13a

Compound **13a** was synthesized according to **Procedure A**, using phosphonate III[2] (300 mg, 0.83 mmol) in dry THF (1.5mL), LDA (2.0M in THF, 0.5mL, 1.00 mmol) and methyl 3-oxo-cyclobutane carboxylate (106 µL, 128 mg, 1.00 mmol). The crude residue was purified by column chromatography (90:10, Hex:EtOAc) to afford compound **13a** in a form of a yellow oil (157 mg, 0.43 mmol, 52%).

^{1}H-NMR (400 MHz, $CDCl_3$) δ 7.18 (t, *J* = 7.9 Hz, 1H), 6.93 (d, *J* = 7.8 Hz, 1H), 6.81 (t, *J* = 1.8 Hz, 1H), 6.72 (dd, *J* = 8.0, 2.2 Hz, 1H), 3.72 (s, 3H), 3.56 (s, 3H), 3.29 – 3.16 (m, 4H), 3.08 (dq, *J* = 7.2, 5.0 Hz, 1H), 0.98 (s, 9H), 0.19 (s, 6H).

^{13}C-NMR (101 MHz, $CDCl_3$) δ 175.60, 155.78, 147.64, 136.27, 129.36, 119.65, 119.33, 118.65, 118.02, 58.71, 52.00, 34.38, 34.07, 32.61, 25.82, 18.33, -4.25.

Diox 11

Compound **13a** (20 mg, 0.06 mmol) was reacted following **Procedure B**. The reaction was monitored by RP-HPLC (90-100% ACN in water, 0.1%TFA). Upon completion (5 min), the crude product was purified by column chromatography (90:10, Hex:EtOAc) to afford **Diox 11** as a diastereomeric mixture (1.5:1 ratio) in the form of a yellow oil (18 mg, 82% yield).

^{1}H-NMR (400 MHz, $CDCl_3$) (major) δ 7.37 – 7.28 (m, 1H), 7.15 (dt, *J* = 8.0, 1.0 Hz, 1H), 7.07 (t, *J* = 1.6 Hz, 1H), 6.93 – 6.86 (m, 1H), 3.61 (s, 3H), 3.35 (s, 3H), 3.30 – 3.21 (m, 1H), 2.63 (dddd, *J* = 14.6, 9.8, 3.5, 1.2 Hz, 1H), 2.52 – 2.32 (m, 2H), 2.17 (dddd, *J* = 14.5, 6.6, 3.1, 1.2 Hz, 1H), 0.99 (s, 9H), 0.21 (s, 6H).

^{1}H-NMR (400 MHz, $CDCl_3$) (minor) δ 7.37 – 7.28 (m, 1H), 7.12 (d, *J* = 8.0 Hz, 1H), 7.02 (t, *J* = 1.6 Hz, 1H), 6.93 – 6.86 (m, 1H), 3.63 (s, 3H), 3.32 (s, 3H), 3.30 – 3.21 (m, 1H), 2.93 (tt, *J* = 9.9, 6.7 Hz, 1H), 2.52 – 2.32 (m, 2H), 2.14 – 2.06 (m, 1H), 0.99 (s, 9H), 0.21 (s, 6H).

^{13}C-NMR (101 MHz, $CDCl_3$) (Diastereomeric mixture) δ [174.61, 174.00], [156.46, 156.18], [136.24, 135.99], [130.21, 129.83], [121.49, 121.34], [120.01, 119.58], [118.90, 118.48], [111.43, 111.27], [93.95, 90.56], [52.02, 51.86], 50.84, [36.83, 35.99], [34.66, 33.86], [29.17, 27.52], 25.67, [18.24, 18.22], -4.43.

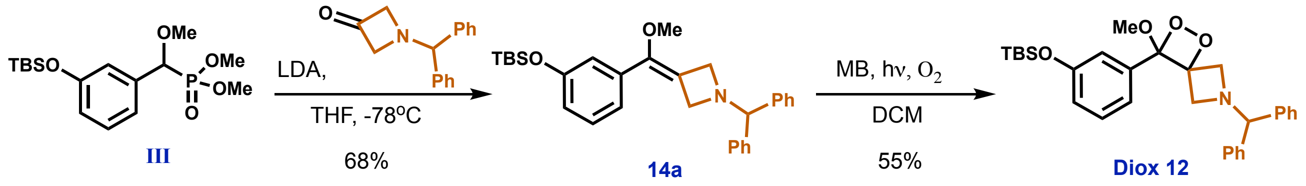

Compound 14a

Compound **14a** was synthesized according to **Procedure A**, using phosphonate III[2] (300 mg, 0.83 mmol) in dry THF (1.5mL), LDA (2.0M in THF, 0.5mL, 1.00 mmol) and 1-benzhydrylazetidin-3-one (237 mg, 1.00 mmol). The crude residue was purified by column chromatography (70:30, Hex:EtOAc) to afford compound **14a** in the form of a white solid (267 mg, 0.57 mmol, 68%).

^{1}H-NMR (400 MHz, $CDCl_3$) δ 7.49 (dd, *J* = 8.0, 1.2 Hz, 4H), 7.31 (t, *J* = 7.5 Hz, 4H), 7.22 (dd, *J* = 7.2, 1.8 Hz, 2H), 7.14 (t, *J* = 7.9 Hz, 1H), 6.84 – 6.79 (m, 2H), 6.73 (ddd, *J* = 8.2, 2.3, 0.9 Hz, 1H), 4.61 (s, 1H), 4.16 (s, 2H), 4.04 (s, 2H), 3.63 (s, 3H), 0.98 (s, 9H), 0.18 (s, 6H).

^{13}C-NMR (101 MHz, $CDCl_3$) δ 155.80, 146.47, 142.42, 136.60, 129.31, 128.72, 127.62, 127.39, 119.64, 119.59, 117.87, 111.80, 77.62, 61.24, 60.20, 58.27, 25.85, 18.38, -4.26.

MS (ES+): m/z calc. for $C_{30}H_{37}NO_2Si$: 471.26; found: 472.6 $[M+H]^+$.

Diox 12

Compound **14a** (20 mg, 0.04 mmol) was reacted following **Procedure B**. The reaction was monitored by RP-HPLC (90-100% ACN in water, 0.1%TFA). Upon completion (7 min), the crude product was purified by column chromatography (70:30, Hex:EtOAc) followed by another purification by RP-HPLC using 100% ACN as eluent to afford **Diox 12** in the form of a white solid (12 mg, 55% yield).

^{1}H-NMR (400 MHz, $CDCl_3$) δ 7.50 (d, *J* = 7.5 Hz, 1H), 7.38 – 7.28 (m, 6H), 7.26 – 7.13 (m, 5H), 7.10 (t, *J* = 1.8 Hz, 1H), 6.92 (ddd, *J* = 8.0, 2.4, 1.4 Hz, 1H), 4.20 (s, 1H), 4.03 (d, *J* = 10.3 Hz, 1H), 3.37 (s, 3H), 3.25 (dd, *J* = 10.3, 1.1 Hz, 1H), 3.12 (dd, *J* = 10.1, 1.1 Hz, 1H), 3.00 (d, *J* = 10.1 Hz, 1H), 1.03 (s, 9H), 0.26 (s, 3H), 0.25 (s, 3H).

^{13}C-NMR (101 MHz, $CDCl_3$) δ 156.22, 141.79, 141.75, 136.34, 129.95, 128.74, 128.50, 128.44, 127.53, 127.25, 127.20, 121.41, 119.65, 118.18, 111.20, 89.95, 77.68, 62.64, 60.77, 50.85, 25.70, 18.25, -4.43.

MS (ES+): m/z calc. for $C_{30}H_{37}NO_4Si$: 503.25; found: 504.5 $[M+H]^+$.

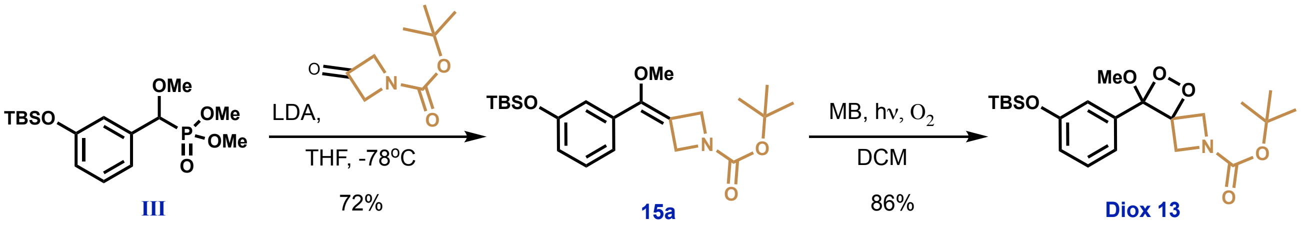

Compound 15a

Compound **15a** was synthesized according to **Procedure A**, using phosphonate III[2] (300 mg, 0.83 mmol) in dry THF (1.5mL), LDA (2.0M in THF, 0.5mL, 1.00 mmol) and 1-Boc-3-azetidinone (171 mg, 1.00 mmol). The crude residue was purified by column chromatography (90:10, Hex:EtOAc) to afford compound **15a** in a form of a yellow oil (243 mg, 0.60 mmol, 72%).

^{1}H-NMR (400 MHz, $CDCl_3$) δ 7.22 (t, *J* = 8.2 Hz, 1H), 6.89 (d, *J* = 7.6 Hz, 1H), 6.81 – 6.77 (m, 2H), 4.78 (s, 2H), 4.68 (s, 2H), 3.66 (s, 3H), 1.48 (s, 9H), 1.00 (s, 9H), 0.21 (s, 6H).

^{13}C-NMR (101 MHz, $CDCl_3$) δ 156.35, 155.94, 147.54, 135.82, 129.63, 120.09, 119.46, 117.95, 79.86, 58.20, 28.52, 25.80, 18.34, -4.27.

MS (ES+): m/z calc. for $C_{22}H_{35}NO_4S$: 405.23; found: 444.22 $[M+K]^+$.

Diox 13

Compound **15a** (20 mg, 0.05 mmol) was reacted following **Procedure B**. The reaction was monitored by RP-HPLC (90-100% ACN in water, 0.1%TFA). Upon completion (7 min), the crude product was purified by column chromatography (90:10, Hex:EtOAc) to afford **Diox 13** in a form of a yellow oil (18 mg, 86% yield).

^{1}H-NMR (400 MHz, $CDCl_3$) δ 7.37 (t, *J* = 7.9 Hz, 1H), 7.16 (d, *J* = 7.7 Hz, 1H), 7.04 (d, *J* = 1.8 Hz, 1H), 6.94 (ddd, *J* = 8.1, 2.4, 0.9 Hz, 1H), 4.67 (dd, *J* = 11.8, 1.8 Hz, 1H), 4.12 (dd, *J* = 11.8, 1.7 Hz, 1H), 3.96 (dd, *J* = 11.4, 1.8 Hz, 1H), 3.60 (dd, *J* = 11.3, 1.6 Hz, 1H), 3.39 (s, 3H), 1.40 (s, 9H), 1.01 (s, 9H), 0.23 (s, 6H).

^{13}C-NMR (101 MHz, $CDCl_3$) δ 156.66, 156.02, 135.68, 130.51, 121.97, 119.60, 118.40, 110.76, 90.06, 80.29, 51.03, 28.36, 25.79, 18.37, -4.29.

MS (ES+): m/z calc. for $C_{22}H_{35}NO_6Si$: 437.22; found: 460.19 $[M+Na]^+$.

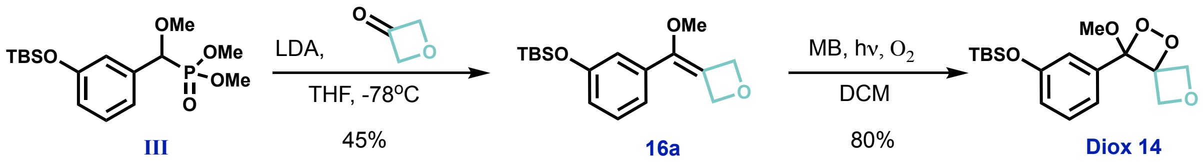

Compound 16a

Compound **16a** was synthesized according to **Procedure A**, using phosphonate III[2] (300 mg, 0.83 mmol) in dry THF (1.5mL), LDA (2.0M in THF, 0.5mL, 1.00 mmol) and 3-oxetanone (64 µL, 72 mg, 1.00 mmol). The crude residue was purified by column chromatography (90:10, Hex:EtOAc), followed by

another purification by RP-HPLC using 100% ACN as eluent to afford compound **16a** in a form of a yellow oil (114 mg, 0.37 mmol, 45%).

^{1}H-NMR (400 MHz, CDCl$_3$) δ 7.19 (t, *J* = 7.9 Hz, 1H), 6.79 (d, *J* = 7.8 Hz, 1H), 6.75 (dd, *J* = 8.1, 1.8 Hz, 1H), 6.70 (t, *J* = 1.8 Hz, 1H), 5.55 (t, *J* = 2.8 Hz, 2H), 5.42 (t, *J* = 2.8 Hz, 2H), 3.65 (s, 3H), 0.98 (s, 9H), 0.19 (s, 6H).

^{13}C-NMR (101 MHz, CDCl$_3$) δ 155.92, 144.83, 135.95, 129.60, 119.97, 119.09, 117.62, 112.93, 78.69, 78.30, 57.81, 25.80, 18.34, -4.27.

Diox 14

Compound **16a** (20 mg, 0.06 mmol) was reacted following **Procedure B**. The reaction was monitored by RP-HPLC (90-100% ACN in water, 0.1%TFA). Upon completion (3 min), the crude product was purified by column chromatography (90:10, Hex: EtOAc) to afford **Diox 14** in a form of a yellow oil (18 mg, 80% yield).

^{1}H-NMR (400 MHz, CDCl$_3$) δ 7.35 (t, *J* = 7.9 Hz, 1H), 7.18 (ddd, *J* = 7.7, 1.5, 1.1 Hz, 1H), 7.07 (t, *J* = 1.8 Hz, 1H), 6.92 (ddd, *J* = 8.1, 2.5, 1.0 Hz, 1H), 5.26 (dd, *J* = 9.3, 1.7 Hz, 1H), 4.81 (dd, *J* = 9.6, 1.5 Hz, 1H), 4.65 (dd, *J* = 9.3, 1.5 Hz, 1H), 4.23 (dd, *J* = 9.1, 1.7 Hz, 1H), 3.38 (s, 3H), 0.99 (s, 9H), 0.21 (s, 6H).

^{13}C-NMR (101 MHz, CDCl$_3$) δ 156.65, 135.72, 130.46, 121.91, 119.57, 118.42, 110.65, 94.04, 79.67, 78.00, 51.01, 25.79, 18.37, -4.28.

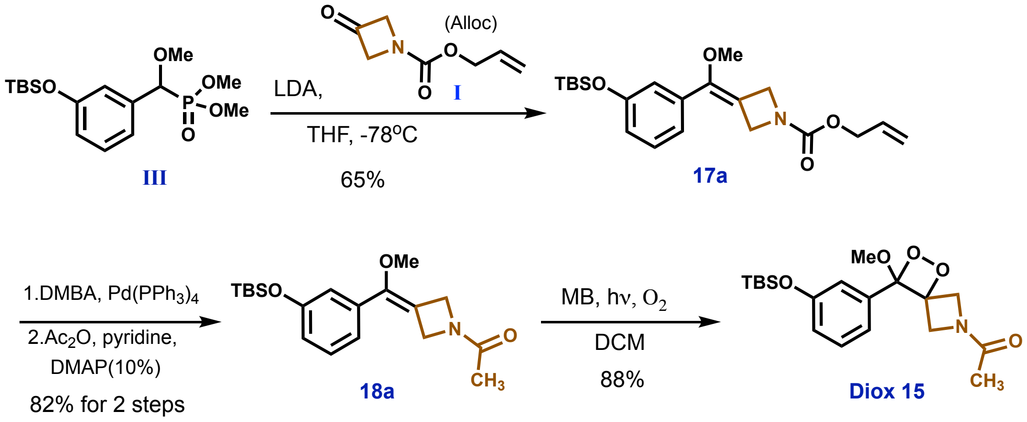

Compound 17a

Compound **17a** was synthesized according to **Procedure A**, using phosphonate III[2] (500 mg, 1.39 mmol) in dry THF (2.5 mL), LDA (2.0M in THF, 0.8 mL, 1.60 mmol) and N-Alloc-3-Azetidinone I (237 mg, 1.53 mmol). The crude residue was purified by column chromatography (70:30, Hex:EtOAc), to afford compound **17a** in a form of a yellow oil (351 mg, 0.90 mmol, 65%).

^{1}H-NMR (400 MHz, CDCl$_3$) δ 7.20 (t, *J* = 8.0 Hz, 1H), 6.86 (d, *J* = 7.8 Hz, 1H), 6.79 – 6.75 (m, 2H), 5.99 – 5.87 (m, 1H), 5.31 (dd, *J* = 17.2, 1.5 Hz, 1H), 5.21 (dd, *J* = 10.4, 1.3 Hz, 1H), 4.84 (t, *J* = 2.9 Hz, 2H), 4.74 (t, *J* = 2.8 Hz, 2H), 4.59 (t, *J* = 1.4 Hz, 1H), 4.58 (t, *J* = 1.4 Hz, 1H), 3.64 (s, 3H), 0.98 (s, 9H), 0.19 (s, 6H).

^{13}C-NMR (101 MHz, $CDCl_3$) δ 156.34, 155.98, 147.86, 135.64, 132.99, 129.66, 120.20, 119.49, 118.00, 117.75, 65.90, 58.19, 25.80, 18.33, -4.25.
MS (ES+): m/z calc. for $C_{21}H_{31}NO_4Si$: 389.20; found: 390.21 $[M+H]^+$.

Compound 18a

To a solution of compound **18a** (300 mg, 0.77 mmol, 1 eq) in DCM (3 mL), DMBA (240 mg, 1.54 mmol, 2 eq), and $Pd(PPh_3)_4$ (92 mg, 0.08 mmol, 0.1 eq) were added. The reaction was stirred at 40°C for 1 hour and monitored by RP-HPLC (70-100% ACN in water, 0.1% TFA). Upon full consumption of starting material, the solvent was removed under reduced pressure, and the crude product was further used without purification. The residue was then dissolved in pyridine (0.66 mL), and acetic anhydride (0.33 mL, 36.35 mmol), and DMAP (9 mg, 0.08 mmol, 0.1 eq) were added. The reaction was monitored by RP-HPLC (90-100% ACN in water, 0.1%TFA). Upon completion, the reaction mixture was purified using preparative RP-HPLC (90-100% ACN in water, 0.1%TFA). The solvents were evaporated under reduced pressure with the addition of a few drops of Et_3N in order to prevent the appearance of side products. compound **18a** was obtained as a diastereomeric mixture (1:1 ratio) in the form of a yellow oil (219 mg, 0.63 mmol, 82% yield).
^{1}H-NMR (400 MHz, DMSO-d6) (Diastereomeric mixture) δ 7.25 (t, *J* = 7.9 Hz, 2H), 6.89 (dd, *J* = 9.2, 8.3 Hz, 2H), 6.79 (d, *J* = 8.0 Hz, 2H), 6.77 – 6.72 (m, 2H), 5.01 (t, *J* = 3.1 Hz, 2H), 4.89 (t, *J* = 3.1 Hz, 2H), 4.71 (t, *J* = 3.0 Hz, 2H), 4.58 (t, *J* = 3.0 Hz, 2H), 3.64 (s, 3H), 3.63 (s, 3H), 1.80 (s, 3H), 1.79 (s, 3H), 0.93 (s, 18H), 0.17 (s, 6H), 0.16 (s, 6H).
^{13}C-NMR (101 MHz, $CDCl_3$) (Diastereomeric mixture) δ [170.47, 170.37], [162.31, 161.95], [156.02, 148.61], [135.68, 135.13], [129.76, 129.71], 120.36, [119.59, 119.51], [118.11, 118.03], [107.56, 105.57], [58.33, 58.14], [58.08, 57.52], [55.67, 55.37], 25.77, 19.05, 18.32, -4.27.
MS (ES+): m/z calc. for $C_{19}H_{29}NO_3Si$: 347.19; found: 348.19 $[M+H]^+$.

Diox 15

Compound **18a** (20 mg, 0.06 mmol) was reacted following **Procedure B**. The reaction was monitored by RP-HPLC (90-100% ACN in water, 0.1%TFA). Upon completion (5 min), the crude product was purified by column chromatography (30:70, Hex:EtOAc) to afford **Diox 15** as a diastereomeric mixture (1:1 ratio) in the form of a yellow oil (19 mg, 88% yield).
^{1}H-NMR (400 MHz, $CDCl_3$) (Diastereomeric mixture) δ 7.39 – 7.32 (m, 2H), 7.15 (d, *J* = 7.7 Hz, 1H), 7.10 (d, *J* = 7.8 Hz, 1H), 7.05 – 7.00 (m, 2H), 6.96 – 6.89 (m, 2H), 4.86 (dd, *J* = 11.4, 1.9 Hz, 1H), 4.73 (d, *J* = 14.5 Hz, 1H), 4.29 (dd, *J* = 11.1, 1.4 Hz, 1H), 4.19 – 4.13 (m, 2H), 3.98 (d, *J* = 12.6 Hz, 1H), 3.74 (dd, *J* = 11.1, 1.9 Hz, 1H), 3.68 (dd, *J* = 12.6, 1.6 Hz, 1H), 3.39 (s, 3H), 3.35 (s, 3H), 1.87 (s, 3H), 1.73 (s, 3H), 0.99 (s, 18H), 0.21 (s, 6H), 0.21 (s, 6H).
^{13}C-NMR (101 MHz, $CDCl_3$) (Diastereomeric mixture) δ [170.50, 170.44], [156.82, 156.78], [135.66, 135.01], 130.67, [122.13, 122.09], [119.55, 119.51], [118.58, 118.32], 110.59, [89.52, 89.40], [60.23, 58.11], [57.67, 55.97], [51.18, 51.07], 25.78, [19.23, 19.18], 18.37, -4.28.
MS (ES+): m/z calc. for $C_{19}H_{29}NO_5Si$: 379.18; found: 380.5 $[M+H]^+$.

General synthetic procedures of the Coumarin 1,2-dioxetanes

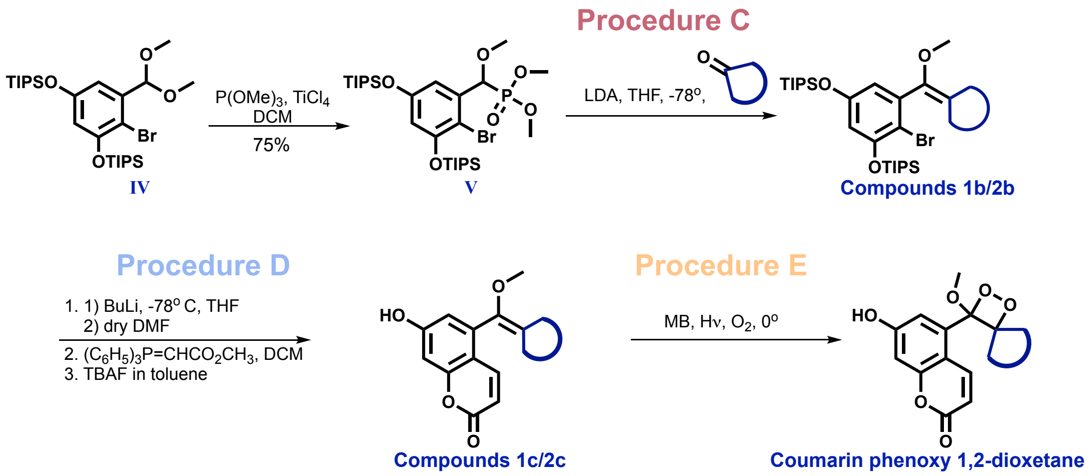

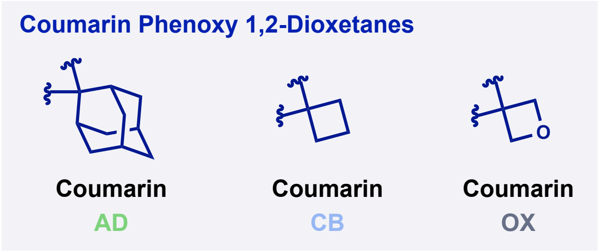

Probe **Coumarin-AD** was synthesized according to known procedure.[3]

Compound IV

Compound **IV** was synthesized according to previously reported procedure, with minor modification (using TIPS instead of TBS as a protecting group).[3]

^{1}H NMR (400 MHz, CDCl$_3$) δ 6.78 (d, J = 2.7 Hz, 1H), 6.46 (d, J = 2.9 Hz, 1H), 5.55 (s, 1H), 3.34 (s, 6H), 1.28 (m, 6H), 1.13 (d, J = 7.4 Hz, 18H), 1.08 (d, J = 7.4 Hz, 18H).

^{13}C NMR (101 MHz, CDCl$_3$) δ 155.58, 153.44, 138.42, 112.67, 111.46, 107.44, 102.89, 53.57, 17.97, 17.84, 13.02, 12.63.

MS (ES+): m/z calc. for $C_{27}H_{51}BrO_4Si_2$: 574.25; found: 597.6 [M+Na]$^+$.

Compound V

compound IV (10.0 gr, 17.36 mmol) and trimethyl phosphite (3.6 mL, 26.0 mmol) were dissolved in DCM (100 mL). The solution was cooled to 0°C and then $TiCl_4$ was added dropwise (2.0 mL, 18.2 mmol). The reaction was monitored by TLC (60:40, Hex:EtOAc). Upon completion (5 min), the solution was poured into a mixture of a saturated solution of $NaHCO_3$ (200 mL) and DCM (200 mL) at 0°C with vigorous stirring. The phases were separated, and the organic layer was washed with brine (100 mL), dried over Na_2SO_4, and evaporated under reduced pressure. The residue was purified by column chromatography on silica gel (60:40, Hex:EtOAc) to afford **compound V** in a form of a colorless oil (8.5 gr, 75% yield).

^{1}H-NMR (400 MHz, $CDCl_3$) δ 6.83 (t, J = 2.6 Hz, 1H), 6.46 (dd, J = 2.8, 1.7 Hz, 1H), 5.18 (d, J = 15.8 Hz, 1H), 3.74 (d, J = 10.6 Hz, 3H), 3.62 (d, J = 10.5 Hz, 3H), 3.31 (s, 3H), 1.33 – 1.19 (m, 6H), 1.12 (d, J = 3.2 Hz, 9H), 1.10 (d, J = 3.1 Hz, 9H), 1.08 (d, J = 1.2 Hz, 9H), 1.06 (d, J = 1.3 Hz, 9H).

^{13}C-NMR (101 MHz, $CDCl_3$) δ 155.96, 153.43, 135.59, 113.79 (d, J = 3.9 Hz), 111.92, 109.47, 79.65, 77.95, 58.43 (d, J = 16.0 Hz), 53.70 (d, J = 6.8 Hz), 53.46, 17.93, 17.80, 12.96, 12.57.
MS (ES+): m/z calc. for $C_{28}H_{54}BrO_6PSi_2$: 652.24; found: 675.7 [M+Na].

Procedure C *– Wittig-Horner reaction*

Compound V (1.0 equiv.) was dissolved in anhydrous THF under argon atmosphere and cooled to -78°C. LDA (2.0 M in THF, 1.2 equiv.) was added dropwise, and the solution was stirred for 20 minutes. Cycloalkyl ketone (~1.2 equiv.) was added, and after 10 minutes of stirring at -78°C, the reaction was warmed to room temperature and stirred for additional 20 minutes. The reaction was monitored by TLC (Hex: EtOAc mixture). Upon completion, the reaction mixture was diluted with EtOAc and washed with brine. The organic layer was dried over Na_2SO_4 and concentrated under reduced pressure. Purification by column chromatography (Hex:EtOAc mixture) afforded compounds **1b/2b.**

Procedure D *– Formylation and cyclization via Wittig reaction and TBS deprotection*

Compounds **1b/2b** (1.0 equiv.) were dissolved in anhydrous THF under an argon atmosphere and cooled to -78°C. n-Butyllithium (2.5M in hexanes, 1.0 equiv.) was added dropwise over few minutes and then dry DMF (1.5 equiv.) was added in one portion. The reaction mixture was allowed to warm to room temperature and stirred for 10 minutes. The solution was diluted with EtOAc (300 mL) and washed with brine (100 mL). The organic layer was dried over Na_2SO_4 and evaporated under reduced pressure. Along with the desired product, a considerable amount of the undesired Retro-Brook rearrangement product was obtained. The crude mixture was passed through a short pad of silica gel (Hex:EtOAc mixture) to remove highly polar impurities and used for the next step without further purification. The residue was dissolved in DCM and methyl (triphenylphosphoranylidene)acetate (2.0 equiv.) was added. The reaction mixture was heated to 35°C and monitored by TLC (Hex:EtOAc mixture). After completion (5 hours), the solution was diluted with DCM (200 mL) and washed with brine (100 mL). The organic layer was dried over Na_2SO_4 and evaporated under reduced pressure. The resulting crude was transferred to a sealed tube, to which toluene and TBAF (1M in THF,1.1 equiv.) were added. The reaction mixture was heated to 120°C and monitored by TLC (Hex:EtOAc mixture). Upon completion (3 hours), the mixture was diluted with EtOAc (200 mL) and washed with brine (100 mL). The organic layer was dried over Na_2SO_4 and concentrated under reduced pressure. The residue was purified by column chromatography on silica gel (Hex:EtOAc mixture) to afford compounds **1c/2c** in the form of a white solids.

Procedure E *– Oxidation reaction*

Compound **1c/2c** (~0.02-0.1 mmol) was dissolved in 5 mL of DCM followed by the addition of a catalytic amount of methylene blue. Then, oxygen was bubbled through the solution while irradiating

with yellow light. The reaction was monitored by TLC (Hex:EtOAc mixture) or RP-HPLC (gradient of 10-90% ACN in water, 0.1%TFA). Upon completion (about 5 min) the crude product was purified by RP-HPLC. The solvents were removed under reduced pressure with cooling bath at 10°C, to afford Coumarin phenoxy 1,2-dioxetanes (**Coumarin-CB/Coumarin-OX**).

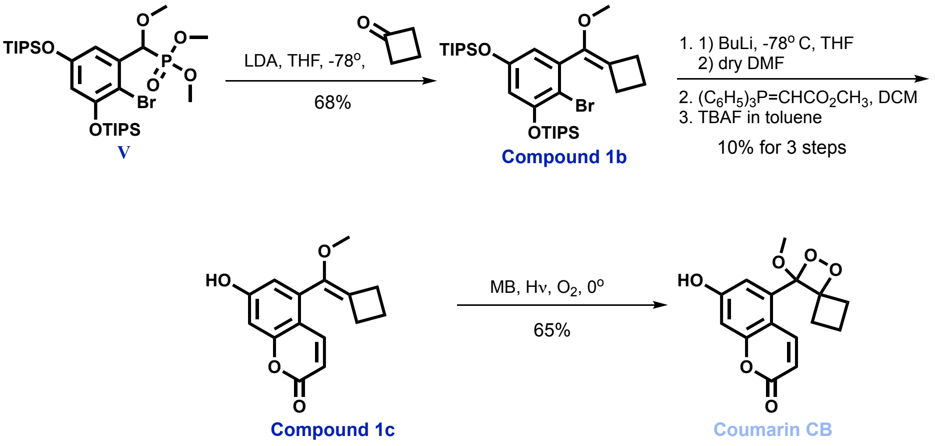

Compound 1b

Compound **1b** was synthesized according to **Procedure C**, using compound **V** as phosphonate (8000 mg, 12.2 mmol) in dry THF (25 mL), LDA (2.0M in THF, 7.3 mL, 14.68 mmol) and cyclobutanone (1.12 gr, 15.9 mmol). The reaction was monitored by TLC (90:10, Hex:EtOAc). Compound **1b** was obtained in the form of a colorless oil (4970 mg, 68%).

^{1}H-NMR (400 MHz, $CDCl_3$) δ δ 6.48 – 6.36 (m, 2H), 3.47 (s, 3H), 2.90 (ddd, J = 9.5, 5.4, 2.4 Hz, 2H), 2.46 (ddd, J = 9.5, 5.5, 2.5 Hz, 2H), 1.97 (p, J = 7.9 Hz, 2H), 1.36 – 1.18 (m, 6H), 1.11 (m, 36H).

^{13}C-NMR (101 MHz, $CDCl_3$) δ 155.35, 153.76, 145.60, 137.50, 119.13, 116.10, 111.44, 108.54, 56.79, 28.43, 27.99, 17.98, 17.83, 17.45, 12.99, 12.59.

MS (ES-): m/z calc. $C_{30}H_{53}BrO_3Si_2$: 596.27; found: 597.7 [M+H]+.

Compound 1c

Compound **1c** was synthesized according to **Procedure D**, using compound **1b** (4500 mg, 7.53 mmol) in dry THF (20 mL), n-Butyllithium (2.5M in hexanes, 3.0 mL, 7.5 mmol), dry DMF (0.90 ml, 11.6 mmol), methyl (triphenylphosphoranylidene)acetate (3700 mg, 11 mmol), and TBAF (1M in THF, 8.2 ml, 8.2 mmol). The reaction was monitored by TLC (90:10, Hex: EtOAc). Compound **1c** was obtained in a form of a white solid (195 mg, 10% for 3 steps).

^{1}H-NMR (400 MHz, DMSO-d6) δ 7.90 (d, J = 9.8 Hz, 1H), 6.72 – 6.49 (m, 2H), 6.23 (d, J = 9.7 Hz, 1H), 3.46 (s, 3H), 2.99 – 2.84 (m, 2H), 2.54 – 2.33 (m, 2H), 2.04 – 1.89 (m, 2H).

^{13}C-NMR (101 MHz, DMSO-d6) δ 161.19, 160.72, 156.63, 143.96, 142.97, 135.60, 121.87, 114.28, 112.18, 109.89, 102.73, 57.71, 29.29, 28.81, 17.60.

MS (ES-): m/z calc. $C_{15}H_{14}O_4$: 258.09; found: 259.2 $[M+H]^+$; 257.2 $[M-H]^-$.

Probe Coumarin CB

Coumarin cyclobutyl phenoxy 1,2-dioxetane (**Probe Coumarin-CB**) was synthesized according to **Procedure E** and obtained in the form of a white solid (36 mg, 65%). ^{1}H-NMR and ^{13}C-NMR were not obtained because of the non-stable nature of this dioxetane.

MS (ES-): m/z calc. $C_{15}H_{14}O_6$: 290.08; found: 289.2 $[M-H]^-$.

RP-HPLC analysis is presented below:

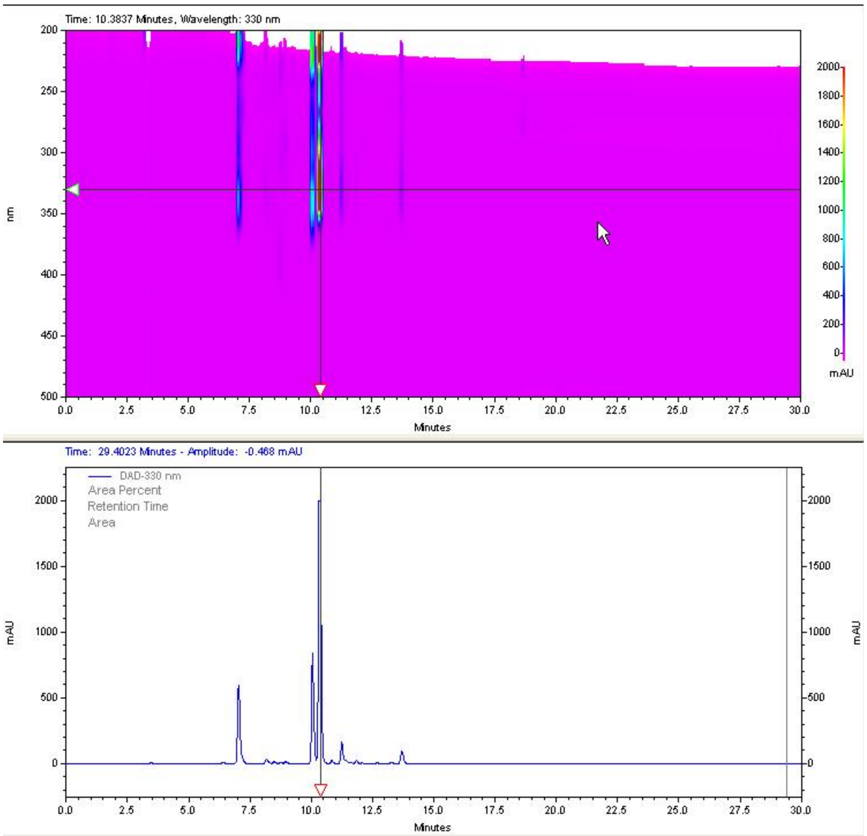

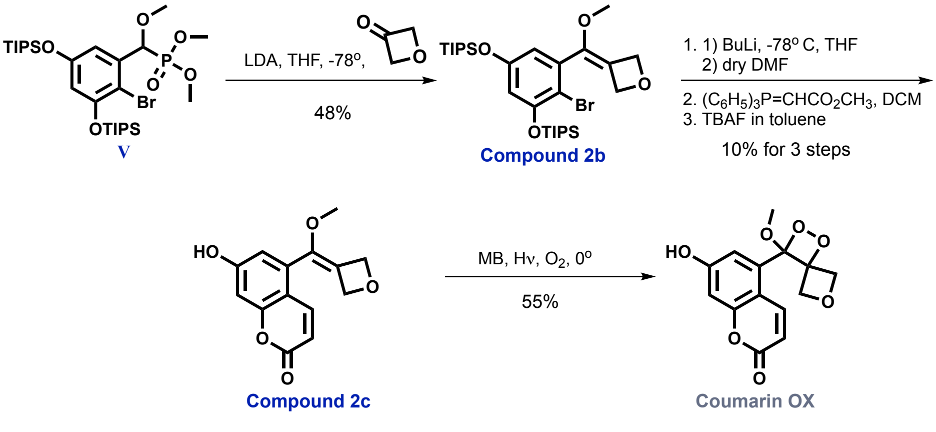

Compound 2b

Compound **2b** was synthesized according to **Procedure C**, using compound **V** as phosphonate (4000 mg, 6.1 mmol) in dry THF (10 mL), LDA (2.0M in THF, 3.7 mL, 7.4 mmol) and 3-oxetanone (0.66 gr, 9.15 mmol). The reaction was monitored by TLC (90:10, Hex:EtOAc). Compound **2b** was obtained in the form of a colorless oil (1760 mg, 48%).

^{1}H-NMR (400 MHz, ACN-d3) δ 6.49 (s, 2H), 5.42 (s, 2H), 4.86 (d, J = 2.0 Hz, 2H), 3.56 (s, 3H), 2.17 (s, 1H), 1.27 (m, 6H), 1.13 (s, 9H), 1.11 (s, 9H), 1.09 (s, 9H), 1.07 (s, 9H).

^{13}C-NMR (101 MHz, ACN-d3) δ 156.07, 154.21, 144.78, 137.40, 115.64, 112.10, 109.40, 107.58, 76.89, 76.70, 56.40, 17.61, 17.47, 13.04, 12.65.
MS (ES-): m/z calc. for $C_{29}H_{51}BrO_4Si_2$: 598.2; found: 599.6 $[M+H]^+$; 621.7 $[M+Na]^+$.

Compound 2c

Compound **2c** was synthesized according to **Procedure D**, using compound **2b** (1760 mg, 2.93 mmol) in dry THF (10 mL), n-Butyllithium (2.5M in hexanes, 1.3 mL, 3.2 mmol), dry DMF (0.50 ml, 6.5 mmol), methyl (triphenylphosphoranylidene)acetate (1700 mg, 5 mmol), and TBAF (1M in THF, 2.5 ml, 2.5 mmol). The reaction was monitored by TLC (90:10, Hex:EtOAc). Compound **2c** was obtained in the form of a white solid (195 mg, 10% for 3 steps).
^{1}H-NMR (400 MHz, DMSO-d6) δ 7.90 (d, J = 9.8 Hz, 1H), 6.72 – 6.49 (m, 2H), 6.23 (d, J = 9.7 Hz, 1H), 3.46 (s, 3H), 2.99 – 2.84 (m, 2H), 2.54 – 2.33 (m, 2H), 2.04 – 1.89 (m, 2H).
^{13}C-NMR (101 MHz, DMSO-d6) δ 161.27, 160.57, 156.67, 142.63, 142.59, 134.78, 113.83, 112.56, 111.41, 109.73, 103.35, 76.98, 57.13.
MS (ES-): m/z calc. for $C_{14}H_{12}O_5$: 260.1; found: 259.1 [M-H]-.

Probe Coumarin OX

Coumarin oxetanyl phenoxy 1,2-dioxetane (**Probe Coumarin-OX**) was synthesized according to **Procedure E** and obtained in the form of a white solid (22 mg, 55%).
MS (ES-): m/z calc. $C_{14}H_{12}O_7$: 292.1; found: 291.1 [M-H]-.
RP-HPLC analysis is presented below:

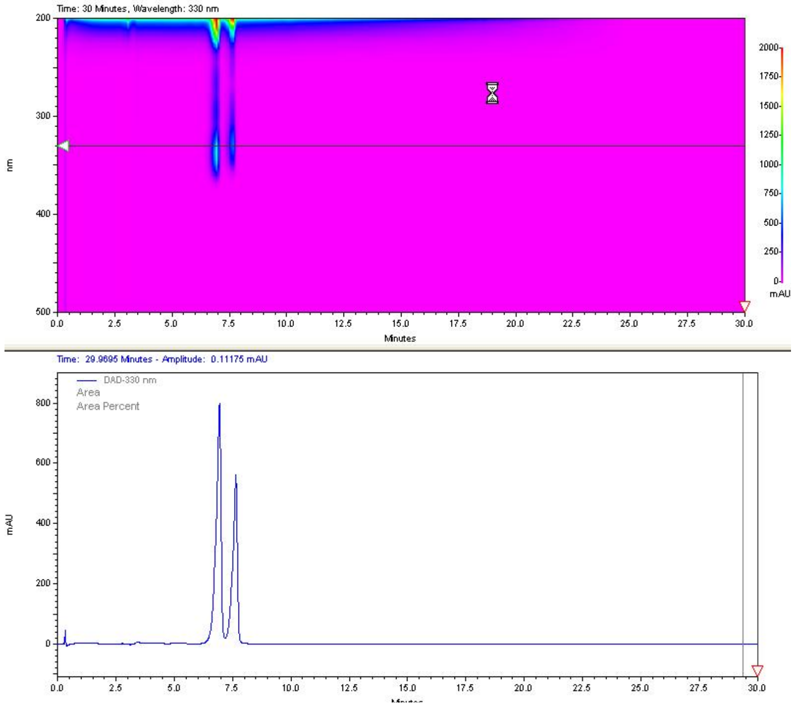

General synthetic procedures for the β-gal-masked 1,2-Dioxetanes

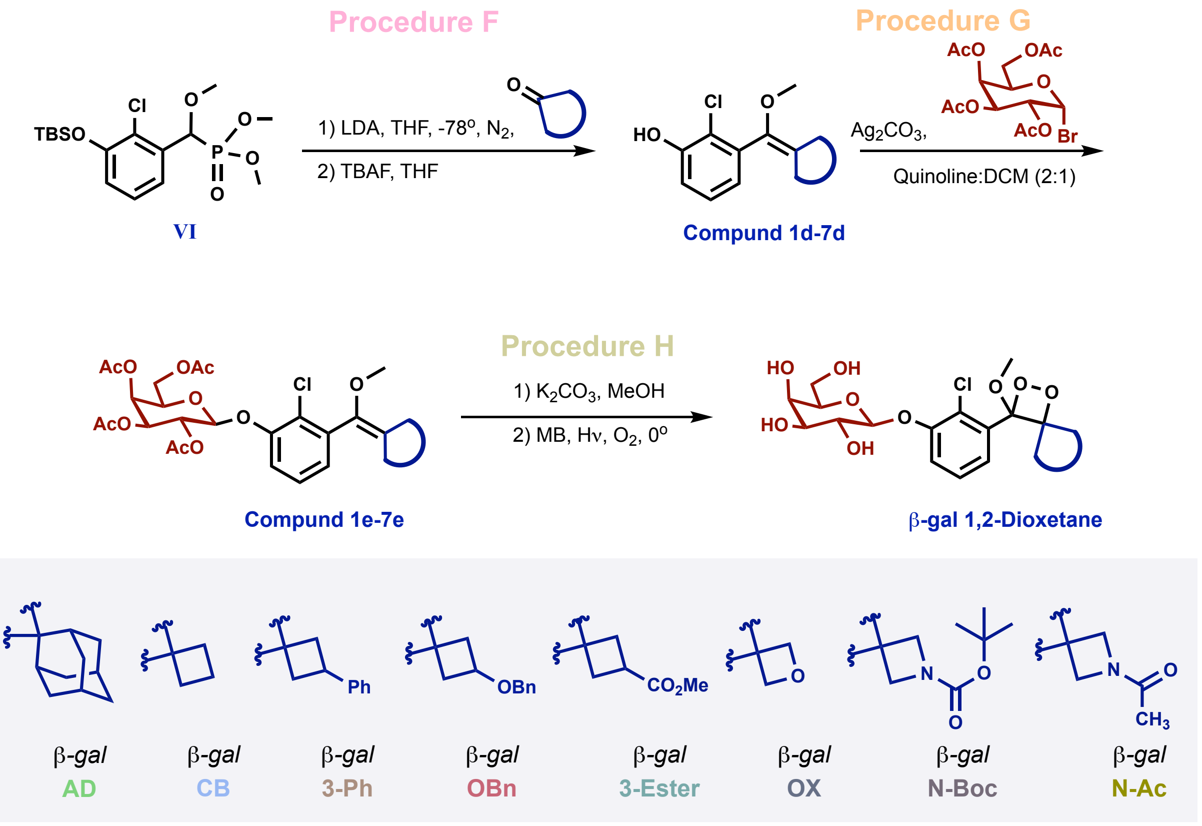

β-gal AD was synthesized according to a known procedure.[4] All compounds were synthesized according to general **procedures C, D** and **E**.

***Procedure F** - Wittig-Horner reaction and TBS deprotection.*

Phosphonate **VI**[4] (1.0 equiv.) was dissolved in anhydrous THF under argon atmosphere and cooled toat -78℃. LDA (2.0 M in THF, 1.2 equiv.) was added dropwise, and the solution was stirred for 20 minutes. Cycloalkyl ketone (~1.2 equiv.) was added, and after 10 minutes of stirring at -78℃, the reaction was warmed to room temperature and stirred for additional 20 minutes. The reaction was monitored by TLC (Hex:EtOAc mixture). Upon completion, TBAF (1.0 M in THF, 1.1 eq) was added, and the reaction was stirred for an additional 10 minutes at room temperature. The reaction was monitored by TLC (Hex: EtOAc mixture) and after full conversion, the reaction mixture was diluted with EtOAc and washed with 1M HCl followed by two additional washes with brine. The organic layer was dried over Na_2SO_4 and concentrated under reduced pressure. Purification by column chromatography (Hex: EtOAc mixture) afforded compounds **1d-7d**.

***Procedure G** – Glycosylation reaction (Koenigs–Knorr reaction).*

Compound **1d-7d** (1.0 eq) was dissolved in DCM: quinoline mixture (2:1). Acetobromo-α-D-galactose (1.2 eq) and silver carbonate (1.3 eq) were added, and the solution was stirred at room temperature. The reaction was monitored by TLC (Hex:EtOAc mixture). Upon completion, the reaction mixture was

filtered through a short celite pad and diluted with DCM (120 ml). The organic layer was washed twice with 1M HCl (2×100 ml) and brine (100 ml), dried over Na_2SO_4 and concentrated under reduced pressure. The resulted crude was purified by column chromatography (Hex:EtOAc mixture) to afford compound **1e-7e**.

Procedure H – *Acetate deprotection and oxidation reaction.*

Compound **1e-7e** (~30-60 mg, 1.0 eq) was dissolved in MeOH. Potassium carbonate (10 eq) was added, and the solution was stirred at room temperature. The reaction was monitored by RP-HPLC (30-100% ACN in water with 0.1% TFA). Upon completion, the reaction mixture was diluted with EtOAc and washed with 1M HCl and brine. The solvent was evaporated under reduced pressure and the obtained crude (in the form of a solid) was dissolved in DCM (10 mL) followed by the addition of a catalytic amount of methylene blue. The solution was cooled to 0°C, then oxygen was bubbled through the solution while irradiating with yellow light. The reaction was monitored by RP-HPLC (gradient of 30-100% ACN in ammonium carbonate buffer [30 mM]). Upon completion (about 5-15 min), DCM was evaporated under reduced pressure at 10°C. The crude product was purified by preparative RP-HPLC (30-100% ACN, ammonium carbonate buffer [30 mM] to afford **β-gal 1,2-dioxatene** derivatives.

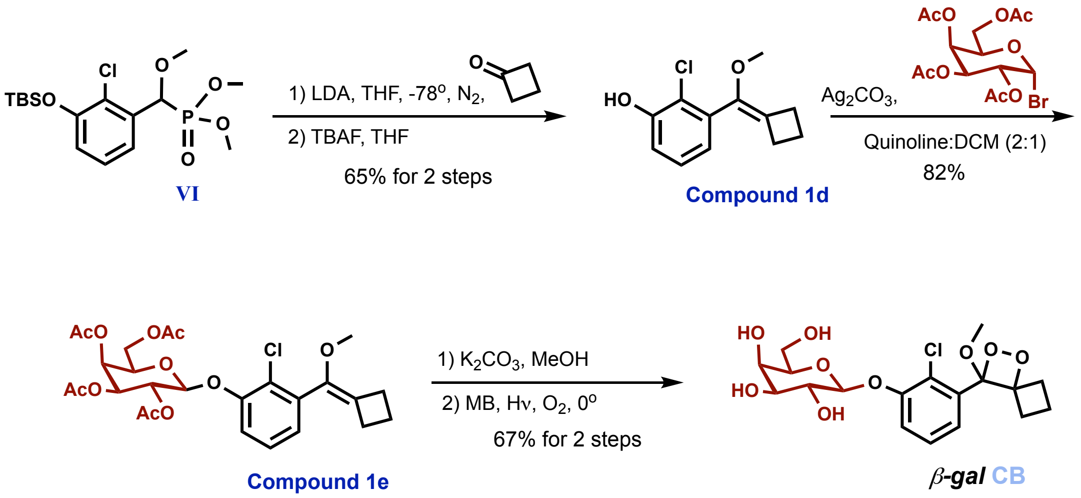

Compound 1d

Compound **1d** was synthesized according to **Procedure F**, using phosphonate **VI**[4] (300 mg, 0.76 mmol) in dry THF (1.5 mL), LDA (2.0M in THF, 0.46 mL, 0.91 mmol) and cyclobutanone (68 μL, 64 mg, 0.91 mmol). The reaction was monitored by TLC (90:10, Hex:EtOAc). Upon completion, TBAF (1.0M in THF, 0.84 mL, 0.84 mmol) was added to the solution. The crude residue was purified by column chromatography (70:30, Hex:EtOAc), to afford compound **1d** in the form of a white solid (111 mg, 0.49 mmol, 65%).

^{1}H-NMR (400 MHz, $CDCl_3$) δ 7.17 – 7.12 (t, J = 8.0 Hz, 1H), 6.98 (dd, J = 8.1, 1.6 Hz, 1H), 6.89 (dd, J = 7.6, 1.6 Hz, 1H), 5.93 (s, 1H), 3.53 (s, 3H), 3.01 – 2.91 (m, 2H), 2.59 – 2.51 (m, 2H), 2.05 – 1.96 (m, 2H).

^{13}C-NMR (101 MHz, $CDCl_3$) δ 151.82, 143.86, 134.61, 127.45, 123.04, 121.30, 119.53, 115.56, 57.33, 28.72, 28.35, 17.47.

MS (ES-): m/z calc. $C_{12}H_{13}ClO_2$: 224.1; found: 223.3 $[M-H]^{-\cdot}$

Compound 1e

To a solution of compound **1d** (111 mg, 0.49 mmol) in DCM (0.3 mL) and quinoline (0.6 mL), Ag_2CO_3 (176 mg, 0.64 mmol) and Acetobromo-α-D-galactose (242 mg, 0.59 mmol) were added following **Procedure G**. The crude residue was purified by column chromatography (60:40, Hex:EtOAc), to afford compound **1e** in the form of a white solid (223 mg, 0.40 mmol, 82%).

^{1}H-NMR (400 MHz, $CDCl_3$) δ 7.16 – 7.14 (m, 2H), 7.05 (dd, *J* = 5.8, 3.4 Hz, 1H), 5.58 (dd, *J* = 10.5, 8.0 Hz, 1H), 5.46 (dd, *J* = 3.4, 0.9 Hz, 1H), 5.10 (dd, *J* = 10.5, 3.4 Hz, 1H), 4.95 (d, *J* = 8.0 Hz, 1H), 4.25 (dd, *J* = 11.3, 7.0 Hz, 1H), 4.16 (dd, *J* = 11.2, 6.3 Hz, 1H), 4.07 – 4.01 (m, 1H), 3.52 (s, 3H), 2.95 (t, *J* = 7.9 Hz, 2H), 2.54 – 2.42 (m, 2H), 2.19 (s, 3H), 2.09 (s, 3H), 2.06 (s, 3H), 2.01 (s, 3H), 2.01 – 1.95 (m, 2H).

^{13}C-NMR (101 MHz, $CDCl_3$) δ 170.35, 170.26, 170.17, 169.43, 152.98, 143.77, 136.12, 127.54, 126.83, 126.28, 123.26, 118.10, 100.82, 71.14, 70.68, 68.22, 66.85, 61.34, 57.39, 28.67, 28.41, 20.89, 20.85, 20.66, 20.60, 17.47.

MS (ES+): m/z calc. $C_{26}H_{31}ClO_{11}$: 554.16; found: 577.5 $[M+Na]^+$.

Probe β-gal CB

Compound **1e** (50 mg, 0.09 mmol) was reacted according to **Procedure H**. Reaction was monitored by RP-HPLC (30-100% ACN, ammonium carbonate buffer [30 mM]). Upon completion, the crude product was purified by preparative RP-HPLC (30-100% ACN, ammonium carbonate buffer [30 mM]) to afford **Probe β-gal CB** as a diastereomers mixture (1:1 ratio) in the form of a white solid (25 mg, 67% yield).

^{1}H-NMR (400 MHz, CDCl$_3$) (Diastereomeric mixture) δ 7.41 (t, J = 7.1 Hz, 1H), 7.29 (t, J = 7.4 Hz, 1H), 7.20 (d, J = 8.3 Hz, 1H), 4.87 (t, J = 7.8 Hz, 1H), 4.18 (s, 1H), 4.14 – 4.05 (m, 1H), 3.91 – 3.74 (m, 3H), 3.66 (t, J = 5.2 Hz, 1H), [3.30, 3.20 (s, 3H)], 3.13 (td, J = 15.5, 7.9 Hz, 1H), 2.43 – 2.28 (m, 1H), 2.28 – 2.05 (m, 2H), 1.77 – 1.64 (m, 2H).

^{13}C-NMR (101 MHz, CDCl$_3$) (Diastereomeric mixture) δ [153.37, 153.26], [135.22, 135.15], 128.06, [123.80, 123.61], [121.21, 121.18], [118.15, 118.10], 111.00, [102.23, 102.12], [95.83, 95.76], [74.79, 74.73], 73.32, 71.25, [68.99, 68.95], 61.66, [51.25, 51.06], 33.12, [31.06, 30.93], 12.53.

MS (ES-): m/z calc. $C_{18}H_{23}ClO_9$: 418.10; found: 417.4 $[M-H]^-$.

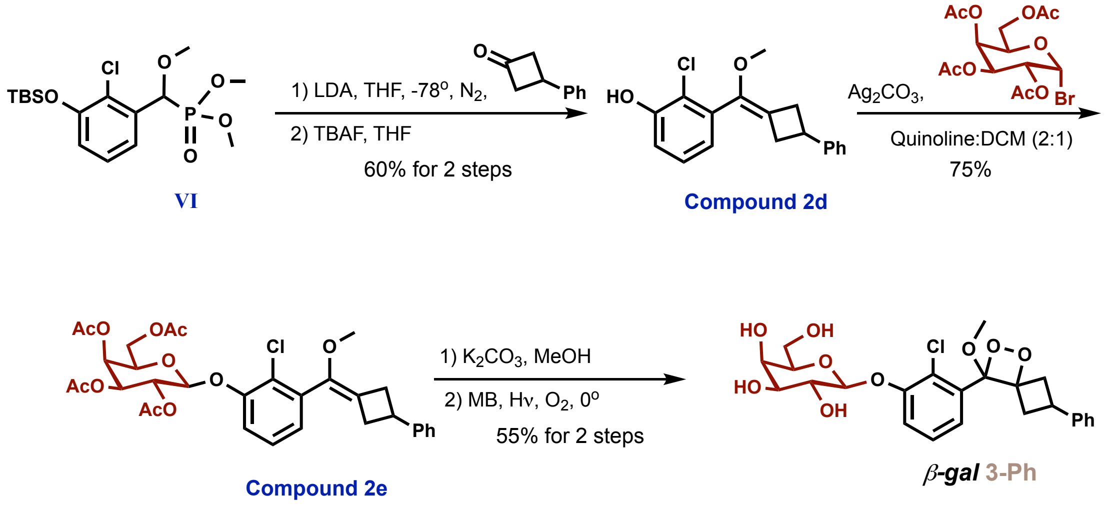

Compound 2d

Compound **2d** was synthesized according to **Procedure F**, using phosphonate **VI**[4] (300 mg, 0.76 mmol) in dry THF (1.5 mL), LDA (2.0M in THF, 0.46 mL, 0.91 mmol) and 3-phenylcyclobutanone (119 µL, 133 mg, 0.91 mmol). The reaction was monitored by TLC (90:10, Hex:EtOAc). Upon completion, TBAF (1.0M in THF, 0.84 mL, 0.84 mmol) was added to the solution. The crude residue was purified by column chromatography (70:30, Hex:EtOAc), to afford compound **2d** in the form of a white solid (137 mg, 0.46 mmol, 60%).

^{1}H-NMR (400 MHz, CDCl$_3$) δ 7.38 – 7.29 (m, 4H), 7.26 – 7.17 (m, 2H), 7.02 (dd, J = 8.2, 1.6 Hz, 1H), 6.95 (dd, J = 7.6, 1.6 Hz, 1H), 5.81 (s, 1H), 3.67 – 3.60 (m, 1H), 3.59 (s, 3H), 3.50 – 3.42 (m, 1H), 3.13 – 3.05 (m, 1H), 2.98 – 2.90 (m, 1H), 2.81 – 2.73 (m, 1H).

^{13}C-NMR (101 MHz, CDCl$_3$) δ 151.82, 145.75, 144.68, 134.55, 128.40, 127.61, 126.45, 126.08, 123.04, 119.50, 116.69, 115.71, 57.41, 36.69, 36.22, 35.57.
MS (ES-): *m/z* calc. $C_{18}H_{17}ClO_2$: 300.1; found: 299.3 $[M-H]^-$.

Compound 2e

To a solution of compound **2d** (137 mg, 0.46 mmol) in DCM (0.3 mL) and quinoline (0.6 mL), Ag_2CO_3 (164 mg, 0.60 mmol) and Acetobromo-α-D-galactose (227 mg, 0.55 mmol) were added following **Procedure G**. The crude residue was purified by column chromatography (50:50, Hex:EtOAc), to afford compound **2e** in the form of a white solid (217 mg, 0.34 mmol, 75%).
Compound **2e** was obtained as a diastereomeric mixture in a 1:1 ratio.
^{1}H-NMR (400 MHz, CDCl$_3$) (Diastereomeric mixture) δ 7.34 – 7.25 (m, 4H), 7.22 – 7.15 (m, 3H), 7.11 – 7.07 (m, 1H), 5.59 (dd, *J* = 9.7, 8.1 Hz, 1H), 5.46 (d, *J* = 3.2 Hz, 1H), 5.14 – 5.08 (m, 1H), 4.97 (dd, *J* = 8.0, 2.1 Hz, 1H), 4.26 (dd, *J* = 11.3, 7.0 Hz, 1H), 4.16 (dd, *J* = 11.3, 6.4 Hz, 1H), 4.04 (t, *J* = 6.5 Hz, 1H), 3.63 – 3.57 (m, 1H), [3.57, 3.56 (s, 3H)], 3.50 – 3.50 (m, 1H), 3.48 – 3.38 (m, 1H), 3.10 – 3.01 (m, 1H), 2.92 – 2.80 (m, 1H), 2.69 (dddd, *J* = 22.7, 15.1, 7.3, 2.6 Hz, 1H), 2.19 (s, 3H), [2.10, 2.09 (s, 3H)], 2.06 (s, 3H), 2.02 (s, 3H).
^{13}C-NMR (101 MHz, CDCl$_3$) (Diastereomeric mixture) δ 170.48, 170.38, 170.29, 169.56, 153.14, [145.89, 145.83], 144.68, 136.14, 128.50, 127.06, 126.55, 126.39, 126.30, 126.18, [118.42, 118.25], [116.58, 116.30], [100.94, 100.89], 71.29, 70.81, 68.35, 66.98, 61.47, [57.57, 57.54], [36.77, 36.70], [36.49, 36.38], 35.67, 21.02, 20.79, 20.73.
MS (ES+): *m/z* calc. $C_{32}H_{35}ClO_{11}$: 630.19; found: 653.5 $[M+Na]^+$.

Probe β-gal 3-Ph

Compound **2e** (60 mg, 0.09 mmol) was reacted according to **Procedure H**. Upon completion, the crude product was purified by preparative RP-HPLC (30-100% ACN, ammonium carbonate buffer [30 mM]) to afford **Probe β-gal 3-Ph** as a diastereomeric mixture (1.4:1 ratio) in the form of a white solid (26 mg, 55% yield).
^{1}H-NMR (400 MHz, MeOD) (Diastereomeric mixture) δ 7.48 – 7.36 (m, 3H), 7.30 – 7.22 (m, 2H), 7.22 – 7.10 (m, 3H), 5.07 – 4.98 (m, 1H), 3.98 – 3.85 (m, 2H), 3.83 – 3.67 (m, 3H), 3.66 – 3.55 (m, 1H), 3.54 – 3.32 (m, 1H), [3.25, 3.23, 3.22, 3.20 (s, 3H)], 2.96 – 2.13 (m, 3H).
^{13}C-NMR (101 MHz, MeOD) (Diastereomeric mixture) δ [154.13, 154.05, 153.96], [144.76, 144.74], [143.68, 143.62], [134.34, 134.21], [128.23, 128.15], [127.67, 127.58], 126.04, [122.61, 122.56], [121.91, 121.80], 120.33, [117.49, 117.48, 117.34, 117.25], [110.94, 110.56], [101.46, 101.33, 101.19], [94.21, 94.17, 91.63], 75.92, 73.68, [70.88, 70.79], 68.92, 61.10, [49.91, 49.86], [40.63, 40.57, 39.65, 39.61], [38.93, 38.82, 38.20], [31.77, 31.67, 30.09, 30.04].
MS (ES-): *m/z* calc. $C_{24}H_{27}ClO_9$: 494.13; found: 493.5 $[M-H]^-$.

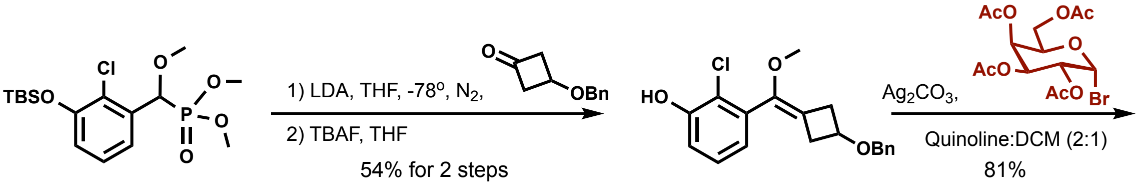

VI

Compound 3d

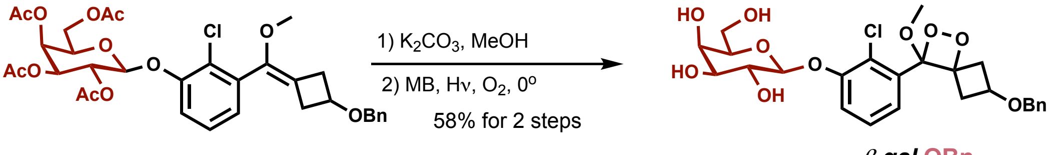

Compound 3e

β-gal OBn

Compound 3d

Compound **3d** was synthesized according to **Procedure F**, using phosphonate **VI**[4] (300 mg, 0.76 mmol) in dry THF (1.5 mL), LDA (2.0M in THF, 0.46 mL, 0.91 mmol) and 3-(benzyloxy)cyclobutanone (144 µL, 160 mg, 0.91 mmol). The reaction was monitored by TLC (90:10, Hex:EtOAc). Upon completion, TBAF (1.0M in THF, 0.84 mL, 0.84 mmol) was added to the solution. The crude residue was purified by column chromatography (70:30, Hex:EtOAc), to afford compound **3d** in the form of a white solid (145 mg, 0.44 mmol, 58%).

^{1}H-NMR (400 MHz, $CDCl_3$) δ 7.39 – 7.27 (m, 5H), 7.15 (t, *J* = 8.0 Hz, 1H), 6.98 (dd, *J* = 8.2, 1.6 Hz, 1H), 6.86 (dd, *J* = 7.6, 1.6 Hz, 1H), 4.44 (s, 2H), 4.20 – 4.11 (m, 1H), 3.51 (s, 3H), 3.26 – 3.18 (m, 1H), 2.95 – 2.86 (m, 1H), 2.69 – 2.64 (m, 1H).

^{13}C NMR (101 MHz, $CDCl_3$) δ 151.91, 146.01, 138.10, 134.68, 128.57, 128.05, 127.86, 127.70, 123.06, 115.86, 112.36, 70.48, 69.63, 57.50, 37.66, 37.02.

MS (ES-): *m/z* calc. for $C_{19}H_{19}ClO_3$: 330.10; found: 329.4 $[M-H]^-$.

Compound 3e

To a solution of compound **3d** (145 mg, 0.44 mmol) in DCM (0.3 mL) and quinoline (0.6 mL), Ag_2CO_3 (157 mg, 0.57 mmol) and Acetobromo-α-D-galactose (217 mg, 0.53 mmol) were added following **Procedure G**. The crude residue was purified by column chromatography (50:50, Hex:EtOAc), to afford compound **3e** as a diastereomeric mixture (1:1 ratio) in the form of a white solid (235 mg, 0.36 mmol, 81%).

^{1}H-NMR (400 MHz, $CDCl_3$) δ (Diastereomeric mixture) 7.36 – 7.26 (m, 5H), 7.16 (d, *J* = 5.0 Hz, 2H), 7.06 – 7.01 (m, 1H), 5.58 (ddd, *J* = 10.5, 8.0, 1.1 Hz, 1H), 5.46 (dd, *J* = 3.3, 0.7 Hz, 1H), 5.10 (dd, *J* = 10.5, 3.4 Hz, 1H), 4.95 (d, *J* = 8.0 Hz, 1H), 4.42 (s, 2H), 4.26 (dd, *J* = 11.3, 7.0 Hz, 1H), 4.20 – 4.10 (m, 2H), 4.04 (dd, *J* = 9.7, 3.5 Hz, 1H), [3.52, 3.51 (s, 3H)], 3.26 – 3.17 (m, 1H), 2.91 (ddd, *J* = 15.3, 3.9, 2.0 Hz, 1H), 2.70 – 2.55 (m, 2H), 2.19 (s, 3H), 2.09 (s, 3H), 2.06 (s, 3H), 2.01 (s, 3H).

^{13}C-NMR (101 MHz, $CDCl_3$) (Diastereomeric mixture) δ 170.34, 170.24, 170.16, 169.41, [153.00, 152.96], 145.73, 137.98, [136.04, 136.00], 128.42, 127.90, 127.71, [126.93, 126.91], [126.17, 126.09],

[124.17, 124.13], [118.30, 118.23], [112.21, 111.69], [100.81, 100.75], 71.16, 70.67, 70.32, [69.53, 69.45], 68.20, 66.84, 61.34, [57.43, 57.37], [37.52, 37.43], 36.95, 20.89, 20.67, 20.60.
MS (ES+): *m/z* calc. for $C_{33}H_{37}ClO_{12}$: 660.20; found: 683.6 $[M+Na]^+$.

Probe β-gal OBn

Compound **3e** (70 mg, 0.10 mmol) was reacted according to **Procedure H**. Upon completion, the crude product was purified by preparative RP-HPLC (30-100% ACN, ammonium carbonate buffer [30 mM]) to afford **Probe β-gal OBn** as a diastereomeric mixture (1.6:1 ratio) in the form of a white solid (32 mg, 58% yield).
^{1}H-NMR (400 MHz, $CDCl_3$) (Diastereomeric mixture) δ 7.49 – 7.37 (m, 1H), 7.35 – 7.21 (m, 7H), 4.93 – 4.80 (m, 1H), 4.39 – 4.31 (m, 2H), 4.11 – 3.98 (m, 2H), 3.92 – 3.80 (m, 2H), 3.73 – 3.52 (m, 2H), [3.28, 3.22, 3.20, 3.15 (s, 3H)], 3.12 – 3.02 (m, 1H), 2.86 – 2.74 (m, 1H), 2.55 – 2.42 (m, 1H), 2.37 – 2.22 (m, 2H).
^{13}C-NMR (101 MHz, $CDCl_3$) (Diastereomeric mixture) δ [153.66, 153.57, 153.51, 153.41], [137.79, 137.57], [134.55, 134.44], 128.57, [128.07, 127.96], [123.82, 123.66, 123.16, 123.10], [121.35, 120.93, 120.84], [118.49, 118.07, 117.89], 110.94, [102.27, 101.91, 101.86], [92.01, 91.95, 89.64, 89.59], 75.06, 73.24, 71.23, [70.79, 70.70, 70.63], [69.04, 68.92], [66.42, 66.34], 61.90, [51.24, 51.08, 50.94], 41.07, [40.50, 40.42].
MS (ES+): *m/z* calc. for $C_{25}H_{29}ClO_{10}$: 524.14; found: 547.5 $[M+Na]^+$.

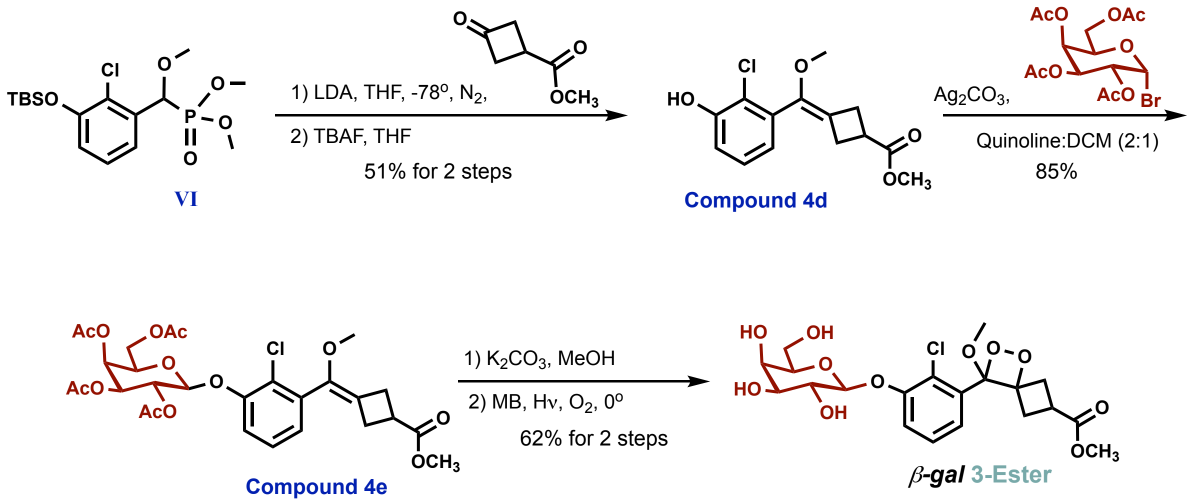

Compound 4d

Compound **4d** was synthesized according to **Procedure F**, using phosphonate **VI**[4] (300 mg, 0.76 mmol) in dry THF (1.5 mL), LDA (2.0M in THF, 0.46 mL, 0.91 mmol) and methyl 3-oxo-cyclobutane carboxylate (96 μL, 116 mg, 1.00 mmol). The reaction was monitored by TLC (90:10, Hex:EtOAc). Upon completion, TBAF (1.0M in THF, 0.84 mL, 0.84 mmol) was added to the solution. The crude residue

was purified by column chromatography (70:30, Hex:EtOAc), to afford compound **4d** in the form of a white solid (109 mg, 0.39 mmol, 51%).

^{1}H-NMR (400 MHz, $CDCl_3$) δ 7.14 (t, *J* = 7.8 Hz, 1H), 7.01 – 6.96 (m, 1H), 6.88 – 6.85 (m, 1H), 6.03 (s, 1H), 3.71 (s, 3H), 3.52 (s, 3H), 3.26 – 3.13 (m, 3H), 2.93 – 2.83 (m, 1H), 2.74 – 2.65 (m, 1H).

^{13}C-NMR (101 MHz, $CDCl_3$) δ 175.67, 151.94, 145.35, 134.00, 127.58, 122.89, 119.45, 115.96, 114.71, 57.24, 51.90, 33.67, 32.24, 31.98.

MS (ES-): *m/z* calc. for $C_{14}H_{15}ClO_4$: 282.1; found: 281.3 $[M-H]^-$.

Compound 4e

To a solution of compound **4d** (109 mg, 0.39 mmol) in DCM (0.3 mL) and quinoline (0.6 mL), Ag_2CO_3 (139 mg, 0.50 mmol) and Acetobromo-α-D-galactose (192 mg, 0.47 mmol) were added following **Procedure G**. The crude residue was purified by column chromatography (60:40, Hex:EtOAc), to afford compound **4e** as a diastereomeric mixture (1:1 ratio) in the form of a white solid (198 mg, 0.33 mmol, 85%).

^{1}H-NMR (400 MHz, $CDCl_3$) (Diastereomeric mixture) δ 7.19 – 7.13 (m, 2H), 7.05 – 7.02 (m, 1H), 5.57 (dd, *J* = 10.3, 8.1 Hz, 1H), 5.45 (dd, *J* = 3.3, 0.8 Hz, 1H), 5.09 (dd, *J* = 10.5, 3.4 Hz, 1H), 4.95 (d, *J* = 8.0 Hz, 1H), 4.24 (dd, *J* = 11.3, 7.0 Hz, 1H), 4.15 (dd, *J* = 11.2, 6.3 Hz, 1H), 4.04 (t, *J* = 6.6 Hz, 1H), [3.69, 3.68 (s, 3H)], [3.53, 3.51 (s, 3H)], 3.23 – 3.11 (m, 3H), 2.89 – 2.76 (m, 1H), 2.70 – 2.58 (m, 1H), 2.18 (s, 3H), 2.09 (s, 3H), 2.05 (s, 3H), 2.01 (s, 3H).

^{13}C-NMR (101 MHz, $CDCl_3$) (Diastereomeric mixture) δ [175.51, 175.45], 170.34, 170.24, 170.15, 169.40, 153.00, 145.20, [135.48, 135.41], 126.99, [126.19, 126.11], [124.19, 124.13], [118.44, 118.31], [114.73, 114.20], 100.74, 71.16, 70.66, 68.19, 66.83, 61.33, 57.31, 51.84, 33.65, [32.25, 32.13], [32.07, 32.01], 20.87, 20.65, 20.59.

MS (ES+): *m/z* calc. for $C_{14}H_{15}ClO_4$: 612.16; found: 635.5 $[M+Na]^+$.

Probe β-gal 3-Ester

Compound **4e** (60 mg, 0.10 mmol) was reacted according to **Procedure H**. Upon completion, the crude product was purified by preparative RP-HPLC (30-100% ACN, ammonium carbonate buffer [30 mM]) to afford **Probe β-gal 3-Ester** as a diastereomeric mixture (1.4:1 ratio) in the form of a white solid (29 mg, 62% yield).

^{1}H-NMR (400 MHz, MeOD) (Diastereomeric mixture) δ 7.43 – 7.37 (m, 2H), 7.36 – 7.28 (m, 1H), 5.04 – 4.99 (m, 1H), 3.94 – 3.87 (m, 2H), 3.81 – 3.70 (m, 3H), [3.65, 3.64 (s, 3H)], 3.62 – 3.58 (m, 1H), 3.53 – 3.39 (m, 1H), [3.20, 3.19, 3.17, 3.16 (s, 3H)], 3.15 – 2.89 (m, 1H), 2.69 – 2.53 (m, 1H), 2.53 – 2.35 (m, 2H).

^{13}C-NMR (101 MHz, MeOD) (Diastereomeric mixture) δ [175.17, 174.86, 174.76], [154.12, 154.07, 154.04], [133.90, 133.80], [127.75, 127.58], [122.43, 122.33, 121.91, 121.80], 120.52, [117.63, 117.59, 117.56, 117.46], [110.52, 110.49, 110.45, 110.39], [101.48, 101.35], [93.47, 91.27, 91.24], 75.95, 73.70, 70.81, 68.93, 61.12, 51.23, 49.80, [36.07, 35.64], [34.24, 34.19, 33.81], [29.58, 28.80].

MS (ES-/+): *m/z* calc. for $C_{20}H_{25}ClO_{11}$: 476.11; found: 475.4 $[M-H]^-$, 494.5 $[M+NH_4]^+$.

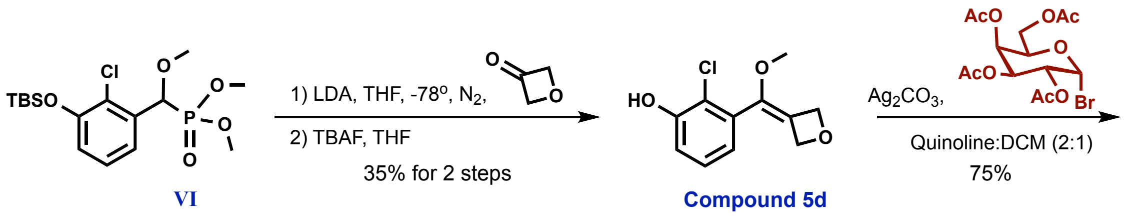

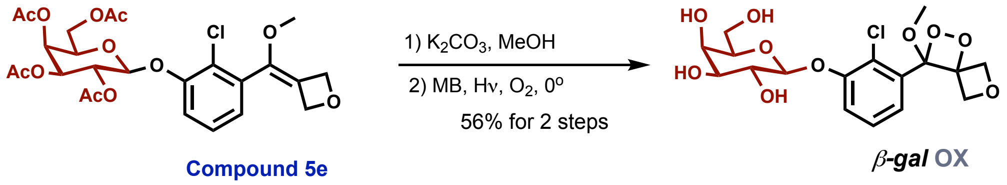

Compound 5d

Compound **5d** was synthesized according to **Procedure F**, using phosphonate **VI**[4] (300 mg, 0.76 mmol) in dry THF (1.5 mL), LDA (2.0M in THF, 0.46 mL, 0.91 mmol) and 3-oxetanone (58 µL, 65 mg, 0.91 mmol). The reaction was monitored by TLC (90:10, Hex:EtOAc). Upon completion, TBAF (1.0M in THF, 0.84 mL, 0.84 mmol) was added to the solution. The crude residue was purified by column chromatography (60:40, Hex:EtOAc), to afford compound **5d** in the form of a white solid (60 mg, 0.27 mmol, 35%).

^{1}H-NMR (400 MHz, $CDCl_3$) δ 7.17 (t, *J* = 7.9 Hz, 1H), 7.02 (dd, *J* = 8.2, 1.4 Hz, 1H), 6.88 (dd, *J* = 7.5, 1.4 Hz, 1H), 5.55 (t, *J* = 2.5 Hz, 2H), 5.06 (t, *J* = 2.5 Hz, 2H), 3.62 (s, 3H).

^{13}C-NMR (101 MHz, $CDCl_3$) δ 152.04, 143.06, 133.43, 127.92, 122.29, 119.13, 116.60, 111.33, 56.82, 29.70.

MS (ES-): *m/z* calc. for $C_{11}H_{11}ClO_3$: 226.0; found: 225.3 $[M-H]^-$.

Compound 5e

To a solution of compound **5e** (60 mg, 0.27 mmol) in DCM (0.2 mL) and quinoline (0.4 mL), Ag_2CO_3 (96 mg, 0.35 mmol) and Acetobromo-α-D-galactose (133 mg, 0.32 mmol) were added following **Procedure G**. The crude residue was purified by column chromatography (60:40, Hex:EtOAc), to afford compound **5e** in the form of a white solid (150 mg, 0.20 mmol, 75%).

^{1}H-NMR (400 MHz, $CDCl_3$) δ 7.21 – 7.17 (m, 2H), 7.04 (t, *J* = 7.8 Hz, 1H), 5.58 (dd, *J* = 10.5, 8.0 Hz, 1H), 5.54 – 5.50 (m, 2H), 5.46 (dd, *J* = 3.4, 0.9 Hz, 1H), 5.10 (dd, *J* = 10.5, 3.4 Hz, 1H), 5.02 – 4.94 (m, 3H), 4.25 (dd, *J* = 11.3, 7.0 Hz, 1H), 4.16 (dd, *J* = 11.3, 6.2 Hz, 1H), 4.05 (t, *J* = 6.6 Hz, 1H), 3.60 (s, 3H), 2.19 (s, 3H), 2.10 (s, 3H), 2.05 (s, 3H), 2.01 (s, 3H).

^{13}C-NMR (101 MHz, $CDCl_3$) δ 170.32, 170.22, 170.13, 169.36, 153.08, 142.85, 134.80, 127.30, 125.45, 123.92, 118.78, 111.21, 100.64, 77.50, 77.20, 71.19, 70.61, 68.15, 66.81, 61.32, 56.83, 20.84, 20.64, 20.57.

MS (ES+): *m/z* calc. for $C_{25}H_{29}ClO_{12}$: 556.13; found: 579.5 $[M+Na]^+$.

Probe β-gal OX

Compound **5e** (70 mg, 0.12 mmol) was reacted according to **Procedure H**. Upon completion, the crude product was purified by preparative RP-HPLC (30-100% ACN, ammonium carbonate buffer [30 mM]) to afford **Probe β-gal OX** as a diastereomeric mixture (1:1 ratio) in the form of a white solid (30 mg, 62% yield).

^{1}H-NMR (400 MHz, MeOD) (Diastereomeric mixture) δ 7.49 – 7.38 (m, 2H), 7.31 (dd, *J* = 7.4, 1.8 Hz, 1H), 5.50 (dd, *J* = 9.5, 1.1 Hz, 1H), 5.04 (d, *J* = 7.7 Hz, 1H), 4.83 (d, *J* = 9.6 Hz, 1H), 4.66 – 4.58 (m, 2H), 3.93 (td, *J* = 7.5, 3.6 Hz, 2H), 3.82 – 3.71 (m, 3H), 3.62 (dd, *J* = 9.7, 3.4 Hz, 1H), 3.23 (s, 3H).

^{13}C-NMR (101 MHz, MeOD) (Diastereomeric mixture) δ 154.09, 133.10, 127.59, 121.48, 121.44, 117.77, 117.71, 101.39, 101.29, 93.55, 79.45, 77.98, 75.84, 73.56, 70.68, 68.79, 60.99, 49.78.

MS (ES-/+): *m/z* calc. for $C_{17}H_{21}ClO_{10}$: 420.08; found: 419.4 $[M-H]^-$, 443.4 $[M+Na]^+$.

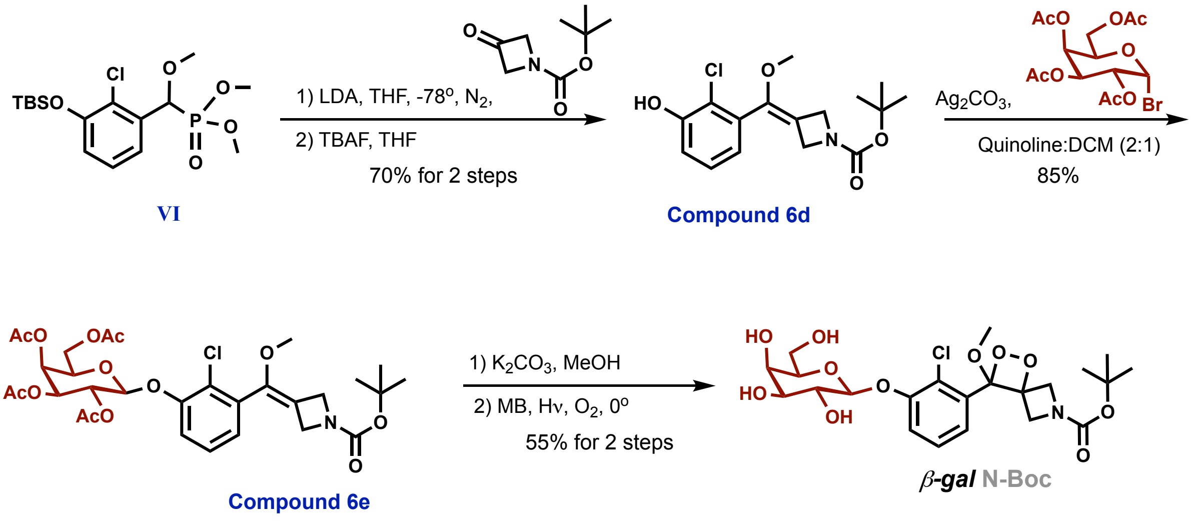

Compound 6d

Compound **6d** was synthesized according to **Procedure F**, using phosphonate **VI**[4] (300 mg, 0.76 mmol) in dry THF (1.5 mL), LDA (2.0M in THF, 0.46 mL, 0.91 mmol) and 1-Boc-3-azetidinone (156 mg, 0.91 mmol). The reaction was monitored by TLC (90:10, Hex:EtOAc). Upon completion, TBAF (1.0M in THF, 0.84 mL, 0.84 mmol) was added to the solution. The crude residue was purified by column chromatography (60:40, Hex:EtOAc), to afford compound **6d** in the form of a white solid (173 mg, 0.53 mmol, 70%).

^{1}H-NMR (400 MHz, $CDCl_3$) δ 7.13 (t, *J* = 7.9 Hz, 1H), 7.00 (dd, *J* = 8.2, 1.5 Hz, 1H), 6.83 (dd, *J* = 7.5, 1.4 Hz, 1H), 4.77 (t, *J* = 2.6 Hz, 2H), 4.28 (t, *J* = 2.7 Hz, 2H), 3.58 (s, 3H), 1.43 (s, 9H).

^{13}C-NMR (101 MHz, $CDCl_3$) δ 156.47, 152.49, 145.83, 133.64, 127.90, 122.38, 119.50, 116.87, 106.00, 80.07, 56.96, 28.51.

MS (ES-): *m/z* calc. for $C_{16}H_{20}ClNO_4$: 325.1; found: 324.4 $[M-H]^-$.

Compound 6e

To a solution of compound **6e** (173 mg, 0.53 mmol) in DCM (0.2 mL) and quinoline (0.4 mL), Ag_2CO_3 (189 mg, 0.69 mmol) and Acetobromo-α-D-galactose (261 mg, 0.64 mmol) were added following **Procedure G**. The crude residue was purified by column chromatography (50:50, Hex:EtOAc), to afford compound **6e** in the form of a white solid (295 mg, 0.45 mmol, 85%).

^{1}H-NMR (400 MHz, $CDCl_3$) δ 7.20 (d, *J* = 4.7 Hz, 2H), 7.04 (t, *J* = 7.8 Hz, 1H), 5.58 (dd, *J* = 10.4, 8.0 Hz, 1H), 5.47 (d, *J* = 3.3 Hz, 1H), 5.10 (dd, *J* = 10.5, 3.4 Hz, 1H), 4.96 (d, *J* = 8.0 Hz, 1H), 4.78 (s, 2H), 4.31 – 4.21 (m, 3H), 4.16 (dd, *J* = 11.2, 6.2 Hz, 1H), 4.04 (t, *J* = 6.5 Hz, 1H), 3.61 (s, 3H), 2.19 (s, 3H), 2.10 (s, 3H), 2.06 (s, 3H), 2.02 (s, 3H), 1.55 (s, 9H).

^{13}C-NMR (101 MHz, $CDCl_3$) δ 170.43, 170.34, 170.24, 169.48, 156.28, 153.20, 145.50, 135.02, 127.45, 125.73, 124.10, 118.93, 100.75, 79.79, 71.32, 70.75, 68.28, 66.94, 61.45, 60.50, 57.02, 28.48, 20.97, 20.76, 20.70, 14.31.

MS (ES+): *m/z* calc. for $C_{30}H_{38}ClNO_{13}$: 655.20; found: 678.2 $[M+Na]^+$.

Probe β-gal N-Boc

Compound **6e** (70 mg, 0.11 mmol) was reacted according to **Procedure H**. Upon completion, the crude product was purified by preparative RP-HPLC (30-100% ACN, ammonium carbonate buffer [30 mM]) to afford **Probe β-gal N-Boc** as a diastereomeric mixture (1:1 ratio) in the form of a white solid (30 mg, 55% yield).

^{1}H-NMR (400 MHz, MeOD) (Diastereomeric mixture) δ 7.48 – 7.39 (m, 2H), 7.31 – 7.25 (m, 1H), 5.04 (dd, *J* = 10.0, 7.8 Hz, 1H), 4.91 – 4.85 (m, 1H), 4.17 – 4.06 (m, 1H), 3.99 – 3.86 (m, 4H), 3.82 – 3.68 (m, 3H), 3.60 (ddd, *J* = 9.7, 3.4, 2.2 Hz, 1H), [3.20, 3.19 (s, 3H)], 1.42 (s, 9H).

^{13}C-NMR (101 MHz, MeOD) (Diastereomeric mixture) δ [156.72, 156.67], 154.16, 133.14, 127.89, [121.62, 121.51], 120.46, 118.02, 109.71, 101.39, [89.54, 89.51], [80.49, 80.46], 75.99, 73.71, 70.78, 68.92, [61.16, 61.11], [49.97, 49.95], 27.30.

MS (ES-): *m/z* calc. for $C_{22}H_{30}ClNO_{11}$: 519.15; found: 518.5 $[M-H]^-$.

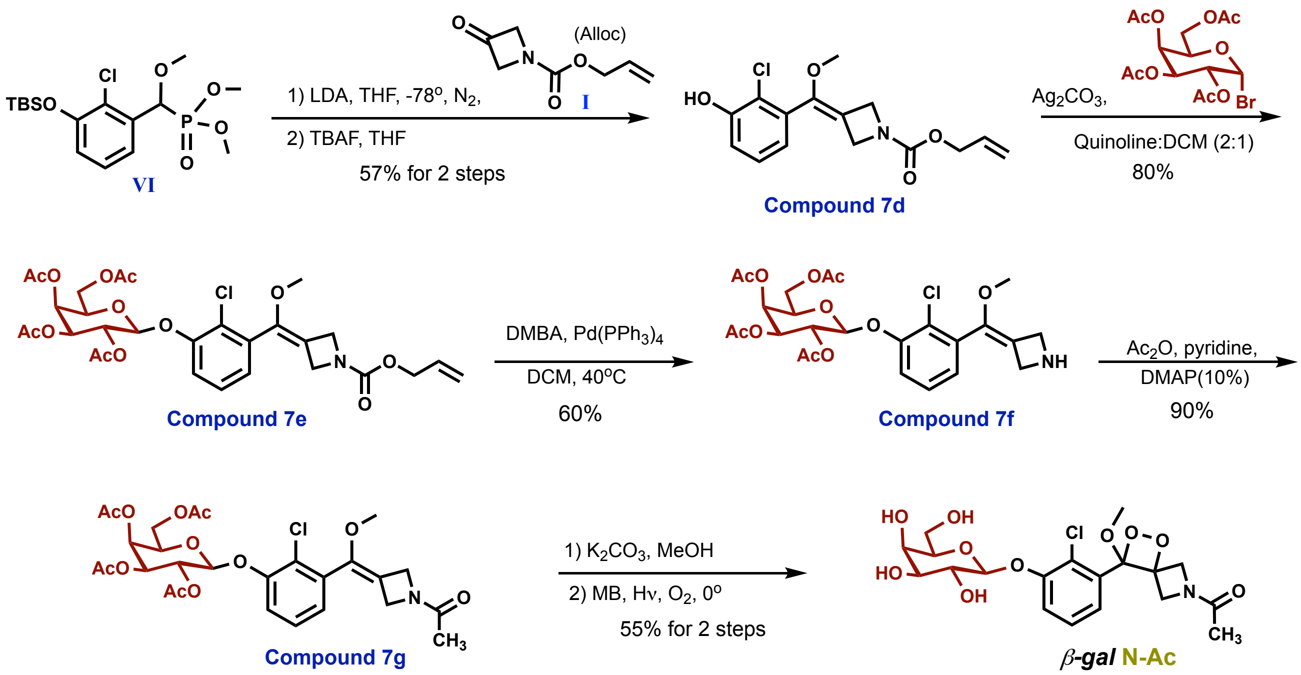

Compound 7d

Compound **7d** was synthesized according to **Procedure F**, using phosphonate **VI**[4] (300 mg, 0.76 mmol) in dry THF (1.5 mL), LDA (2.0M in THF, 0.46 mL, 0.91 mmol) and N-Alloc-3-Azetidinone **I** (117 mg, 0.76 mmol). The reaction was monitored by TLC (90:10, Hex:EtOAc). Upon completion, TBAF (1.0M in THF, 0.84 mL, 0.84 mmol) was added to the solution. The crude residue was purified by column chromatography (60:40, Hex:EtOAc), to afford compound **7d** in the form of a white solid (118 mg, 0.38 mmol, 50%).

^{1}H-NMR (400 MHz, $CDCl_3$) δ 7.13 (t, *J* = 8.0 Hz, 1H), 7.00 (dd, *J* = 8.2, 1.5 Hz, 1H), 6.82 (dd, *J* = 7.5, 1.5 Hz, 1H), 5.95 – 5.85 (m, 1H), 5.28 (d, *J* = 17.1 Hz, 1H), 5.19 (d, *J* = 10.3 Hz, 1H), 4.85 (t, *J* = 2.7 Hz, 2H), 4.56 (d, *J* = 5.3 Hz, 2H), 4.36 (t, *J* = 2.4 Hz, 2H), 3.58 (s, 3H).

^{13}C-NMR (101 MHz, $CDCl_3$) δ 156.51, 152.48, 146.10, 133.44, 132.85, 127.97, 122.37, 119.47, 117.86, 116.99, 105.65, 66.00, 56.96, 56.26, 29.82.

MS (ES+): *m/z* calc. $C_{15}H_{16}ClNO_4$: 309.1; found: 332.3 $[M+Na]^+$.

Compound 7e

To a solution of compound **7d** (118 mg, 0.38 mmol) in DCM (0.2 mL) and quinoline (0.4 mL), Ag_2CO_3 (136 mg, 0.49 mmol) and Acetobromo-α-D-galactose (187 mg, 0.45 mmol) were added following **Procedure G**. The crude residue was purified by column chromatography (50:50, Hex:EtOAc), to afford compound **7e** in the form of a white solid (194 mg, 0.30 mmol, 80%).

^{1}H-NMR (400 MHz, $CDCl_3$) δ 7.21 (dd, *J* = 6.8, 3.2 Hz, 2H), 7.04 (dd, *J* = 5.5, 3.6 Hz, 1H), 5.98 – 5.84 (m, 1H), 5.58 (dd, *J* = 10.5, 8.0 Hz, 1H), 5.47 (d, *J* = 2.8 Hz, 1H), 5.24 (dd, *J* = 34.4, 12.6 Hz, 1H), 5.11 (dd, *J* = 10.5, 3.4 Hz, 1H), 4.97 (d, *J* = 8.0 Hz, 1H), 4.86 (s, 2H), 4.55 (d, *J* = 5.2 Hz, 2H), 4.37 – 4.30 (m, 2H), 4.29 – 4.03 (m, 4H), 3.60 (s, 3H), 2.19 (s, 3H), 2.10 (s, 3H), 2.06 (s, 3H), 2.02 (s, 3H).

^{13}C-NMR (101 MHz, $CDCl_3$) δ 170.32, 170.22, 170.12, 169.35, 156.22, 153.08, 145.65, 134.66, 132.81, 127.37, 125.56, 123.97, 118.91, 117.61, 105.60, 100.62, 71.20, 70.62, 68.14, 66.80, 65.72, 61.32, 56.88, 56.10, 20.85, 20.64, 20.58, 14.18.
MS (ES+): *m/z* calc. $C_{29}H_{34}ClNO_{13}$: 639.17; found: 662.6 $[M+Na]^+$.

Compound 7f

To a solution of compound **7e** (194 mg, 0.30 mmol, 1 eq) in DCM (2 mL), DMBA (94 mg, 0.6 mmol, 2 eq), and $Pd(PPh_3)_4$ (35 mg, 0.03 mmol, 0.1 eq) were added. The reaction was stirred at 40°C and monitored by RP-HPLC (10-90% ACN in water, 0.1%TFA). Upon completion, the solvent was removed under reduced pressure. The crude product purified by column chromatography (90:10, DCM:MeOH) to afford compound **7f** in the form of a white solid (100 mg, 0.18 mmol, 60% yield).
^{1}H-NMR (400 MHz, $CDCl_3$) δ 7.24 – 7.20 (m, 2H), 7.06 – 7.01 (m, 1H), 5.57 (dd, *J* = 10.5, 7.9 Hz, 1H), 5.46 (d, *J* = 2.6 Hz, 1H), 5.10 (dd, *J* = 10.5, 3.4 Hz, 1H), 4.97 (d, *J* = 7.9 Hz, 1H), 4.76 (s, 1H), 4.33 – 4.20 (m, 2H), 4.15 (dd, *J* = 11.3, 6.3 Hz, 1H), 4.05 (t, *J* = 6.5 Hz, 1H), 3.55 (s, 3H), 3.29 (s, 2H), 2.18 (s, 3H), 2.09 (s, 3H), 2.05 (s, 3H), 2.01 (s, 3H).
^{13}C-NMR (101 MHz, $CDCl_3$) δ 170.38, 170.27, 170.17, 169.41, 153.21, 141.68, 139.33, 127.73, 125.52, 123.83, 119.12, 114.16, 100.52, 71.34, 70.67, 68.20, 66.91, 61.43, 57.11, 29.74, 20.94, 20.73, 20.66.
MS (ES+): *m/z* calc. $C_{25}H_{30}ClNO_{11}$: 555.15; found: 556.5 $[M+H]^+$.

Compound 7g

Compound **7f** (100 mg, 0.18 mmol) was dissolved in pyridine (0.66 mL). Acetic anhydride (0.33 mL, 36.35 mmol) and DMAP (3 mg, 0.03 mmol, 0.15 eq) were added. The reaction was monitored by RP-HPLC (10-90% ACN in water, 0.1%TFA). Upon completion, the reaction mixture was purified using preparative RP-HPLC (10-90% ACN in water, 0.1%TFA). The ACN was removed under reduced pressure with the addition of few drops of Et_3N to prevent the appearance of side products. The crude in the water phase was then extracted with EtOAc, and the organic phase was dried over Na_2SO_4, and evaporated under reduced pressure. Compound **7g** was obtained as a diastereomeric mixture (1.4:1 ratio) in the form of a white solid (97 mg, 0.16 mmol, 90% yield).
^{1}H-NMR (400 MHz, $CDCl_3$) (Diastereomeric mixture) δ 7.22 – 7.16 (m, 2H), 7.06 – 6.99 (m, 1H), 5.56 (dd, *J* = 10.5, 8.0 Hz, 1H), 5.45 (d, *J* = 3.1 Hz, 1H), 5.09 (ddd, *J* = 10.5, 3.3, 1.2 Hz, 1H), 4.96 (dd, *J* = 8.0, 3.5 Hz, 1H), 4.92 (t, *J* = 2.8 Hz, 1H), 4.83 (s, 1H), 4.47 – 4.36 (m, 1H), 4.30 (t, *J* = 2.6 Hz, 1H), 4.24 (ddd, *J* = 11.1, 6.9, 4.1 Hz, 1H), 4.18 – 4.11 (m, 1H), 4.04 (dt, *J* = 9.8, 4.9 Hz, 1H), [3.63, 3.53 (s, 3H)], 2.17 (s, 3H), [2.08, 2.07 (s, 3H)], 2.04 (s, 3H), 2.00 (s, 3H), [1.90, 1.82 (s, 3H)].
^{13}C-NMR (101 MHz, $CDCl_3$) (Diastereomeric mixture) δ 170.46, 170.36, 170.33, 170.26, 169.49, 153.20, [146.43, 146.31], [134.94, 134.22], 127.58, [125.68, 125.58], 124.06, [119.11, 119.05], [105.14, 103.73], 100.70, 71.35, [70.75, 70.70], 68.27, 66.92, 61.42, [57.21, 57.10], [56.94, 56.87], [54.74, 54.67], 20.98, 20.77, 20.70, [19.19, 18.97].
MS (ES+): *m/z* calc. $C_{27}H_{32}ClNO_{12}$: 597.16; found: 620.5 $[M+Na]^+$.

Probe β-gal N-Ac

Compound **7g** (70 mg, 0.12 mmol) was reacted according to **Procedure H**. Upon completion, the crude product was purified by preparative RP-HPLC (30-100% ACN, ammonium carbonate buffer [30 mM]) to afford **Probe β-gal N-Ac** as a diastereomeric mixture (% ratio) in the form of a white solid (30 mg, 55% yield).

^{1}H-NMR (400 MHz, MeOD) (Diasetromeric mixture) δ 7.51 – 7.41 (m, 2H), 7.34 – 7.27 (m, 1H), 5.21 – 4.92 (m, 2H), 4.51 – 4.30 (m, 1H), 4.27 – 4.13 (m, 1H), 4.01 (d, *J* = 8.8 Hz, 1H), 3.95 – 3.85 (m, 2H), 3.83 – 3.70 (m, 3H), 3.61 (ddd, *J* = 9.6, 4.4, 3.7 Hz, 1H), [3.23, 3.22 (s, 3H)], [1.92, 1.83 (s, 3H)].

^{13}C-NMR (101 MHz, MeOD) (Diastereomeric mixture) δ [171.89, 171.69], [154.15, 154.03], [132.90, 132.76], 127.85, [121.60, 121.46], 120.31, [118.22, 117.80], 109.46, [101.45, 101.24, 101.15], [88.67, 88.57], 75.84, 73.53, 70.62, [68.79, 68.75], 60.98, [60.44, 58.57], [57.83, 56.13], 49.89, 17.60.

MS (ES+): *m/z* calc. for $C_{19}H_{24}ClNO_{10}$: 461.11; found: 462.4 $[M+H]^+$.

Synthetic procedures for the β-gal-masked-acrylate 1,2-dioxetanes

Synthesis of Adamantyl-1,2-dioxetanes

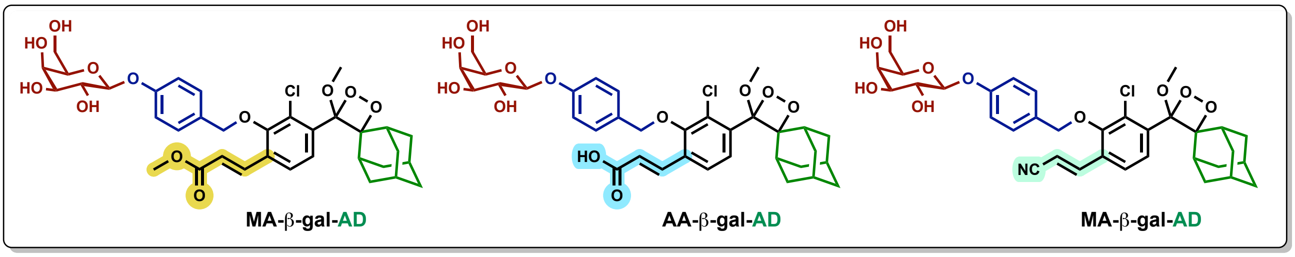

Probe MA-β-gal-AD and **Probe CN-β-gal-AD** were synthesized according to a known procedure.[2]

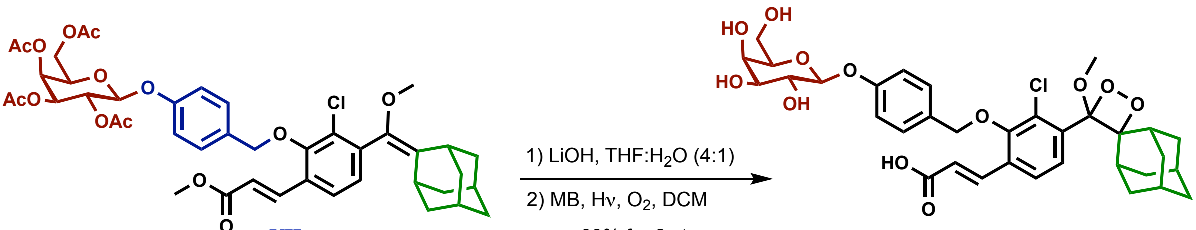

Probe AA-β-gal-AD

Compound **VII** [2] (61 mg, 0.07 mmol) and LiOH (18 mg, 0.7 mmol) were dissolved in 1 mL solution of 4:1 THF: H_2O. The reaction mixture was stirred at 40°C and monitored by RP-HPLC (50-100% ACN in water with 0.1% TFA). Upon completion, the reaction mixture was diluted with EtOAc and washed with 1M HCl followed by an additional wash with brine. The organic phase was dried over Na_2SO_4 and concentrated under reduced pressure. The crude product was further reacted without purification. The crude product and catalytic amount of methylene blue (~1 mg) were dissolved in 10 mL of DCM. Oxygen was bubbled through the solution at room temperature while irradiating with yellow light. The reaction was monitored by RP-HPLC (50-100% ACN in water, 0.1% TFA). After completion, the reaction mixture was concentrated by evaporation under reduced pressure. The crude product was purified by preparative RP-HPLC (50-100% ACN in water, 0.1% TFA) to afford **Probe AA-β-gal-AD** in the form of a white solid (31 mg, 63% yield).

^{1}H-NMR (400 MHz, DMSO) δ 7.95 (d, J = 8.4 Hz, 1H), 7.83 – 7.72 (m, J = 12.2, 6.2 Hz, 2H), 7.38 (d, J = 7.6 Hz, 2H), 7.04 (d, J = 8.6 Hz, 2H), 6.65 (d, J = 16.1 Hz, 1H), 4.93 – 4.79 (m, 3H), 3.70 (d, J = 3.3 Hz, 2H), 3.61 – 3.38 (m, 6H), 3.12 (s, 3H), 2.89 (s, 1H), 2.24 (d, J = 11.9 Hz, 1H), 1.91 (s, 1H), 1.77 – 1.15 (m, 12H).

^{13}C-NMR (101 MHz, DMSO) δ 167.60, 158.21, 154.05, 137.19, 134.52, 131.82, 130.64, 129.32, 128.82, 127.30, 126.51, 123.41, 116.65, 111.66, 101.39, 95.88, 75.95, 73.74, 70.73, 68.56, 60.79, 49.88, 36.33, 33.75, 33.54, 32.28, 32.12, 31.56, 31.33, 25.97, 25.64.
MS (ES+): m/z calc. for $C_{34}H_{39}ClO_{12}$: 674.21; found:675.6 $[M-H]^+$, 697.6 $[M+Na]^+$

Synthesis of Cyclobutyl-1,2-dioxetanes

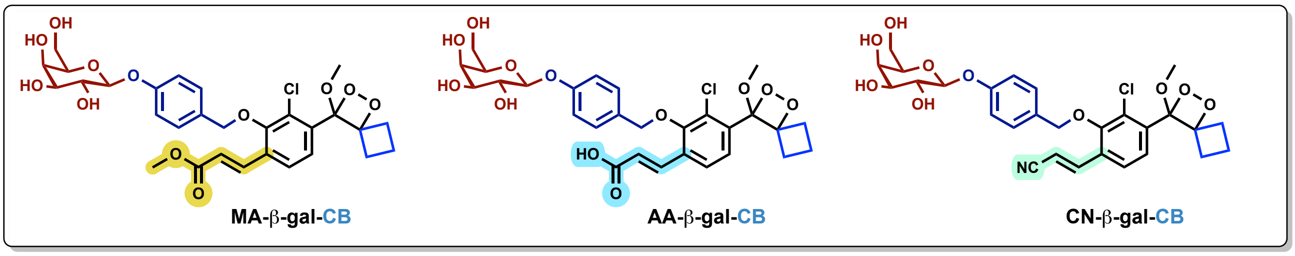

Probes MA-β-gal-CB, **AA-β-gal-CB** and **CN-β-gal-CB** were synthesized according to the following procedures.

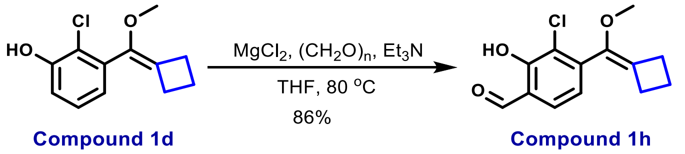

Compound 1h

Et_3N (743 µL, 5.32 mmol) was added into a solution of compound **1d** (300 mg, 1.33 mmol) in dry THF (4 mL). Then, $MgCl_2$ (253 mg, 2.66 mmol) and paraformaldehyde (319 mg, 10.6 mmol) were added. The reaction was heated under reflux and was monitored by TLC (90:10, Hex:EtOAc). Upon completion, the reaction mixture was diluted with EtOAc and washed with 1M HCl. The organic layer was separated, washed with brine, dried over Na_2SO_4, and evaporated under reduced pressure. The residue was purified by column chromatography (90:10, Hex:EtOAc) affording compound **1h** in the form of a pale-yellow oil (289 mg, 1.14 mmol, 86% yield).
^{1}H-NMR (400 MHz, $CDCl_3$) δ 11.63 (s, 1H), 9.88 (s, 1H), 7.47 (d, J = 8.0 Hz, 1H), 7.02 (d, J = 8.0 Hz, 1H), 3.55 (s, 3H), 3.02 – 2.91 (m, 2H), 2.62 – 2.52 (m, 2H), 2.03 (quin, J = 7.9 Hz, 2H).
^{13}C-NMR (101 MHz, $CDCl_3$) δ 195.69, 157.76, 143.22, 142.81, 130.93, 123.97, 121.81, 121.62, 120.29, 57.78, 28.95, 28.62, 17.51.
MS (ES-): m/z calc. $C_{13}H_{13}ClO_3$: 252.06; found: 251.2 $[M-H]^-$.

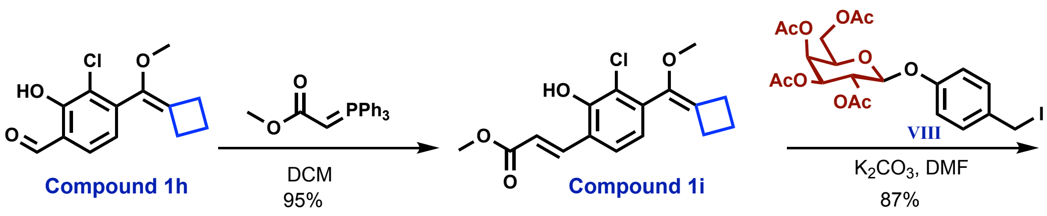

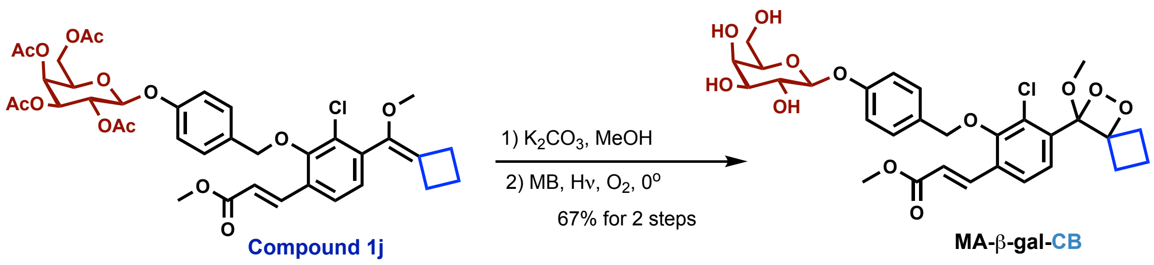

Compound 1i

To a solution of compound **1h** (100 mg, 0.39 mmol) in DCM (1 mL), methyl (triphenyl-phosphoranylidene) acetate (130 mg, 0.39 mmol) was added. The reaction was stirred for 15 min and monitored by TLC (80:20, Hex:EtOAc). Upon completion, the reaction mixture was diluted with DCM and washed with 1M HCl followed by an additional wash with brine. The organic layer was then dried over Na_2SO_4, filtered, and concentrated under reduced pressure. The crude residue was purified by column chromatography (80:20, Hex:EtOAc) to obtain compound **1i** in the form of a white solid (114 mg, 0.37 mmol, 95%).

^{1}H-NMR (400 MHz, $CDCl_3$) δ 7.95 (d, *J* = 16.2 Hz, 1H), 7.34 (d, *J* = 8.1 Hz, 1H), 6.86 (d, *J* = 8.1 Hz, 1H), 6.64 (brs, 1H), 6.58 (d, *J* = 16.2 Hz, 1H), 3.79 (s, 3H), 3.49 (s, 3H), 2.92 (t, *J* = 6.4 Hz, 2H), 2.52 (t, *J* = 9.1 Hz, 2H), 1.97 (quin, *J* = 8.0 Hz, 2H).

^{13}C-NMR (101 MHz, $CDCl_3$) δ 167.91, 151.04, 143.77, 139.48, 136.25, 126.80, 122.67, 122.12, 120.51, 119.52, 57.63, 51.87, 28.99, 28.60, 17.60.

MS (ES-): *m/z* calc. $C_{16}H_{17}ClO_4$:308.08; found: 307.3 $[M-H]^-$.

Compound 1j

Compound **1i** (83 mg, 0.27 mmol) was dissolved in dry DMF (1 mL). K_2CO_3 (45 mg, 0.32 mmol) was added, and the solution was stirred for 10 minutes at room temperature before compound **VIII** [2] (152 mg, 0.27 mmol) was added. The reaction mixture was stirred for 1 hour and monitored by TLC (50:50, Hex:EtOAc). After completion, the reaction mixture was diluted with EtOAc and washed with saturated 1M HCl. The organic layer was separated, washed with brine, dried over Na_2SO_4, and evaporated under reduced pressure. The crude product was purified by column chromatography (50:50, Hex:EtOAc) to obtain compound **1j** in the form of a white solid (186 mg, 0.23 mmol, 87%) .

^{1}H-NMR (400 MHz, $CDCl_3$) δ 7.83 (d, *J* = 16.2 Hz, 1H), 7.38 (dd, *J* = 8.3, 3.4 Hz, 3H), 7.06 (d, *J* = 8.1 Hz, 1H), 6.98 (d, *J* = 8.5 Hz, 2H), 6.38 (d, *J* = 16.2 Hz, 1H), 5.47 – 5.39 (m, 2H), 5.09 (dd, *J* = 10.4, 3.4 Hz, 1H), 5.03 (d, *J* = 7.9 Hz, 1H), 4.86 (s, 2H), 4.20 – 4.10 (m, 2H), 4.06 (t, *J* = 7.1 Hz, 1H), 3.73 (s, 3H), 3.47

(s, 3H), 2.91 (t, *J* = 6.5 Hz, 2H), 2.47 (t, *J* = 6.5 Hz, 2H), 2.13 (s, 3H), 2.02 (s, 3H), 2.01 (s, 3H), 1.96 (s, 3H), 1.95 – 1.89 (m, 2H).
^{13}C-NMR (101 MHz, $CDCl_3$) δ 170.45, 170.36, 170.18, 169.51, 167.14, 157.32, 153.94, 143.80, 138.88, 137.89, 131.13, 130.48, 129.69, 128.72, 127.04, 125.26, 121.84, 119.91, 117.08, 99.71, 75.68, 71.14, 70.93, 68.77, 67.07, 61.51, 57.57, 51.83, 20.78, 20.72, 20.66.
MS (ES+): *m/z* calc. $C_{37}H_{41}ClO_{14}$: 744.2; found: 767.6 $[M+Na]^+$.

Probe MA-β-gal-CB

Compound **1j** (50 mg, 0.06 mmol) was dissolved in MeOH (1 mL). K_2CO_3 (41 mg, 0.30 mmol) was added, and the solution was stirred at room temperature for 30 minutes. The reaction was monitored by RP-HPLC (30-100% ACN, ammonium carbonate buffer [30 mM]). Upon completion, the reaction mixture was diluted with EtOAc and washed with brine. The organic phase was dried over Na_2SO_4, filtered, and concentrated under reduced pressure. The crude product was further reacted without purification. The crude product and catalytic amount of methylene blue (~1 mg) were dissolved in 10 mL of DCM and cooled to 0°C. Oxygen was bubbled through the solution while irradiating with yellow light. The reaction was monitored by RP-HPLC (30-100% ACN, ammonium carbonate buffer [30 mM]). After completion, the reaction mixture was concentrated by evaporation under reduced pressure at 10°C. The crude product was purified by preparative RP-HPLC (30-100% ACN, ammonium carbonate buffer [30 mM]) to afford **Probe MA-β-gal-CB** in the form of a white solid (50 mg, 67% yield).
^{1}H-NMR (400 MHz, MeOD) δ 7.85 (d, *J* = 16.2 Hz, 1H), 7.73 (d, *J* = 8.3 Hz, 1H), 7.52 (d, *J* = 8.3 Hz, 1H), 7.34 (d, *J* = 8.6 Hz, 2H), 7.09 (d, *J* = 8.7 Hz, 2H), 6.56 (d, *J* = 16.2 Hz, 1H), 5.03 – 4.95 (m, 2H), 3.90 (dd, *J* = 3.3, 0.5 Hz, 1H), 3.80 (s, 3H), 3.83 – 3.72 (m, 3H), 3.71 – 3.67 (m, 1H), 3.58 (dd, *J* = 9.7, 3.4 Hz, 1H), 3.27 – 3.16 (m, 1H), 3.22 (s, 3H), 2.39 – 2.28 (m, 1H), 2.26 – 2.15 (m, 1H), 2.14 – 2.03 (m, 1H), 1.82 – 1.72 (m, 2H).
^{13}C-NMR (101 MHz, MeOD) δ 167.30, 158.45, 154.08, 138.31, 136.39, 131.54, 130.62, 129.59, 126.86, 125.85, 124.86, 120.57, 116.49, 110.56, 101.64, 95.43, 76.01, 75.72, 73.58, 70.97, 68.93, 61.14, 51.16, 49.95, 32.61, 30.39, 11.88.
MS (ES+): *m/z* calc. $C_{29}H_{33}ClO_{12}$: 608.17; found: 631.6 $[M+Na]^+$.

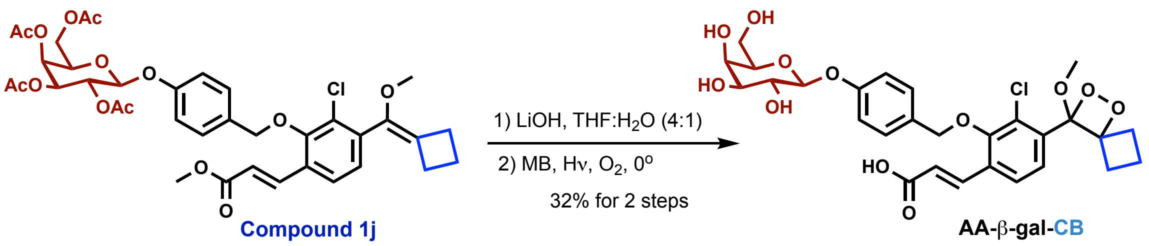

Probe AA-β-gal-CB

Compound 1j (60 mg, 0.08 mmol) and LiOH (19 mg, 0.8 mmol) were dissolved in 1 mL solution of 4:1 THF: H_2O. The reaction mixture was stirred at 40°C and monitored by RP-HPLC (10-90% ACN,

ammonium carbonate buffer [30 mM]). Upon completion, the reaction mixture was diluted with EtOAc and washed with 1M HCl followed by an additional wash with brine. The organic phase was dried over Na_2SO_4 and concentrated under reduced pressure. The crude product was further reacted without purification. The crude product and catalytic amount of methylene blue (~1 mg) were dissolved in 10 mL of DCM and cooled to 0°C. Oxygen was bubbled through the solution while irradiating with yellow light. The reaction was monitored by RP-HPLC (10-90% ACN, ammonium carbonate buffer [30 mM]). After completion, the reaction mixture was concentrated by evaporation under reduced pressure. The crude product was purified by preparative RP-HPLC (10-90% ACN, ammonium carbonate buffer [30 mM]) to afford **Probe AA-β-gal-CB** in the form of a white solid. (16 mg, 32% yield)

^{1}H NMR (400 MHz, MeOD) δ 7.81 (d, *J* = 16.1 Hz, 1H), 7.71 (d, *J* = 8.3 Hz, 1H), 7.50 (d, *J* = 8.3 Hz, 1H), 7.41 (d, *J* = 8.4 Hz, 2H), 7.10 (d, *J* = 8.6 Hz, 2H), 6.59 (d, *J* = 16.1 Hz, 1H), 5.00 (d, *J* = 2.7 Hz, 2H), 3.94 – 3.91 (m, 1H), 3.84 – 3.68 (m, 5H), 3.60 (dd, *J* = 9.7, 3.3 Hz, 1H), 3.21 (s, 3H), 3.26 – 3.15 (m, 1H), 2.38 – 2.30 (m, 1H), 2.29 – 2.17 (m, 1H), 2.14 – 2.07 (m, 1H), 1.82 – 1.71 (m, 2H).

^{13}C NMR (101 MHz, MeOD) δ 172.99, 159.55, 154.90, 136.49, 136.21, 133.87, 131.74, 131.13, 128.00, 126.86, 126.02, 117.76, 117.67, 111.91, 102.89, 96.68, 76.95, 74.85, 72.26, 70.22, 62.40, 51.17, 49.00, 33.88, 31.60, 13.12.

MS (ES-): *m/z* calc. $C_{28}H_{31}ClO_{12}$: 594.15; found: 593.4 $[M-H]^-$.

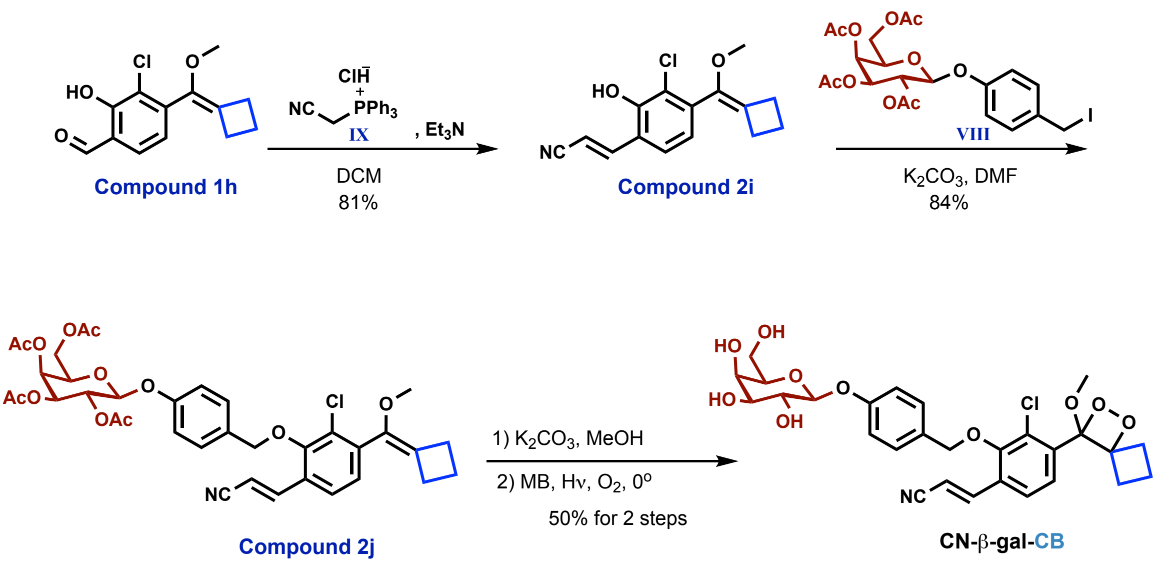

Compound 2i

To a solution phosphonium salt **IX**[5] (145 mg, 0.43 mmol) in DCM (1 mL), Et_3N (180 μL, 1.29 mmol) and compound **1h** (100 mg, 0.39 mmol) were added. The reaction was stirred for 20 min and monitored by TLC (80:20, Hex:EtOAc). Upon completion, the reaction mixture was diluted with DCM and washed with 1M HCl followed by an additional wash with brine. The organic layer was then dried over Na_2SO_4, filtered, and concentrated under reduced pressure. The crude residue was purified by

column chromatography (80:20, Hex:EtOAc) to obtain compound **2i** in the form of a white solid (90 mg, 0.33 mmol, 84%).

^{1}H-NMR (400 MHz, $CDCl_3$) δ 7.55 (d, *J* = 16.8 Hz, 1H), 7.25 (d, *J* = 8.1 Hz, 1H), 6.92 (d, *J* = 8.1 Hz, 1H), 6.32 (s, 1H), 6.15 (d, *J* = 16.8 Hz, 1H), 3.52 (s, 3H), 2.99 – 2.92 (m, 2H), 2.59 – 2.51 (m, 2H), 2.01 (quin, *J* = 8.0 Hz, 2H).

^{13}C-NMR (101 MHz, $CDCl_3$) δ 150.63, 145.31, 143.35, 137.02, 126.80, 123.11, 122.77, 120.97, 120.47, 118.57, 98.51, 57.67, 28.92, 28.57, 17.50.

MS (ES-): *m/z* calc. $C_{15}H_{14}ClNO_2$:275.07; found: 274.3 $[M-H]^-$.

Compound 2j

Compound **2i** (90 mg, 0.33 mmol) was dissolved in dry DMF (1 mL). K_2CO_3 (55 mg, 0.40 mmol) was added, and the solution was stirred for 10 minutes at room temperature before compound **VIII** [2] (186 mg, 0.33 mmol) was added. The reaction mixture was stirred for 1 hour and monitored by TLC (50:50, Hex:EtOAc). After completion, the reaction mixture was diluted with EtOAc and washed with saturated 1M HCl. The organic layer was separated, washed with brine, dried over Na_2SO_4, and evaporated under reduced pressure. The crude product was purified by column chromatography (50:50, Hex:EtOAc) to obtain compound **2j** in the form of a white solid (201 mg, 0.28 mmol, 84%).

^{1}H-NMR (400 MHz, $CDCl_3$) δ 7.44 (d, *J* = 16.8 Hz, 1H), 7.35 (d, *J* = 8.6 Hz, 2H), 7.28 (d, *J* = 8.1 Hz, 1H), 7.12 (d, *J* = 8.1 Hz, 1H), 7.04 (d, *J* = 8.6 Hz, 2H), 5.84 (d, *J* = 16.8 Hz, 1H), 5.53 – 5.44 (m, 2H), 5.16 – 5.06 (m, 2H), 4.94 (s, 2H), 4.20 (qd, *J* = 11.2, 6.6 Hz, 2H), 4.12 – 4.07 (m, 1H), 3.56 (s, 3H), 2.98 (t, *J* = 9.2 Hz, 2H), 2.53 (t, *J* = 9.2 Hz, 2H), 2.18 (s, 3H), 2.08 (s, 3H), 2.06 (s, 3H), 2.06 – 1.96 (m, 2H), 2.01 (s, 3H).

^{13}C-NMR (101 MHz, $CDCl_3$) δ 170.51, 170.41, 170.25, 169.61, 157.53, 153.60, 145.00, 143.59, 139.09, 130.75, 130.49, 128.96, 128.89, 127.16, 124.61, 122.37, 118.15, 117.39, 99.83, 98.19, 77.18, 76.86, 75.82, 71.20, 70.97, 68.76, 67.03, 61.47, 57.78, 20.89, 20.81, 20.72.

MS (ES+): *m/z* calc. $C_{36}H_{38}ClNO_{12}$: 711.21; found: 734.6 $[M+Na]^+$.

Probe CN-β-gal-CB

Compound **2j** (50 mg, 0.07 mmol) was dissolved in MeOH (1 mL). K_2CO_3 (47 mg, 0.34 mmol) was added, and the solution was stirred at room temperature for 30 minutes. The reaction was monitored by RP-HPLC (30-100% ACN, ammonium carbonate buffer [30 mM]). Upon completion, the reaction mixture was diluted with EtOAc and washed with brine. The organic phase was dried over Na_2SO_4, filtered, and concentrated under reduced pressure. The crude product was further reacted without purification. The crude product and catalytic amount of methylene blue (~1 mg) were dissolved in 10 mL of DCM and cooled to 0°C. Oxygen was bubbled through the solution while irradiating with yellow light. The reaction was monitored by RP-HPLC (30-100% ACN, ammonium carbonate buffer [30 mM]). After completion, the reaction mixture was concentrated by evaporation under reduced pressure at 10°C. The crude product was purified by preparative RP-HPLC (30-100% ACN, ammonium carbonate buffer [30 mM]) to afford **Probe CN-β-gal-CB** in the form of a white solid (20 mg, 50% yield).

^{1}H-NMR (400 MHz, MeOD) δ 7.65 (d, *J* = 8.3 Hz, 1H), 7.52 (d, *J* = 8.3 Hz, 1H), 7.50 (d, *J* = 16.9 Hz, 1H), 7.29 (d, *J* = 8.4 Hz, 2H), 7.10 (d, *J* = 8.6 Hz, 2H), 6.19 (d, *J* = 16.8 Hz, 1H), 5.10 – 5.01 (m, 2H), 4.91 – 4.87 (m, 2H), 3.90 (d, *J* = 2.8 Hz, 1H), 3.82 – 3.75 (m, 3H), 3.73 – 3.67 (m, 1H), 3.58 (dd, *J* = 9.7, 3.4 Hz, 1H), 3.23 (s, 3H), 3.26 – 3.16 (m, 1H), 2.40 – 2.28 (m, 1H), 2.26 – 2.17 (m, 1H), 2.14 – 2.05 (m, 1H), 1.82 – 1.73 (m, 2H).
^{13}C-NMR (101 MHz, MeOD) δ 158.59, 153.52, 144.35, 137.16, 131.19, 130.74, 129.32, 126.95, 125.32, 124.88, 117.69, 116.64, 110.47, 101.64, 99.24, 95.43, 76.02, 75.73, 73.56, 70.96, 68.95, 61.16, 49.99, 32.60, 30.41, 11.89.
MS (ES+): *m/z* calc. C28H30ClNO10: 575.16; found: 598.6 [M+Na]$^+$.

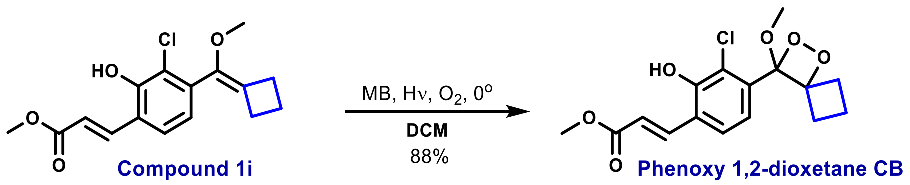

Probe Phenoxy 1,2-dioxetane CB

Compound **1i** (20 mg, 0.06 mmol) and catalytic amount of methylene blue were dissolved in 5 mL of DCM and cooled to 0°C. Then, oxygen was bubbled through the solution while irradiating with yellow light. The reaction was monitored by RP-HPLC (50-100% ACN in water, 0.1% TFA). Upon completion (about 5 min) the crude product was immediately passed through a silica gel column (60:40, Hex:EtOAc) to filter out methylene blue. The solvents were removed under reduced pressure while cooling the bath to 10°C. **Probe Phenoxy 1,2-dioxetane CB** (19 mg, 88%) was obtained as a white solid.
^{1}H NMR (400 MHz, CD_3CN) δ 7.91 (d, *J* = 16.2 Hz, 1H), 7.64 (d, *J* = 8.2 Hz, 1H), 7.25 (d, *J* = 8.2 Hz, 1H), 6.63 (d, *J* = 16.2 Hz, 1H), 3.76 (s, 3H), 3.19 (s, 3H), 3.19 – 3.11 (m, 1H), 2.27 – 2.16 (m, 2H), 2.16 – 2.08 (m, 1H), 1.80 – 1.67 (m, 2H).
^{13}C NMR (101 MHz, CD_3CN) δ 167.89, 152.76, 139.03, 138.76, 136.00, 127.83, 127.35, 121.35, 121.29, 96.27, 52.27, 51.23, 33.44, 31.32, 12.78.

Experimental protocols

Determination of the ratio of ene-products and 1,2-dioxetanes (Figure 3, and Figure S7 in Appendix II).

cyclooctyl-, cycloheptyl-, cyclohexyl-, cyclopentyl, and cyclobuyl- enol ethers were dissolved in DCM (10 mg/ml), followed by the addition of a catalytic amount of methylene blue. Then, oxygen was bubbled through the solution while irradiating with a yellow light at room temperature. The reaction was terminated after 5 minutes by fast filtration of the methylene blue through a short silica pad. The filtrate containing the reaction mixture was stored at -20°C, product ratio was analyzed by RP-HPLC (90-100% ACN in water with 0.1% TFA) and TLC (90:10, Hex:EtOAc). The ene-products and 1,2-dioxetanes were fully characterized and their spectral data appears in the section **general synthetic procedures for the formation of TBS-masked Diox 1-15 and Ene-product 1-3.**

Stability assay of Diox 3 and Diox 6 -Diox 15 (Table 1 and Figures S8-S9 in Appendix II).

Diox 3 and **Diox 6** – **Diox 15** stock solutions were prepared in ACN at a final concentration of 10 mM. To a vial containing 270μL of PBS, 15μL of ACN and 15μL of dioxetane stock solution were added and vortexed until a clear solution was obtained. In the case of a slightly cloudy solution, an additional 15μL ACN was added. The vials were kept at room temperature in the dark. HPLC analysis was conducted for each compound at T=0, 0.5, 4, 8, 16, and 32 hours, and the ratio between the percentage of the 1,2-dioxetane and forming benzoate was evaluated at 270 nm by calculating the area under each peak. Three independent measurements were conducted for each dioxetane.

Stability assay of Diox 1, 2, 4, 5, and Diox 8 (Table 1 and Figures S10-S11 in Appendix II).

Diox 1, 2, 4, 5, and **Diox 8** stock solutions were prepared in ACN at a final concentration of 10 mM. To a vial containing 900μL of PBS, 50μL of ACN and 50μL of dioxetane stock solution were added and vortexed until a clear solution was obtained. In the case of a slightly cloudy solution, an additional 50μL ACN was added. The vials were heated to 50°C in an oil bath and covered with aluminum foil for the entire assay duration. HPLC analysis was conducted for each compound at T=0, 0.5, 4, and 8 hours, and the ratio between the percentage of the 1,2-dioxetane and forming benzoate was evaluated at 270 nm by calculating the area under each peak. Three independent measurements were conducted for each dioxetane.

Chemiluminescent kinetic measurements of Diox 1- Diox 15 (Table 1 and Figures S12-S15 in Appendix II).

Diox 1 – Diox-15 stock solutions were prepared in ACN at a final concentration of 10 mM. Chemiluminescent kinetic profiles were recorded using Spectramax iD3 with an injector cartridge. The injector settings were fixed on the following parameters: Integration time: 50 msec, injection volume: 10μL, and measuring interval time: 50 msec (with the exception of **Diox -1** and **Diox - 3** measurements in DMSO in which the measuring interval was set to 300 msec). The injectors were pre-washed with water, EtOH (70%), and DMSO, and primed with a solution of 100 nM of **Diox 1** – **Diox 15** in ACN before any measurements. Measurements were conducted in a white 96-well Corning™ plate, each well contained 89 μL DMSO or Acetone and 1μL of TBAF (1M in THF), with a final volume of 100μL after the addition via injection of 10μL of **Diox** [100 nM] solution. TBAF was added fresh to each well at the beginning of a measurement, and new aliquots were used if measurements were conducted on separate days. Measurements were repeated in three independent experiments.

Camera-based Image Processing Protocol

The light emission profile was recorded using the ProCamera software on an iPhone 13 Pro camera. Shutter speed and ISO settings were set during the filming . To a vial containing 1mL of DMSO was added 10μL of TBAF (1M in THF) [10 mM] followed by the addition of 50μL of the dioxetane probe [500 μM]. The recording was taken at 60 to 240 fps speed based on the dioxetane kinetics to allow high sensitivity and yet small enough file size to analyze. The kinetic profile was extracted using ImageJ software by plotting the mean grey area profile over time. $t_{1/2}$ values were determined by integration of the kinetic profile curve and extraction of the time at which the normalized intensity was at 0.5. The image sequence was compiled using Adobe Photoshop.

Visual demonstration of Diox 1, Diox 8, and Diox 14 (Figure 4).

Light emission was recorded using a standard camera or iPhone 13Pro. To a vial containing 500μL of Acetone was added 10μL of TBAF (1M in THF) and stirred for a few seconds, then 25μL of **Diox 1** /**Diox 8**/ **Diox 14** was added, and the light emission was recorded for 2 minutes in total.

Visual demonstration of Coumarin-AD, Coumarin-CB, and Coumarin-OX (Figure 4).

Light emission was recorded using a standard camera or iPhone 13Pro. To a vial containing 500μL PBS (pH 7.4) 50μL of stock solution of **Coumarin-AD**/ **Coumarin-CB**/ **Coumarin-OX** was added, and the light emission was recorded for 20 minutes in total.

Chemiluminescent kinetic measurements of β-gal dioxetanes (Figure 5 - 6 and Figures S16-S44 in Appendix II).

All stock solutions were prepared in ACN at a final concentration of 10 mM. Measurements were recorded by Spectramax iD3 with integration time parameters set at 140 msec. β-gal dioxetanes were measured in a white 96-well Corning™ plate, in a final well volume of 100 μL, 1% ACN unless otherwise mentioned. Measurements of β-gal Schaap dioxetanes (Figure 5) were conducted with the addition of 5% Emerald II in PB, pH 7.4. *β-galactosidase* was added in various concentrations to the well and the light emission was recorded immediately. Measurements were repeated in three independent experiments.

Bacterial experiments

Detection of β-galactosidase activity in a live bacterial assay

All bacterial strains used in this study were purchased from the American Type Culture Collection (ATCC): *Escherichia coli* (ATCC 9637) or clinical isolate: *Escherichia coli* (MF -3). Starter cultures were grown overnight with shaking at 37°C in a brain heart infusion (BHI) medium. Then cells were diluted 1:100 with fresh BHI medium and were grown for 2 hours with shaking at 37°C. Cultures were then centrifuged, washed with PBS buffer, and resuspended in PBS buffer (pH 7.4) to obtain OD_{600} of 1.0. 50µL sample of pre-incubated tested probe (30 min, 37°C) in PBS (2% ACN) was added to 96-well plate pre-loaded with a 50µL aliquot of bacterial cell sample to obtain a final concentration of 10µM (1% ACN) and final OD_{600} of 0.5. For limit of detection assay, serial of 1:5 dilution of *Escherichia coli* concentrations starting from $O.D_{600}$ 0.1 in PBS were prepared. Next, the total light emission and the chemiluminescence emission spectra of MA-β-gal-CB and MA-β-gal-AD probes were measured at 37°C using a Molecular Devices Spectramax iD3. Measurements were repeated in three independent experiments.

IVIS-visualization

As described above, a pre-loaded 96-well plate with 50µL of serial dilution (1:2) of *Escherichia coli* starting from $O.D_{600}$ 0.1 in PBS was prepared. Then, 50µL samples of MA-β-gal-CB or MA-β-gal-AD probes in PBS (2% ACN) were added to obtain a final concentration of 10µM (1% ACN). Next the chemiluminescence emission spectra of the probes were measured using IVIS® Lumina imager. The images were taken after 1 min of incubation with exposure time parameter set on 10 sec.

For agar plates pictures, *Escherichia coli* cultures were streaked from frozen culture onto Luria-Bertani (LB) agar plates and incubated for 24 h at 37°C. Then, each agar plate was sprayed homogeneously with 100µM (1%ACN) MA-β-gal-CB or MA-β-gal-AD probes. Next the chemiluminescence emission spectra of the probes were measured using IVIS lumina imager. The images were taken after 0.5 min of incubation with exposure time parameter set on 30 sec.

Appendix I: Computational Data

Computational methods

All the calculations were carried out using Gaussian 16 program.[6] The geometries were optimized using ωB97X-D[7] functional with 6-31G(d)[8, 9] basis set with SMD solvent model[10] to describe the DMSO environment. Single point energies were calculated using CAM-B3LYP-D3(BJ)[11-13] functional, 6-311++G(2d,2p) basis set[8, 9] and SMD solvent model.[10] The stabilities of wavefunctions of diradical species were checked to ensure convergence to the lowest energy wavefunction. Quasiharmonic[14] and concentration corrections to enthalpy and entropy were made using Paton's GoodVibes software.[15] Conical Intersection (CI) geometries were located using Spin-Flip TDDFT[16, 17] in ORCA 5.0.3 program[18] with BHandHLYP[19] functional, def2-SVP[20] basis set, and SMD solvent model.[10] The rate constants we reported are calculated via TST using Eyring's equation ($k = \frac{k_B T}{h} e^{-\frac{\Delta G^{\ddagger}}{RT}}$), where the free energies of **TS1** are used as the overall free energy barriers since it is the rate determine step.

Keywords of Spin Flip TDDFT calculation:

```
! BHANDHLYP def2-SVP def2/J RIJCOSX miniprint tightSCF CI-opt
%CPCM
SMD TRUE
SMDSOLVENT "DMSO"
END
%CONICAL
ETOL 1e-3
END
%geom
MaxStep 0.1 # maximum step length
Trust -0.1 # trust radius in internal coordinates
end
%tddft
IROOT 1
JROOT 2
nroots 5
SF True
end
```

Results

For each of the following Figures, the results of the computations by the methods described above are given. In each case the TS1_OO involves mainly OO cleavage. The charges and spin densities on relevant atoms indicate that this is a homolytic cleavage in the case of adamantyl and dimethyl substitution, but also involves substantial charge transfer for the spiro-cyclobutyl and oxetanyl cases. In all cases, the Int1 is a radical anion, with both more negative charge on the ketal O and spin on the cycloalkanone (or acetone) O. The very low barrier for TS2_CC involves CC cleavage and formation of excited phenoxide ester. The exothermic formation of the excited state of the product is notable, and the origin of the chemiluminescence.

To compare the solvent effects on excitation energies with the state-specific (SS) method, we have tried calculating emission energies of Int2 using state-specific (SS) and corrected linear response (cLR) method. However, we found there is no significant difference in results between these methods and the default linear response method.

Method	Energy (eV)	Wavelength(nm)
LR	3.0251	409.86
cLR	3.0243	409.96
SS	2.9664	418.02

Detailed computational investigation of spiro-adamantyl-phenoxy-1,2-dioxetane and spiro-cyclobutyl-phenoxy-1,2-dioxetane

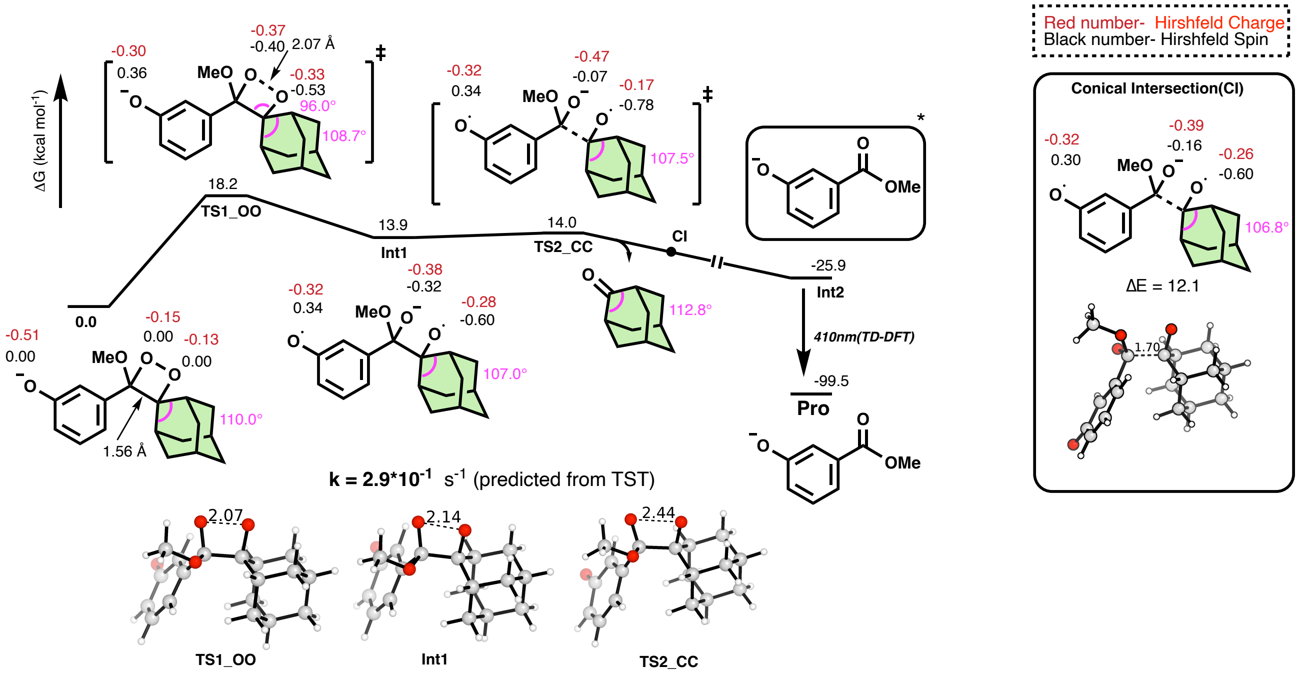

Figure S1: Spiro-**adamantyl**-phenoxy-1,2-dioxetane computed Gibbs free energy chemiexcitation profile, essential geometric features along the reaction path, charges, and spin densities on important atoms involved in the reaction. Rate constants were predicted from computed activation free energies using transition state theory (TST).

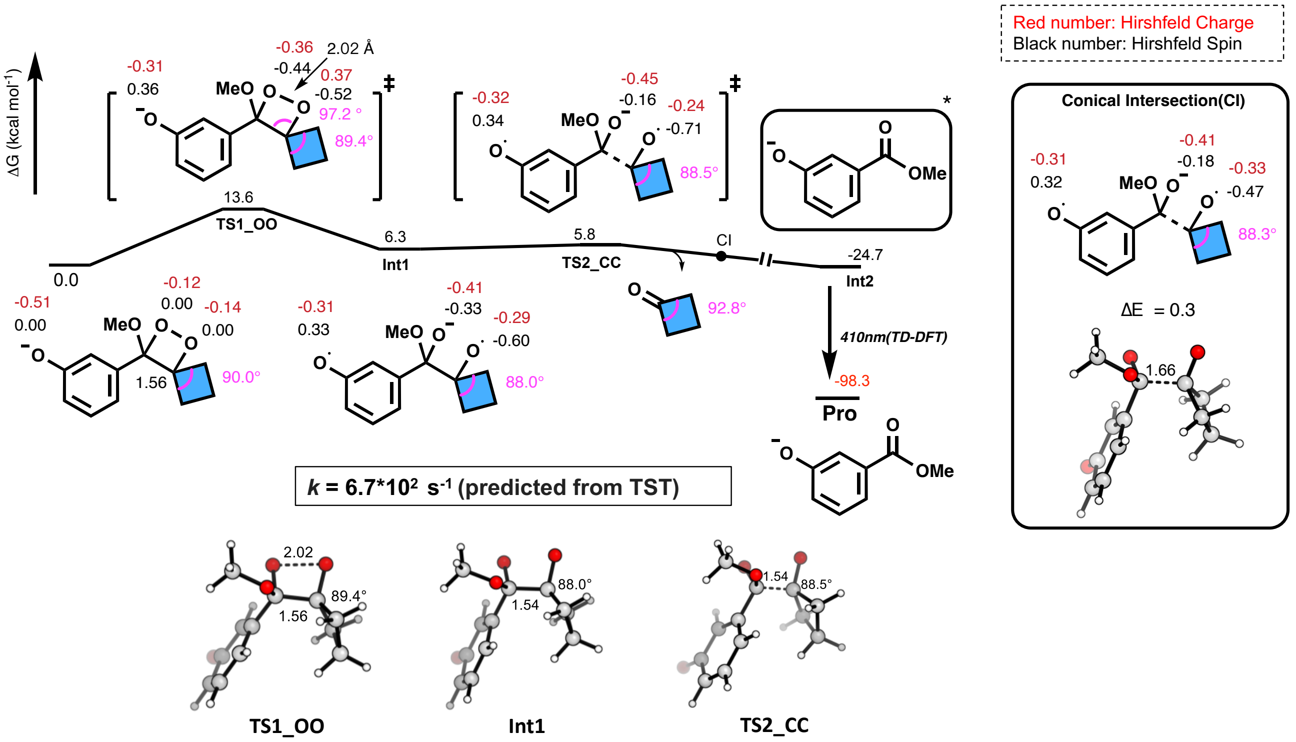

Figure S2: Spiro-**cyclobutyl**-phenoxy-1,2-dioxetane computed Gibbs free energy chemiexcitation profile, essential geometric features along the reaction path, charges, and spin densities on important atoms involved in the reaction. Rate constants were predicted from computed activation free energies using transition state theory (TST).

Computational investigation of additional spiro-substituted dioxetanes and of electron-withdrawing group substituent at the ortho position of a phenoxy-adamantyl-1,2-dioxetane

Several other systems were also investigated computationally. As shown in **Figure S3**, after introducing oxygen into the cyclobutyl ring, the barrier for the oxetanyl dioxetane is further lowered, which can be attributed to the electronegativity of oxygen, which stabilizes the anion intermediate and facilitates the charge transfer process. Conversely, when the phenyl becomes more electron deficient, the charge transfer is inhibited and leads to a higher barrier. As shown in **Figure S5-S6**, EWG (acrylate and acrylonitrile) attached to the phenyl ring results in less charge transfer in TS and higher barriers.

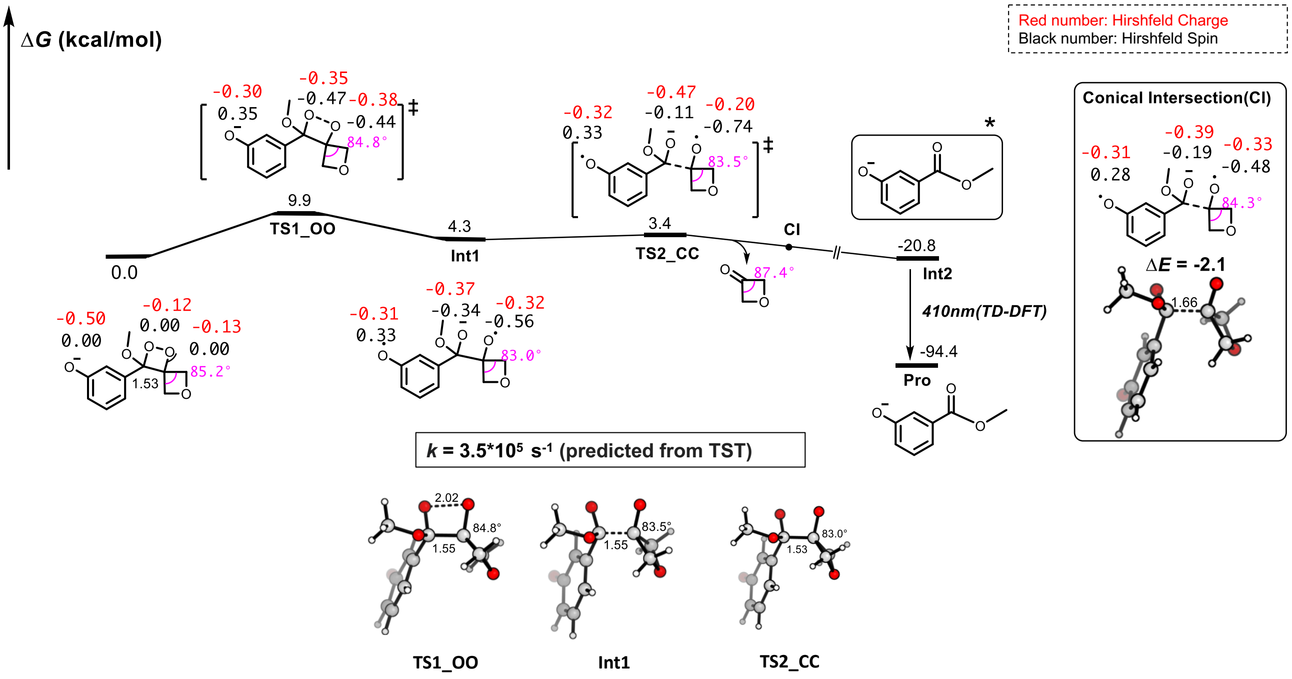

Figure S3: Spiro-**oxetanyl** phenoxy-1,2-dioxetane computed Gibbs free energy chemiexcitation profile, essential geometric features along the reaction path, charges, and spin densities on important atoms involved in the reaction. Rate constants were predicted from computed activation free energies using transition state theory (TST).

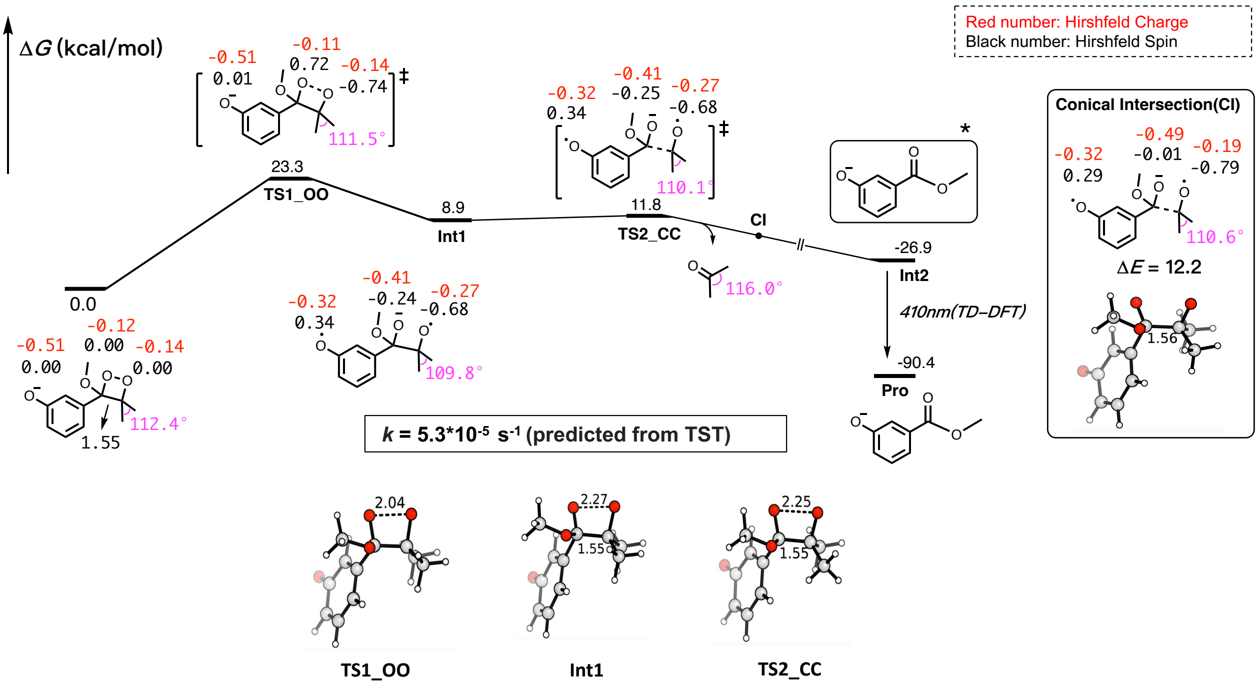

Figure S4: Spiro-**dimethyl**-phenoxy-1,2-dioxetane computed Gibbs free energy chemiexcitation profile, essential geometric features along the reaction path, charges, and spin densities on important atoms involved in the reaction. Rate constants were predicted from computed activation free energies using transition state theory (TST).

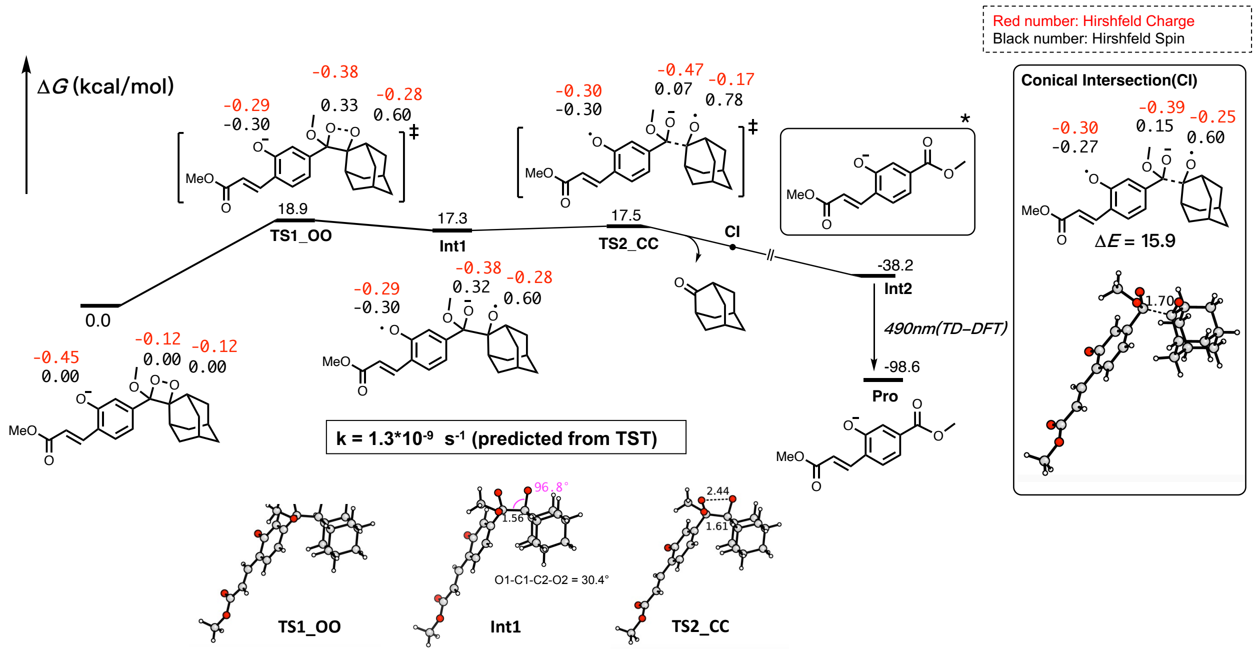

Figure S5: **Ortho-methyl acrylate**-spiro-**adamantyl** phenoxy-1,2-dioxetane computed Gibbs free energy chemiexcitation profile, essential geometric features along the reaction path, charges, and spin densities on important atoms involved in the reaction. Rate constants were predicted from computed activation free energies using transition state theory (TST).

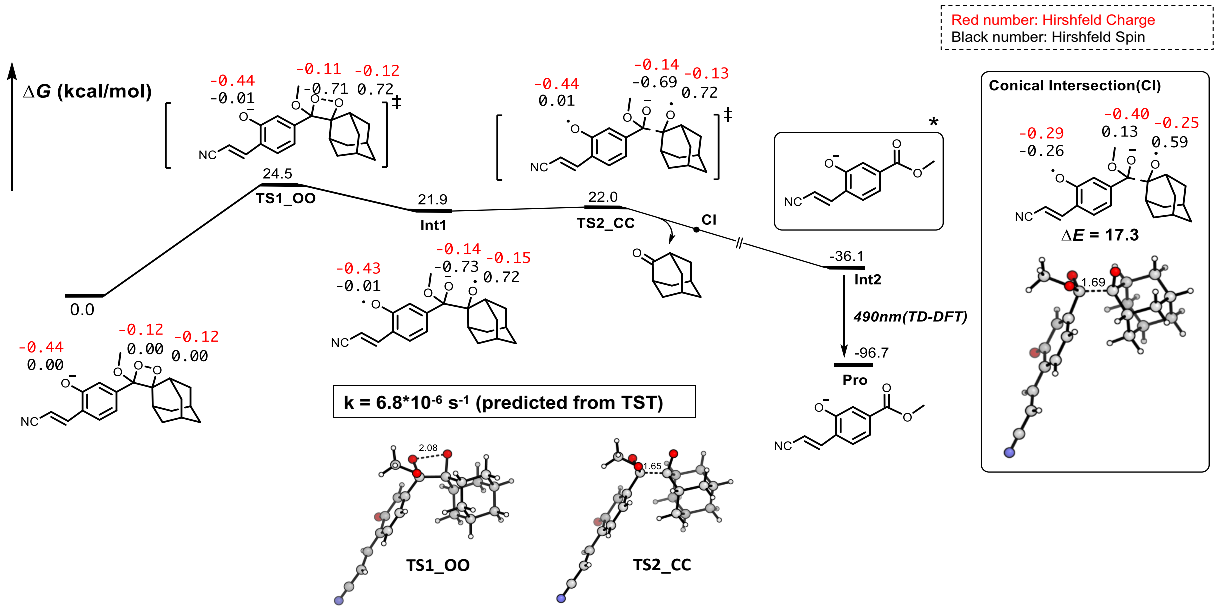

Figure S6: **Ortho-acrylonitrile**-spiro-**adamantyl**-phenoxy-1,2-dioxetane computed Gibbs free energy chemiexcitation profile, essential geometric features along the reaction path, charges, and spin densities on important atoms involved in the reaction. Rate constants were predicted from computed activation free energies using transition state theory (TST).

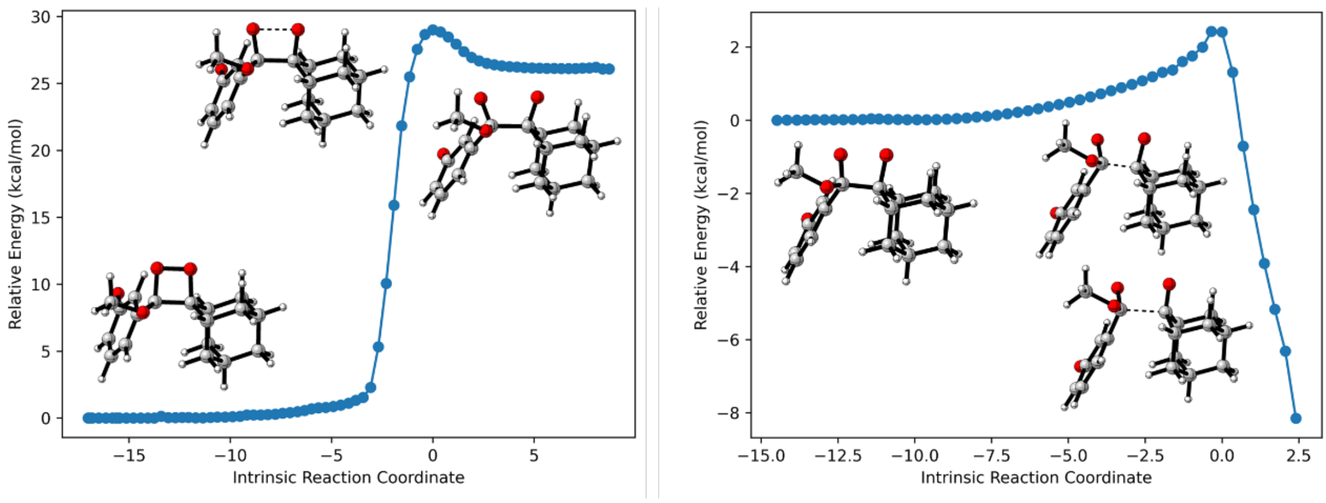

Figure S7: Plot of ωB97XD/6-31G(d)/SMD(DMSO) energies for points along the Intrinsic reaction coordinate (IRC) of **TS1_oo** (left) and **TS2_cc** (right) for Spiro-adamantyl-phenoxy-1,2-dioxetane. Geometries of TSs and end points are also shown in the figure.

Cartesian Coordinates and Energies for Calculated Species

For all minimum structures, no imaginary frequency was observed. Energies are reported in this section directly from the output file at the optimization level of theory (ωB97X-D/6-31G(d)/SMD(DMSO)). E_SP, H and G are energies combining final single point energy with thermal corrections. (CAM-B3LYP-D3(BJ)/ 6-311++G(2d,2p)/SMD(DMSO)). All the energies here are in Hartree.

Adamantanone

E=-464.62483

E_SP=-464.665004

H=-464.428961

G=-464.466738

Imag. Freq. 0

Cartesian coordinates

C -1.505611 -0.000016 0.000755

O -2.722891 -0.000011 0.000428

C -0.665534 1.264866 -0.001846

C -0.665501 -1.264853 0.002723

H -1.331923 2.132369 -0.002659

C 0.226410 1.250113 -1.263001

C 0.228199 1.254789 1.258450

H -1.331928 -2.132317 0.005140

C 0.226570 -1.254899 -1.258317

C 0.228080 -1.250024 1.263170

H 0.843035 2.157671 -1.273689

H -0.397773 1.267679 -2.165303

C 1.116910 -0.002342 -1.257781

H -0.395370 1.276003 2.161066

H 0.844421 2.162650 1.264582

C 1.118384 0.002330 1.256859

H -0.397599 -1.275697 -2.160541

H 0.843168 -2.162515 -1.265656

H -0.395615 -1.267665 2.165768

H 0.844164 -2.157955 1.272830

H 1.746480 -0.003966 -2.154918

C 2.001808 0.000014 -0.001040

H 1.749164 0.003925 2.153139

H 2.652295 -0.884263 0.000074

H 2.652307 0.884295 -0.003096

Cyclobutanone

E=-231.166008

E_SP=-231.202609

H=-231.104682

G=-231.135321

Imag. Freq. 0

Cartesian coordinates

O	1.873208	0.000036	-0.041719
C	0.669135	0.000013	0.016542
C	-0.383238	-1.105065	0.043369
C	-0.383317	1.105047	0.043382
C	-1.468497	-0.000038	-0.068819
H	-0.385005	-1.653591	0.991753
H	-0.313879	-1.823875	-0.779178
H	-0.385091	1.653551	0.991789
H	-0.314083	1.823922	-0.779239
H	-2.208603	0.000005	0.733676
H	-1.983503	-0.000040	-1.031892

Int2

E=-534.567196

E_SP=-534.697537

H=-534.553465

G=-534.596608

Imag. Freq. 0

Cartesian coordinates

C	1.476404	-0.428098	0.000048
O	1.860838	-1.609907	0.000025
O	2.383846	0.617021	0.000018
C	0.114168	0.010621	0.000024
C	-0.259078	1.393791	0.000004
C	-0.946977	-0.937214	0.000014
C	-1.594636	1.801006	-0.000029
H	0.522746	2.146829	0.000008
C	-2.316996	-0.532522	-0.000002
H	-0.727746	-2.000541	0.000021
C	-2.637700	0.875836	-0.000039
H	-1.812109	2.867121	-0.000050
O	-3.254538	-1.392198	-0.000039
C	3.741837	0.227462	-0.000005
H	3.996296	-0.361514	-0.888460
H	4.323332	1.152778	-0.000035
H	3.996335	-0.361482	0.888459
H	-3.682157	1.172185	-0.000071

adam_Cl

E_SP=-999.281788

Imag. Freq. 0

Cartesian coordinates

C	-2.226518	0.533185	-0.206694
C	-1.013530	1.542029	0.421448
O	-3.111808	1.368570	-0.780690
O	-1.175662	1.748496	1.666793
O	-1.156893	2.736347	-0.421493
C	-2.930864	-0.194108	0.970781
C	-1.834085	-0.565490	-1.246737
H	-3.128289	0.568037	1.723870
C	-2.050242	-1.293619	1.563863

C -4.229564 -0.819218 0.448994
H -1.298574 -0.095508 -2.075776
C -0.994167 -1.700081 -0.647802
C -3.136995 -1.187888 -1.777662
H -2.569702 -1.750682 2.415533
H -1.127022 -0.854848 1.946811
C -1.746813 -2.358627 0.507916
H -4.882797 -0.039882 0.047412
H -4.765866 -1.302479 1.274789
C -3.908628 -1.855385 -0.632857
H -0.025231 -1.344769 -0.300343
H -0.796146 -2.439445 -1.433239
H -3.751794 -0.417026 -2.248601
H -2.898111 -1.932339 -2.546335
H -1.124985 -3.147294 0.946397
C -3.051018 -2.963171 -0.016291
H -4.839081 -2.283918 -1.022626
H -2.831795 -3.734361 -0.763913
H -3.598117 -3.451024 0.799077
C 0.415774 1.044238 0.065154
C 0.924709 1.108131 -1.248421
C 1.255303 0.649660 1.065225
C 2.236877 0.708968 -1.552213
H 0.288849 1.492352 -2.035020
C 2.622666 0.227284 0.817384
H 0.891209 0.655067 2.085648
C 3.070754 0.269477 -0.565576
H 2.582416 0.761707 -2.579635
O 3.368789 -0.140802 1.723783
C -0.615089 3.885182 0.130955
H 0.487467 3.870100 0.159944
H -0.917330 4.734610 -0.489987
H -0.966164 4.043388 1.155308
H 4.089014 -0.044256 -0.769381

adam_Int1

E=-999.150849

E_SP=-999.312939

H=-998.931582

G=-998.990038

Imag. Freq. 0

Cartesian coordinates

C 0.954196 0.743215 -0.614459
C -0.522777 1.304783 -0.370759
O 1.508186 1.593153 -1.511617
O -1.016749 1.688556 -1.562085
O -0.360851 2.370700 0.548740
C 1.064303 -0.739747 -1.119449
C 1.918896 0.842909 0.651852
H 0.379402 -0.854136 -1.966467
C 0.726753 -1.774885 -0.030226
C 2.514627 -0.988998 -1.575914
H 1.818238 1.854493 1.044053
C 1.517550 -0.189068 1.713015
C 3.364723 0.573585 0.203750
H 0.843069 -2.774740 -0.469658

H	-0.309633	-1.689987	0.297593
C	1.671174	-1.614840	1.165367
H	2.791010	-0.296568	-2.375694
H	2.582295	-2.006499	-1.982260
C	3.479538	-0.840557	-0.385469
H	0.489319	-0.022474	2.047965
H	2.172303	-0.042531	2.582565
H	3.692614	1.323659	-0.521334
H	4.016253	0.660397	1.083589
H	1.400446	-2.336046	1.945547
C	3.116864	-1.857112	0.707974
H	4.507417	-1.013109	-0.726335
H	3.807500	-1.756930	1.556086
H	3.218294	-2.880658	0.323260
C	-1.641575	0.338588	0.118562
C	-2.045356	0.371326	1.456469
C	-2.262280	-0.503904	-0.799042
C	-3.057049	-0.513124	1.855718
H	-1.568256	1.040108	2.162887
C	-3.304211	-1.435957	-0.425346
H	-1.955900	-0.498062	-1.839502
C	-3.665320	-1.386119	0.970657
H	-3.361562	-0.520215	2.902040
O	-3.850552	-2.207499	-1.260106
C	-1.340738	3.384277	0.478487
H	-2.360160	2.978307	0.511431
H	-1.187267	4.019139	1.355149
H	-1.233434	3.991196	-0.427691
H	-4.446171	-2.062619	1.314659

adam_TS1

E=-999.146184

E_SP=-999.31384

H=-998.933042

G=-998.990679

Imag. Freq. -340.27

Cartesian coordinates

C	0.940532	0.668808	-0.523295
C	-0.495126	1.280733	-0.305308
O	1.311611	1.533301	-1.584065
O	-0.745768	1.732787	-1.589302
O	-0.358012	2.328531	0.621565
C	0.997797	-0.806313	-0.979344
C	1.954626	0.874019	0.626258
H	0.260541	-0.962025	-1.773370
C	0.736997	-1.780869	0.184404
C	2.413650	-1.073237	-1.527404
H	1.880219	1.907971	0.965258
C	1.653971	-0.094872	1.780417
C	3.367943	0.593565	0.080053
H	0.816710	-2.808295	-0.194982
H	-0.275219	-1.661800	0.579352
C	1.766739	-1.549425	1.298467
H	2.619513	-0.409591	-2.374830
H	2.465727	-2.104299	-1.899951
C	3.458826	-0.855916	-0.420493

H 0.651842 0.091636 2.182490
H 2.370896 0.093252 2.590596
H 3.609284 1.293116 -0.730436
H 4.098561 0.764241 0.881433
H 1.561754 -2.228314 2.134858
C 3.178997 -1.809234 0.751918
H 4.462083 -1.044311 -0.821099
H 3.925314 -1.658583 1.543624
H 3.263731 -2.851920 0.417798
C -1.658134 0.356306 0.066560
C -2.055697 0.264672 1.407481
C -2.296773 -0.383302 -0.922138
C -3.088383 -0.624082 1.725272
H -1.565414 0.857961 2.170820
C -3.371458 -1.308699 -0.638388
H -1.984030 -0.293382 -1.957783
C -3.723721 -1.387002 0.758330
H -3.397402 -0.720604 2.765826
O -3.943499 -1.981515 -1.539726
C -1.363398 3.321779 0.558910
H -2.371281 2.892010 0.619949
H -1.205016 3.972154 1.422424
H -1.283681 3.916237 -0.357837
H -4.523121 -2.071879 1.037887

adam_TS2

E=-999.158616

E_SP=-999.323512

H=-998.944623

G=-999.002908

Imag. Freq. -717.44

Cartesian coordinates

C 0.999934 0.816961 -0.369991
C -0.518139 1.351619 -0.393071
O 1.600880 1.918123 -0.961966
O -0.755198 1.831678 -1.583257
O -0.498886 2.351872 0.704546
C 1.245293 -0.448520 -1.238872
C 1.717372 0.594993 0.994719
H 0.687648 -0.321748 -2.173939
C 0.834604 -1.759159 -0.540472
C 2.757321 -0.524646 -1.530322
H 1.495043 1.451065 1.636334
C 1.297110 -0.716409 1.678969
C 3.234338 0.516311 0.716068
H 1.055314 -2.605063 -1.207392
H -0.240035 -1.786461 -0.341550
C 1.607247 -1.919595 0.776202
H 3.075465 0.382233 -2.055222
H 2.970908 -1.379879 -2.186739
C 3.539920 -0.673261 -0.212264
H 0.232360 -0.709334 1.928596
H 1.844994 -0.809571 2.627682
H 3.580410 1.451980 0.260764
H 3.778005 0.394806 1.663766
H 1.293597 -2.844109 1.277500

C 3.113933 -1.973246 0.486220
H 4.616136 -0.703890 -0.425646
H 3.674882 -2.100787 1.422533
H 3.343742 -2.838155 -0.151312
C -1.616102 0.332404 0.037093
C -1.988792 0.109726 1.378910
C -2.314810 -0.323981 -0.949365
C -3.005571 -0.803933 1.725104
H -1.489069 0.677612 2.156614
C -3.357583 -1.282631 -0.650182
H -2.077471 -0.116621 -1.988166
C -3.673425 -1.501587 0.749837
H -3.257446 -0.947901 2.772404
O -3.969812 -1.895652 -1.557875
C -1.505863 3.309774 0.566700
H -2.520612 2.883791 0.673056
H -1.366849 4.047422 1.365938
H -1.457627 3.813665 -0.406419
H -4.455986 -2.216135 0.987899

cyclobut_Int1

E=-765.707673

E_SP=-765.873487

H=-765.62953

G=-765.682453

Imag. Freq. 0

Cartesian coordinates

C 0.893069 0.662461 -0.296299
O 2.884380 0.054615 -1.122779
O 1.004716 1.218936 -1.508215
O 1.368648 1.504004 0.804774
C -0.563135 0.298514 0.060024
C -0.952611 0.038364 1.393586
C -1.509774 0.212206 -0.931504
C -2.273721 -0.323538 1.718285
H -0.212184 0.135749 2.181221
C -2.884012 -0.151980 -0.649783
H -1.225938 0.424393 -1.957614
C -3.227374 -0.423454 0.734271
H -2.533530 -0.517942 2.755207
O -3.743558 -0.230855 -1.559181
C 0.880703 2.813924 0.734396
H -0.206153 2.873229 0.923490
H 1.390174 3.396982 1.509640
H 1.077154 3.263570 -0.246668
H -4.254006 -0.699040 0.956795
C 1.886556 -0.509799 -0.358483
C 2.341902 -1.209279 0.954322
C 1.300543 -1.878589 -0.821864
C 1.389856 -2.383793 0.634563
H 2.193864 -0.651299 1.883398
H 3.394029 -1.508238 0.878947
H 0.322165 -1.892616 -1.315036
H 2.033699 -2.376381 -1.466357
H 0.435142 -2.297317 1.163349
H 1.780076 -3.394914 0.787763

cyclobut_TS1

E=-765.687845

E_SP=-765.863588

H=-765.620248

G=-765.672002

Imag. Freq. -498.16

Cartesian coordinates

C	0.853807	0.595992	-0.322163
O	2.609264	-0.224186	-1.393465
O	0.949282	0.891367	-1.681378
O	1.447211	1.581889	0.479832
C	-0.599738	0.339445	0.055453
C	-0.983340	0.496474	1.394847
C	-1.509480	-0.073919	-0.910046
C	-2.312746	0.217031	1.731093
H	-0.267026	0.825640	2.139916
C	-2.893926	-0.362262	-0.608452
H	-1.186925	-0.201829	-1.939557
C	-3.236338	-0.194334	0.783599
H	-2.627624	0.324783	2.768753
O	-3.720820	-0.729209	-1.488452
C	0.952297	2.892107	0.272919
H	-0.126925	2.955992	0.460514
H	1.474618	3.533836	0.986068
H	1.161987	3.242834	-0.744039
H	-4.264482	-0.403828	1.075911
C	1.822573	-0.622968	-0.319284
C	2.544628	-0.952249	1.008779
C	1.222495	-2.042537	-0.327327
C	1.581875	-2.151113	1.172063
H	2.546254	-0.165720	1.765063
H	3.571696	-1.275387	0.811637
H	0.177054	-2.158084	-0.622219
H	1.852828	-2.692788	-0.942332
H	0.731902	-1.901985	1.812862
H	2.024493	-3.092361	1.506224

cyclobut_TS2

E=-765.707263

E_SP=-765.87395

H=-765.631537

G=-765.683161

Imag. Freq. -324.1

Cartesian coordinates

C	-0.827122	-0.700000	-0.218548
O	-2.778677	0.022194	-1.134399
O	-0.954387	-1.439009	-1.308715
O	-1.291631	-1.342994	1.030179
C	0.638062	-0.380368	0.148101
C	1.021231	0.090550	1.424708
C	1.607188	-0.547812	-0.809867
C	2.362915	0.398702	1.722542
H	0.264468	0.207257	2.193057

C 3.001350 -0.248426 -0.553628
H 1.319104 -0.922234 -1.787560
C 3.340451 0.241681 0.770033
H 2.618406 0.759193 2.715273
O 3.881420 -0.403190 -1.433509
C -2.479195 -2.075521 0.912941
H -2.529843 -2.576054 -0.060415
H -2.482817 -2.830148 1.711156
H -3.377399 -1.449325 1.025187
H 4.382067 0.472249 0.973688
C -1.699519 0.565481 -0.476204
C -2.074844 1.491132 0.717089
C -0.994264 1.782033 -1.145028
C -1.040601 2.522346 0.210230
H -1.942594 1.057590 1.712832
H -3.102273 1.859808 0.618871
H -0.014421 1.623180 -1.608204
H -1.668860 2.235778 -1.879659
H -0.092235 2.459583 0.752658
H -1.361319 3.568327 0.190449

cyclobut_CI

E_SP=-765.887654

Imag. Freq. 0

Cartesian coordinates

C 0.945459 0.645055 -0.437848
O 3.146742 -0.378012 -0.377811
O 1.034235 0.955490 -1.666968
O 1.474055 1.611816 0.507490
C -0.517012 0.371411 0.016926
C -0.953990 0.528400 1.348407
C -1.448171 0.043190 -0.938533
C -2.293465 0.304128 1.714857
H -0.241687 0.849993 2.097164
C -2.830978 -0.201133 -0.611831
H -1.142071 -0.014378 -1.975993
C -3.220098 -0.062833 0.773565
H -2.589019 0.430379 2.751544
O -3.661533 -0.506315 -1.486453
C 1.294482 2.934605 0.130182
H 0.236345 3.243423 0.156148
H 1.843183 3.565594 0.835987
H 1.663408 3.118308 -0.883298
H -4.259206 -0.244004 1.027279
C 1.906529 -0.673147 -0.110834
C 1.692025 -1.374431 1.278336
C 1.339087 -1.959350 -0.794360
C 0.908135 -2.496817 0.579610
H 1.176774 -0.798828 2.046631
H 2.657494 -1.705514 1.666199
H 0.559210 -1.792616 -1.538748
H 2.155941 -2.528374 -1.243155
H -0.169362 -2.427685 0.744052
H 1.228981 -3.514604 0.815266

hydroxybenzoate

E=-534.684923

E_SP=-534.802009

H=-534.655292

G=-534.697792

Imag. Freq. 0

Cartesian coordinates

C -1.480500 -0.424469 0.000032

O -1.815389 -1.593454 0.000101

O -2.377678 0.575851 -0.000056

C -0.071484 0.063296 0.000015

C 0.224514 1.436348 0.000065

C 0.939542 -0.892884 -0.000030

C 1.570928 1.809056 0.000023

H -0.564028 2.179525 0.000148

C 2.341272 -0.553769 -0.000025

H 0.671346 -1.946467 -0.000054

C 2.590419 0.868228 -0.000026

H 1.826324 2.868236 0.000049

O 3.259952 -1.420225 -0.000026

C -3.748815 0.180114 -0.000062

H -3.985300 -0.406825 0.891571

H -4.323757 1.106702 -0.000096

H -3.985267 -0.406864 -0.891677

H 3.630349 1.192802 -0.000045

spiro-adamantyl-dioxetane

E=-999.19237

E_SP=-999.351862

H=-998.967335

G=-999.025144

Imag. Freq. 0

Cartesian coordinates

C 0.906046 0.579380 -0.429055

C -0.490110 1.215889 -0.186185

O 0.990036 1.402372 -1.636417

O -0.419467 1.749516 -1.524970

O -0.370109 2.226046 0.766382

C 0.956049 -0.908869 -0.797494

C 2.030121 0.912771 0.550110

H 0.140556 -1.132211 -1.492054

C 0.823219 -1.774285 0.468193

C 2.310809 -1.201554 -1.467583

H 1.968935 1.971534 0.808883

C 1.873249 0.047716 1.813058

C 3.381476 0.608495 -0.121092

H 0.865269 -2.832656 0.180161

H -0.146115 -1.605602 0.949025

C 1.960448 -1.441376 1.444543

H 2.413645 -0.607533 -2.383428

H 2.344115 -2.258740 -1.760207

C 3.456690 -0.879043 -0.495600

H 0.912191 0.261390 2.296927

H 2.663846 0.310943 2.527564

H 3.507954 1.232062 -1.014917

H 4.191610 0.869729 0.571897

H 1.856779 -2.050070 2.350616

C 3.313561 -1.731801 0.775288

H 4.418692 -1.089936 -0.977654

H 4.133606 -1.507483 1.470643

H 3.383411 -2.798123 0.522158

C -1.720270 0.358224 0.025661

C -2.148343 0.111464 1.341122

C -2.376195 -0.215054 -1.054198

C -3.242537 -0.737490 1.529114

H -1.639067 0.570535 2.182338

C -3.515155 -1.093694 -0.909063

H -2.032228 -0.008239 -2.064076

C -3.904397 -1.323830 0.460512

H -3.584066 -0.942711 2.543550

O -4.107317 -1.611928 -1.896957

C -1.396113 3.204607 0.739471

H -2.383156 2.768733 0.931903

H -1.159263 3.917616 1.531942

H -1.413795 3.730483 -0.221829

H -4.754276 -1.982066 0.637118

spiro-cyclobutyl-dioxetane

E=-765.73021

E_SP=-765.888232

H=-765.640955

G=-765.692525

Imag. Freq. 0

Cartesian coordinates

C 0.797257 0.621527 -0.195543

O 2.108915 0.050386 -1.703404

O 1.000779 0.993027 -1.582066

O 1.357010 1.557834 0.664397

C -0.641149 0.288814 0.127023

C -0.989208 0.131583 1.478771

C -1.567284 0.068634 -0.881413

C -2.299255 -0.254992 1.777764

H -0.259947 0.314482 2.261954

C -2.935778 -0.317520 -0.618843

H -1.270268 0.182427 -1.920446

C -3.241383 -0.470623 0.783513

H -2.587284 -0.385434 2.820752

O -3.780102 -0.507248 -1.538392

C 0.721243 2.826758 0.662491

H -0.311639 2.764846 1.023391

H 1.300228 3.460415 1.337441

H 0.729764 3.271589 -0.339403

H -4.255440 -0.769780 1.046118

C 1.798921 -0.522123 -0.419704

C 2.904797 -0.807739 0.596232

C 1.348981 -1.984509 -0.329992

C 2.100820 -2.061538 1.019825

H 3.111644 -0.031799 1.335377

H 3.829833 -1.082985 0.079080

H	0.275470	-2.188263	-0.354092
H	1.853522	-2.574244	-1.101842
H	1.436093	-1.850229	1.862317
H	2.667430	-2.972652	1.22432

Appendix II- Supplementary Figures

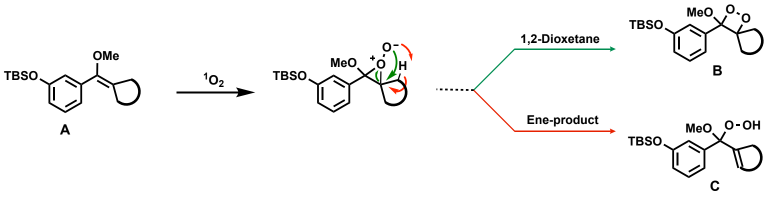

HPLC comperison between oxidation of different cycloalkene enol ethers

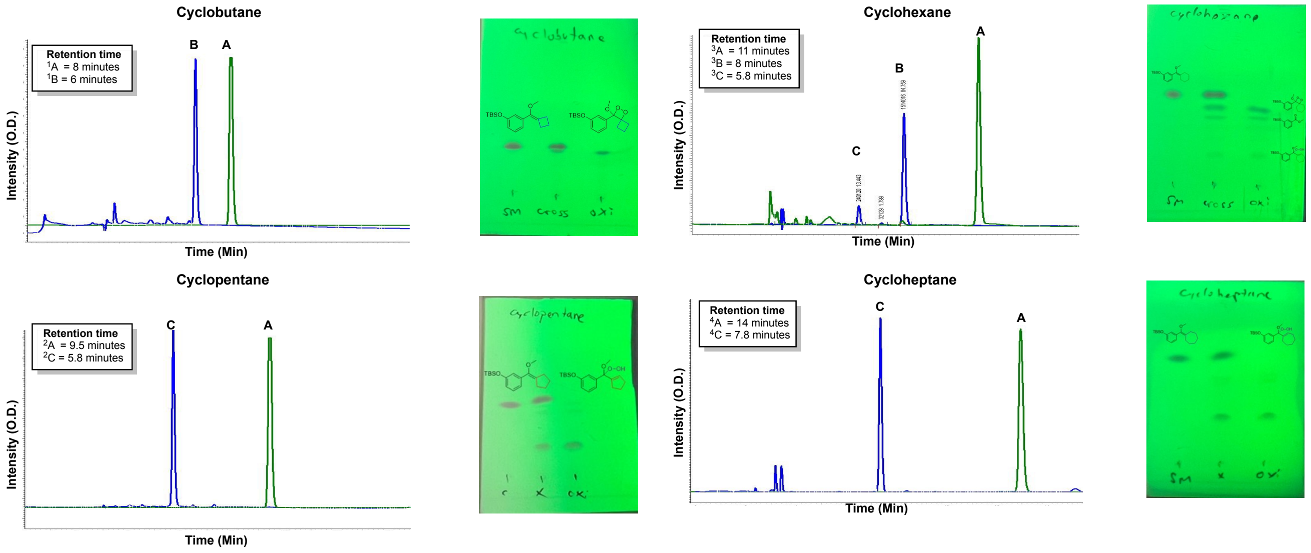

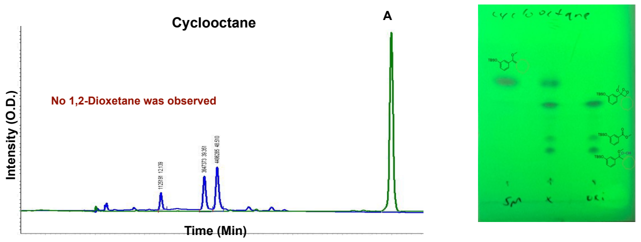

#	^{n}B (Dioxetane) HPLC%	^{n}C (Ene-product) HPLC%	D (Benzoate) HPLC%
Cyclobutane	100%	0%	0%
Cyclopentane	0%	100%	0%
Cyclohexane	87%	13%	0%
Cycloheptane	0%	100%	0%
Cyclooctane	ND	ND	ND

Figure **S8**: (Top) Oxidation of enolethers by singlet oxygen: the ene-product vs the 1,2-dioxetane product. (Bottom) Oxidation of five synthesized cycloalkyl-enolether dioxetane precursors; ratio of product distribution was determined by RP-HPLC. Reaction monitoring was conducted by RP-HPLC (90-100% ACN in water with 0.1% TFA) and TLC (90:10, Hex:EtOAc).

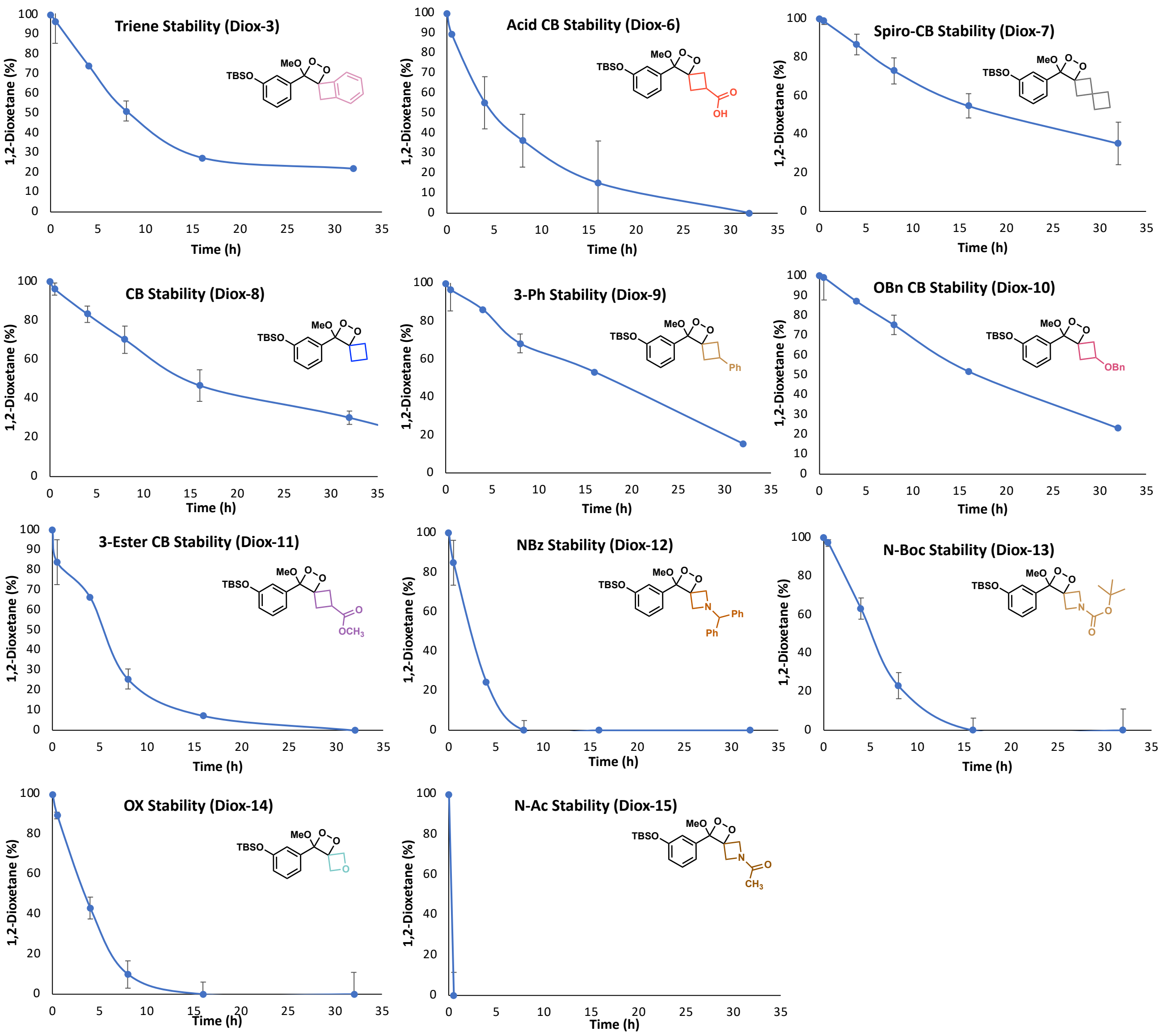

Figure **S9**: Stability of **Diox 3** and **Diox 6** -**Diox 15** [500 μM] measured in PBS [100 mM], pH 7.4, 10% ACN at room temperature; the ratio of product distribution was determined using RP-HPLC (90-100% ACN in water with 0.1% TFA). The detailed assay procedure is mentioned in the experimental protocols section.

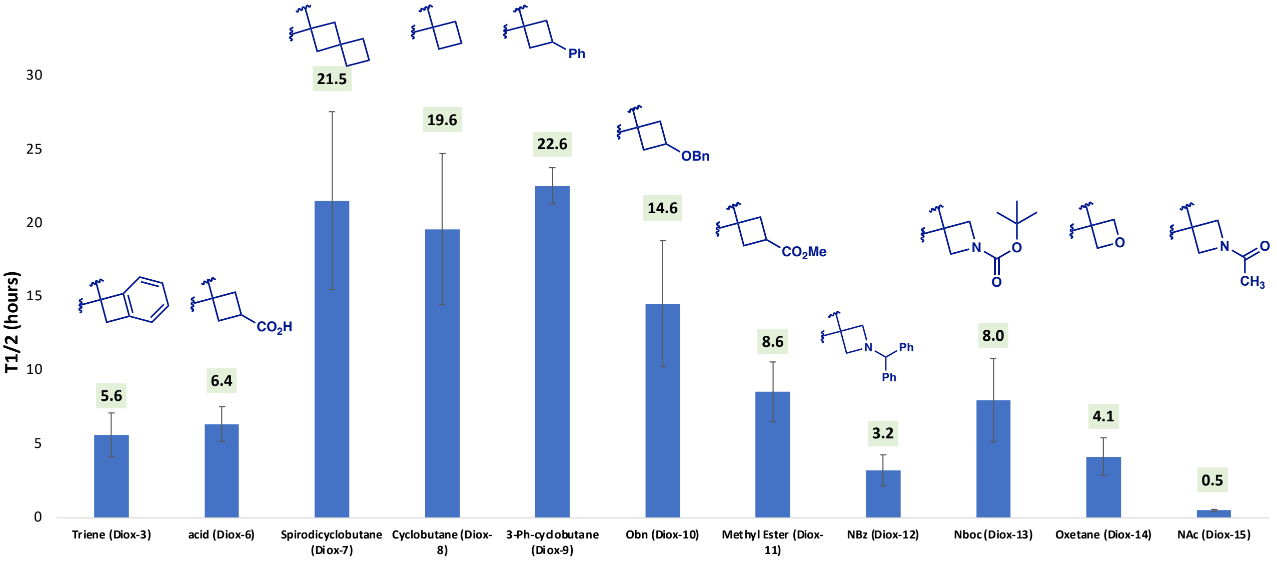

Figure **S10**: **Diox 3** and **Diox 6** -**Diox 15** stability results summary. **Diox 1, 2, 4, 5, and 8** did not decompose at room temperature for over 400 hours and therefore were further evaluated at 50°C.

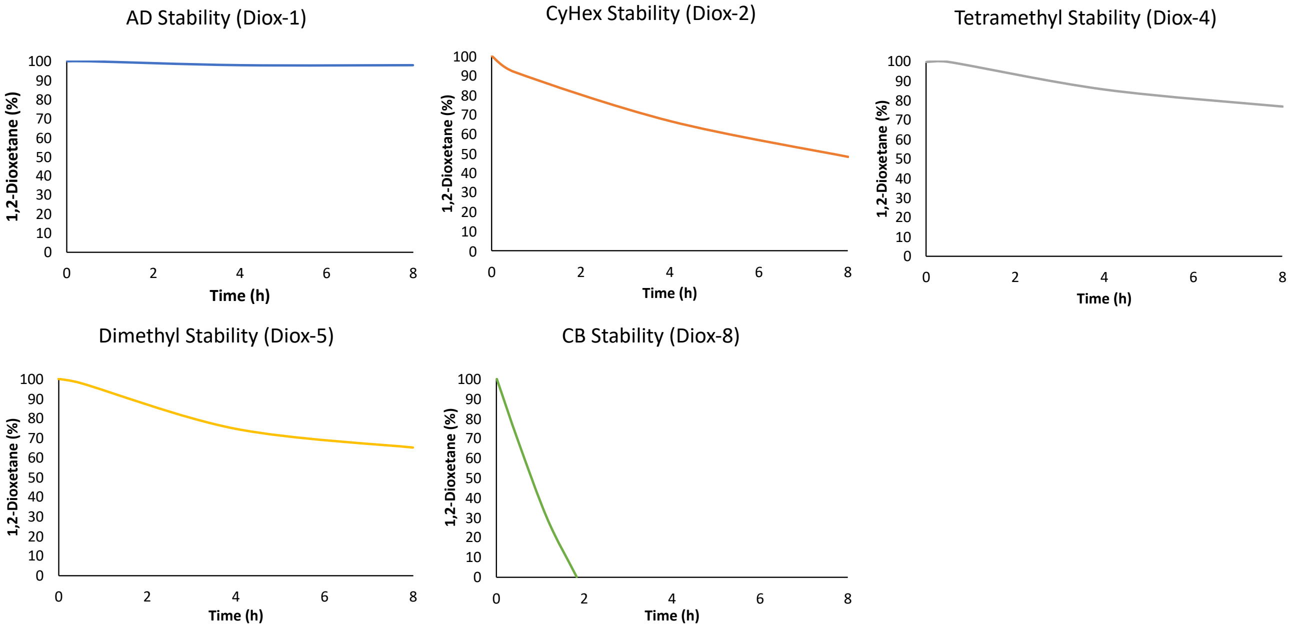

Figure **S11**: Stability of **Diox 1, 2, 4, 5, and 8** [500 µM] measured in PBS [100 mM], pH 7.4, 10% ACN at 50°C; the ratio of product distribution was determined using RP-HPLC (90-100% ACN in water with 0.1% TFA). The detailed assay procedure is mentioned in the experimental protocols section.

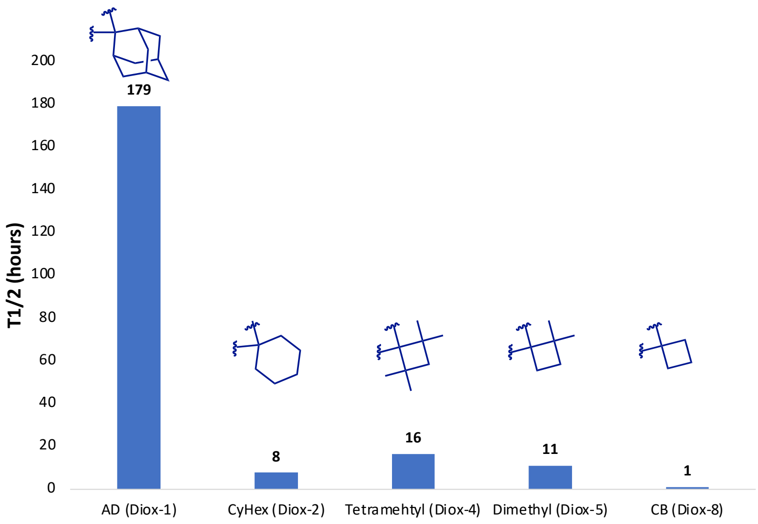

Figure **S12**: Stability result summary of **Diox 1, 2, 4, 5, and 8** at 50°C.

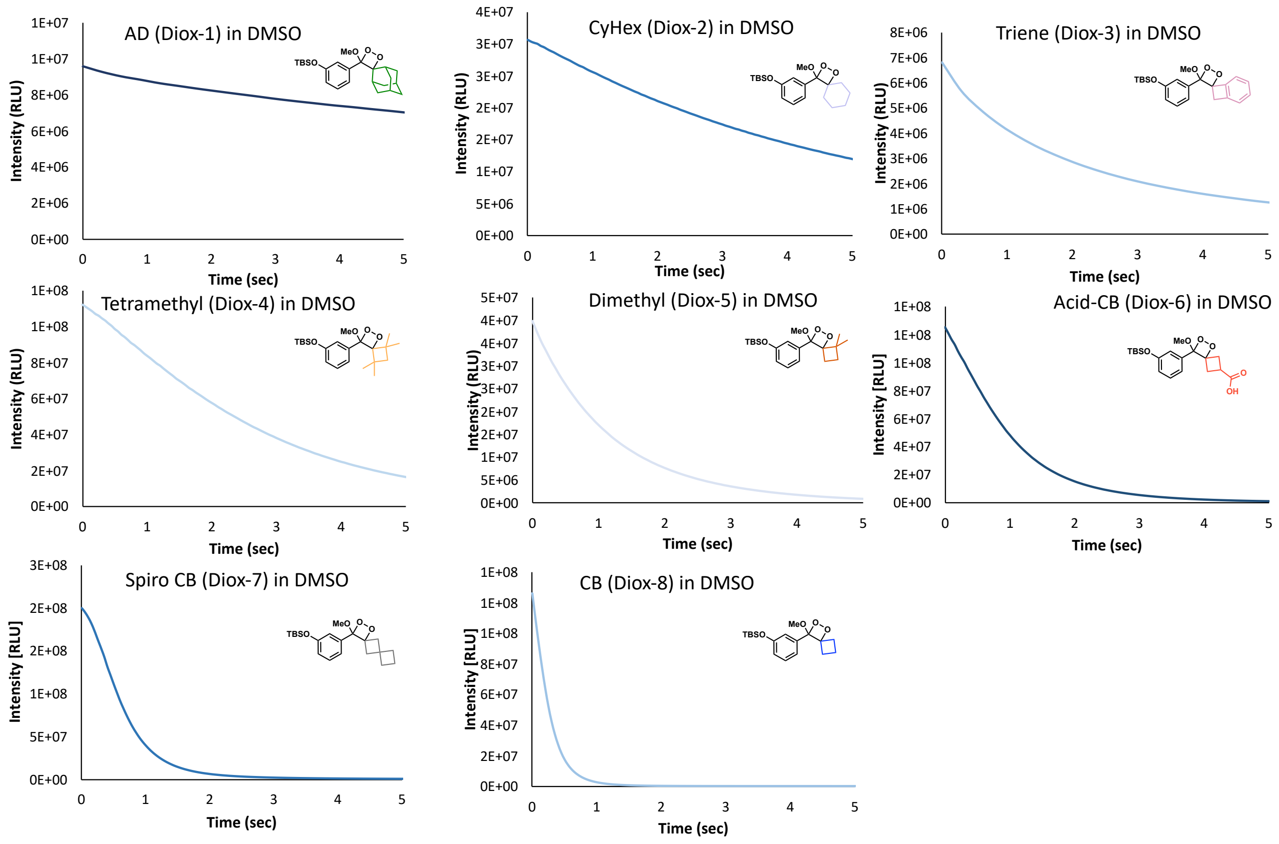

Figure **S13**: Chemiluminescent kinetic profiles during the first 5 seconds of **Diox 1-Diox 8** [10 nM], measured in DMSO, with TBAF [10 mM]. The detailed assay procedure is mentioned in the experimental protocols section.

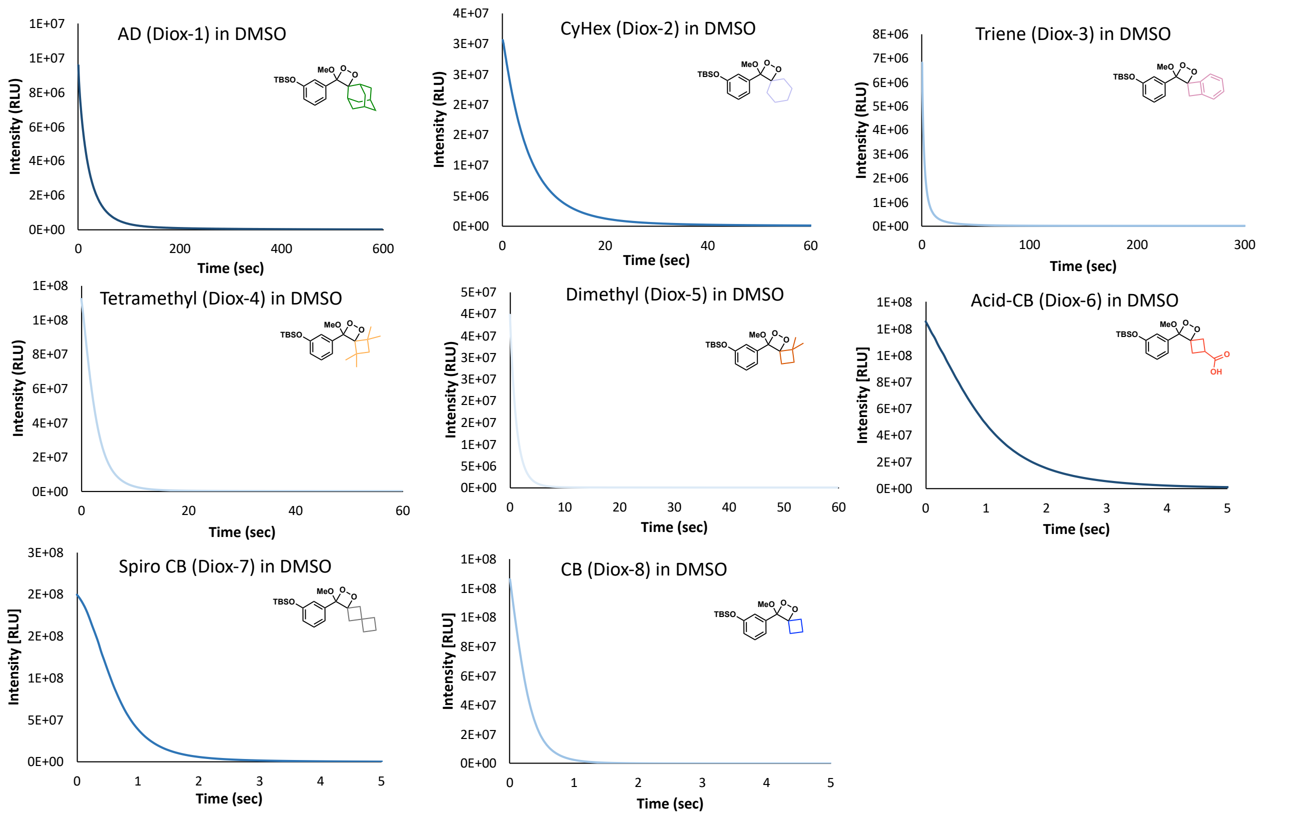

Figure **S14**: Full chemiluminescent kinetic profiles of **Diox 1-Diox 8** [10 nM] measured in DMSO, with TBAF [10 mM]. The detailed assay procedure is mentioned in the experimental protocols section.

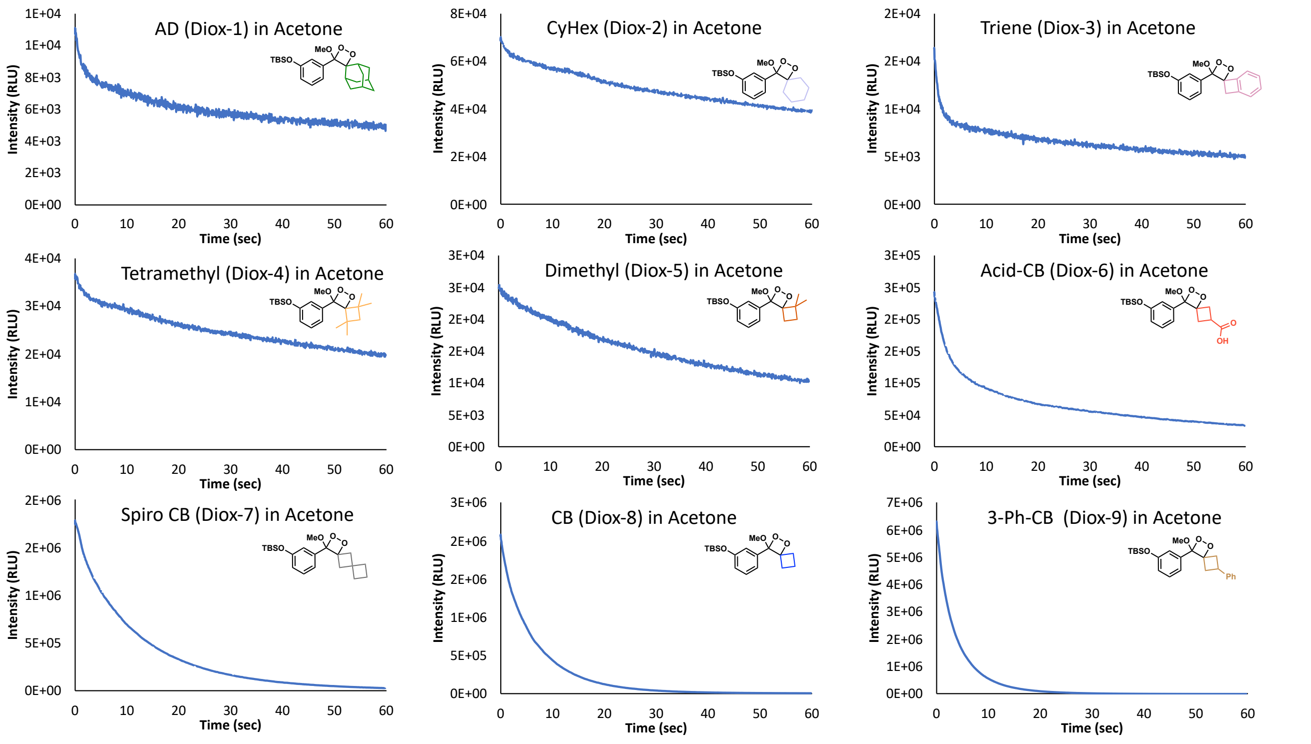

Figure **S15**: Chemiluminescent kinetic profiles of **Diox 1-Diox 9** [10 nM] measured in Acetone, with TBAF [10 mM]. The detailed assay procedure is mentioned in the experimental protocols section.

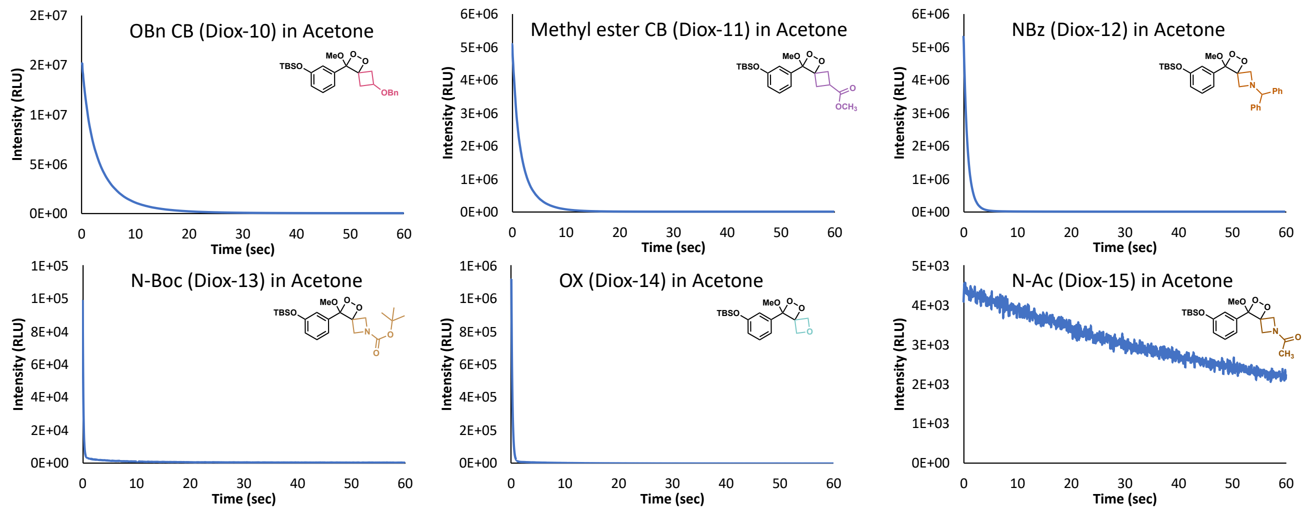

Figure **S16**: Chemiluminescent kinetic profiles of **Diox 10-Diox 15** [10 nM] measured in Acetone, with TBAF [10 mM]. The detailed assay procedure is mentioned in the experimental protocols section.

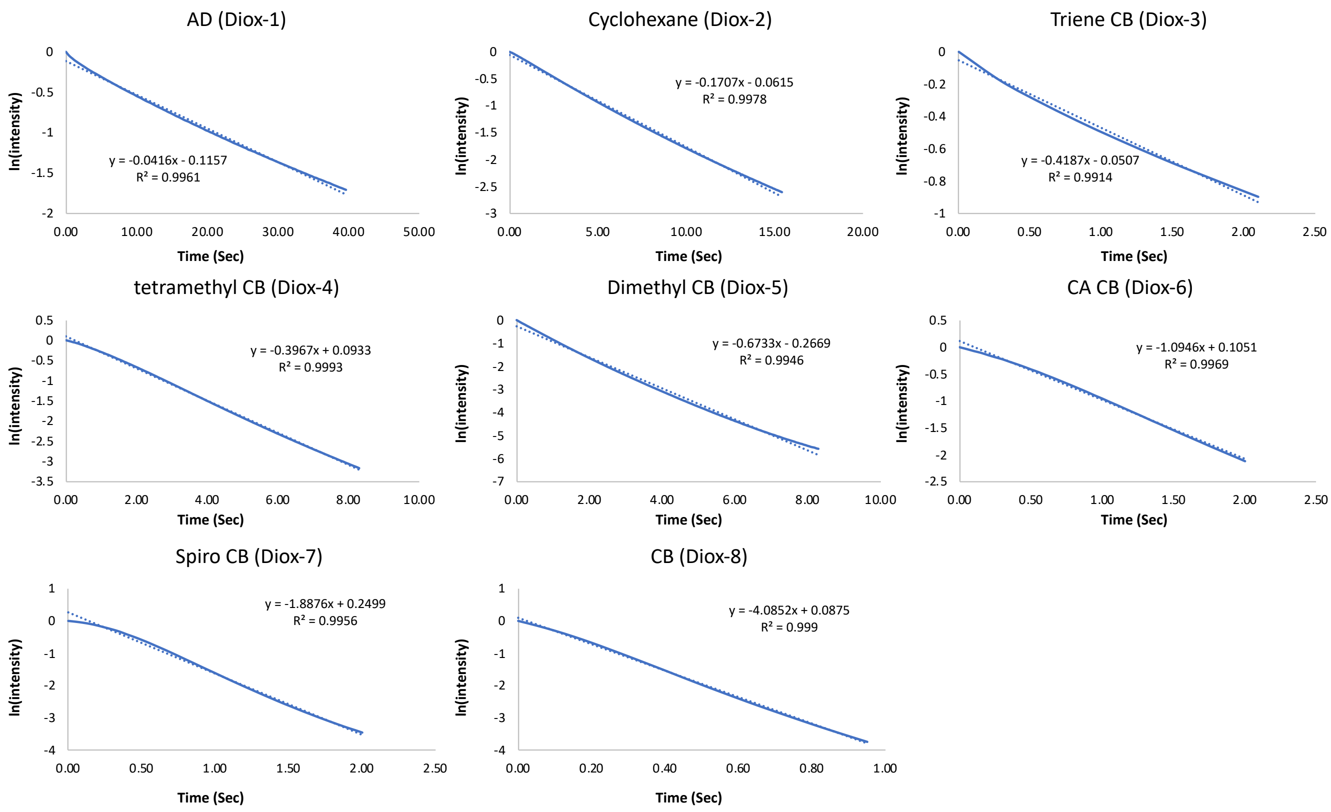

Figure **S17**: Rate constant calculations. 1/intensity kinetic profiles of **Diox 1-Diox 8** [10 nM] measured in DMSO, with TBAF [10 mM].

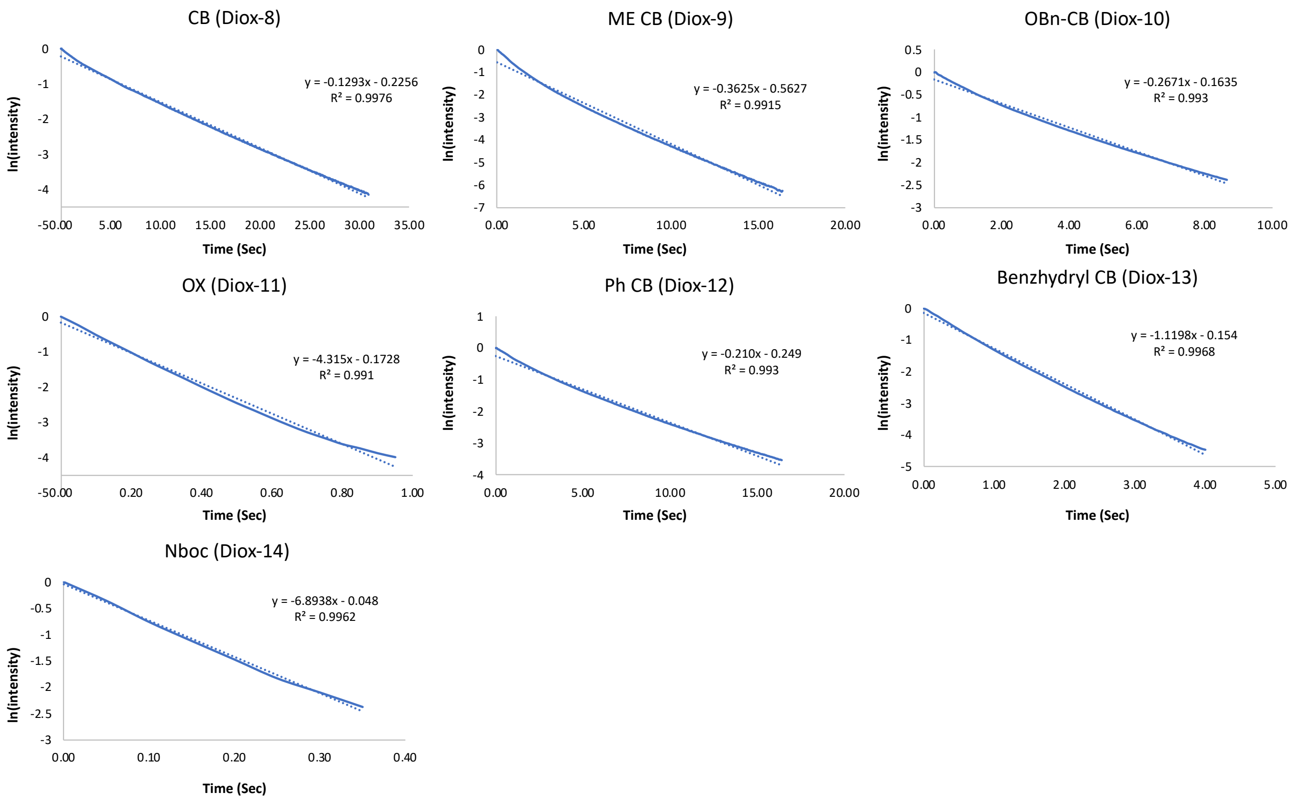

Figure **S18**: Rate constant calculations. 1/intensity kinetic profiles of **Diox 8-Diox 14** [10 nM] measured in Acetone, with TBAF [10 mM].

Chemiluminescent Properties of Selected 1,2-Dioxetanes							
**Entry**	**Molecular Structure**	**$T_{1/2}$ (sec)** Total Light Emission **DMSO**	**Acetone**	**Relative Chemiexcitation Rate ($T_{1/2}$)**	**Rate constant (1/sec) DMSO**	**Acetone**	**Relative Chemiexcitation Rate (Rate constant)**
**Diox 1**	***Adamantyl**-dioxetane* 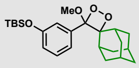	21.3	ND	**1**	0.041	ND	**1**
**Diox 2**	***Cyclohexyl**-dioxetane* 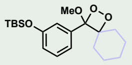	4.3	ND	**4.95**	0.17	ND	**4**
**Diox 3**	***Cyclobutyl**-dioxetanes* 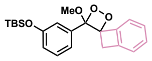	3.9	ND	**5.5**	0.418	ND	**10**
**Diox 4**	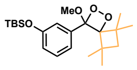	1.9	ND	**11.5**	0.396	ND	**10**
**Diox 5**	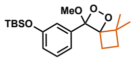	1.0	ND	**22.4**	0.673	ND	**16**
**Diox 6**	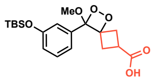	0.7	ND	**30.4**	1.094	ND	**27**
**Diox 7**	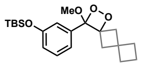	8.9	ND	**59.8**	1.887	ND	**46**
**Diox 8**	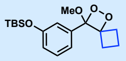	0.2	5	**106.5**	4.08	0.129	**100**
**Diox 9**	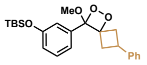	ND	3.0	**177.5**	ND	0.21	**162**
**Diox 10**	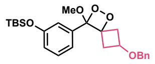	ND	2.7	**197.2**	ND	0.267	**206**
**Diox 11**	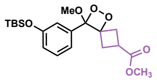	ND	1.4	**380.3**	ND	0.362	**279**
**Diox 12**	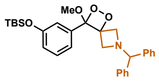	ND	0.55	**968.2**	ND	1.119	**863**
**Diox 13**	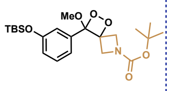	ND	<0.2	**>2662**	ND	6.893	**5317**
**Diox 14**	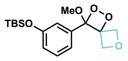	ND	<0.2	**>2662**	ND	4.315	**3329**
**Diox 15**	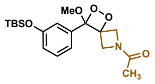	ND	<0.2	**>2662**	ND	ND	**ND**

Figure **S19**: Comparison between the Relative chemiexcitation rate calculated according to the Half-life value ($T_{1/2}$) or Rate constant of Diox 1-Diox 15 [10 nM] in DMSO or Acetone, with TBAF [10 mM]. All measurements were conducted using SpectraMax iD3, with injector settings fixed on an integration time of 50 msec.

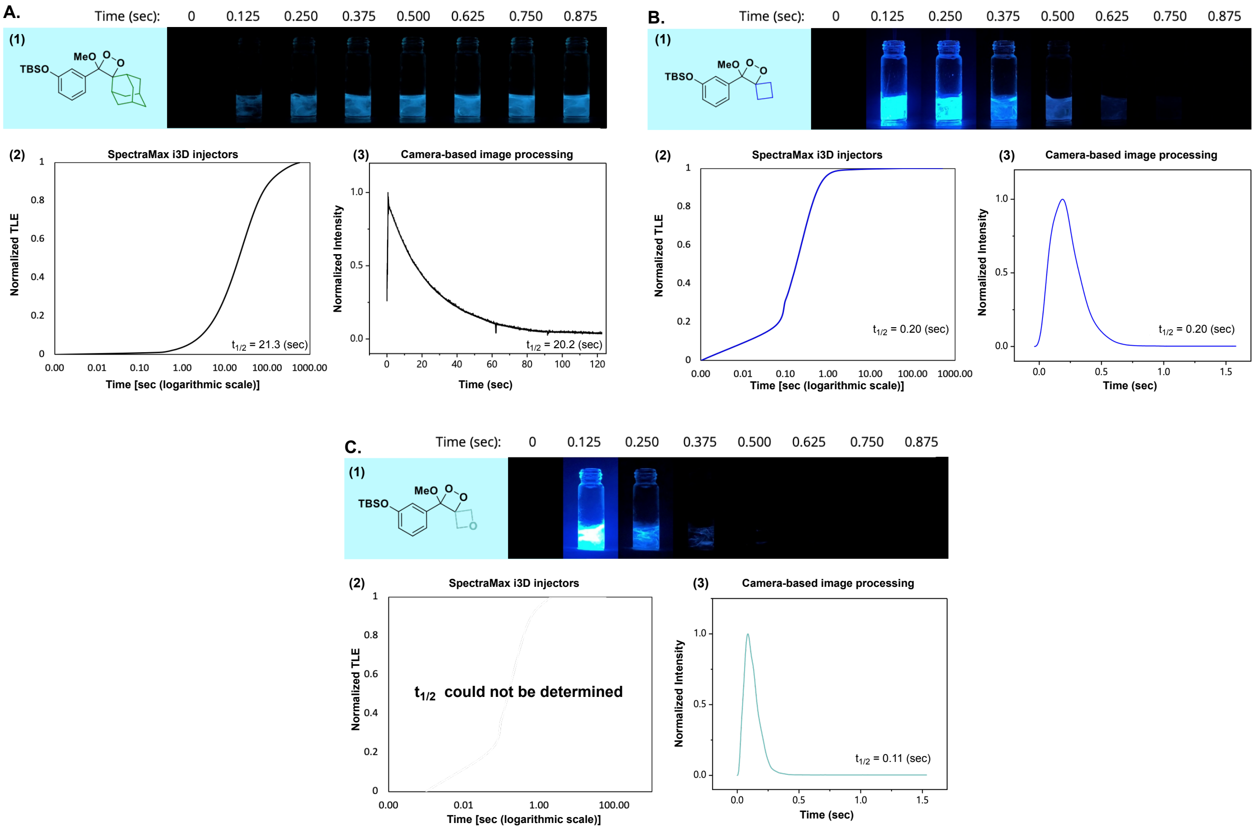

Figure **S20**: A. **Diox 1** [500 μM], B. **Diox 8** [500 μM], and C. **Diox 14** [500 μM] 1) Visual representation of the light emission profile during 1 sec in the presence of TBAF [10 mM] in DMSO. 2) Normalized total light emission kinetic profile (time represented in logarithmic scale) measured by SpectraMax i3D (protocol described in Experimental Protocols). 3) Relative light intensity profile derived from the camera-based image processing.

For **Diox 14**, as a result of the SpectraMax i3D instrument limitation (0.3 sec delay from the injection to the start of the measurement) normalized total light emission kinetic profile could not be obtained and $t_{1/2}$ values were determined as faster than 0.2 sec.

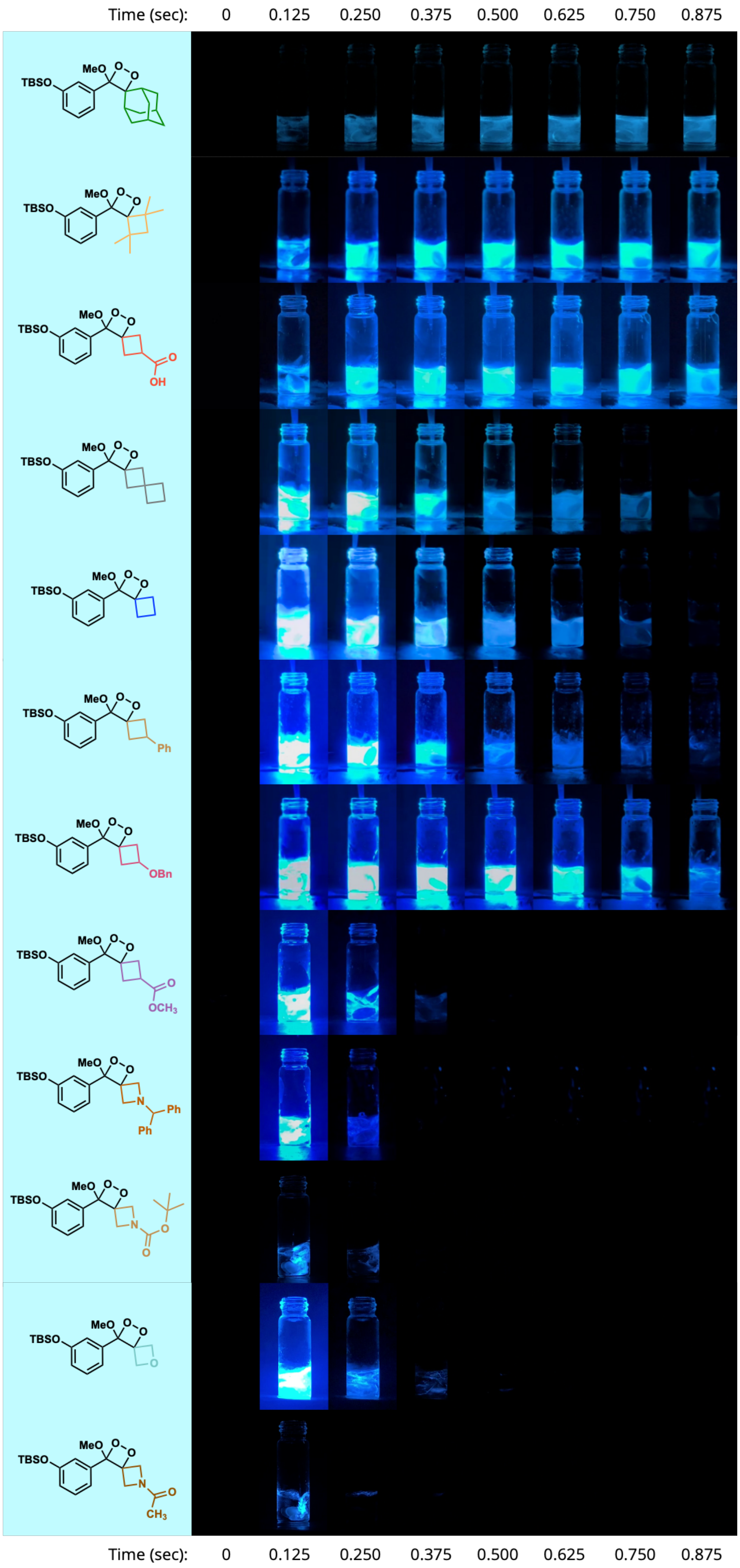

Figure **S21**: Visual representation of the light emission profile of **Diox 1** and **Diox 3 - Diox 15** [500 µM] during 1 sec in the presence of TBAF [10 mM] in DMSO.

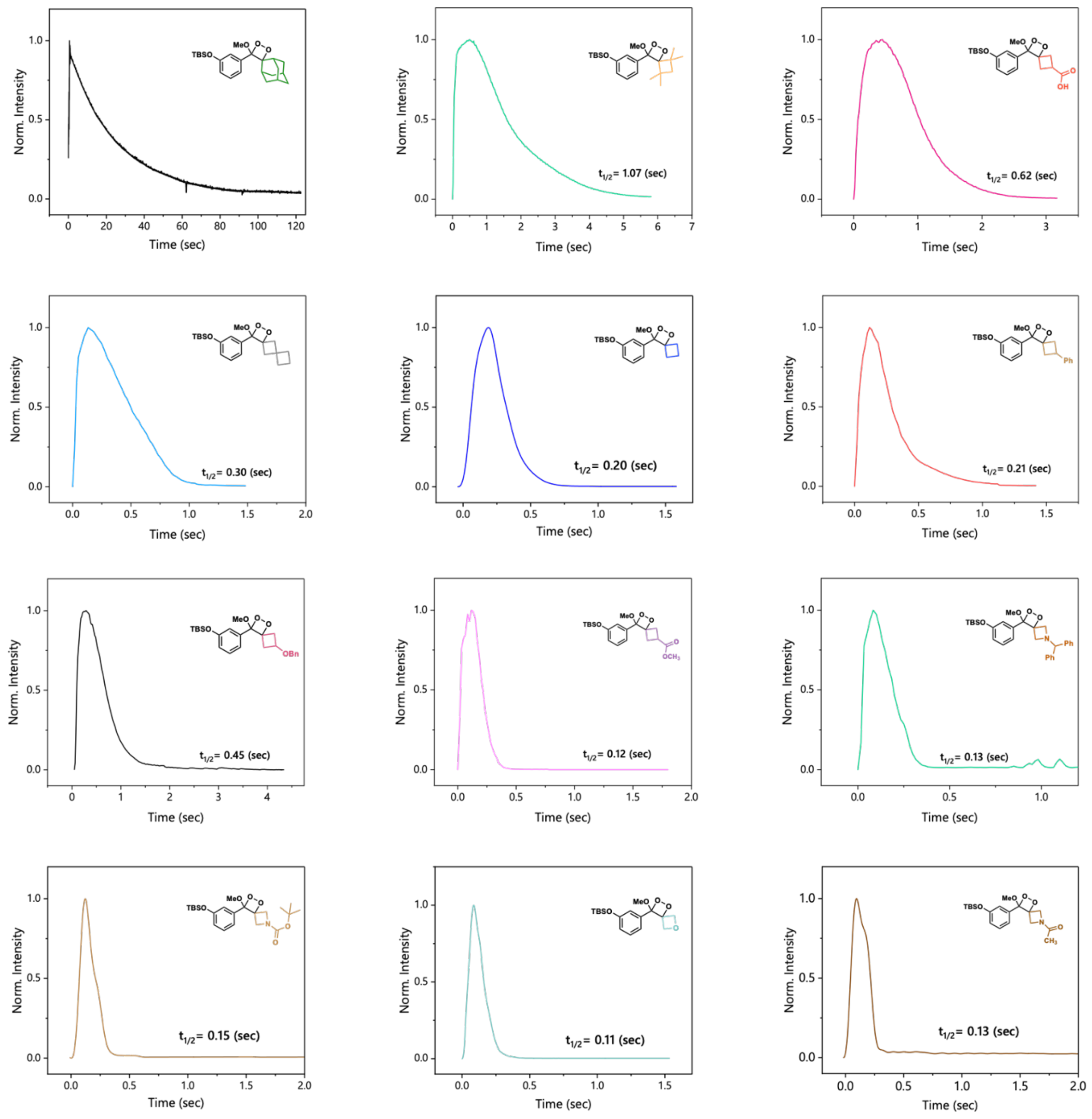

Figure **S22**: Normalized kinetic profiles of **Diox 1** and **Diox 3 - Diox 15** [500 μM] in the presence of TBAF [10 mM] in DMSO. Normalized intensity was derived from the camera-based image processing.

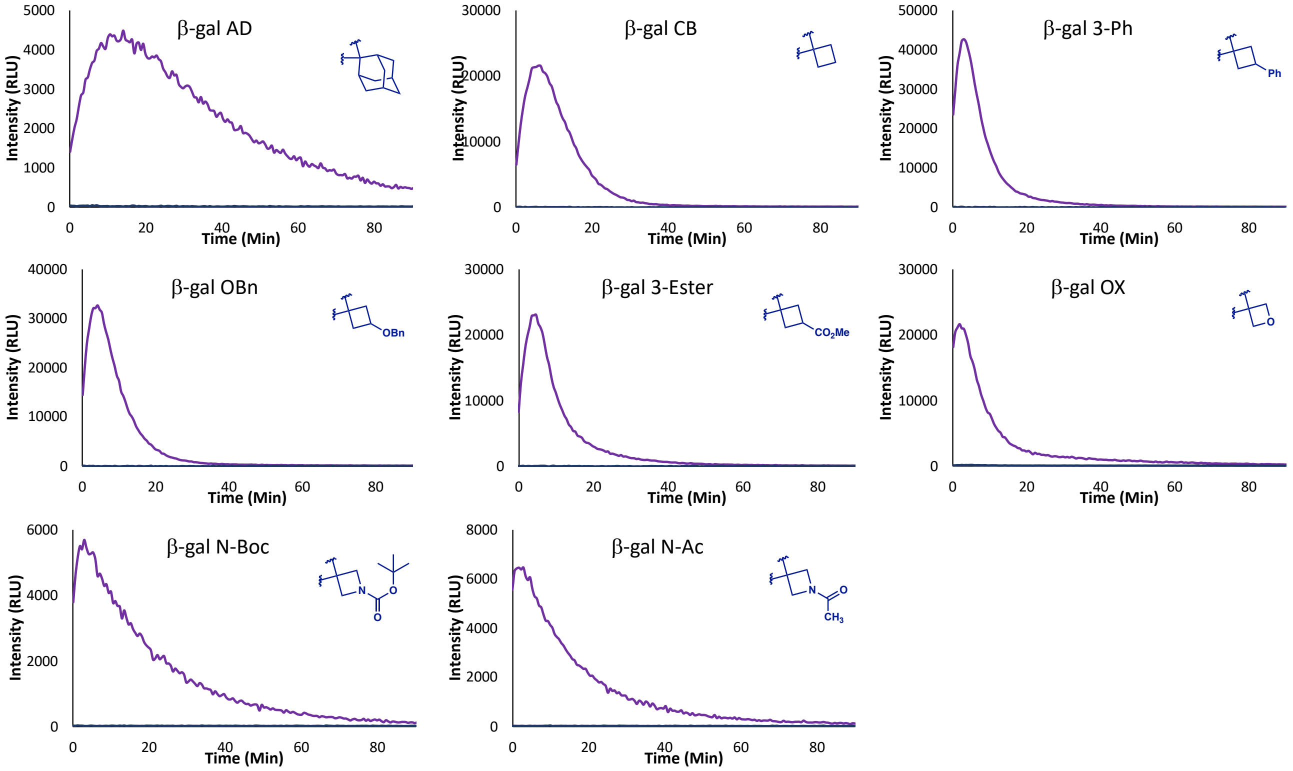

Figure **S23**: Chemiluminescent kinetic profiles of the eight β-gal probes [10 μM] with and without β-galactosidase [2 U/mL], PBS pH 7.4, 10% ACN.

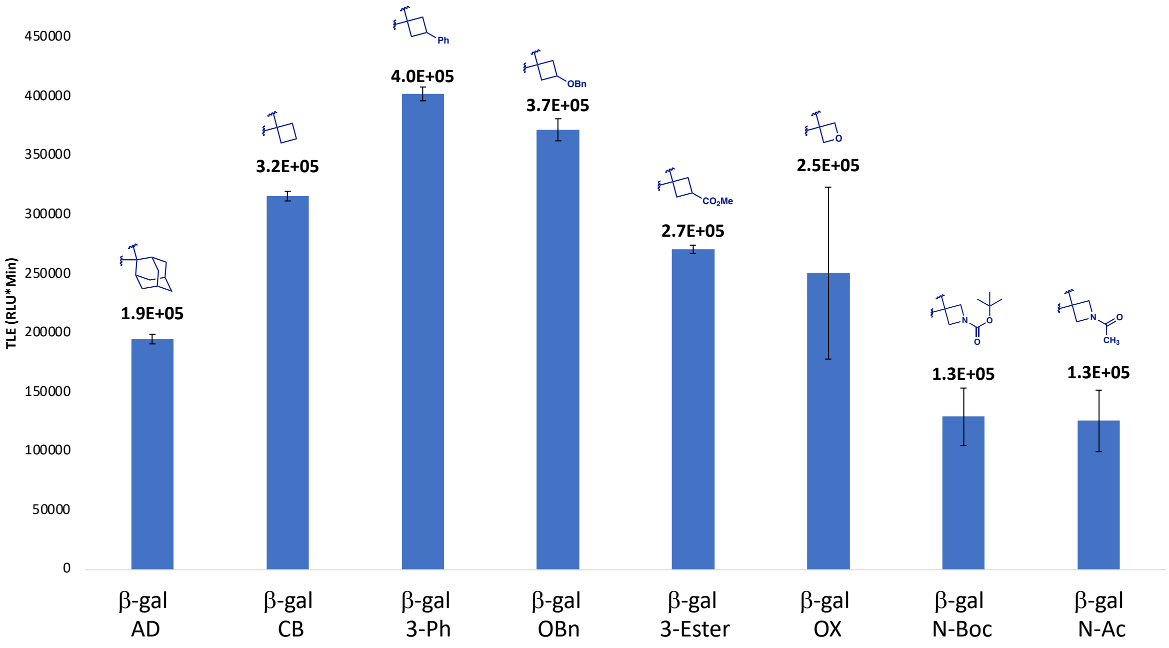

Figure **S24**: Total light emitted during 80 minutes by the eight β-gal probes [10 μM] with and without β-galactosidase [2 U/mL], PBS pH 7.4, 10% ACN.

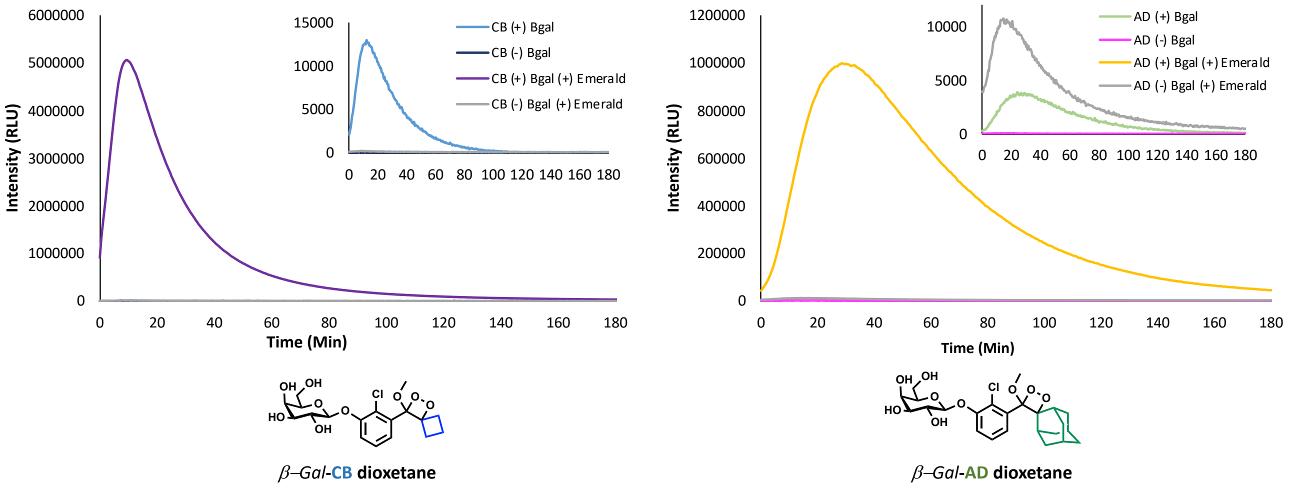

Figure **S25**: Chemiluminescent kinetic profiles of β-gal-**Cyclobutyl-dioxetane** (left) [10μM] and β-gal-**Adamantyl-dioxetane** (right) [10μM] in PB pH 7.4, 5% DMF, 37°C with β-galactosidase [2 U/mL] with and without 10% *Emerald II.*

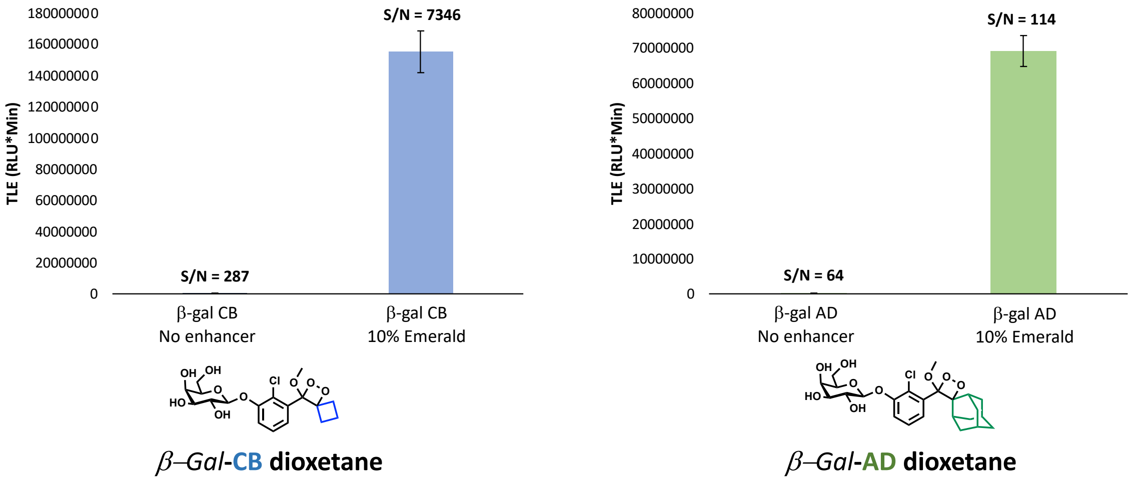

Figure **S26**: Total light emitted during 3 hours by β-gal-**Cyclobutyl-dioxetane** (left) [10μM] and β-gal-**Adamantyl-dioxetane** (right) [10μM] in PB pH 7.4, 5% DMF, 37°C with β-galactosidase [2 U/mL] with and without 10% *Emerald II* enhancer.

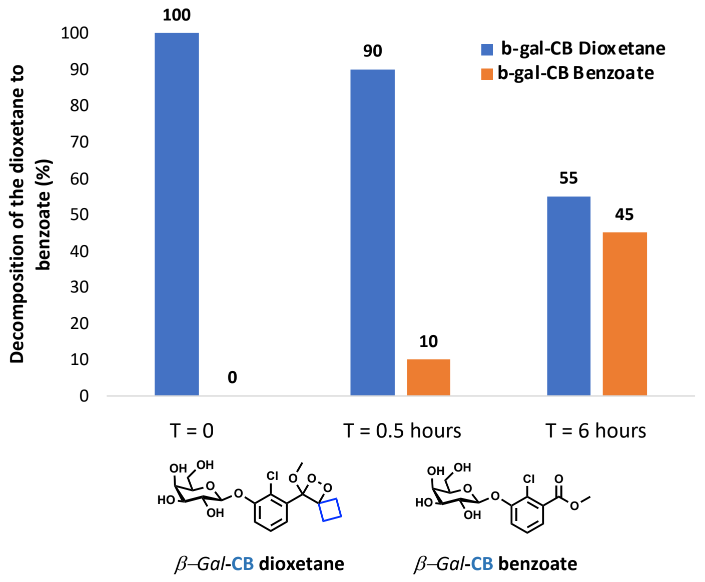

Figure **S27**: HPLC stability analysis of β-gal-**Cyclobutyl-dioxetane** (100 μM) with *Emerald II* enhancer (5%). After **30 minutes** 10% decomposition to β-gal-CB benzoate is observed. After **6 hours** 45% decomposition to β-gal-CB benzoate is observed.

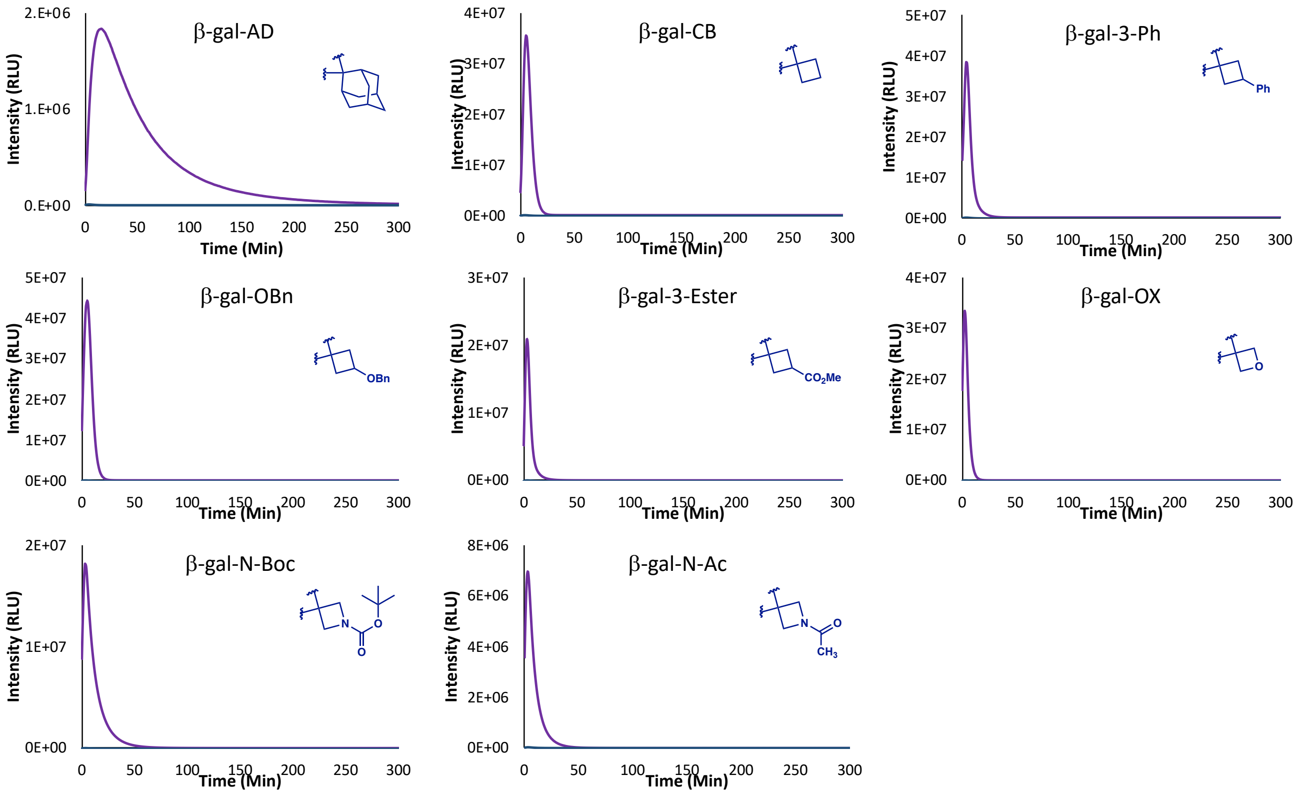

Figure **S28**: Chemiluminescent kinetic profiles of the eight β-gal probes [10 μM] with and without β-galactosidase [2 U/mL], in the presence of 5% *Emerald II* enhancer, PB pH 7.4, 1% ACN.

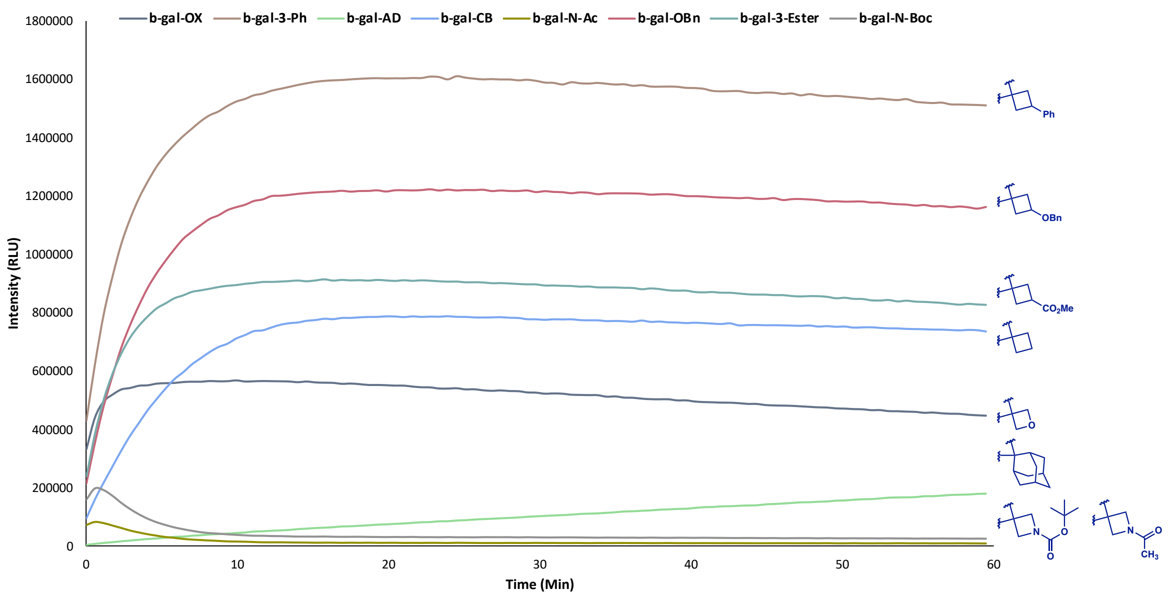

Figure **S29**: Chemiluminescent kinetic profiles of the eight β-gal probes [100 μM] with β-galactosidase [0.004 U/mL], in the presence of 5% *Emerald II* enhancer, PB pH 7.4, 1% ACN.

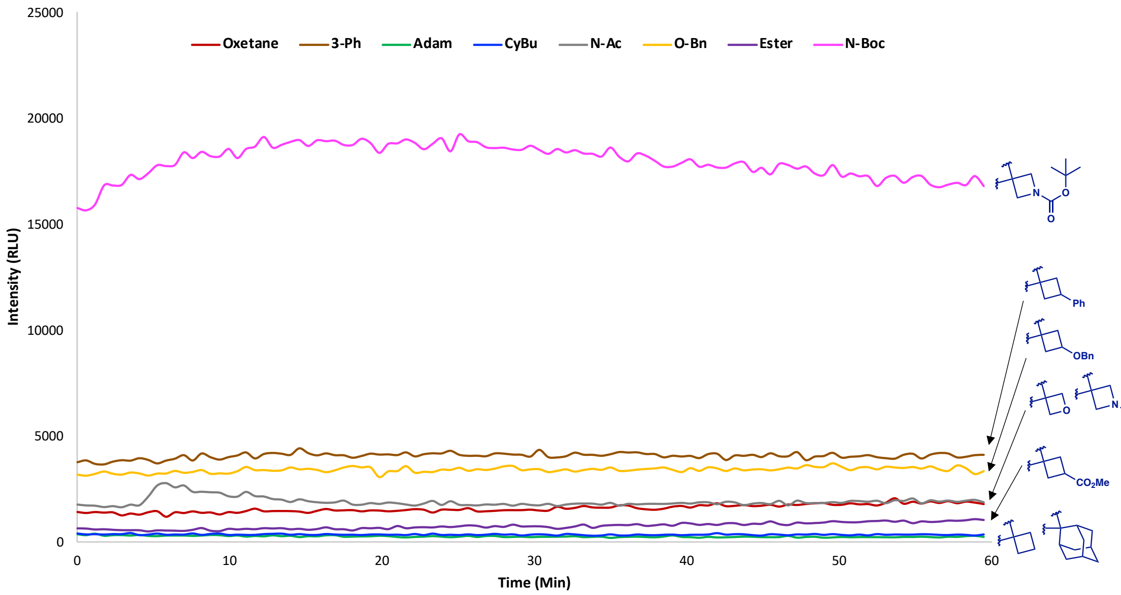

Figure **S30**: **Background comparison**. Chemiluminescent kinetic profiles of the eight β-gal probes [100 μM] without β-galactosidase in the presence of 5% *Emerald II* enhancer, PB pH 7.4, 1% ACN.

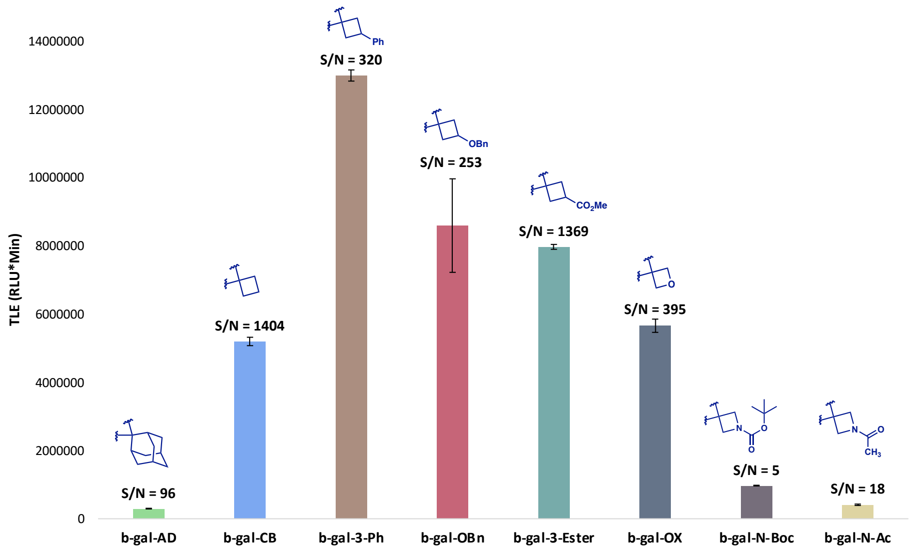

Figure **S31**: Total light emitted during **10 minutes** by the eight β-gal probes [100 μM] with β-galactosidase [0.004 U/mL], in the presence of 5% *Emerald II* enhancer, PB pH 7.4, 1% ACN.

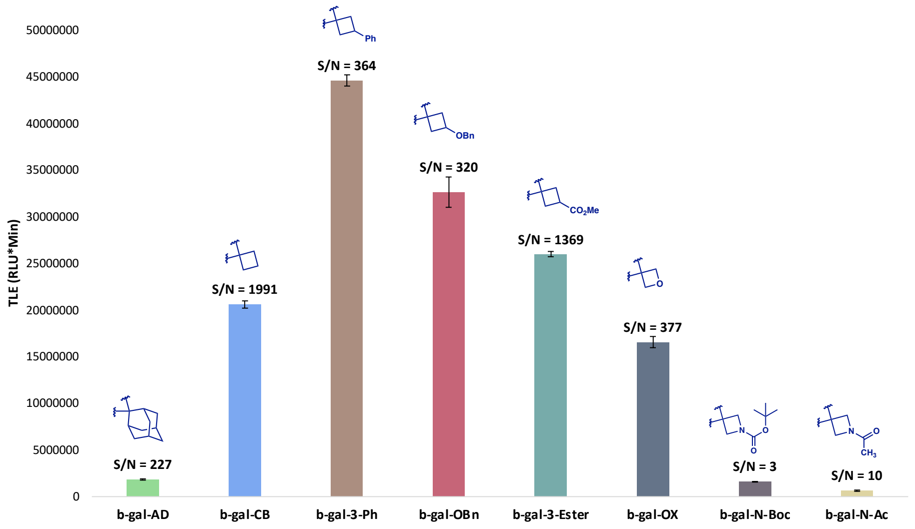

Figure **S32**: Total light emitted during **30 minutes** by the eight β-gal probes [100 μM] with β-galactosidase [0.004 U/mL], in the presence of 5% *Emerald II* enhancer, PB pH 7.4, 1% ACN.

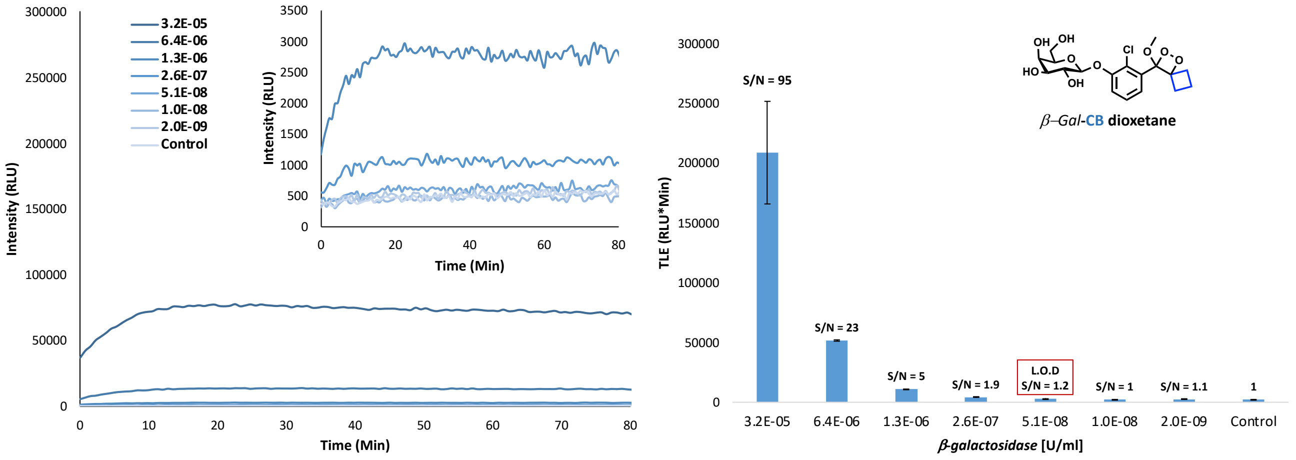

Figure **S33**: Chemiluminescent kinetic profiles and the total light emitted during 5 minutes by β-gal-**Cyclobutyl-dioxetane** [100 μM] with various concentrations of β-galactosidase [3.2*10^{-5} - 2.0*10^{-9} U/mL], in the presence of 5% *Emerald II* enhancer, PB pH 7.4, 1% ACN. Limit-of-detection (L.O.D) was defined as blank + 3SD (standard deviation).

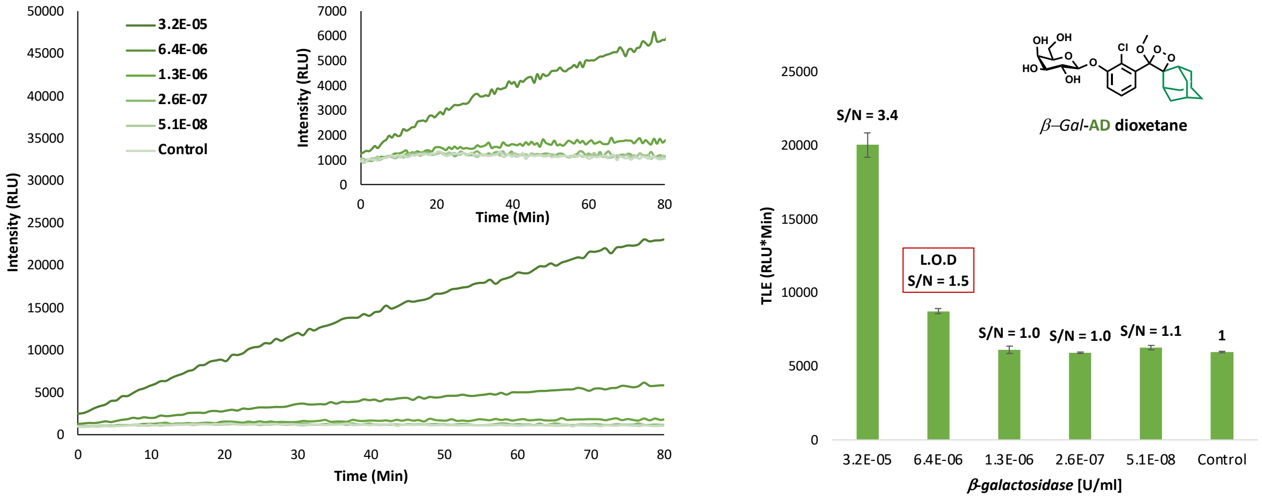

Figure **S34**: Chemiluminescent kinetic profiles and the total light emitted during 5 minutes by β-gal-**Adamantyl-dioxetane** [100 μM] with various concentrations of β-galactosidase [3.2*10^{-5} – 5.1*10^{-8} U/mL], in the presence of 5% *Emerald II* enhancer, PB pH 7.4, 1% ACN. Limit-of-detection (L.O.D) was defined as blank + 3SD (standard deviation).

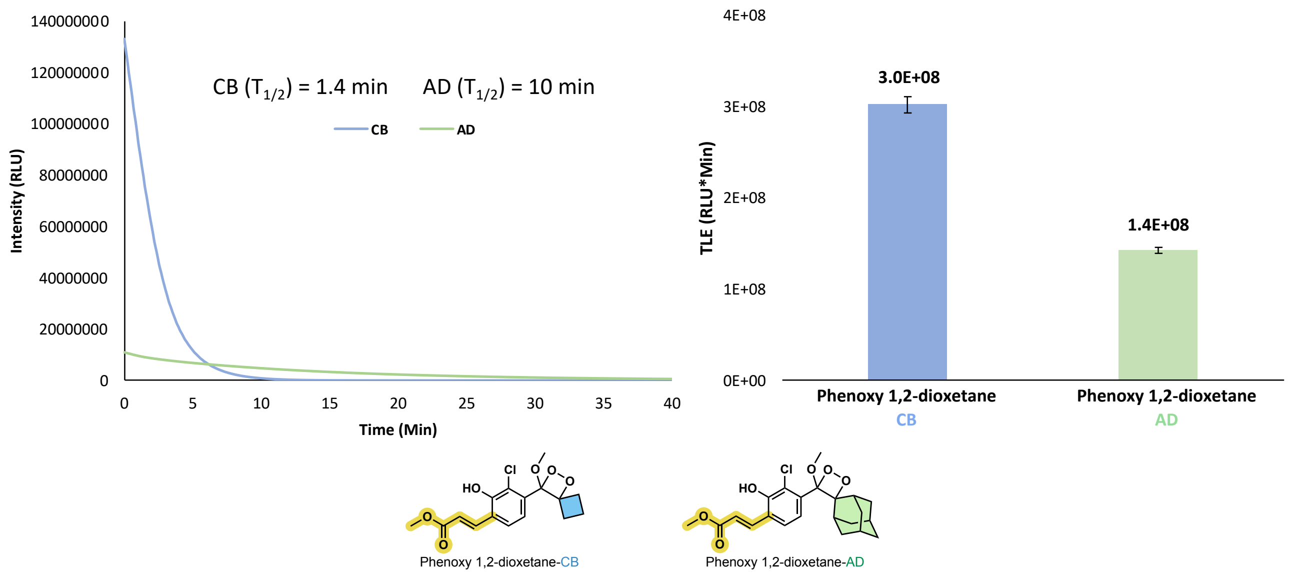

Figure **S35**: Chemiluminescent kinetic profiles and the total light emitted during 30 minutes by phenoxy 1,2-dioxetane **CB** and of phenoxy 1,2-dioxetane **AD** [10μM] in PBS pH 7.4, 10% ACN, at room temperature.

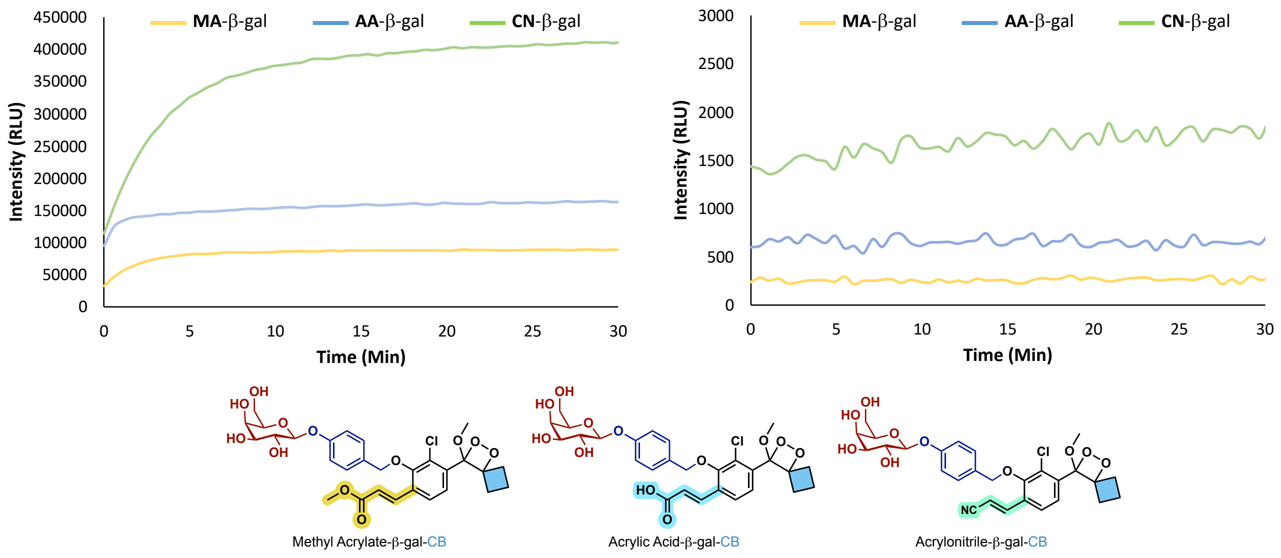

Figure **S36**: Chemiluminescent kinetic profiles of **MA CB, AA CB, and CN CB** [10μM] in PBS pH 7.4, 10% ACN, with (left) or without (right) β-galactosidase [0.001 U/mL] at room temperature.

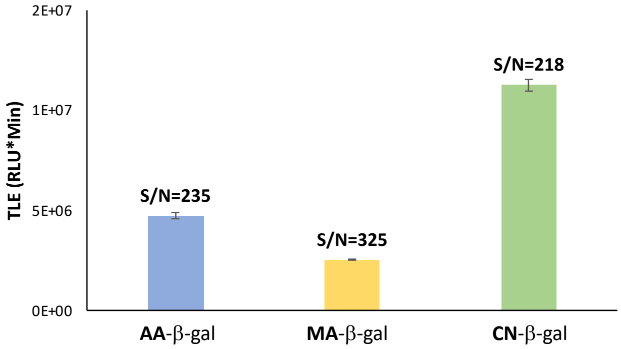

	MA-β-gal	AA-β-gal	CN-β-gal
Relative S/N	1.5	1.1	1

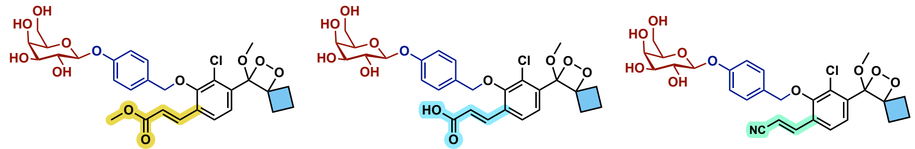

Methyl Acrylate-β-gal-CB　Acrylic Acid-β-gal-CB　Acrylonitrile-β-gal-CB

Figure **S37**: Total light emitted during 30 minutes by β-gal **MA CB, AA CB, and CN CB** [10μM] in PBS (pH 7.4, 10% ACN) with β-galactosidase [0.001 U/mL] at room temperature.

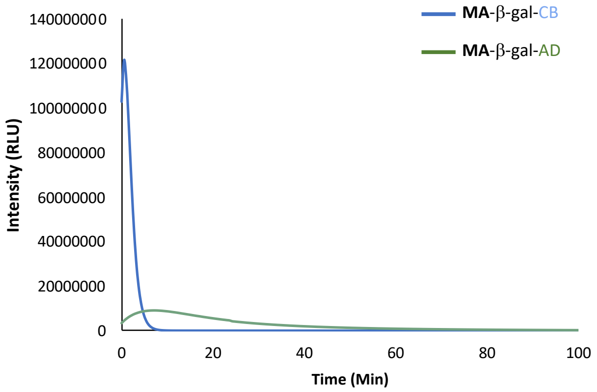

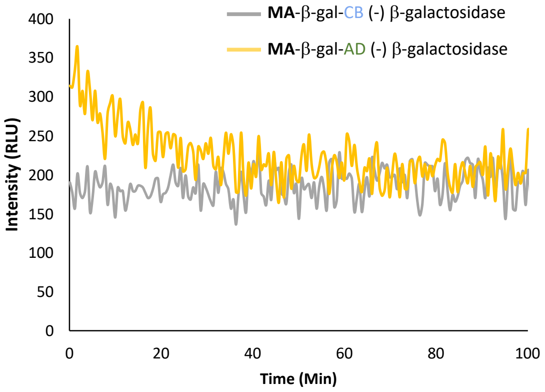

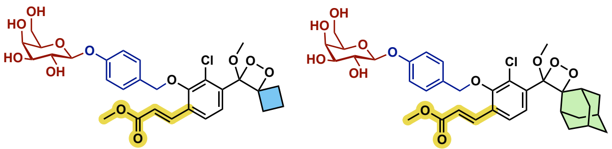

MA-β-gal-CB　MA-β-gal-AD

Figure **S38**: Chemiluminescent kinetic profiles of **MA β-gal CB** and **MA β-gal AD** [10μM] in PBS pH 7.4, 10% ACN with (left) or without (right) of β-galactosidase [20 U/mL] at room temperature.

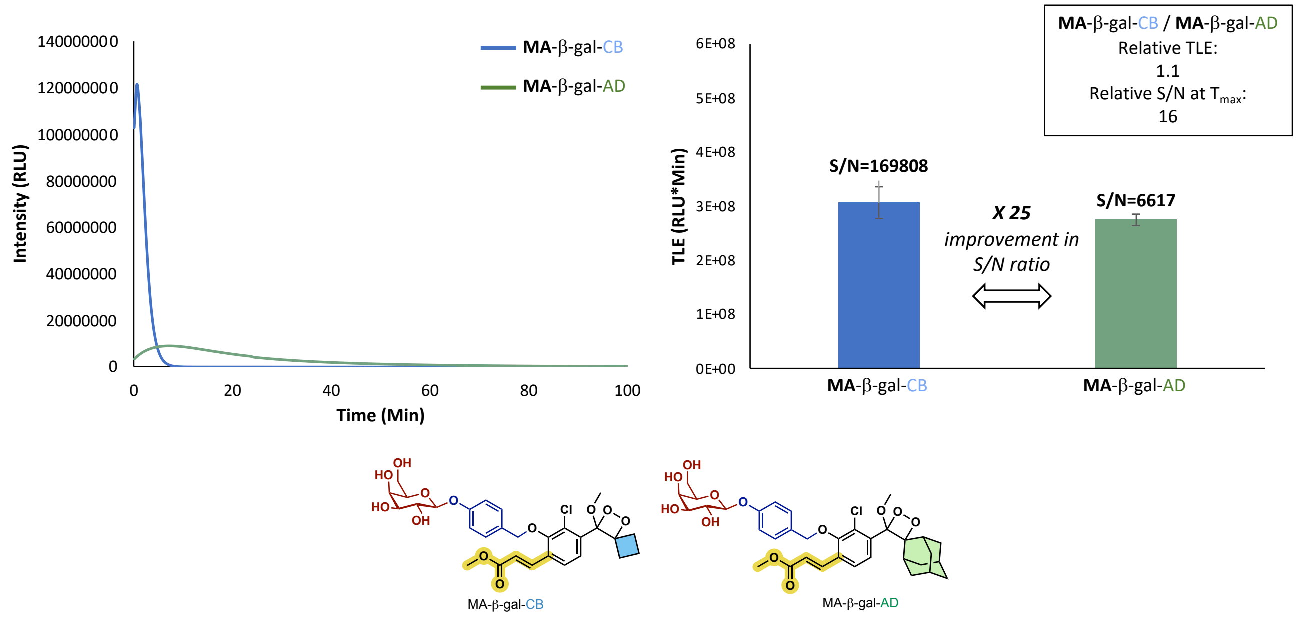

Figure **S39**: Chemiluminescent kinetic profiles and the total light emitted during 30 minutes by **MA β-gal CB** and **MA β-gal AD** [10µM] with or without β-galactosidase [20 U/mL] in PBS pH 7.4, 10% ACN, at room temperature.

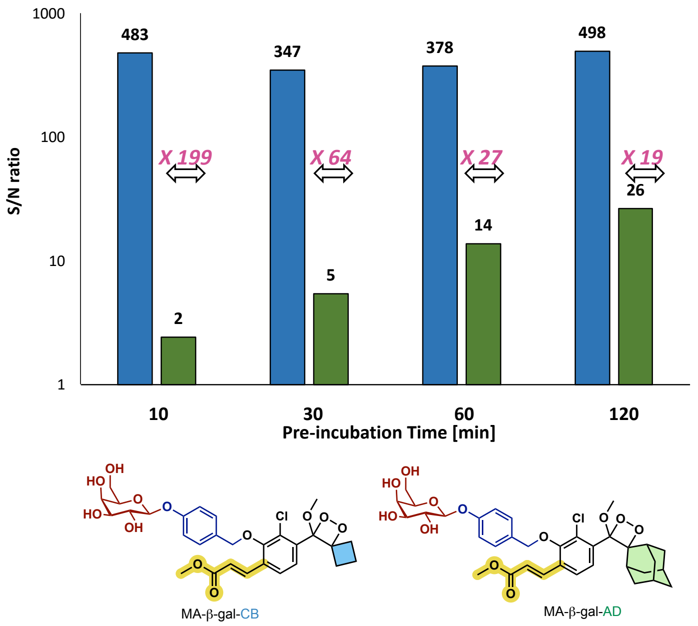

Figure **S40**: Signal-to-noise ratio (of total light emitted during 15 min) in a logarithmic scale, as a function of different pre-incubation durations: 10, 15 30, 60 mins, obtained from **MA β-gal CB** and **MA β-gal AD** [10µM] with or without β-galactosidase [0.001 U/mL], PBS pH 7.4, 10% ACN, at room temperature.

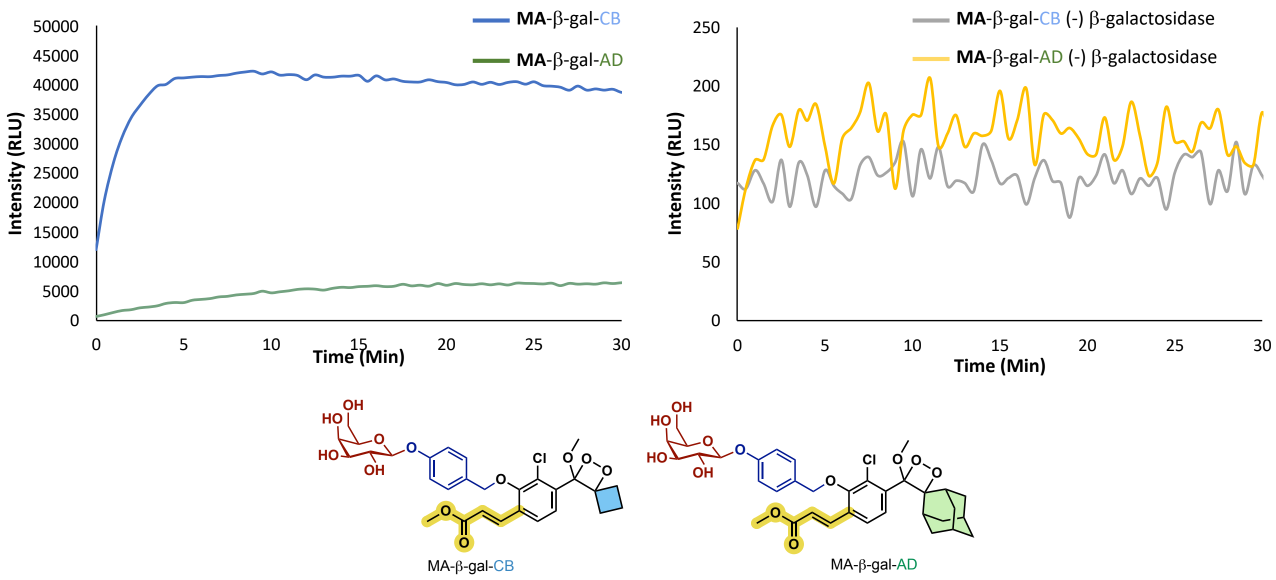

Figure **S41**: Chemiluminescence kinetic profile of **MA β-gal CB** and **MA β-gal AD** [10μM] in PBS pH 7.4, 10% ACN with (left) or without (right) of β-galactosidase [0.001 U/mL] at room temperature.

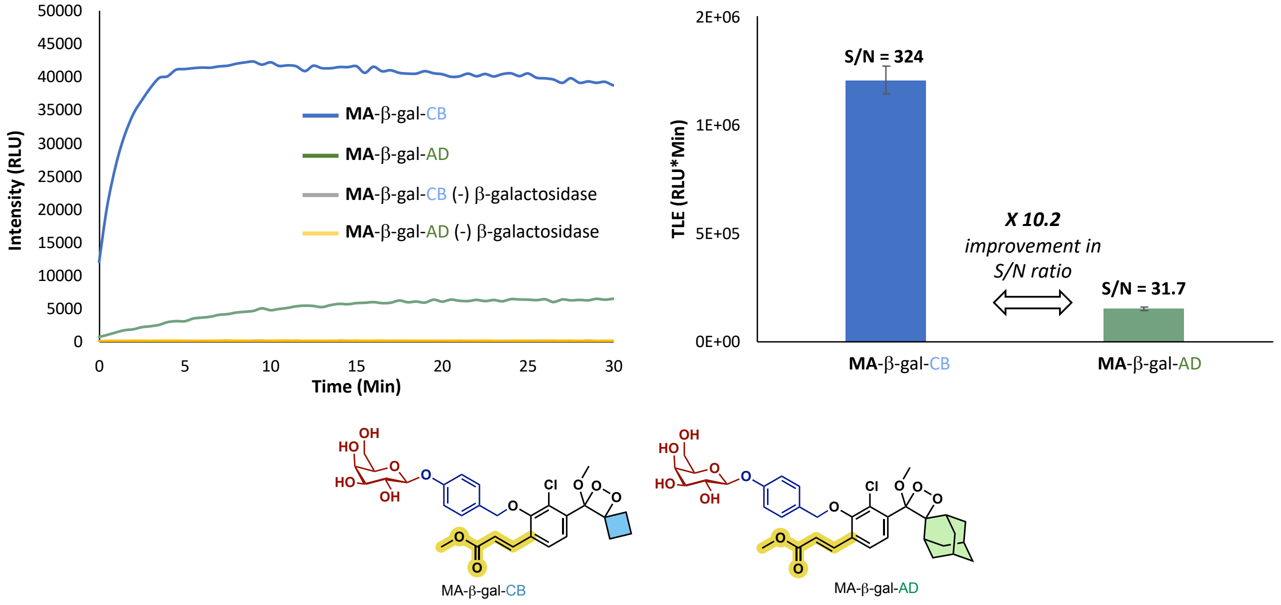

Figure **S42**: Chemiluminescent kinetic profiles and the total light emitted during **30 minutes** by **MA β-gal CB** and **MA β-gal AD** [10μM] with or without β-galactosidase [0.001 U/mL] in PBS pH 7.4, 10% ACN, at room temperature.

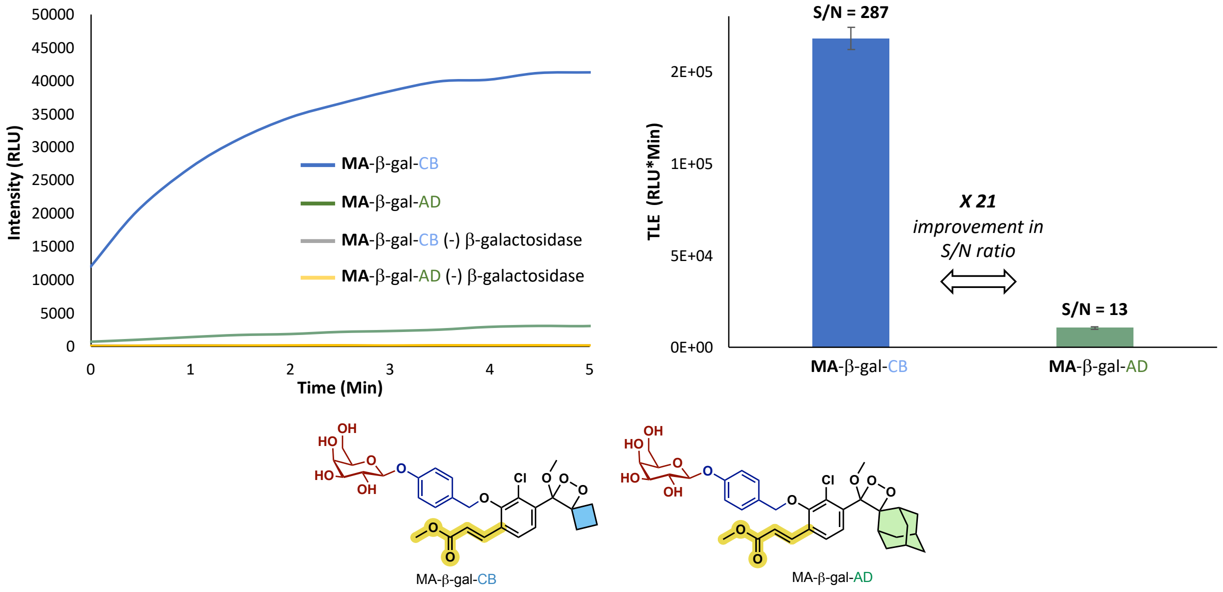

Figure **S43**: Chemiluminescent kinetic profiles and the total light emitted during **5 minutes** by **MA β-gal CB** and **MA β-gal AD** [10μM] with or without β-galactosidase [0.001 U/mL] in PBS pH 7.4, 10% ACN, at room temperature.

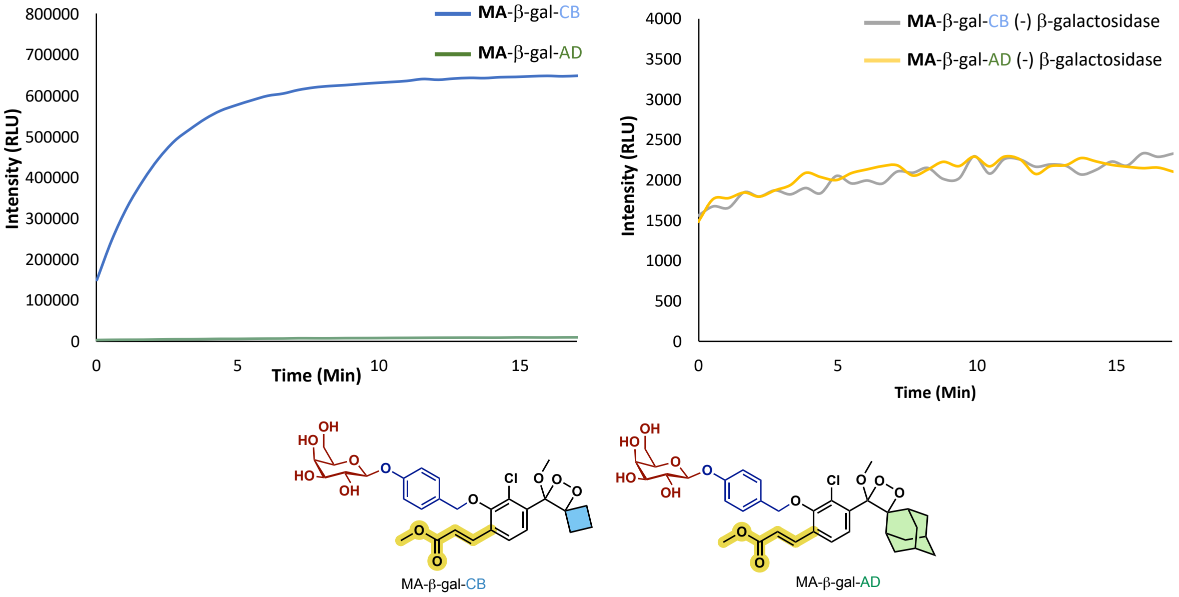

Figure **S44**: Chemiluminescent kinetic profiles of **MA β-gal CB** and **MA β-gal AD** [100μM] in PBS pH 7.4, 10% ACN with (left) or without (right) of β-galactosidase [0.001 U/mL] at room temperature.

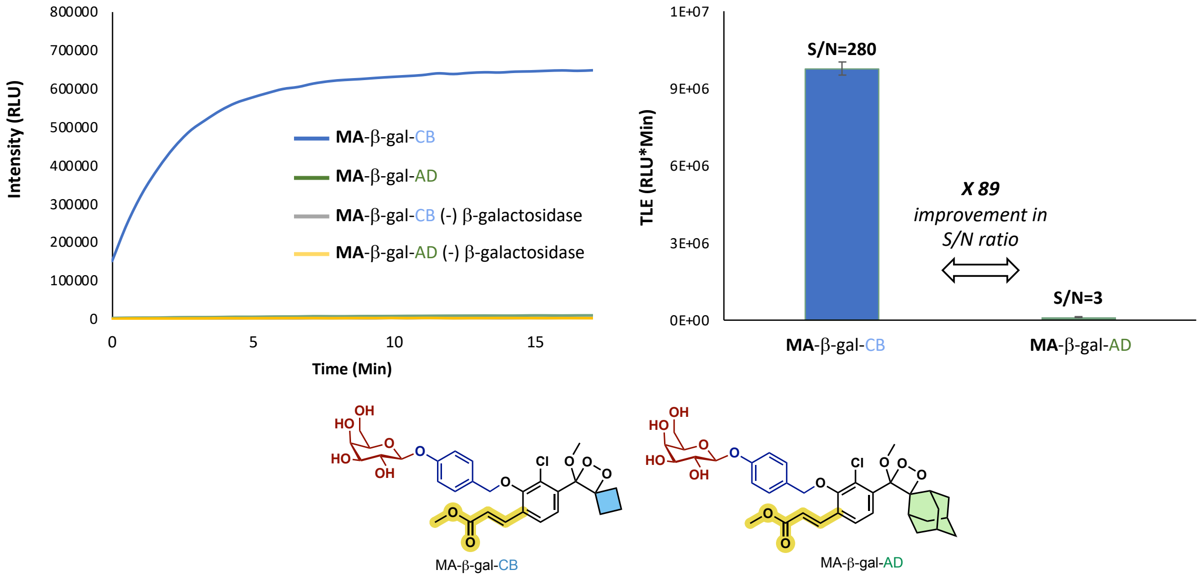

Figure **S45**: Chemiluminescent kinetic profiles and the total light emitted during 17 minutes by **MA β-gal CB** and **MA β-gal AD** [100µM] with or without β-galactosidase [0.001 U/mL] in PBS pH 7.4, 10% ACN, at room temperature.

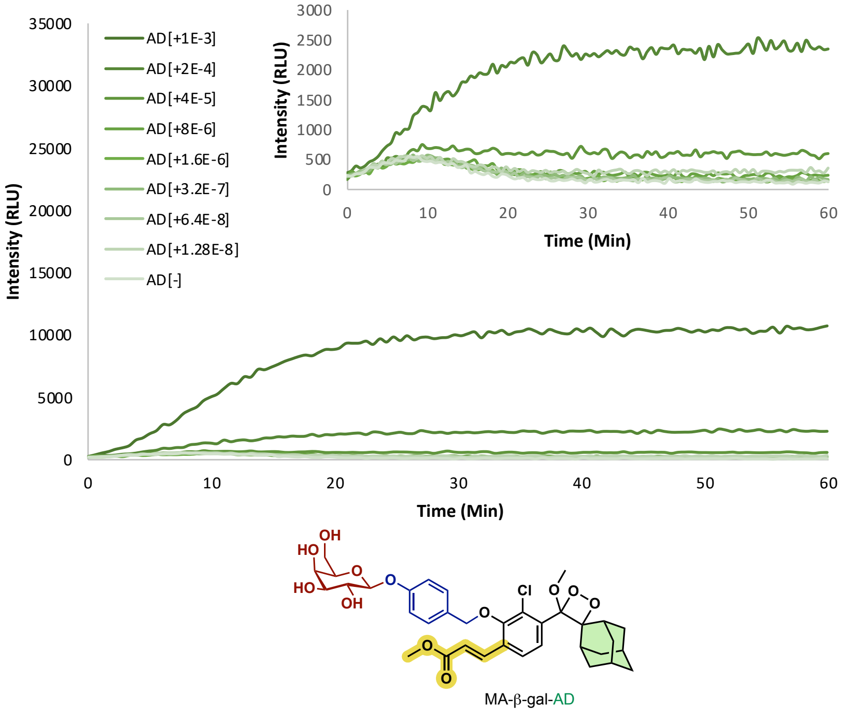

Figure **S46**: Chemiluminescent kinetic profiles of **MA β-gal AD** [10 µM] with varying concentrations of β-galactosidase [$1.0*10^{-3}$ - $1.0*10^{-8}$ U/mL in PBS pH 7.4, 1% ACN, 37°C.

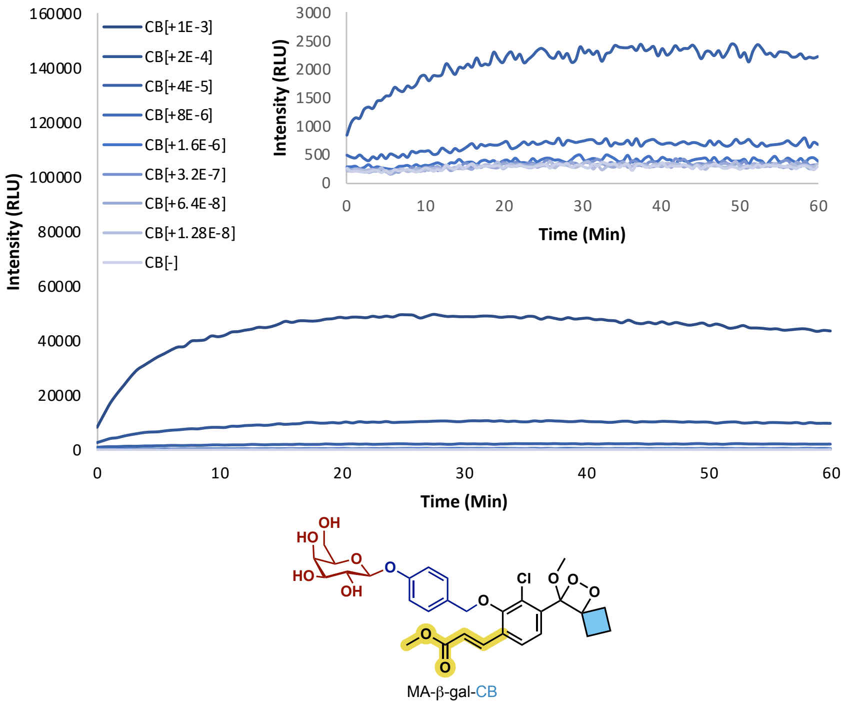

Figure **S47**: Chemiluminescent kinetic profiles of **MA β-gal CB** [10 μM] with varying concentrations of β-galactosidase [1.0*10^{-3} - 1.0*10^{-8} U/mL in PBS pH 7.4, 1% ACN, 37°C.

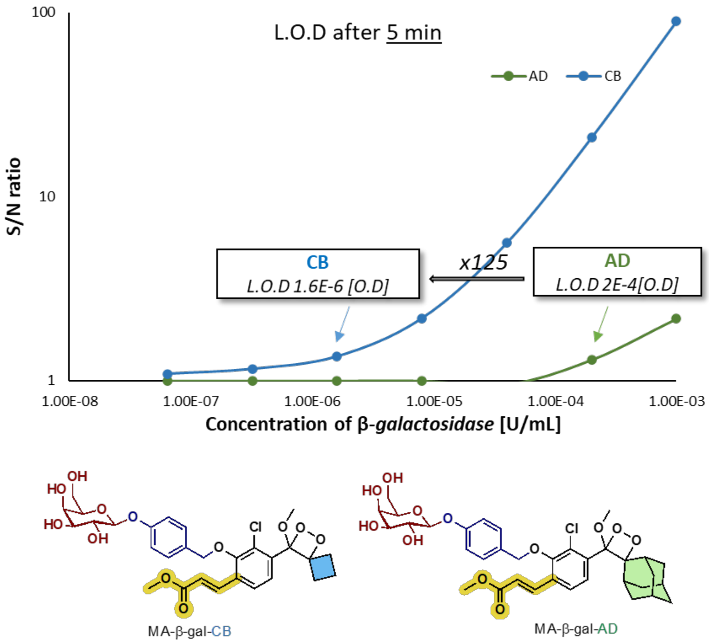

Figure **S48**: Signal-to-Noise ratio after **5 minutes** of **MA β-gal CB** and **MA β-gal AD** [10μM] with varying concentrations of β-galactosidase [1.0*10^{-3} - 1.0*10^{-8} U/mL] in PBS pH 7.4, 1% ACN, 37°C.

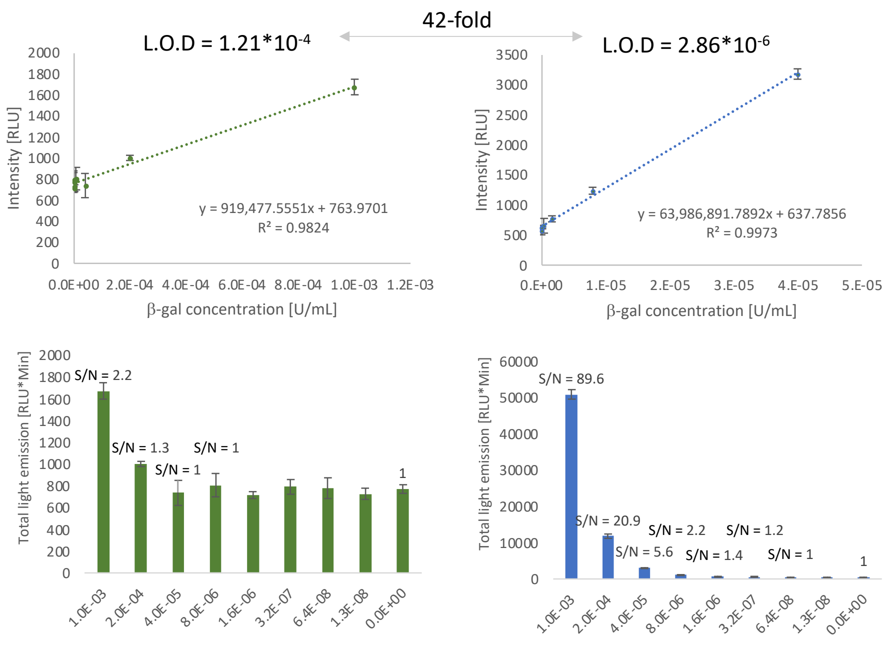

Figure **S49**: linear calibration curve and the total light emission after **5 minutes** of **MA β-gal AD** (left) and **MA β-gal CB** (right)[10μM] with varying concentrations of β-galactosidase [$1.0*10^{-3}$ - $1.0*10^{-8}$ U/mL] in PBS pH 7.4, 1% ACN, 37°C. The limit of detection was determined as 3*(S.D. of the blank) divided by the slope of the linear calibration curve (L.O.D = 3σ/k). Error bars are S.D. from three independent experiments.

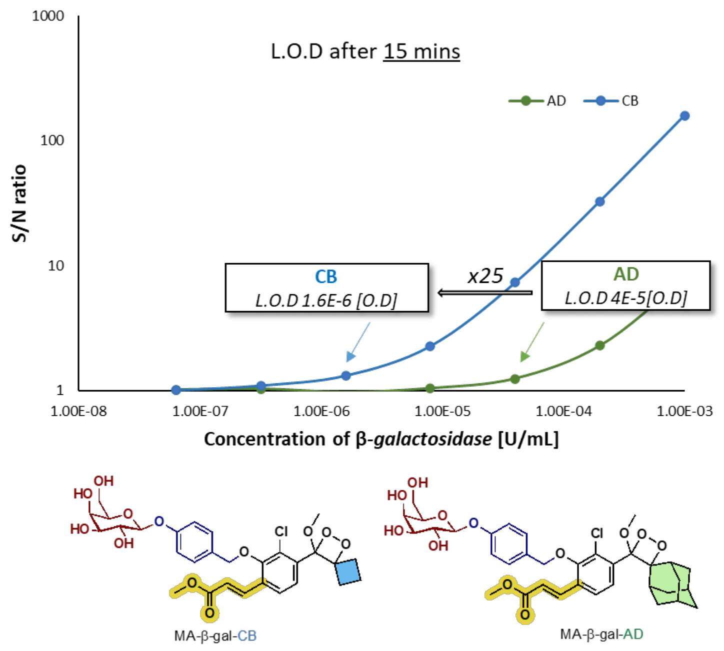

Figure **S50**: Signal-to-Noise ratio after **15 minutes** of **MA β-gal CB** and **MA β-gal AD** [10μM] with varying concentrations of β-galactosidase [$1.0*10^{-3}$ - $1.0*10^{-8}$ U/mL] in PBS pH 7.4, 1% ACN, 37°C.

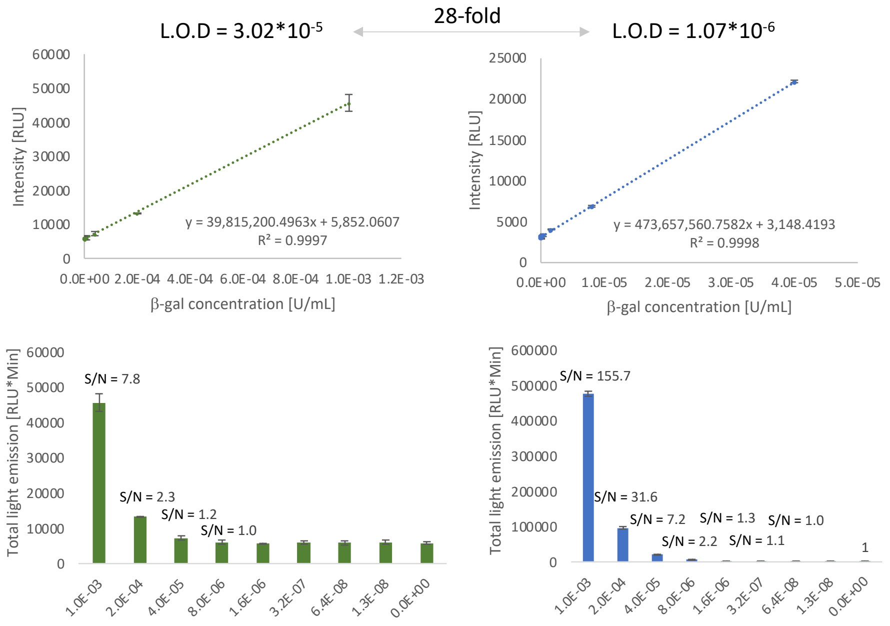

Figure **S51**: linear calibration curve and the total light emission after **15 minutes** of **MA β-gal AD** (left) and **MA β-gal CB** (right)[10μM] with varying concentrations of β-galactosidase [$1.0*10^{-3}$ - $1.0*10^{-8}$ U/mL] in PBS pH 7.4, 1% ACN, 37°C.

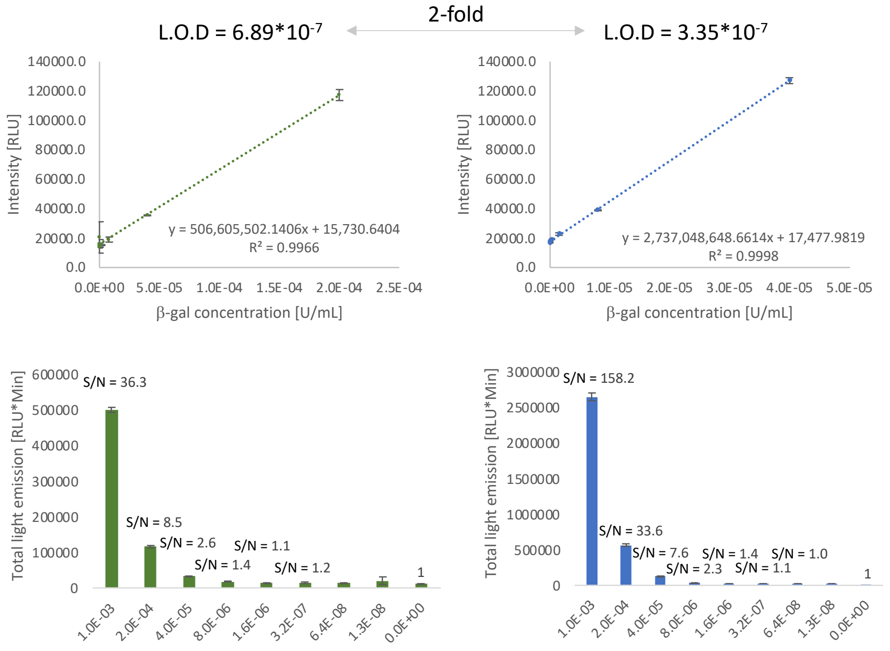

Figure **S52**: linear calibration curve and the total light emission after **60 minutes** of **MA β-gal AD** (left) and **MA β-gal CB** (right)[10μM] with varying concentrations of β-galactosidase [$1.0*10^{-3}$ - $1.0*10^{-8}$ U/mL] in PBS pH 7.4, 1% ACN, 37°C.

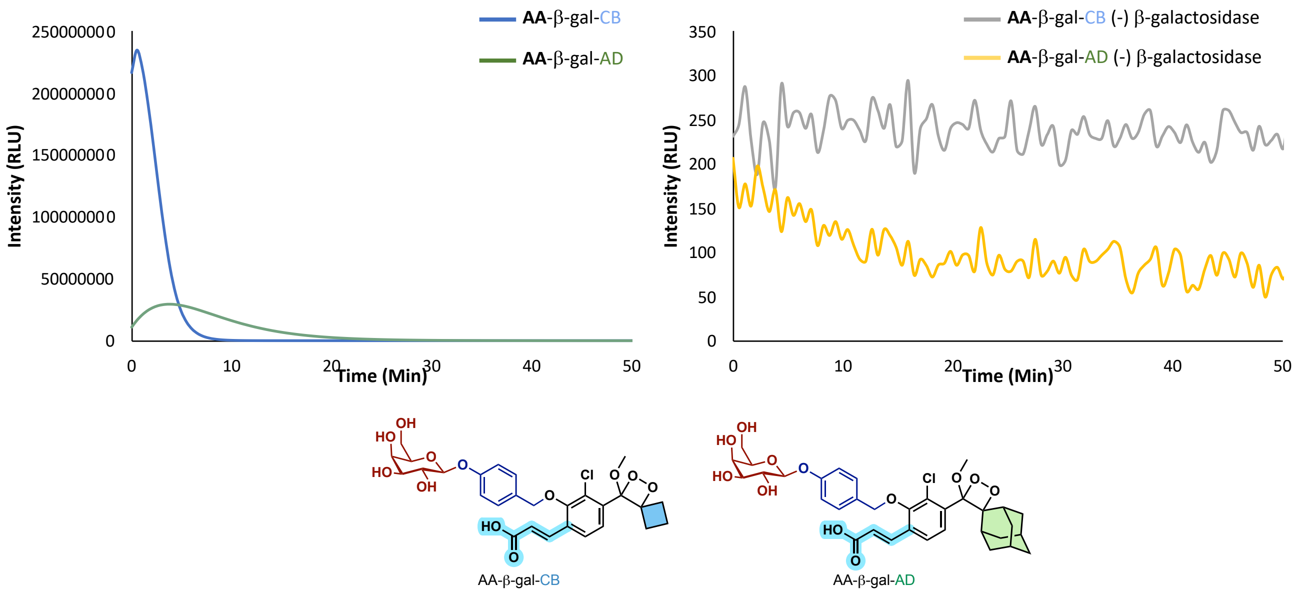

Figure **S53**: Chemiluminescent kinetic profiles of **AA β-gal CB** and **AA β-gal AD** [10µM] in PBS pH 7.4, 10% ACN with (left) or without (right) of β-galactosidase [20 U/mL] at room temperature.

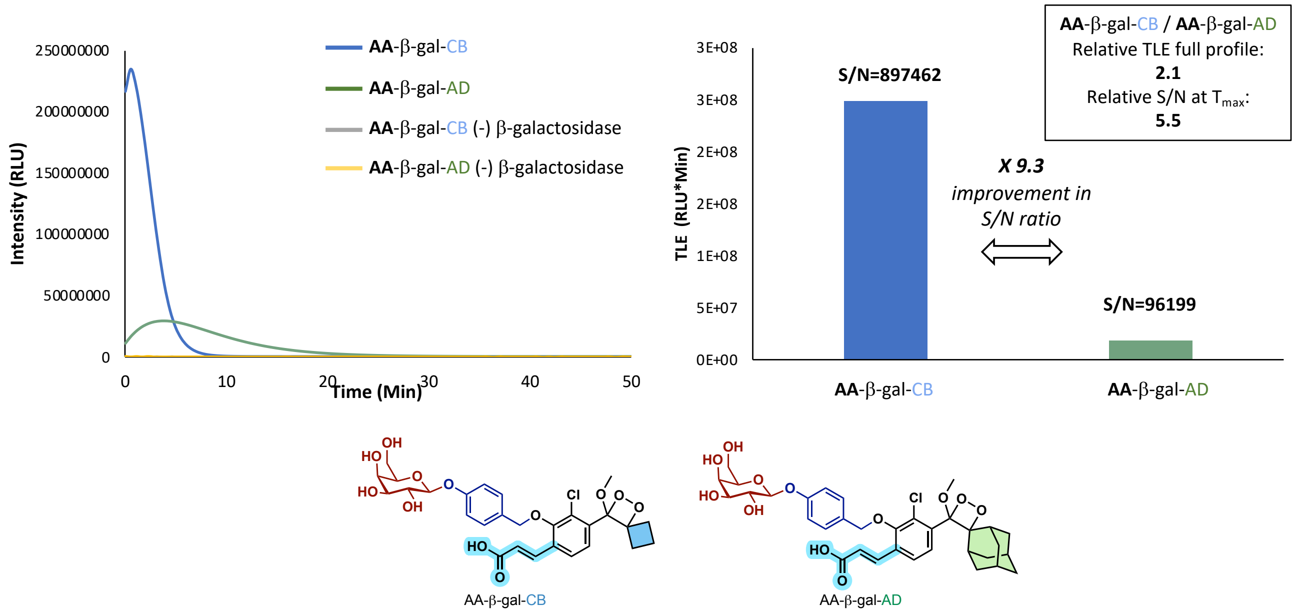

Figure **S54**: Chemiluminescent kinetic profiles and the total light emitted during 1 minute by **AA β-gal CB** and **AA β-gal AD** [10µM] with or without β-galactosidase [20 U/mL] in PBS pH 7.4, 10% ACN, at room temperature.

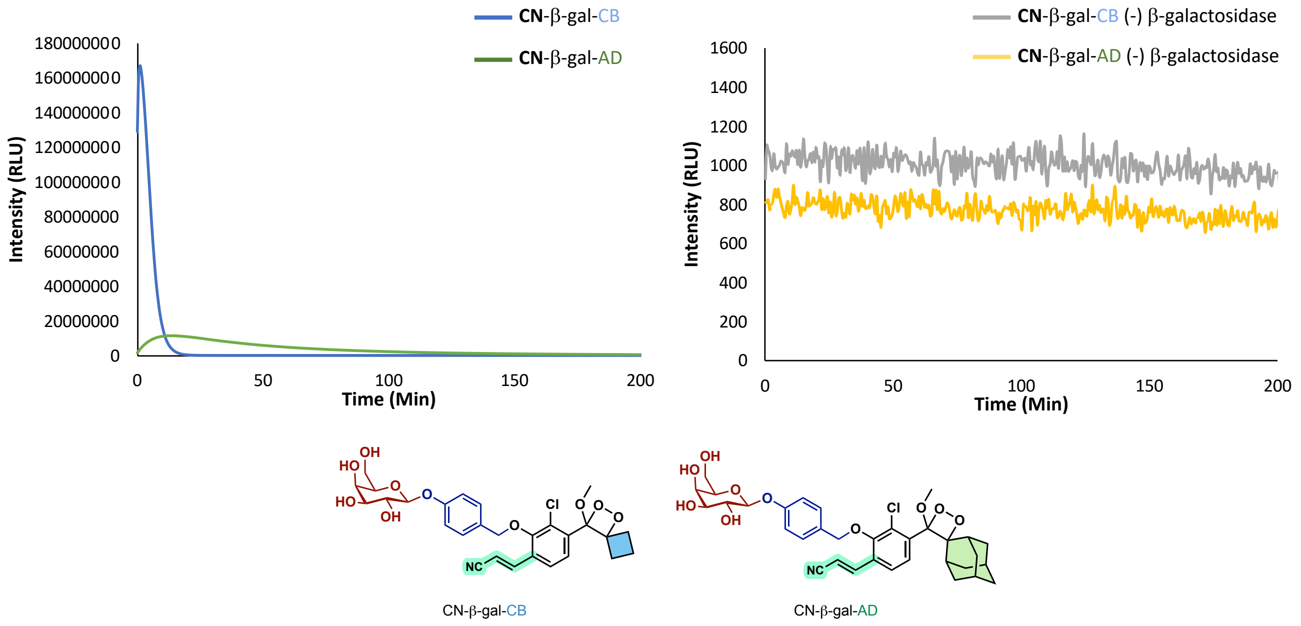

Figure **S55**: Chemiluminescent kinetic profiles of **CN β-gal CB** and **CN β-gal AD** [10µM] in PBS pH 7.4, 10% ACN with (left) or without (right) of β-galactosidase [20 U/mL] at room temperature.

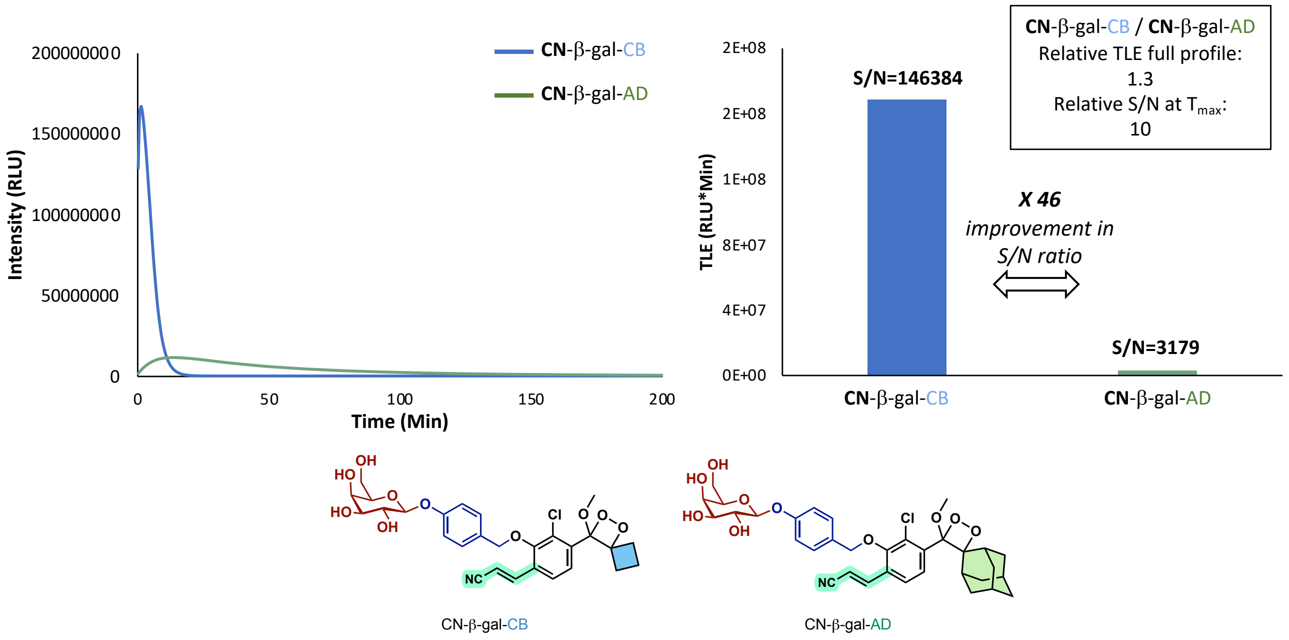

Figure **S56**: Chemiluminescent kinetic profiles and the total light emitted during 1 minute by **CN β-gal CB** and **CN β-gal AD** [10µM] with or without β-galactosidase [20 U/mL] in PBS pH 7.4, 10% ACN, at room temperature.

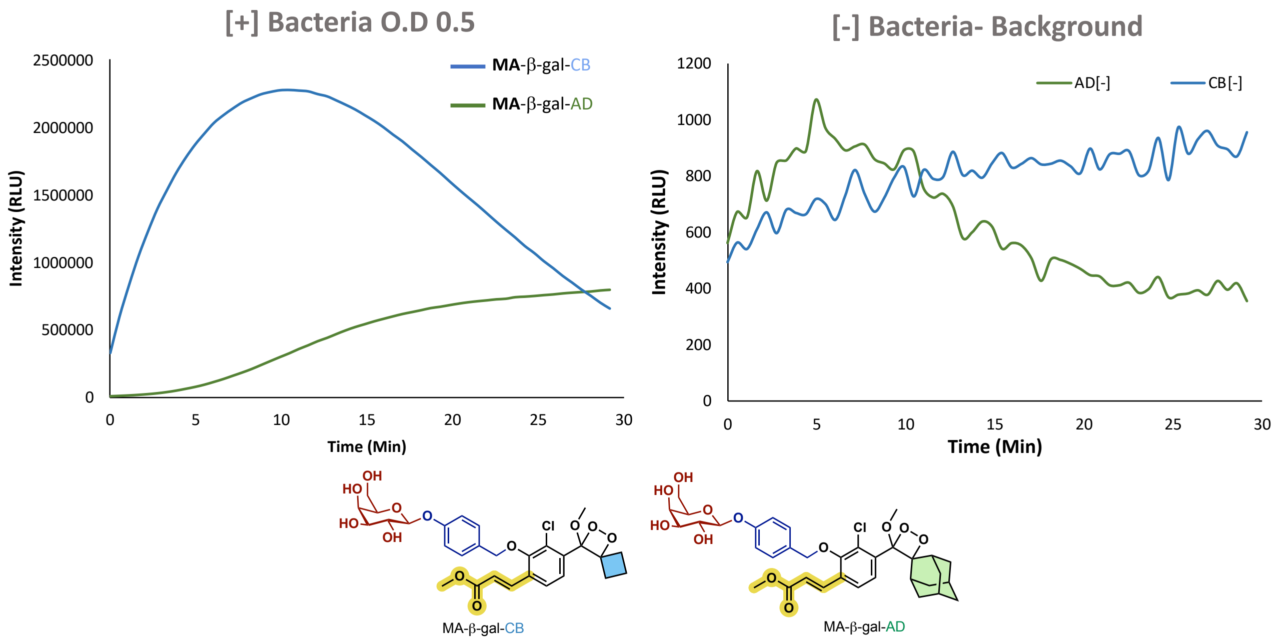

Figure **S57**: Chemiluminescent kinetic profiles of **MA β-gal CB** and **MA β-gal AD** [10μM] incubated with (left) or without (right) *Escherichia coli* (ATCC 9637) [O.D_{600} 0.5] in PBS 7.4, 1% ACN, 37°C.

**Time [min]**	0.5	5	10	15	30
**Relative TLE (CB/AD)**	78.5	43.0	16.6	8.9	3.6

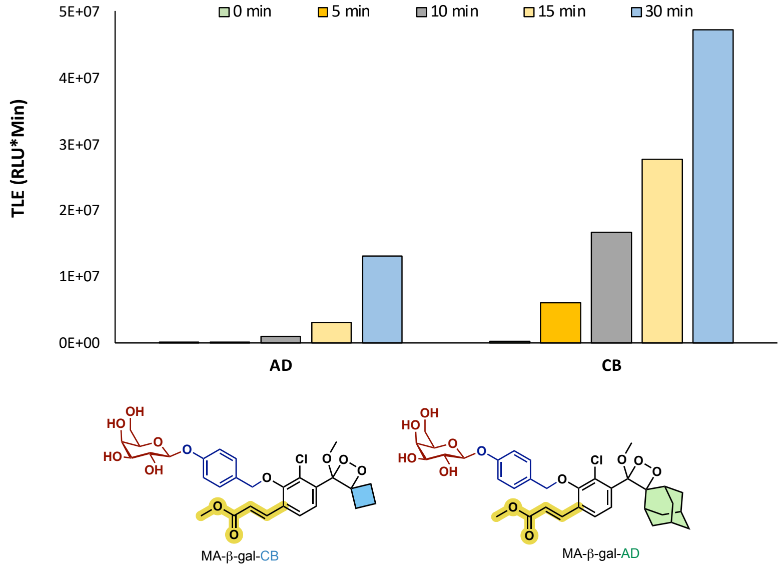

Figure **S58**: Total light emitted during 0.5, 5, 10, 15, and 30 minutes by **MA β-gal CB** and **MA β-gal AD** [10μM] incubated with *Escherichia coli* (ATCC 9637) [O.D_{600} 0.5] in PBS 7.4, 1% ACN, 37°C.

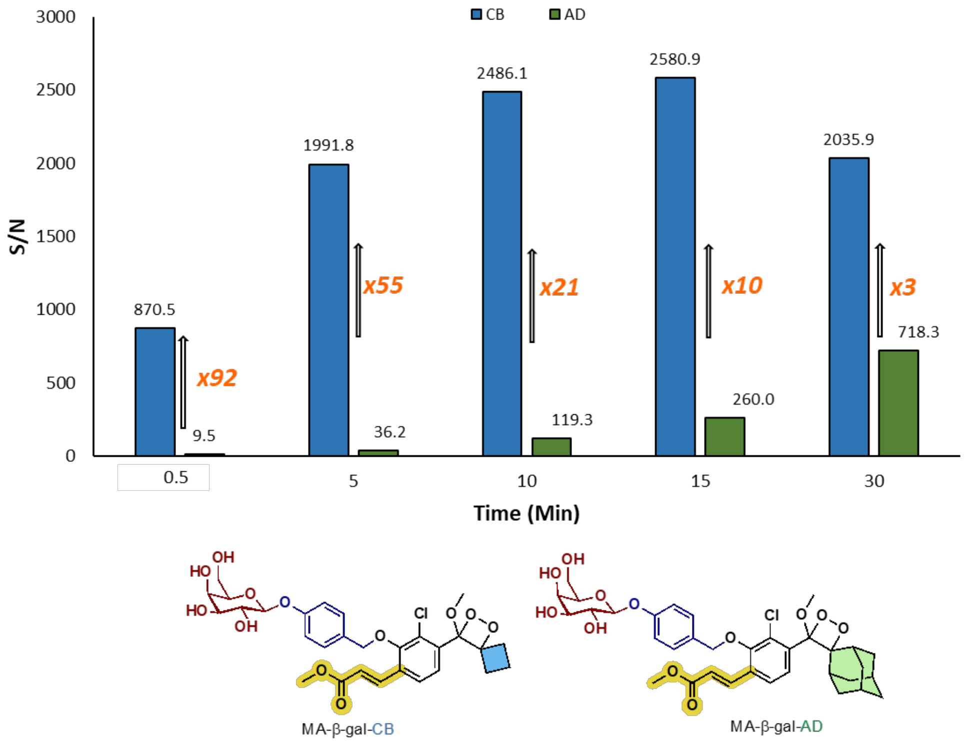

Figure **S59**: Signal-to-Noise ratio after 0.5, 5, 10, 15, and 30 minutes of **MA β-gal CB** and **MA β-gal AD** [10μM] incubated with *Escherichia coli* (ATCC 9637) [O.D_{600} 0.5] in PBS 7.4, 1% ACN, 37°C.

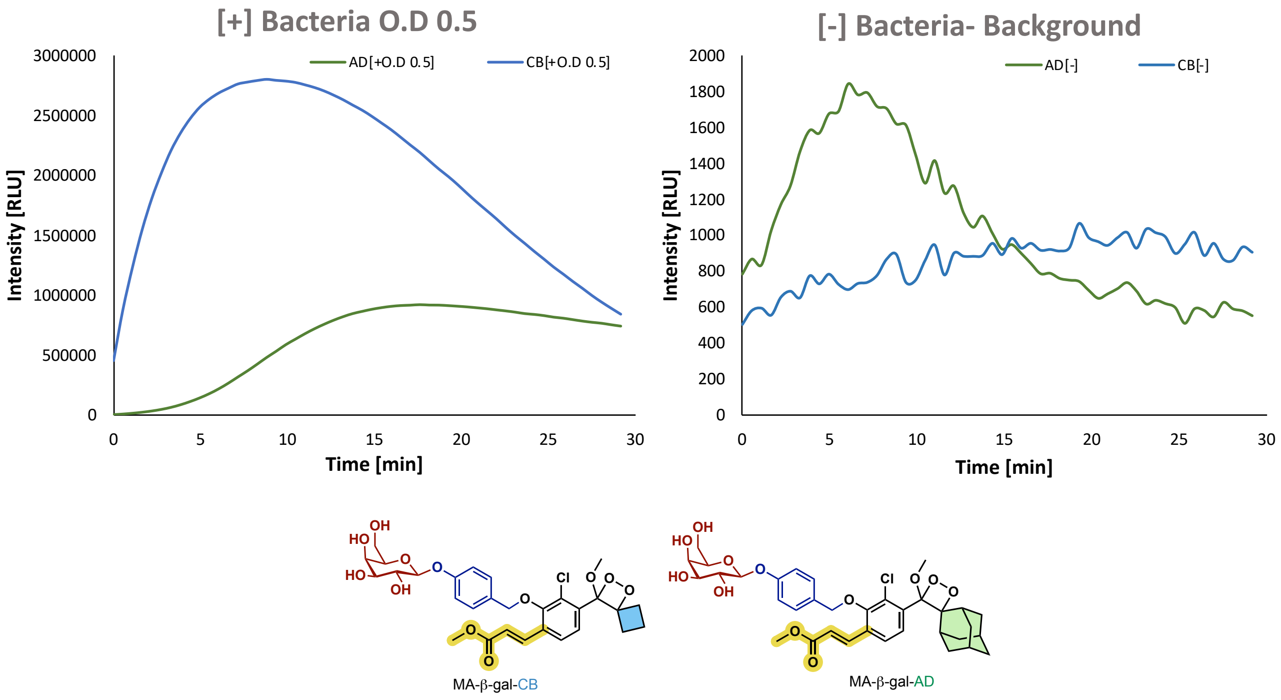

Figure **S60**: Chemiluminescent kinetic profiles of **MA β-gal CB** and **MA β-gal AD** [10μM] incubated with (left) or without (right) *Escherichia coli* (Clinical isolate) [O.D_{600} 0.5] in PBS 7.4, 1% ACN, 37°C.

.5

Time [min]	0	5	10	15	30
Relative TLE (CB/AD)	66.1	32.0	11.0	6.1	3.2

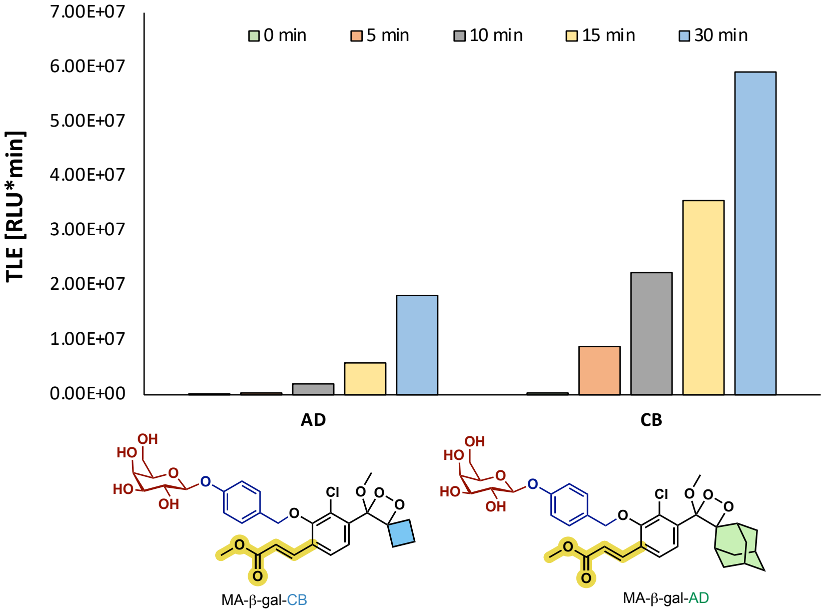

Figure **S61**: Total light emitted during 0.5, 5, 10, 15, and 30 minutes by **MA β-gal CB** and **MA β-gal AD** [10μM] incubated with *Escherichia coli* (Clinical isolate) [$O.D_{600}$ 0.5] in PBS 7.4, 1% ACN, 37°C.

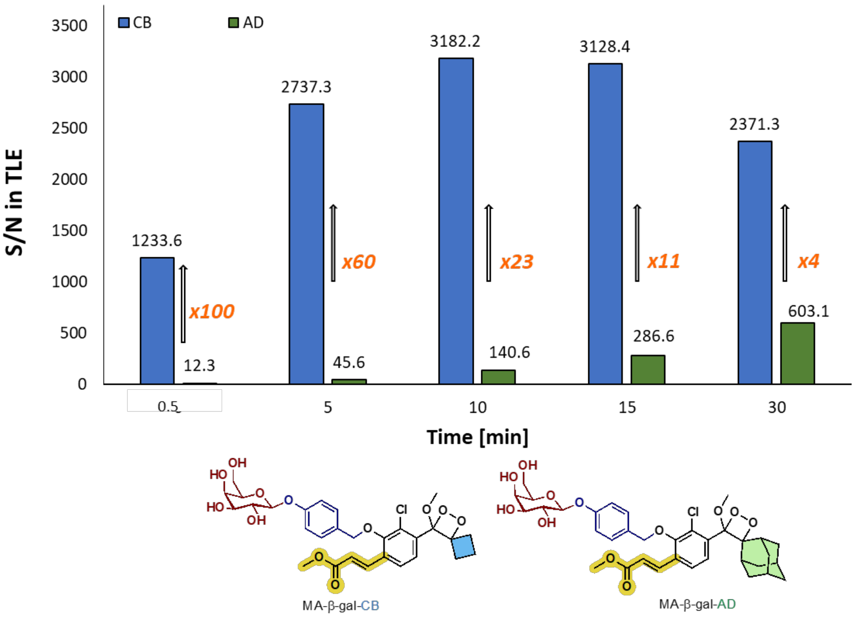

Figure **S62**: Signal-to-Noise ratio after 0.5, 5, 10, 15, and 30 minutes of **MA β-gal CB** and **MA β-gal AD** [10μM] incubated with *Escherichia coli* (Clinical isolate) [$O.D_{600}$ 0.5] in PBS 7.4, 1% ACN, 37°C.

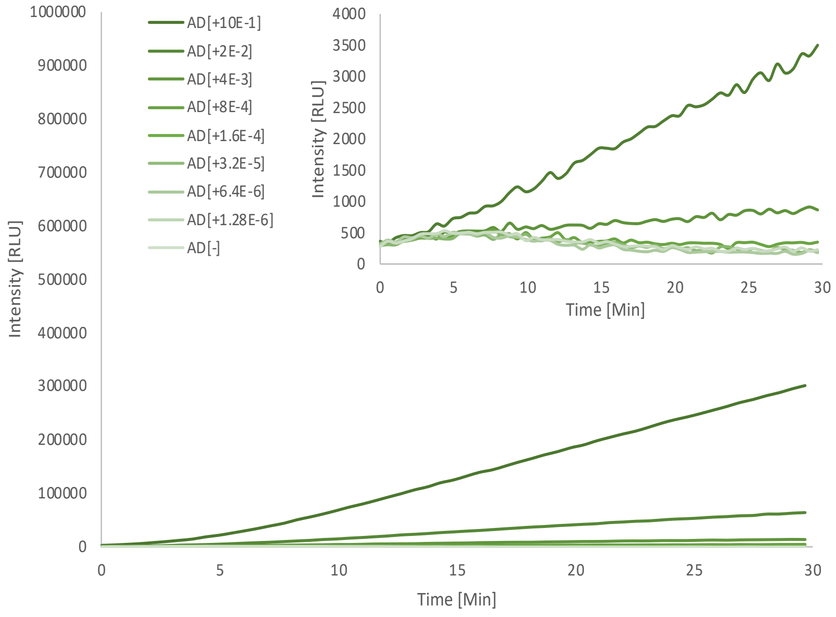

Figure **S63**: Chemiluminescent kinetic profiles of **MA β-gal AD** [10 μM] with varying concentrations of E-coli [$1.0*10^{-1}$ - $1.28*10^{-6}$] in PBS pH 7.4, 1% ACN, 37°C.

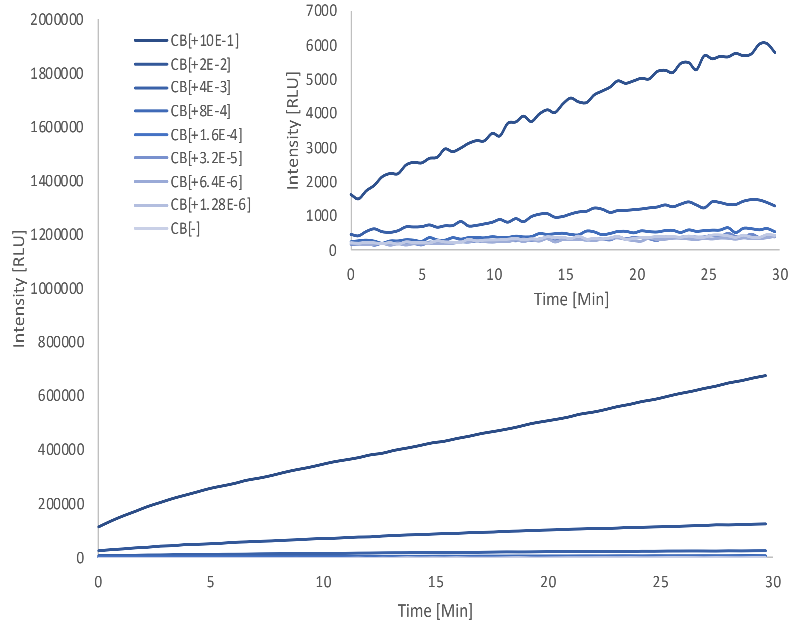

Figure **S64**: Chemiluminescent kinetic profiles of **MA β-gal CB** [10 μM] with varying concentrations of *Escherichia coli* (Clinical isolate) [$O.D_{600}$ $1.0*10^{-1}$ - $1.28*10^{-6}$] in PBS pH 7.4, 1% ACN, 37°C.

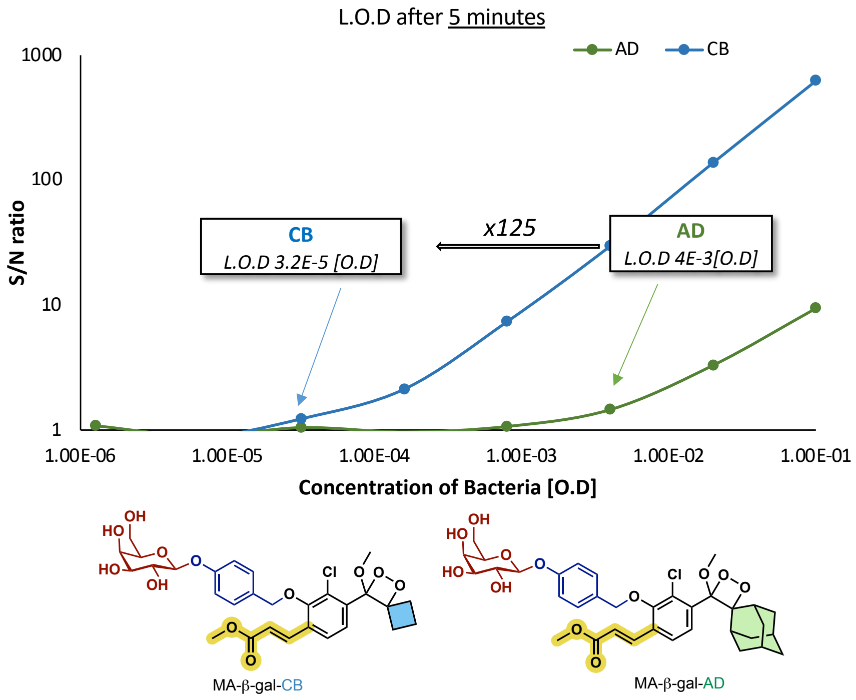

Figure **S65**: Signal-to-Noise ratio after **5 minutes** of **MA β-gal CB** and **MA β-gal AD** [10μM] incubated with varying concentrations of *Escherichia coli* (Clinical isolate) [$O.D_{600}$ 0.1 - 1.0*10^{-6}] in PBS 7.4, 1% ACN, 37°C.

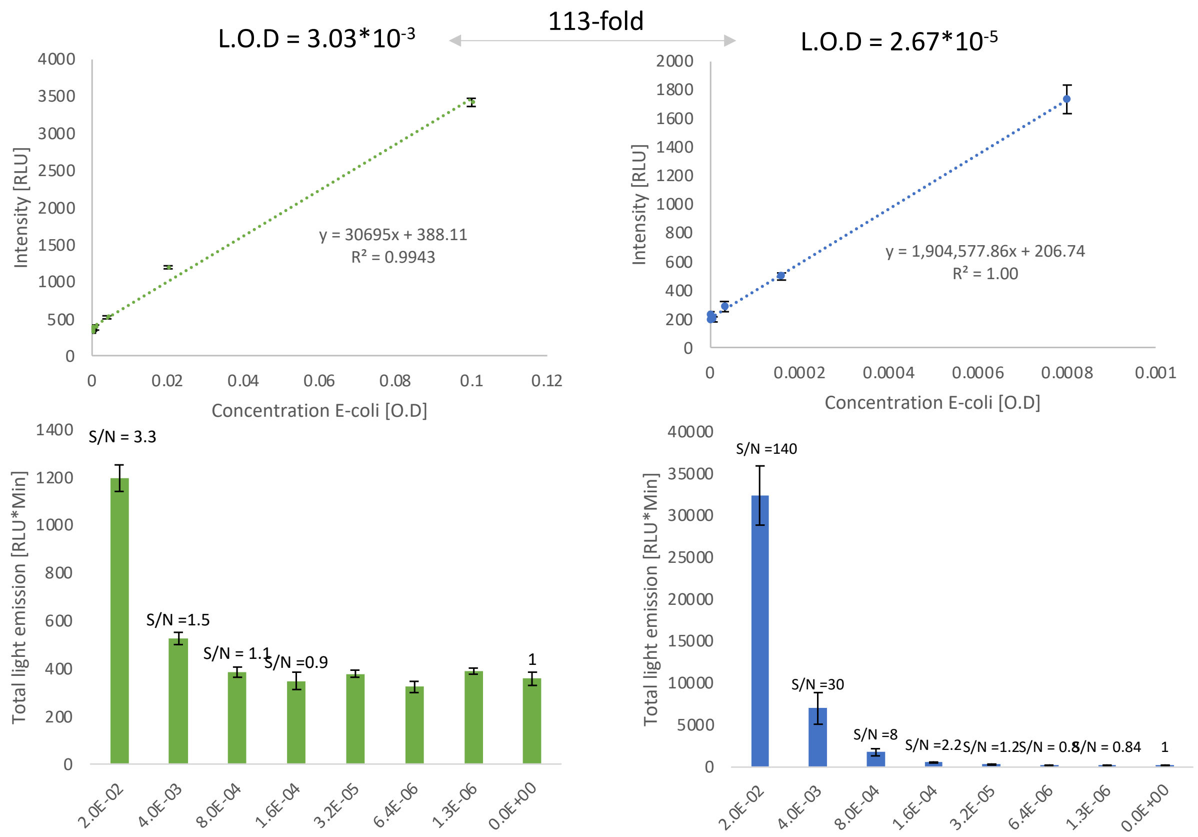

Figure **S66**: linear calibration curve and the total light emission after **5 minutes** of **MA β-gal AD** (left) and **MA β-gal CB** (right)[10μM] with varying concentrations of *Escherichia coli* (Clinical isolate) [$O.D_{600}$ 0.1 - 1.0*10^{-6}] in PBS 7.4, 1% ACN, 37°C. The limit of detection was determined as 3*(S.D. of the blank) divided by the slope of the linear calibration curve (L.O.D = 3σ/k). Error bars are S.D. from three independent experiments.

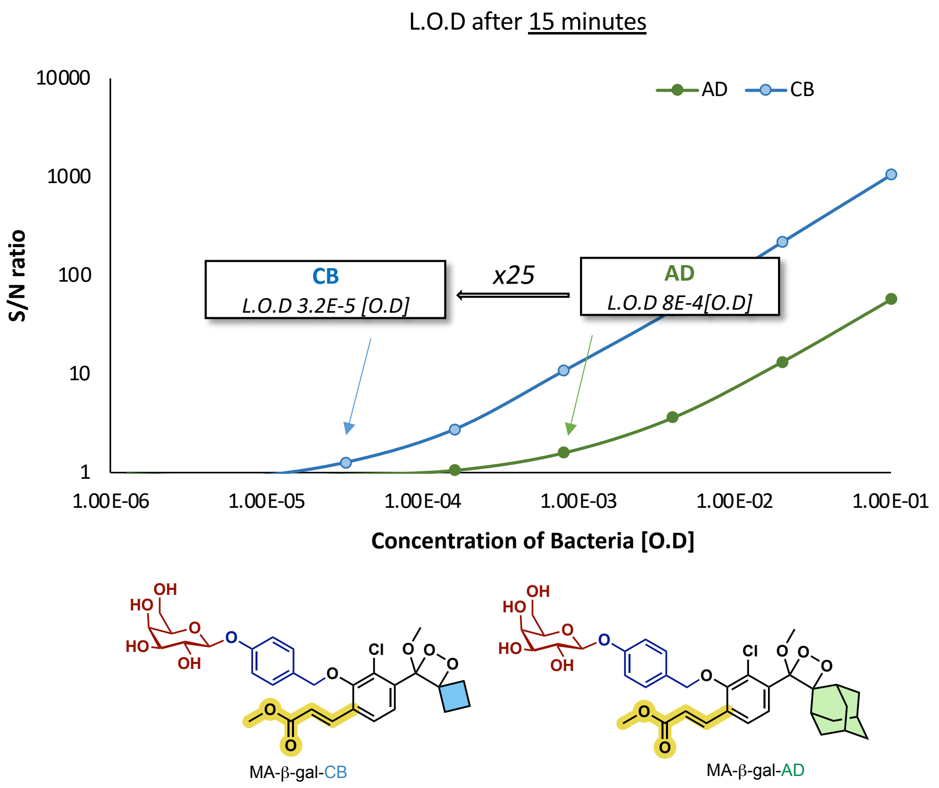

Figure **S67**: Signal-to-Noise ratio after **15 minutes** of **MA β-gal CB** and **MA β-gal AD** [10μM] incubated with varying concentrations of *Escherichia coli* (Clinical isolate) [O.D_{600} 0.1 - 1.0*10^{-6}] in PBS 7.4, 1% ACN, 37°C.

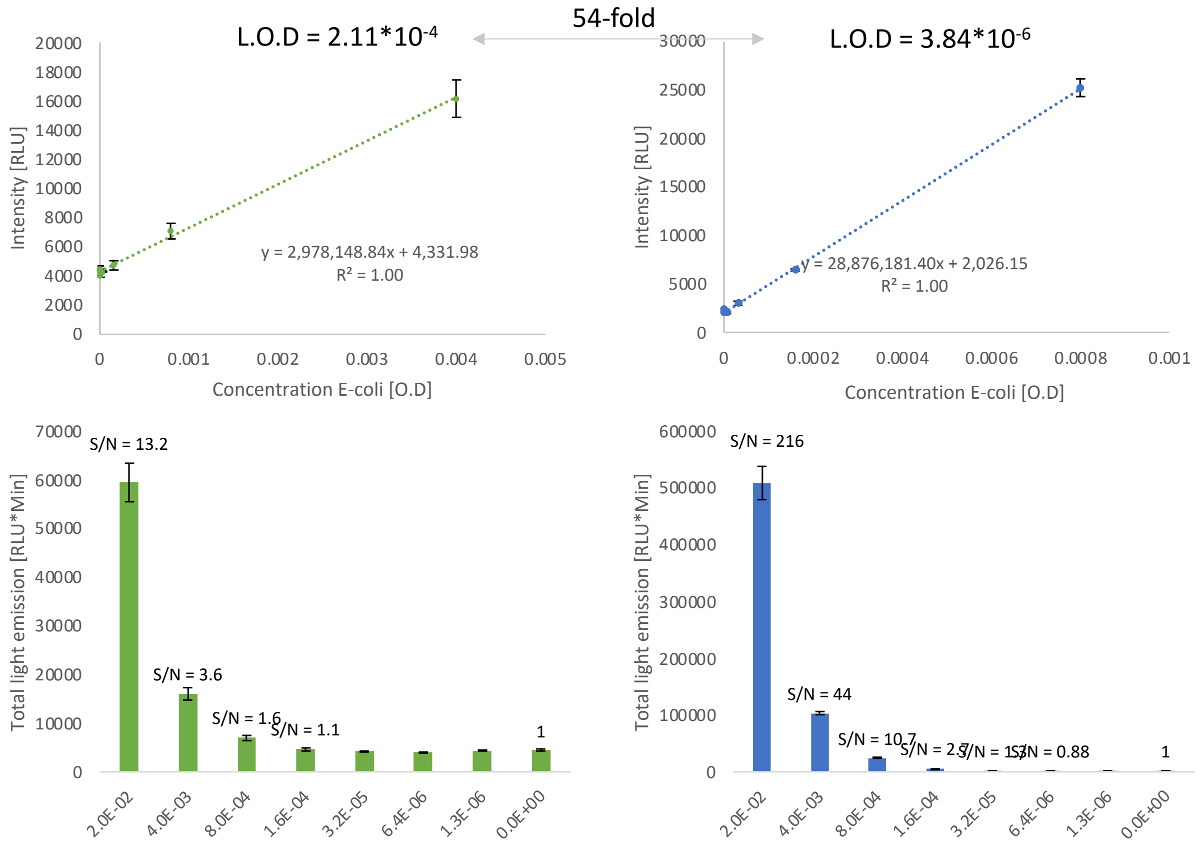

Figure **S68**: linear calibration curve and the total light emission after **15 minutes** of **MA β-gal AD** (left) and **MA β-gal CB** (right)[10μM] with varying concentrations of *Escherichia coli* (Clinical isolate) [$O.D_{600}$ 0.1 - $1.0*10^{-6}$] in PBS 7.4, 1% ACN, 37°C.

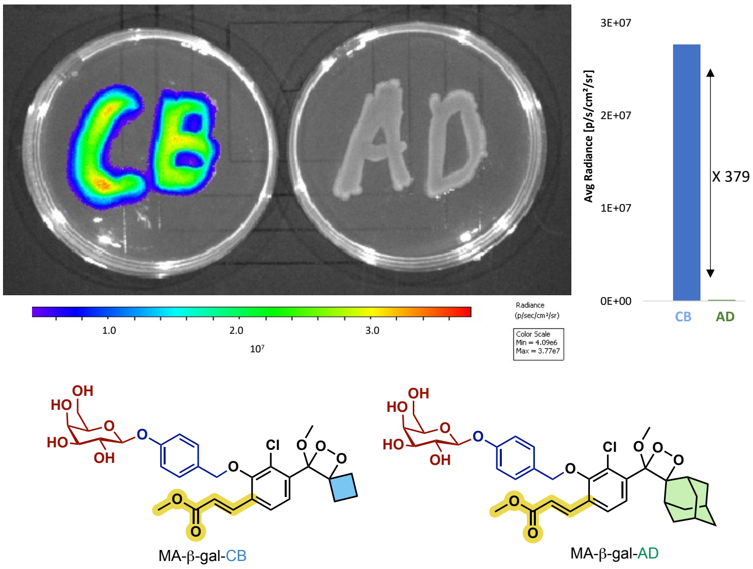

Figure **S69**: Chemiluminescence imaging using IVIS® Lumina for **MA β-gal CB** and **MA β-gal AD** [100μM] after 30 sec of incubation with *E.coli (Clinical isolate) on LB agar,* 1% ACN,37°C. Exposure time 30 sec.

pH	logD
1.50	3.35
5.00	3.35
6.50	3.35
7.40	3.35

pH	logD
0.00	3.35
0.50	3.35
1.00	3.35
1.50	3.35
2.00	3.35
2.50	3.35
3.00	3.35
3.50	3.35
4.00	3.35
4.50	3.35
5.00	3.35
5.50	3.35
6.00	3.35
6.50	3.35
7.00	3.35
7.50	3.35
8.00	3.35
8.50	3.35
9.00	3.35
9.50	3.35
10.00	3.35
10.50	3.34
11.00	3.32
11.50	3.27
12.00	3.13
12.50	2.82
13.00	2.29
13.50	1.54
14.00	0.57

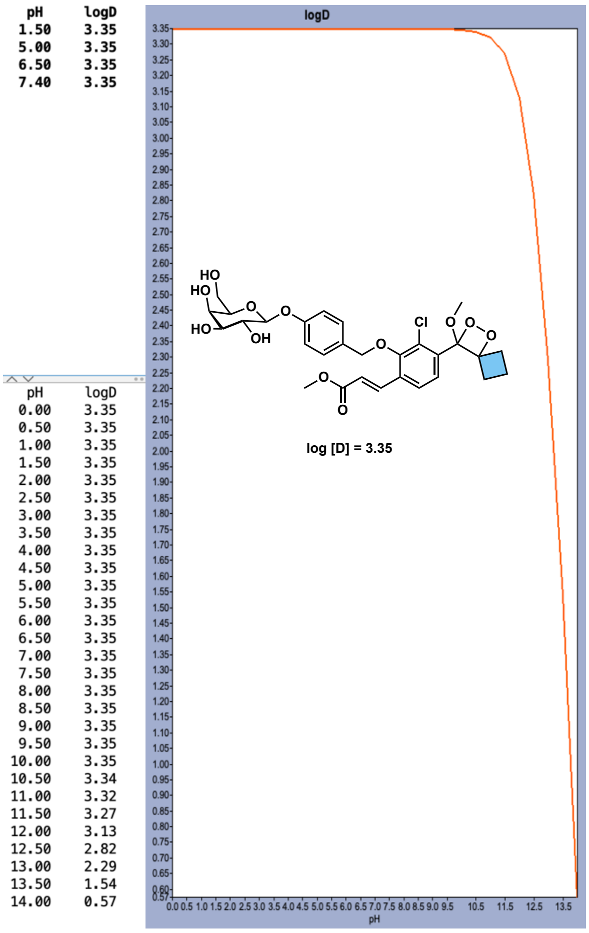

pH	logD
1.50	4.62
5.00	4.62
6.50	4.62
7.40	4.62

pH	logD
0.00	4.62
0.50	4.62
1.00	4.62
1.50	4.62
2.00	4.62
2.50	4.62
3.00	4.62
3.50	4.62
4.00	4.62
4.50	4.62
5.00	4.62
5.50	4.62
6.00	4.62
6.50	4.62
7.00	4.62
7.50	4.62
8.00	4.62
8.50	4.62
9.00	4.62
9.50	4.61
10.00	4.61
10.50	4.61
11.00	4.59
11.50	4.54
12.00	4.39
12.50	4.08
13.00	3.56
13.50	2.80
14.00	1.84

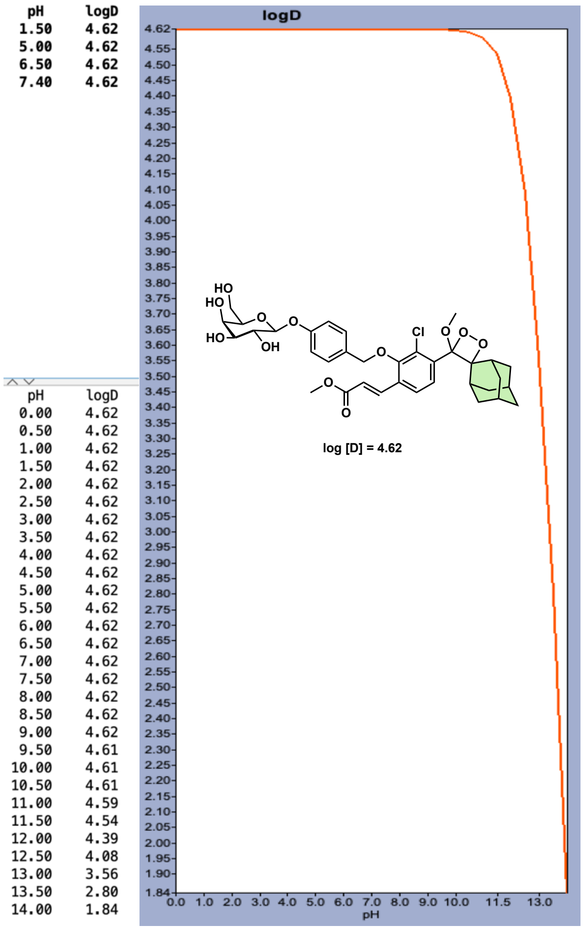

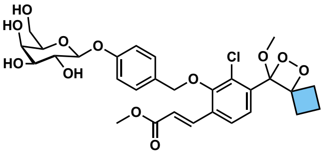

log [D] $_{pH\ 7.4}$ = 3.35

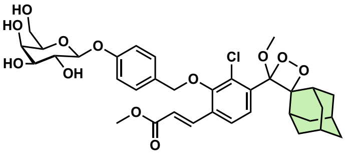

log [D] $_{pH\ 7.4}$ = 4.62

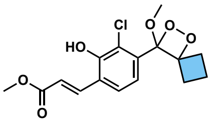

log [D] $_{pH\ 7.4}$ = 3.95

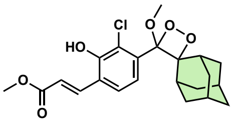

log [D] $_{pH\ 7.4}$ = 5.21

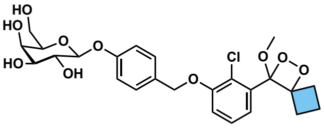

log [D] $_{pH\ 7.4}$ = 2.81

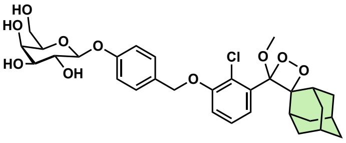

log [D] $_{pH\ 7.4}$ = 4.07

Figure **S70:** Calculated logD results of the cyclobutyl (CB) and adamantly (AD) derivatives. The distribution coefficient (logD) was calculated using MarvinSketch (version 6.3.1) with default parameters and with an electrolyte concentration of 0.1 M NaCl, pH 7.4.

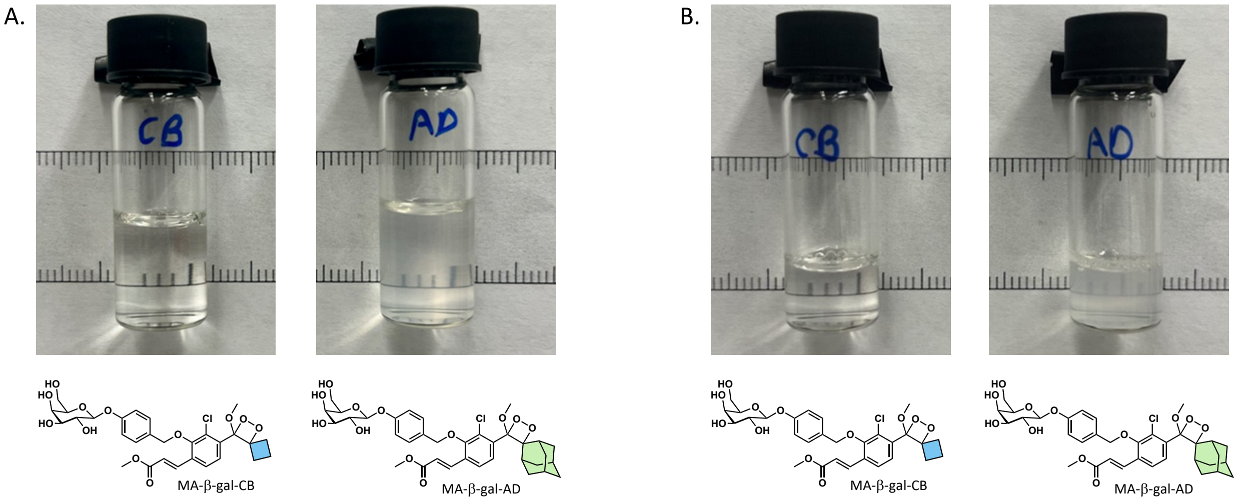

Figure **S71:** Visual comparison between MA-β-gal-CB and MA-β-gal-AD solubility in aqueous conditions. A. Stock solutions of the probes were diluted in PBS, pH 7.4 to a final concentration of 150μM (1.5% ACN as cosolvent). B. Stock solutions of the probes were diluted in PBS, pH 7.4 to a final concentration of 300μM (3% ACN as cosolvent).

Determination of aqueous solubility of MA-β-gal-CB and MA-β-gal-AD

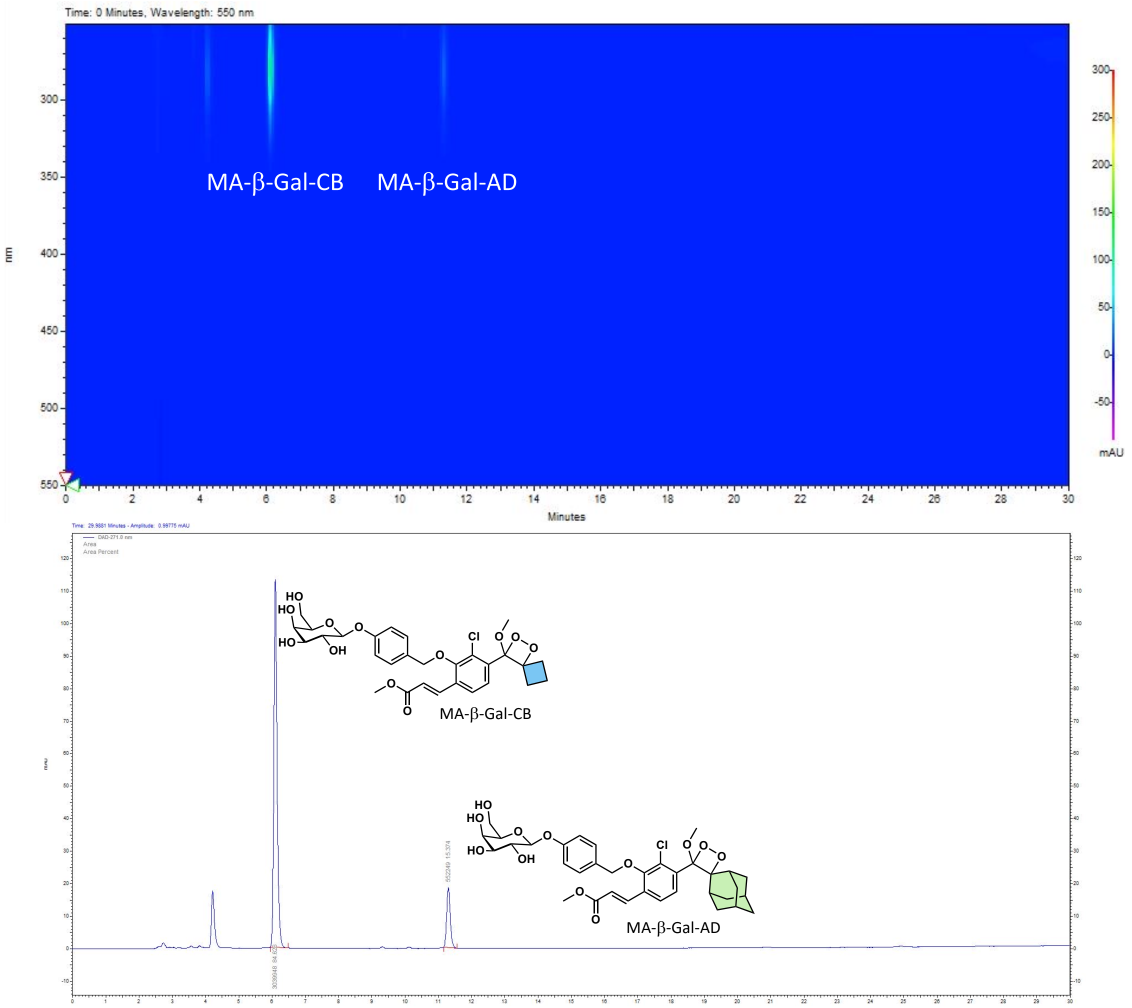

Figure **S72:** The solubility ratio in aqueous conditions between MA-β-gal-CB and MA-β-gal-AD was measured using HPLC with UV detection. Stock solutions of the probes were diluted in PBS, pH 7.4 to a final concentration of 300μM (3% ACN as cosolvent). The diluted solutions were vortexed and allowed to stand in dark at room temperature for 10 minutes. Then, the solutions were filtered to remove the precipitated compounds. HPLC analysis was conducted for the filtered solutions and the ratio between the dioxetanes was evaluated at 280 nm by calculating the area under each peak. The solubility ratio obtained of MA-β-gal-CB and MA-β-gal-AD is 6:1, respectively.

Absolut solubility values of >187μg/mL for MA-β-gal-CB and 35 μg/mL for MA-β-gal-AD.

Appendix III-NMR and MS Spectra

Cyclooctyl enolether (Compound 1a)

^{1}H-NMR

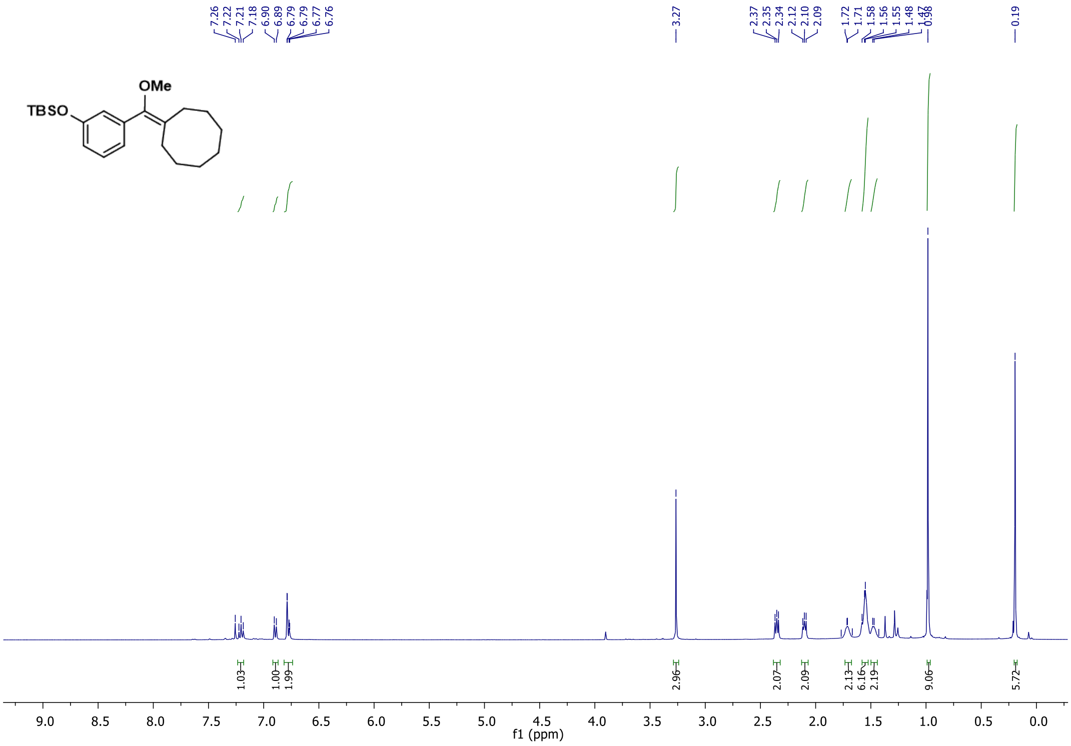

^{13}C-NMR

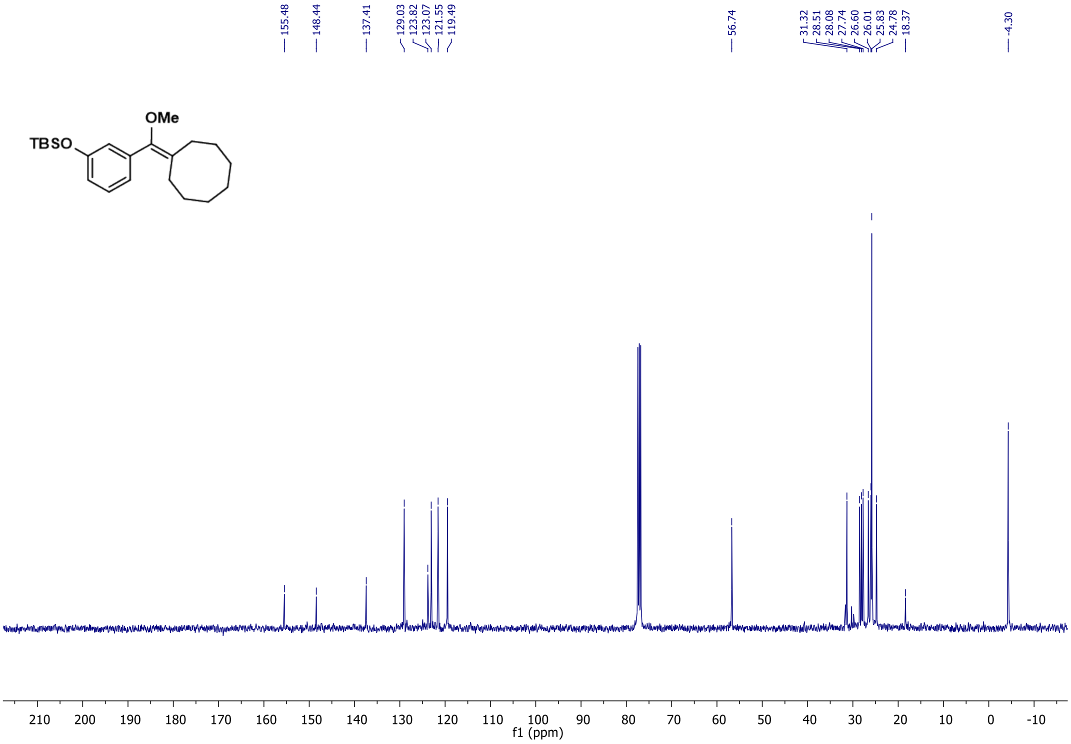

3D HPLC spectra (Compound 1a) (90-100% ACN in water, 0.1%TFA)

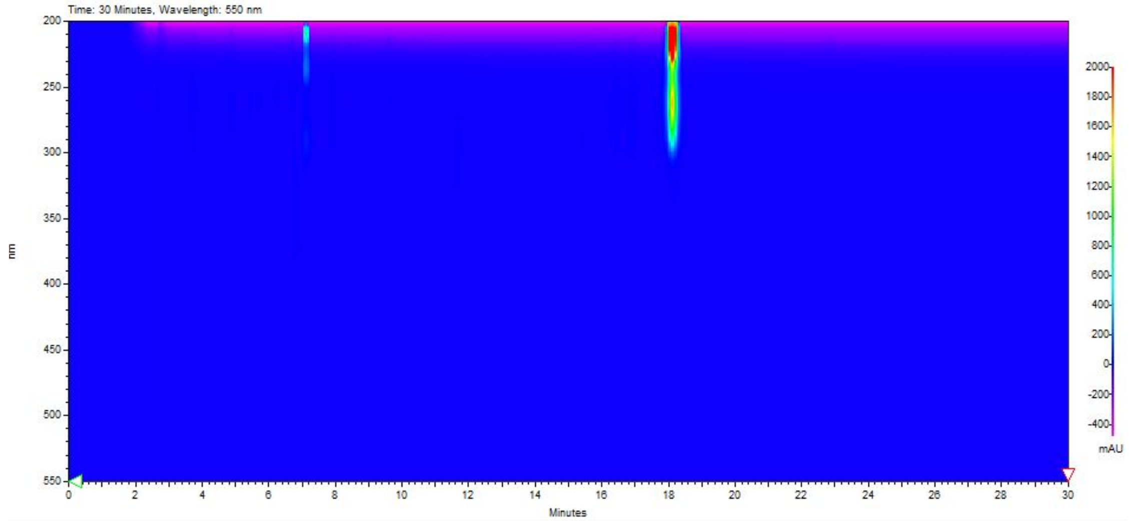

2D HPLC spectra (Compound 1a) (Absorbance measured at 275nm)

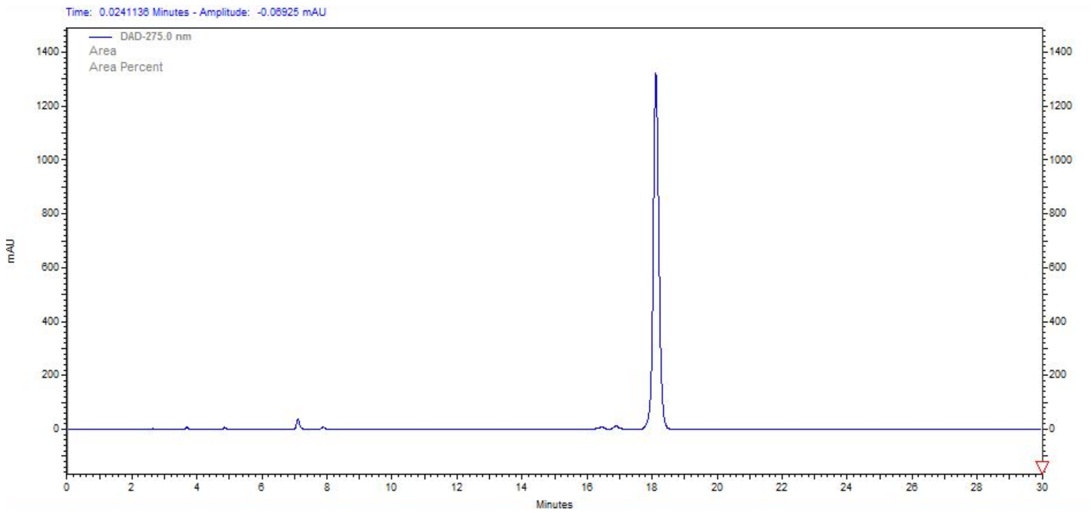

Cycloheptyl enolether (Compound 2a)

^{1}H-NMR

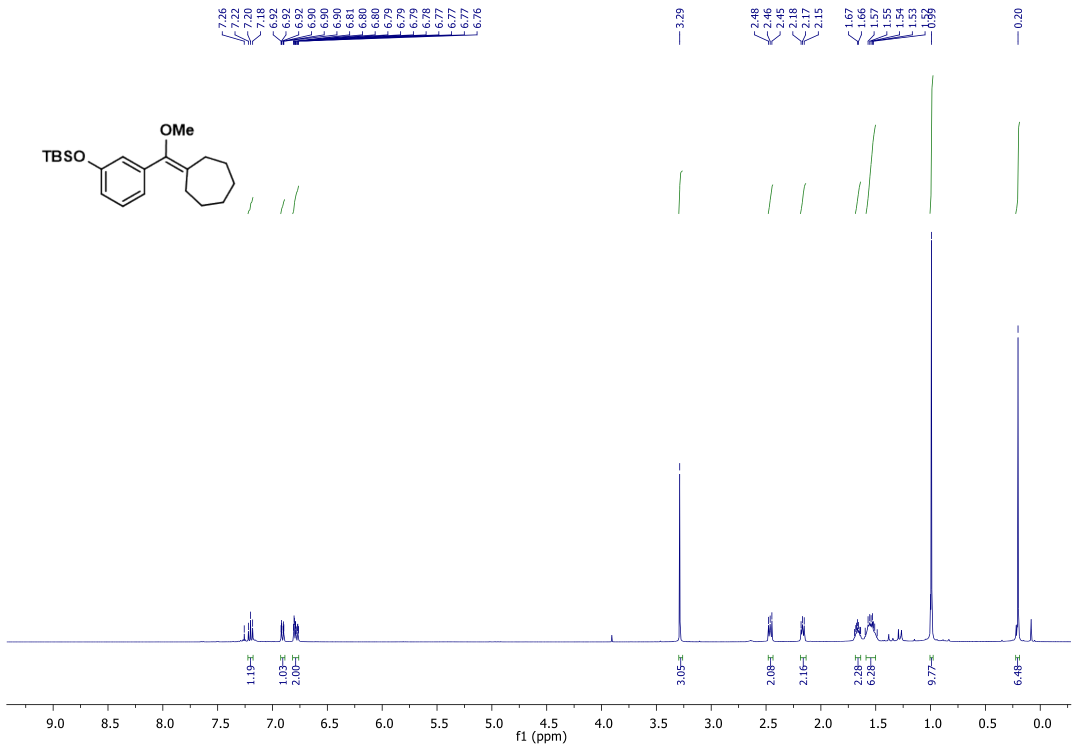

^{13}C-NMR

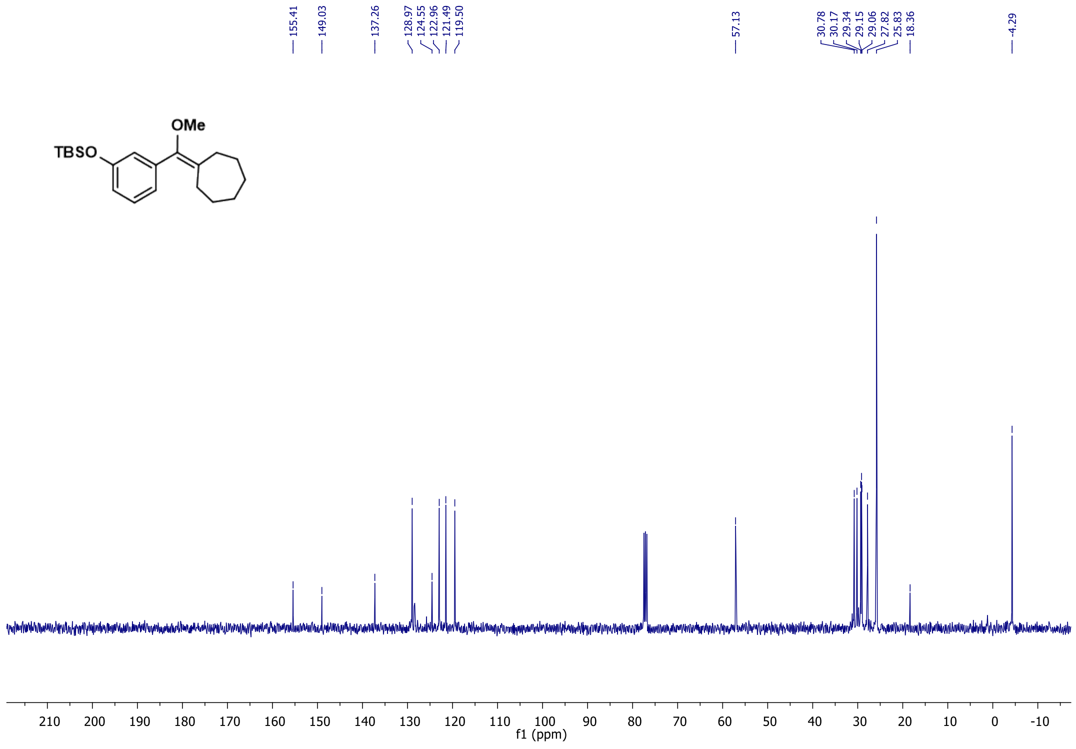

3D HPLC spectra (90-100% ACN in water, 0.1%TFA)

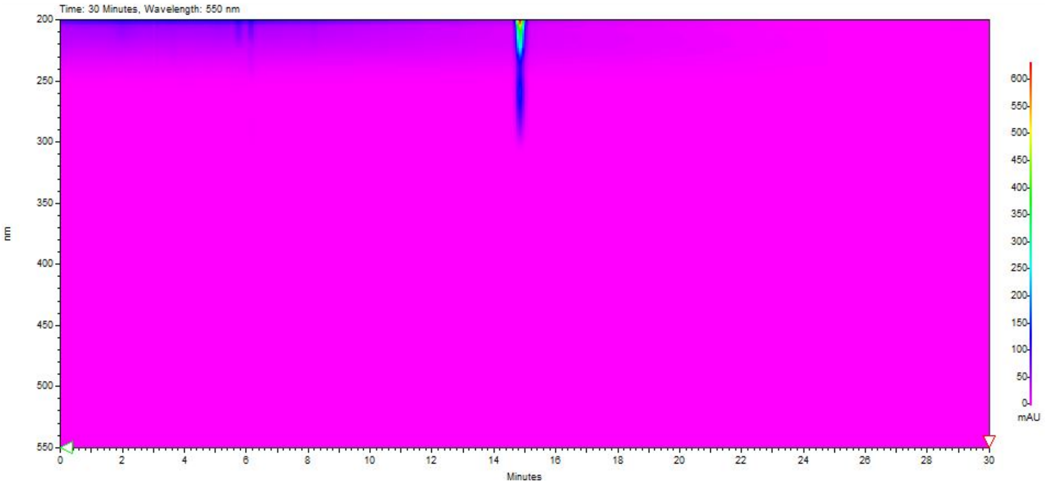

2D HPLC spectra (Absorbance measured at 275nm)

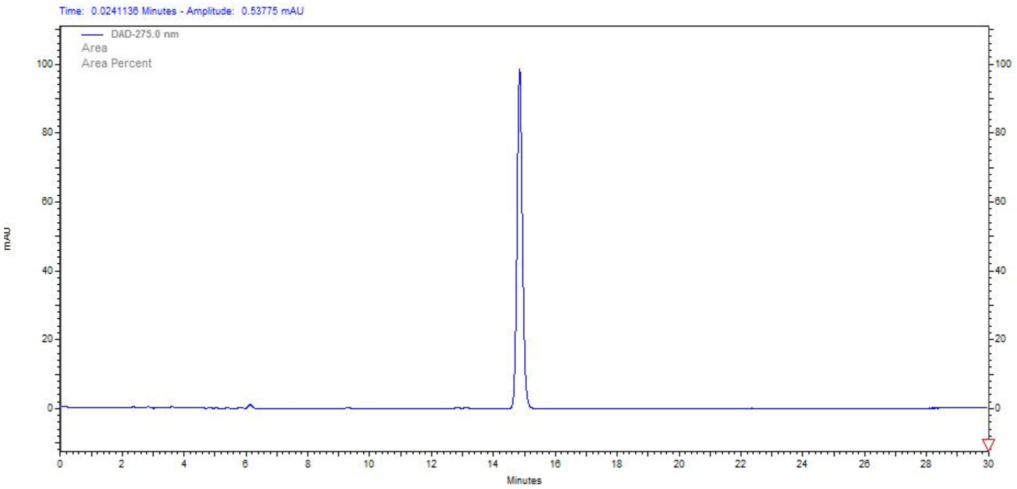

Mass spectra

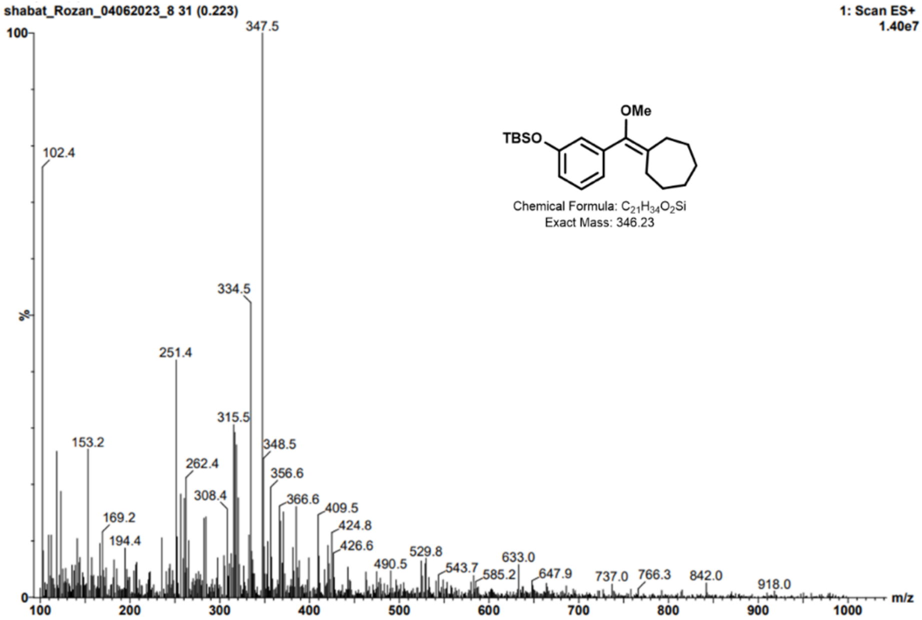

Ene-product 1

^{1}H-NMR

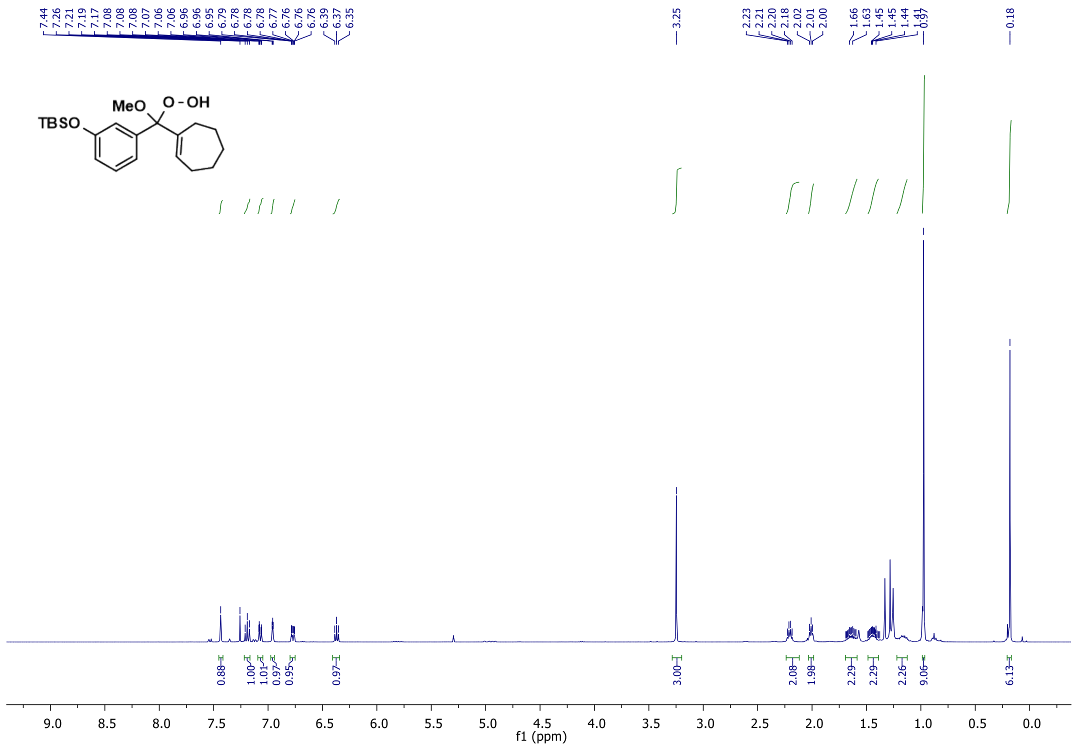

^{13}C-NMR

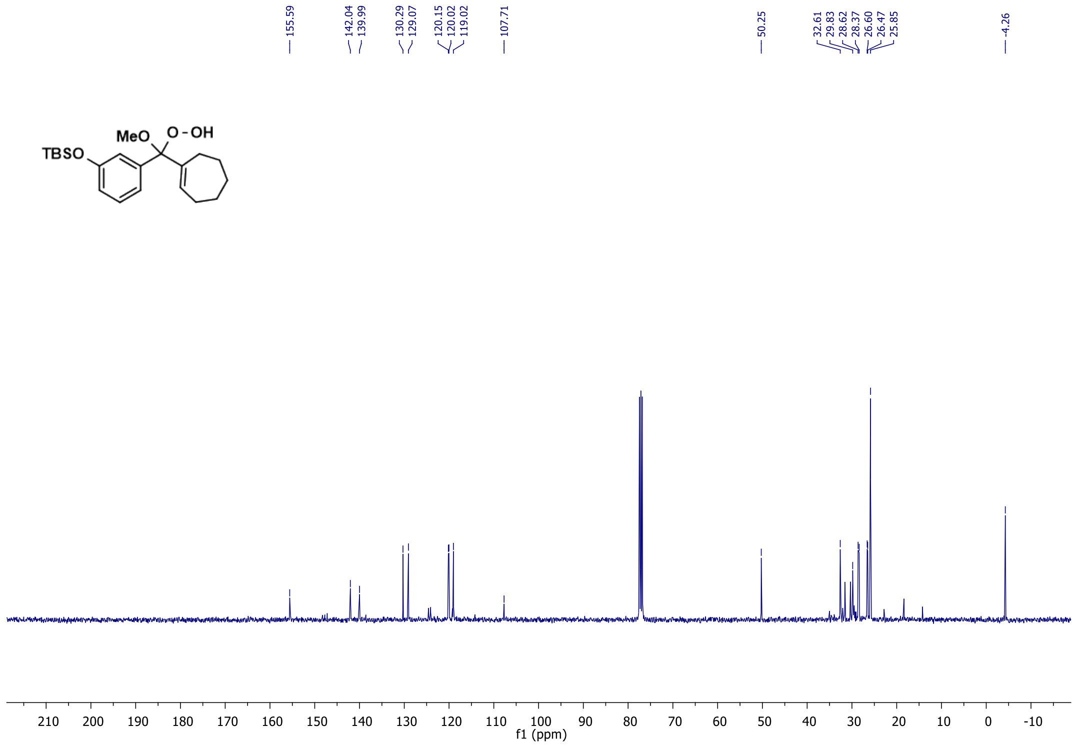

3D HPLC spectra (90-100% ACN in water, 0.1%TFA)

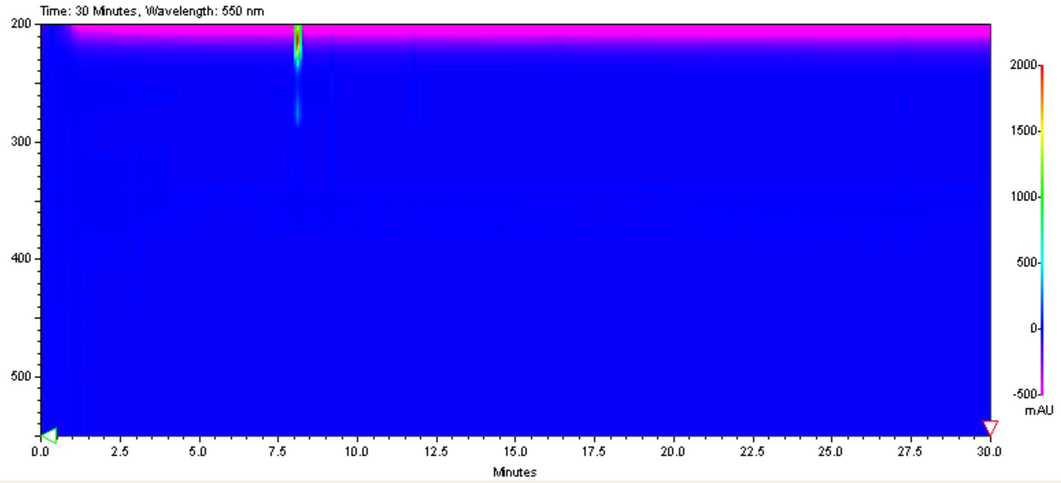

2D HPLC spectra (Absorbance measured at 275nm)

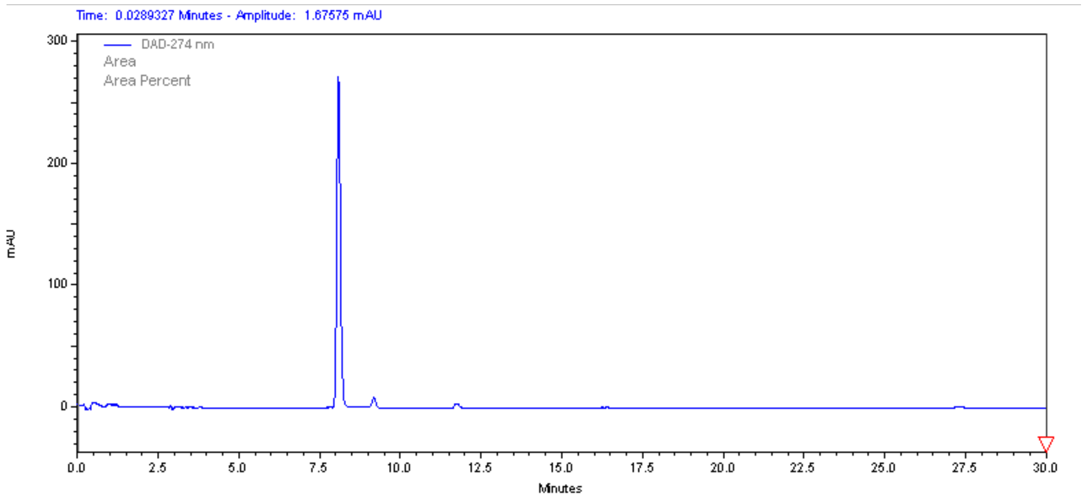

Cyclohexyl enolether (Compound 3a)

^{1}H-NMR

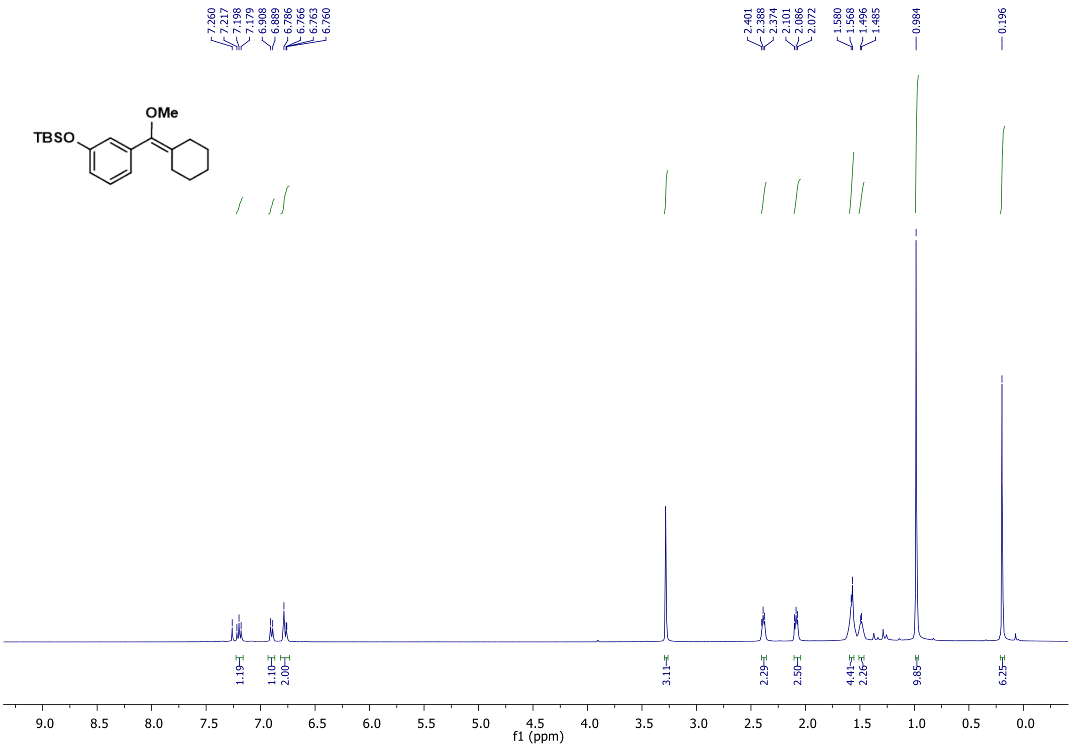

^{13}C-NMR

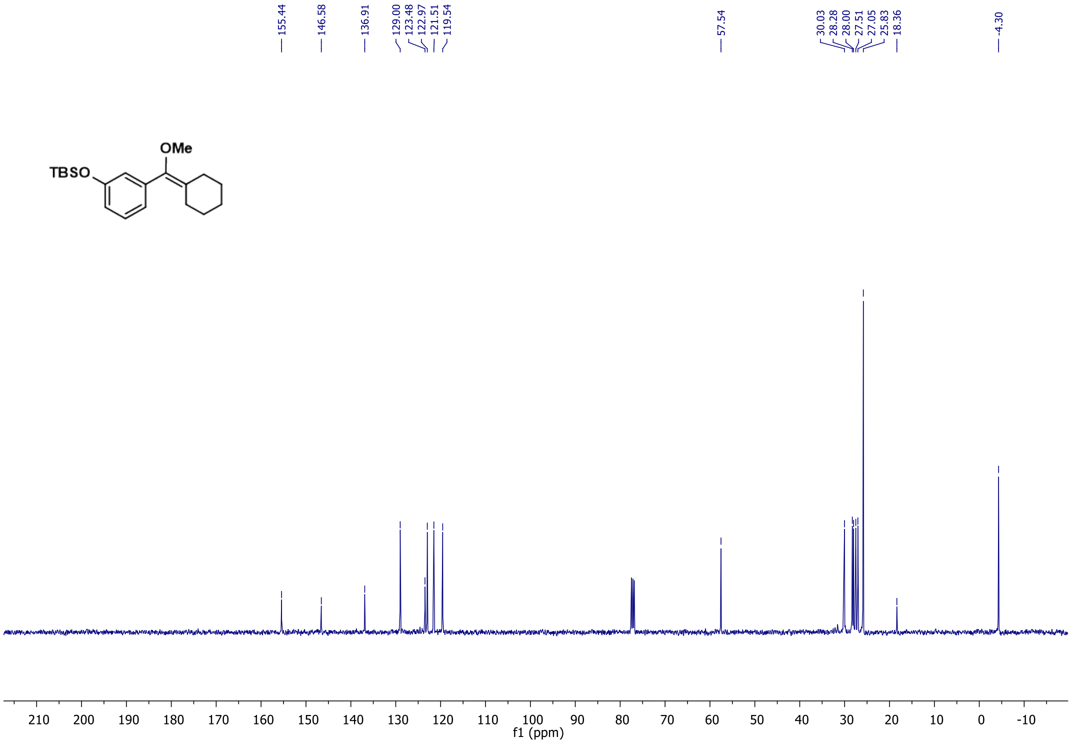

3D HPLC spectra (90-100% ACN in water, 0.1%TFA)

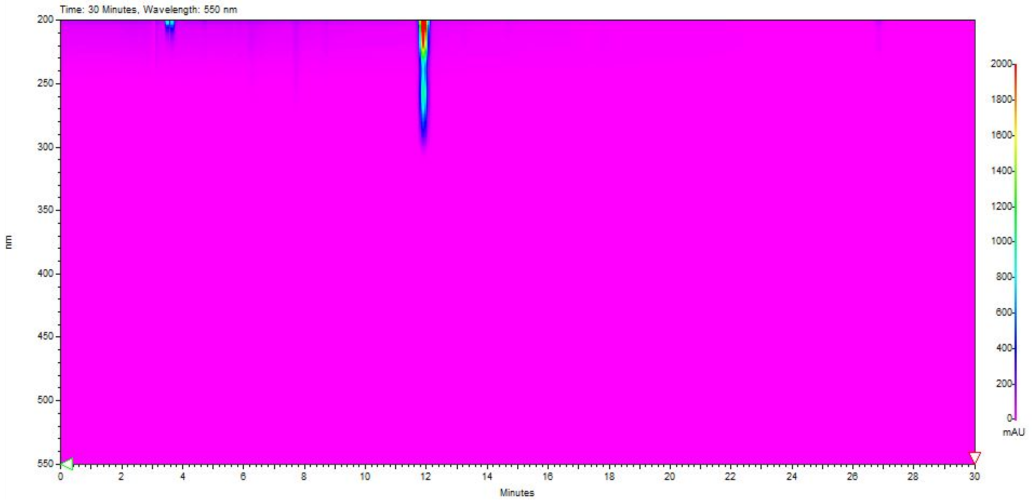

2D HPLC spectra (Absorbance measured at 275nm)

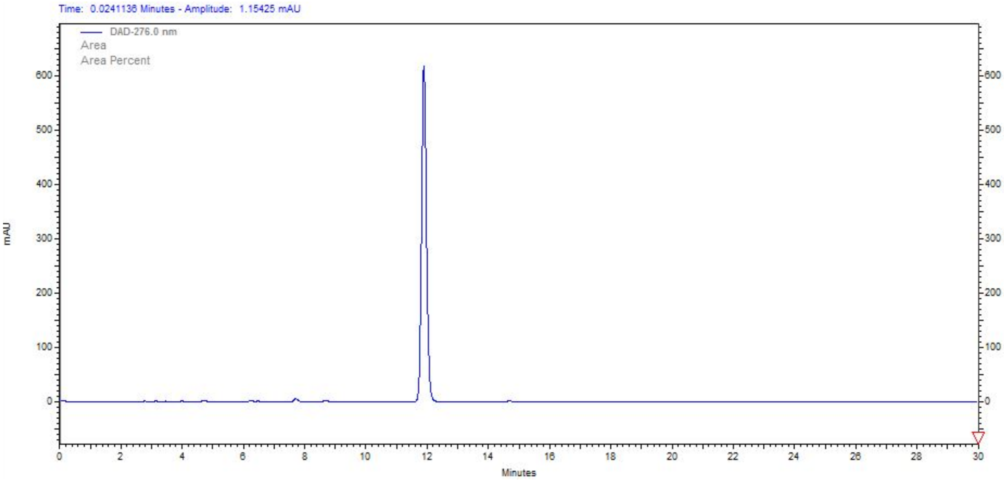

Mass spectra

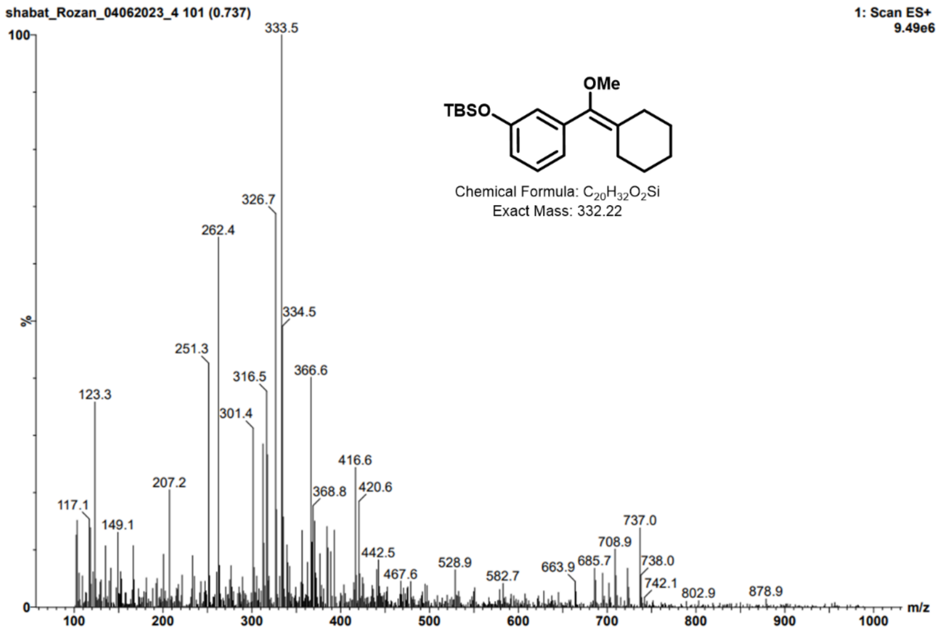

Diox 2

^{1}H-NMR

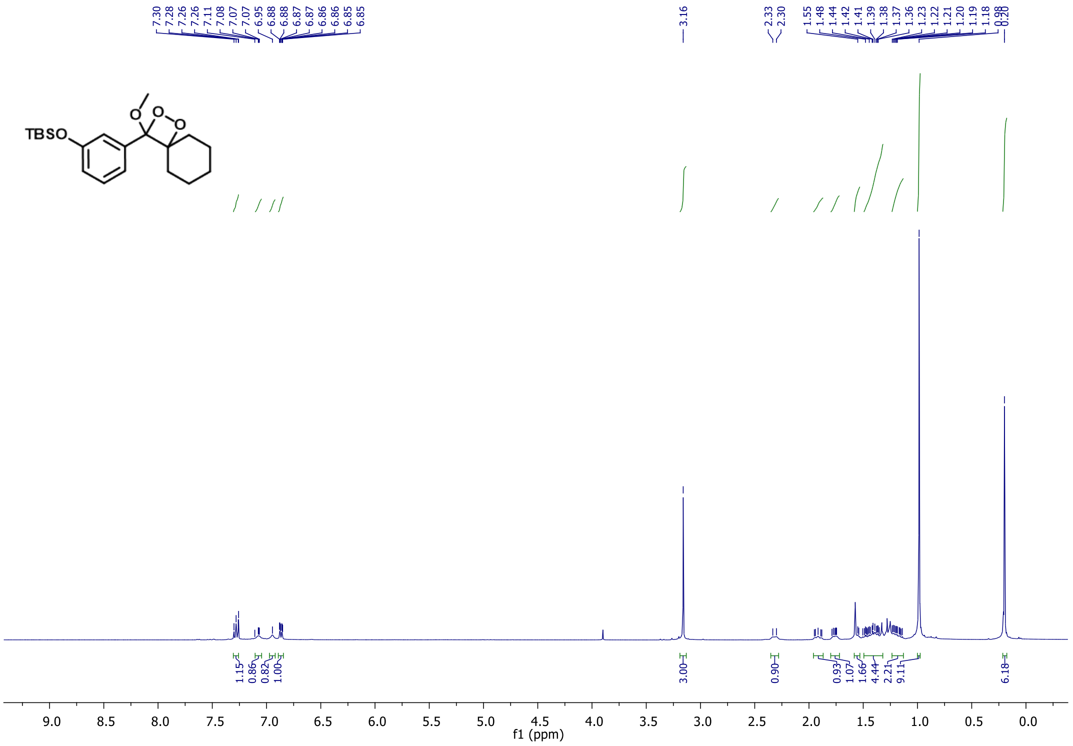

^{13}C-NMR

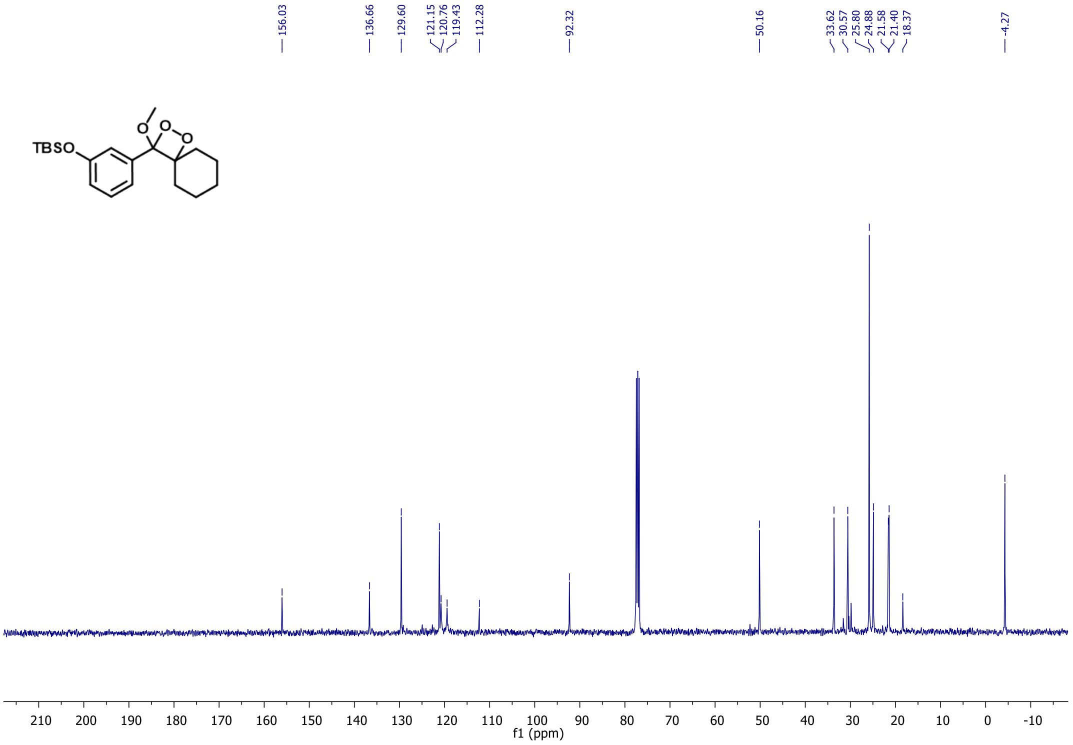

3D HPLC spectra (90-100% ACN in water, 0.1%TFA)

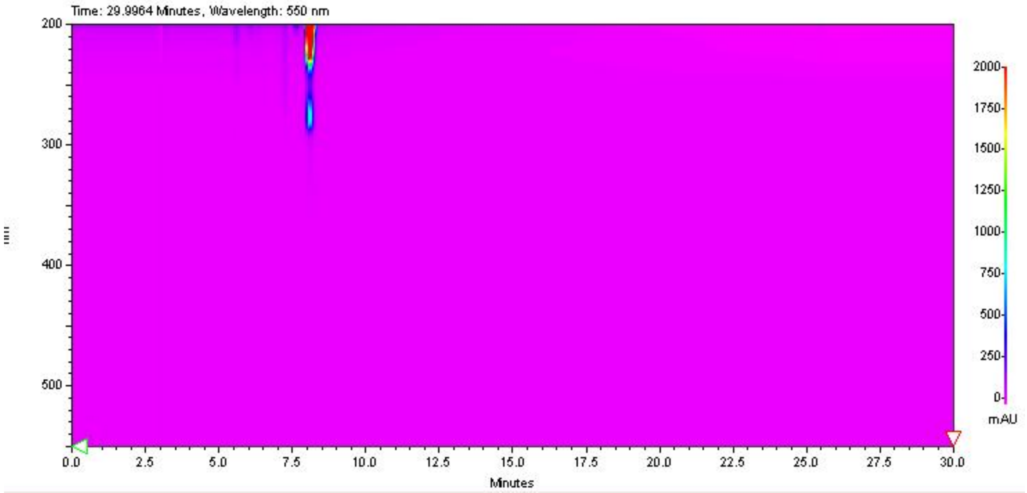

2D HPLC spectra (Absorbance measured at 275nm)

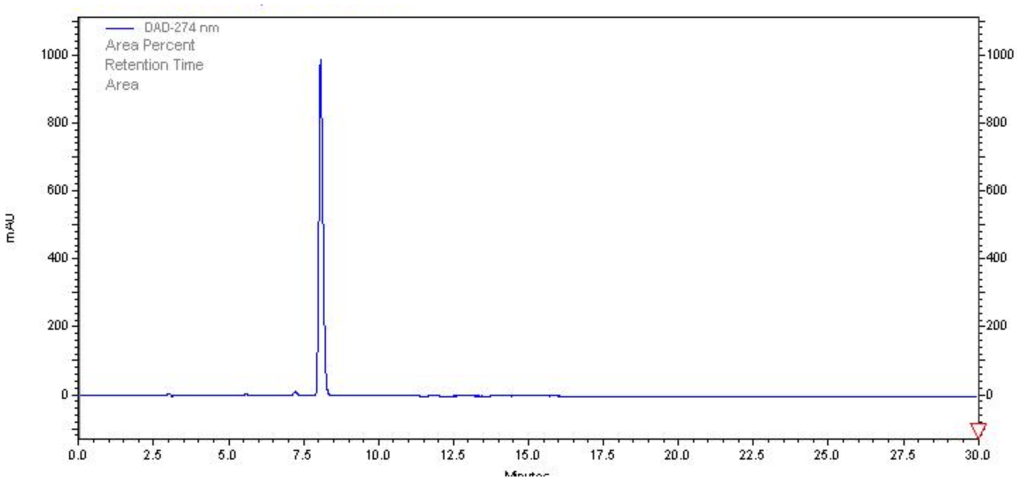

Ene-product 2

^{1}H-NMR

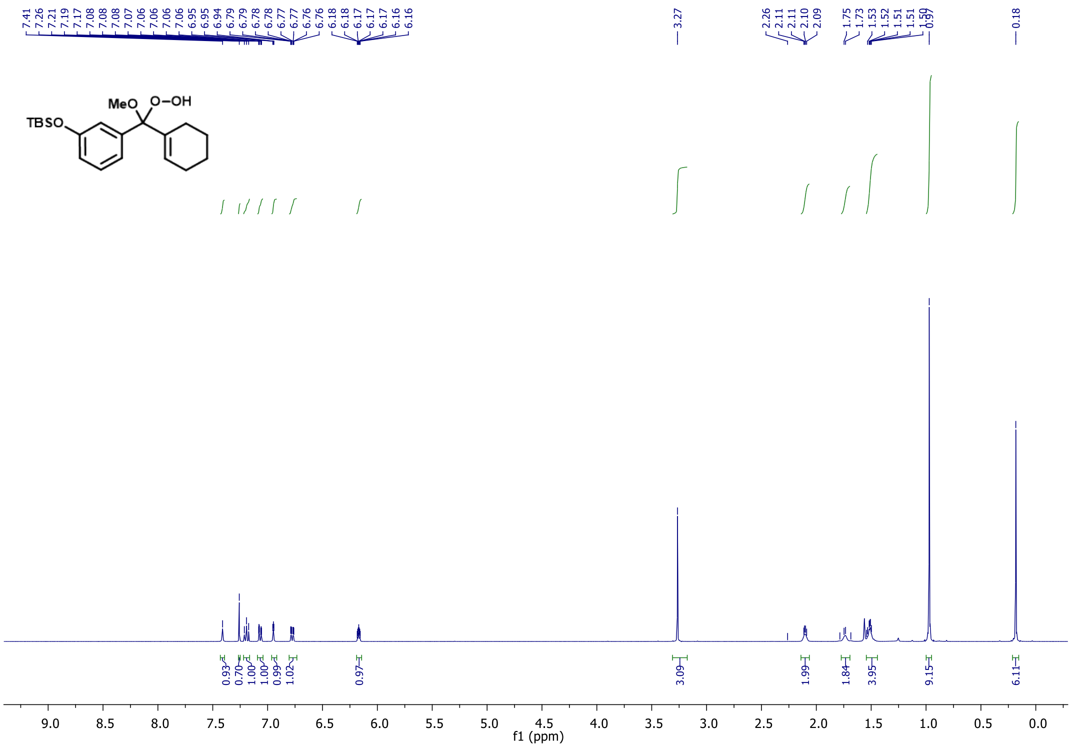

^{13}C-NMR

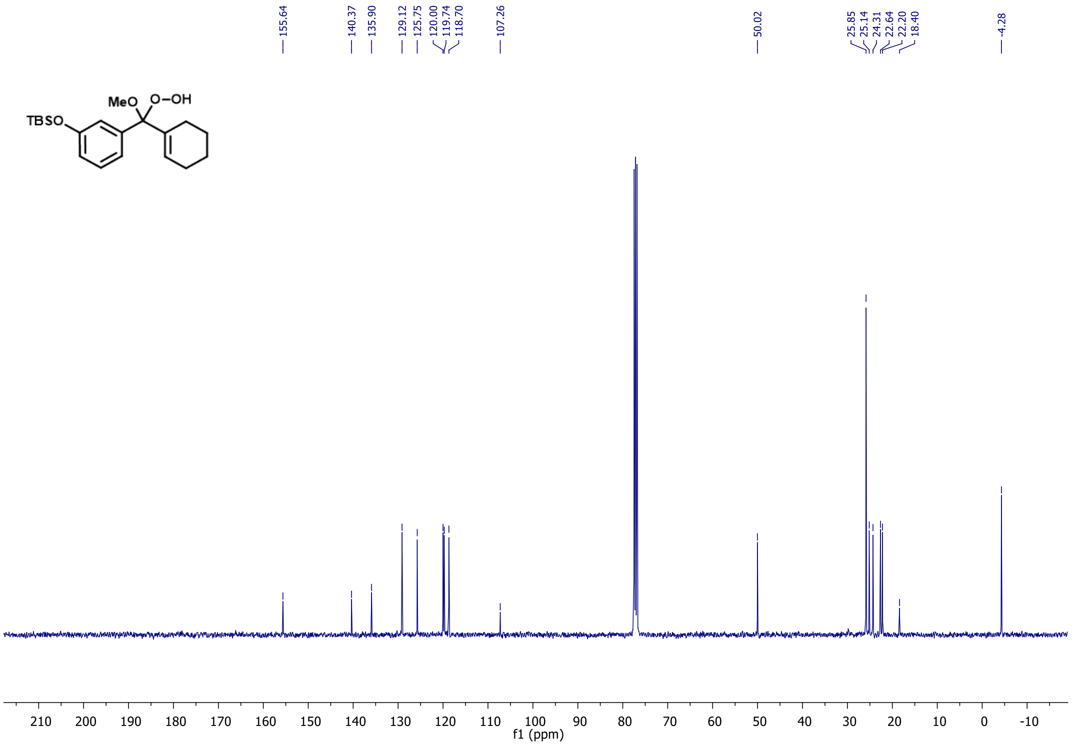

3D HPLC spectra (90-100% ACN in water, 0.1%TFA)

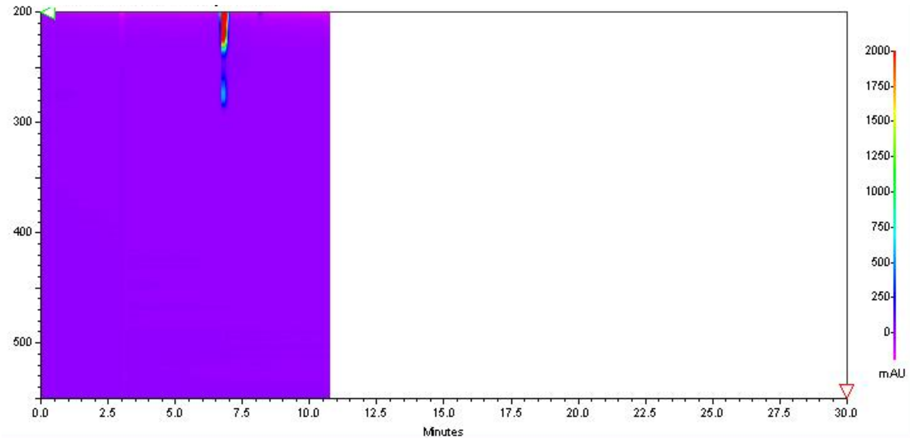

2D HPLC spectra (Absorbance measured at 275nm)

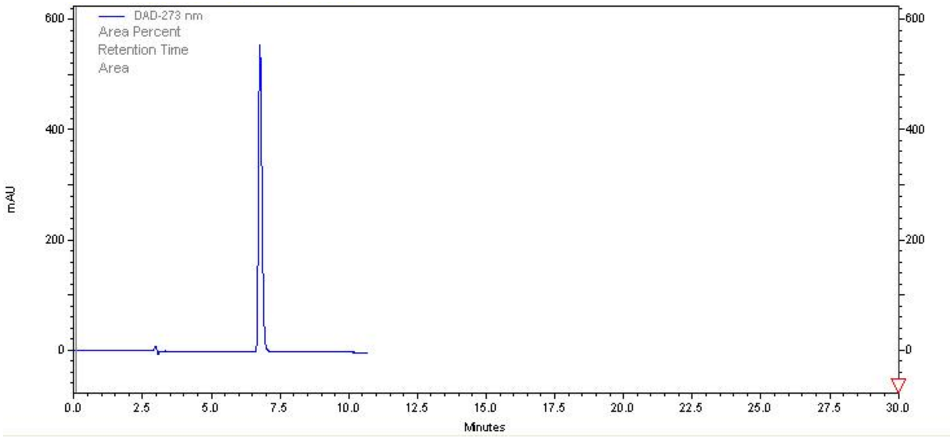

Cyclopentyl enolether (Compound 4a)

^{1}H-NMR

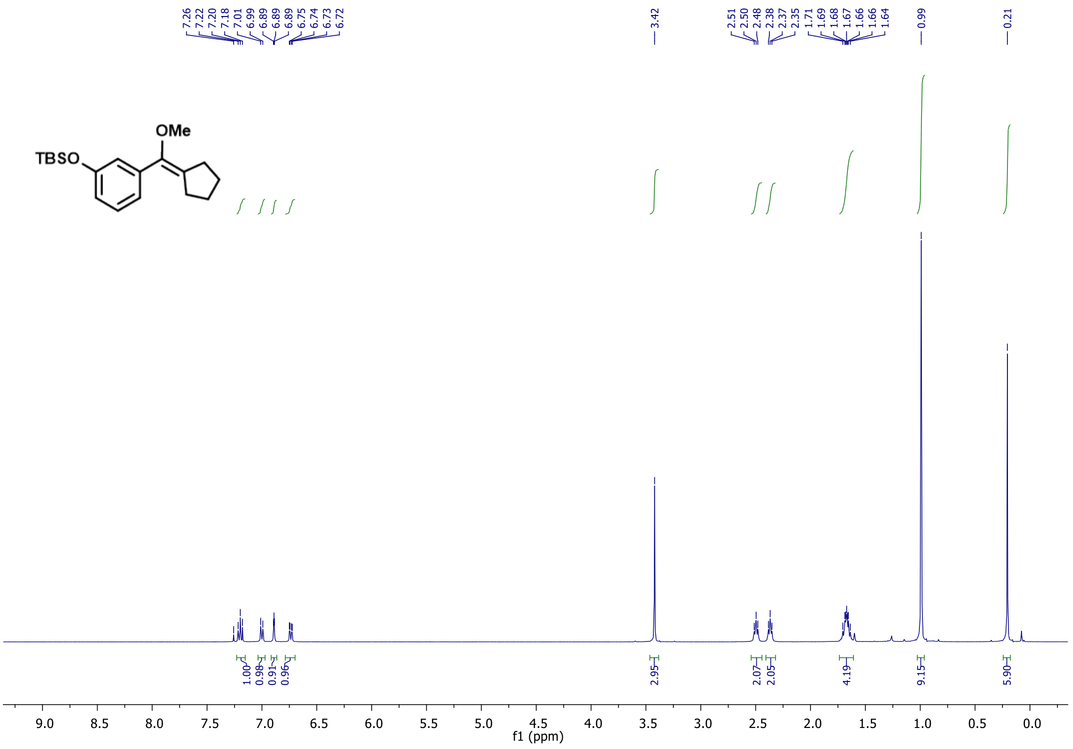

^{13}C-NMR

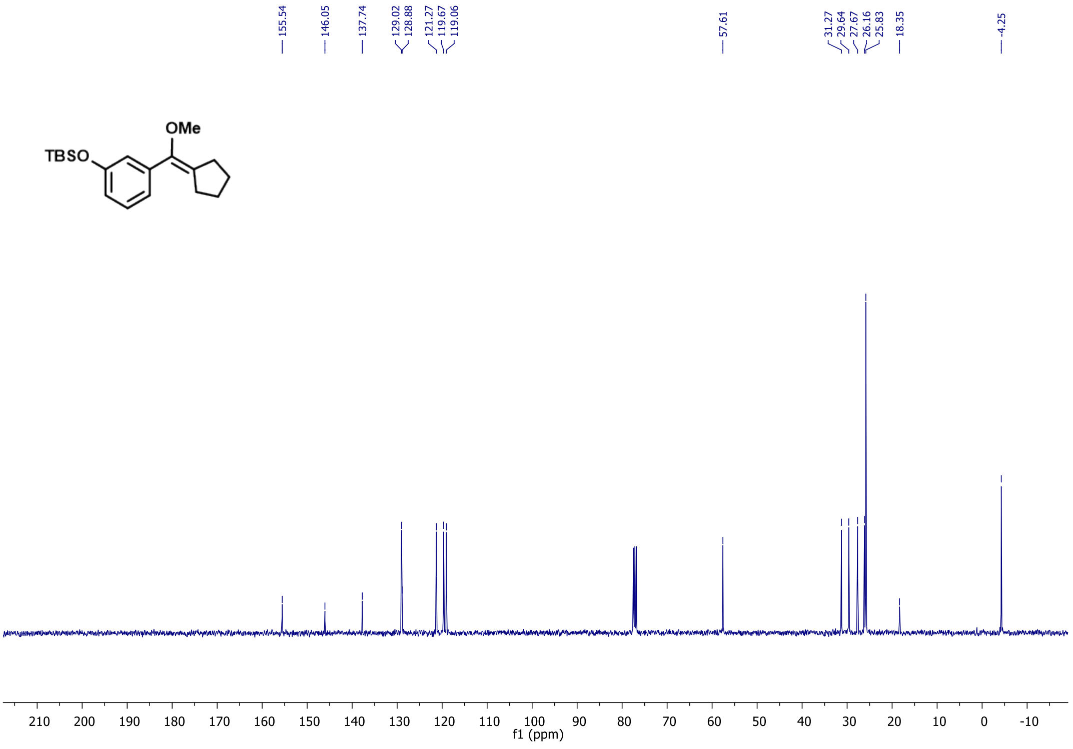

3D HPLC spectra (90-100% ACN in water, 0.1%TFA)

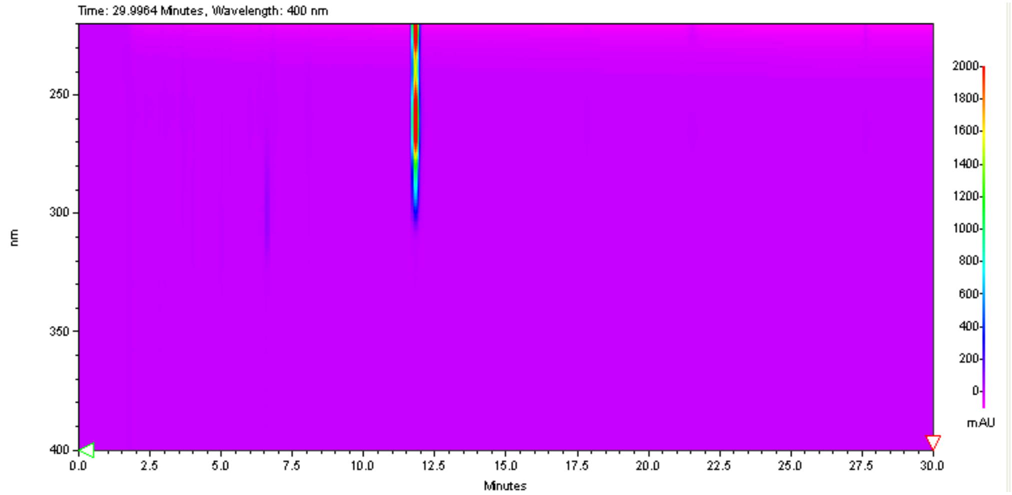

2D HPLC spectra (Absorbance measured at 275nm)

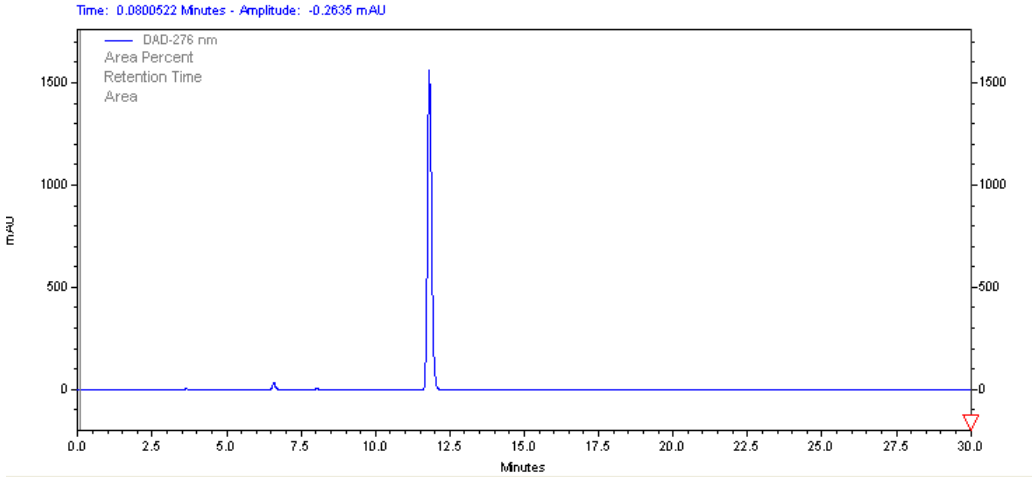

Mass spectra

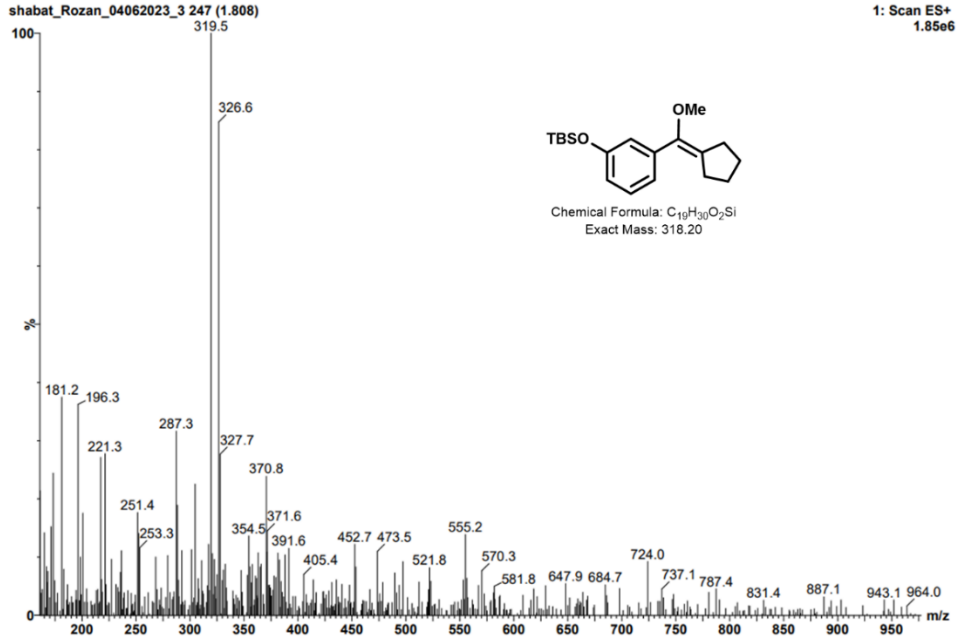

Ene-product 3

^{1}H-NMR

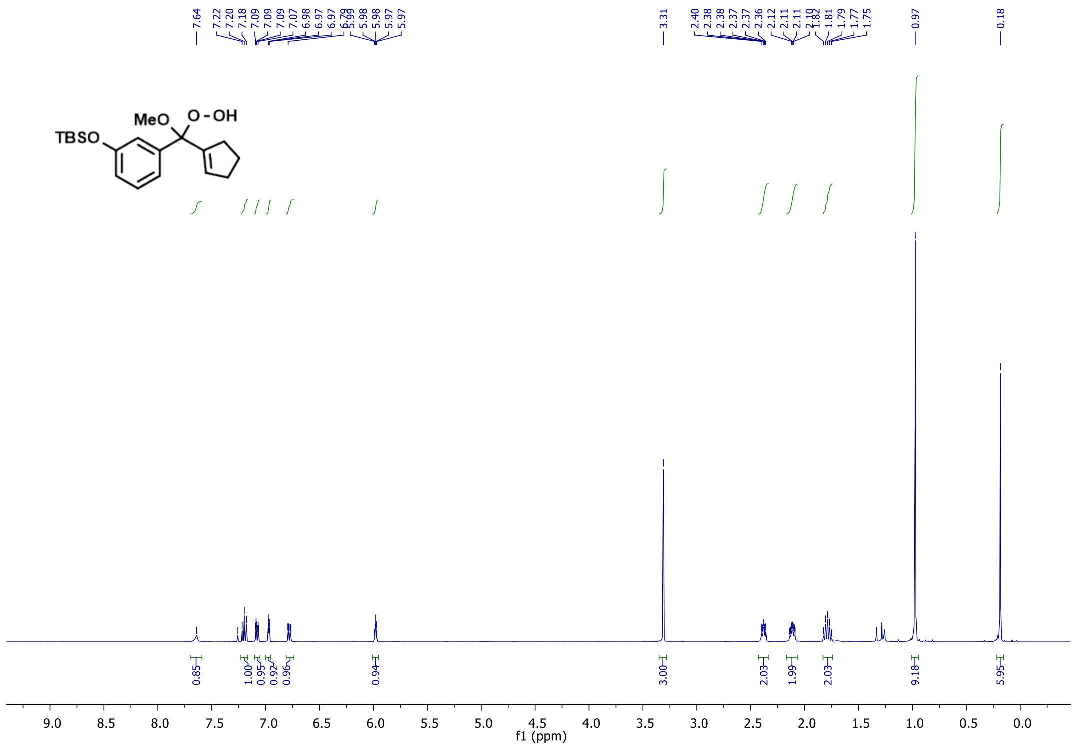

^{13}C-NMR

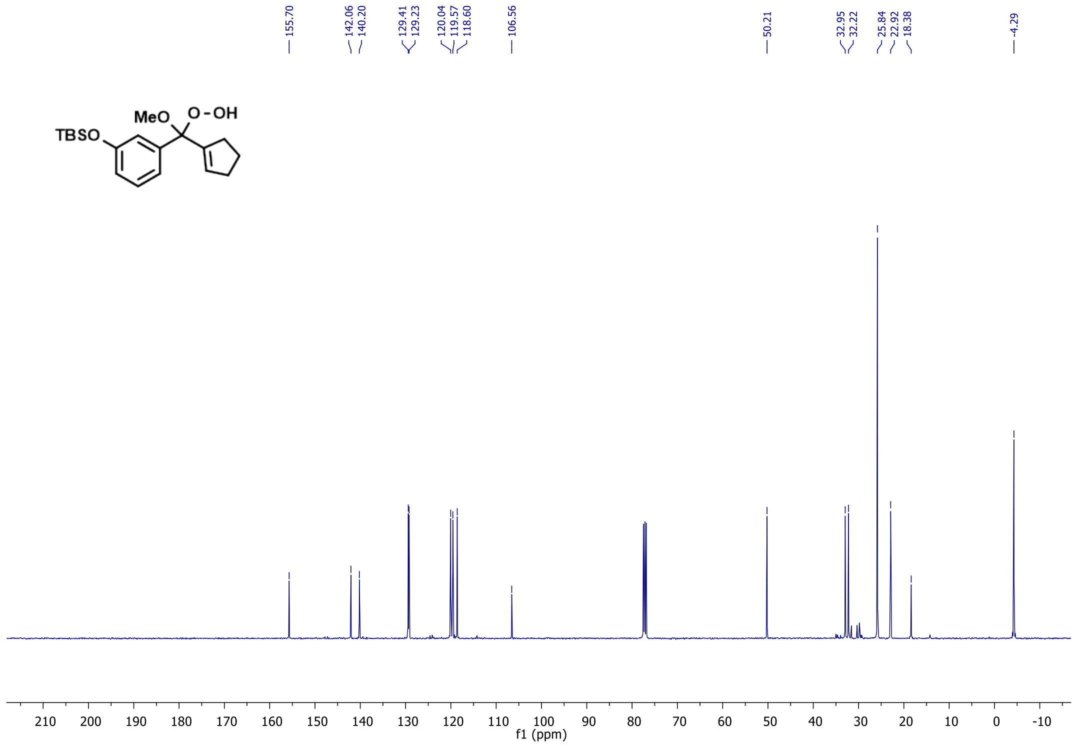

3D HPLC spectra (90-100% ACN in water, 0.1%TFA)

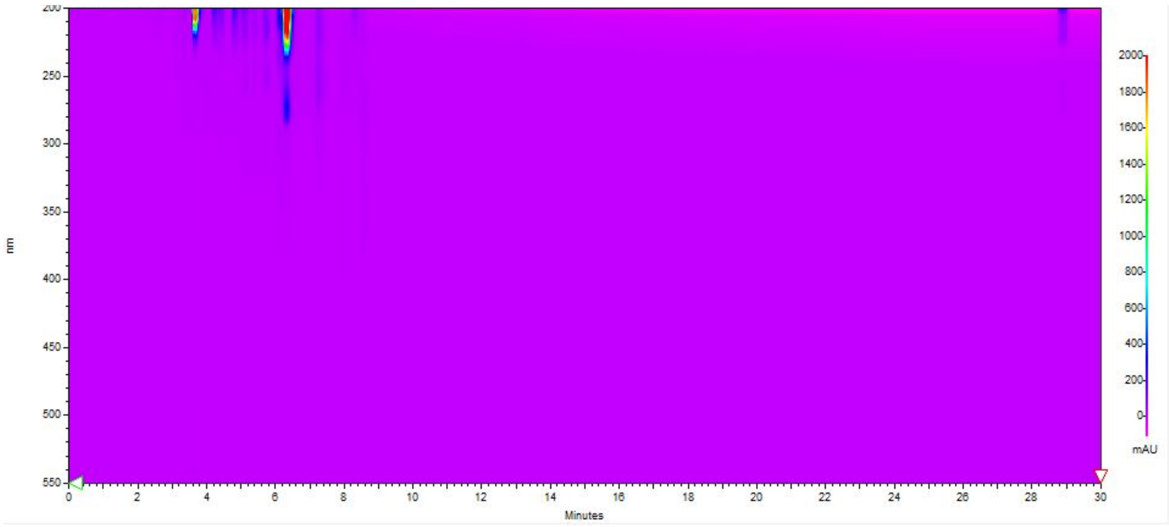

2D HPLC spectra (Absorbance measured at 275nm)

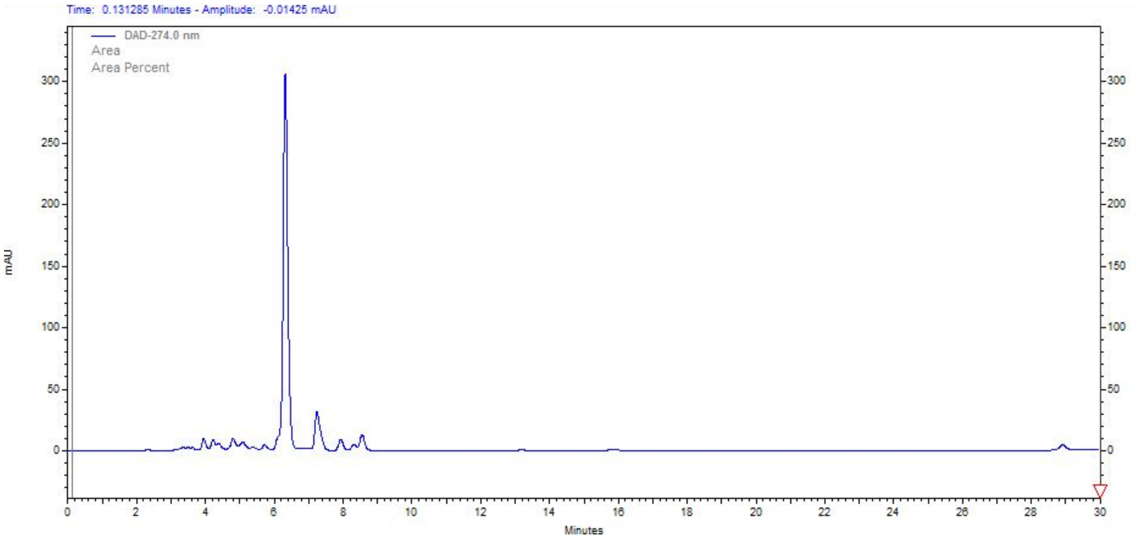

Cyclobutyl derivatives

Cyclobutyl enolether (Compound 5a)

^{1}H-NMR

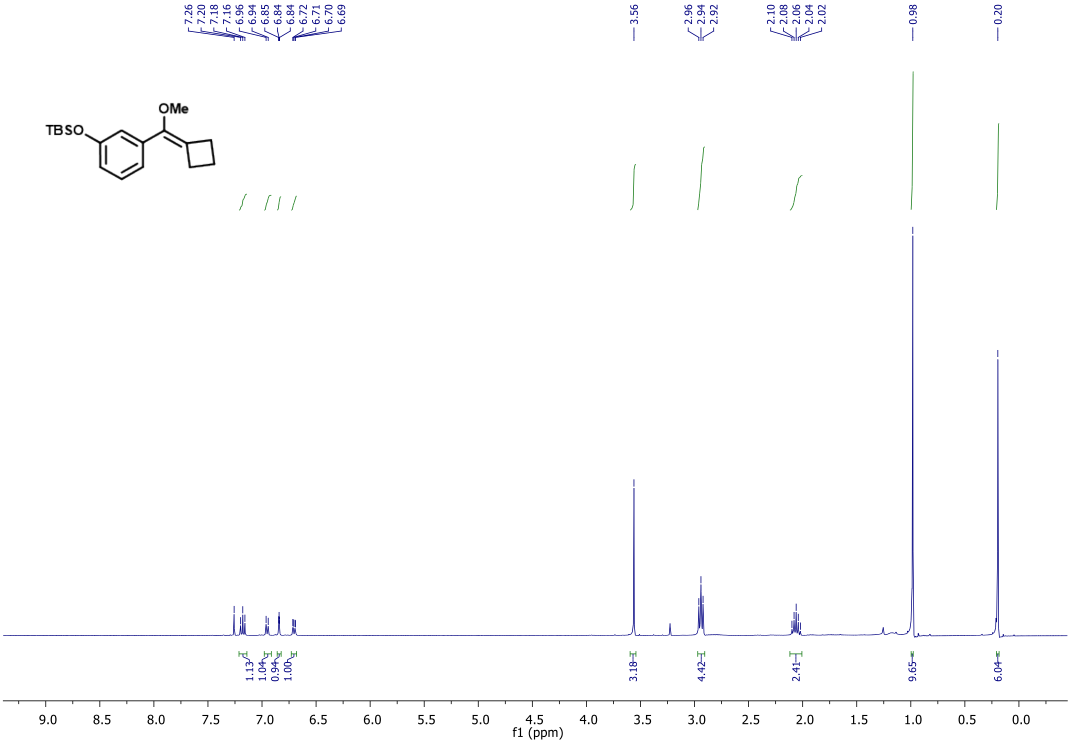

^{13}C-NMR

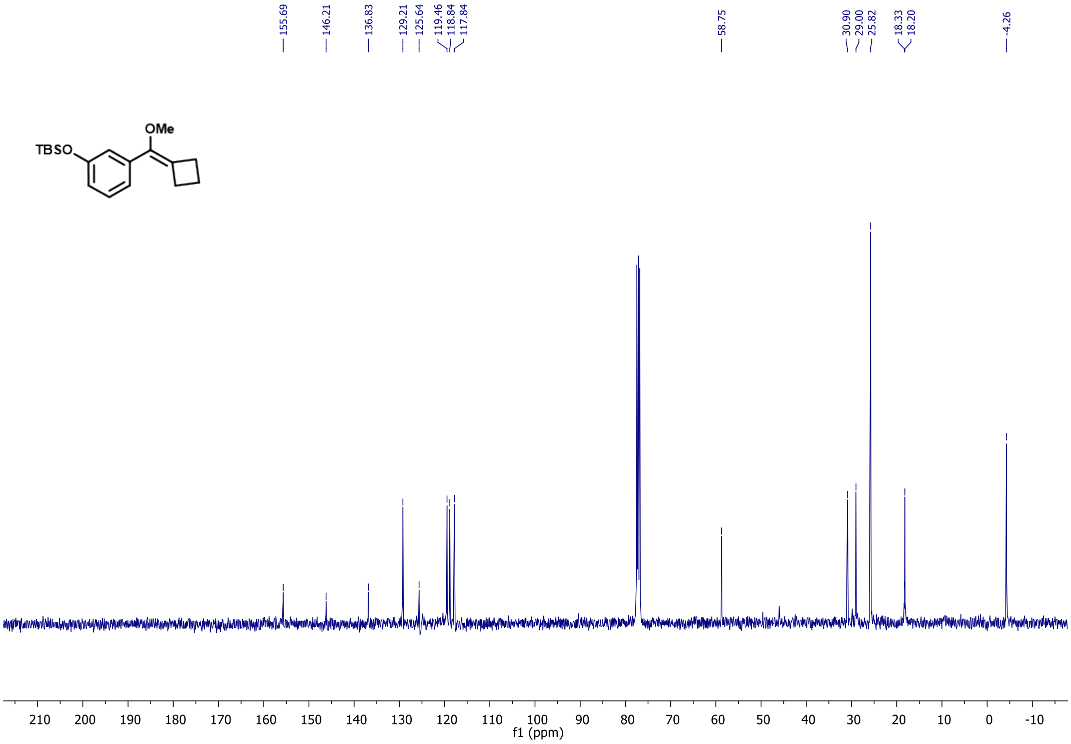

3D HPLC spectra (90-100% ACN in water, 0.1%TFA)

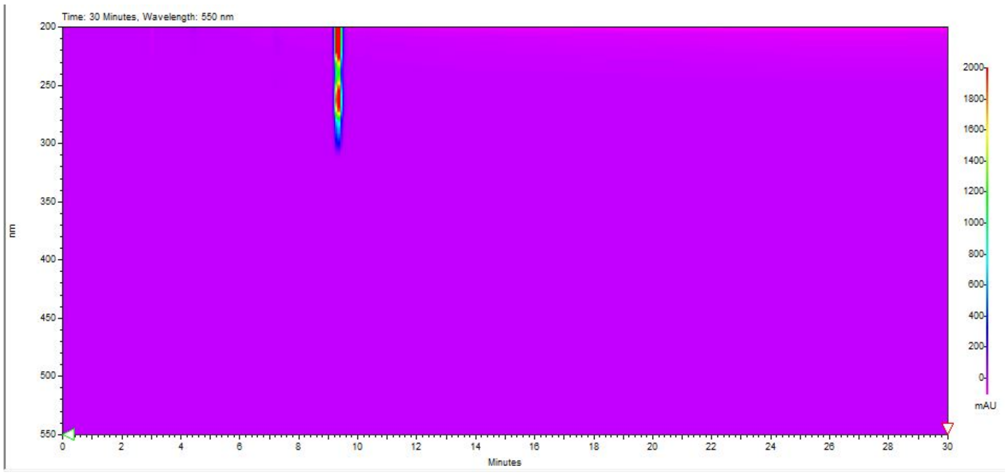

2D HPLC spectra (Absorbance measured at 275nm)

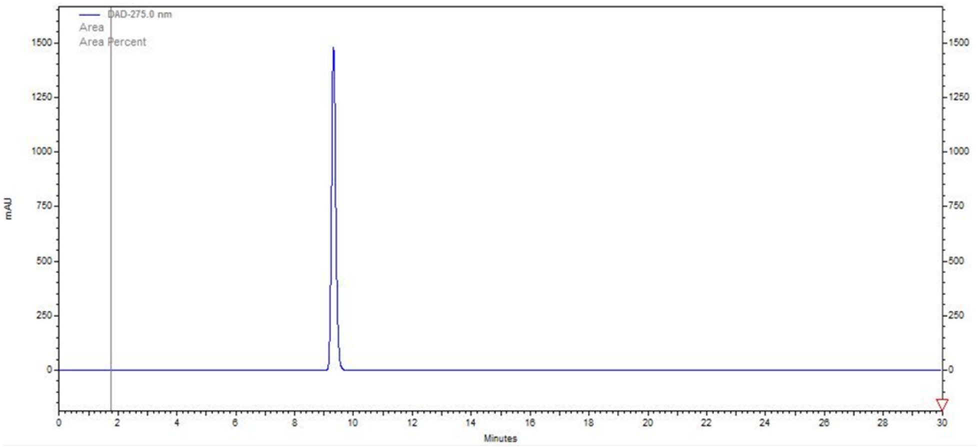

Mass spectra

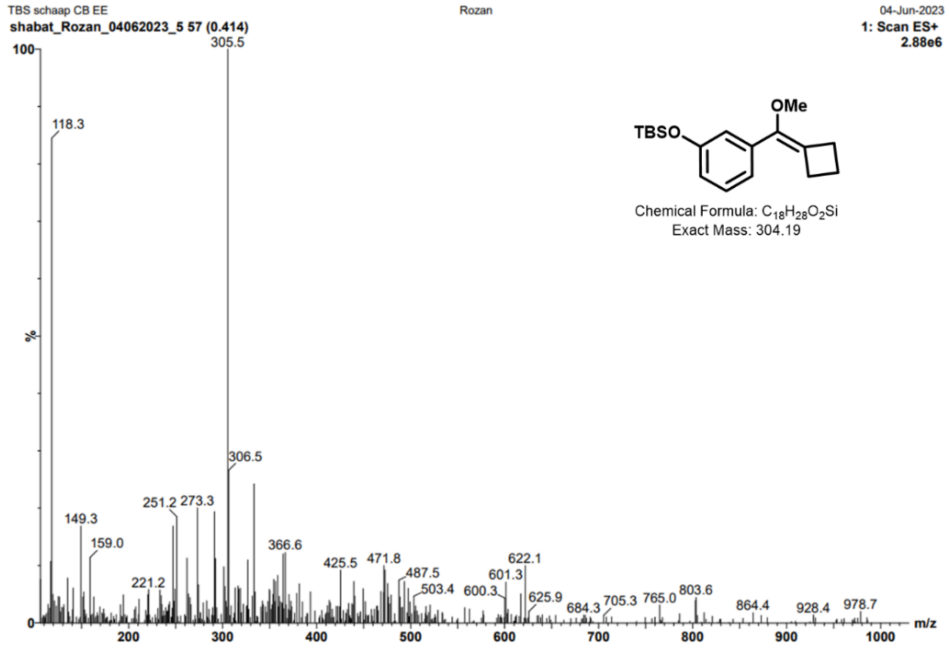

Diox 8

^{1}H-NMR

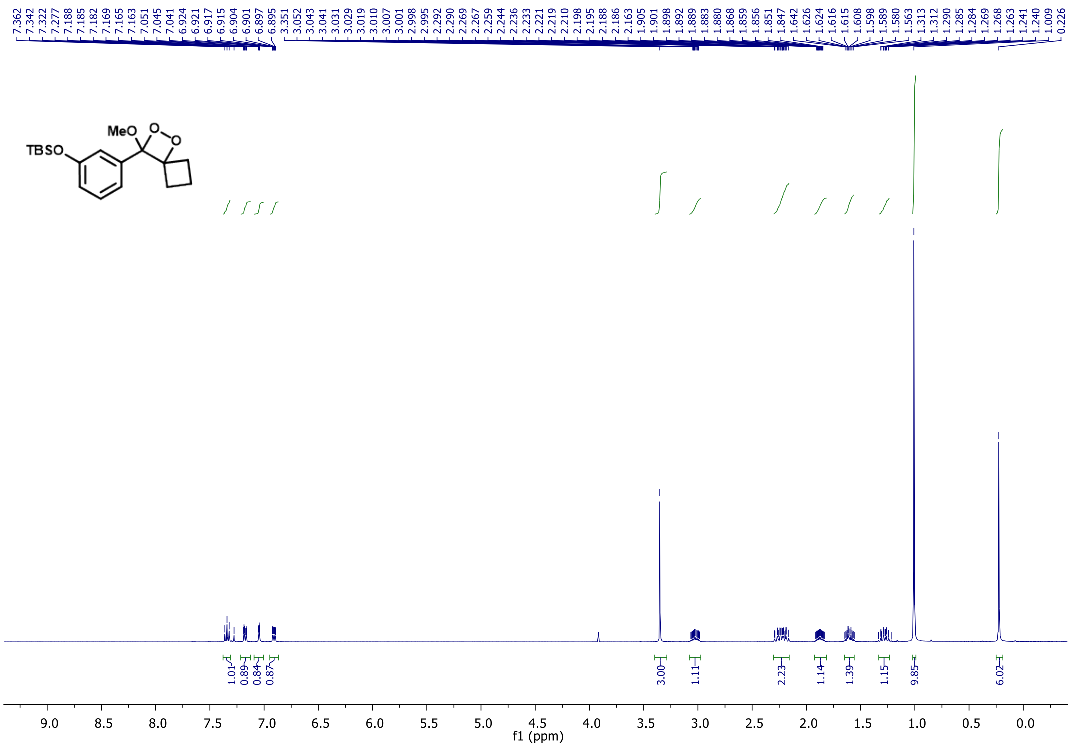

^{13}C-NMR

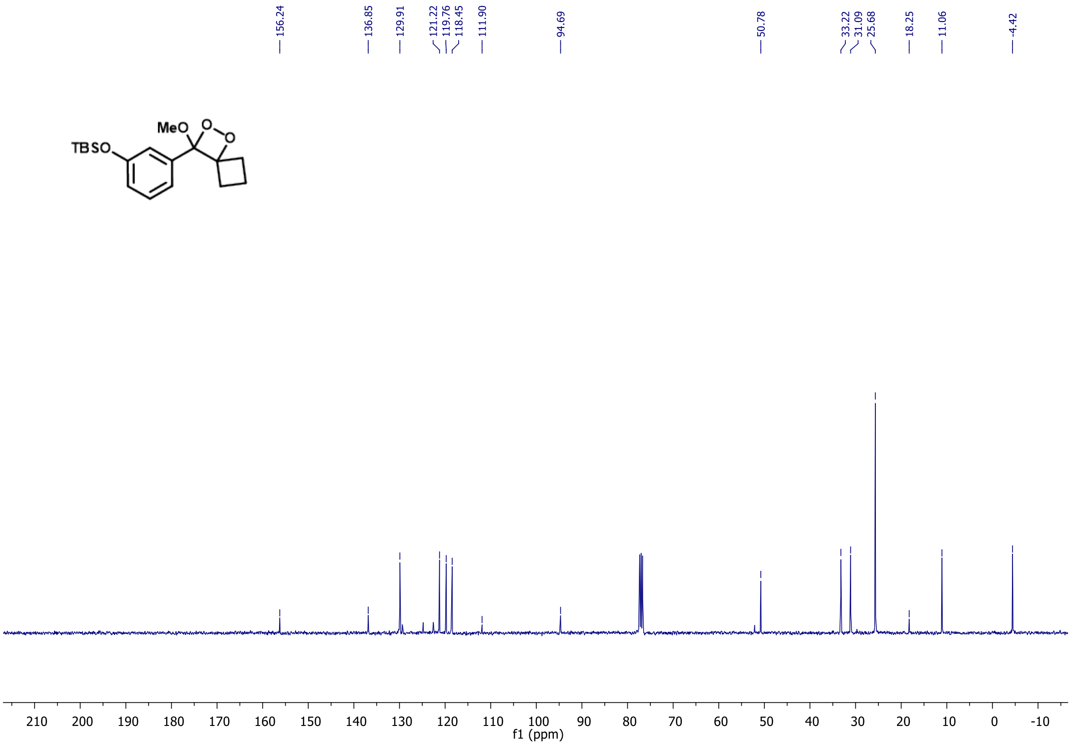

3D HPLC spectra (90-100% ACN in water, 0.1%TFA)

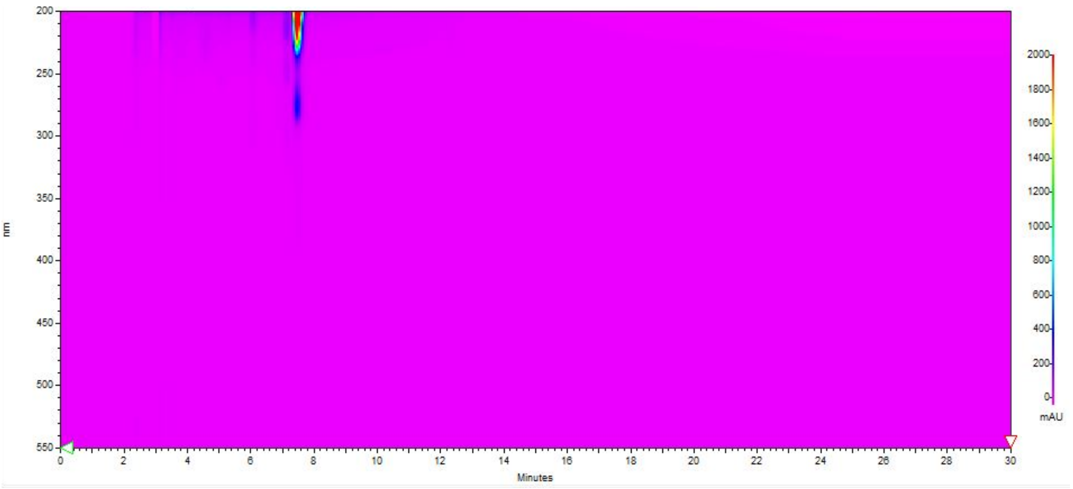

2D HPLC spectra (Absorbance measured at 277nm)

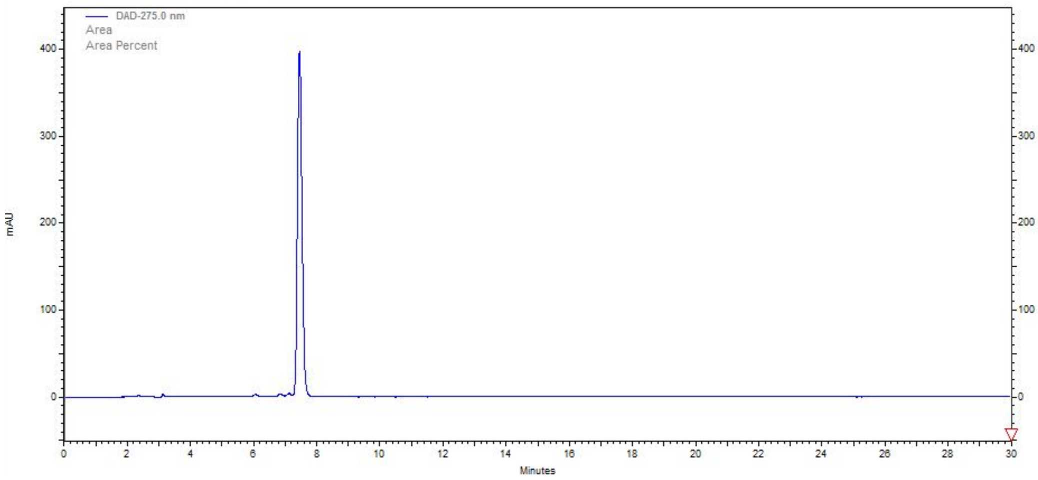

Mass spectra

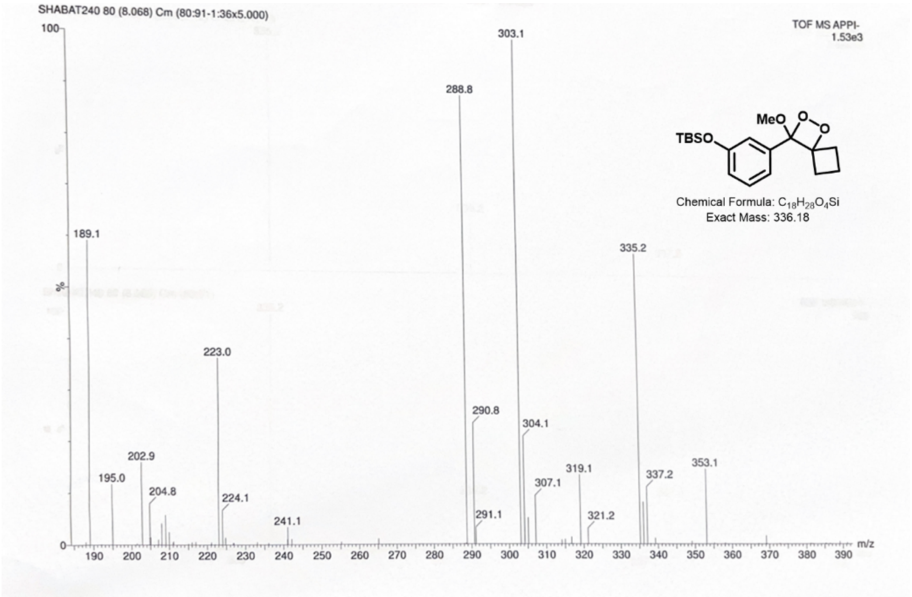

Compound 6a

^{1}H-NMR

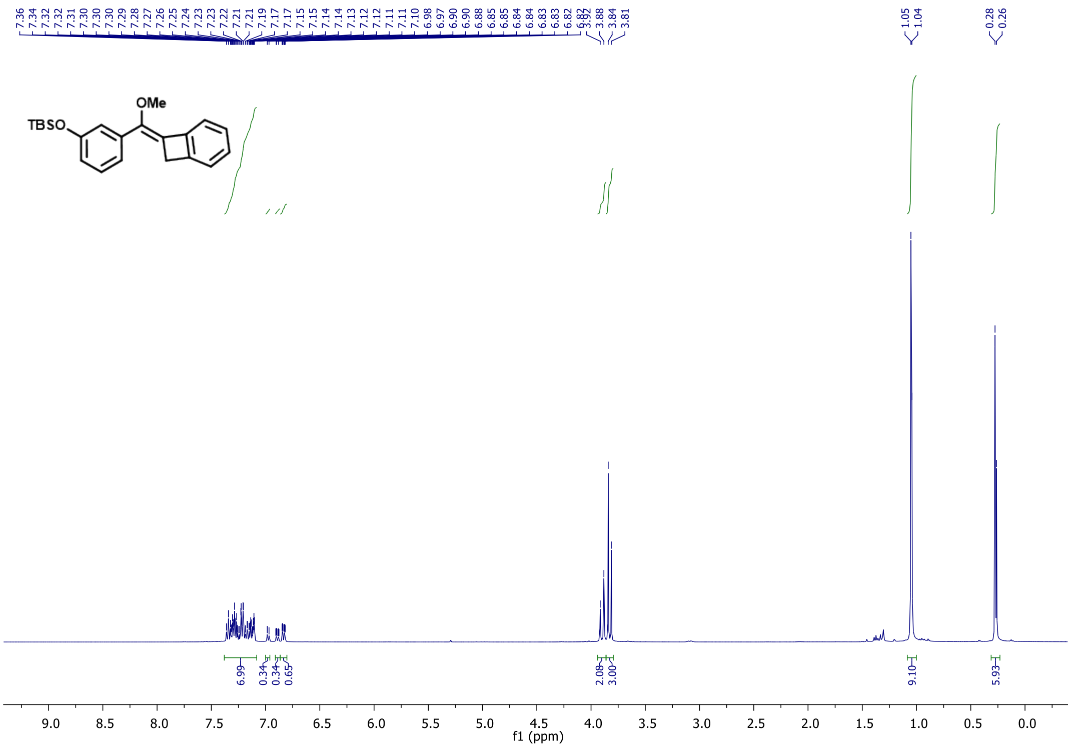

^{13}C-NMR

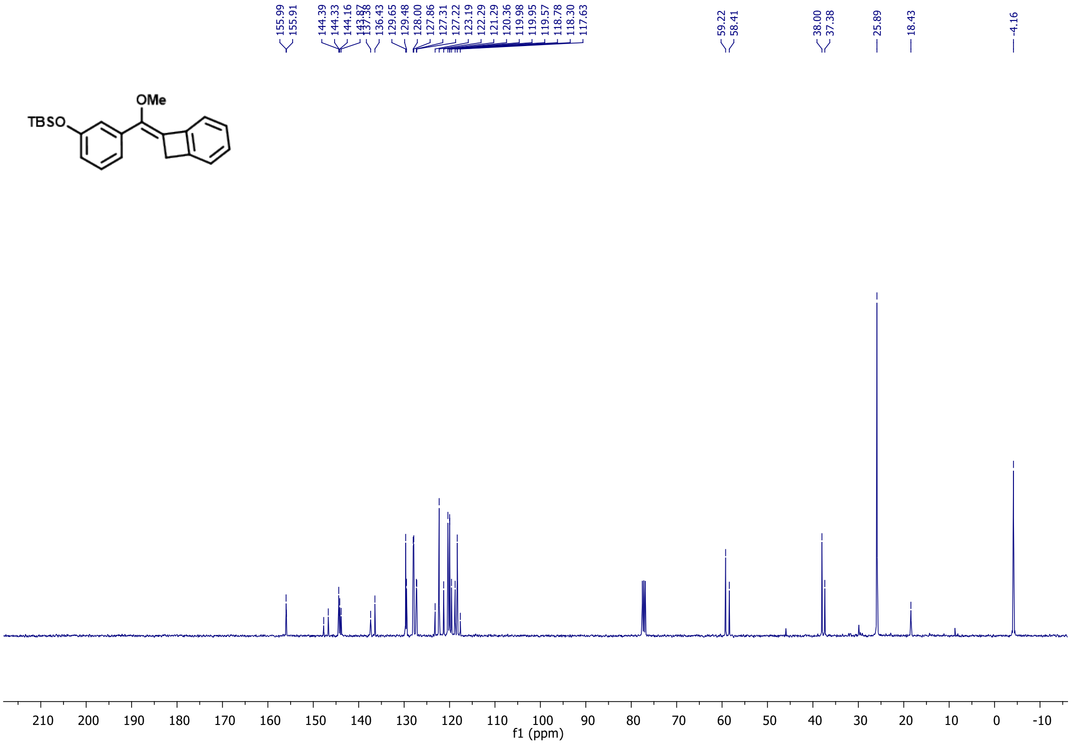

3D HPLC spectra (90-100% ACN in water, 0.1%TFA)

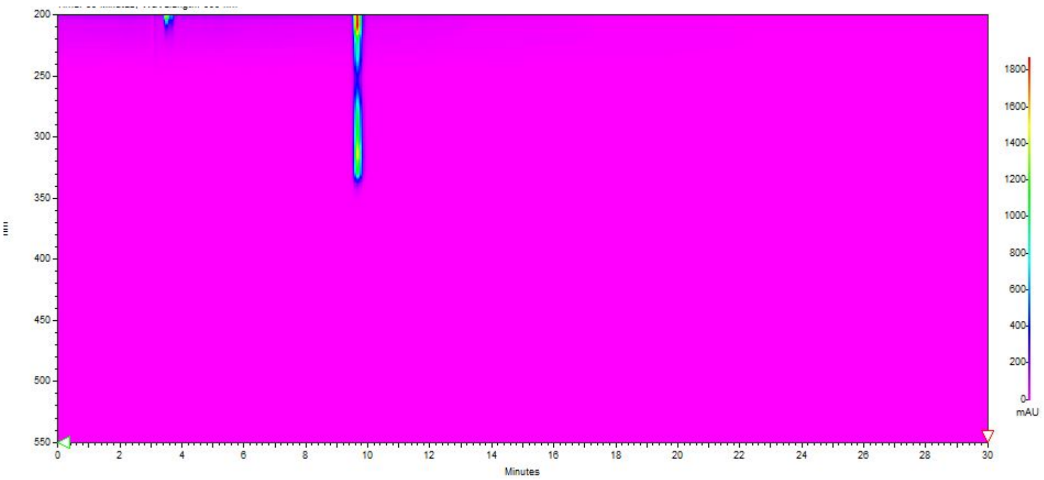

2D HPLC spectra (Absorbance measured at 300nm)

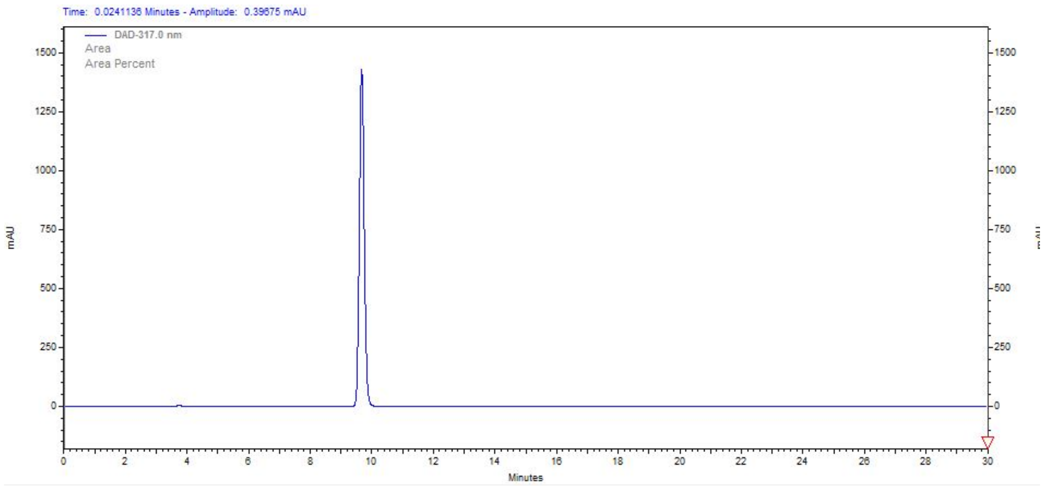

Mass spectra

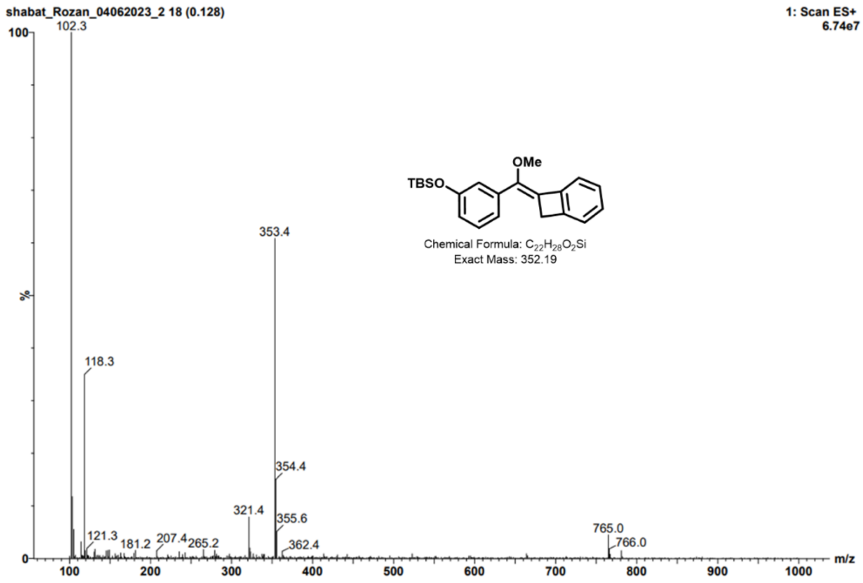

Diox 3

^{1}H-NMR

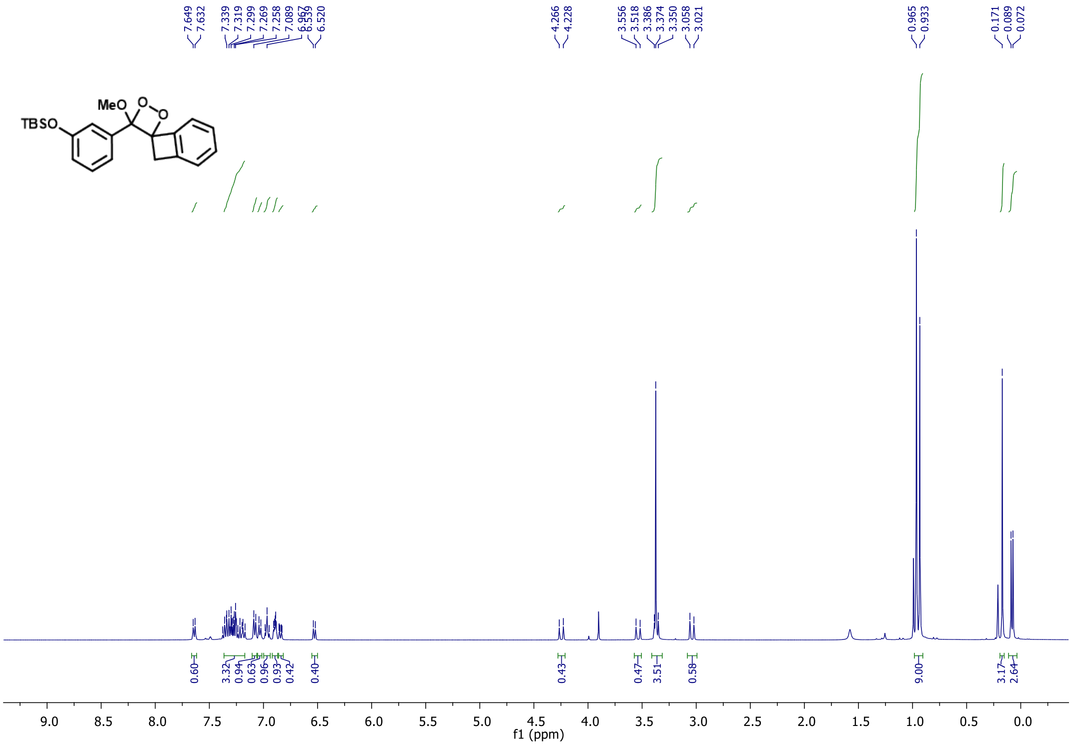

^{13}C-NMR

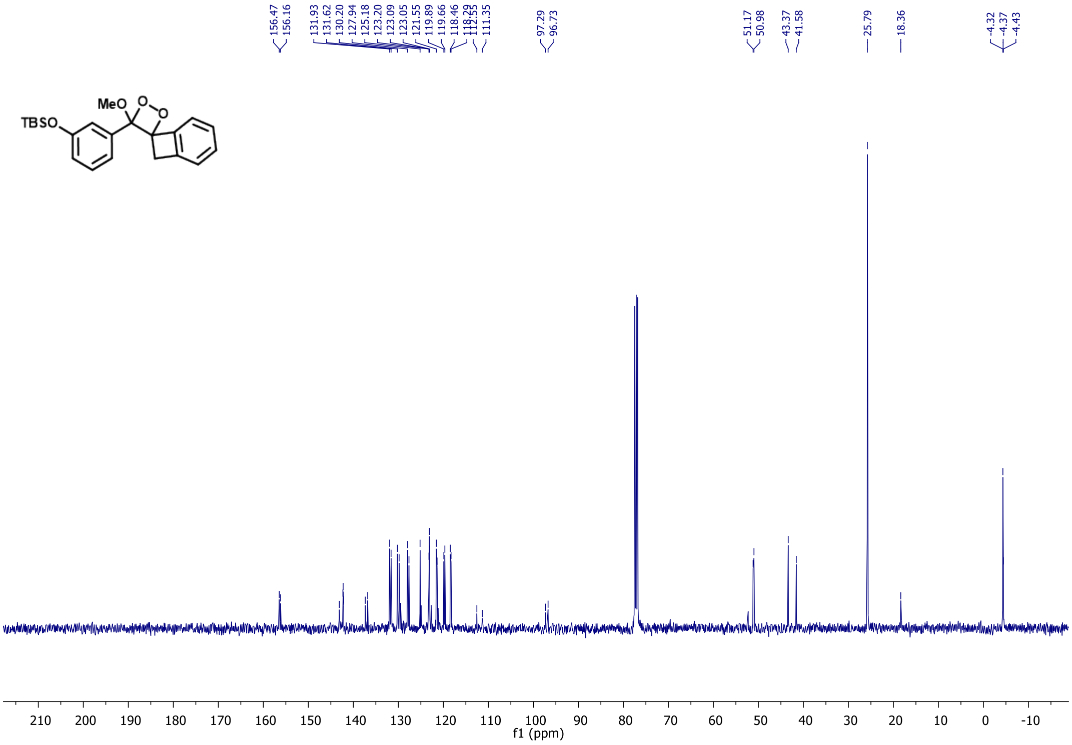

3D HPLC spectra (90-100% ACN in water, 0.1%TFA)

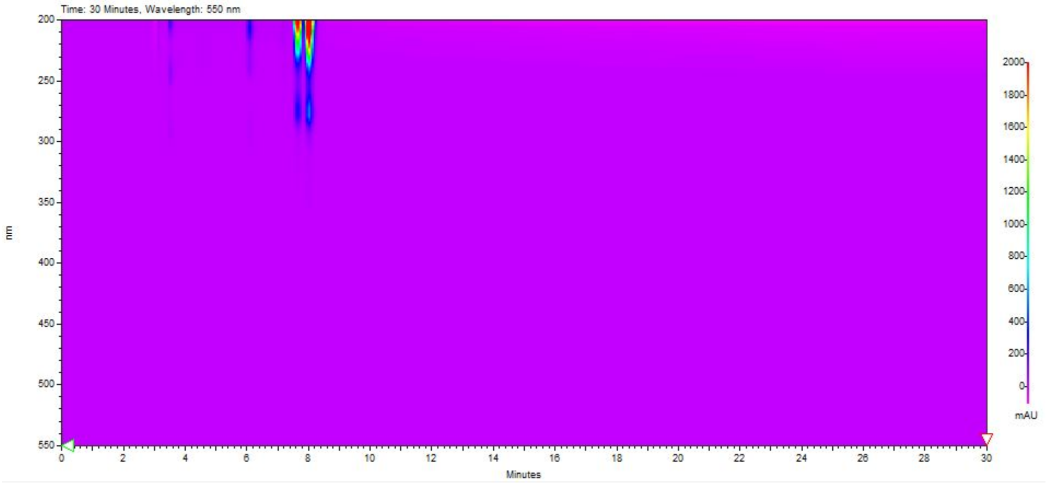

2D HPLC spectra (Absorbance measured at 277nm)

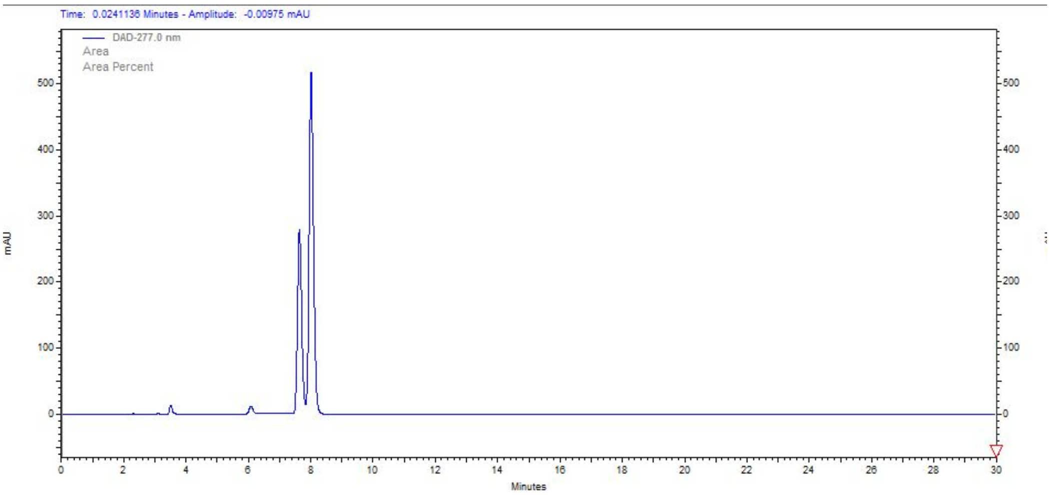

Compound 7a

^{1}H-NMR

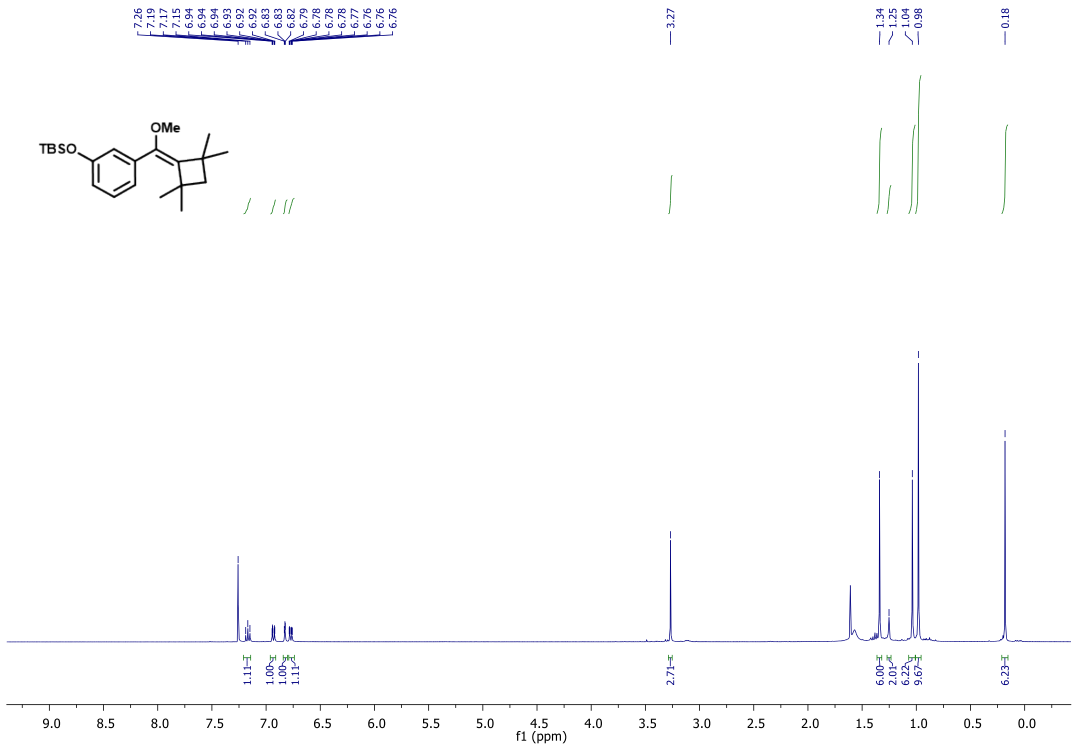

^{13}C-NMR

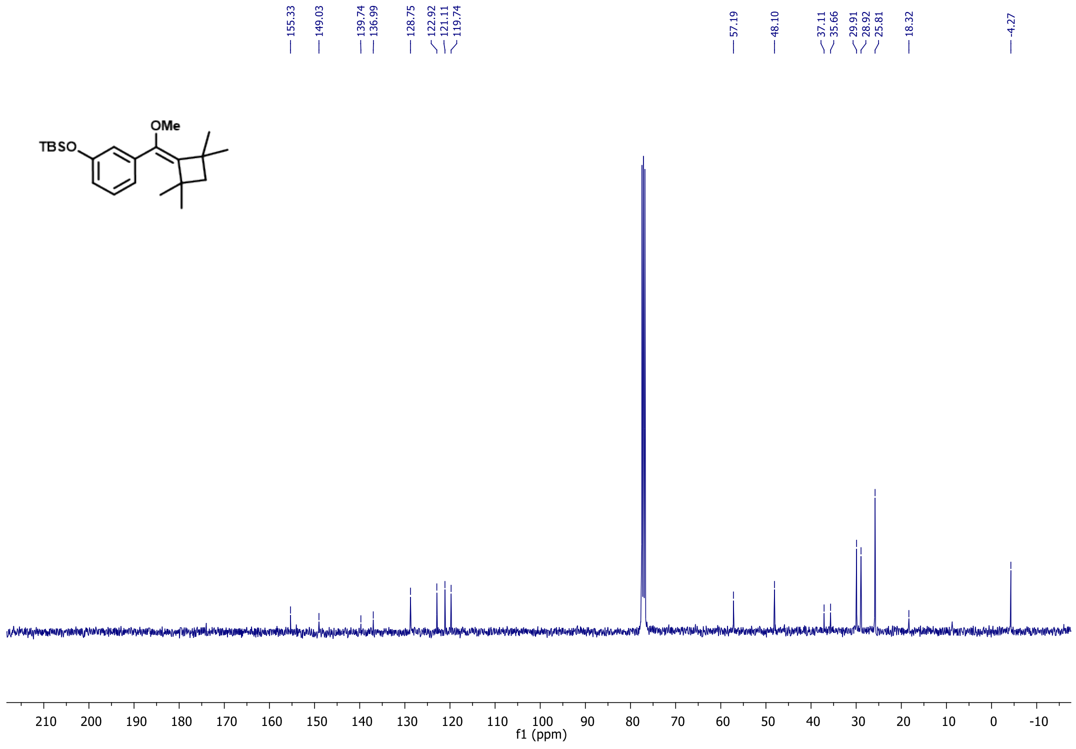

3D HPLC spectra (90-100% ACN in water, 0.1%TFA)

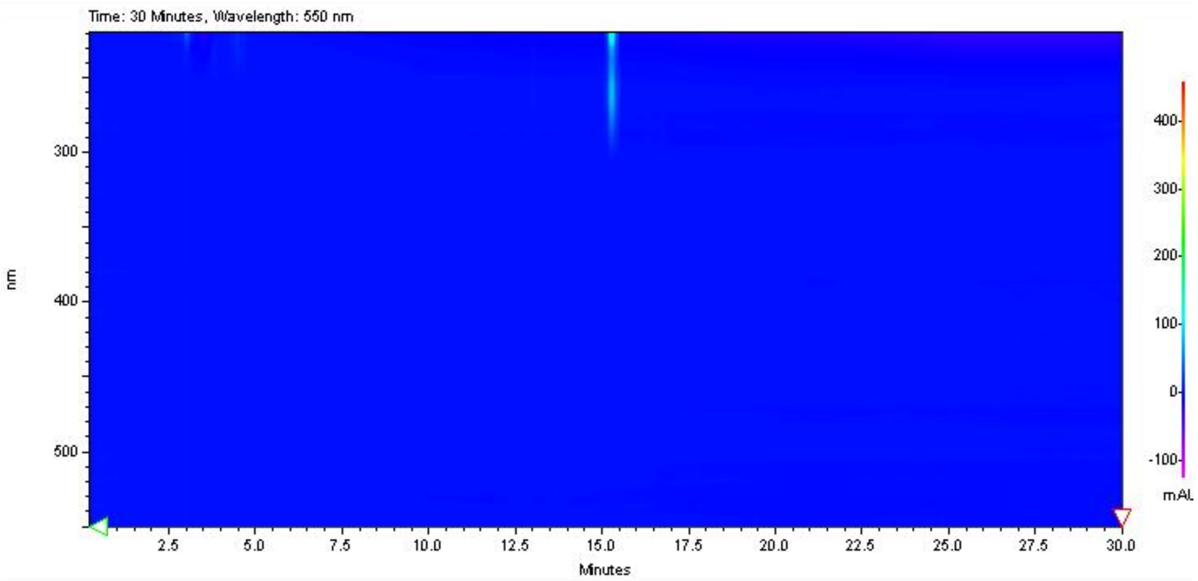

2D HPLC spectra (Absorbance measured at 275nm)

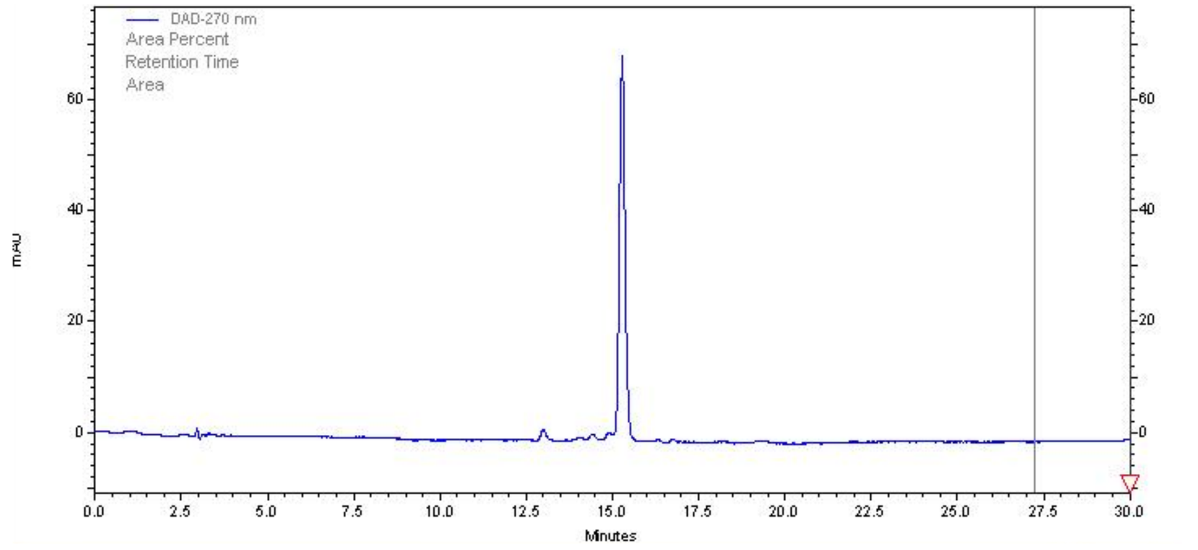

Diox 4

^{1}H-NMR

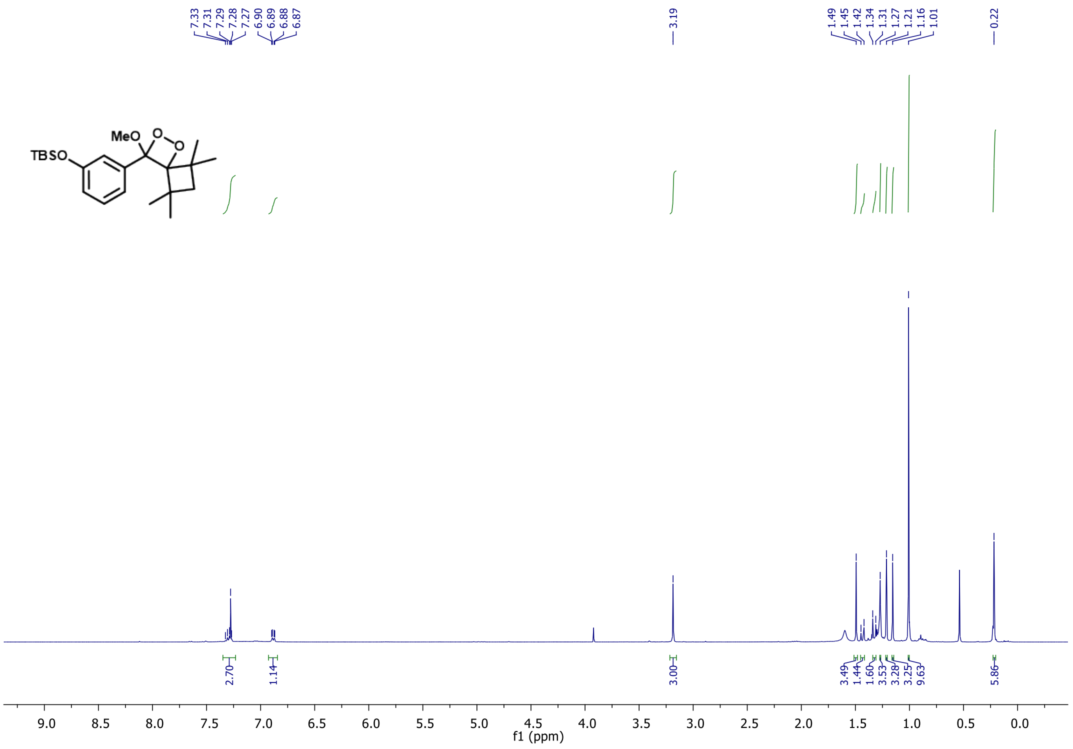

^{13}C-NMR

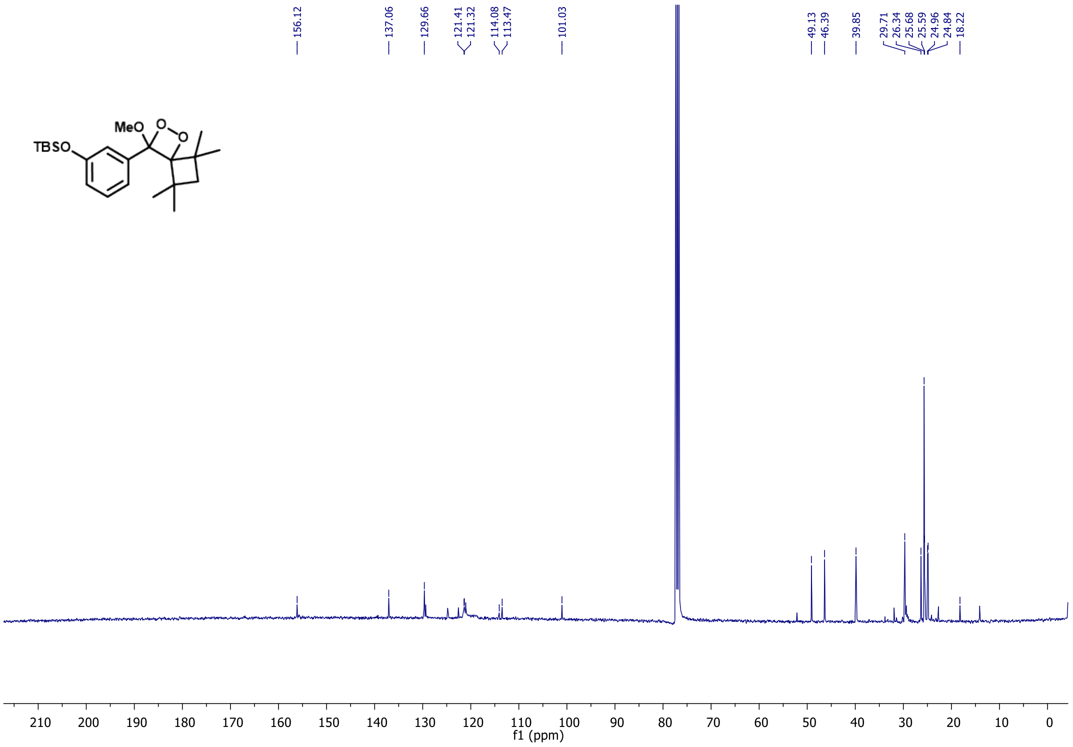

3D HPLC spectra (90-100% ACN in water, 0.1%TFA)

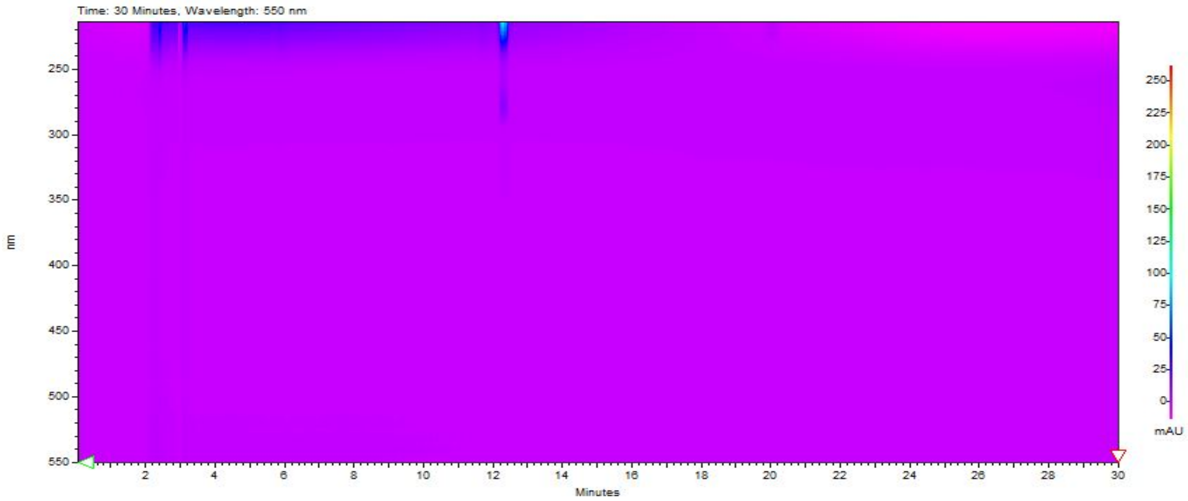

2D HPLC spectra (Absorbance measured at 277nm)

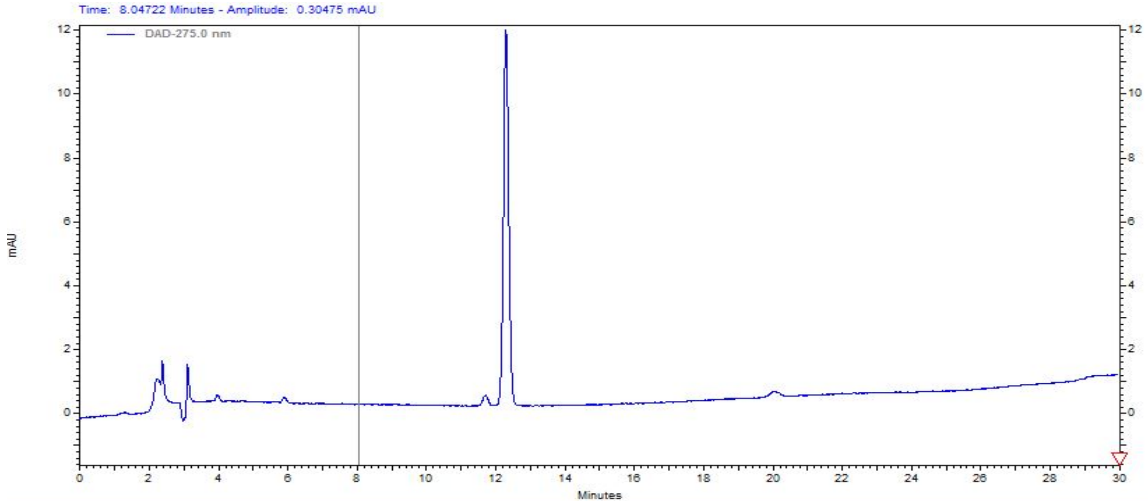

Compound 8a

^{1}H-NMR

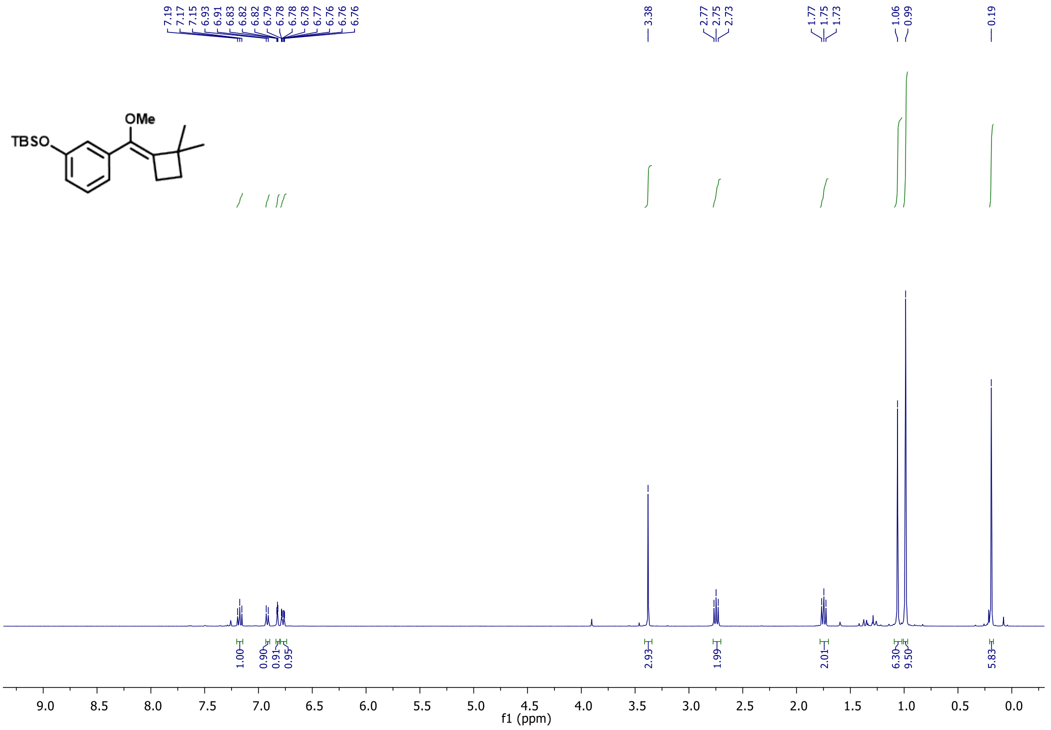

^{13}C-NMR

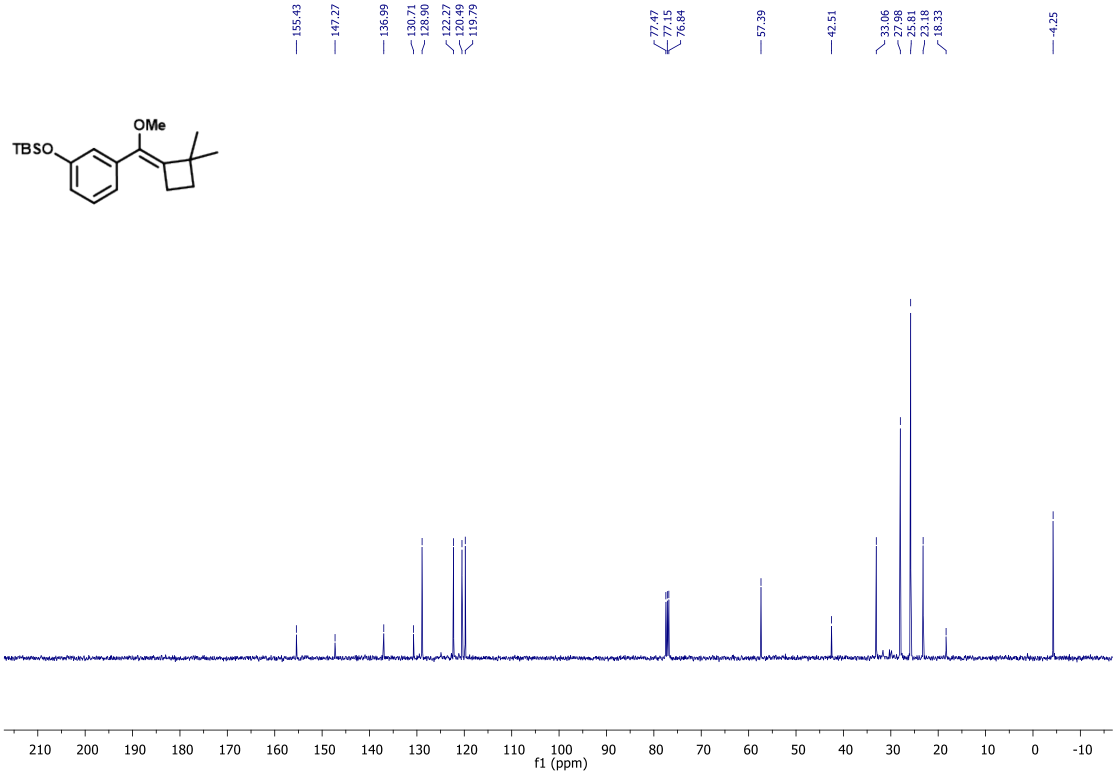

3D HPLC spectra (90-100% ACN in water, 0.1%TFA)

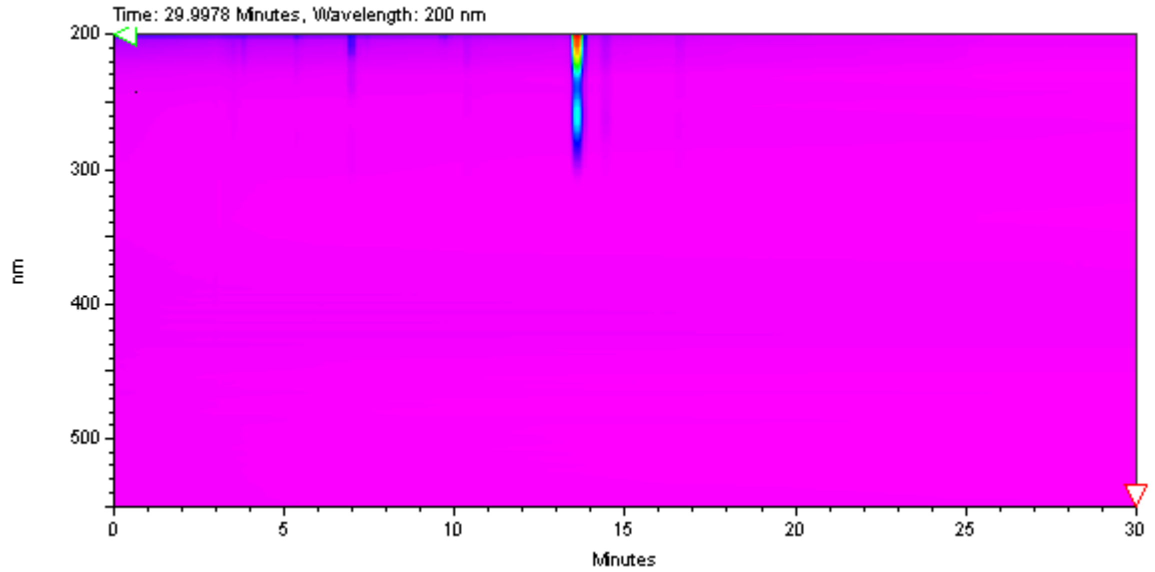

2D HPLC spectra (Absorbance measured at 267nm)

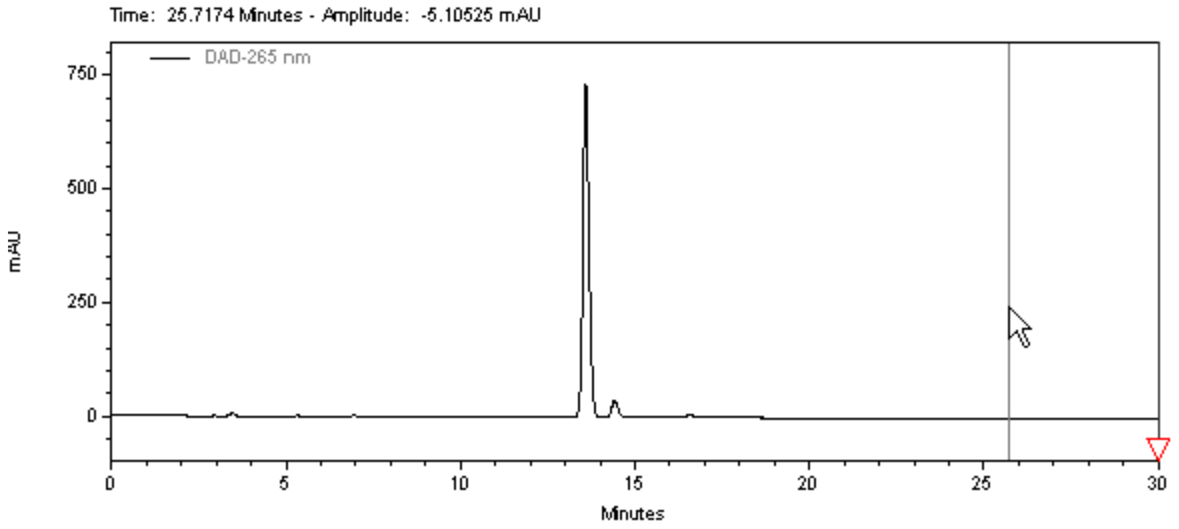

Diox 5

^{1}H-NMR

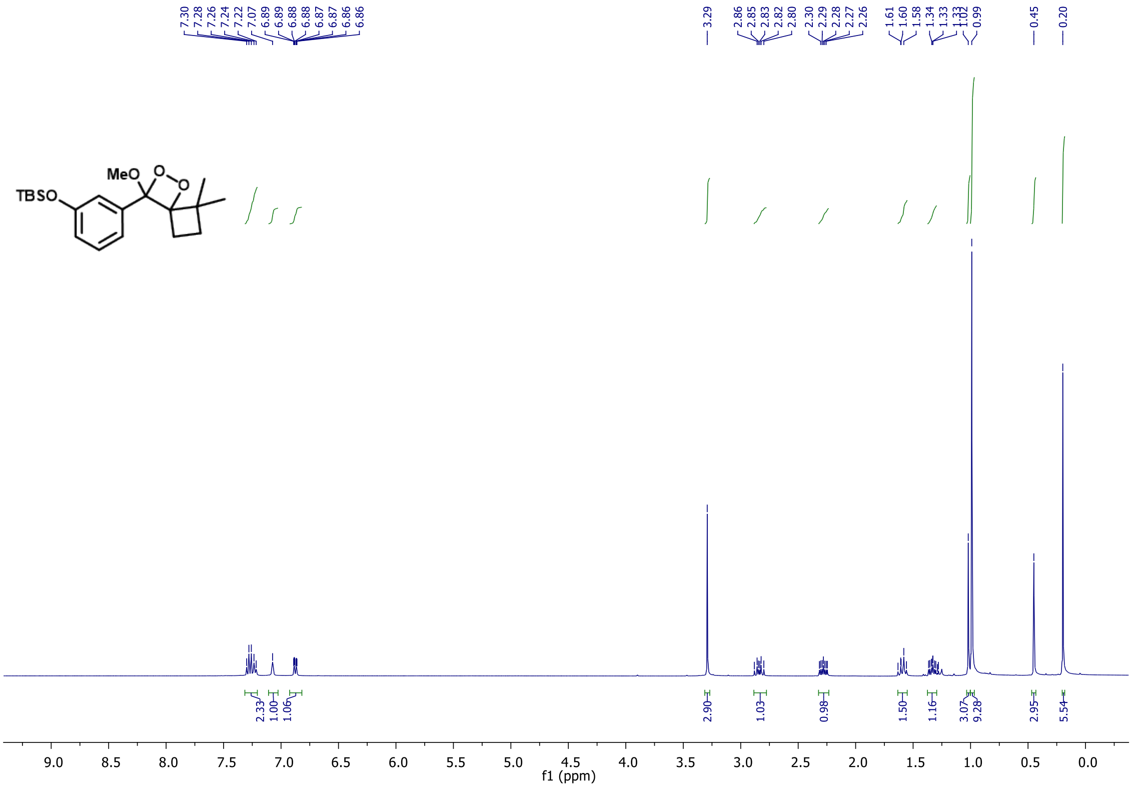

^{13}C-NMR

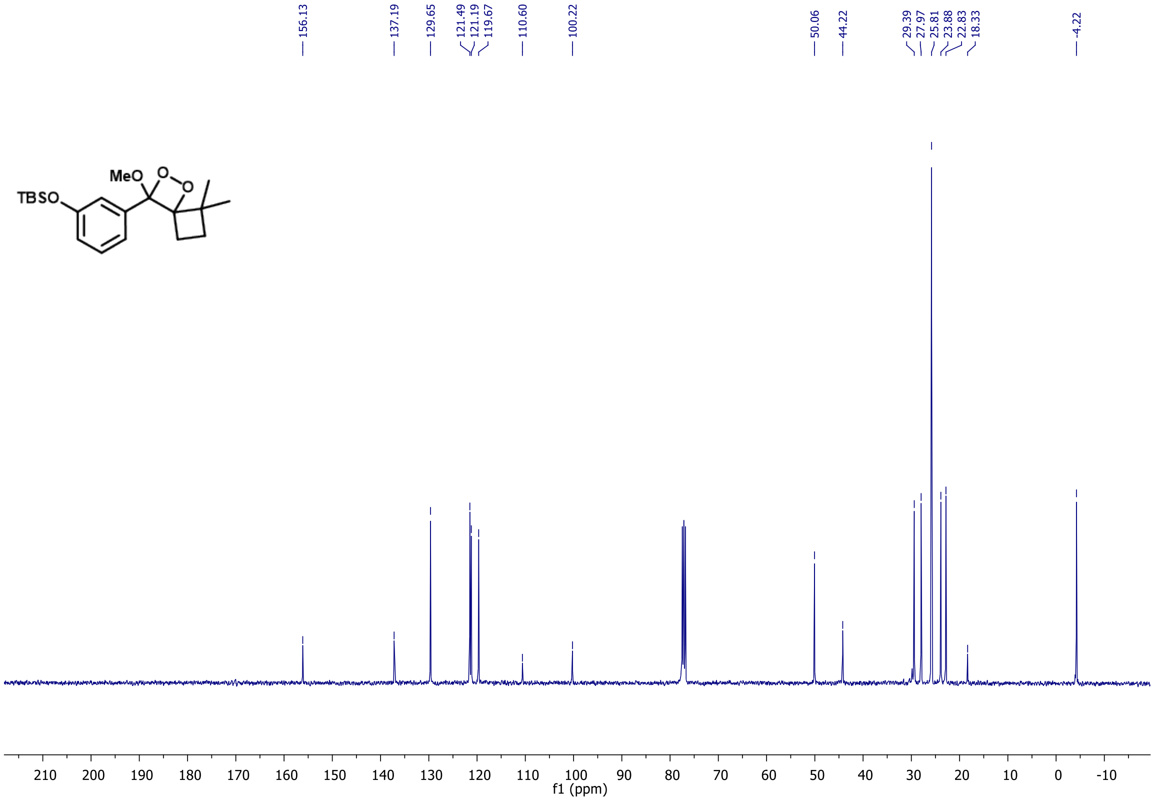

3D HPLC spectra (90-100% ACN in water, 0.1%TFA)

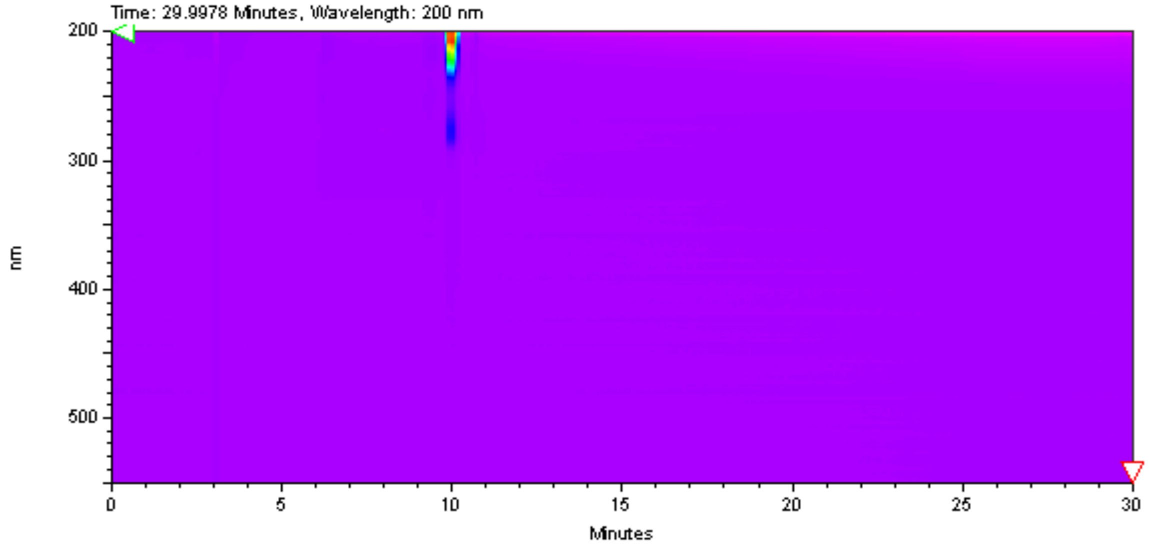

2D HPLC spectra (Absorbance measured at 277nm)

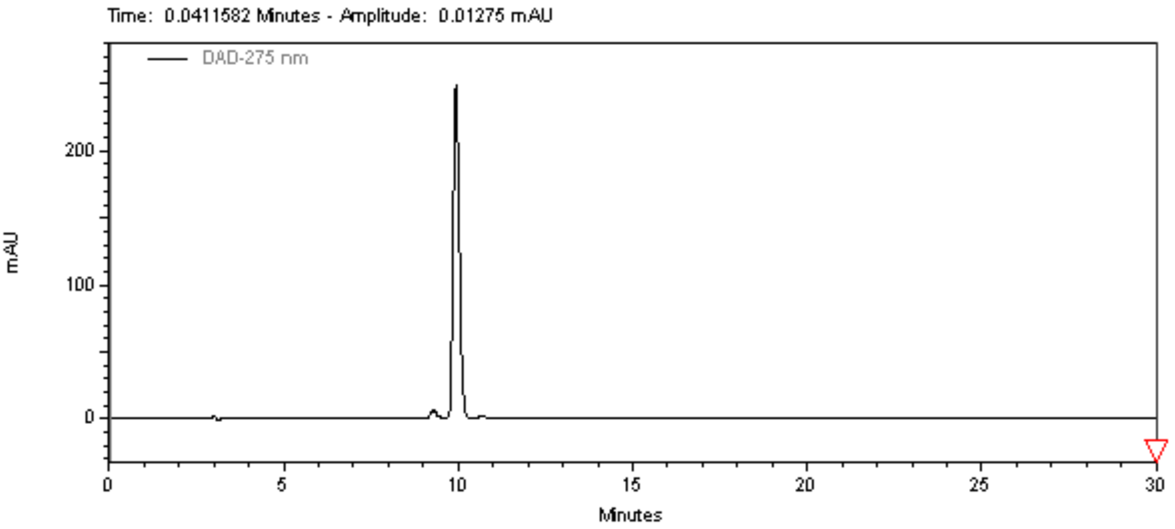

Compound 9a

^{1}H-NMR

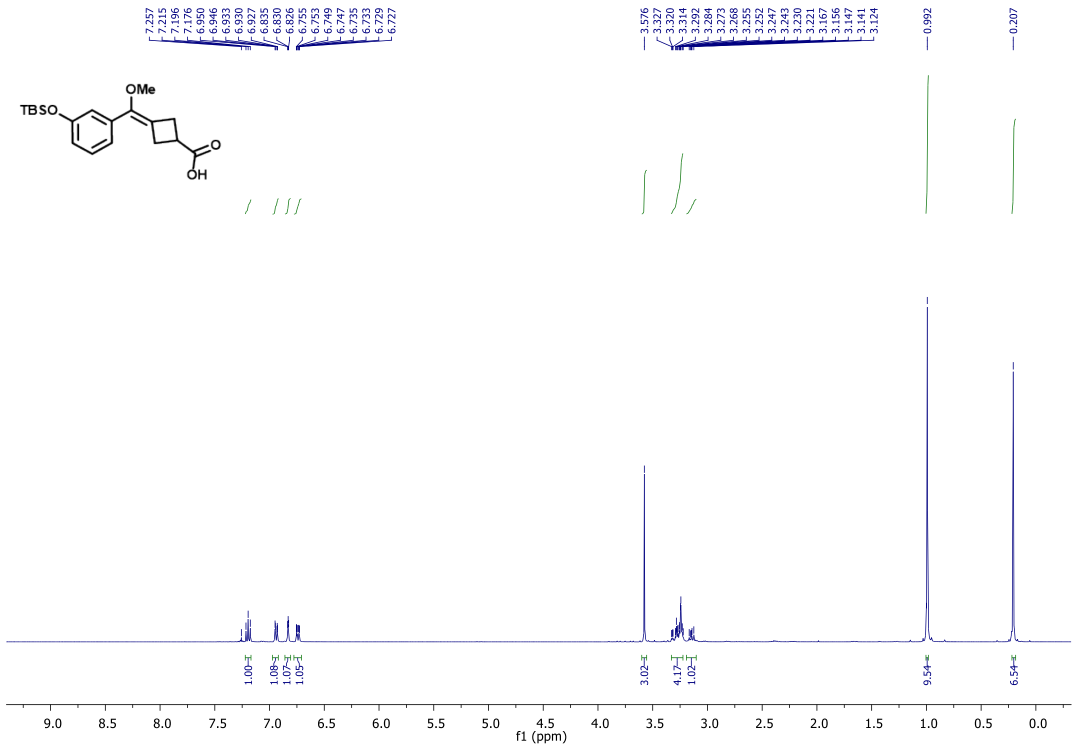

^{13}C-NMR

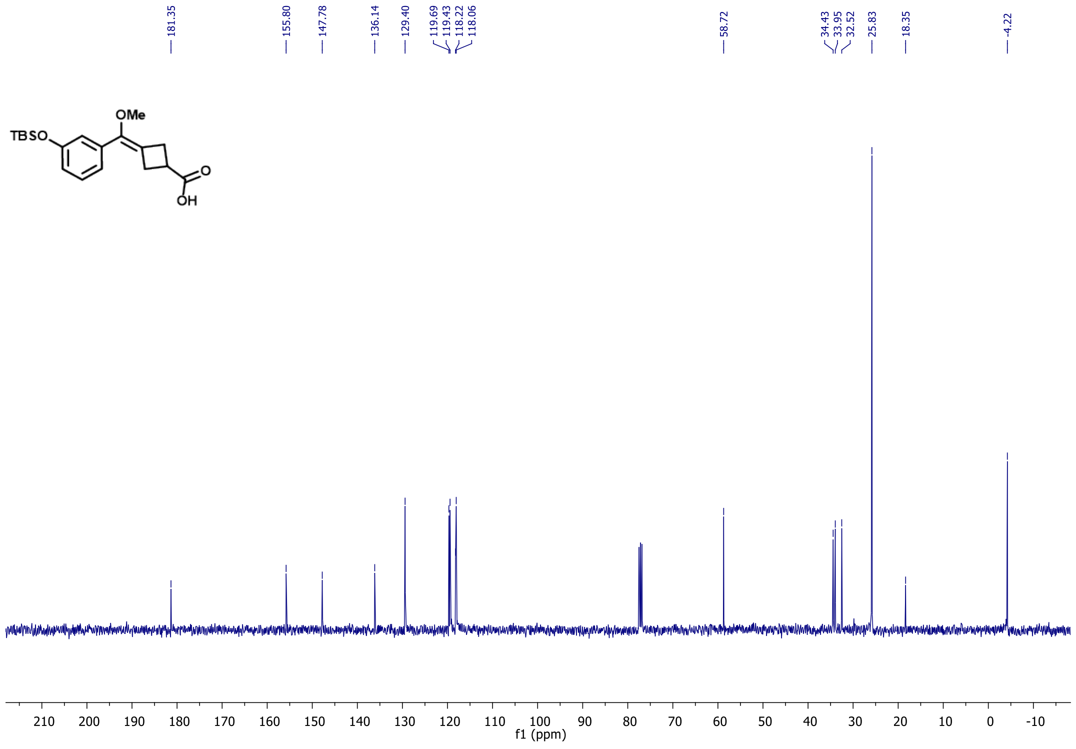

3D HPLC spectra (90-100% ACN in water, 0.1%TFA)

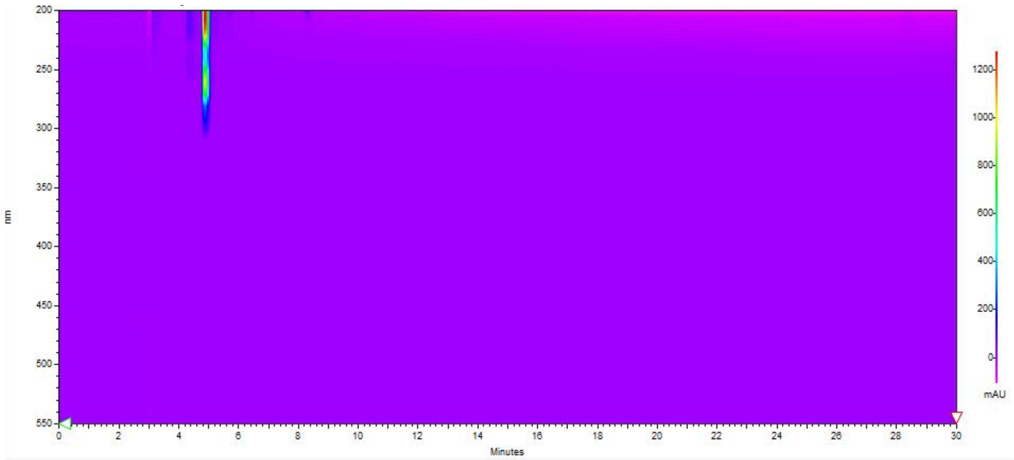

2D HPLC spectra (Absorbance measured at 275nm)

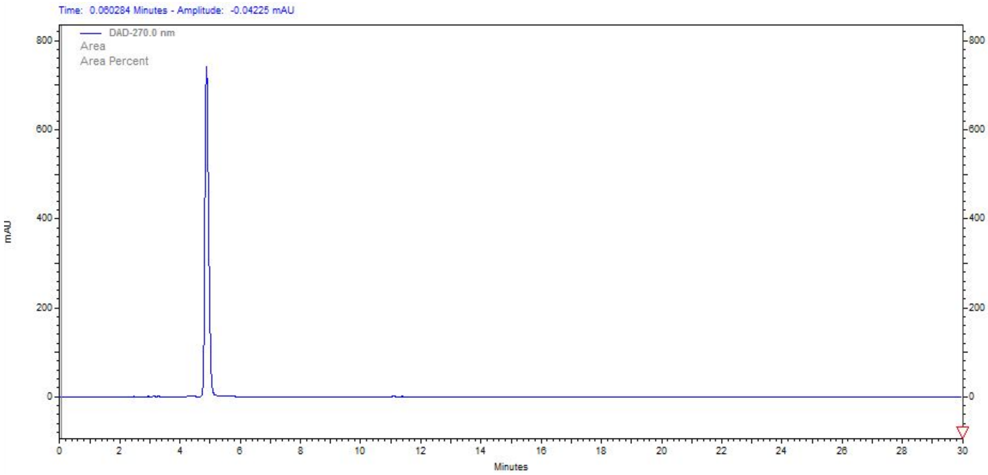

Mass spectra

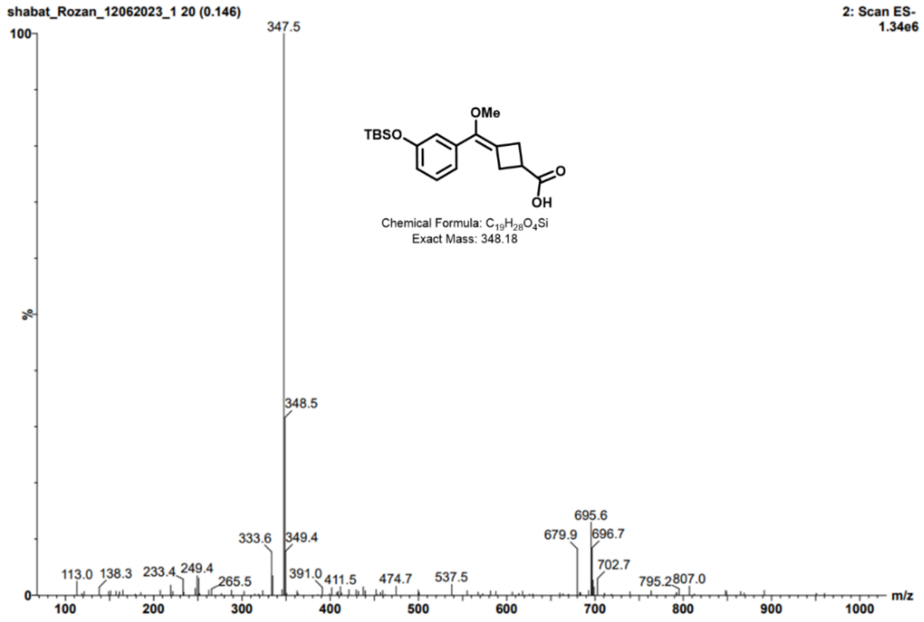

Diox 6

^{1}H-NMR

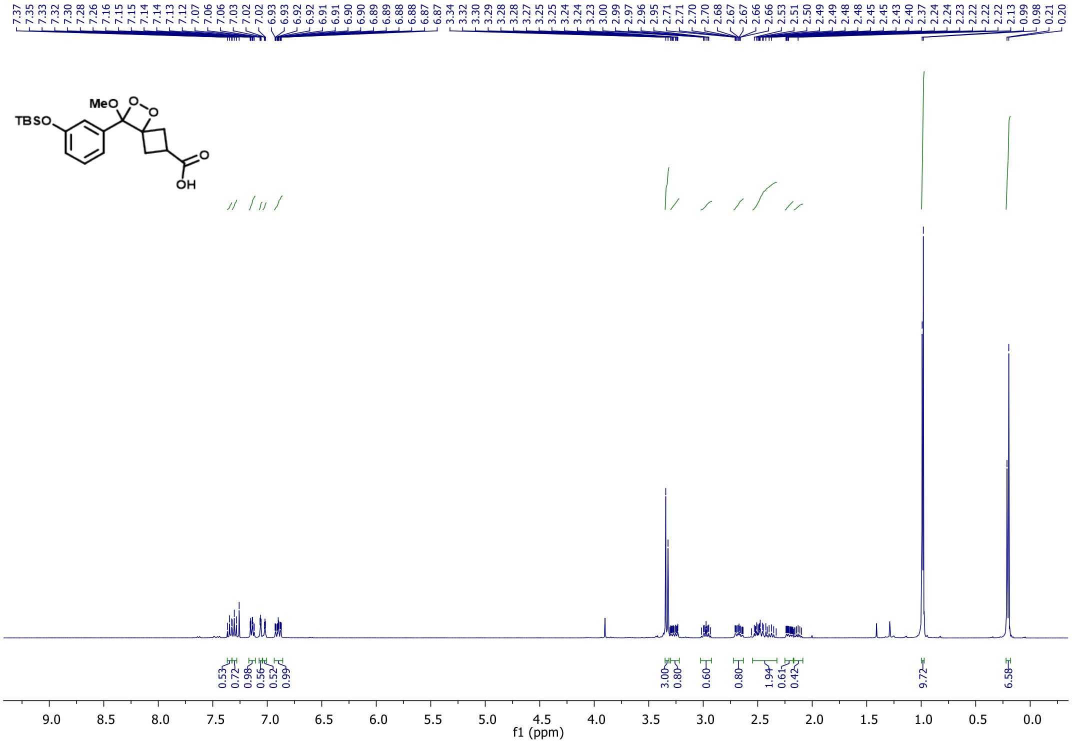

^{13}C-NMR

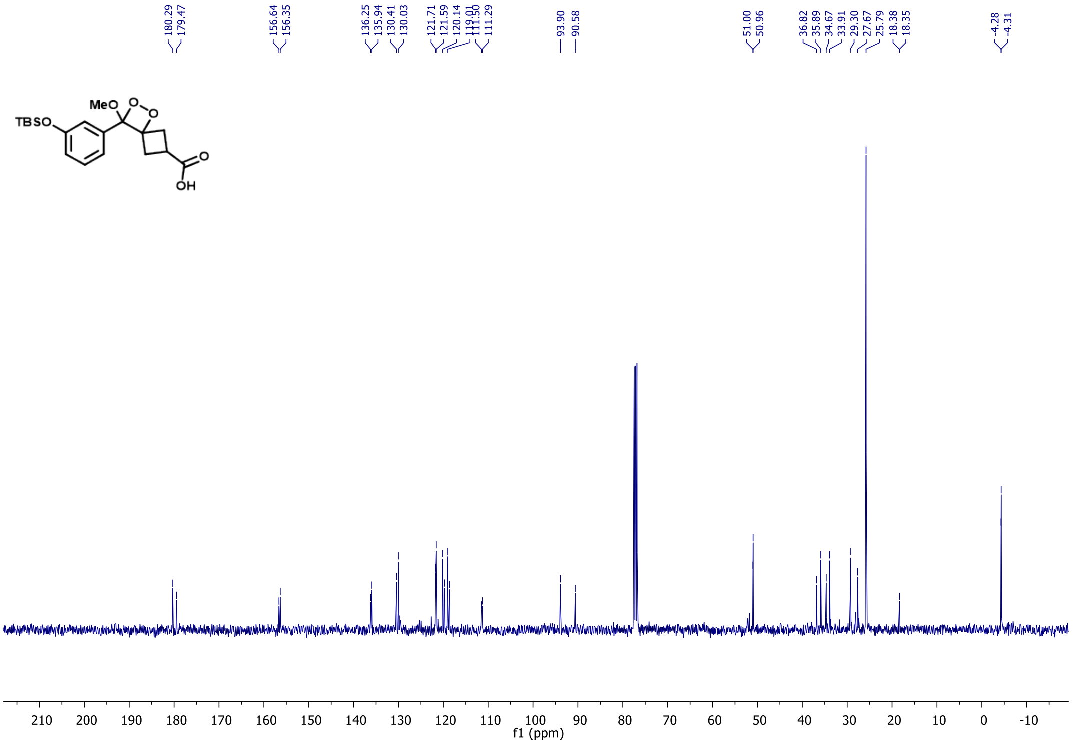

3D HPLC spectra (90-100% ACN in water, 0.1%TFA)

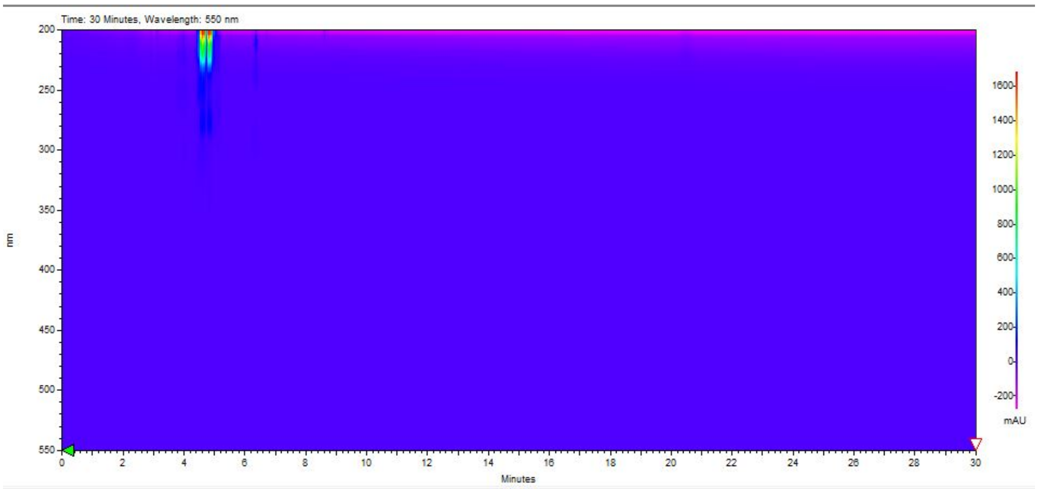

2D HPLC spectra (Absorbance measured at 277nm)

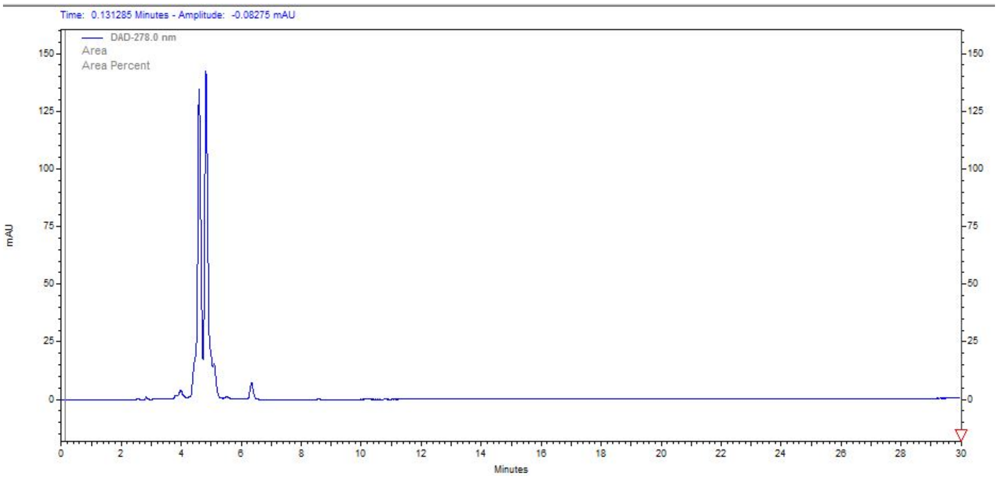

Mass spectra

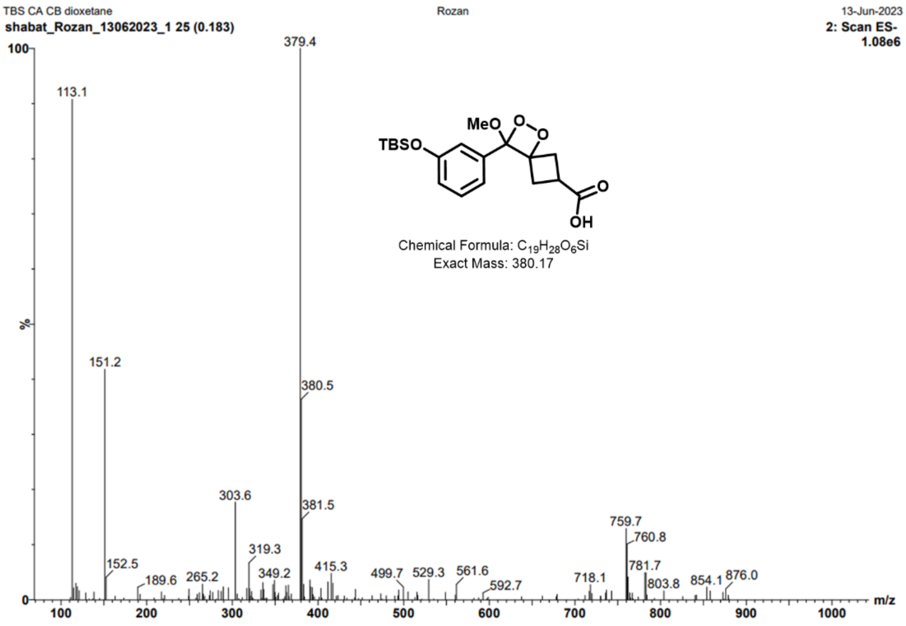

Compound 10a

^{1}H-NMR

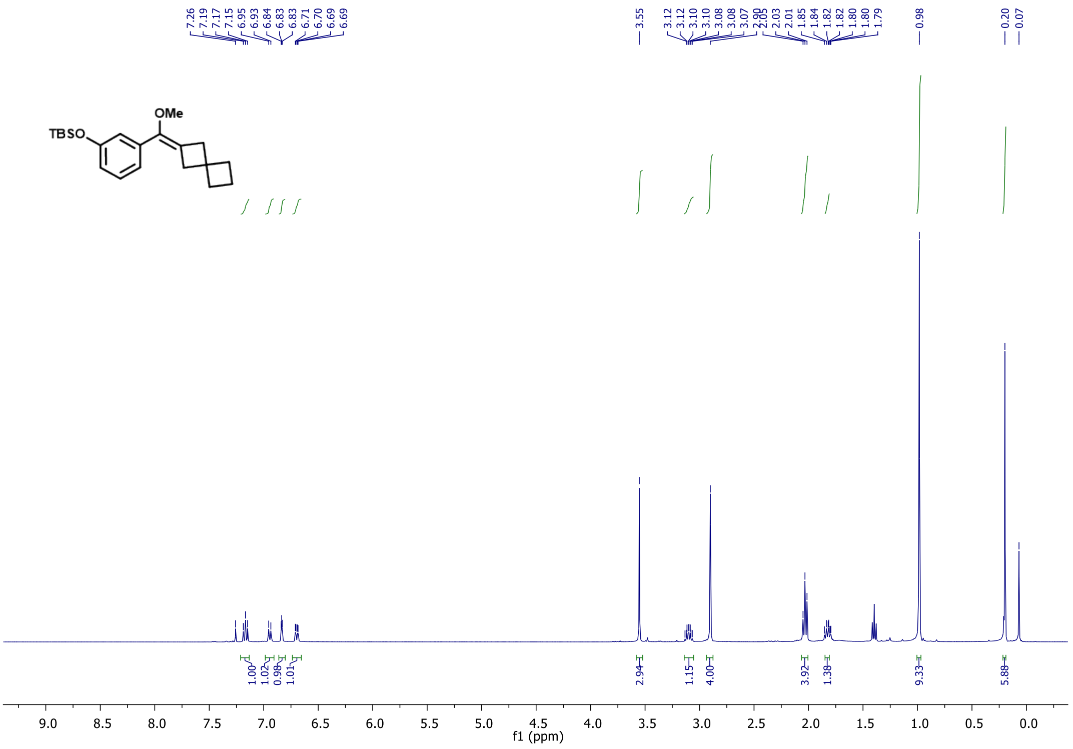

^{13}C-NMR

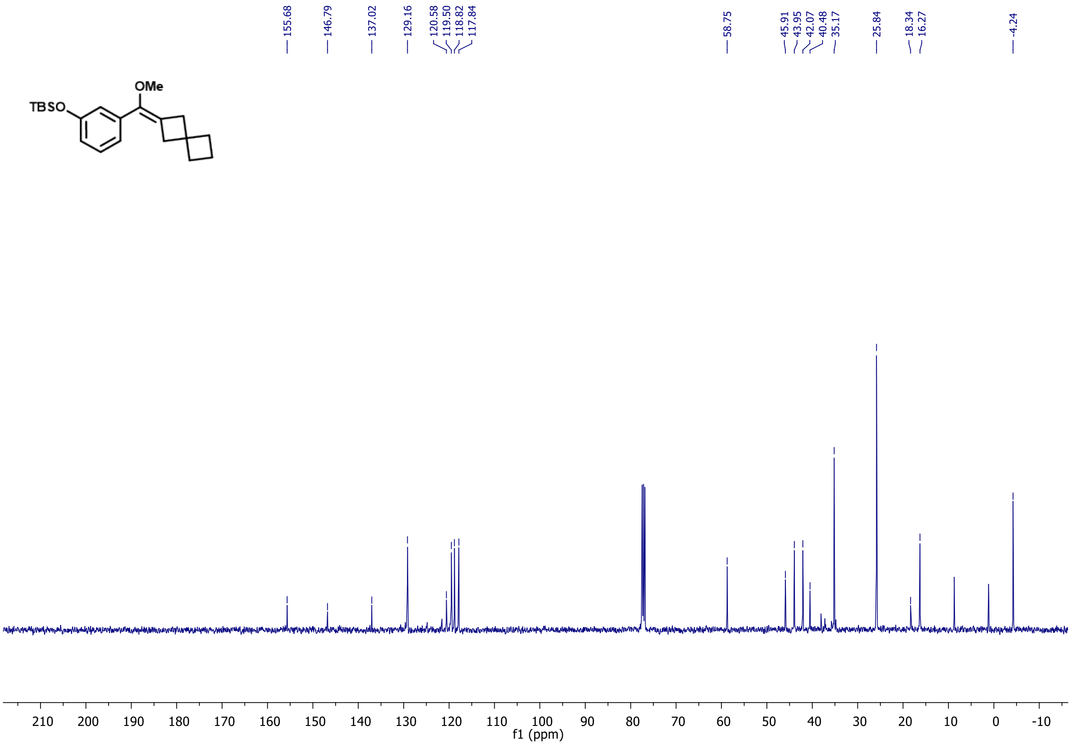

3D HPLC spectra (90-100% ACN in water, 0.1%TFA)

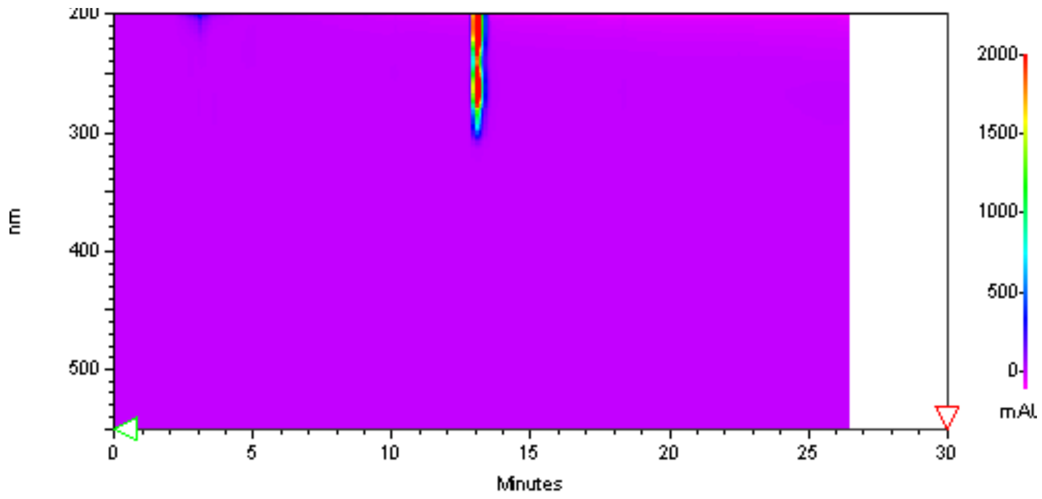

2D HPLC spectra (Absorbance measured at 275nm)

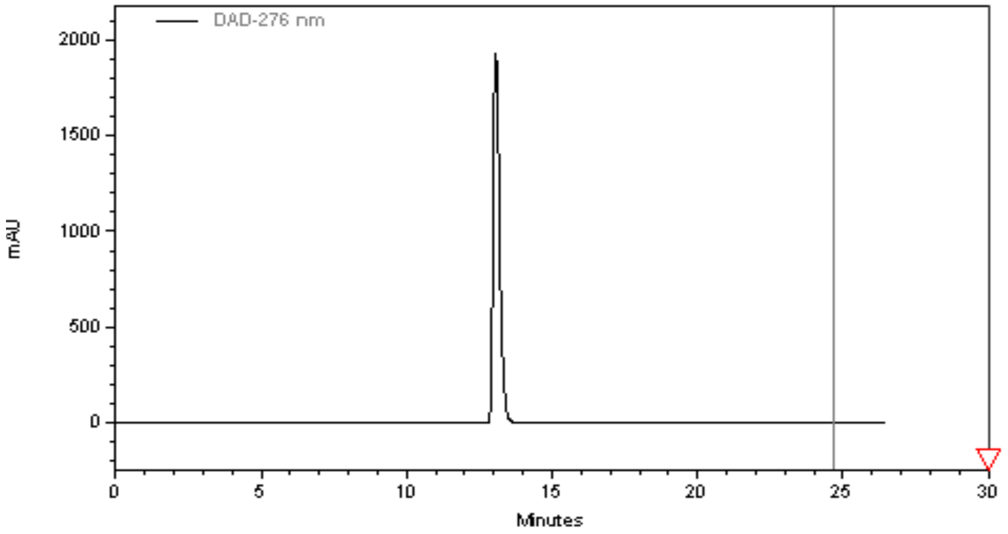

Diox 7

^{1}H-NMR

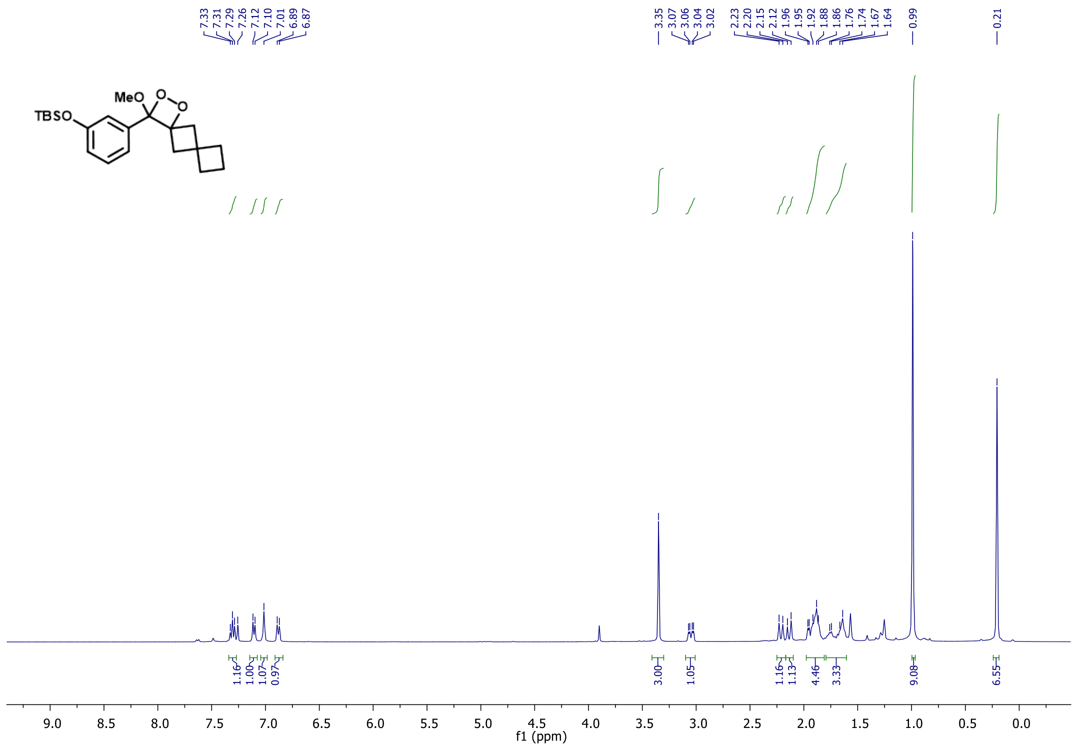

^{13}C-NMR

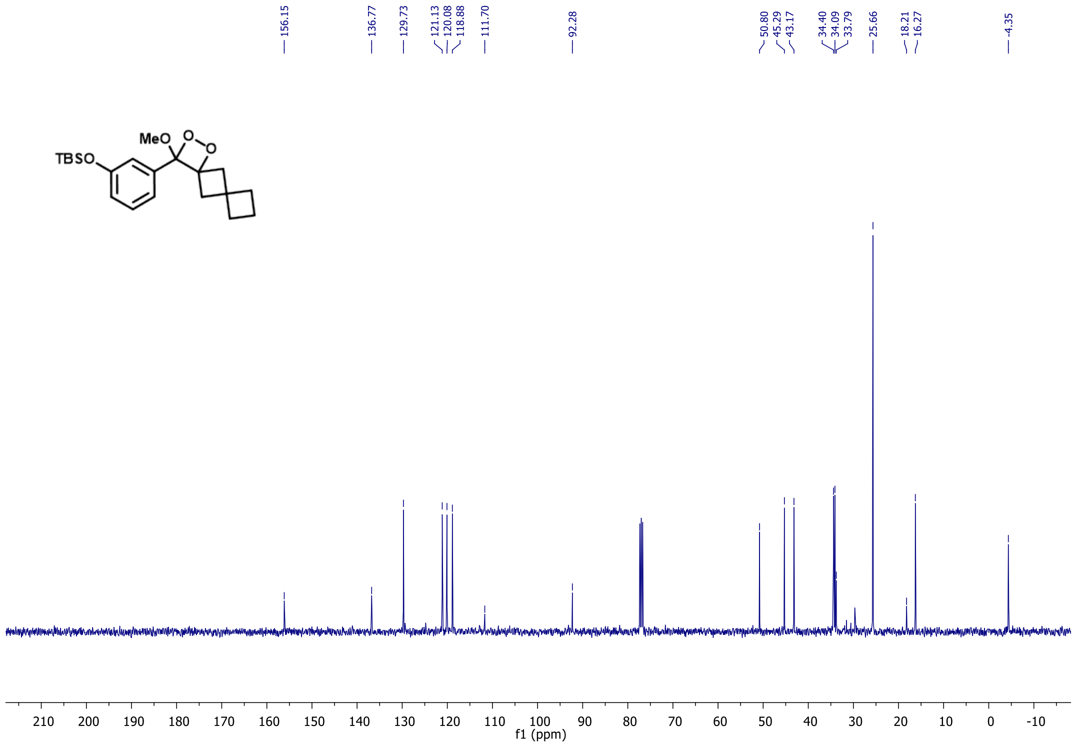

3D HPLC spectra (90-100% ACN in water, 0.1%TFA)

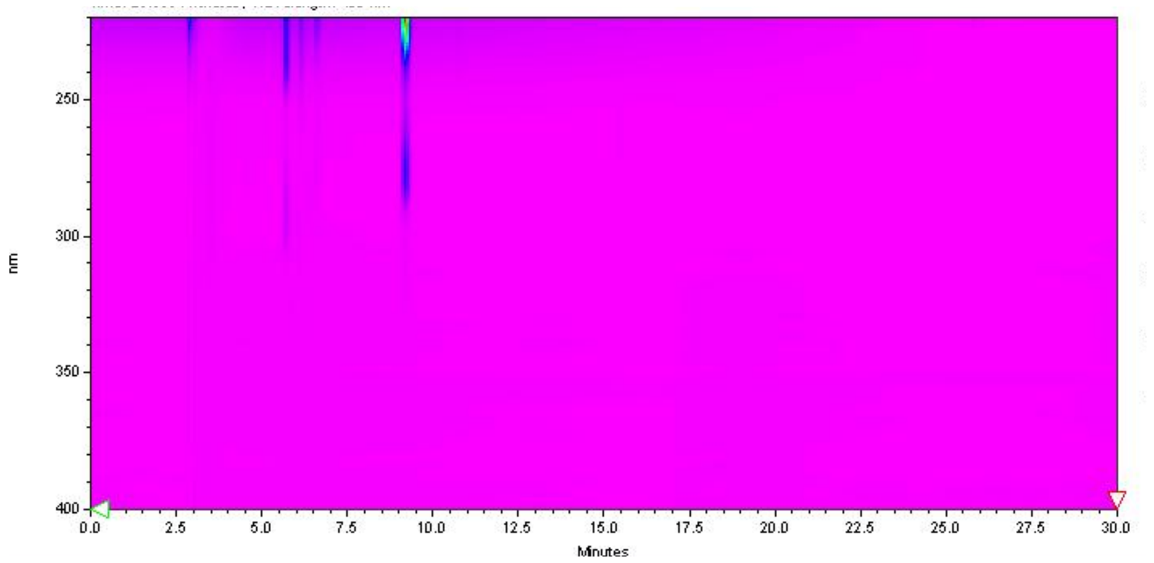

2D HPLC spectra (Absorbance measured at 277nm)

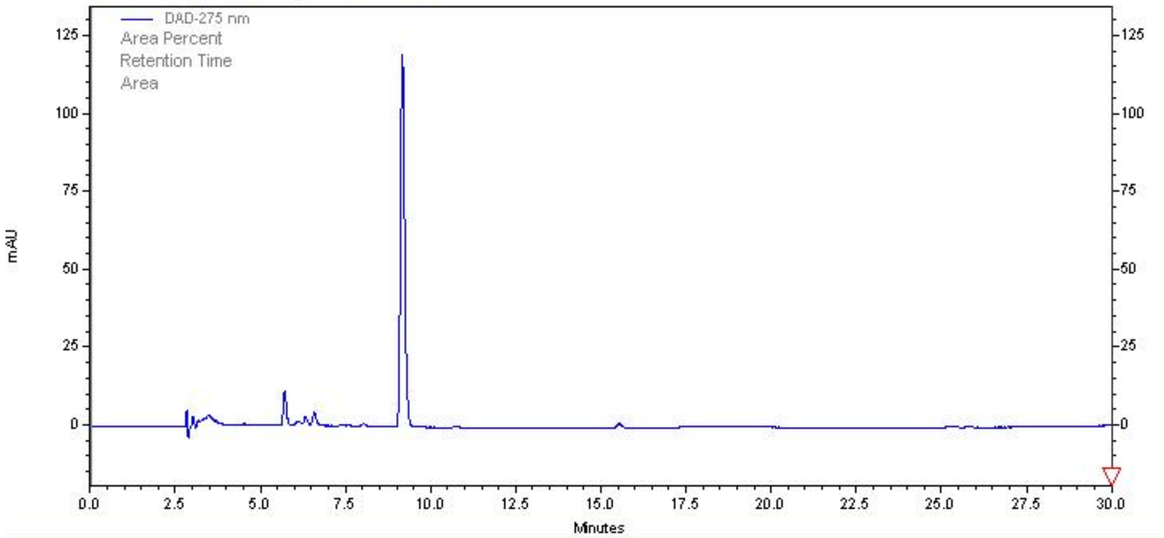

Compound 11a

^{1}H-NMR

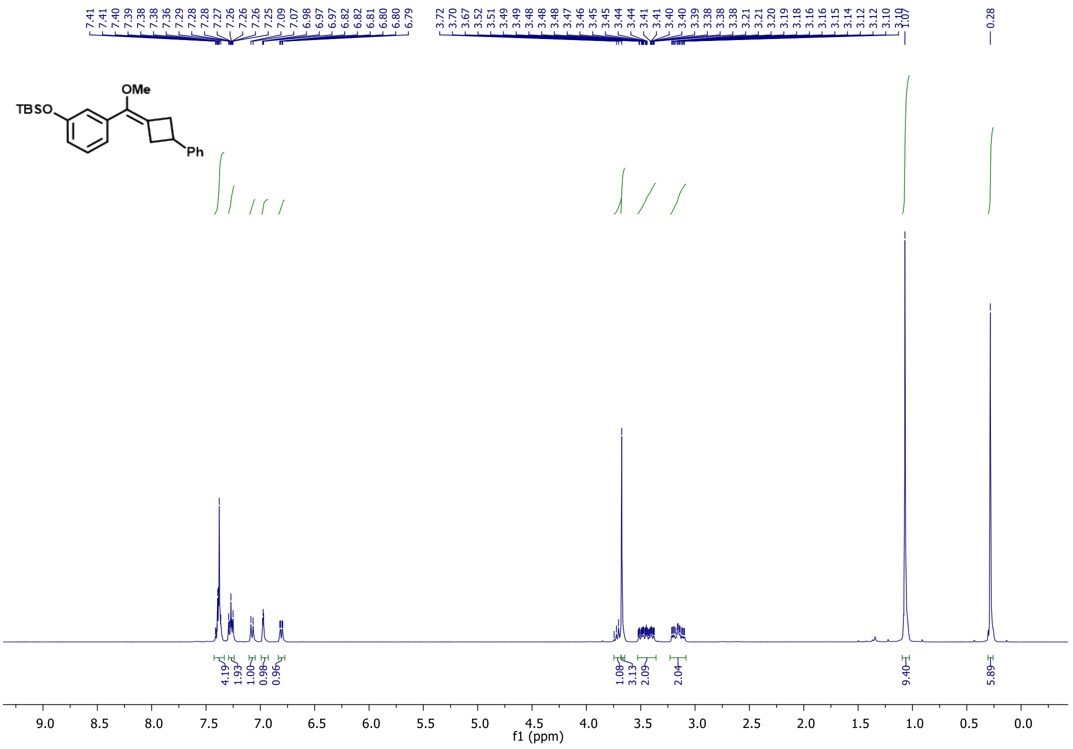

^{13}C-NMR

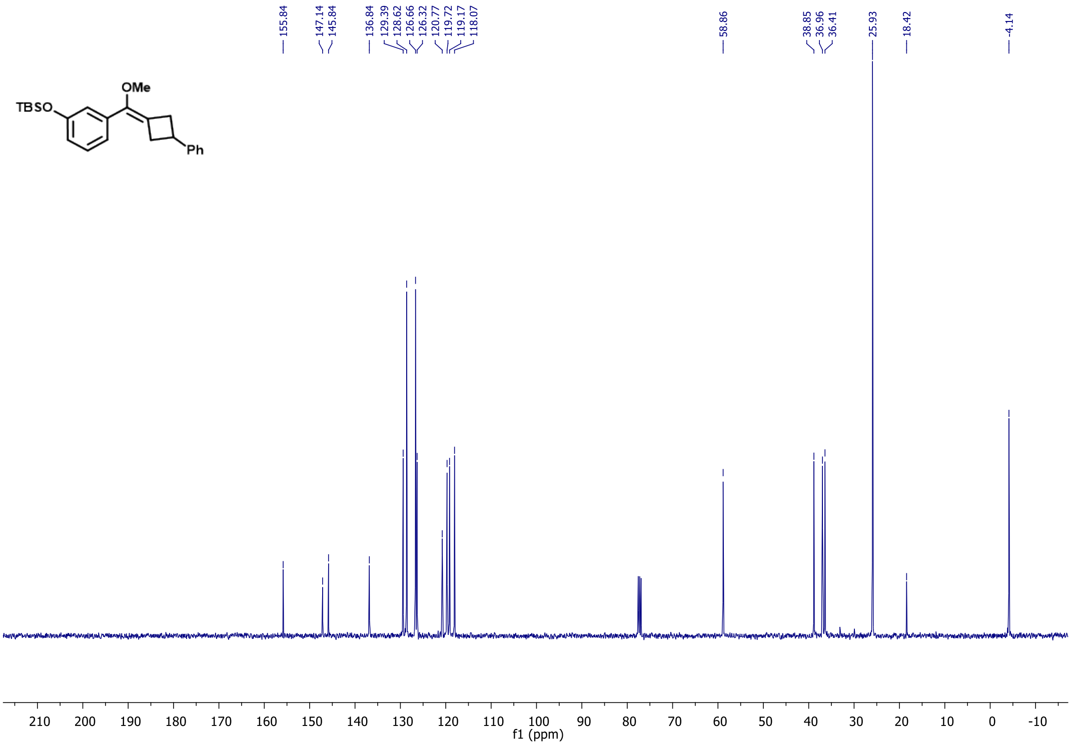

3D HPLC spectra (90-100% ACN in water, 0.1%TFA)

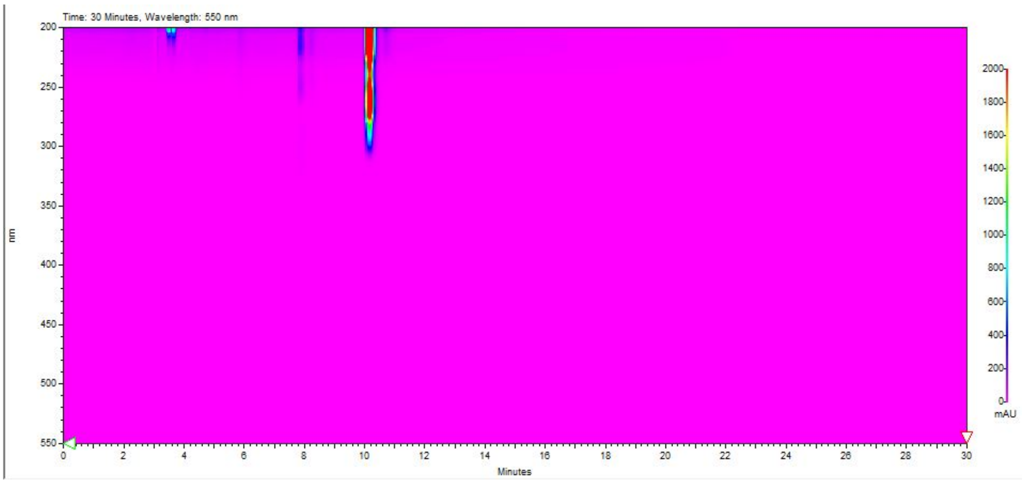

2D HPLC spectra (Absorbance measured at 275nm)

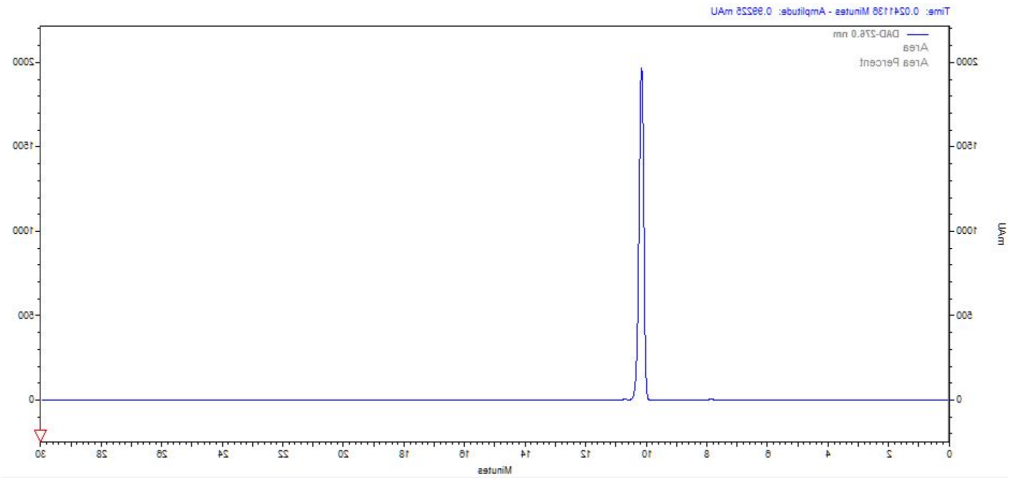

Mass spectra

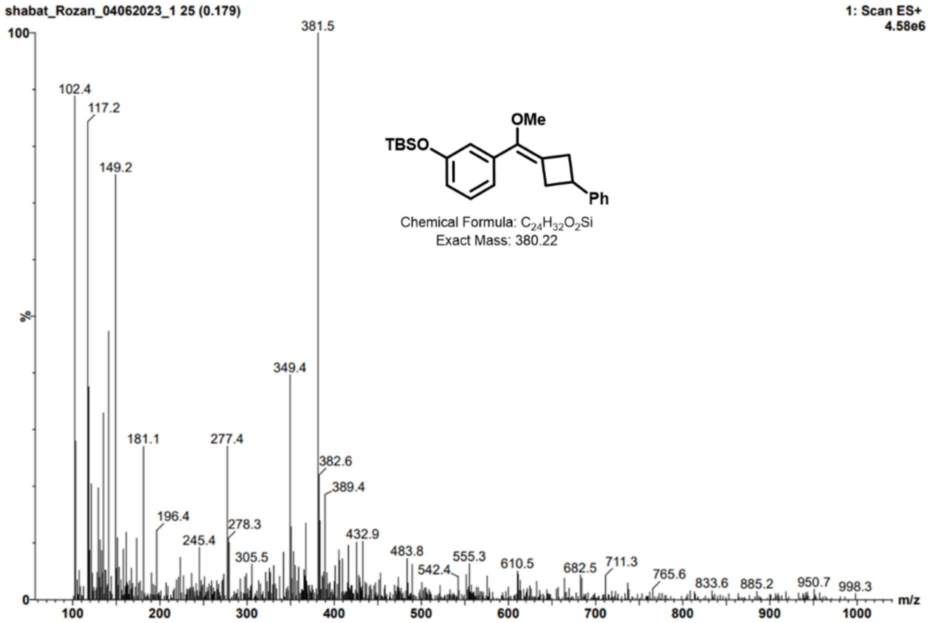

Diox 9

^{1}H-NMR

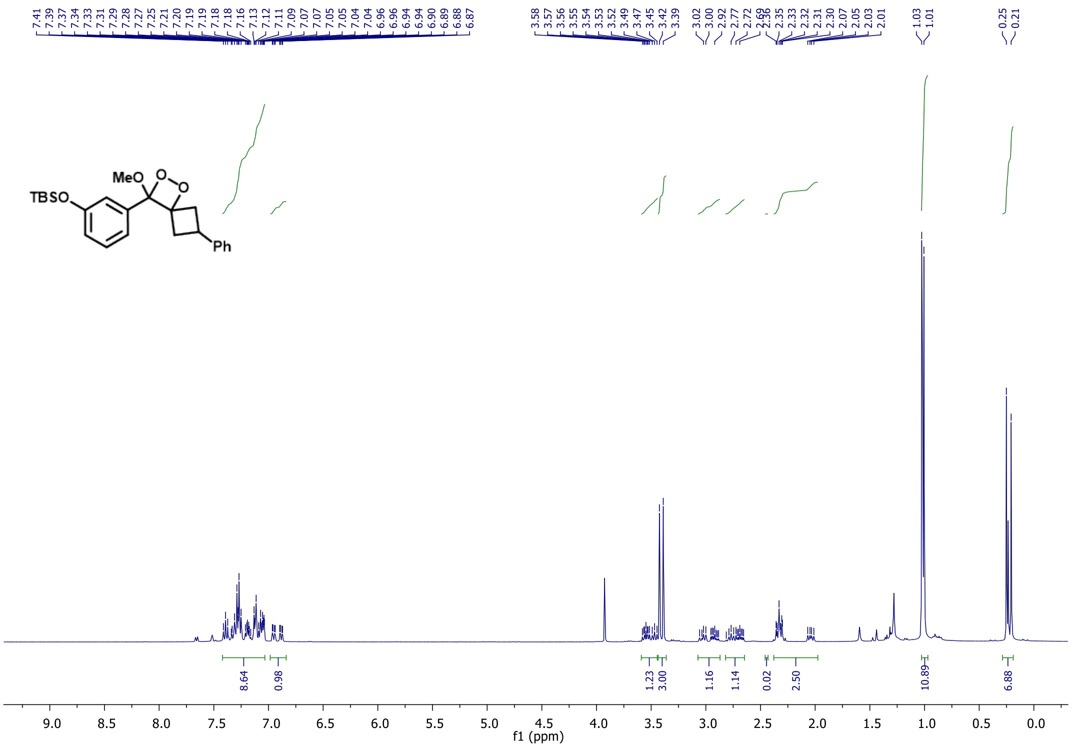

^{13}C-NMR

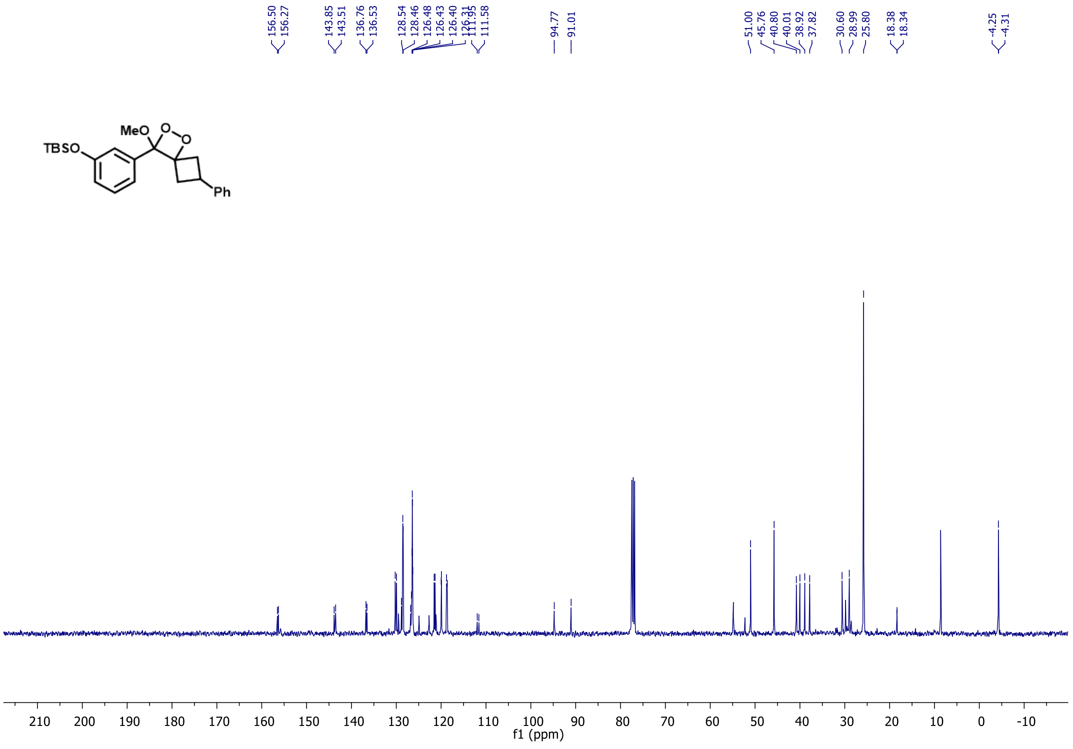

3D HPLC spectra (90-100% ACN in water, 0.1%TFA)

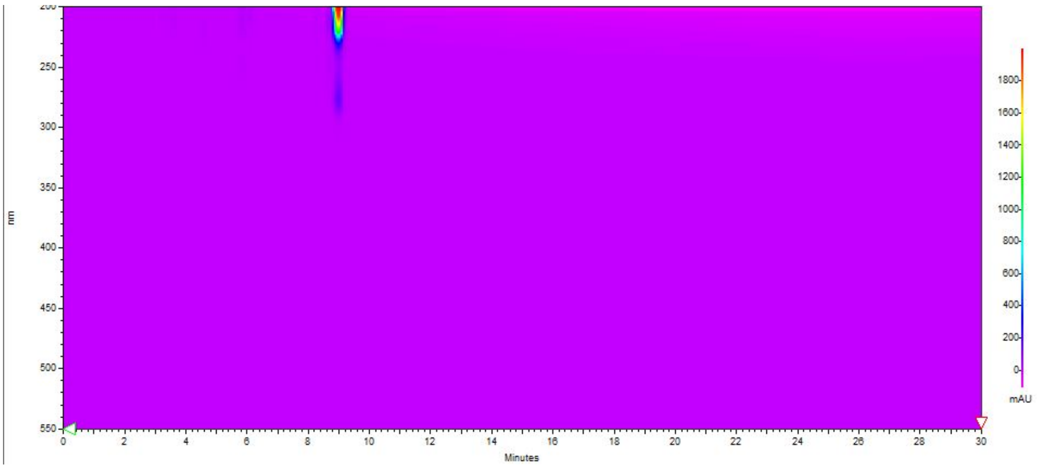

2D HPLC spectra (Absorbance measured at 277nm)

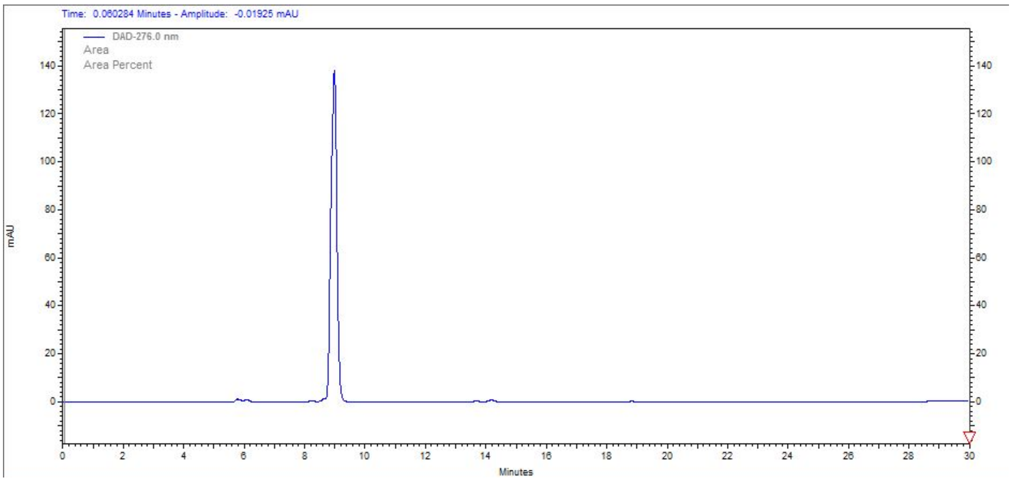

Compound 12a

^{1}H-NMR

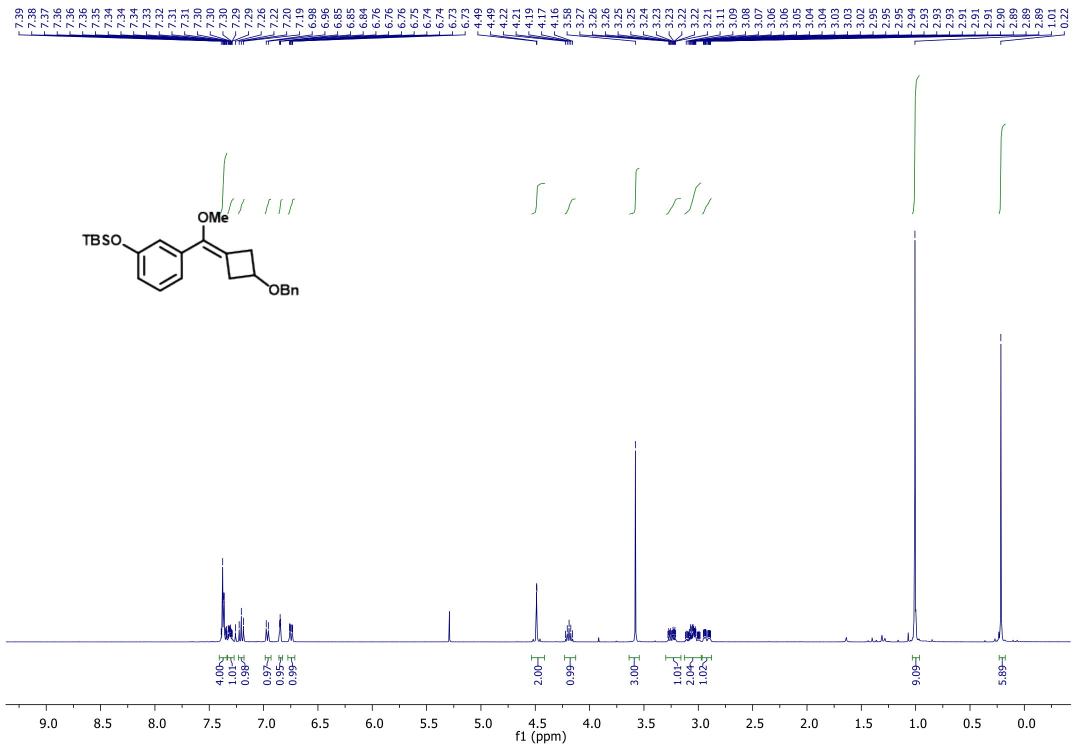

^{13}C-NMR

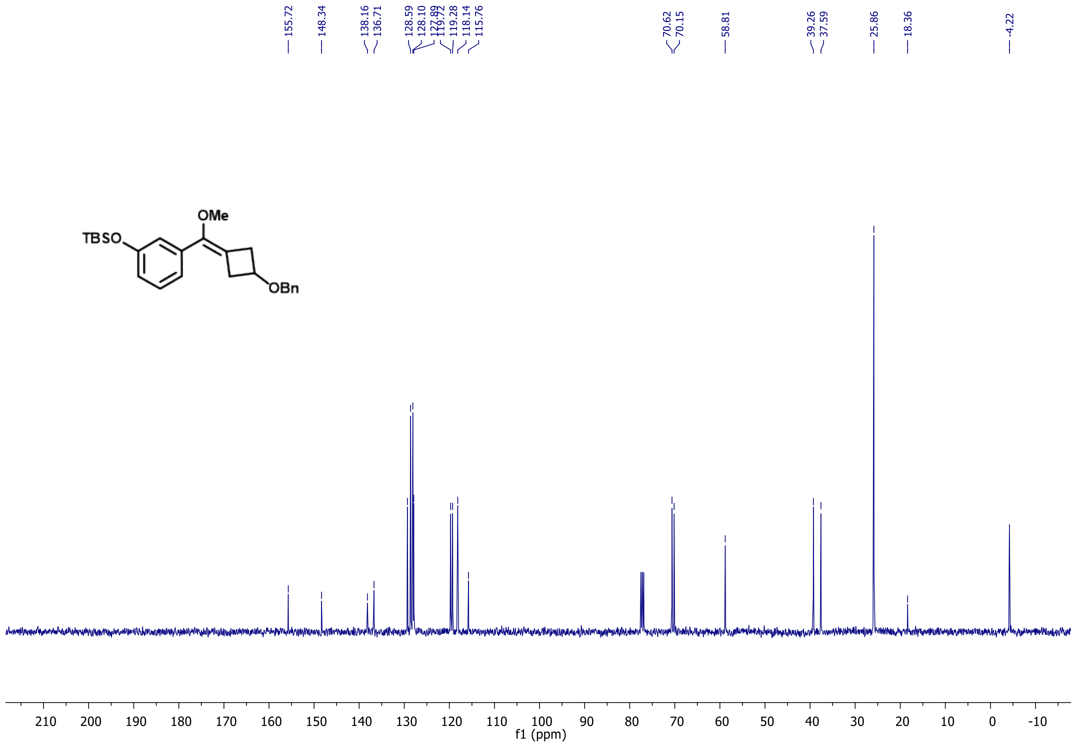

3D HPLC spectra (90-100% ACN in water, 0.1%TFA)

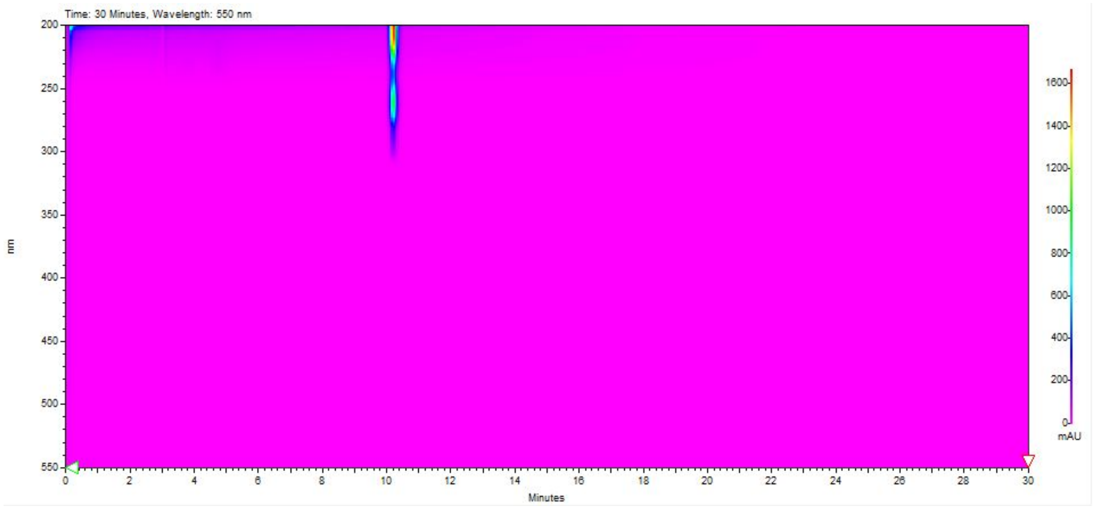

2D HPLC spectra (Absorbance measured at 275nm)

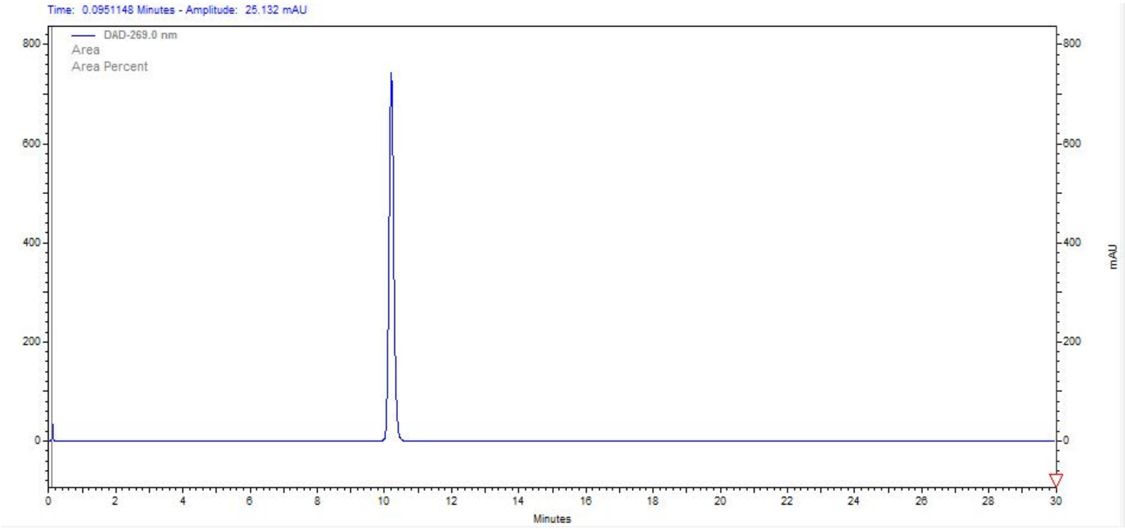

Diox 10

^{1}H-NMR

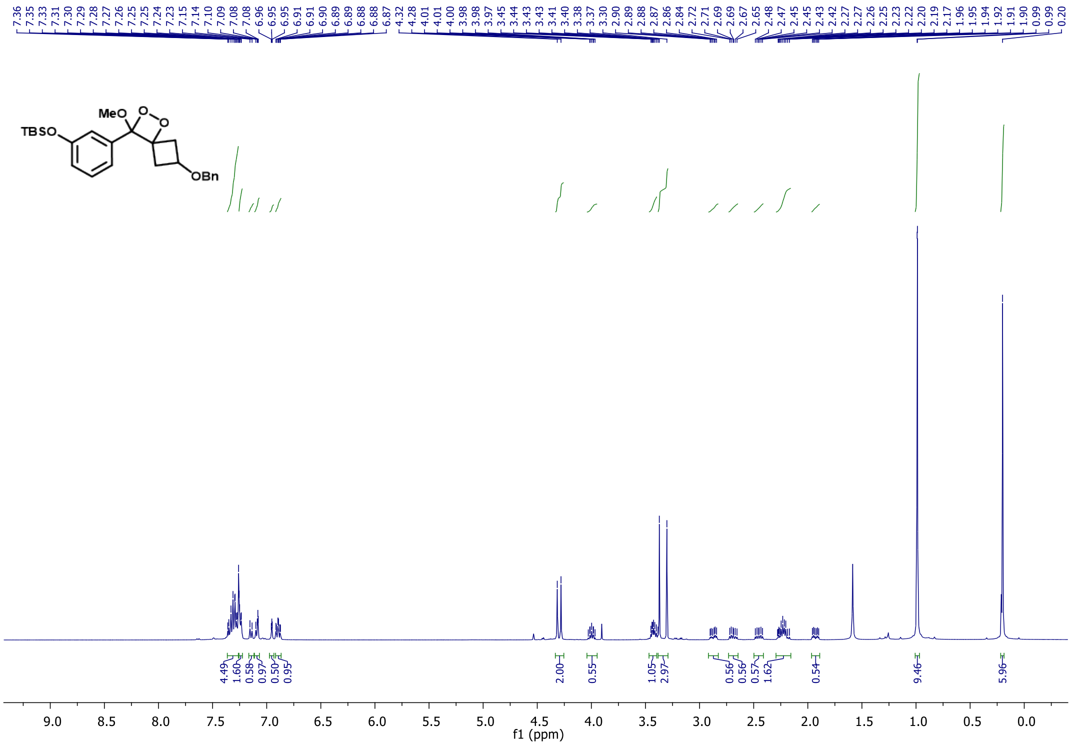

^{13}C-NMR

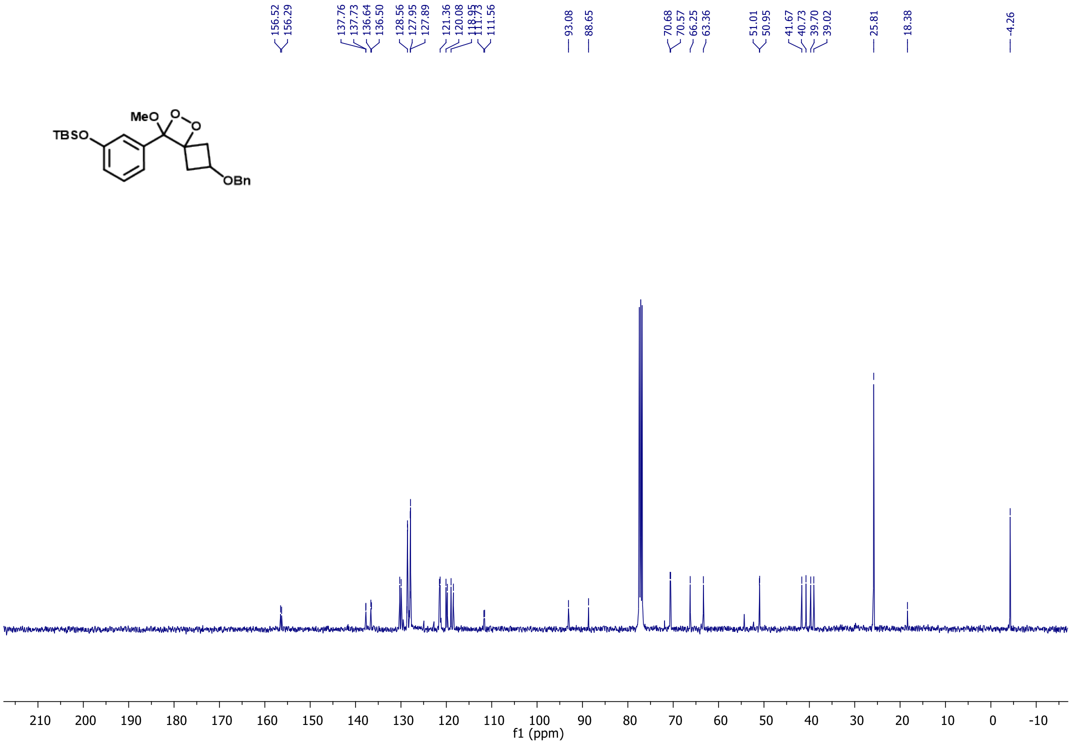

3D HPLC spectra (90-100% ACN in water, 0.1%TFA)

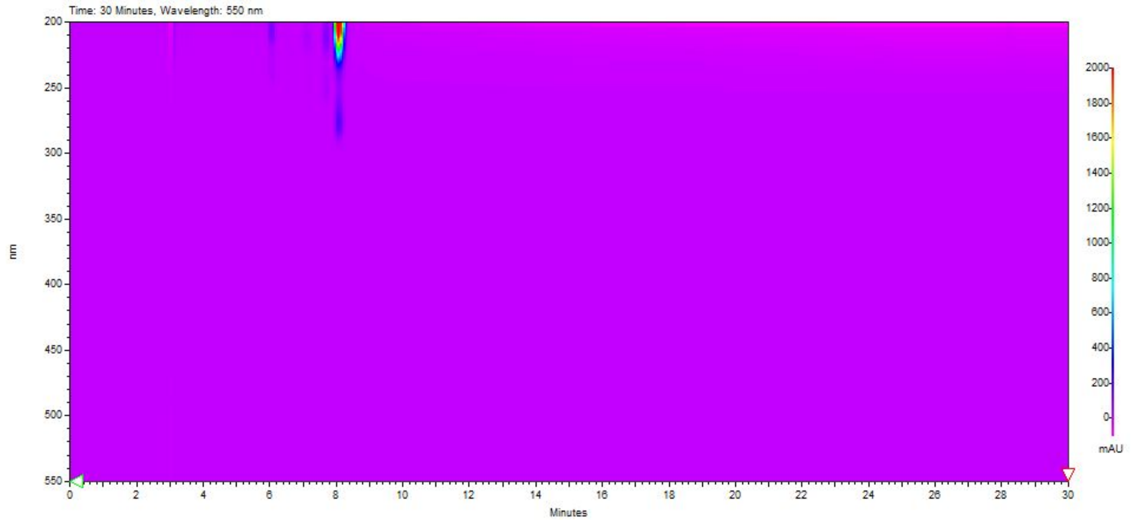

2D HPLC spectra (Absorbance measured at 277nm)

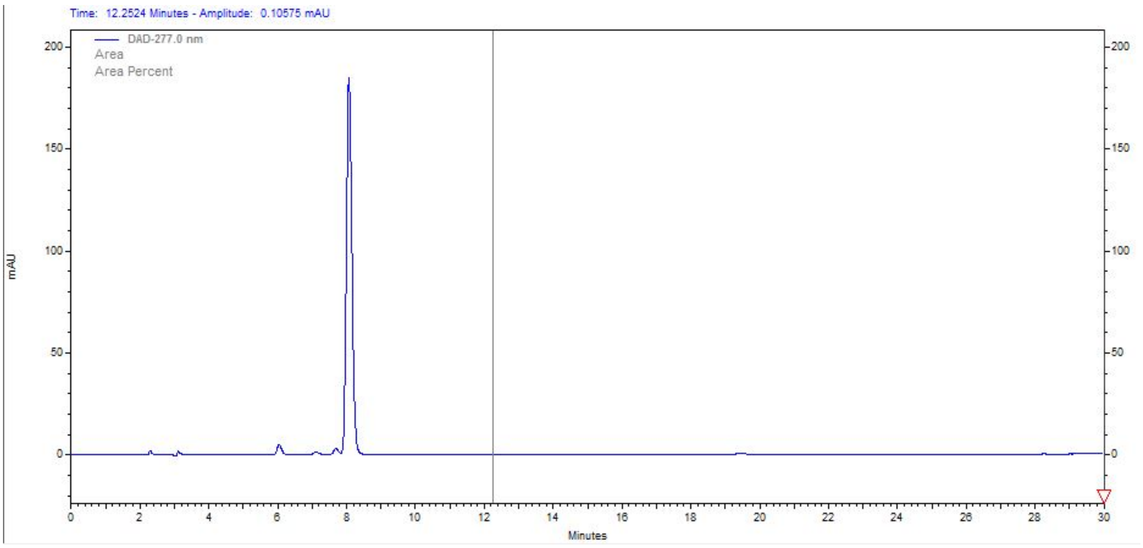

Compound 13a

^{1}H-NMR

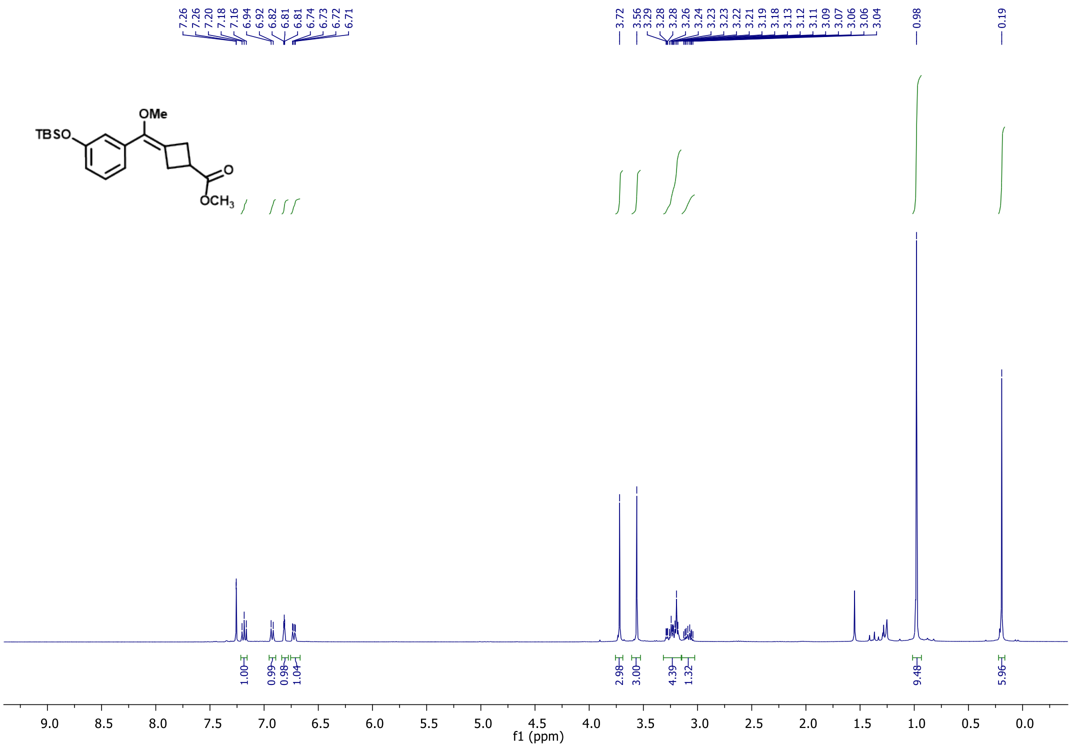

^{13}C-NMR

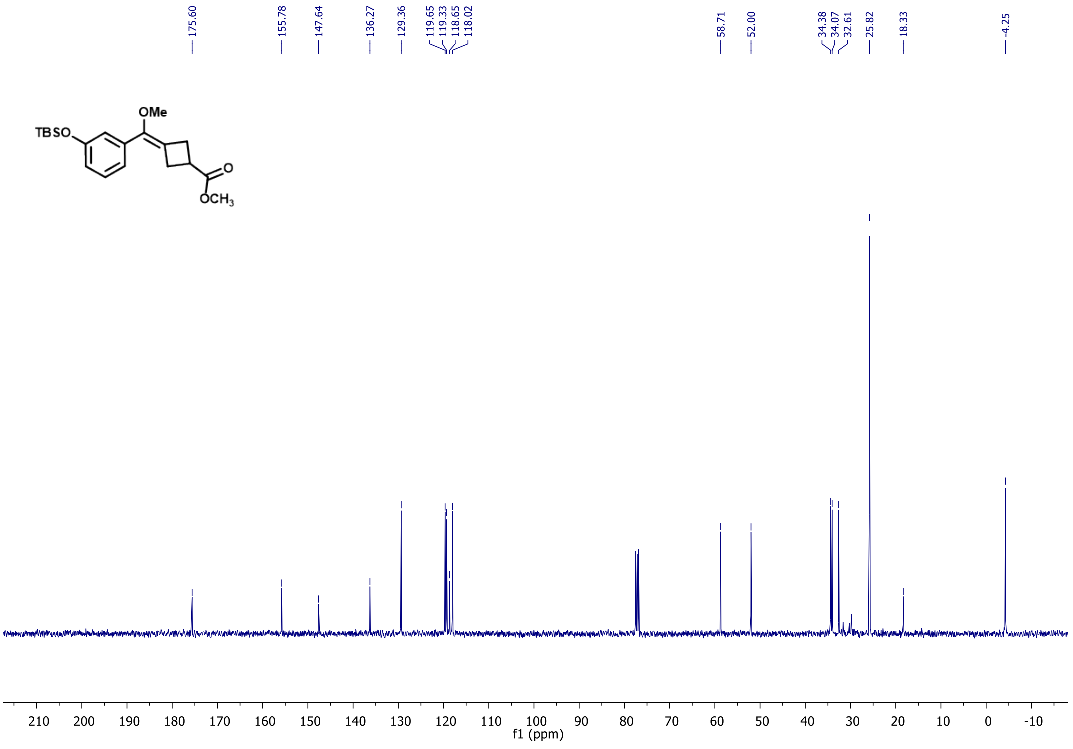

3D HPLC spectra (90-100% ACN in water, 0.1%TFA)

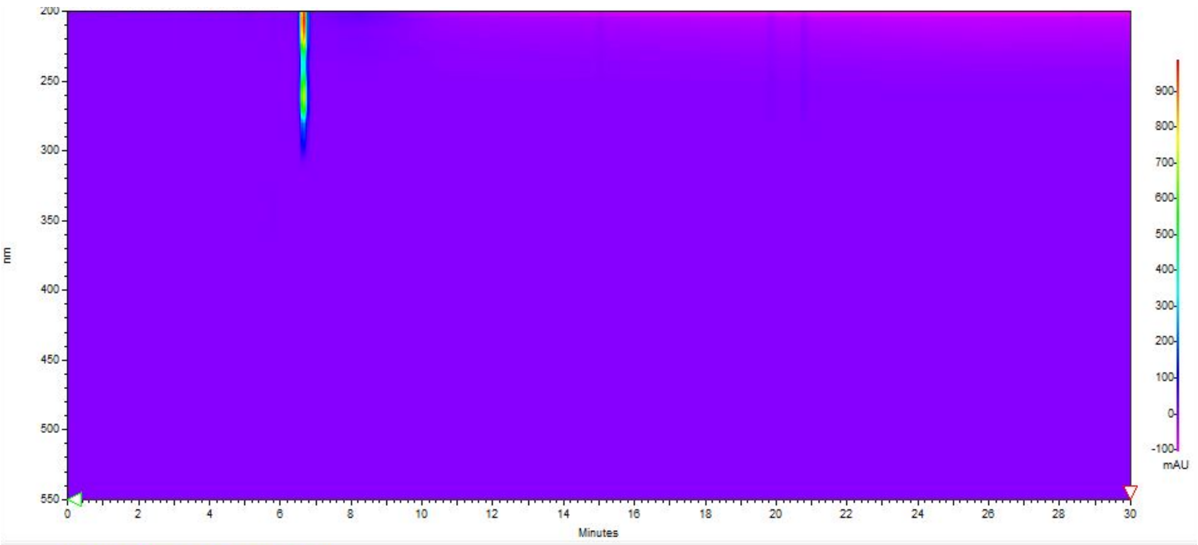

2D HPLC spectra (Absorbance measured at 275nm)

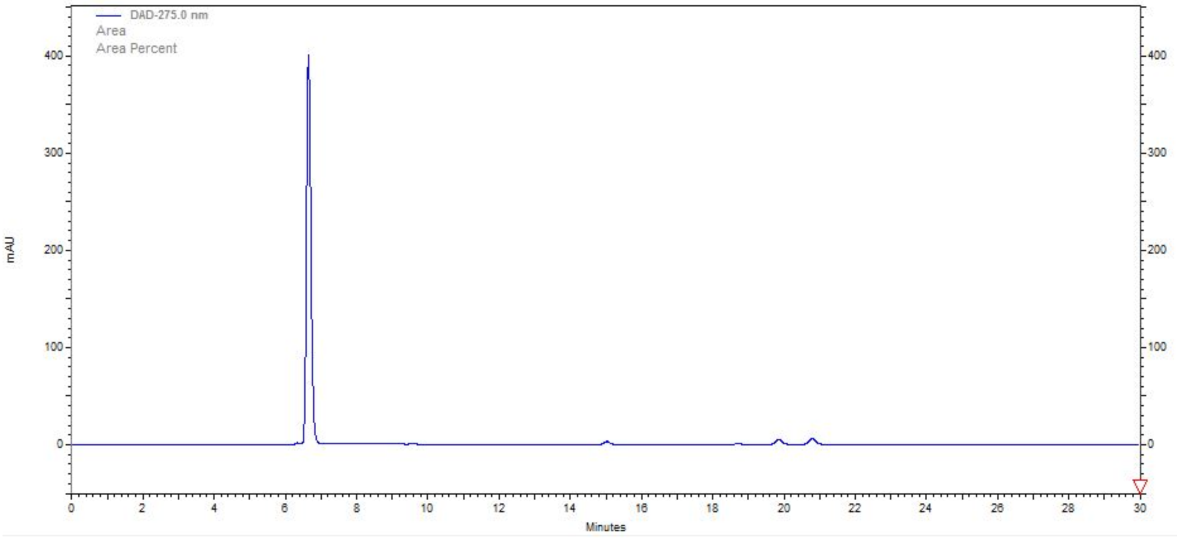

Diox 11

^{1}H-NMR

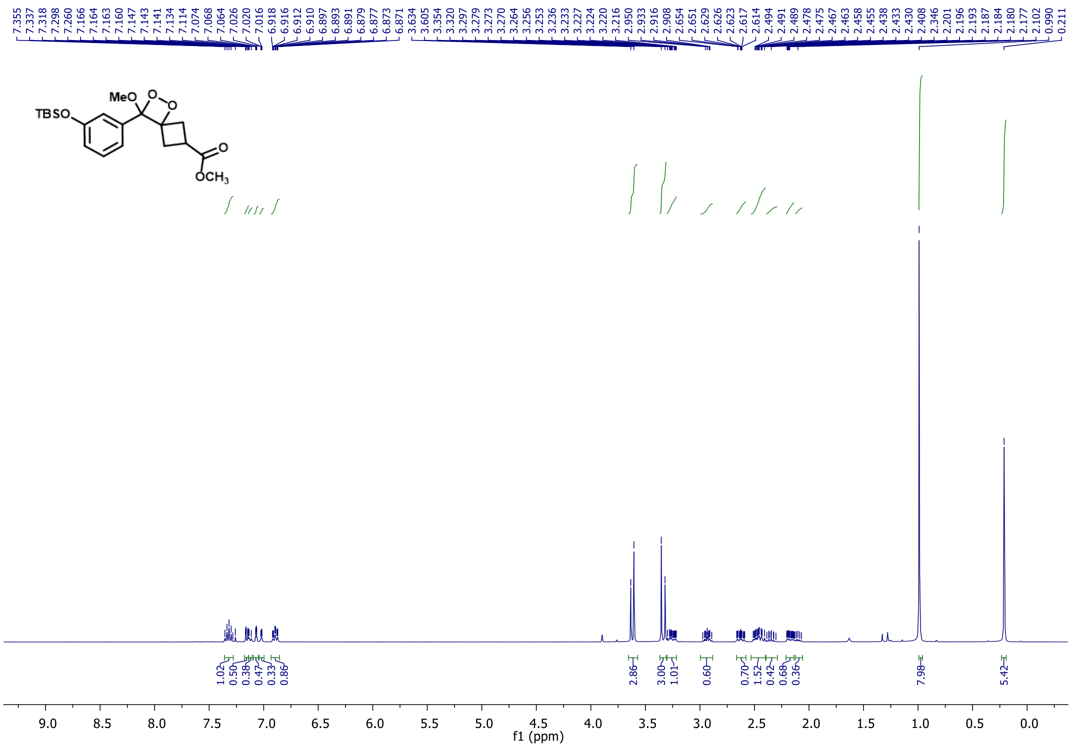

^{13}C-NMR

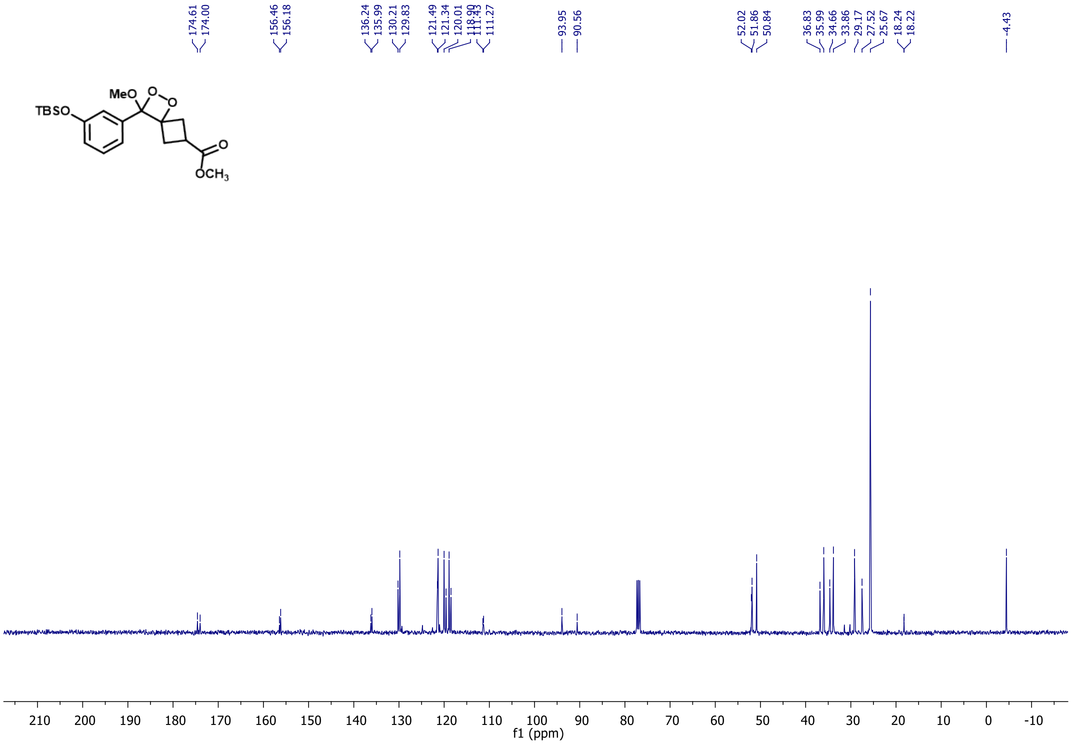

3D HPLC spectra (90-100% ACN in water, 0.1%TFA)

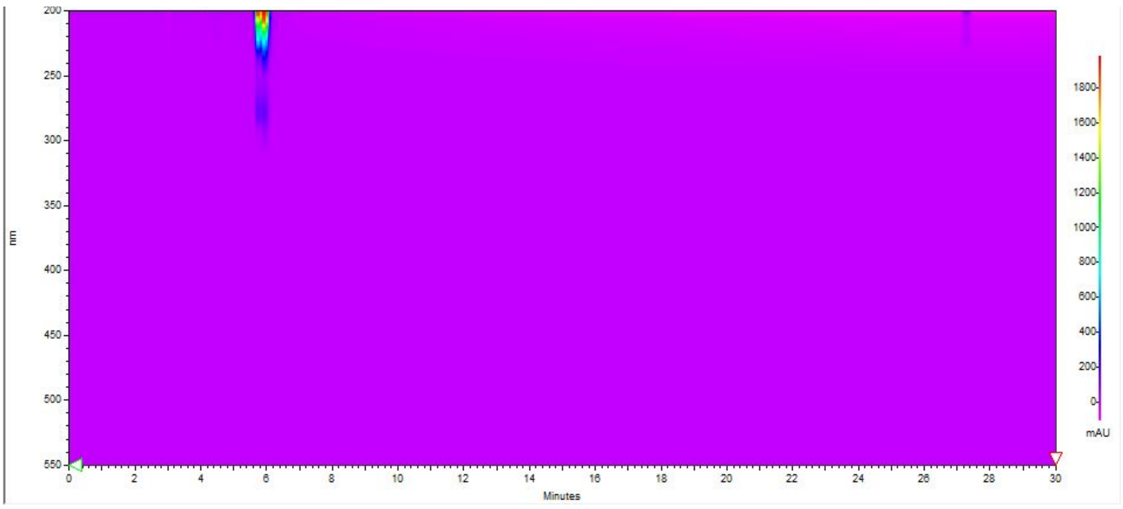

2D HPLC spectra (Absorbance measured at 275nm)

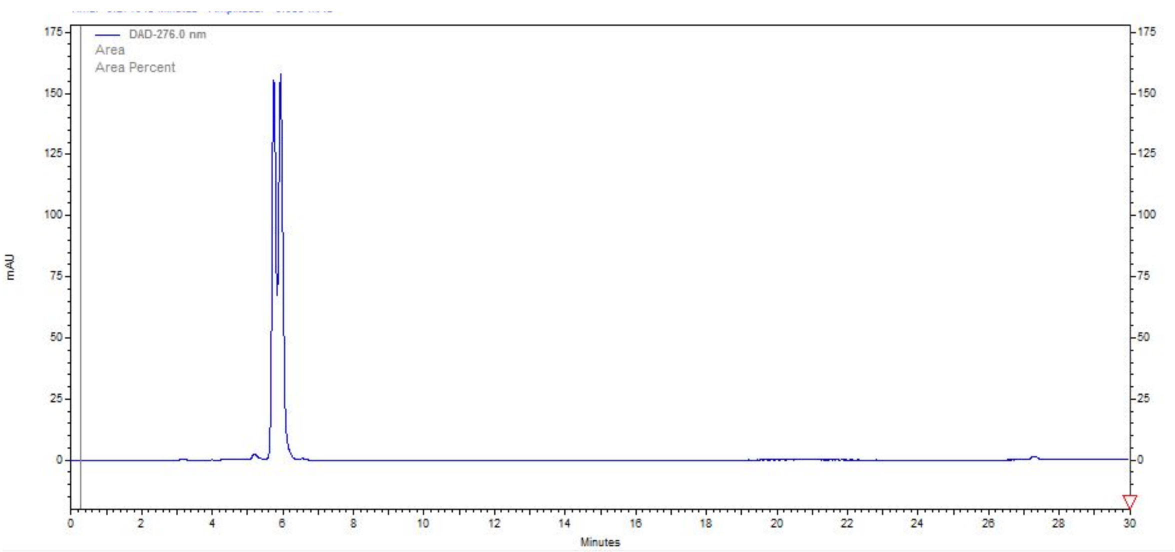

Compound 14a

^{1}H-NMR

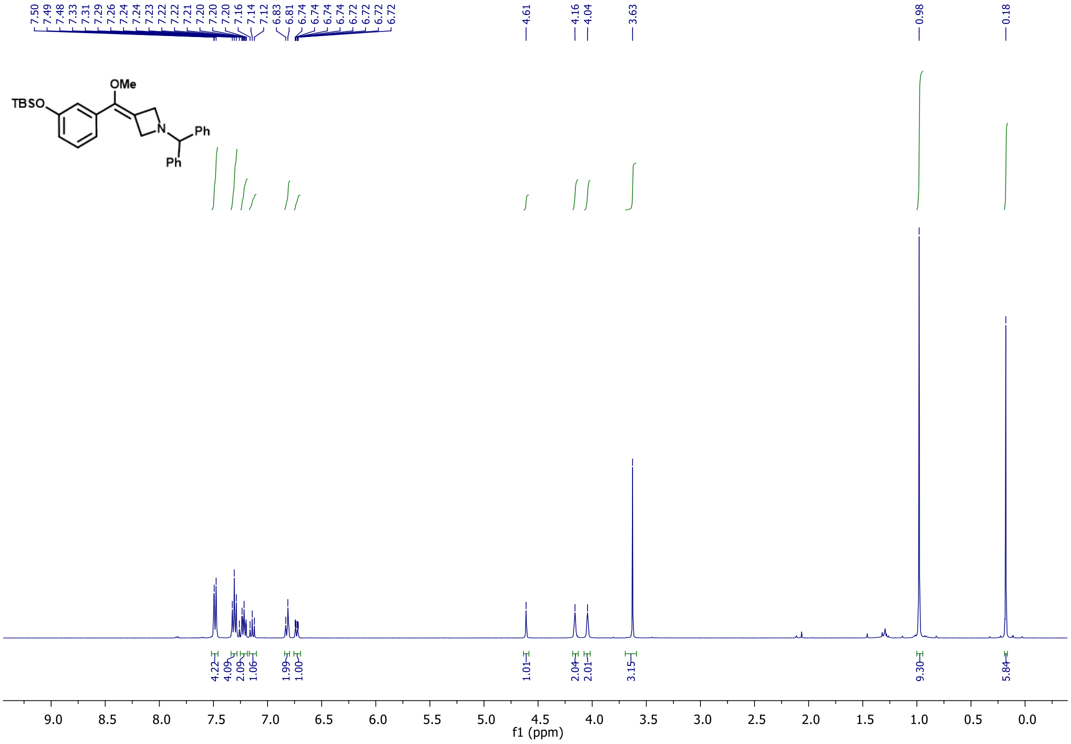

^{13}C-NMR

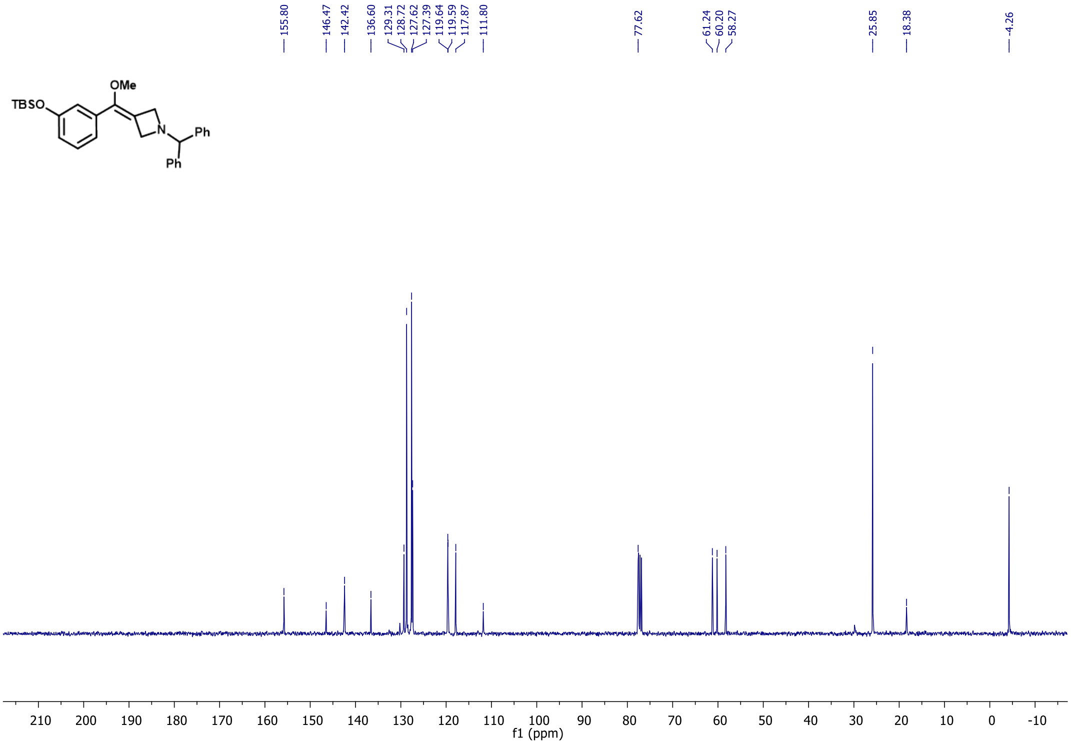

3D HPLC spectra (30-100% ACN in water, 0.1%TFA)

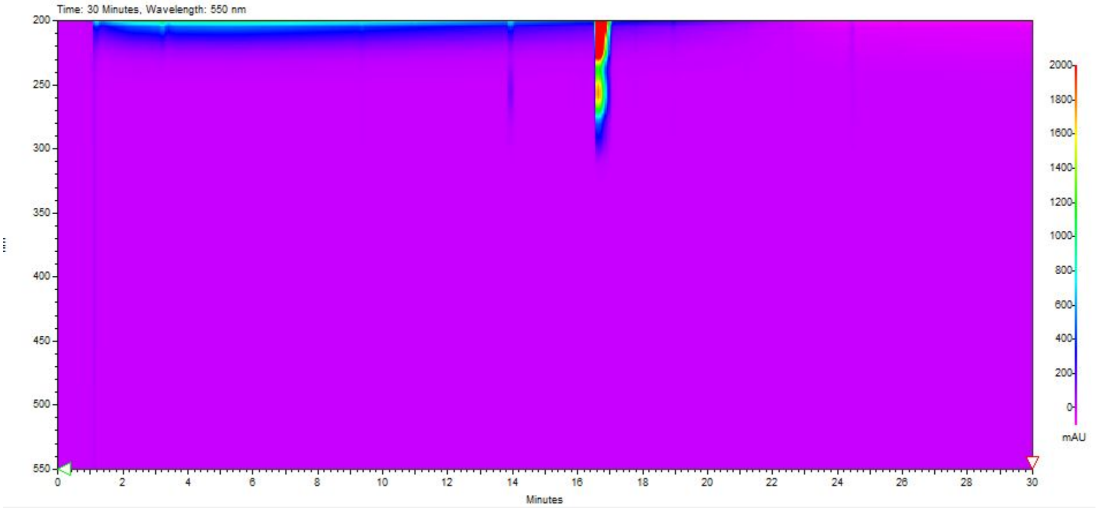

2D HPLC spectra (Absorbance measured at 275nm)

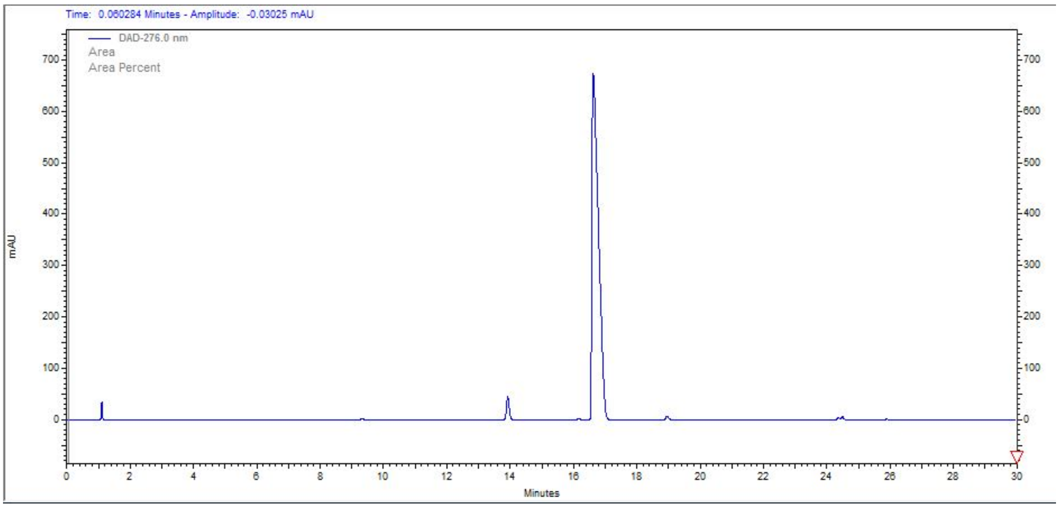

Mass spectra

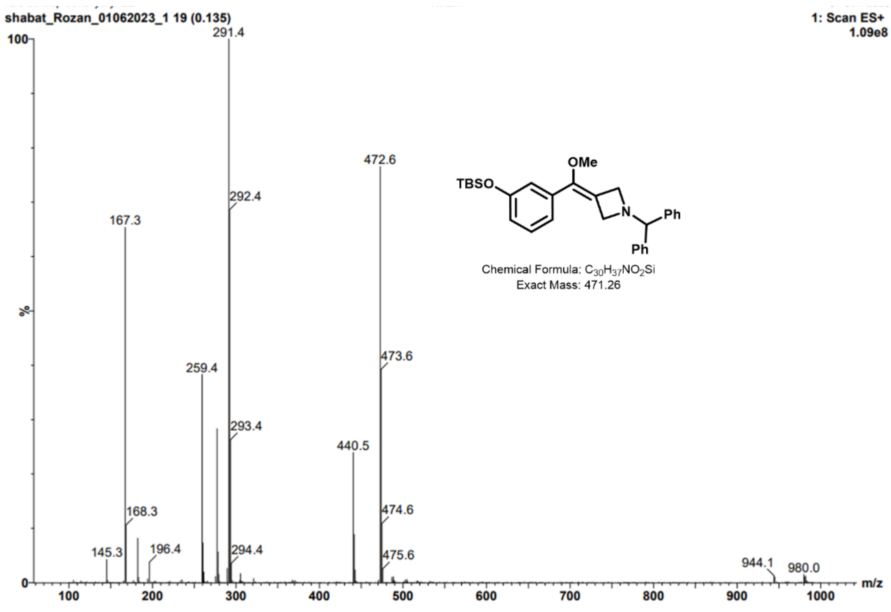

Diox 12

^{1}H-NMR

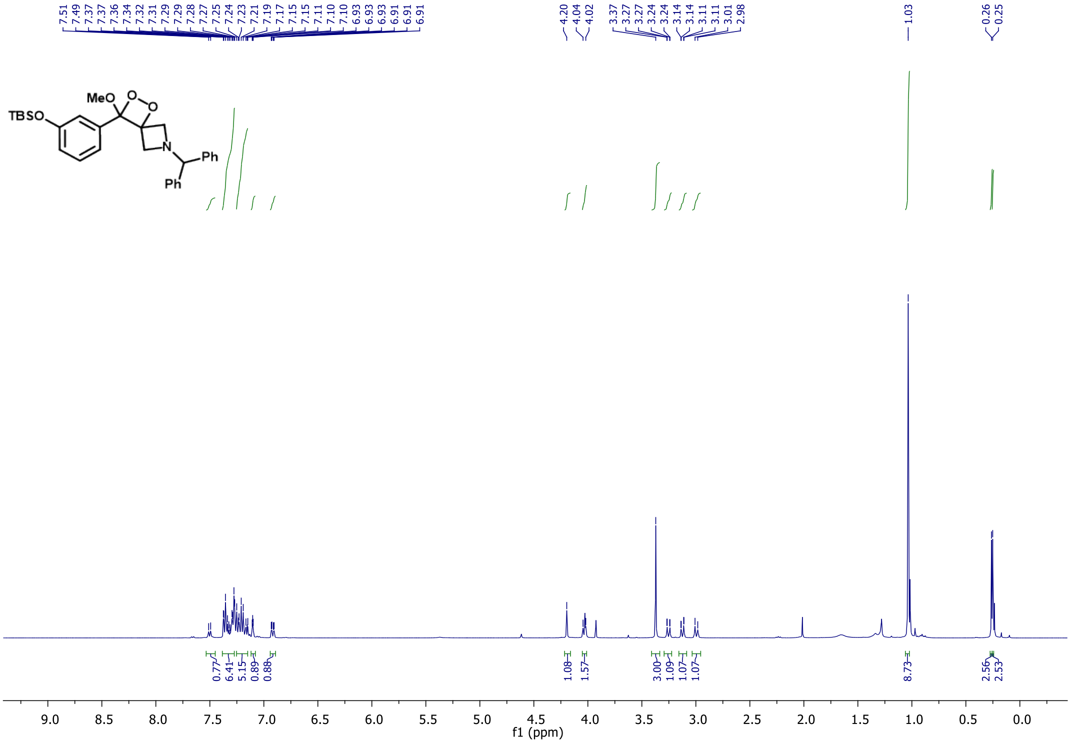

^{13}C-NMR

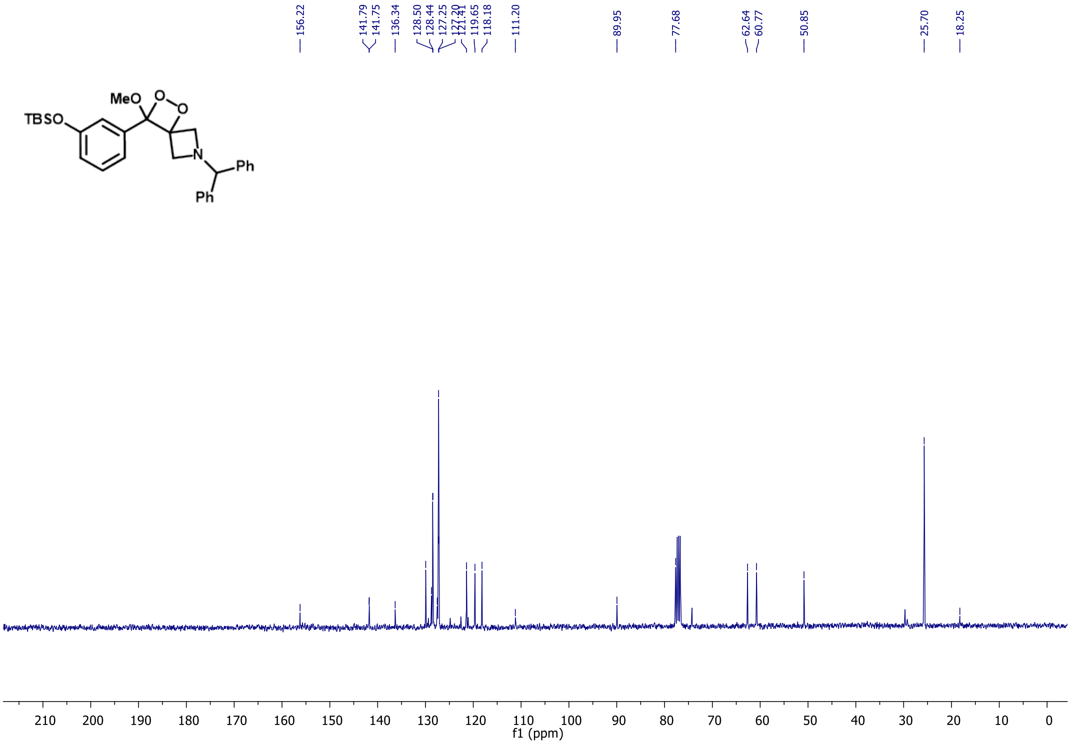

3D HPLC spectra (90-100% ACN in water, 0.1%TFA)

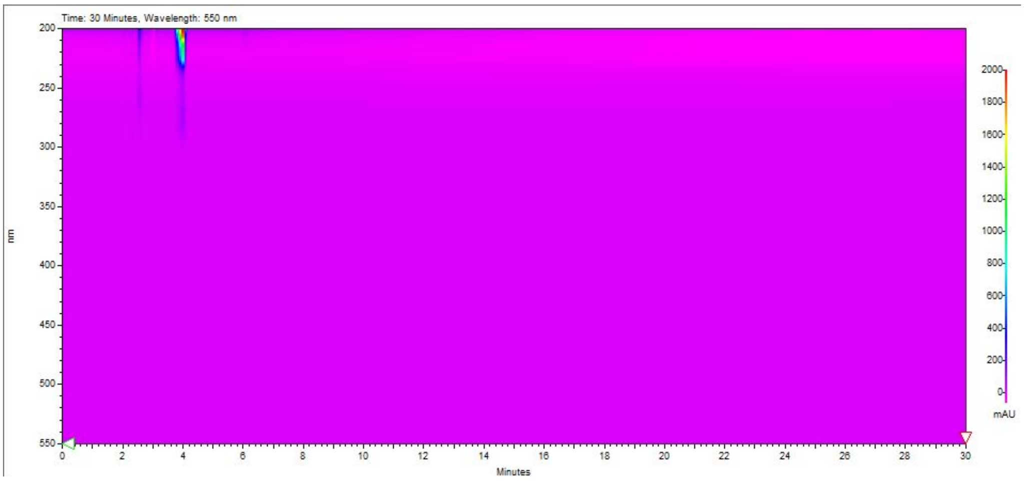

2D HPLC spectra (Absorbance measured at 277nm)

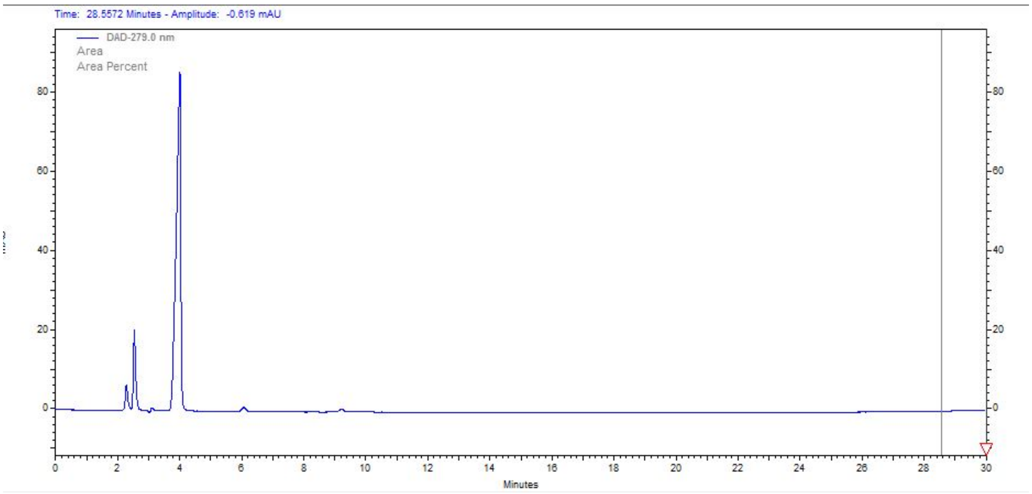

Mass spectra

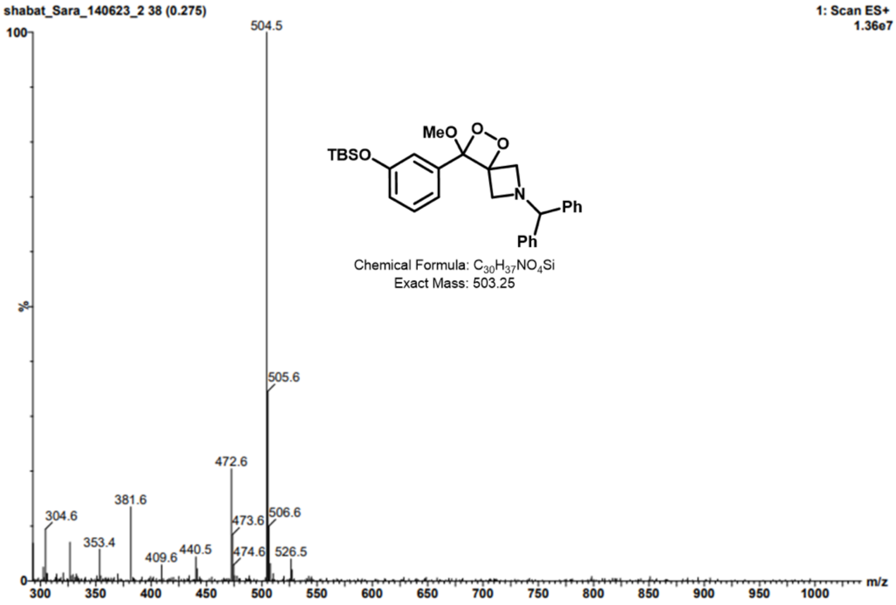

Compound 15a

^{1}H-NMR

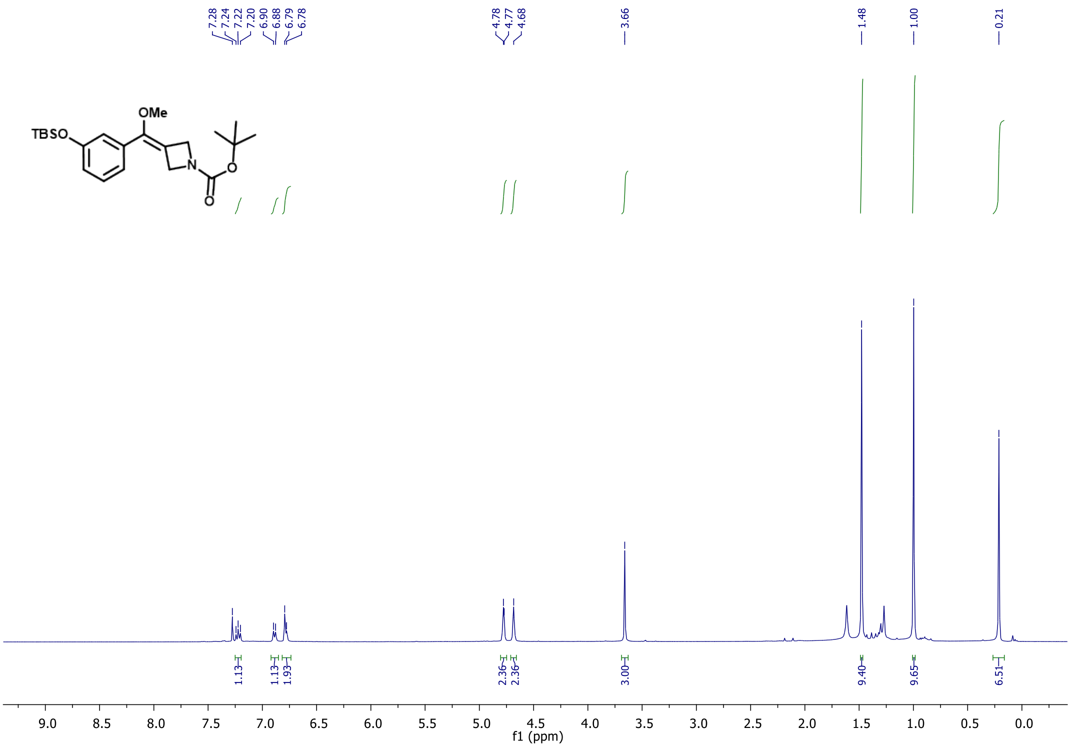

^{13}C-NMR

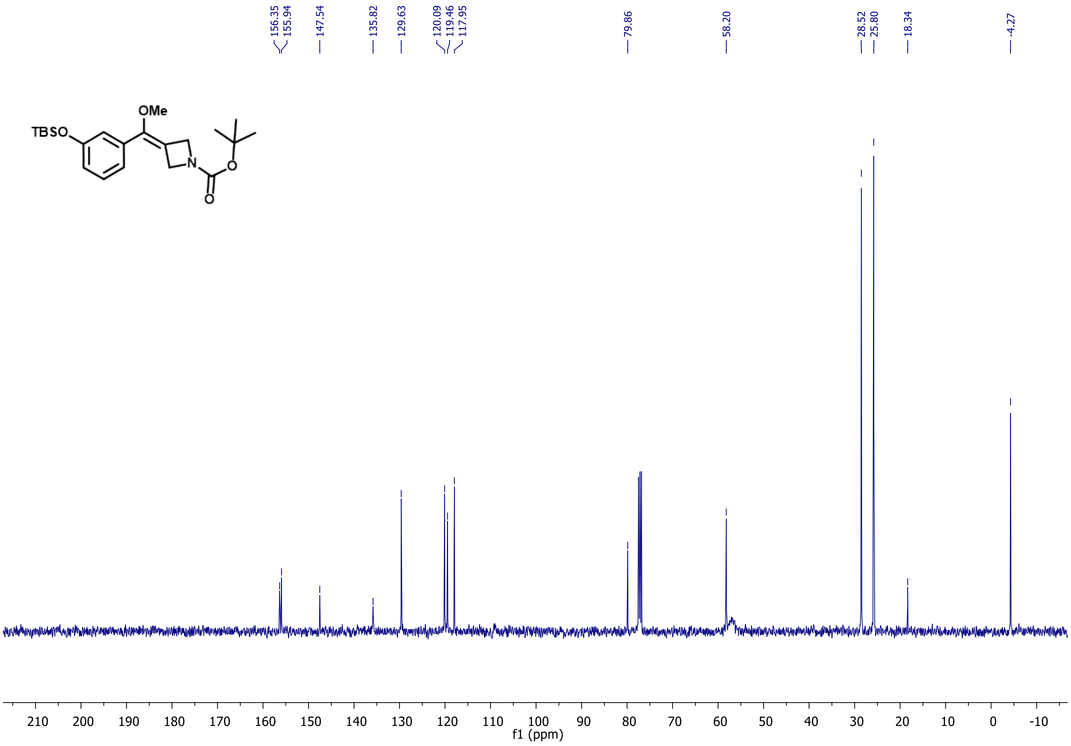

3D HPLC spectra (90-100% ACN in water, 0.1%TFA)

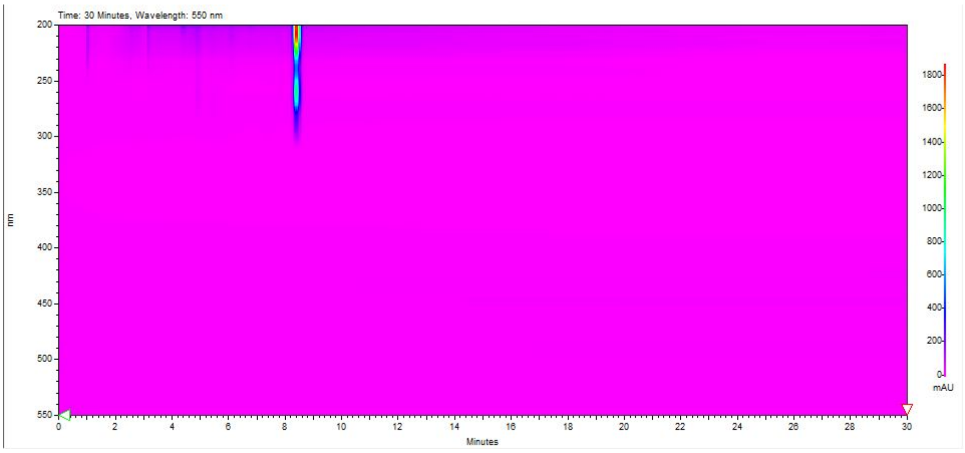

2D HPLC spectra (Absorbance measured at 275nm)

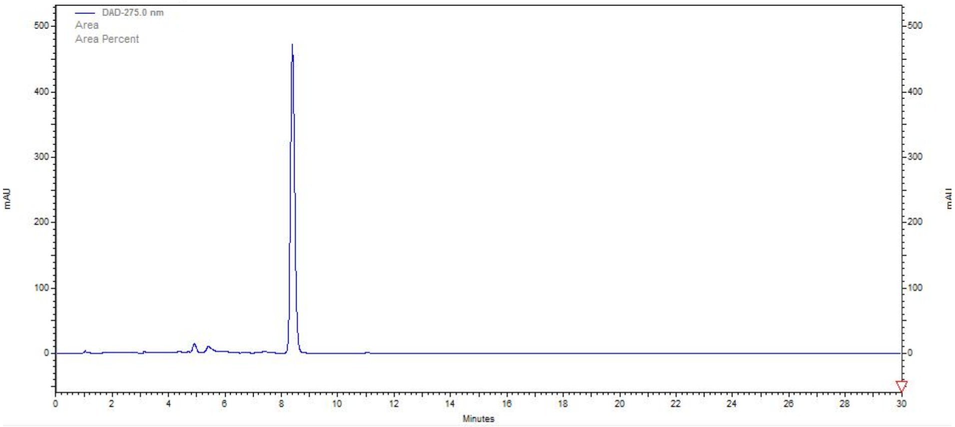

Mass spectra

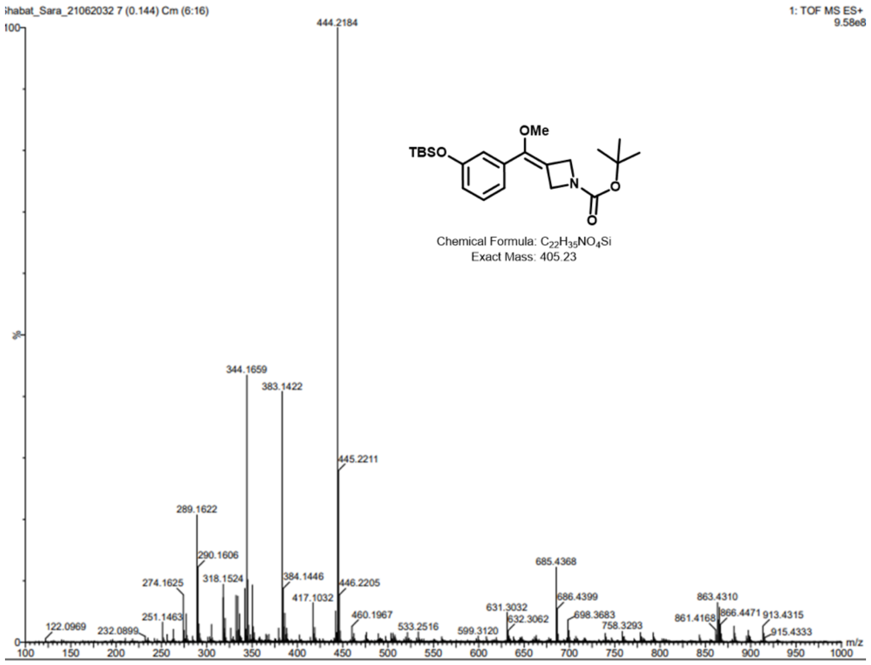

Diox 13

^{1}H-NMR

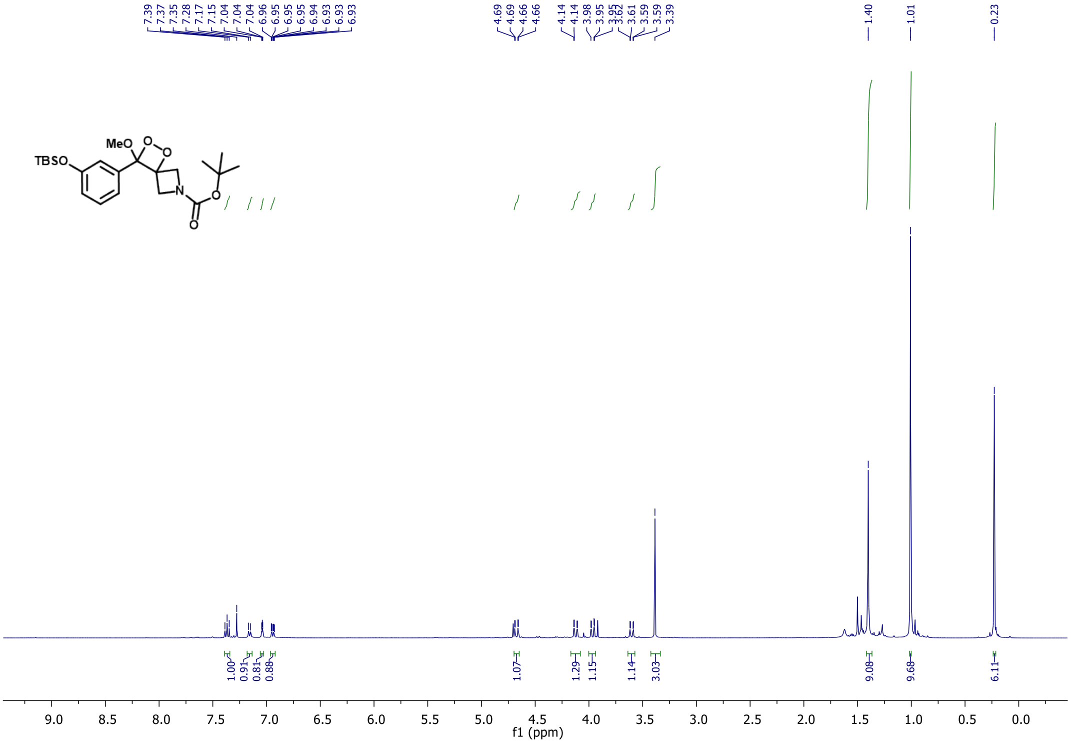

^{13}C-NMR

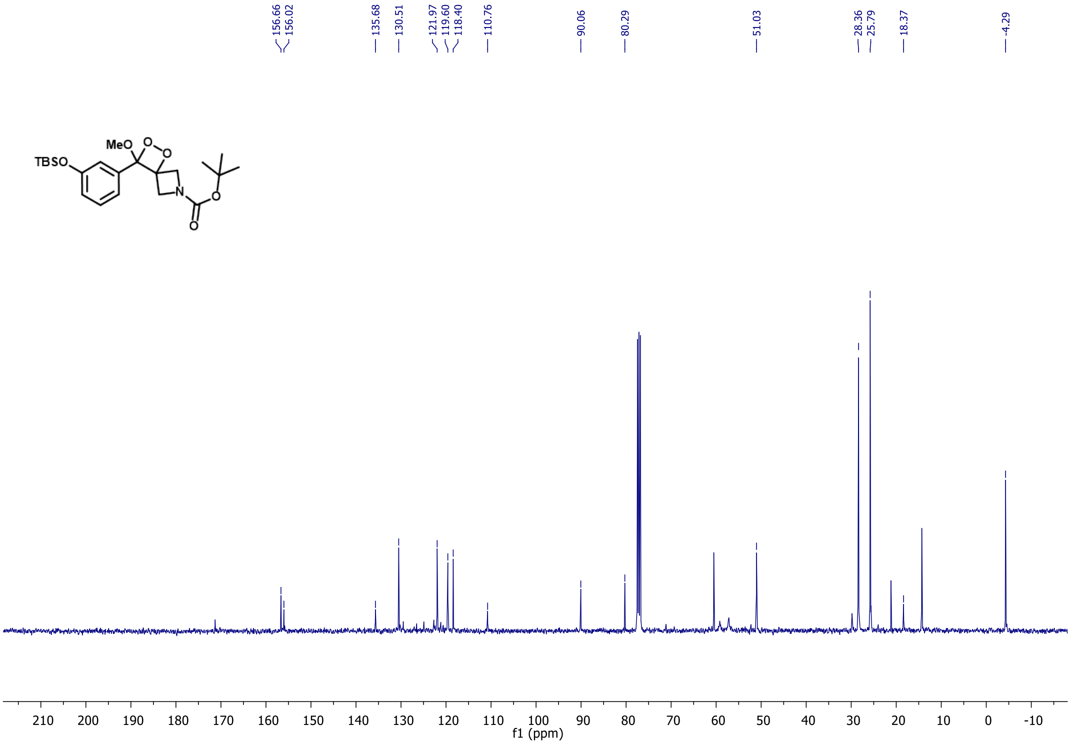

3D HPLC spectra (90-100% ACN in water, 0.1%TFA)

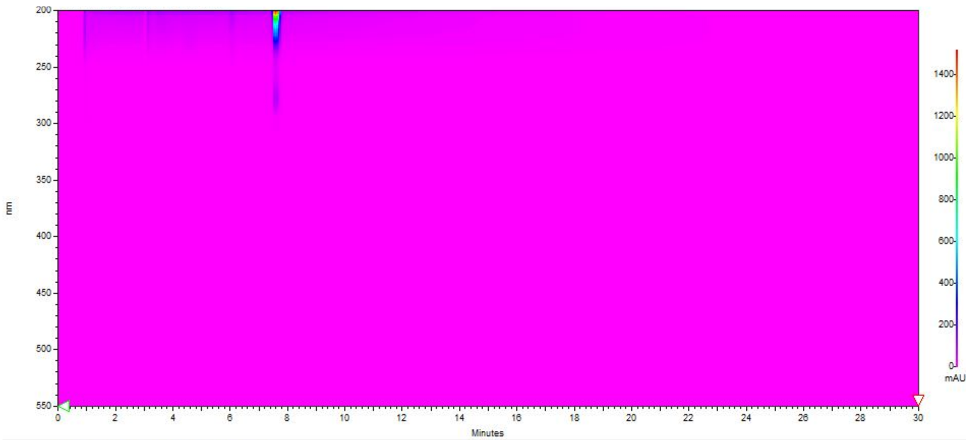

2D HPLC spectra (Absorbance measured at 277nm)

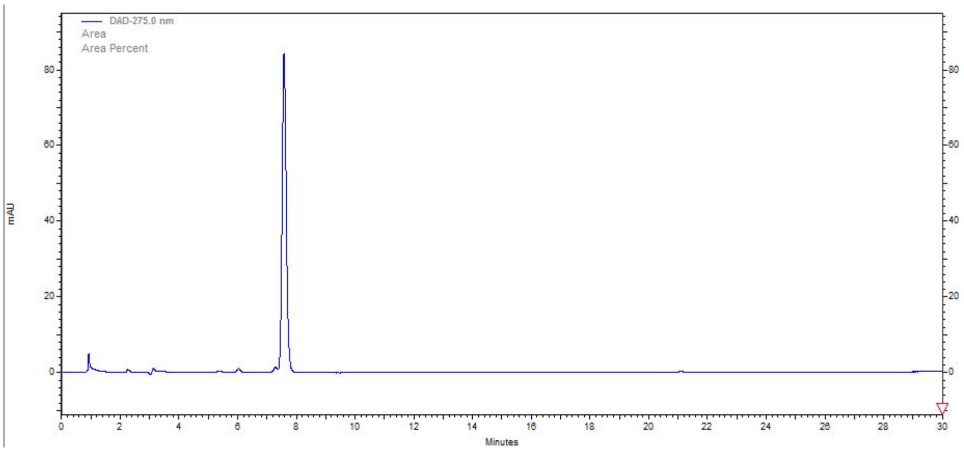

Mass spectra

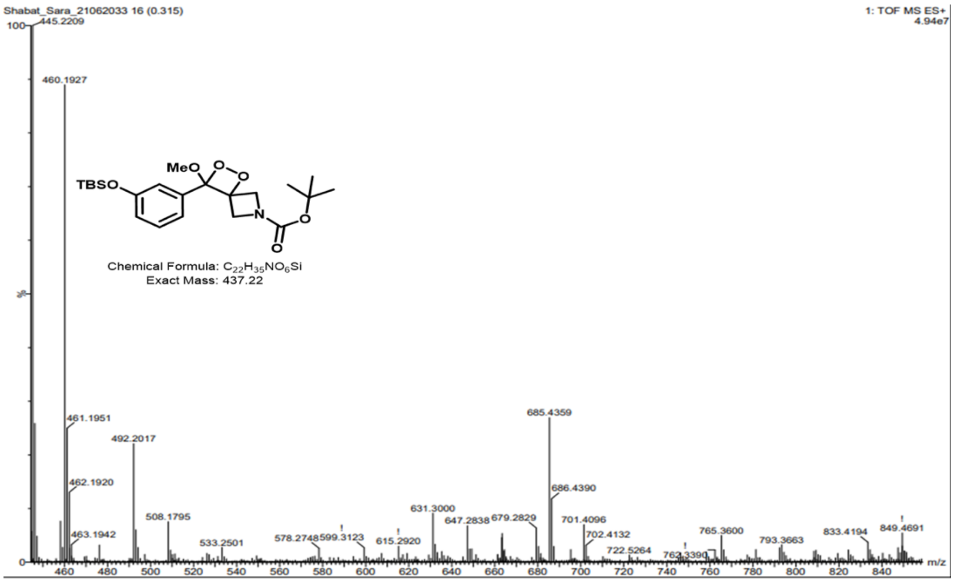

Compound 16a

^{1}H-NMR

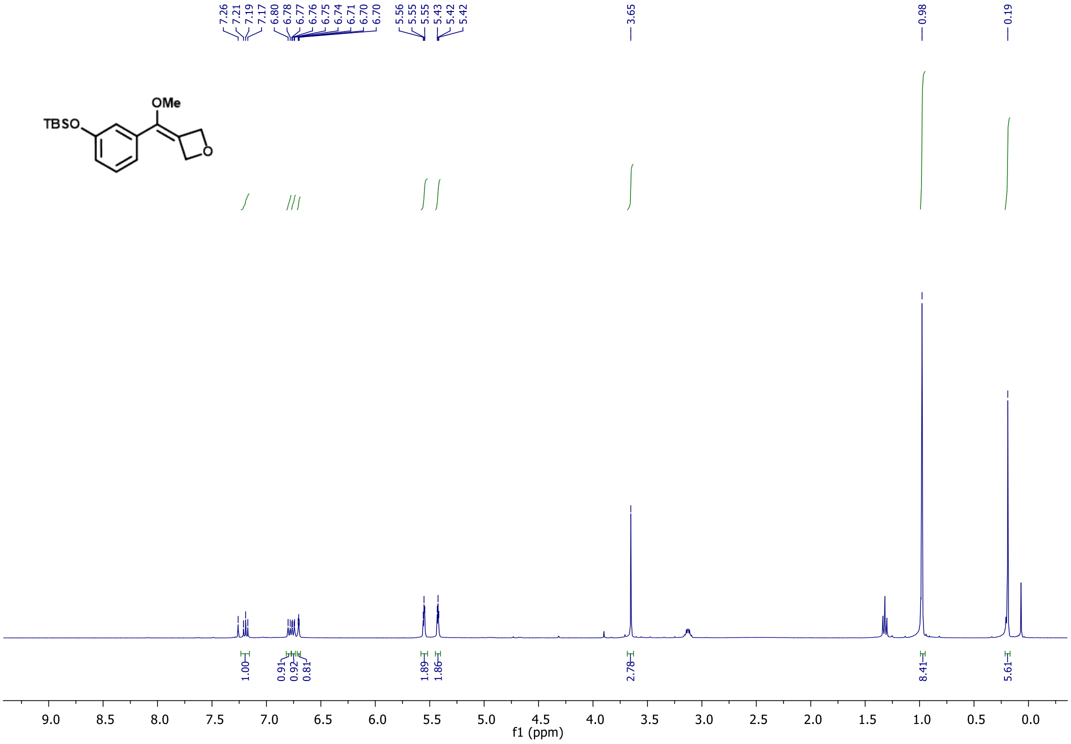

^{13}C-NMR

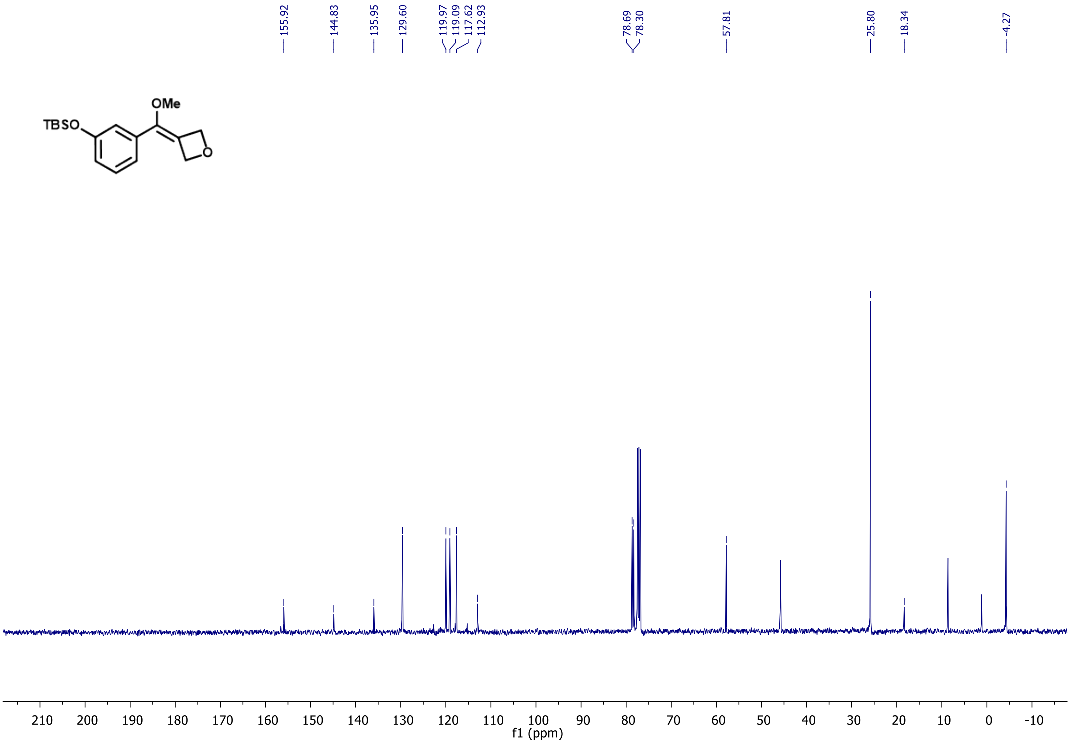

3D HPLC spectra (90-100% ACN in water, 0.1%TFA)

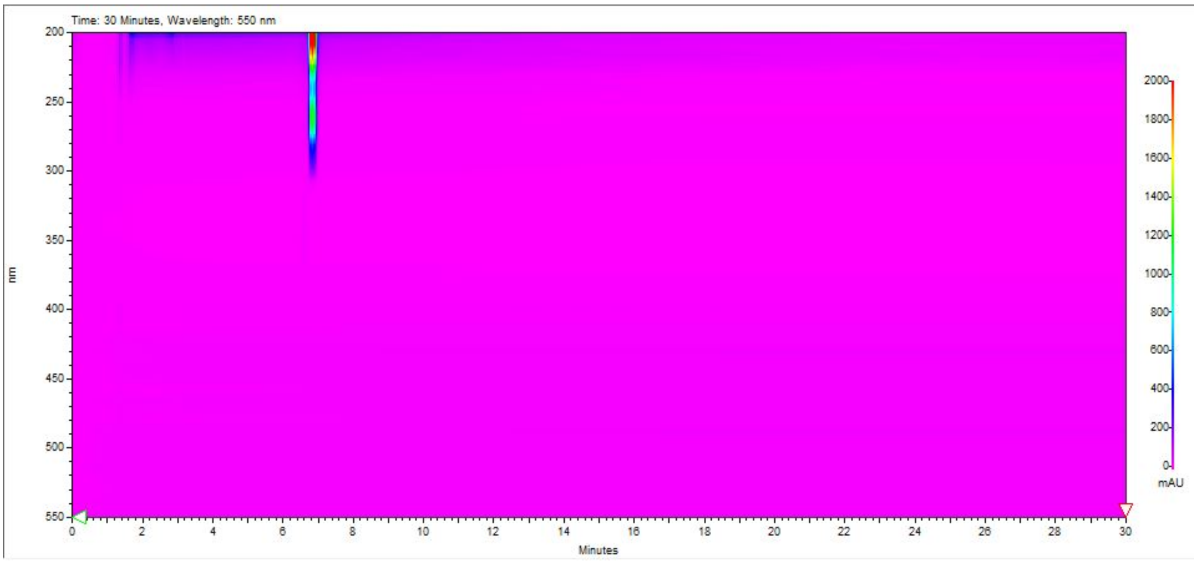

2D HPLC spectra (Absorbance measured at 275nm)

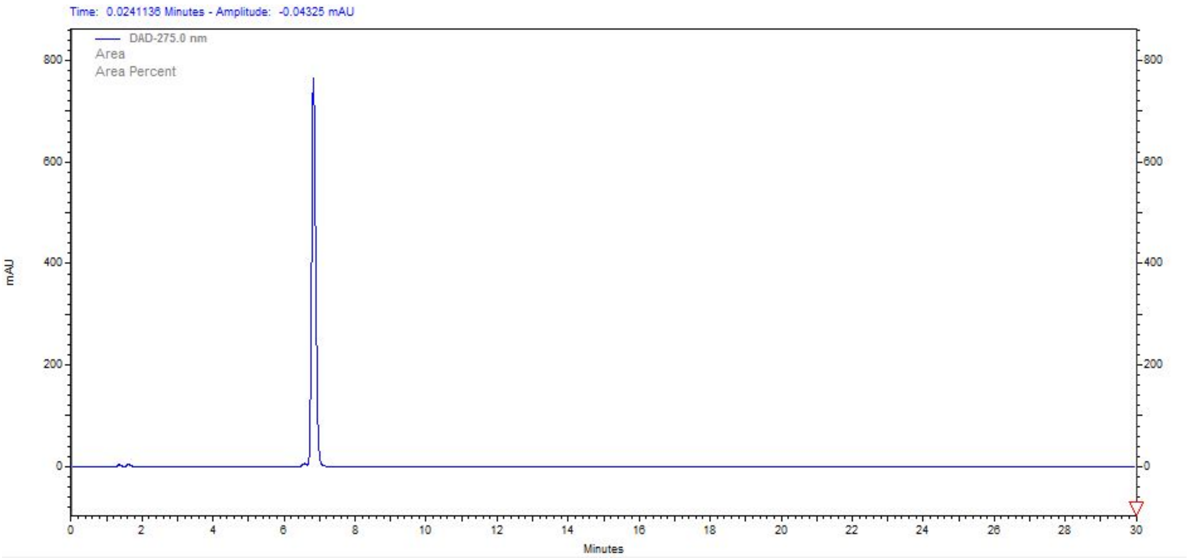

Diox 14

^{1}H-NMR

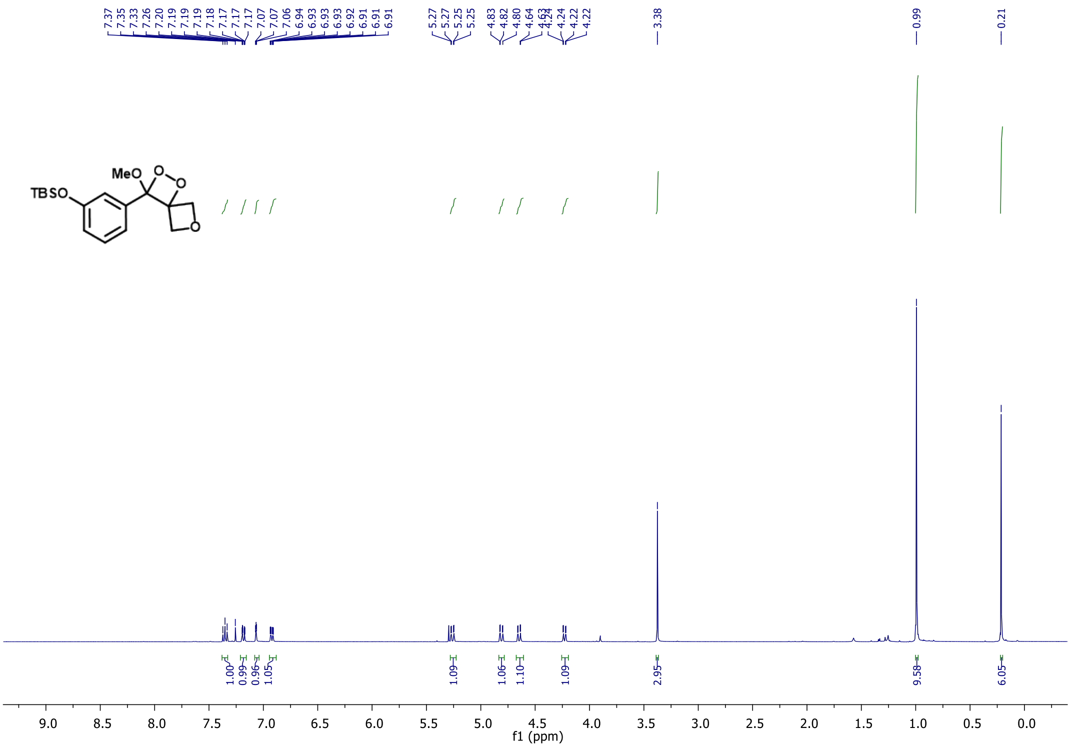

^{13}C-NMR

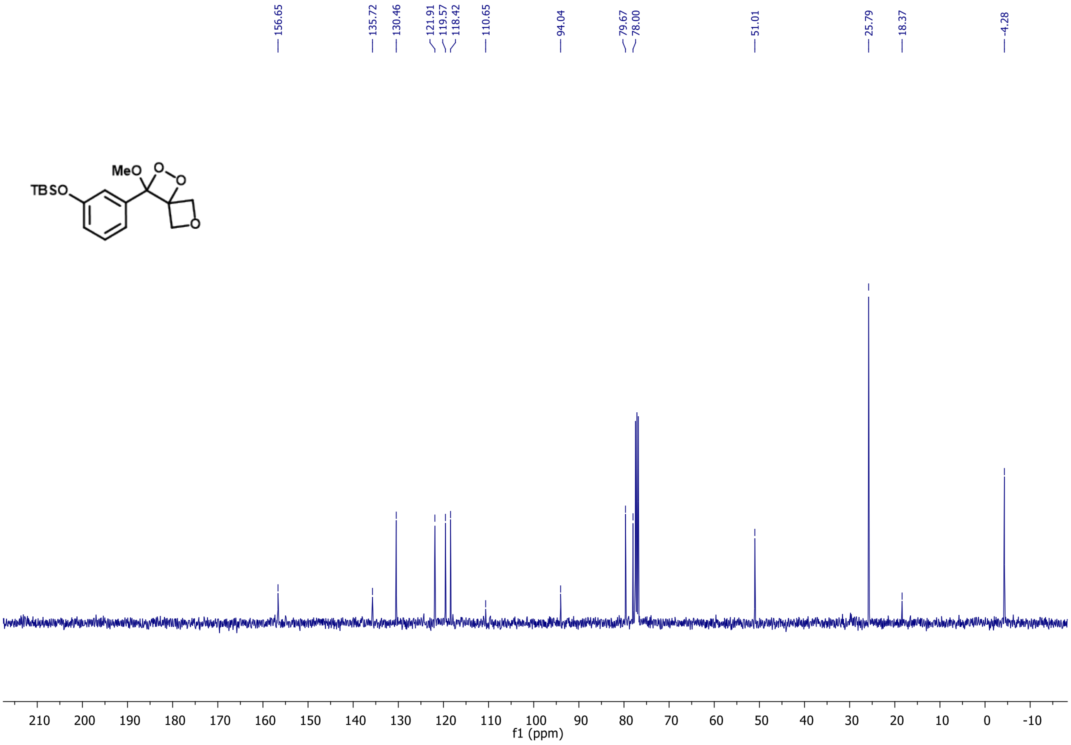

3D HPLC spectra (90-100% ACN in water, 0.1%TFA)

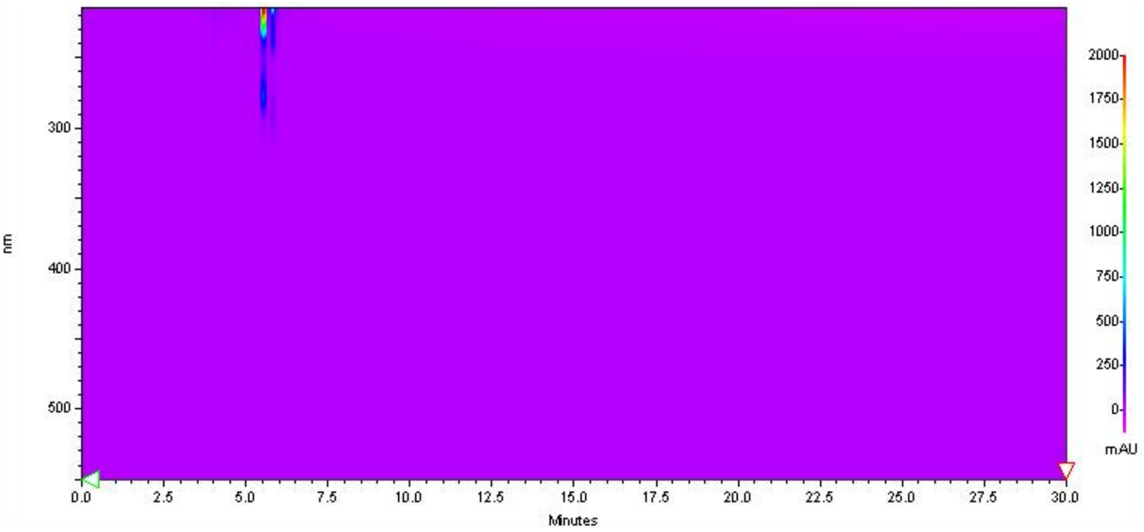

2D HPLC spectra (Absorbance measured at 277nm)

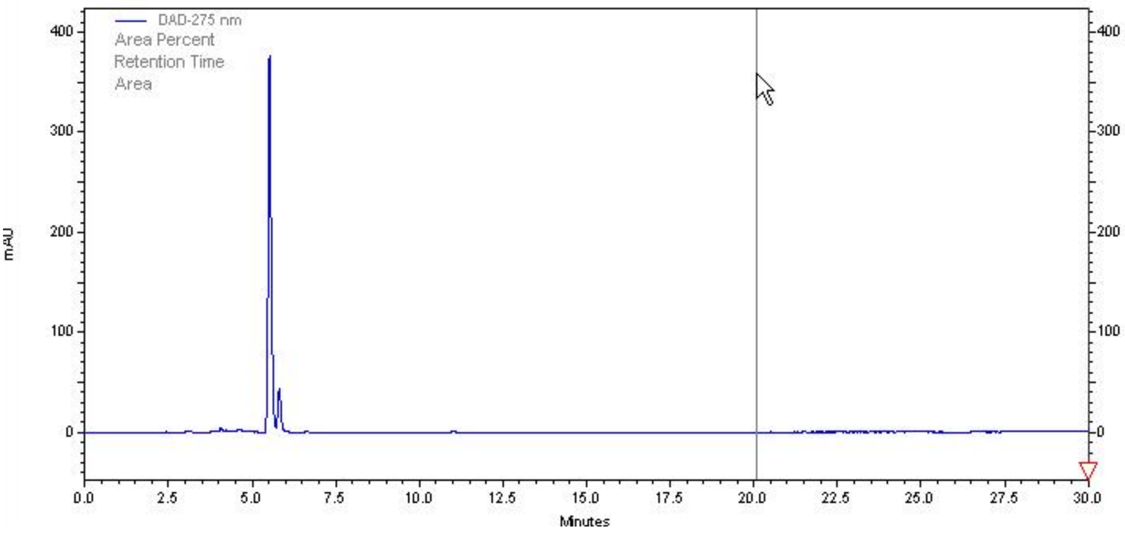

Compound 17a

^{1}H-NMR

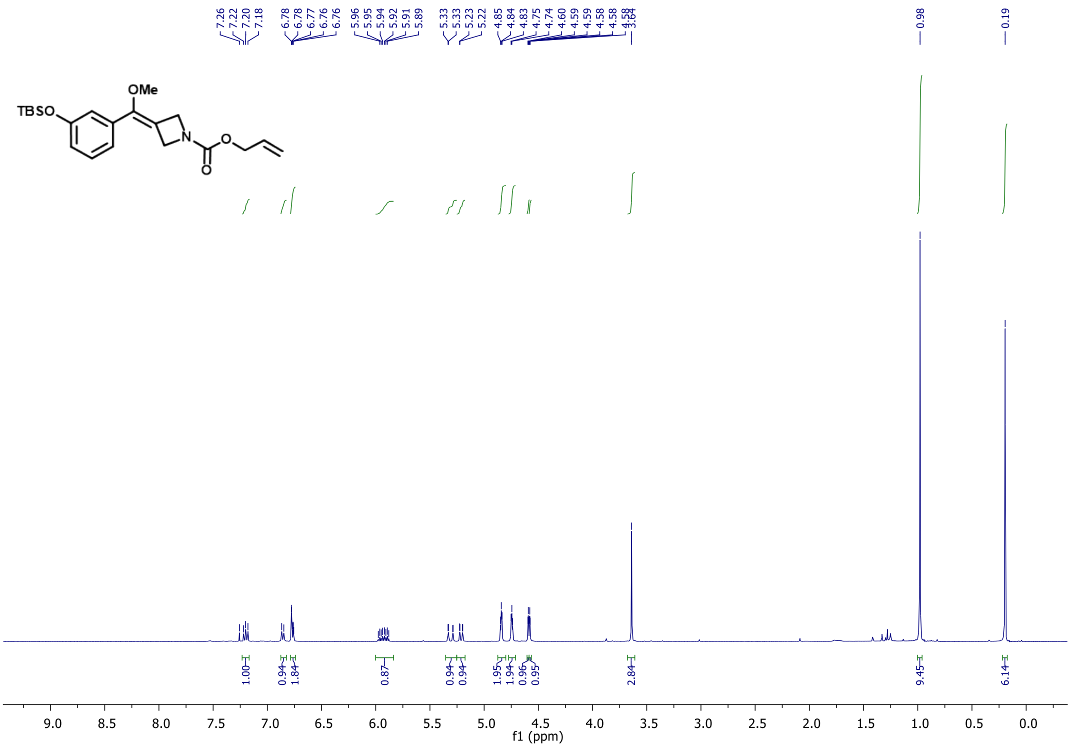

^{13}C-NMR

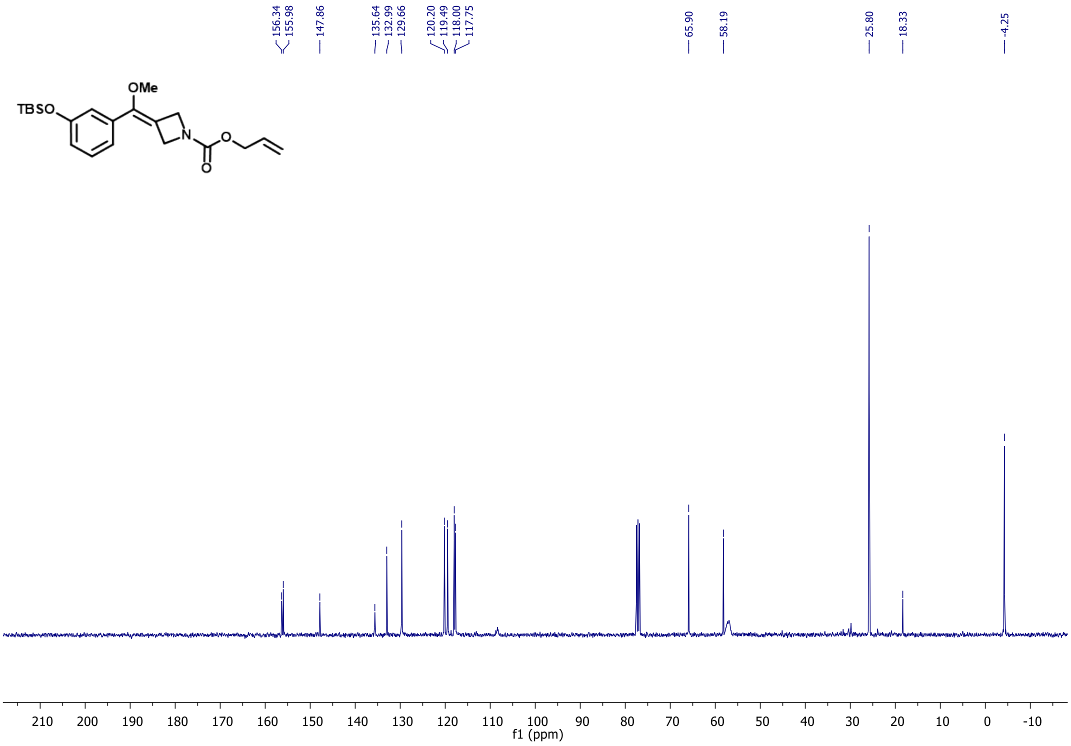

Mass spectra

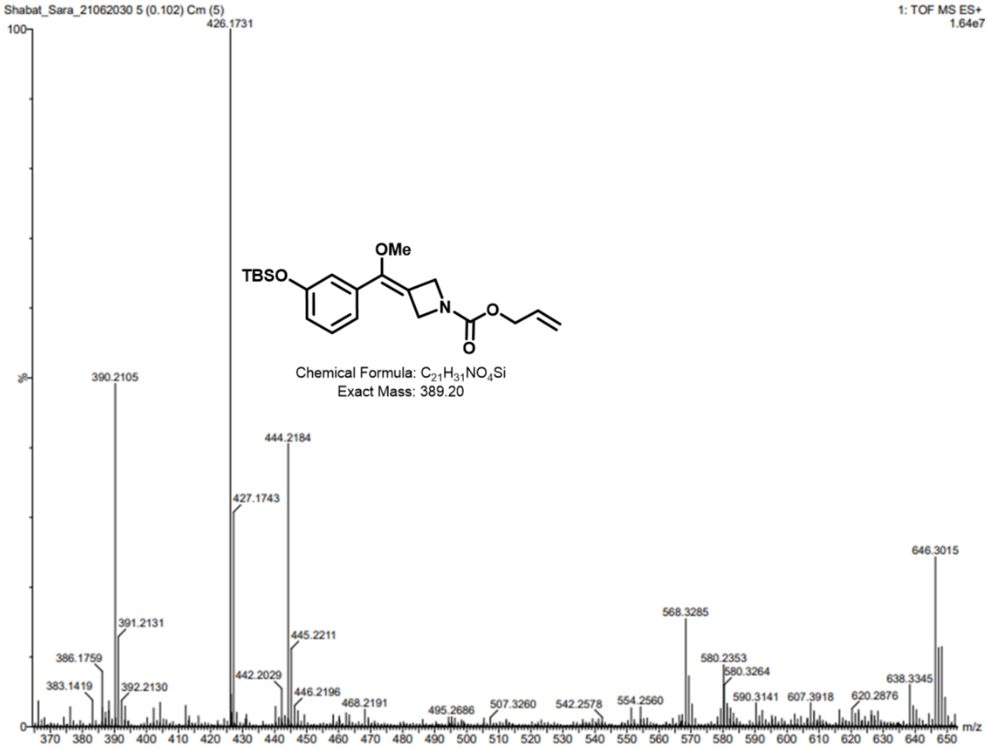

Compound 18a

^{1}H-NMR

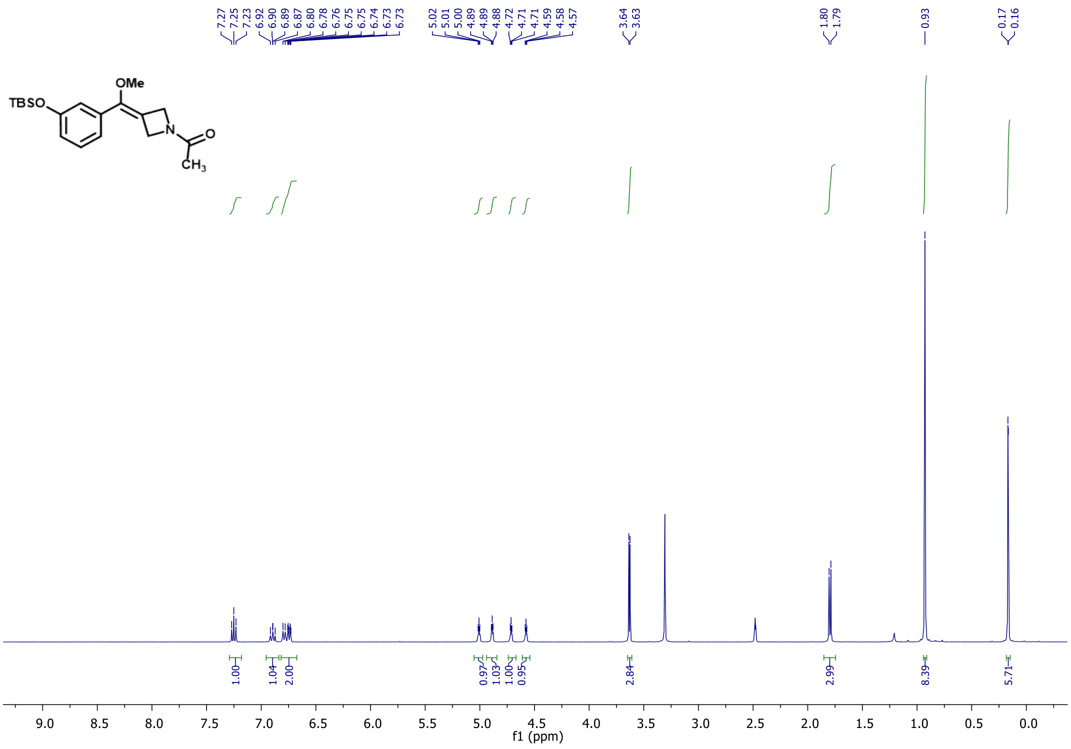

^{13}C-NMR

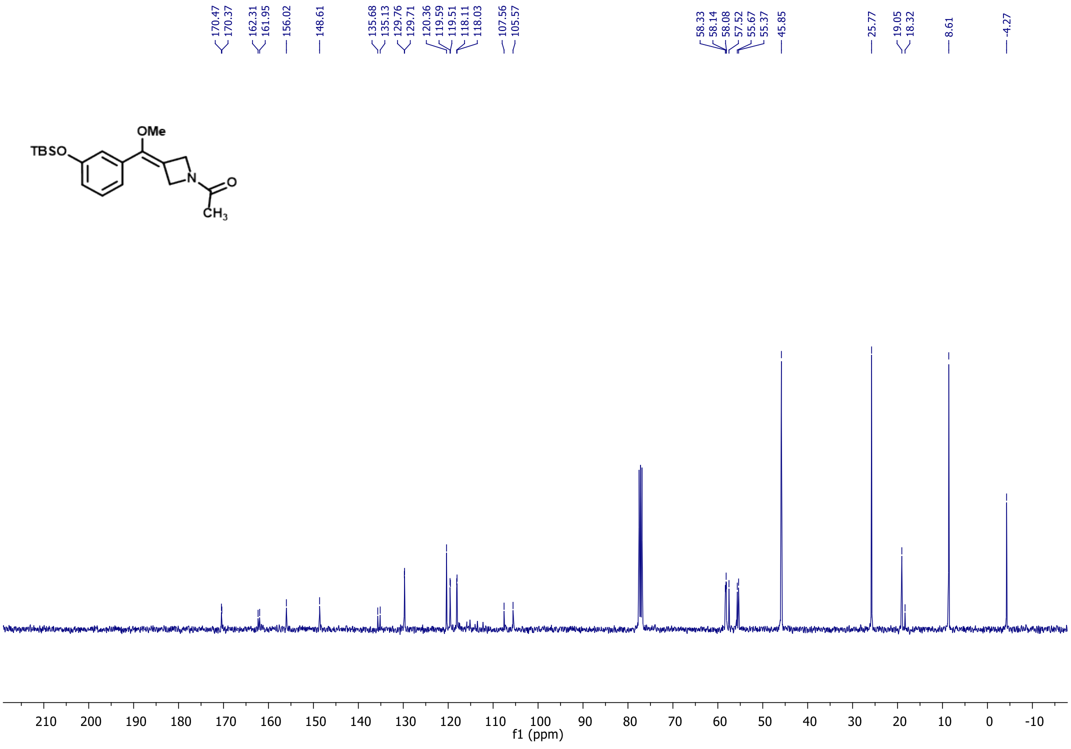

Mass spectra

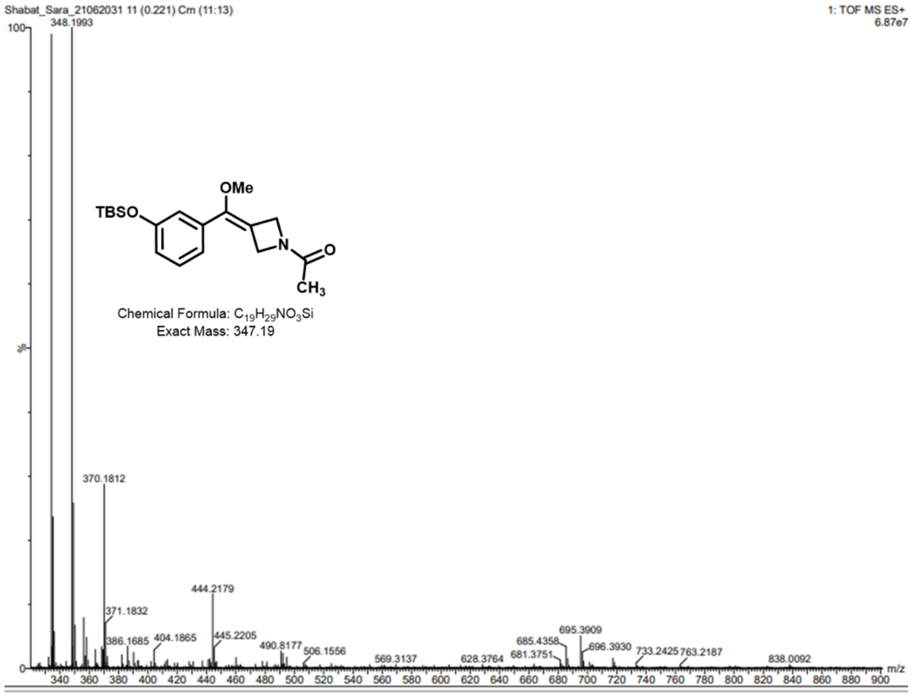

Diox 15

^{1}H-NMR

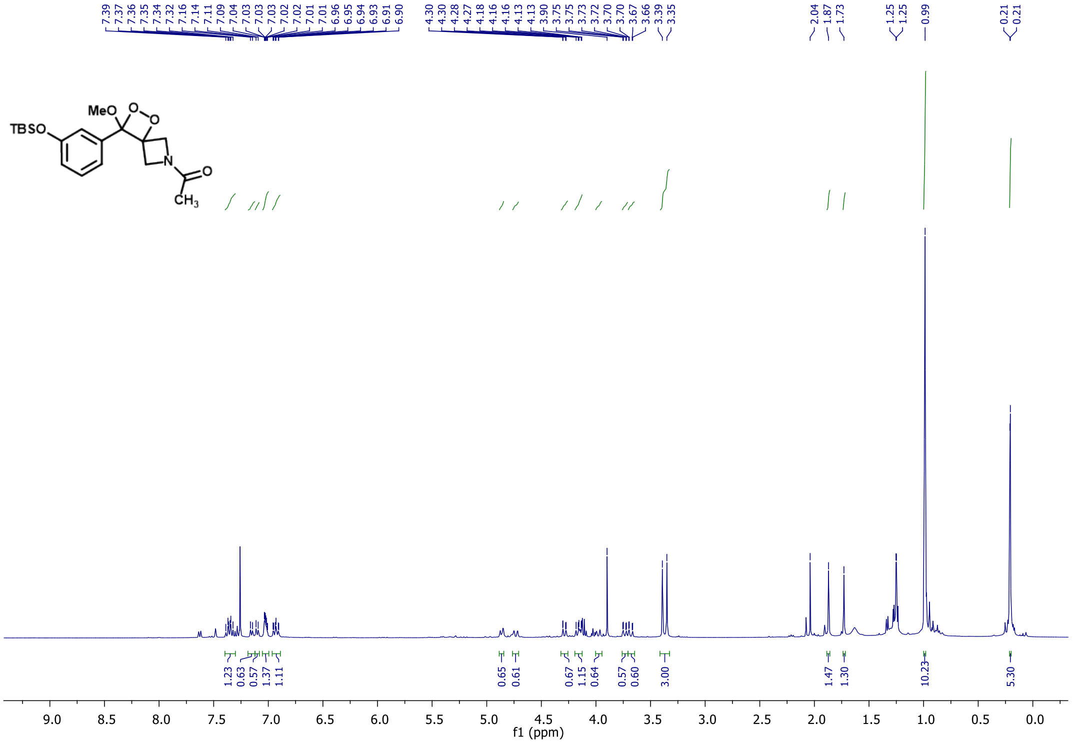

^{13}C-NMR

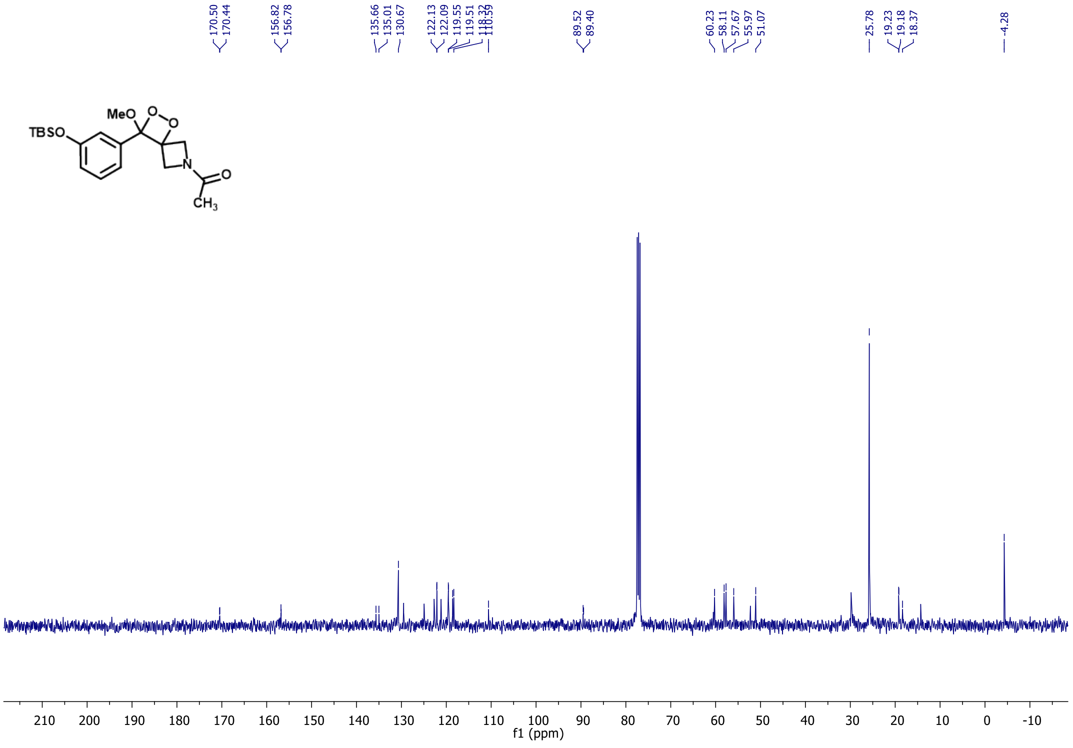

Mass spectra

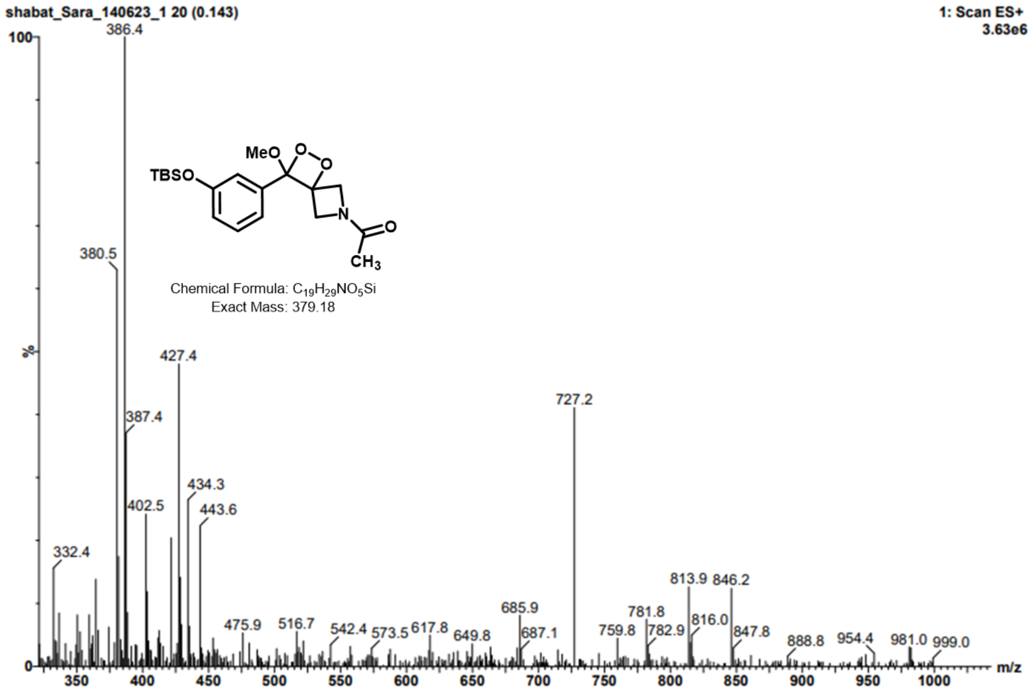

Coumarin compounds

Compound IV

^{1}H-NMR

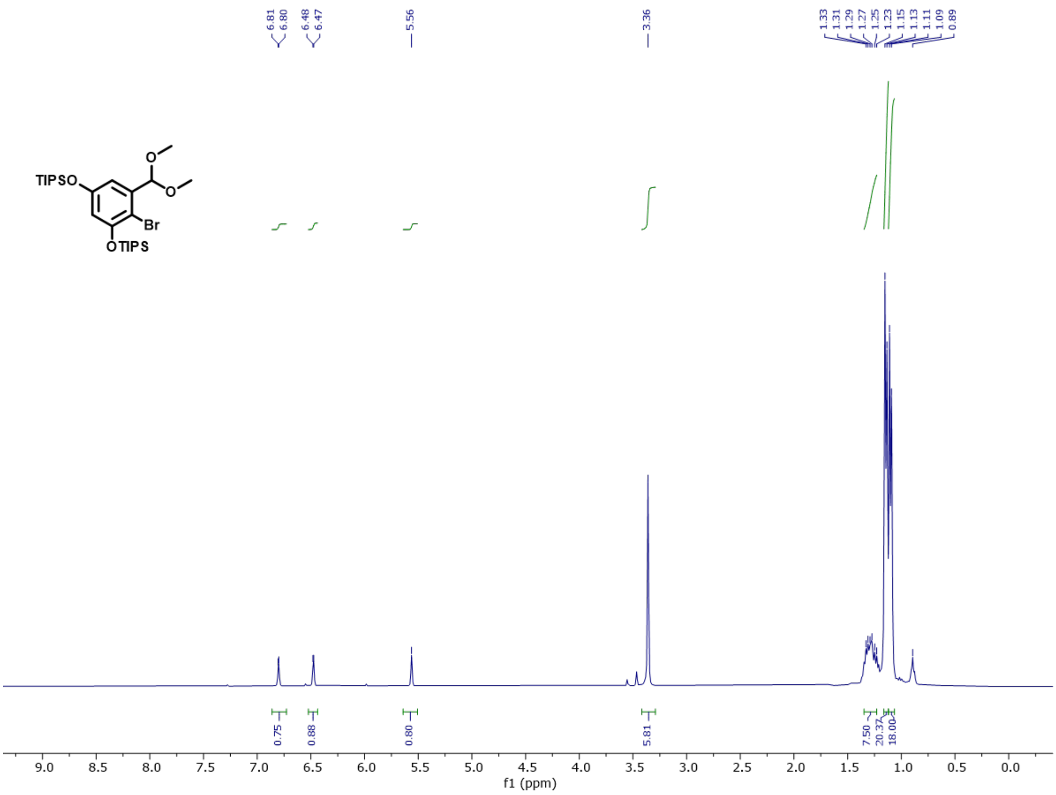

^{13}C-NMR

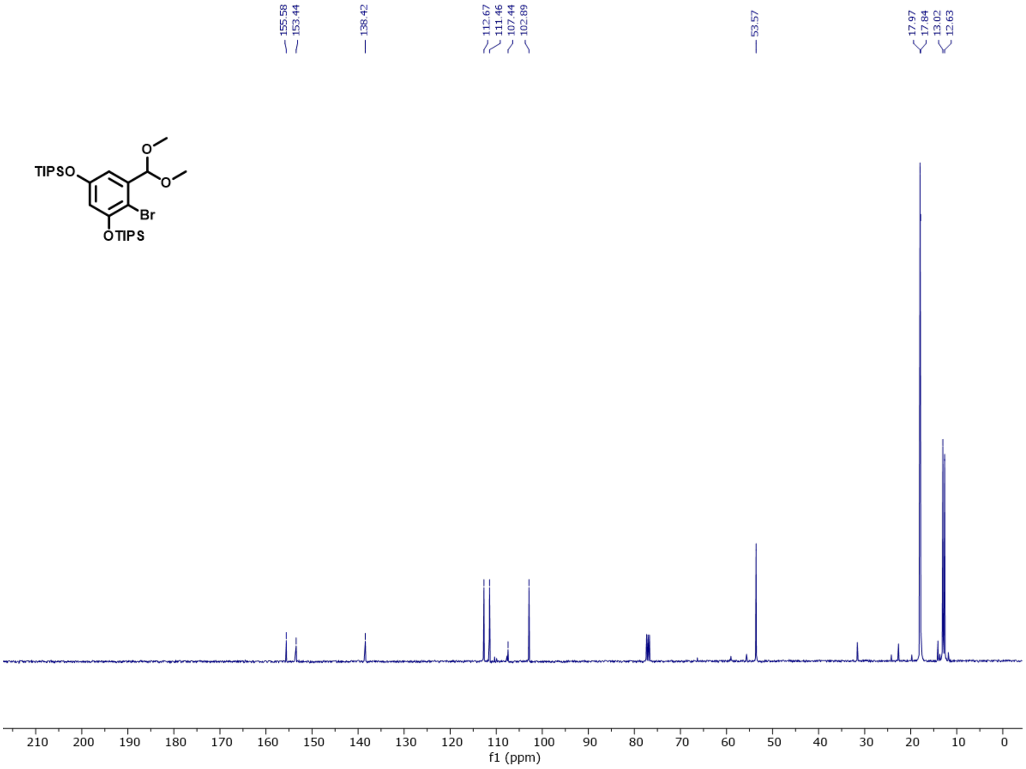

Compound V

^{1}H-NMR

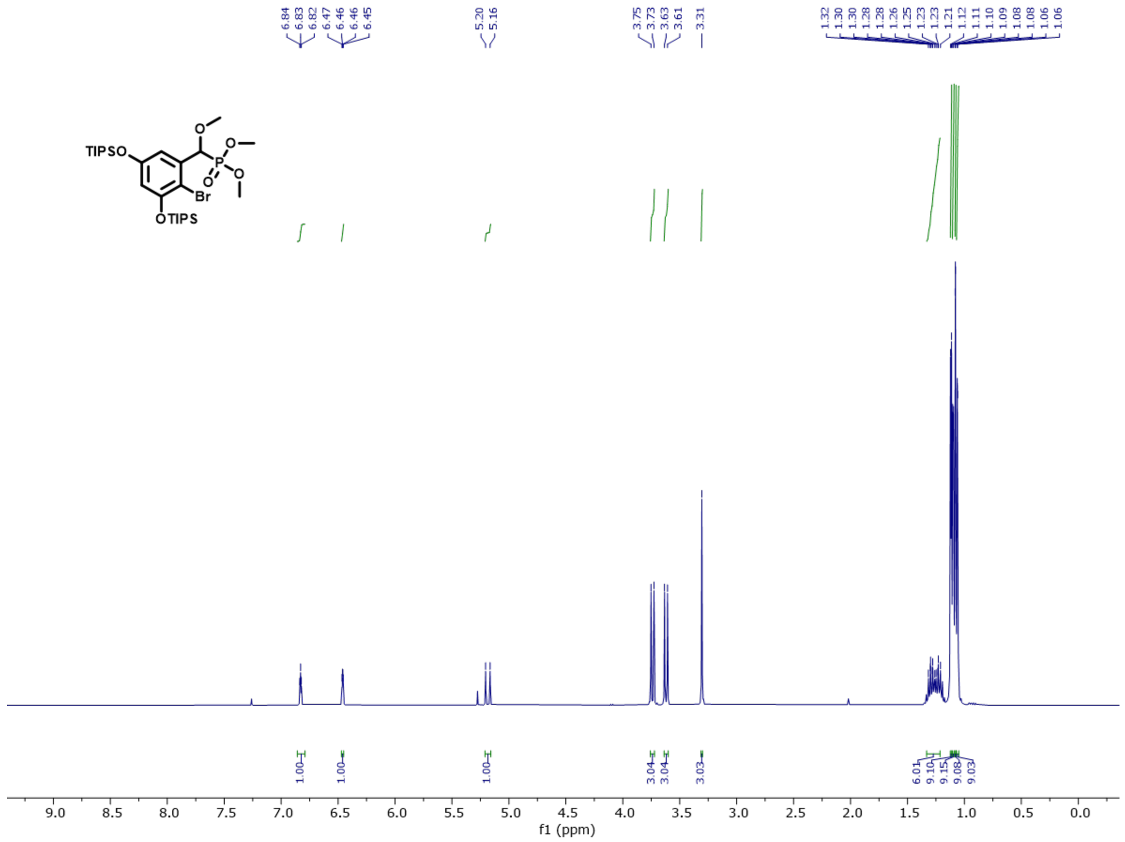

^{13}C-NMR

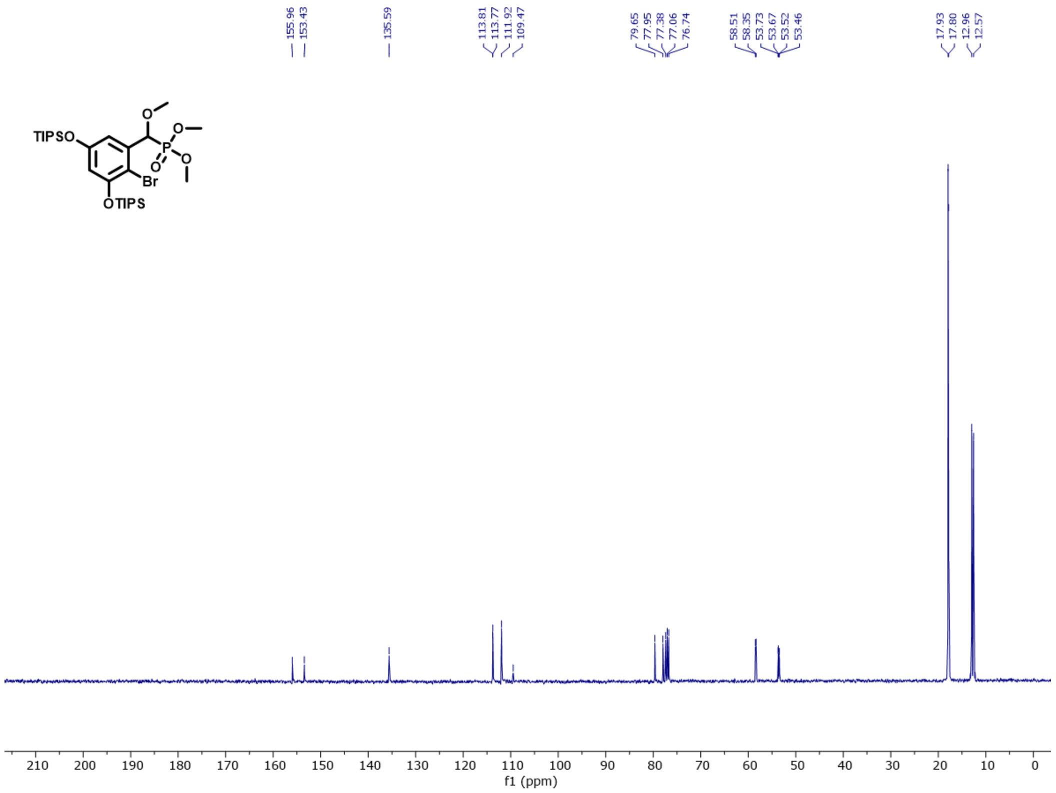

Compound 1b

^{1}H-NMR

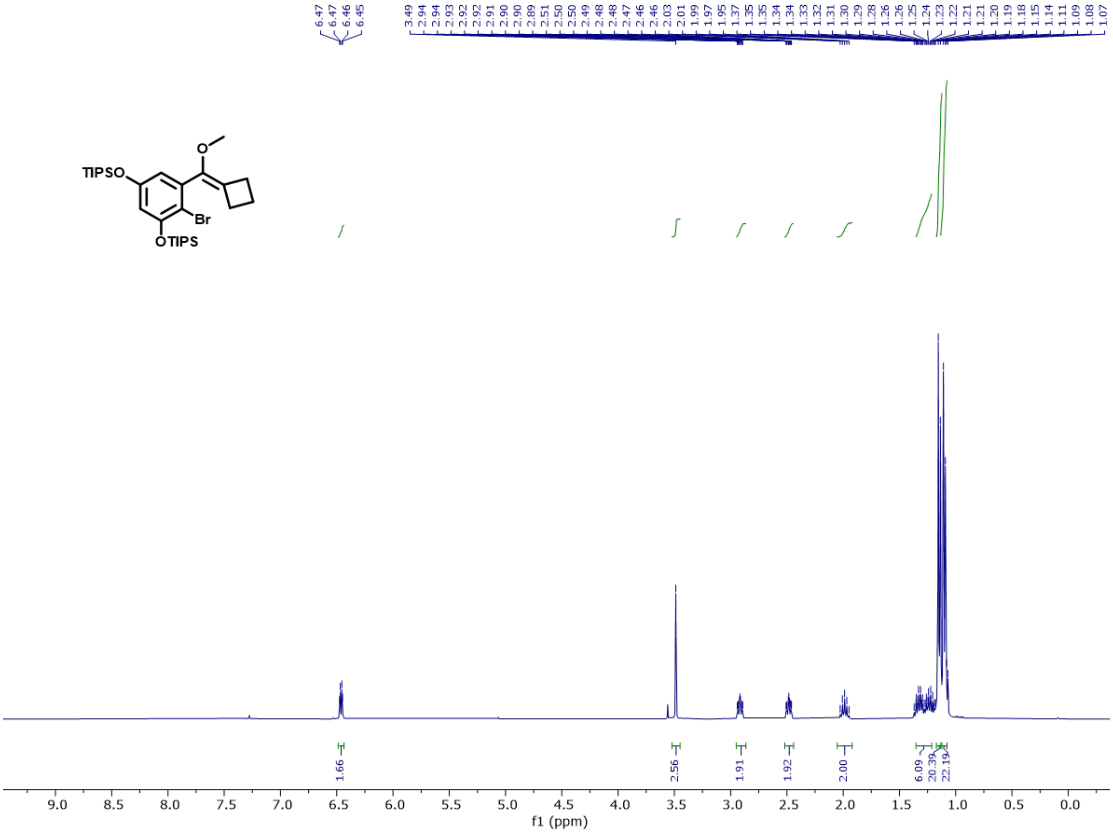

^{13}C-NMR

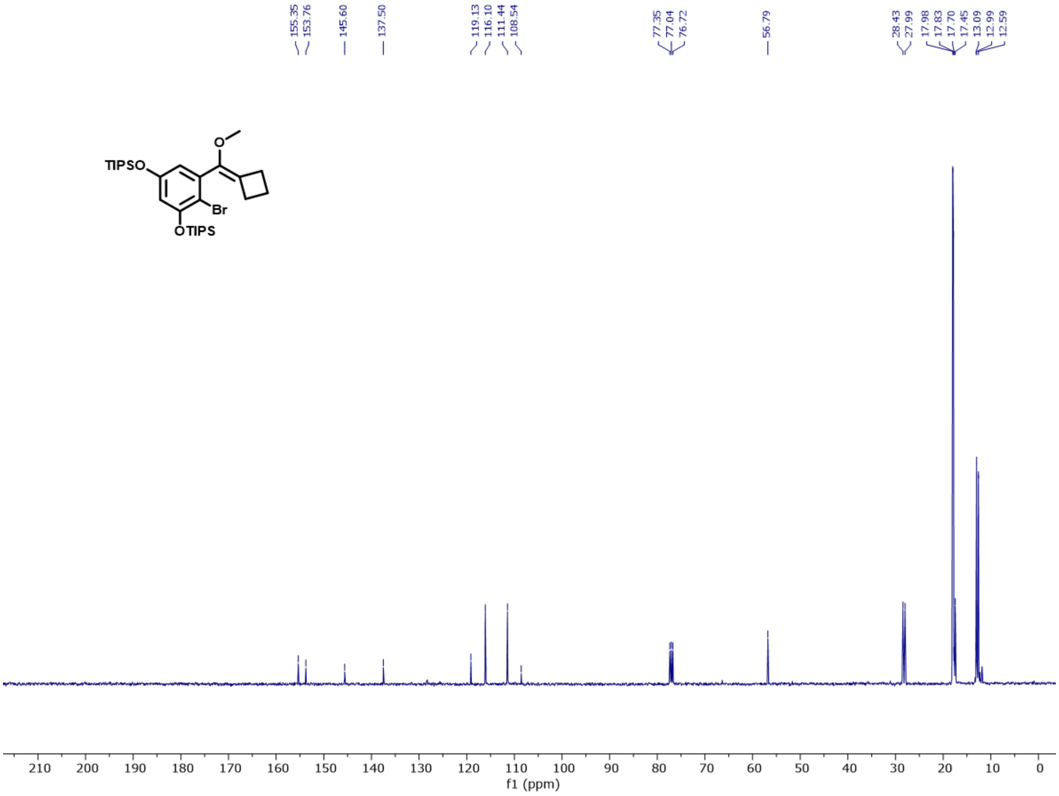

Compound 1c

^{1}H-NMR

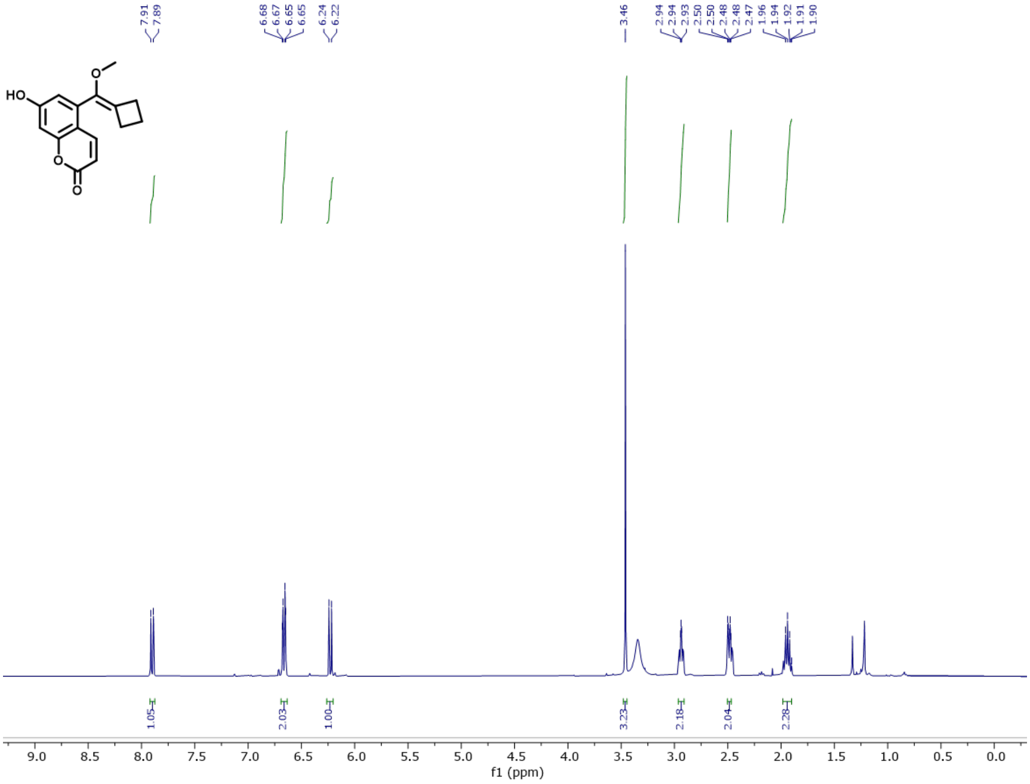

^{13}C-NMR

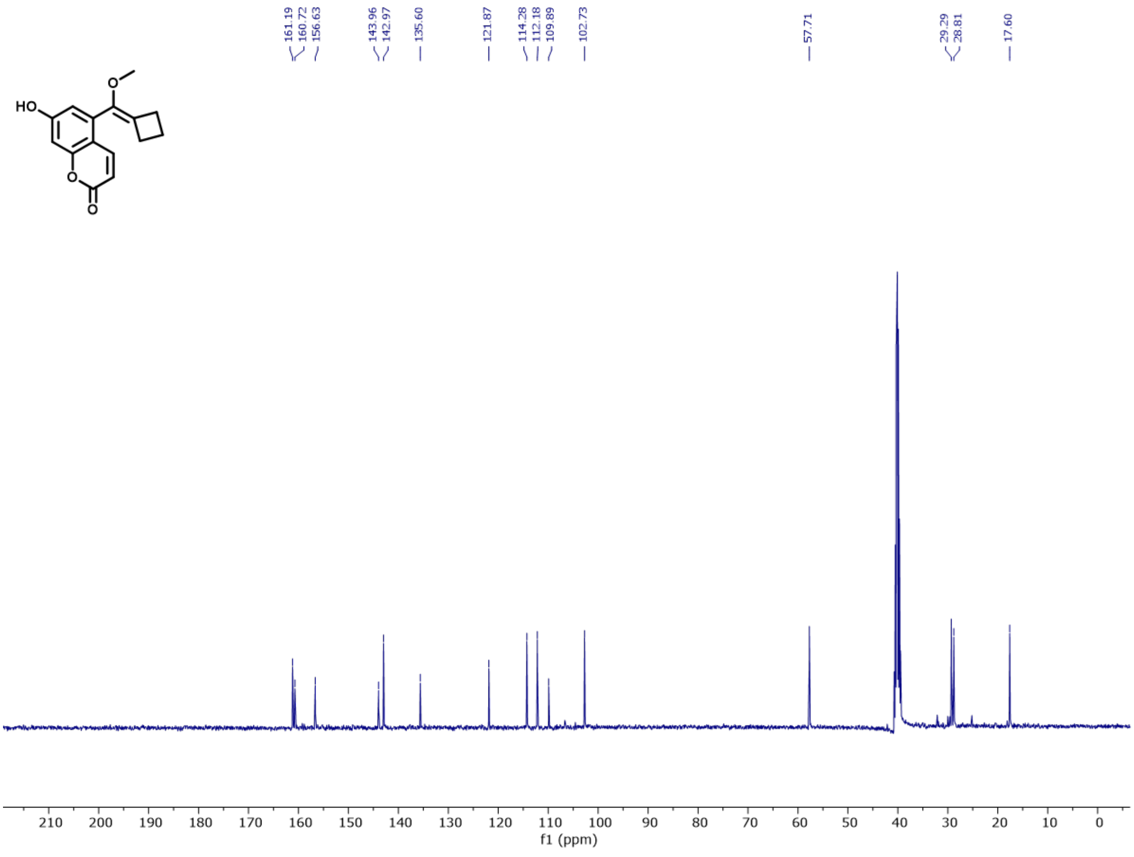

Probe Coumarin CB

Mass spectra

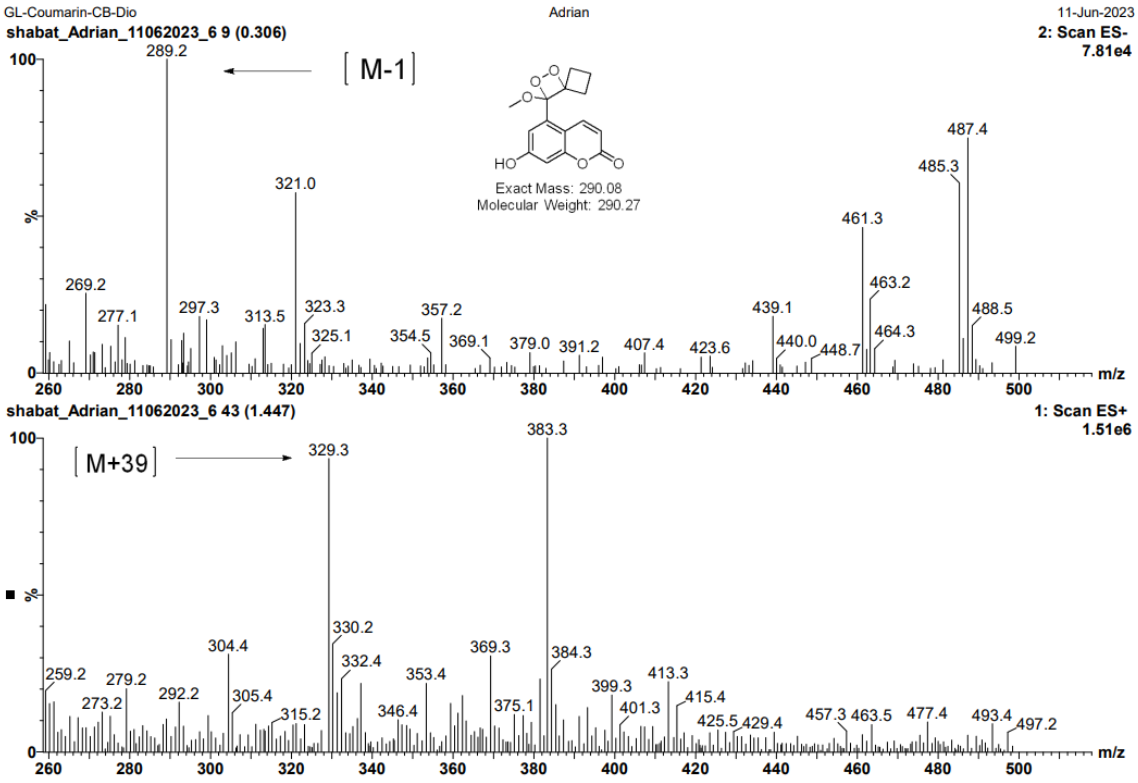

Compound 2b

^{1}H-NMR

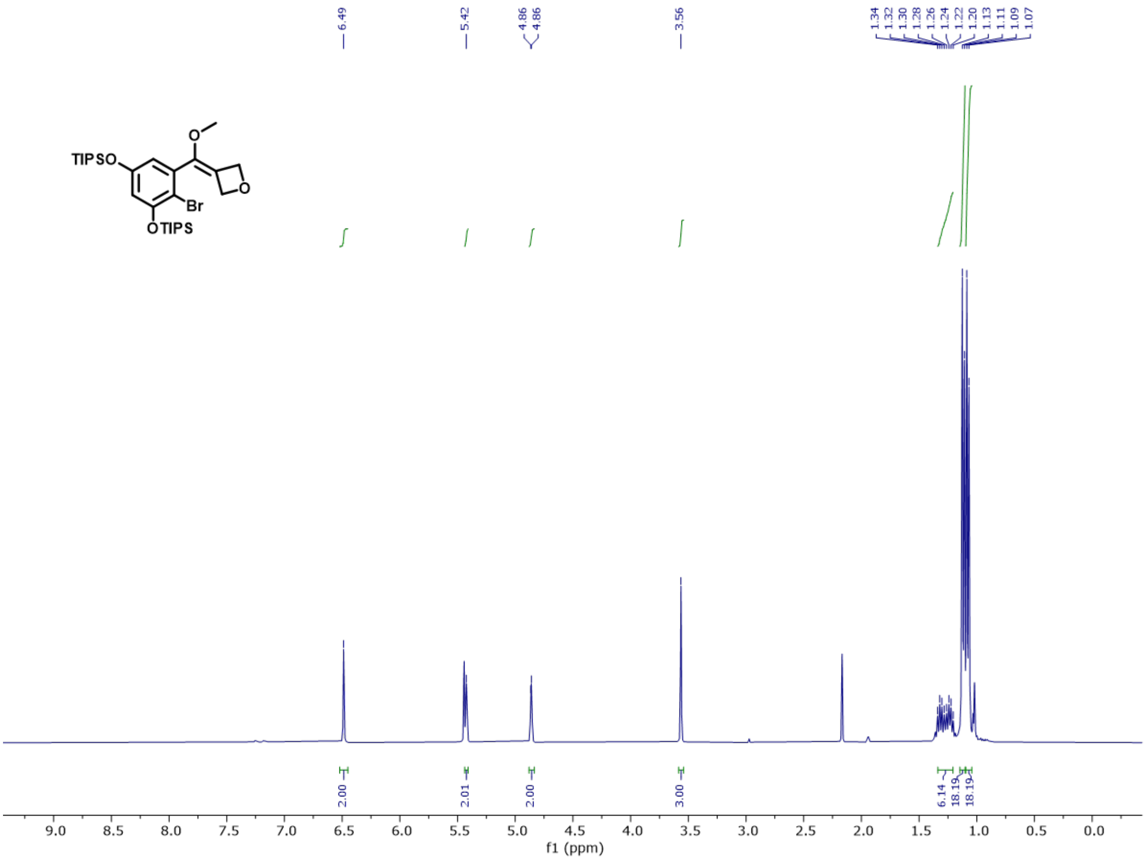

^{13}C-NMR

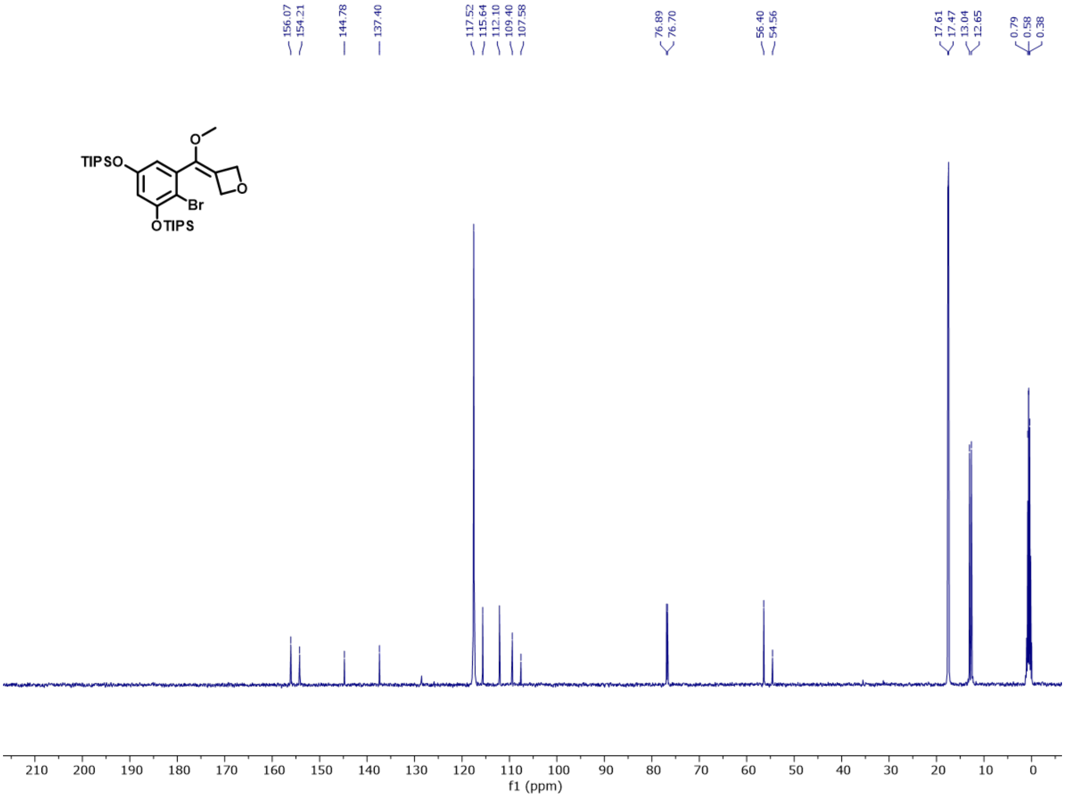

Compound 2c

^{1}H-NMR

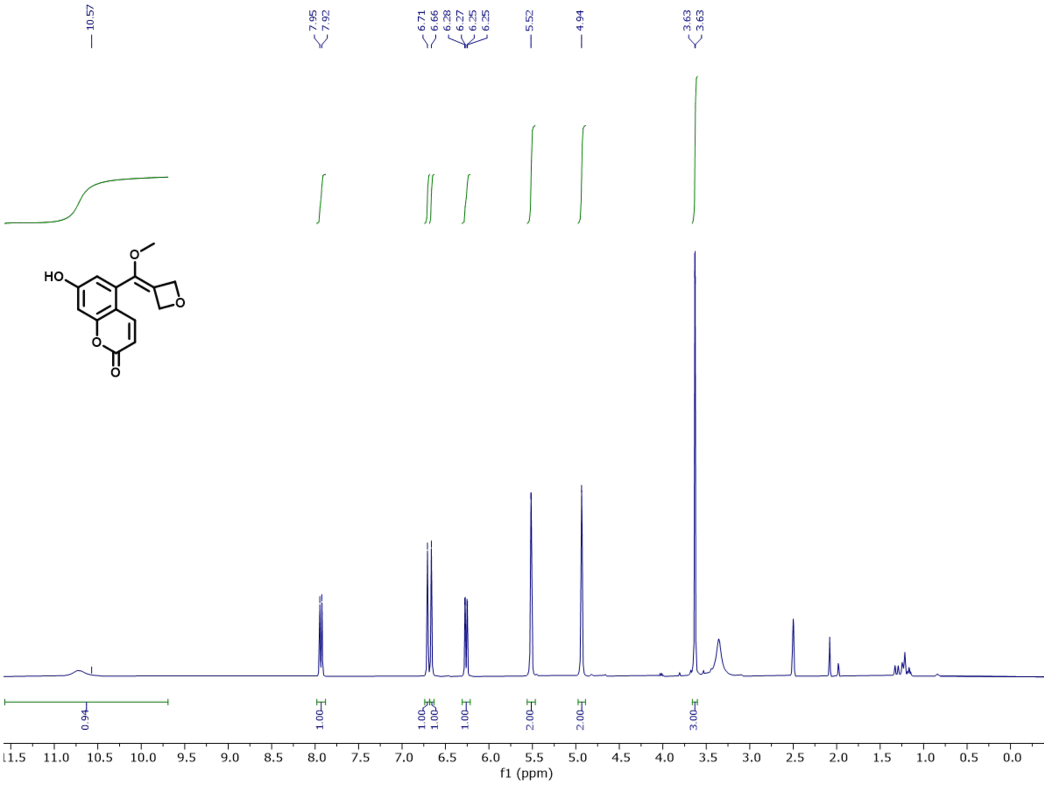

^{13}C-NMR

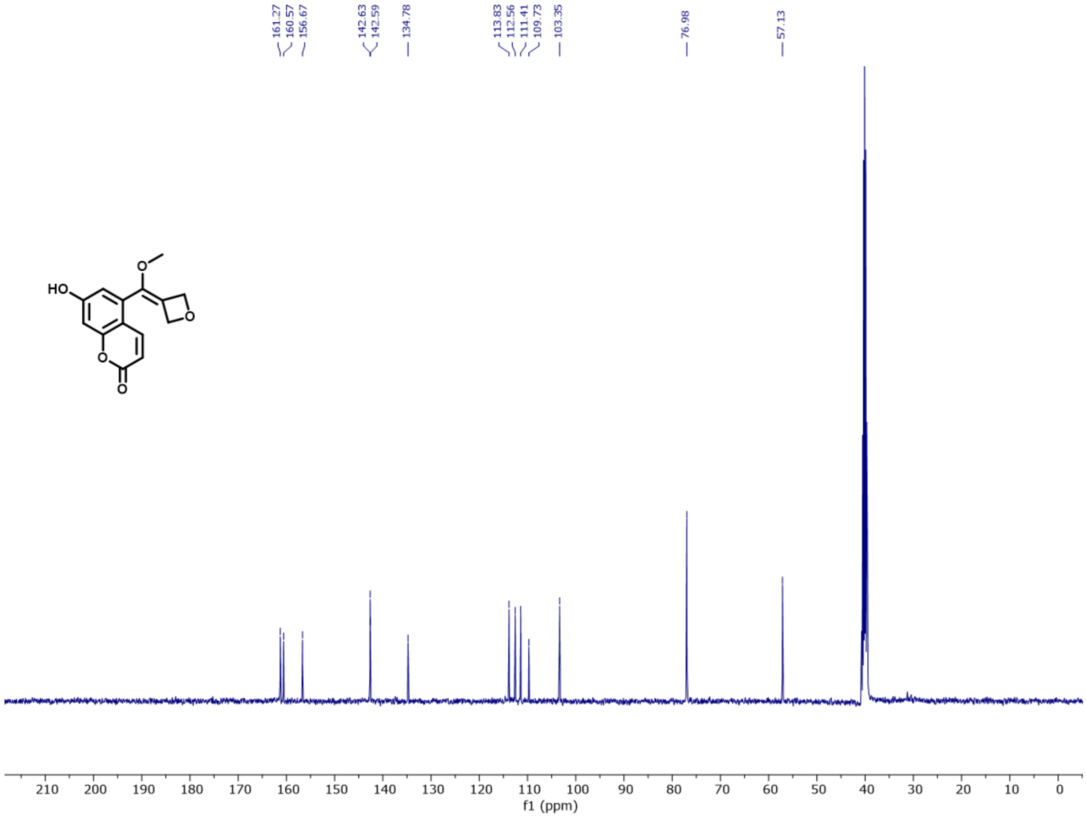

Probe Coumarin OX

Mass spectra

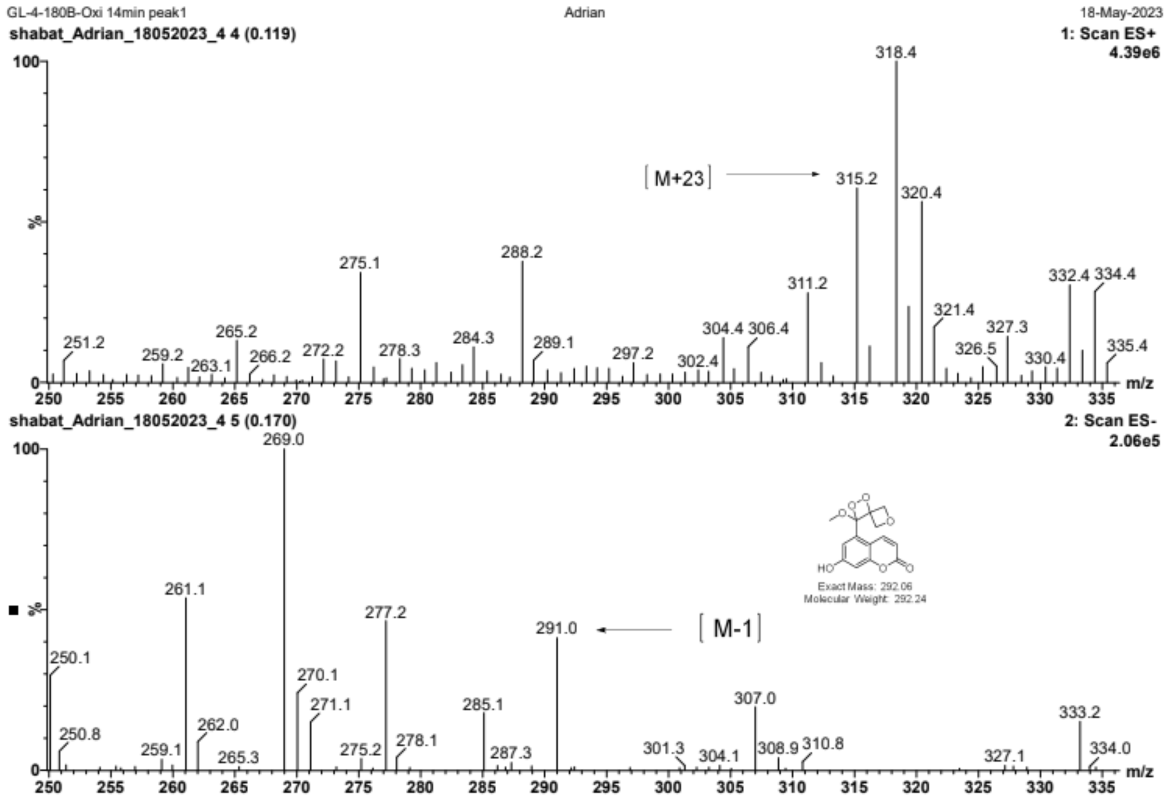

β-gal compounds

Compound 1d

^{1}H-NMR

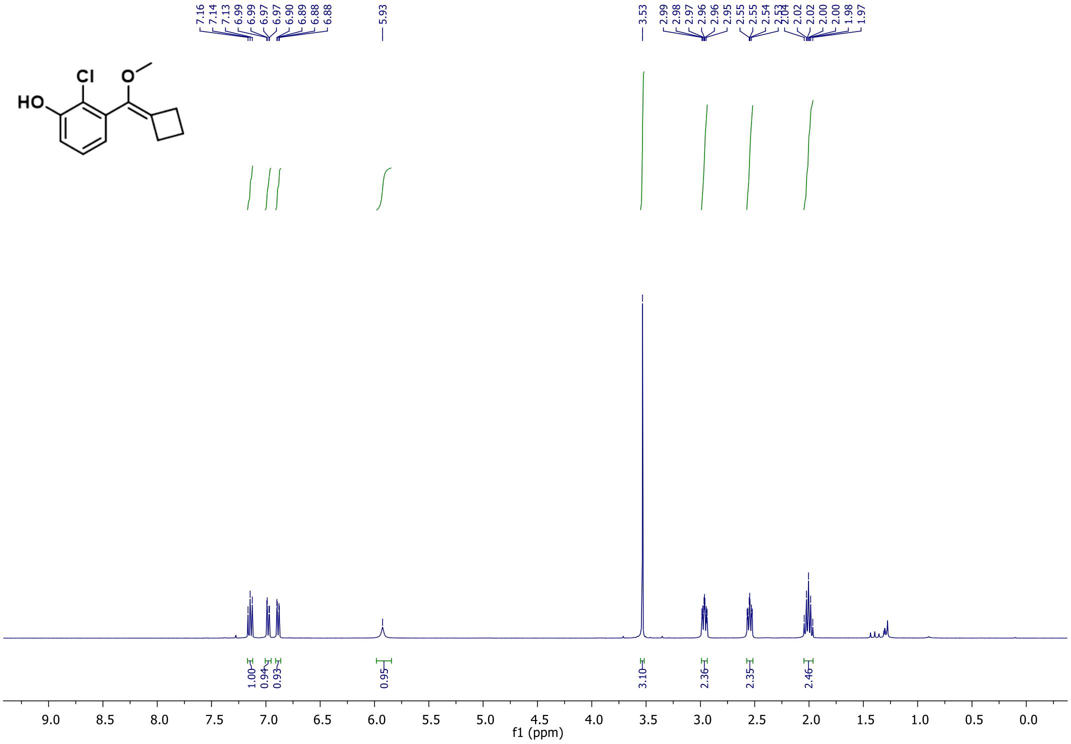

^{13}C-NMR

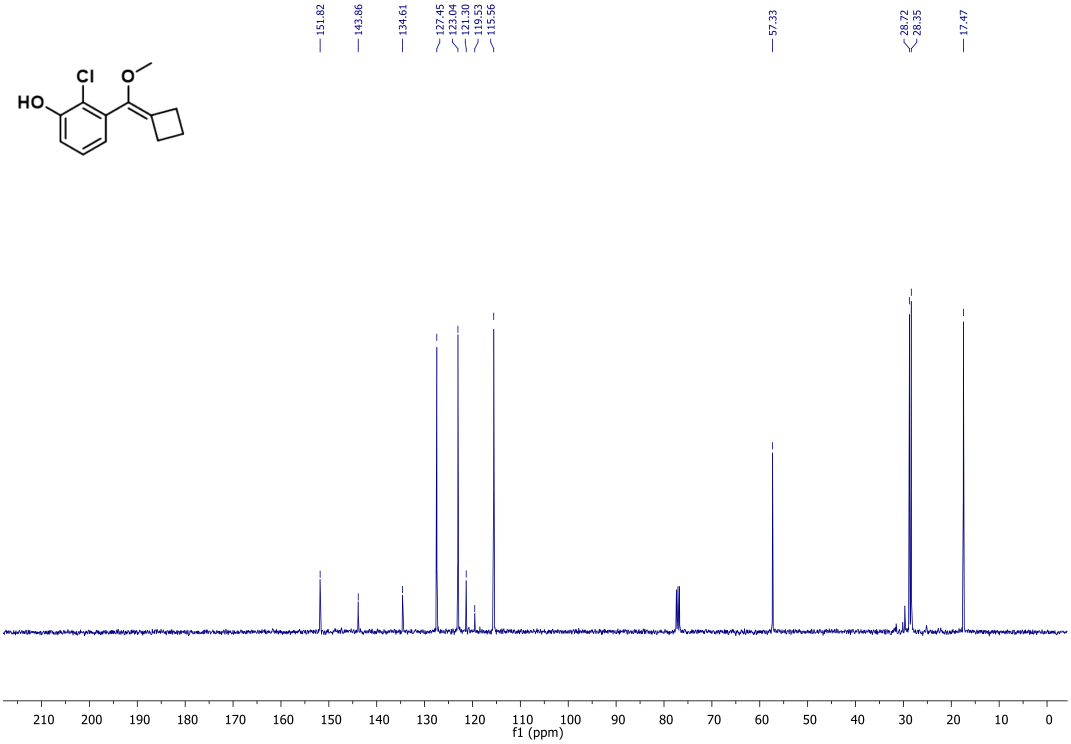

Mass spectra

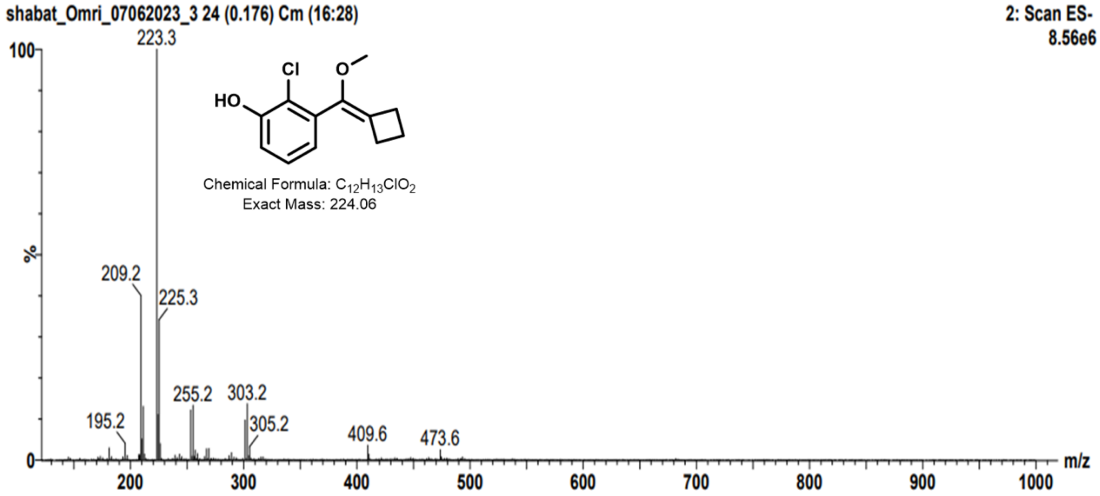

Compound 1e

^{1}H-NMR of

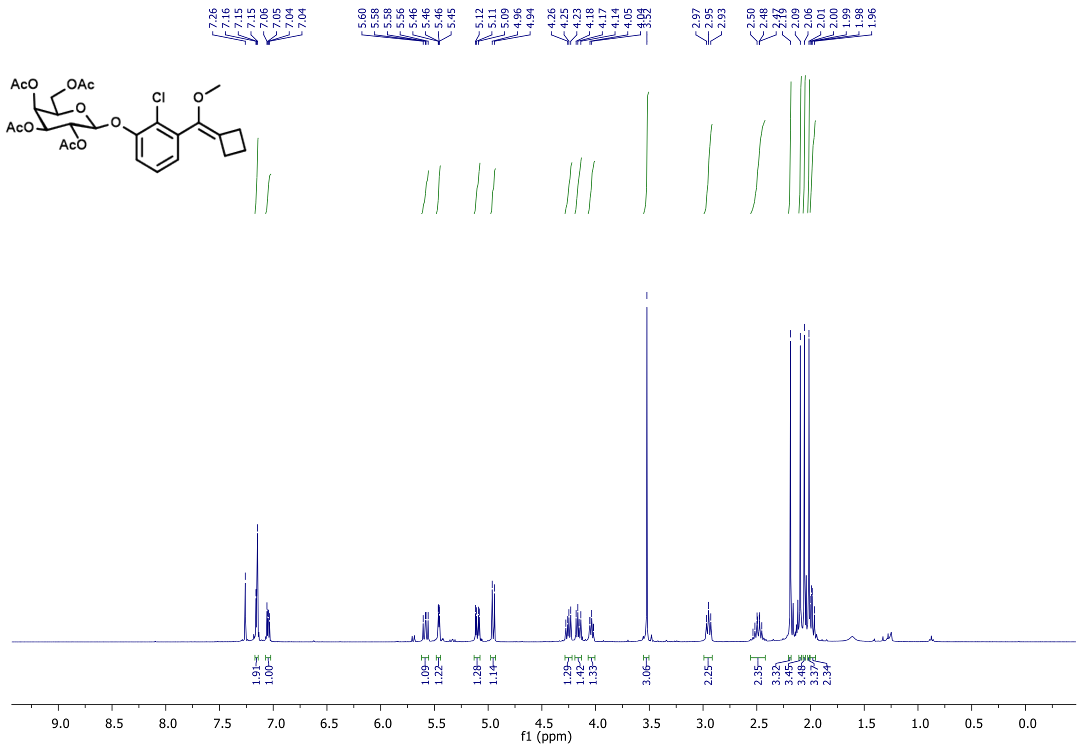

^{13}C-NMR

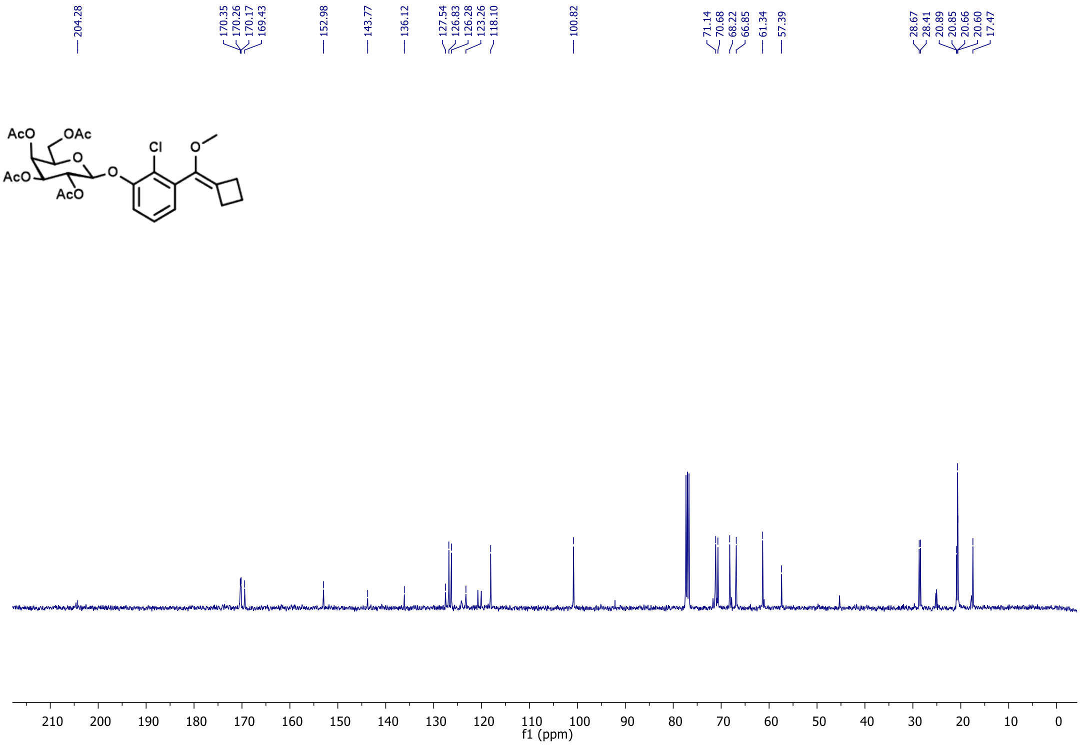

Mass spectra

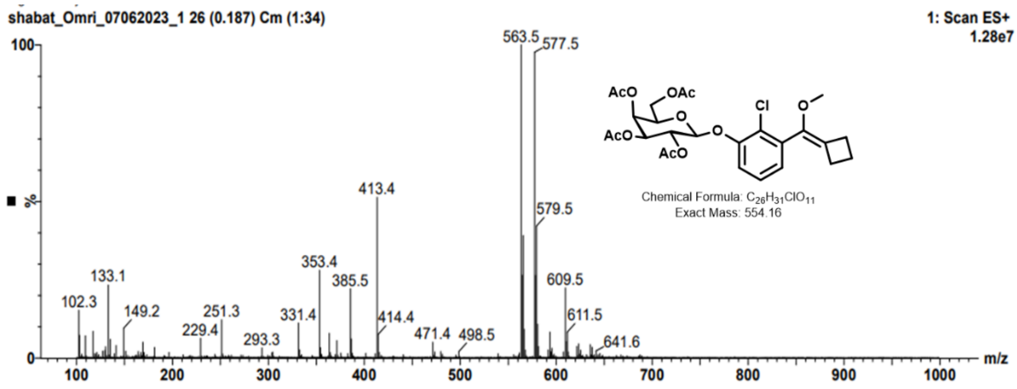

Probe β-gal CB intermediate

Mass spectra

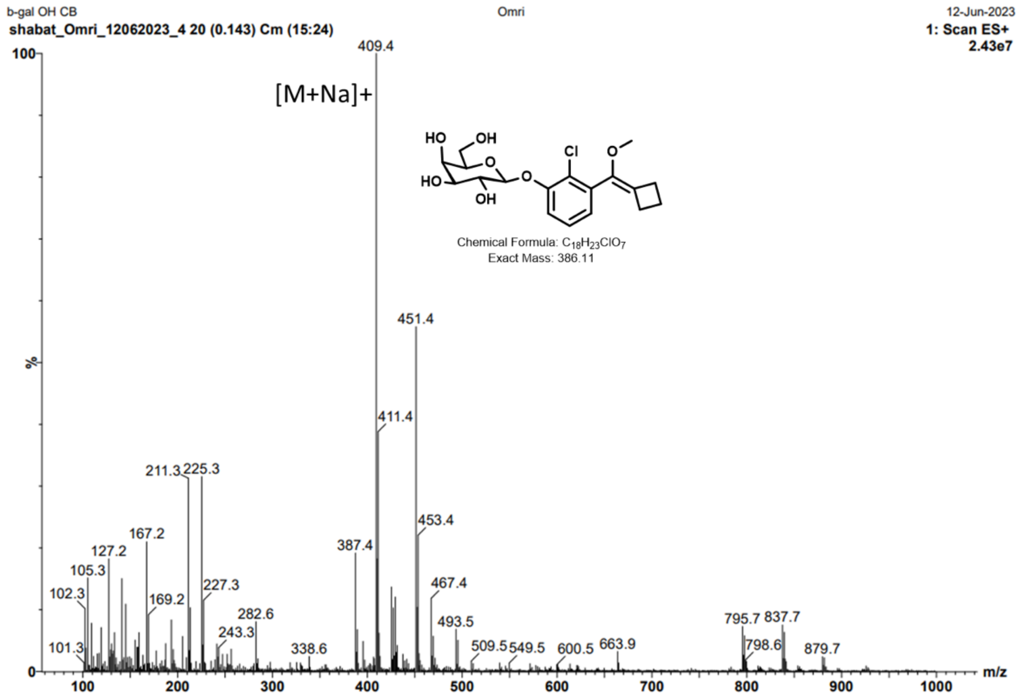

Probe β-gal CB

^{1}H-NMR

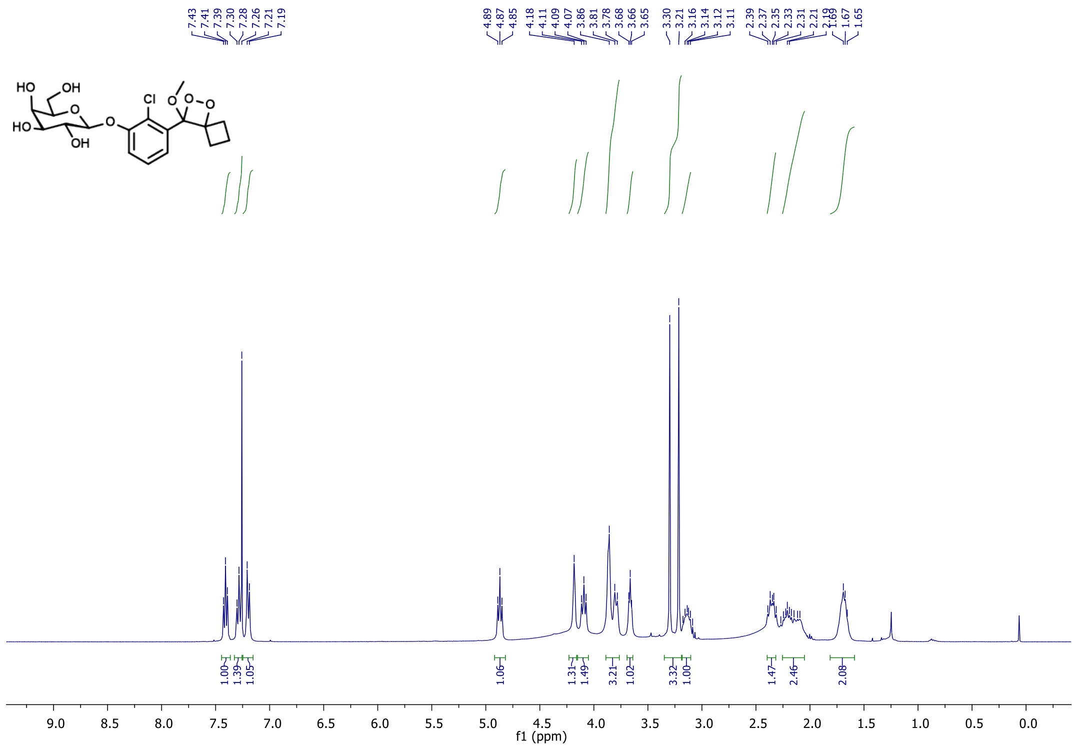

^{13}C-NMR

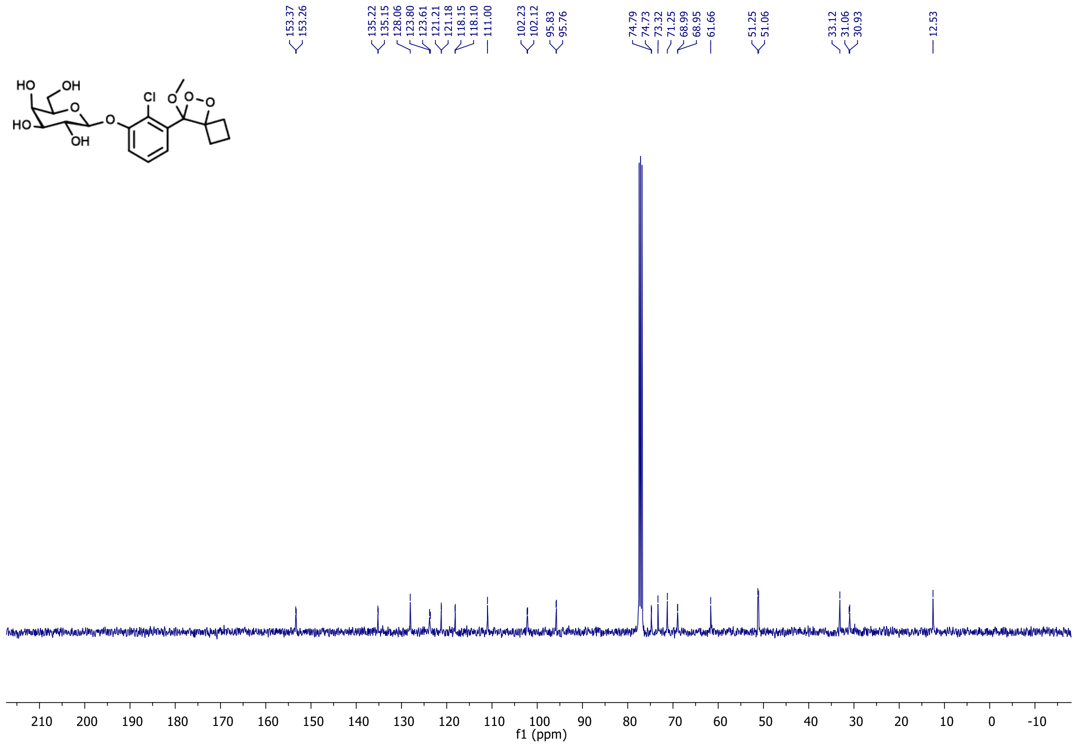

3D HPLC spectra (30-100% ACN in water, 0.1%TFA)

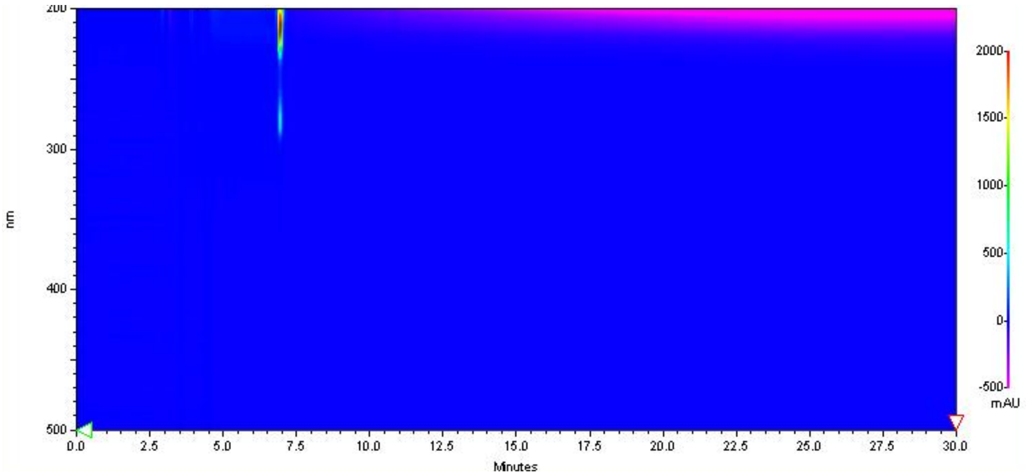

2D HPLC spectra (Absorbance measured at 277nm)

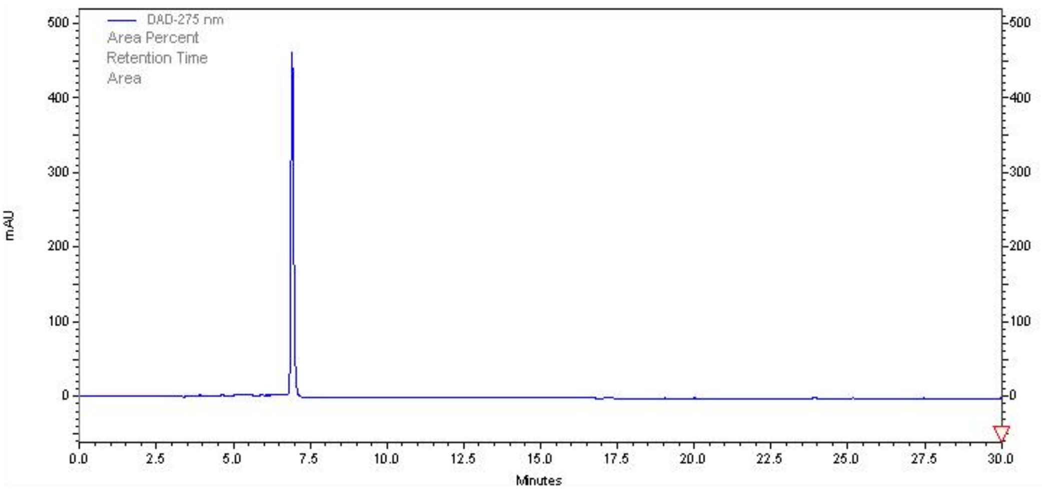

Mass spectra

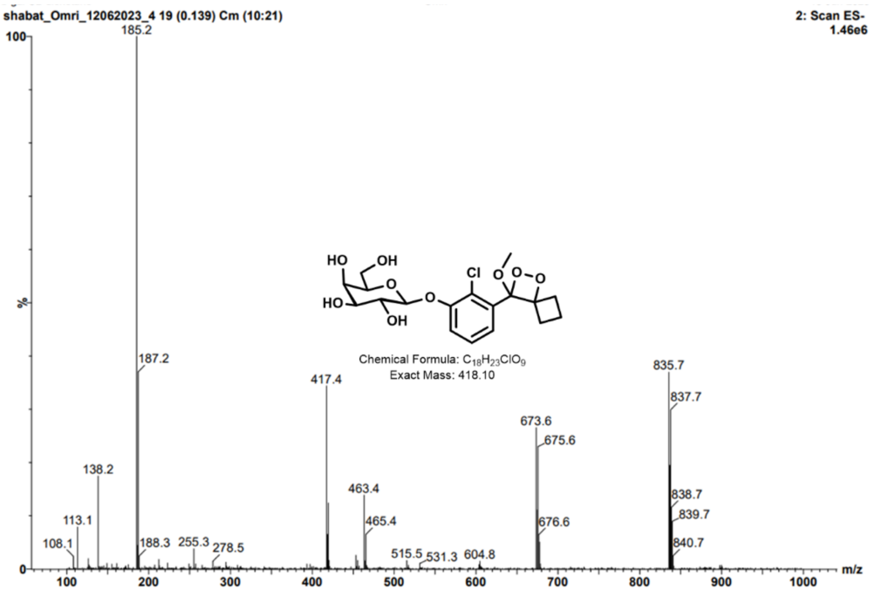

Compound 2d

^{1}H-NMR

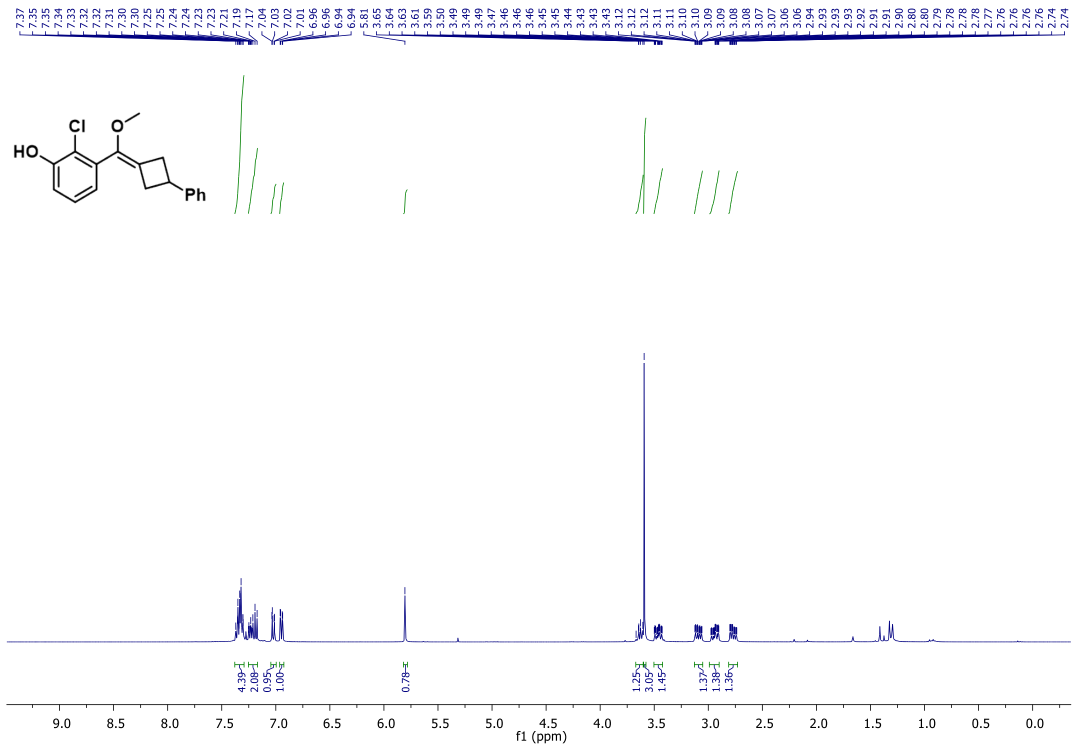

^{13}C-NMR

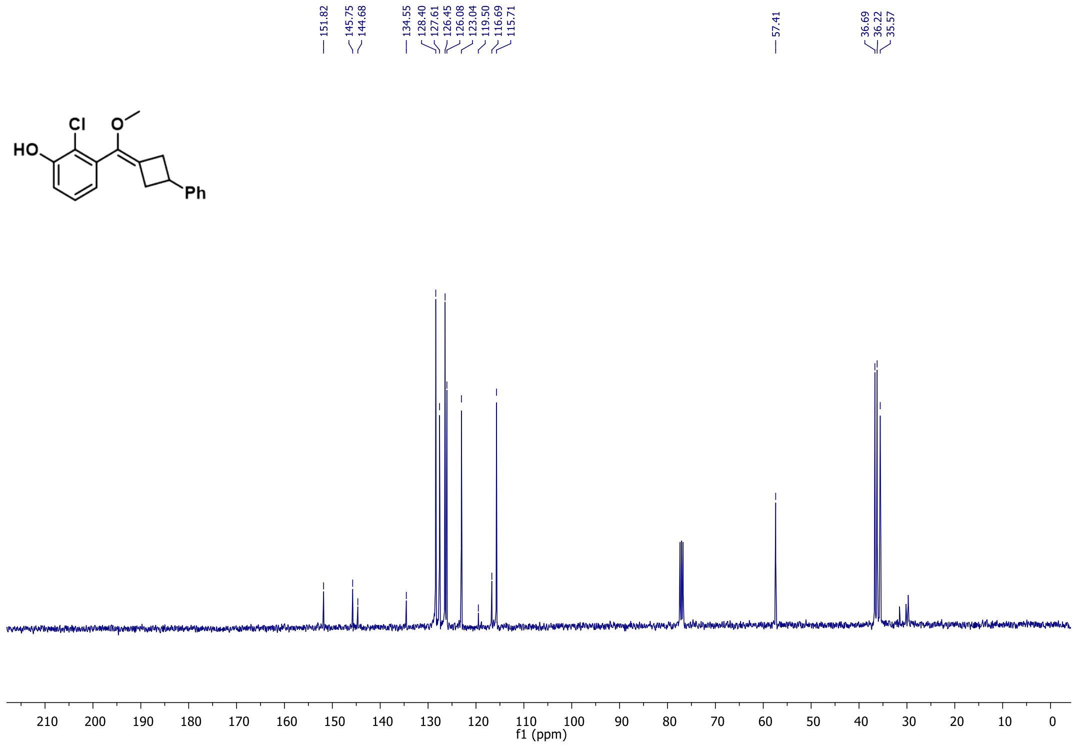

Mass spectra

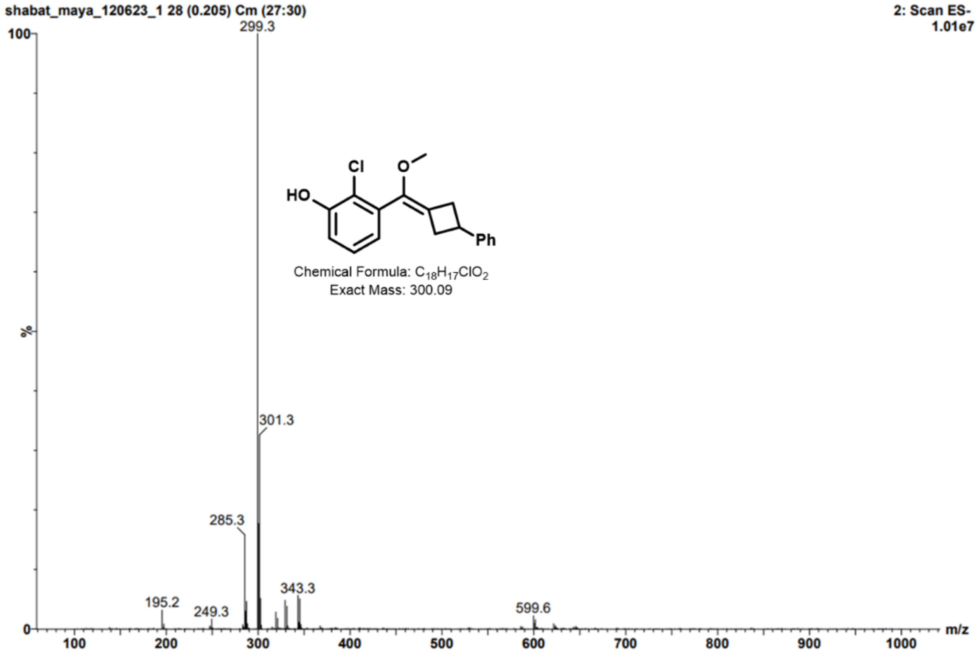

Compound 2e

^{1}H-NMR

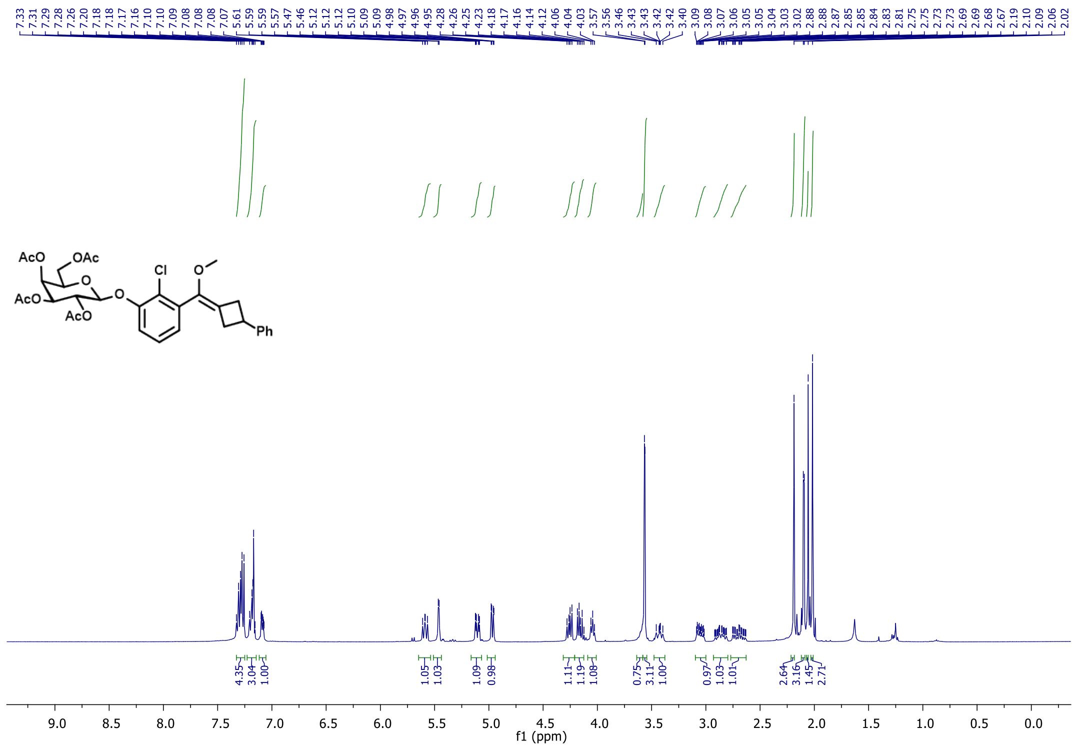

^{13}C-NMR

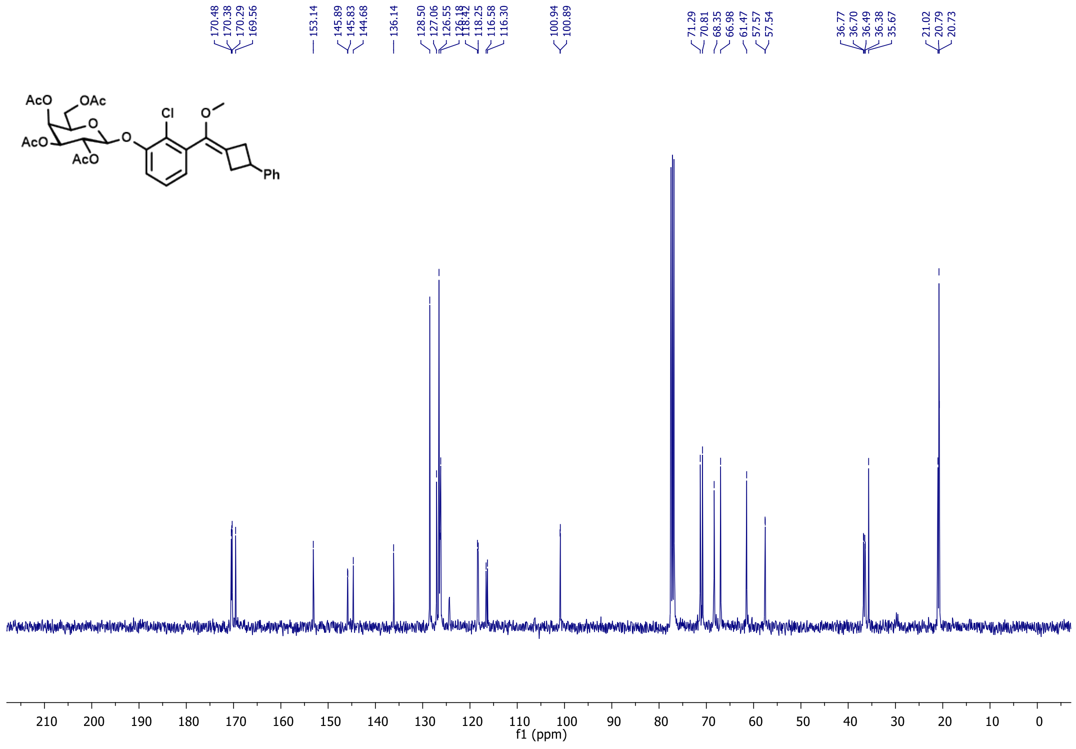

Mass spectra

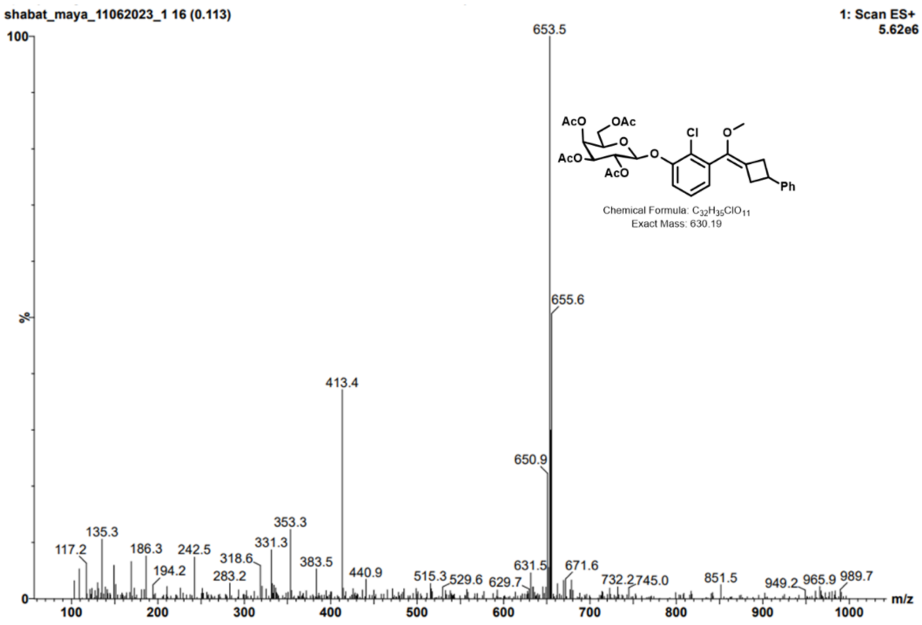

Probe β-gal 3-Ph intermediate

Mass spectra

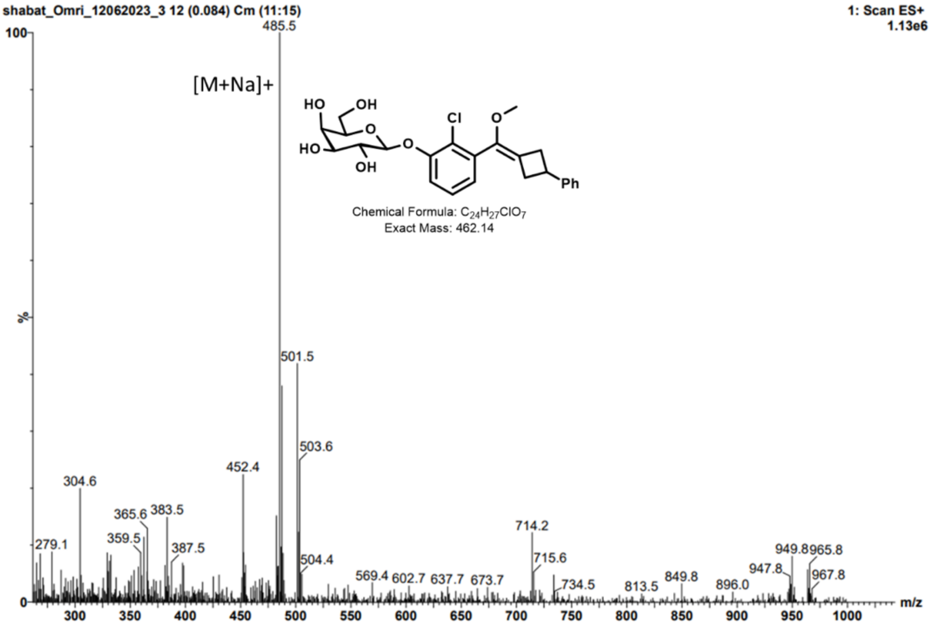

Probe β-gal 3-Ph

^{1}H-NMR

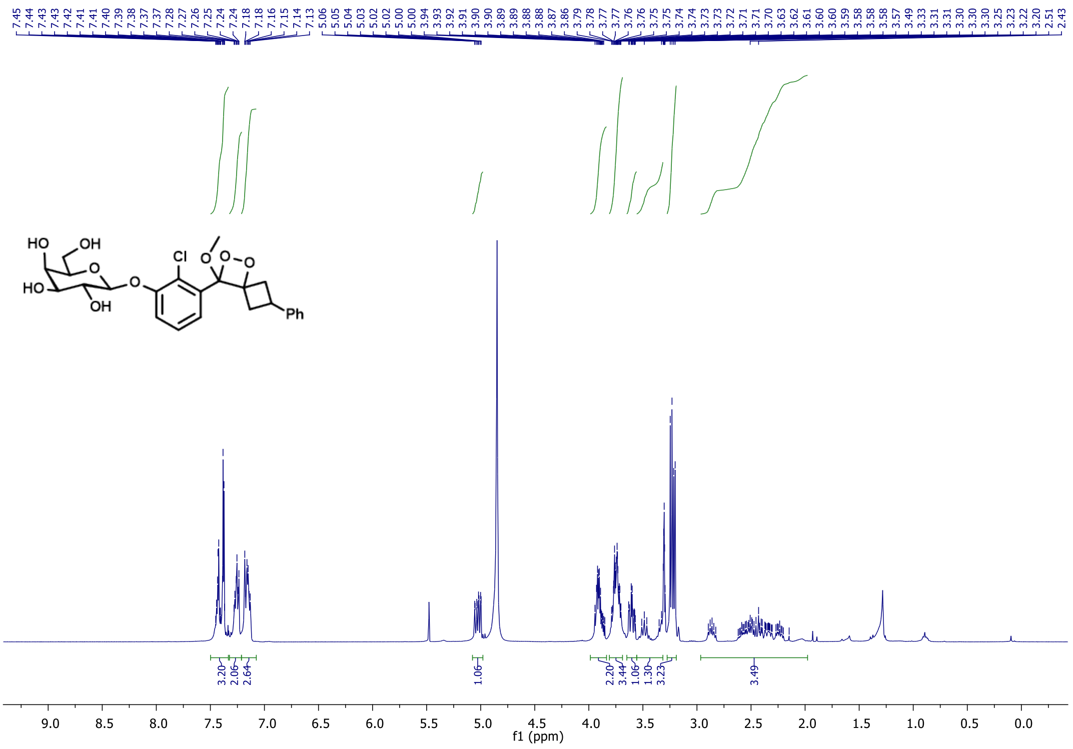

^{13}C-NMR

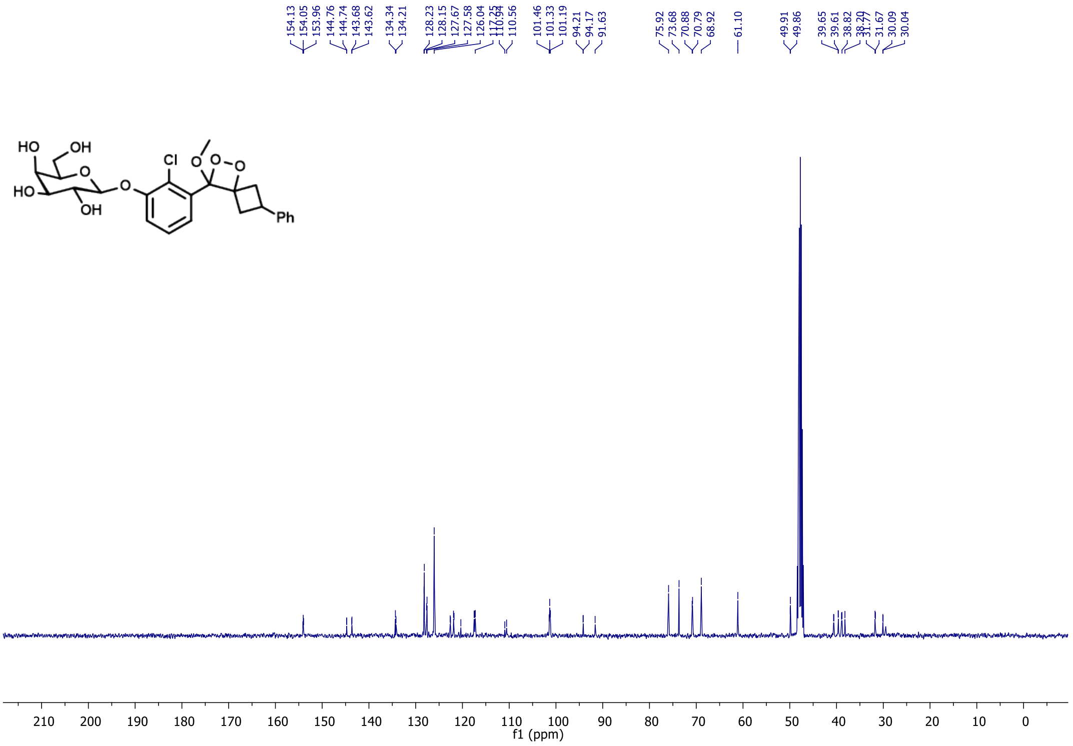

3D HPLC spectra (50-100% ACN in water, 0.1%TFA)

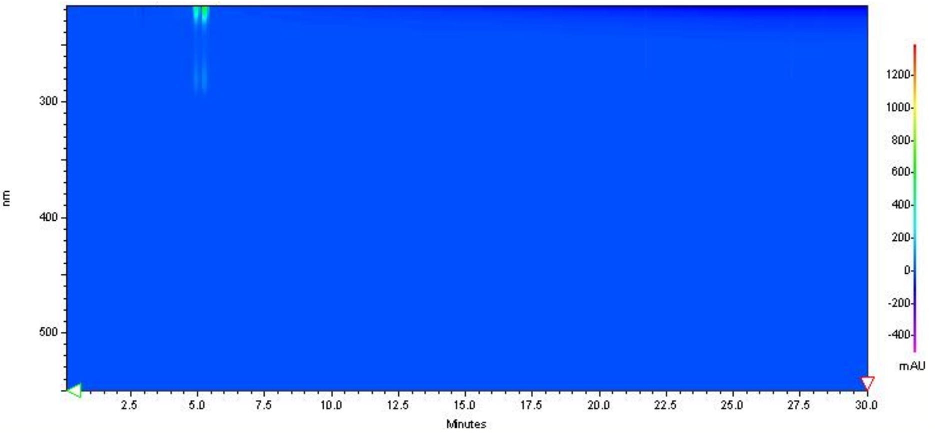

2D HPLC spectra (Absorbance measured at 277nm)

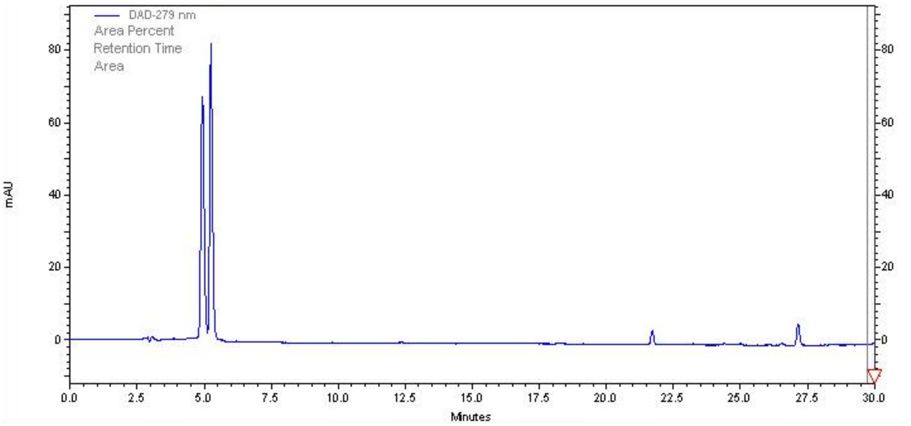

Mass spectra

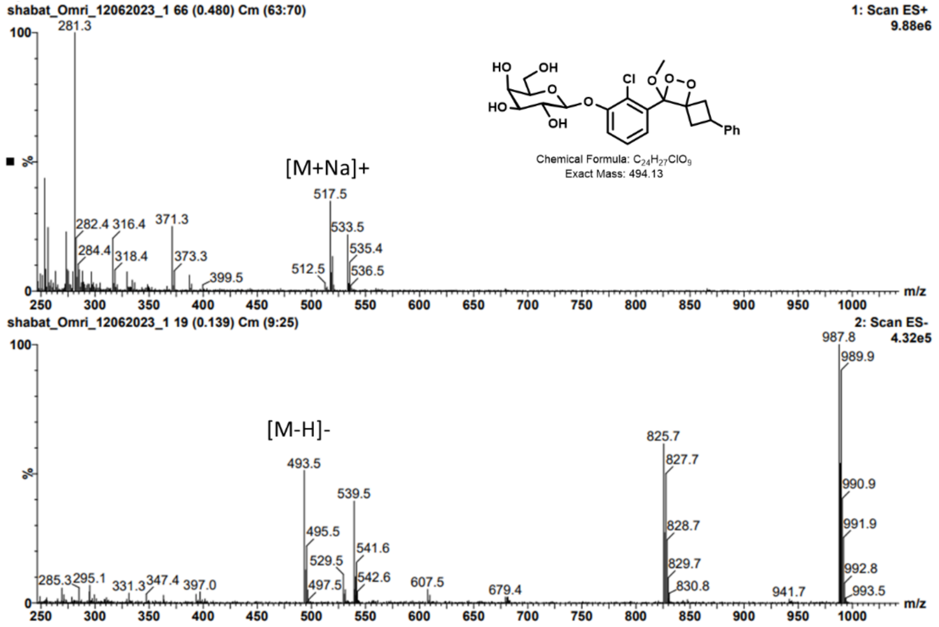

Compound 3d

^{1}H-NMR

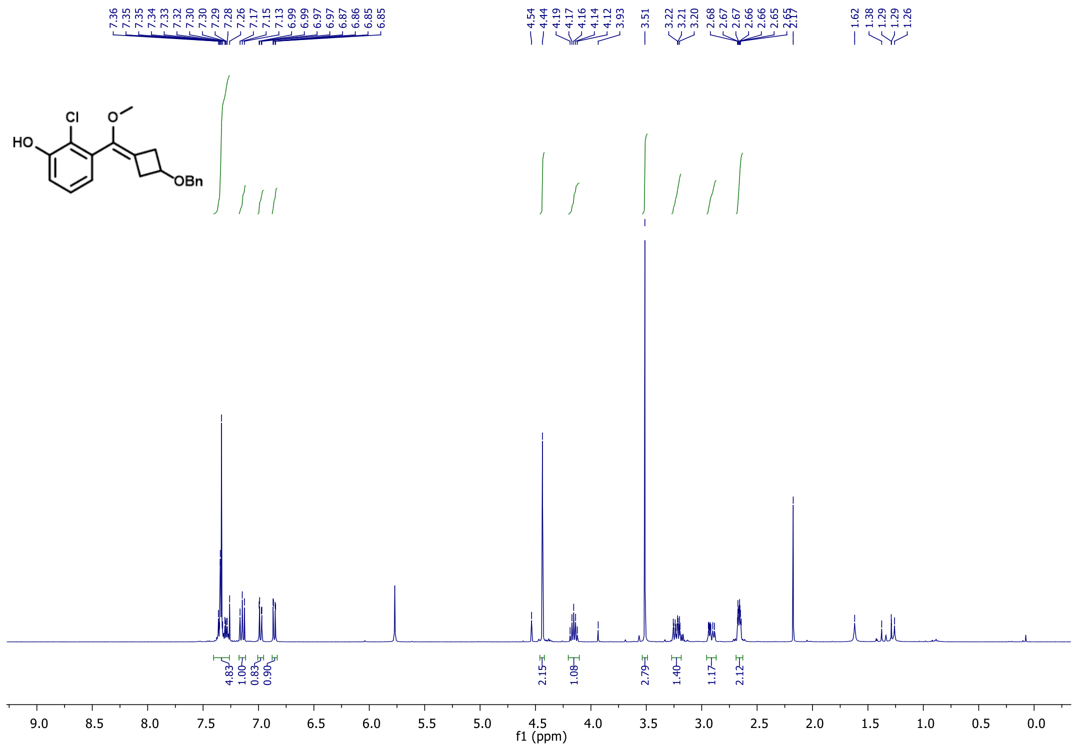

^{13}C-NMR

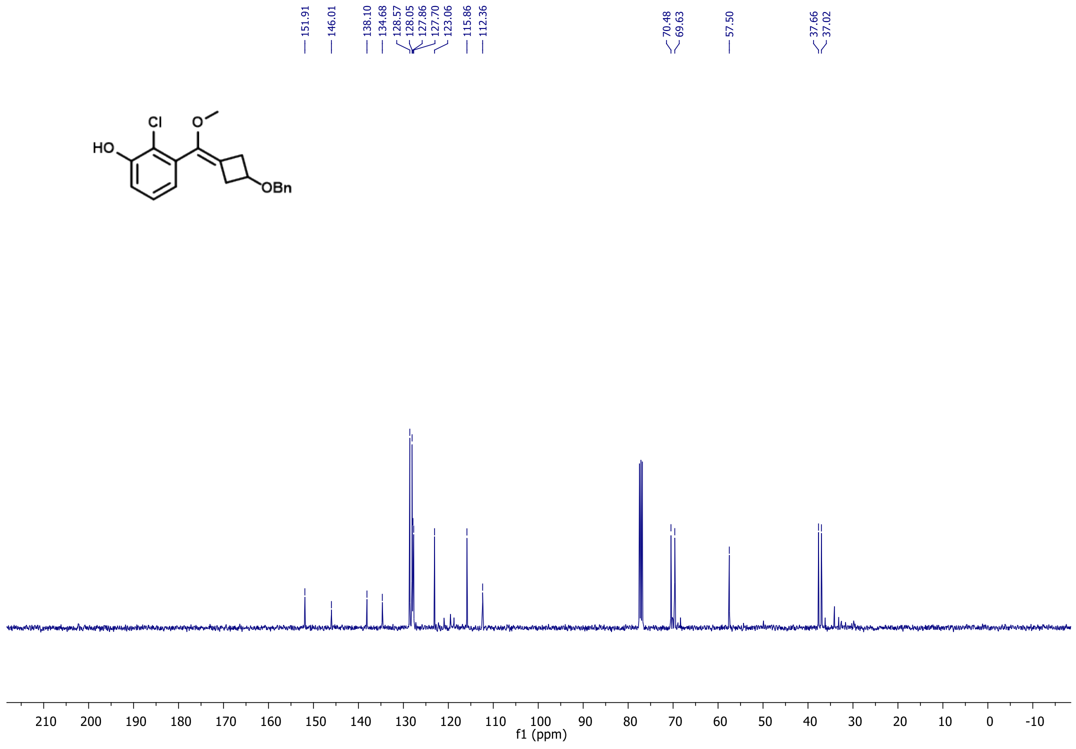

Mass spectra

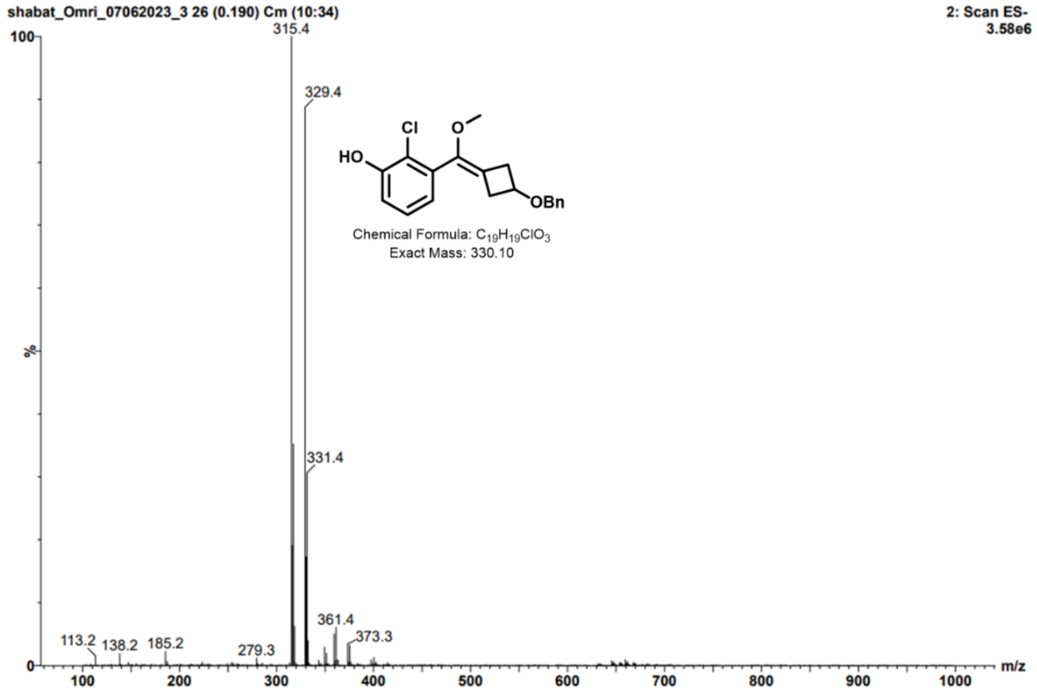

Compound 3e

^{1}H-NMR

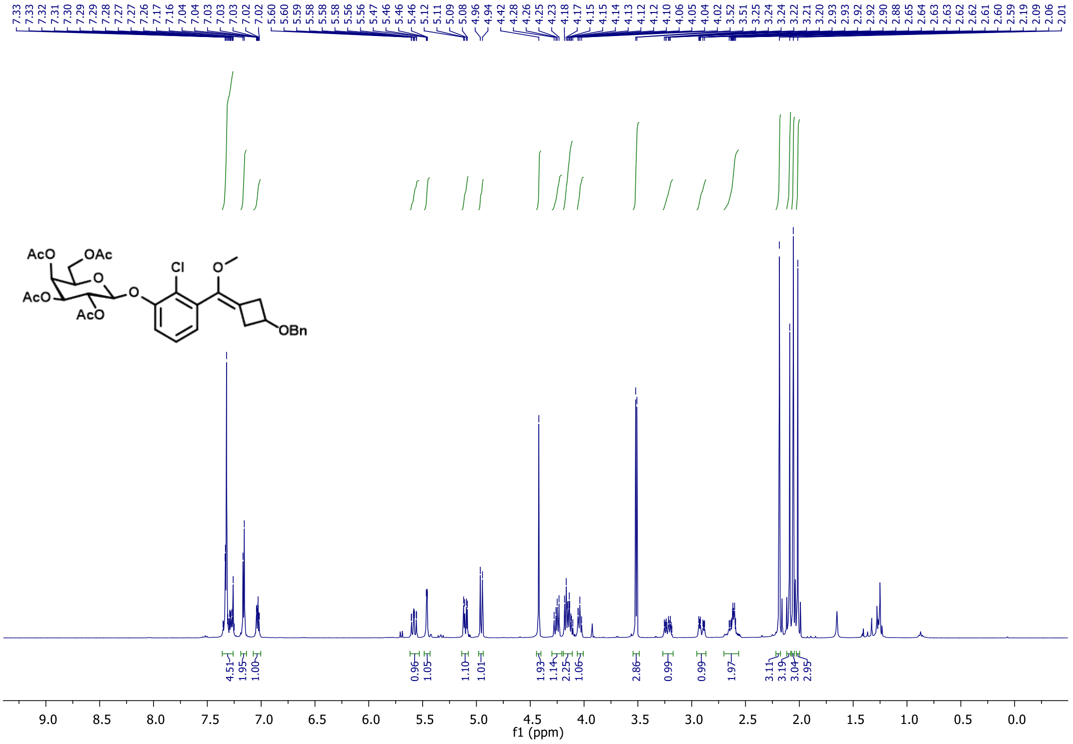

^{13}C-NMR

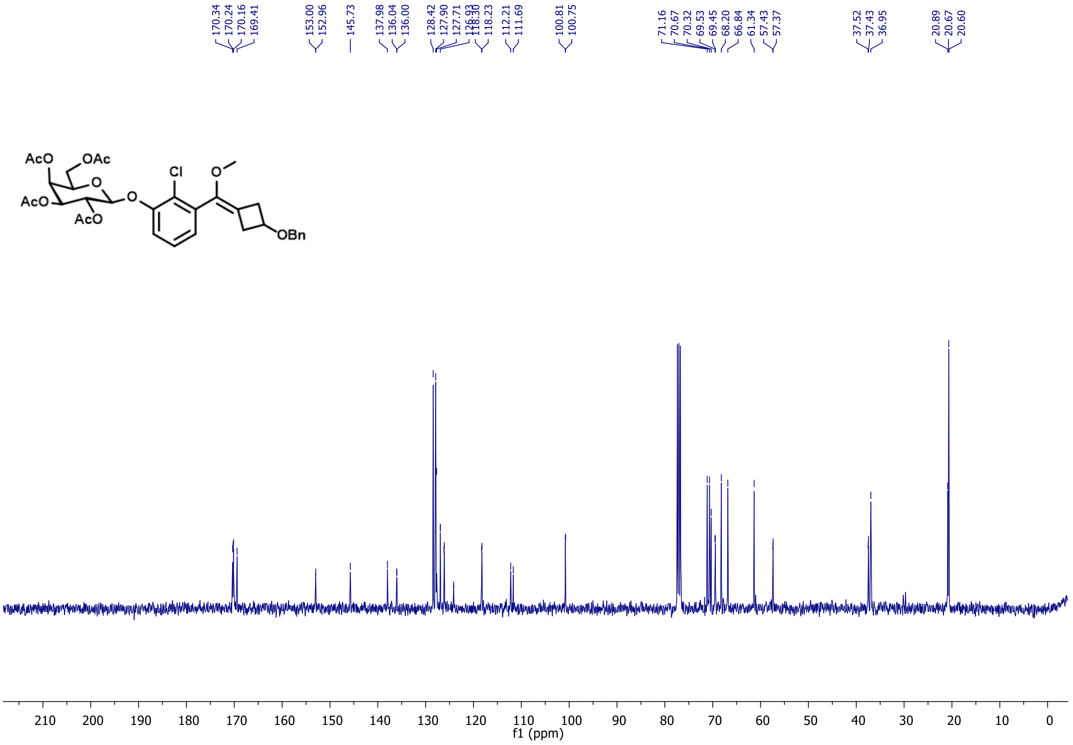

Mass spectra

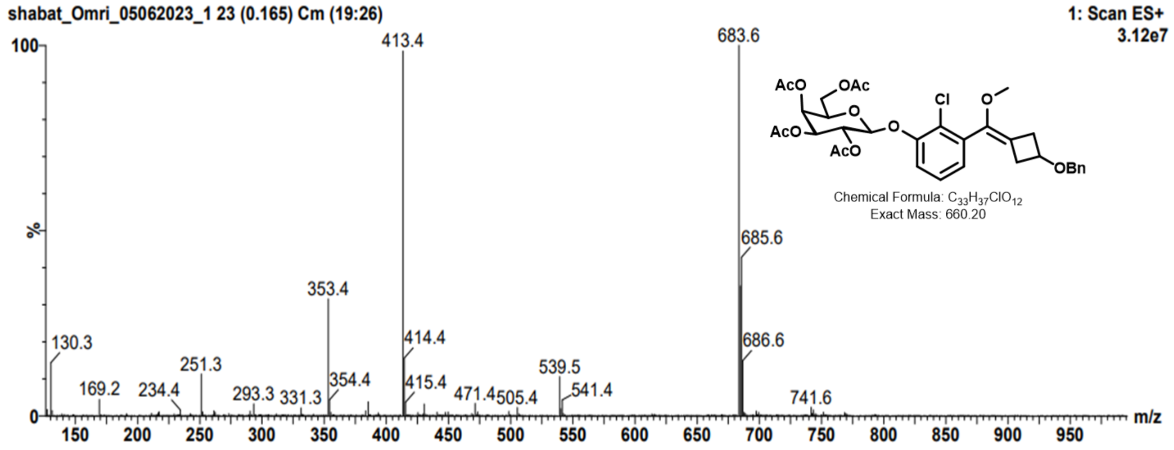

Probe β-gal OBn

^{1}H-NMR

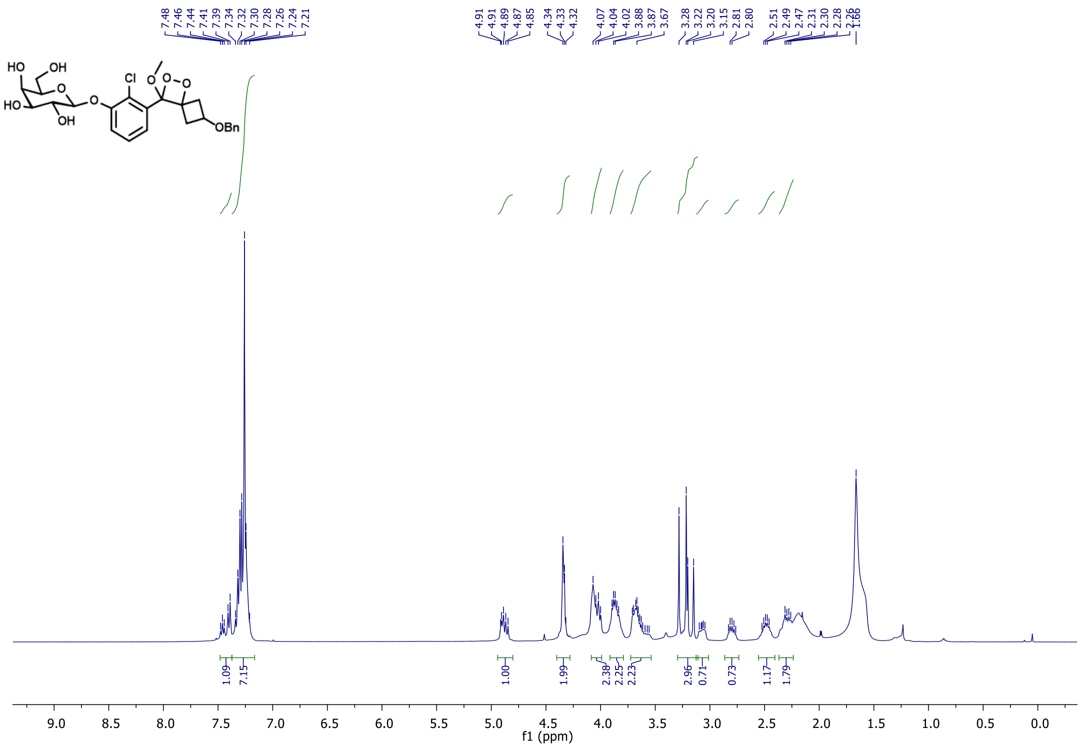

^{13}C-NMR

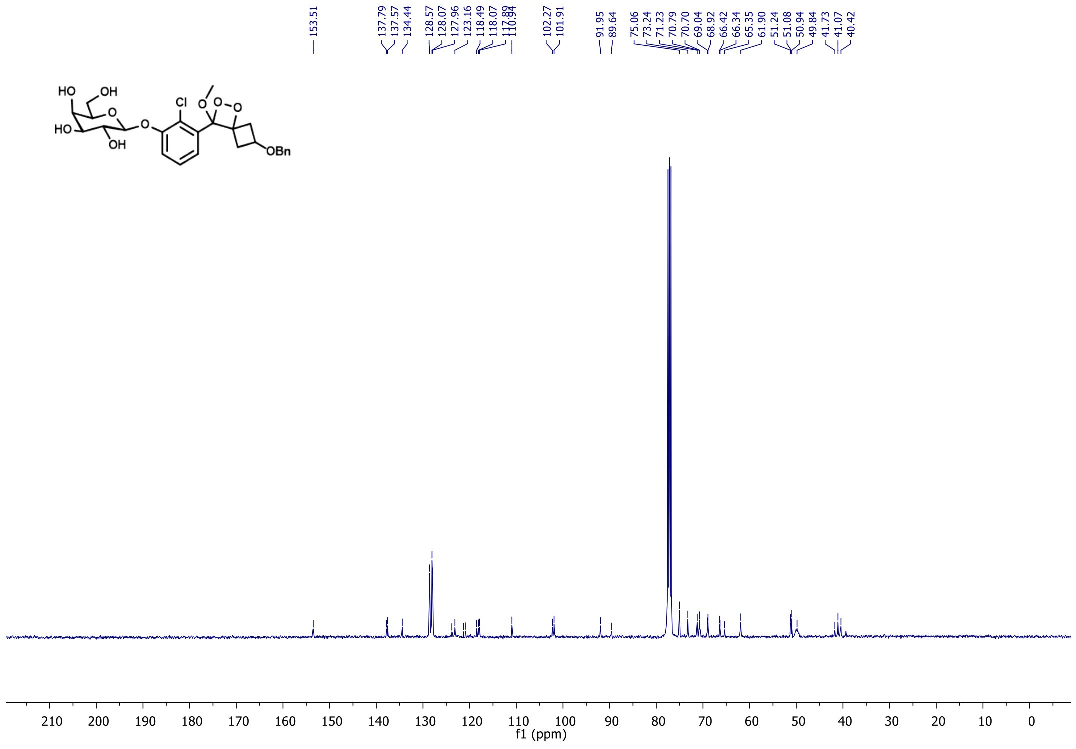

3D HPLC spectra (30-100% ACN in water, 0.1%TFA)

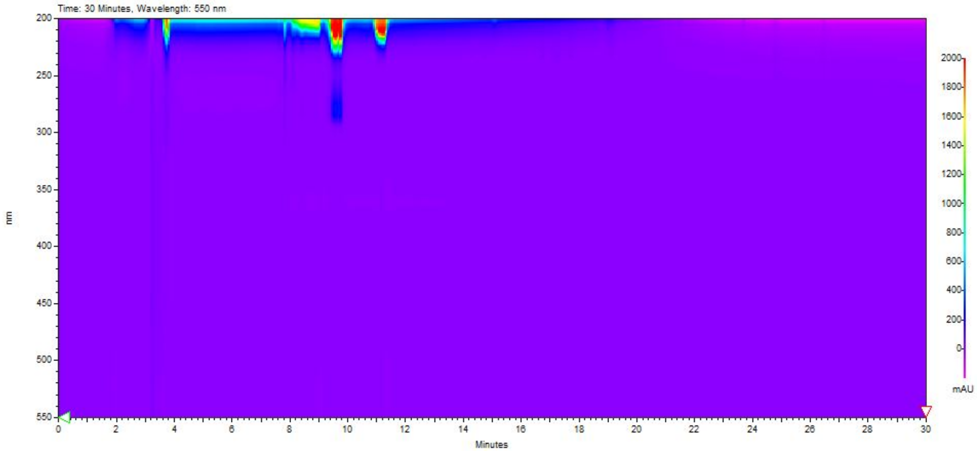

2D HPLC spectra (Absorbance measured at 277nm)

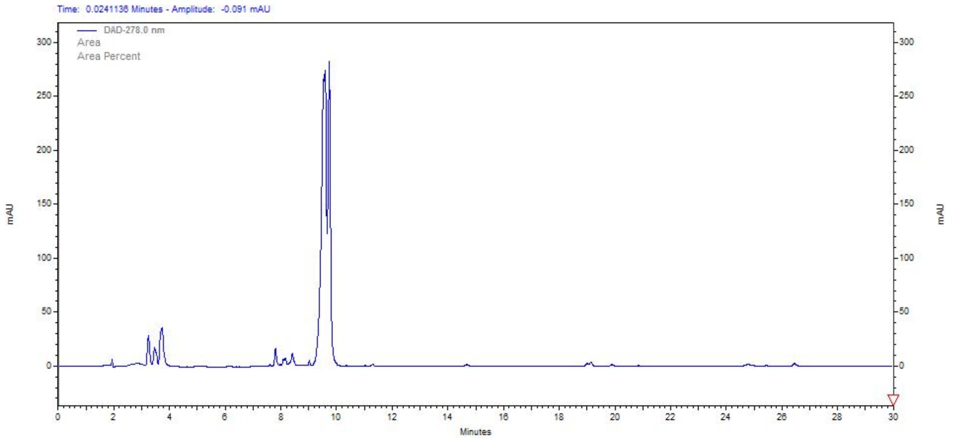

Mass spectra

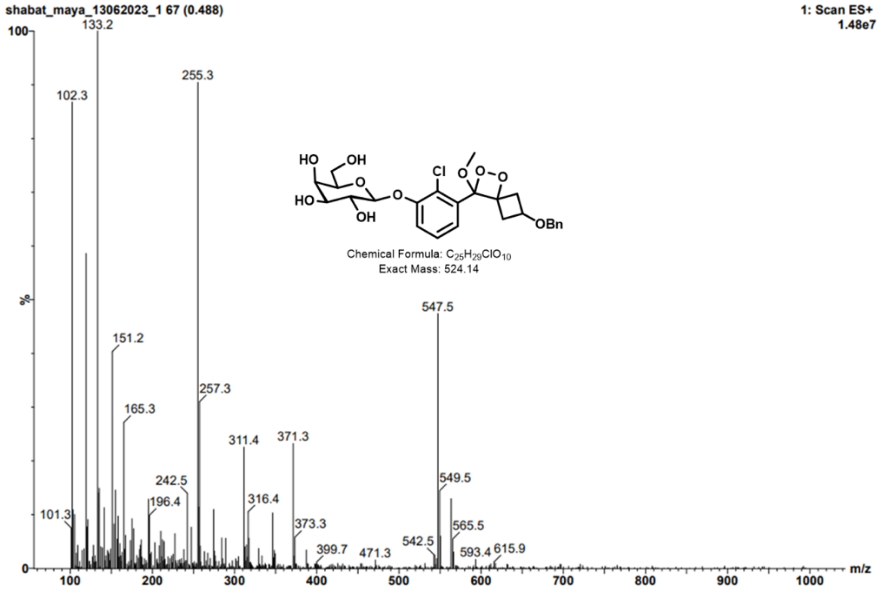

Compound 4d

^{1}H-NMR

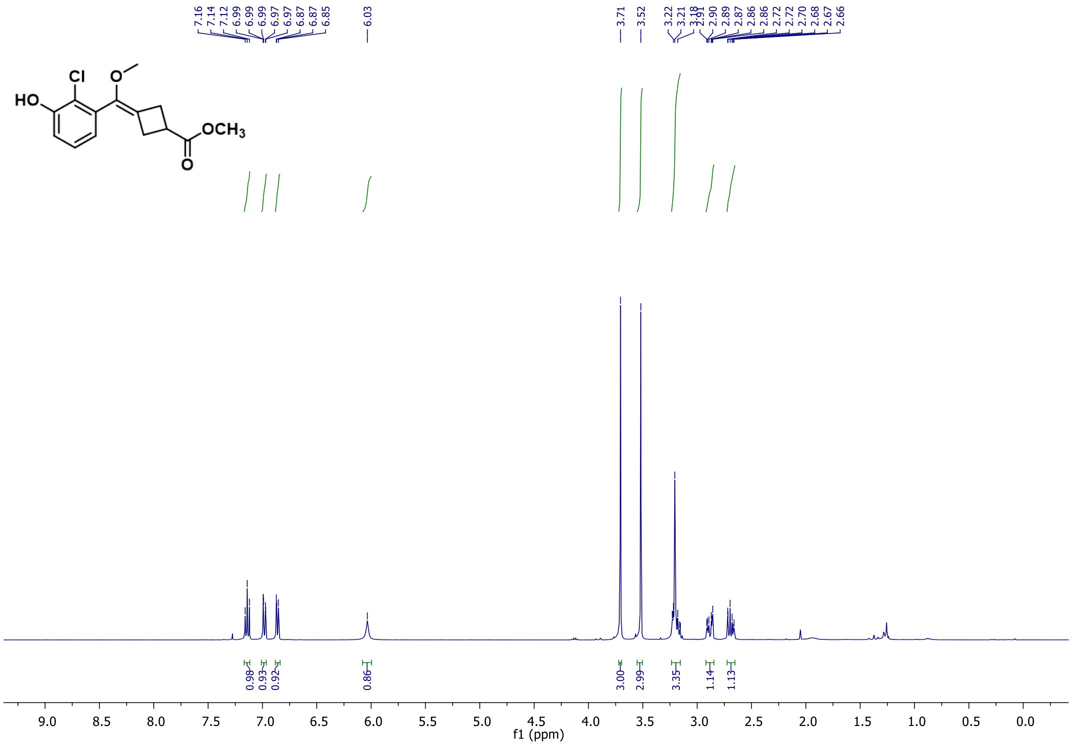

^{13}C-NMR

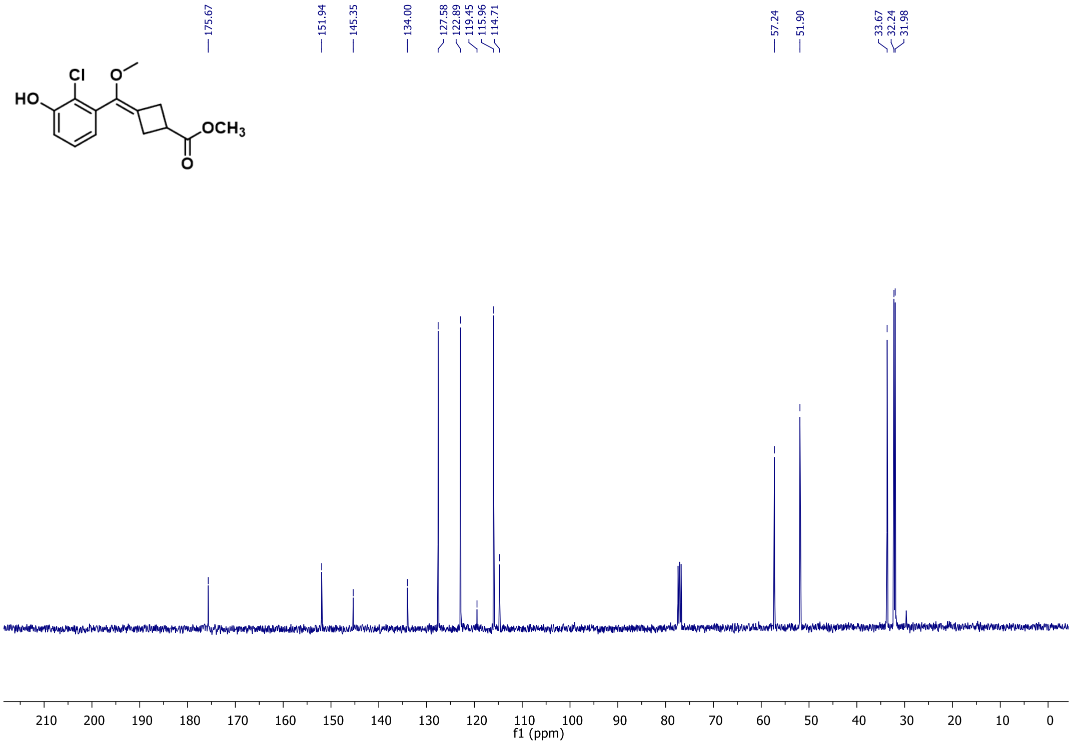

Mass spectra

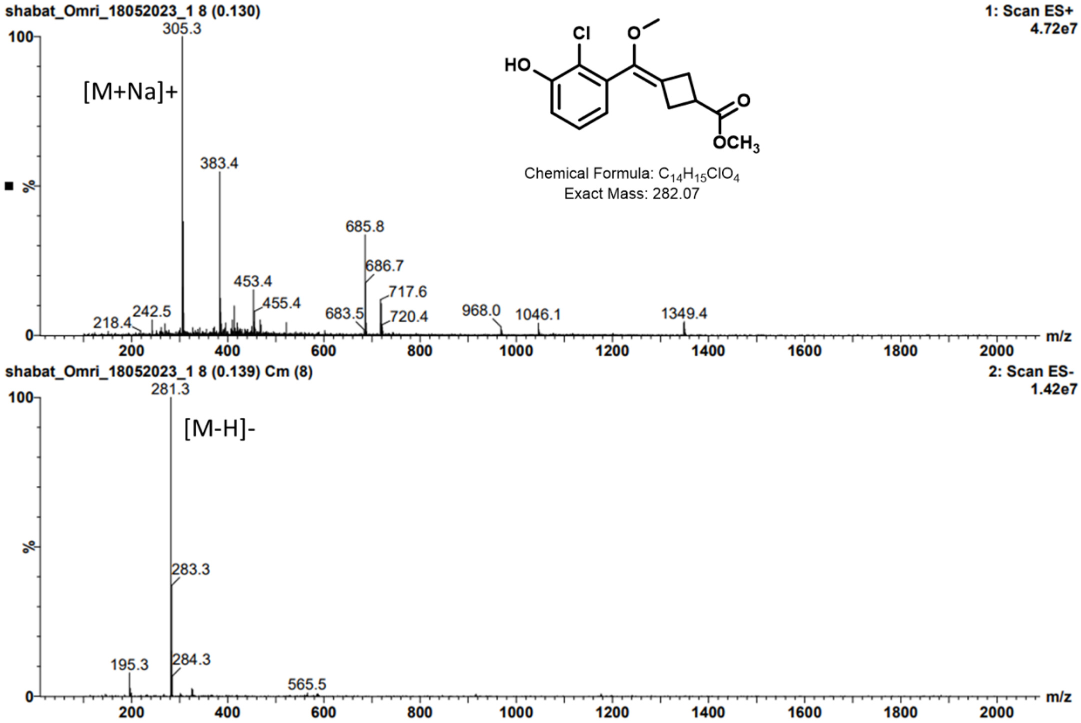

Compound 4e

^{1}H-NMR

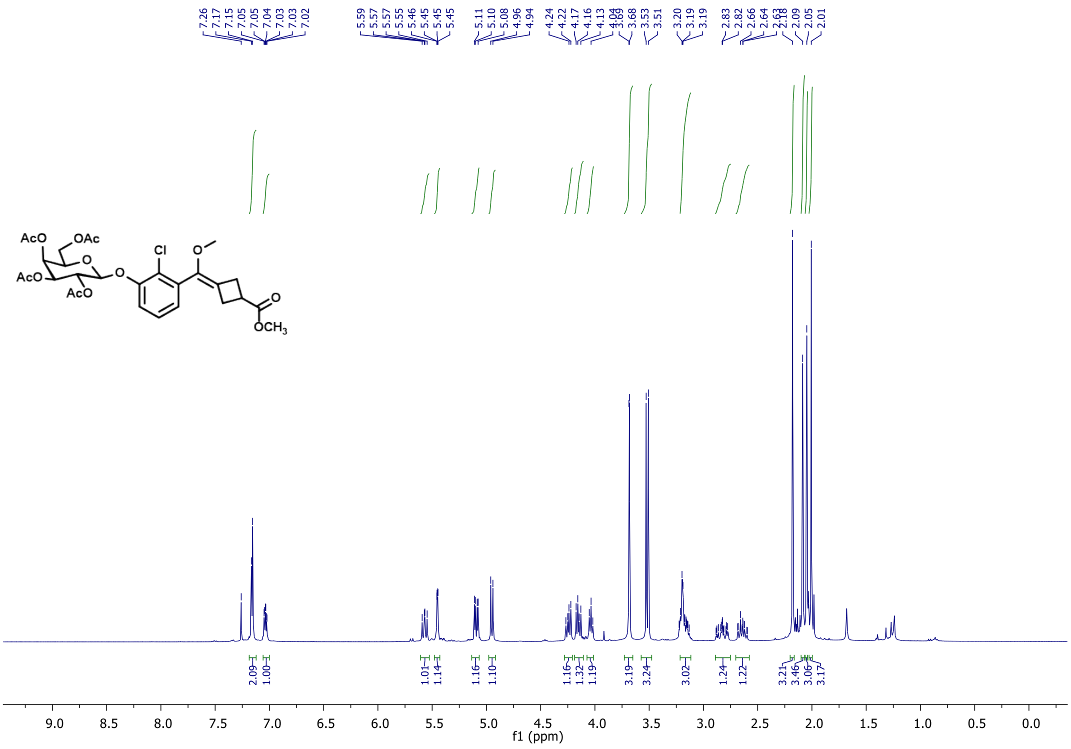

^{13}C-NMR

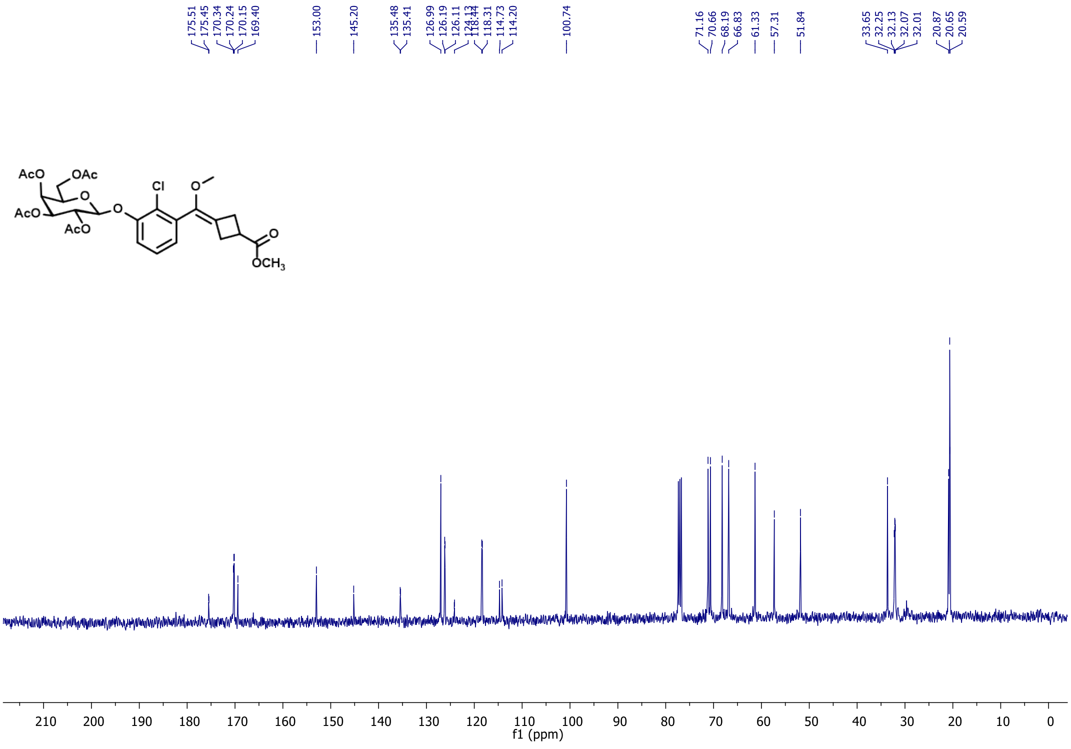

Mass spectra

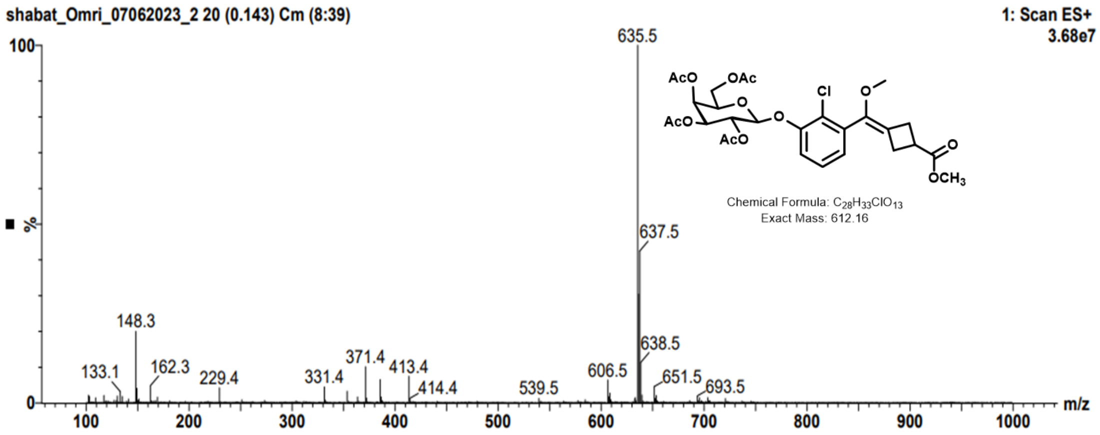

Probe β-gal 3-Ester intermediate

Mass spectra

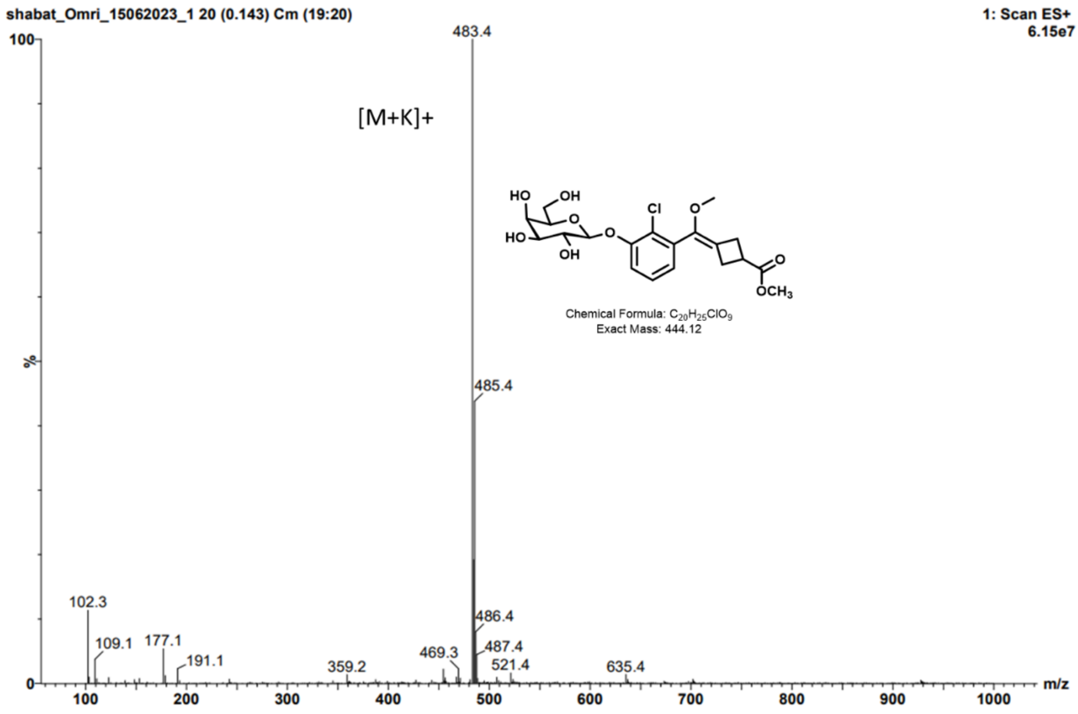

Probe β-gal 3-Ester

^{1}H-NMR

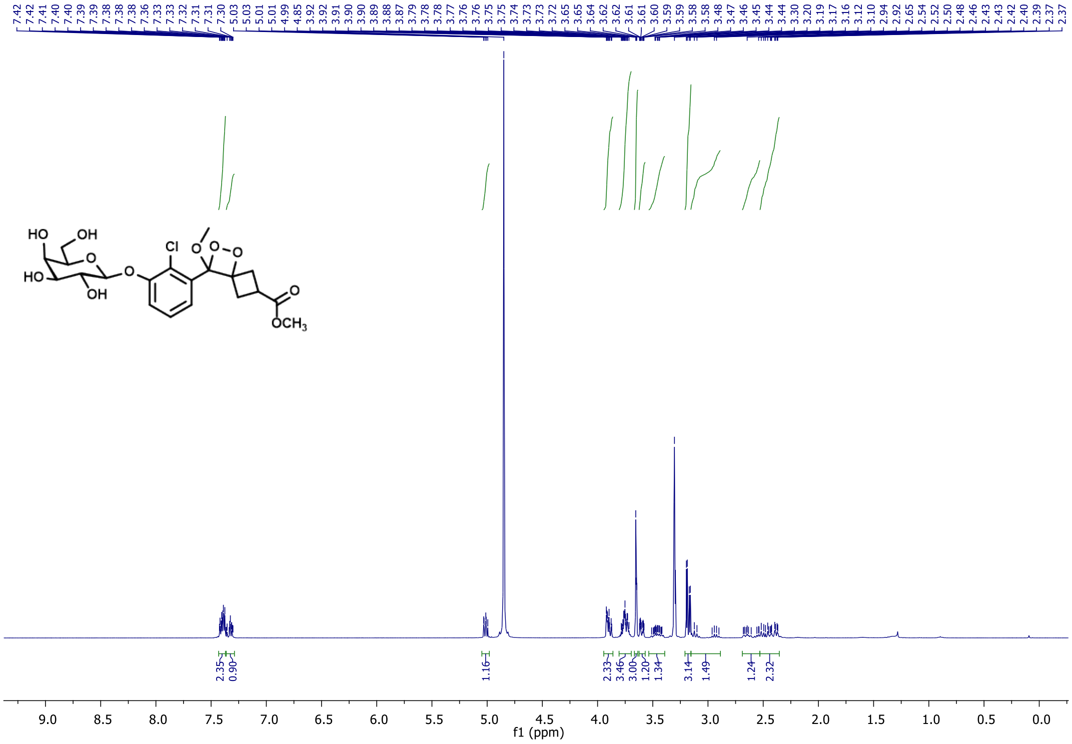

^{13}C-NMR

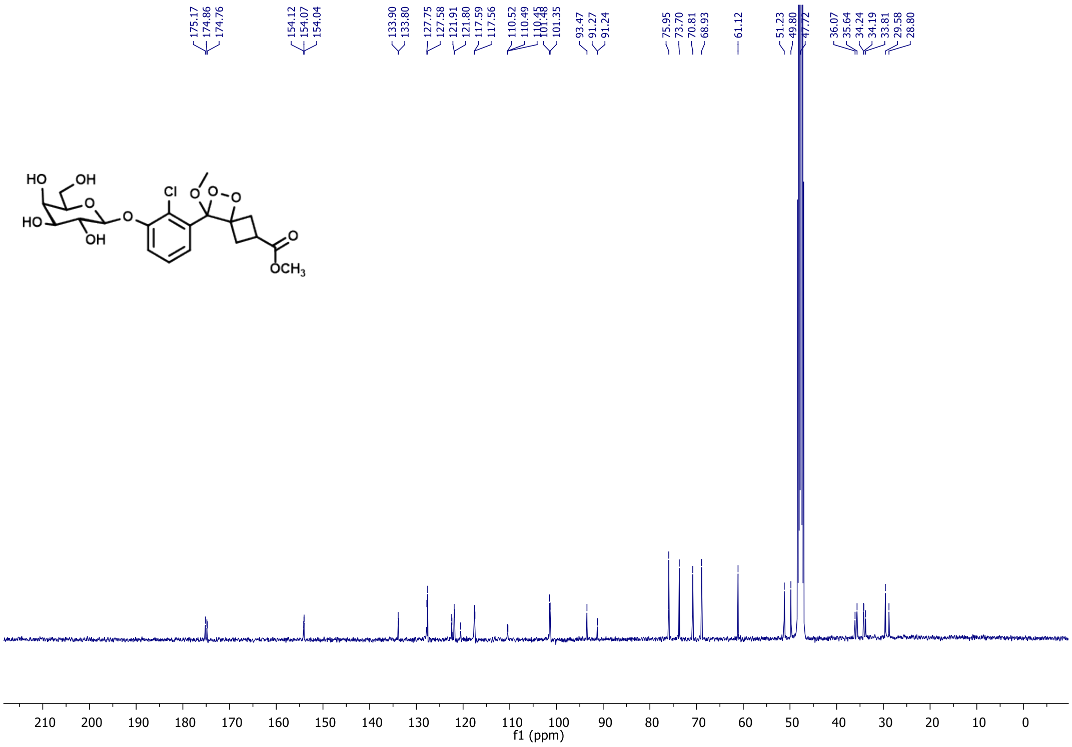

3D HPLC spectra (30-100% ACN in water, 0.1%TFA)

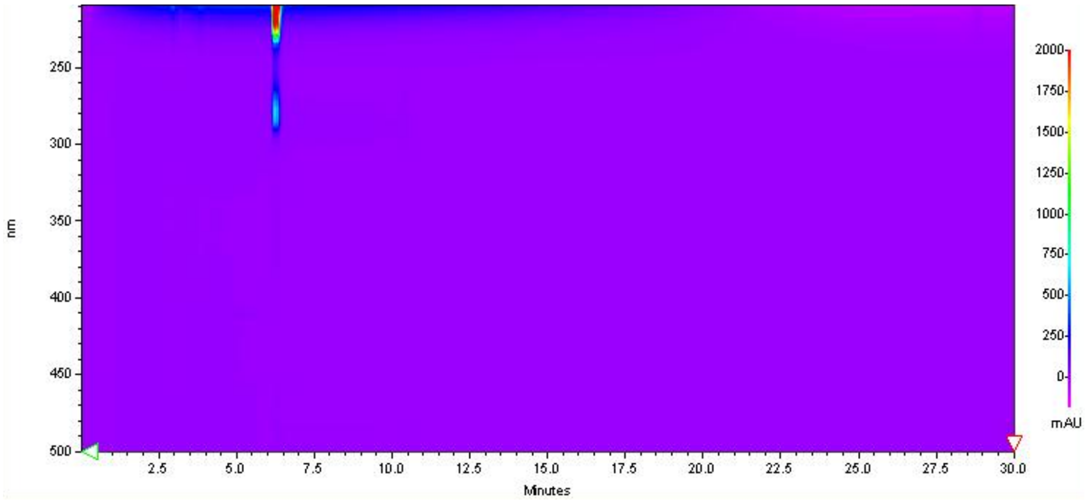

2D HPLC spectra (Absorbance measured at 277nm)

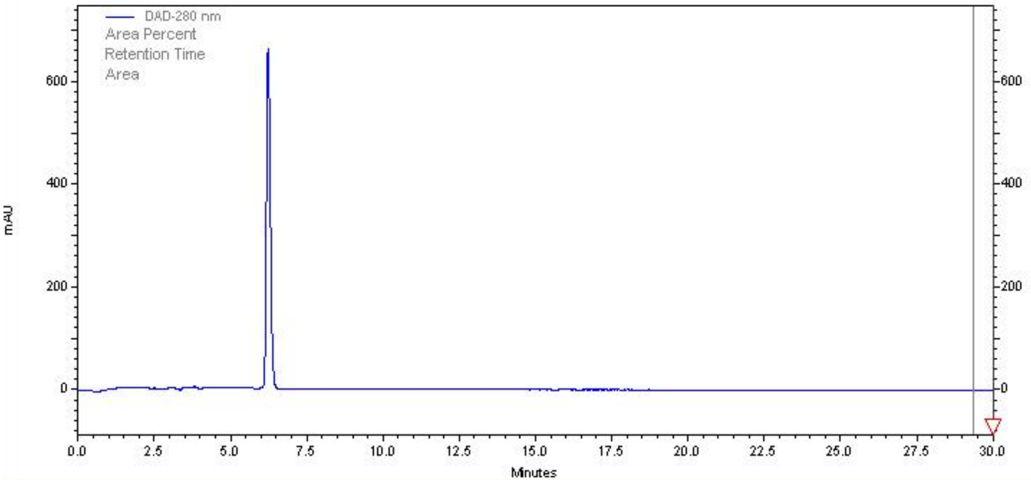

Mass spectra

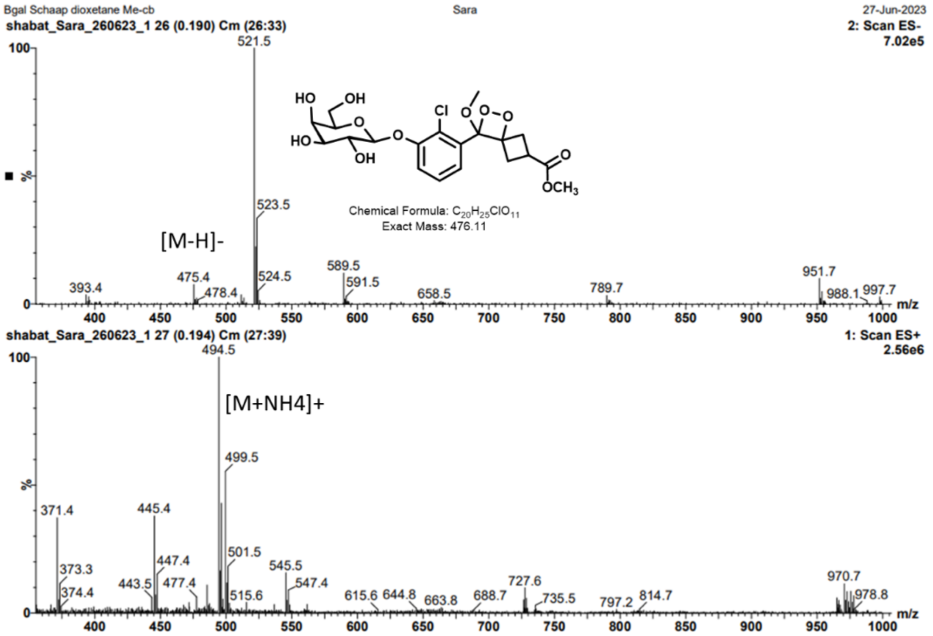

Compound 5d

^{1}H-NMR

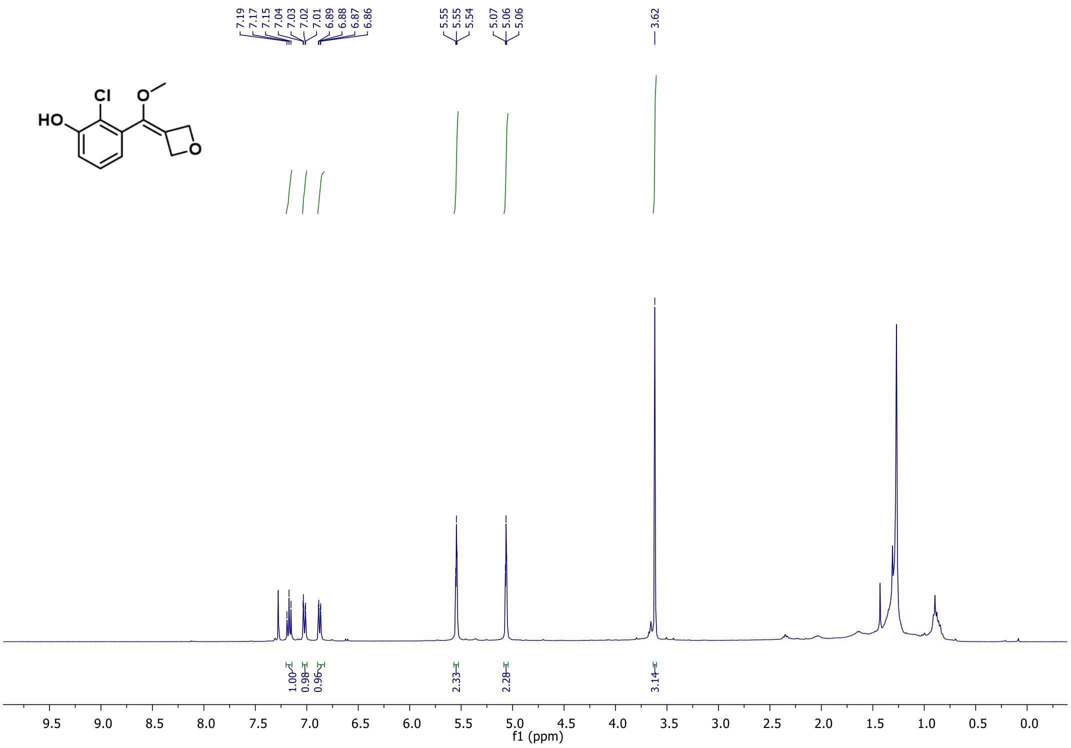

^{13}C-NMR

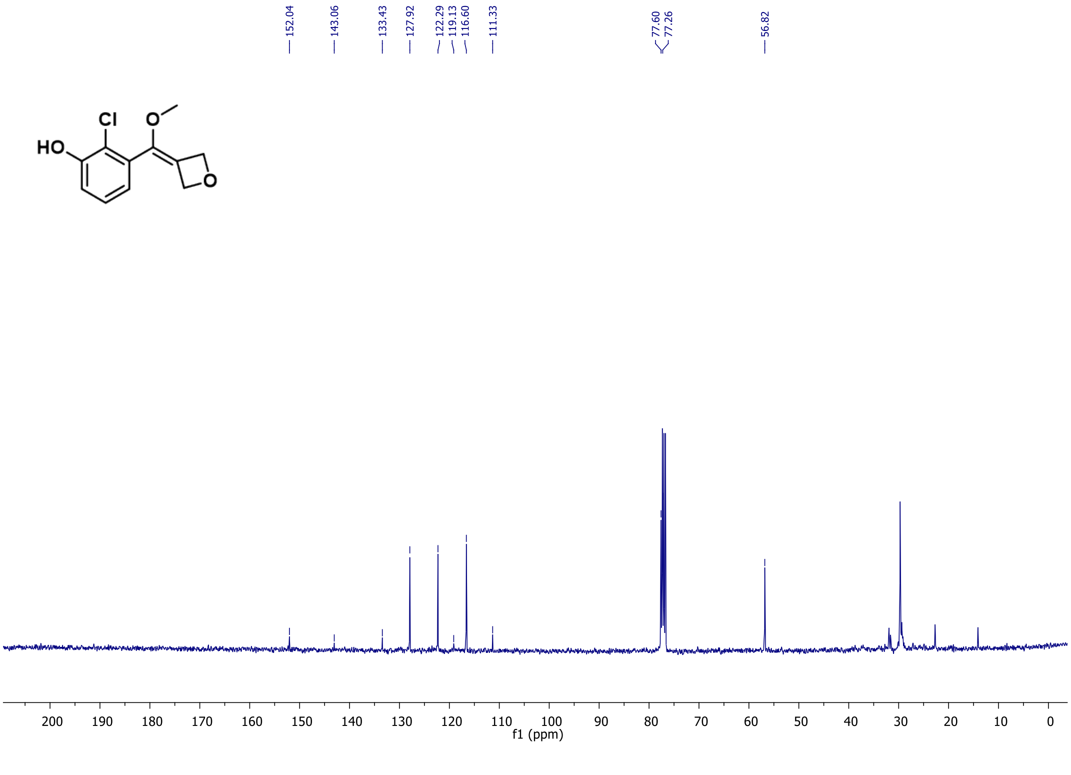

Mass spectra

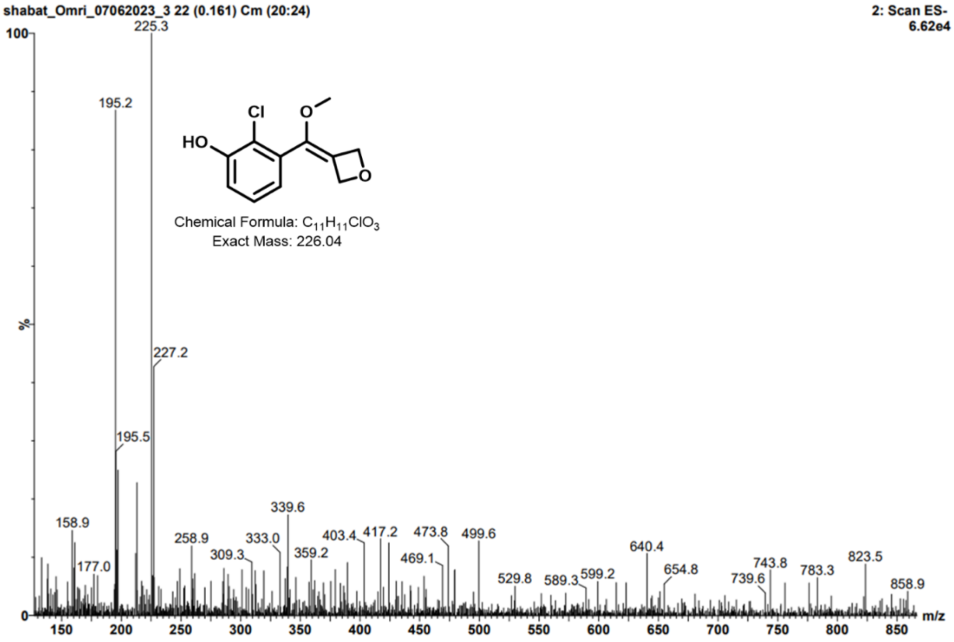

Compound 5e

^{1}H-NMR

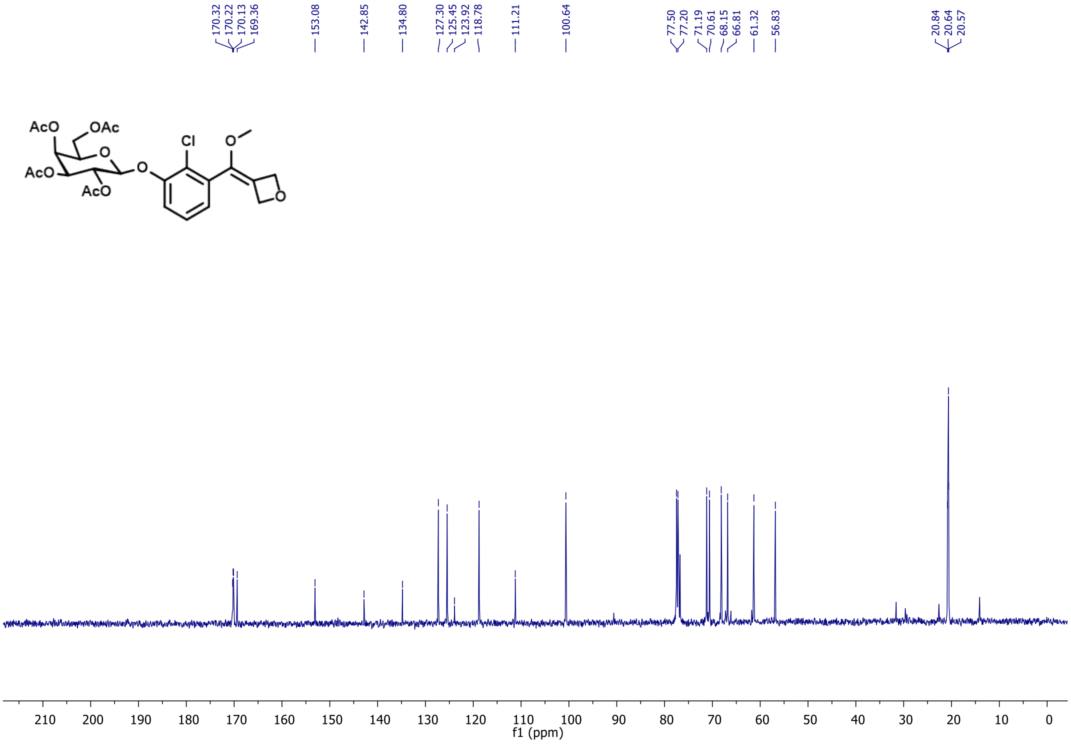

^{13}C-NMR

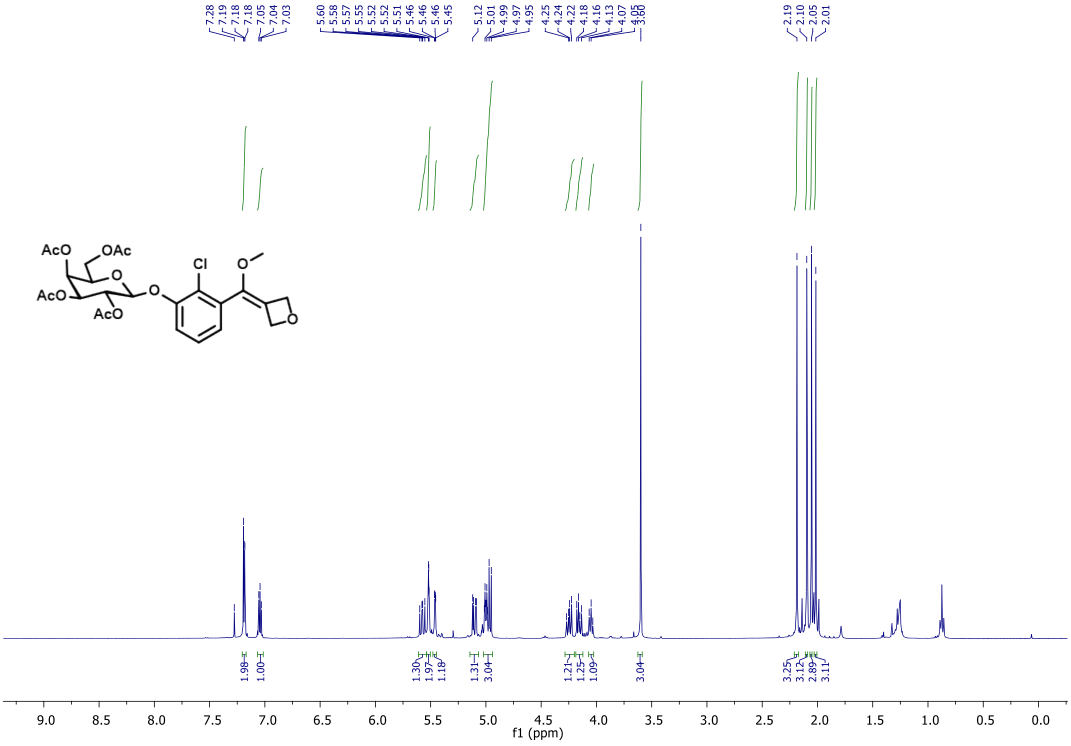

Mass spectra

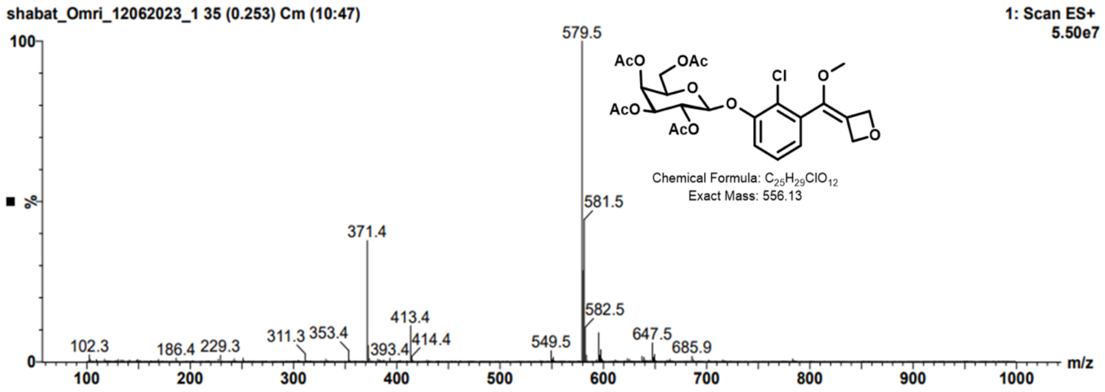

Probe β-gal N-Boc intermediate

Mass spectra

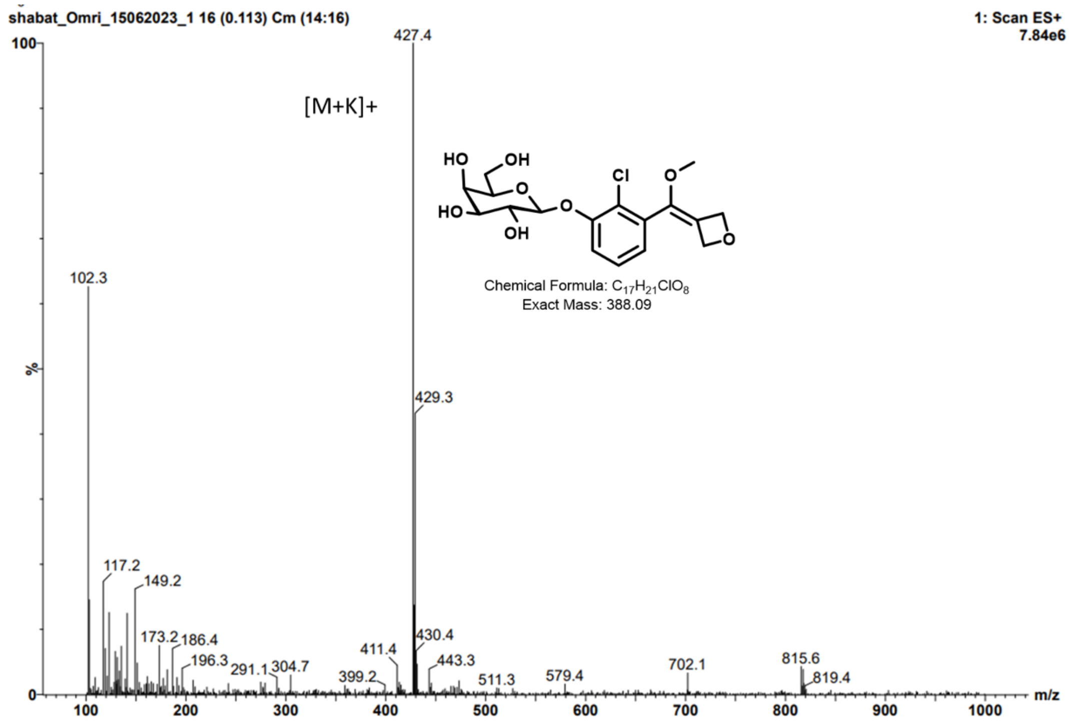

Probe β-gal N-Boc

^{1}H-NMR

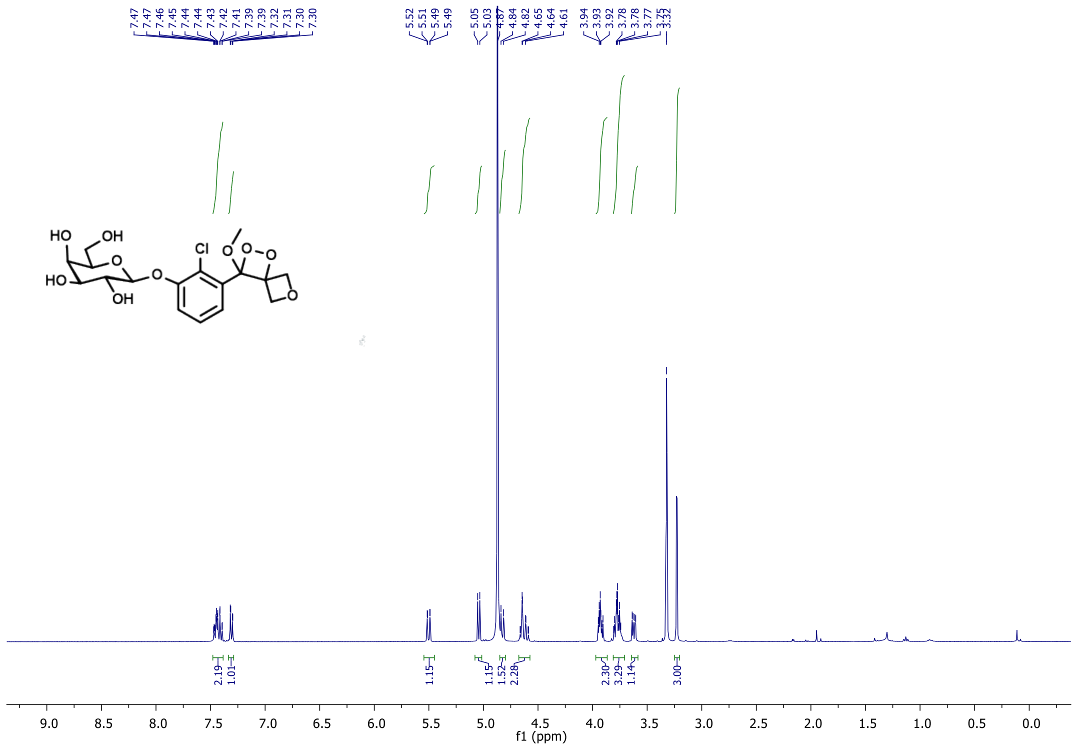

^{13}C-NMR

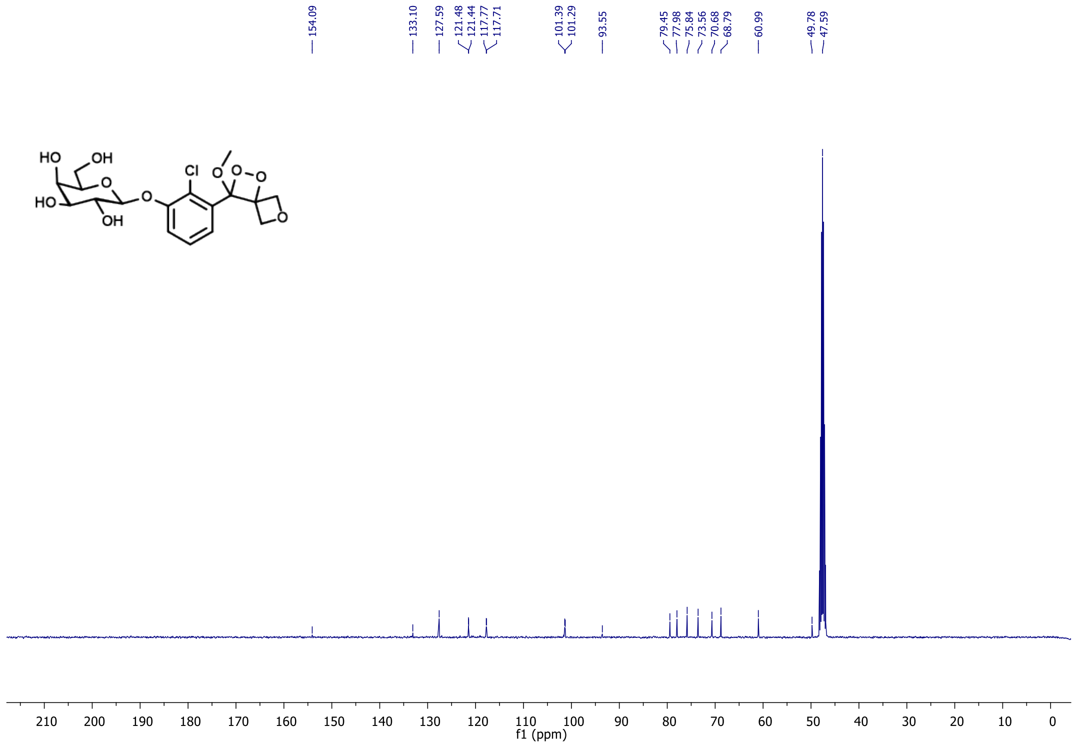

3D HPLC spectra (10-90% ACN in water, 0.1%TFA)

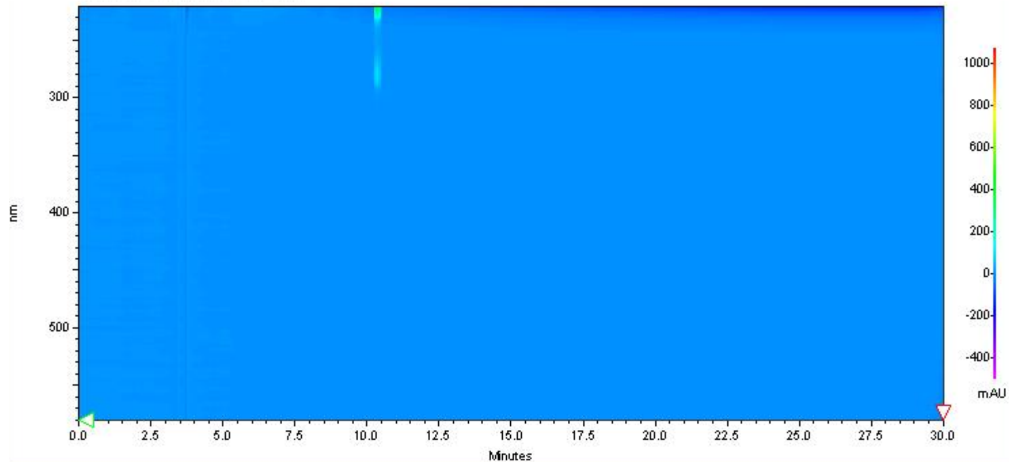

2D HPLC spectra (Absorbance measured at 277nm)

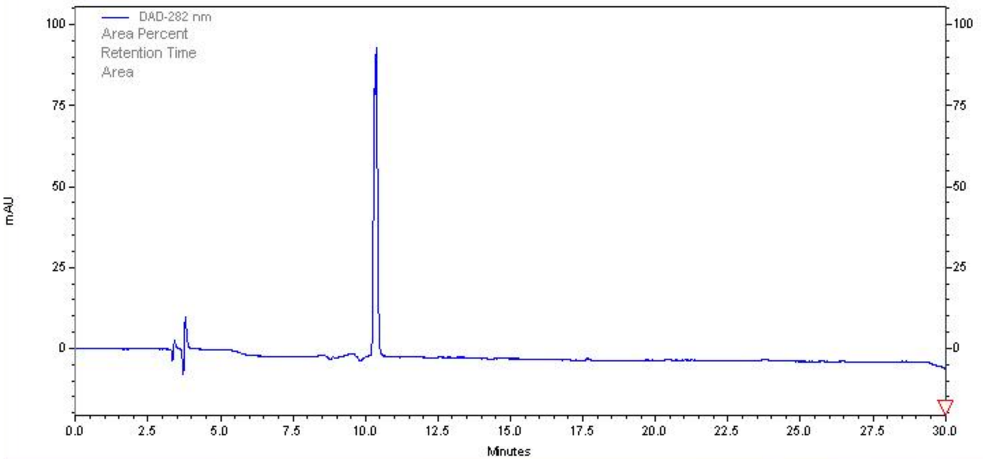

Mass spectra

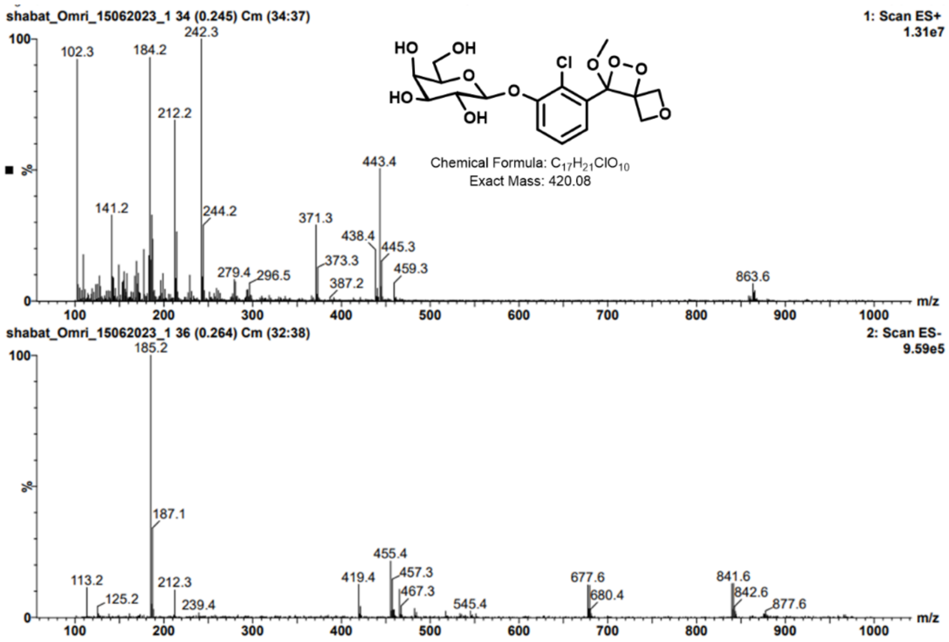

Compound 6d

^{1}H-NMR

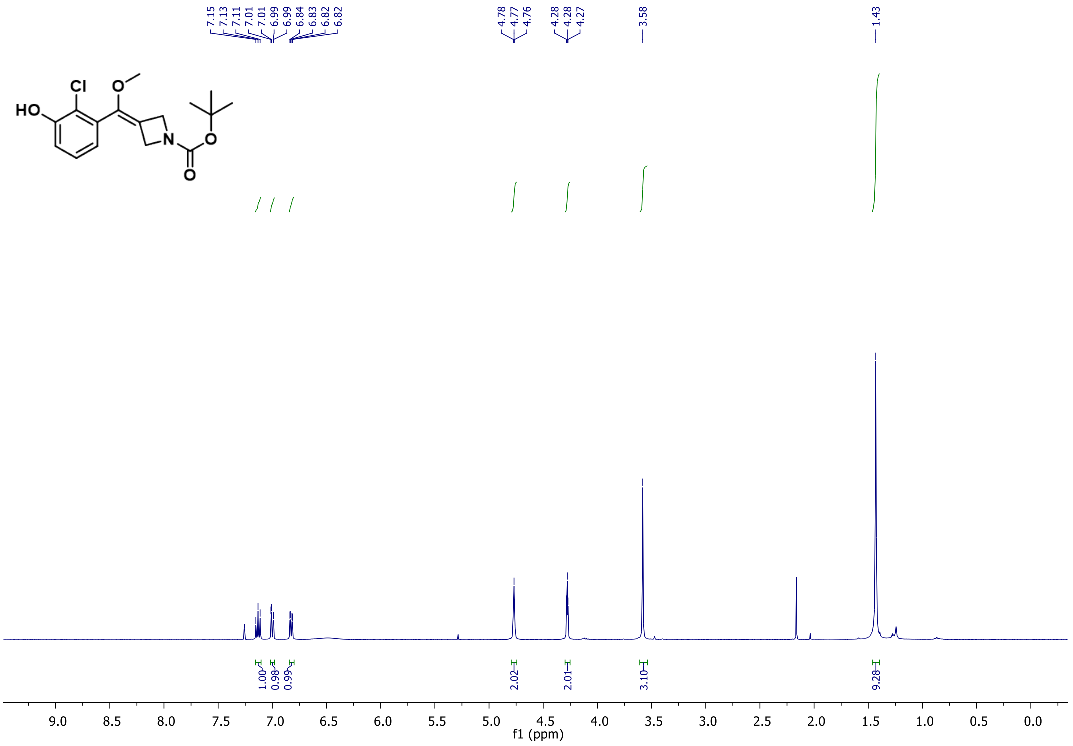

^{13}C-NMR

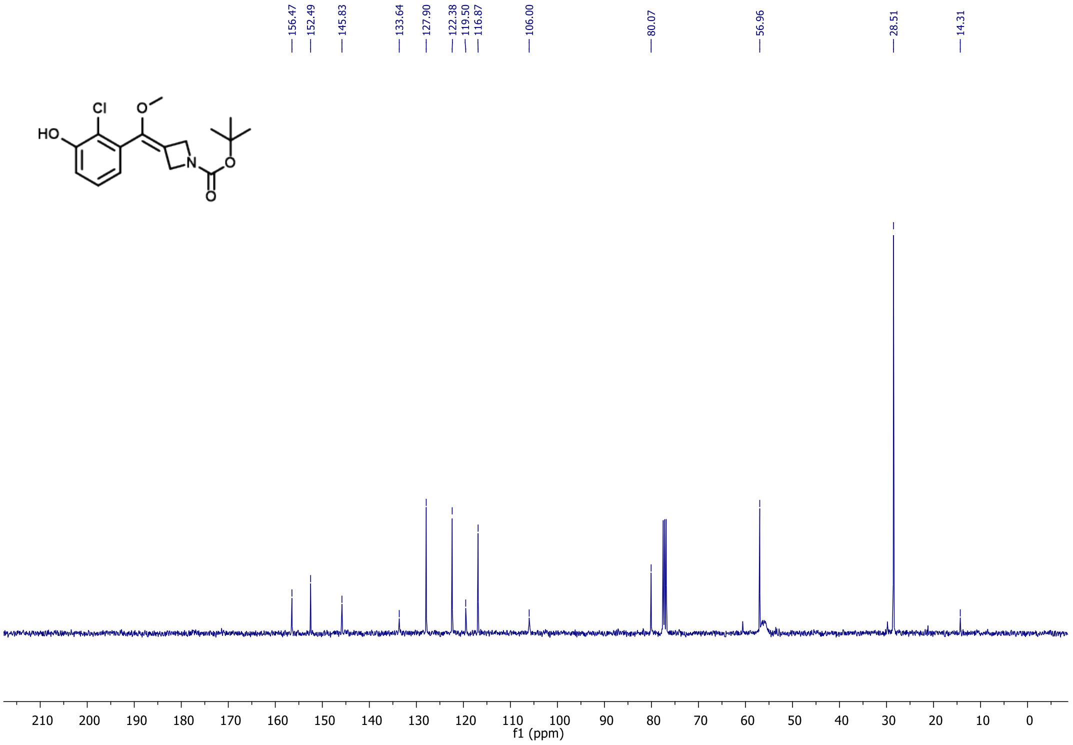

Mass spectra

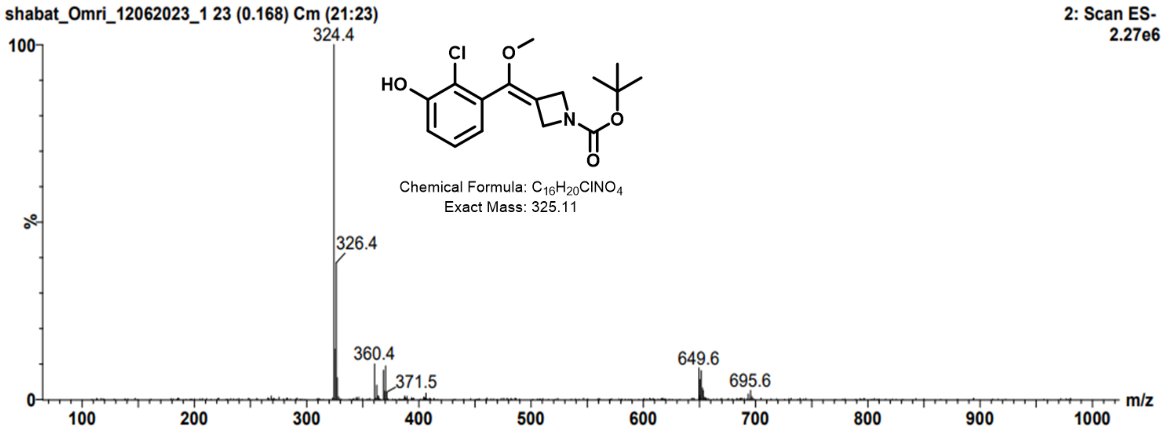

Compound 6e

^{1}H-NMR

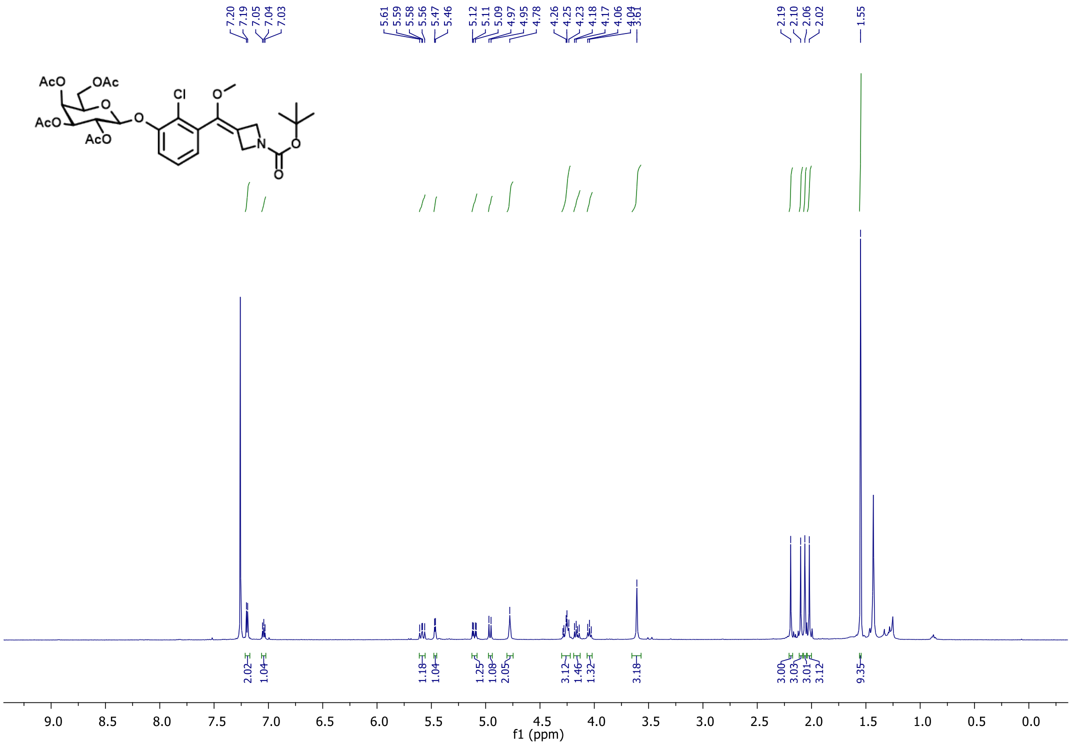

^{13}C-NMR

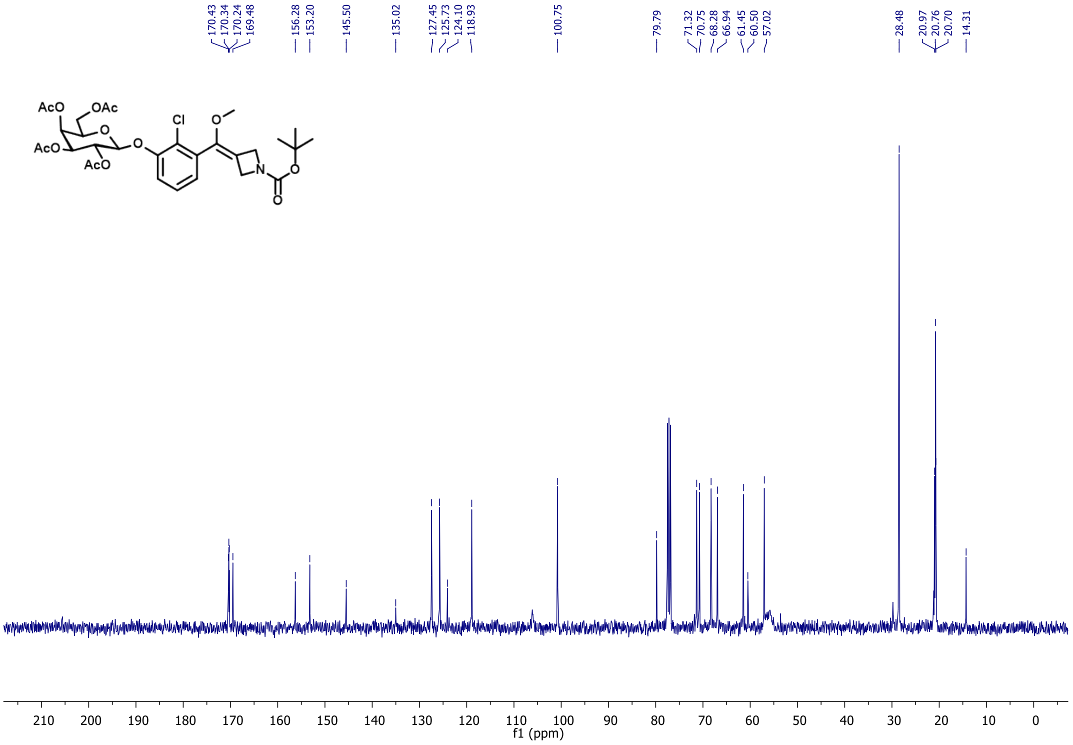

Mass spectra

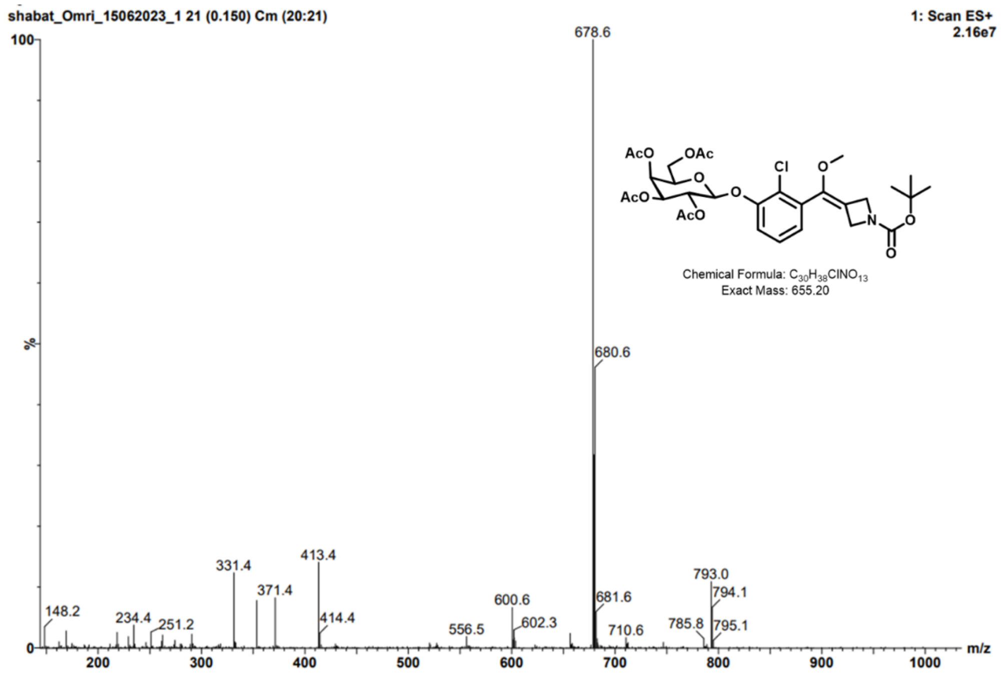

Probe β-gal N-Boc intermediate

Mass spectra

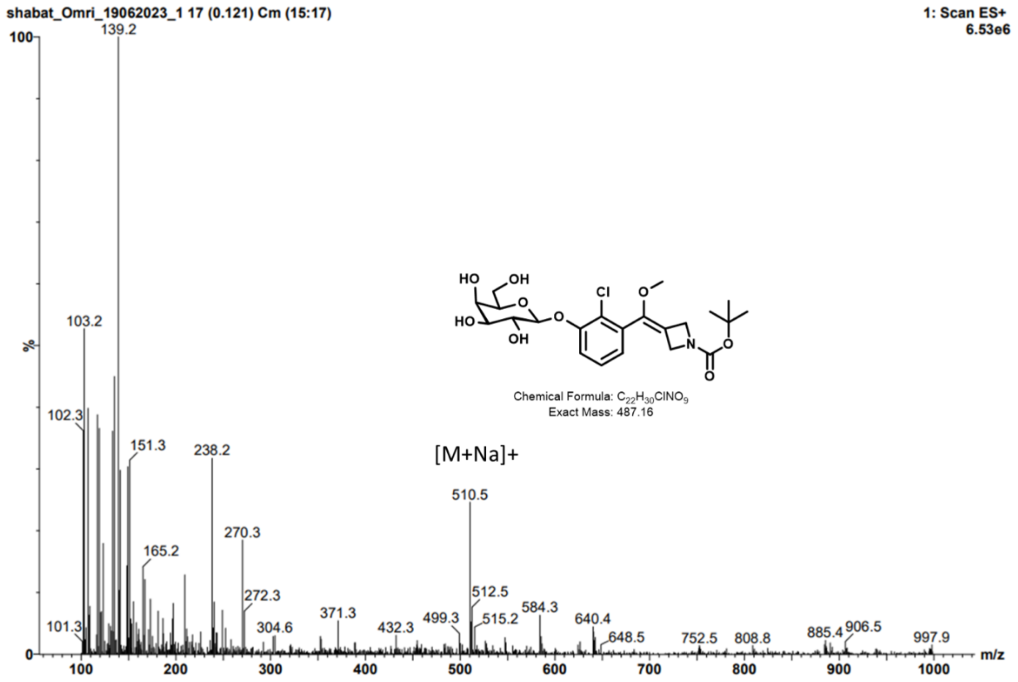

Probe β-gal N-Boc

^{1}H-NMR

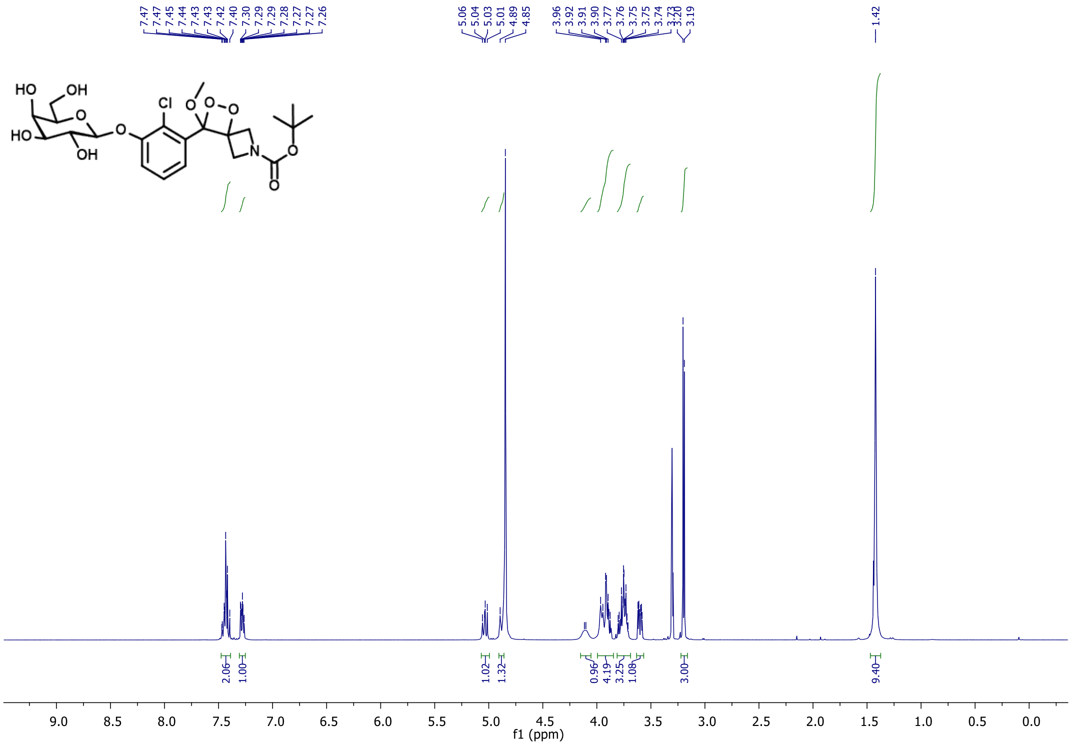

^{13}C-NMR

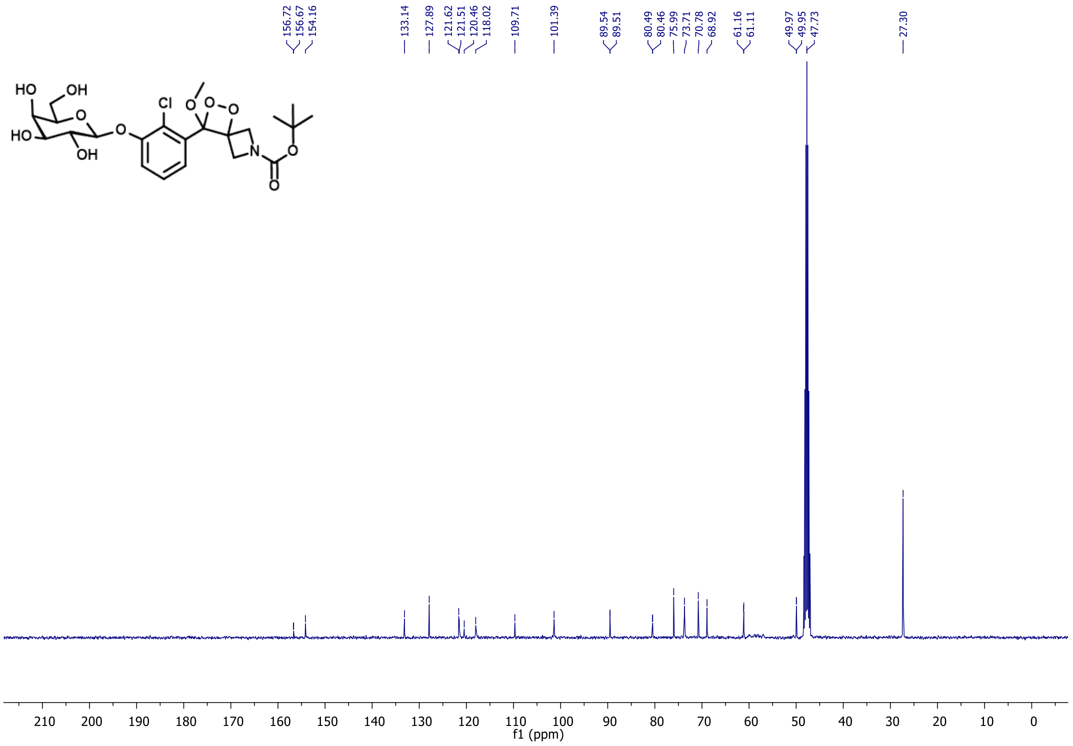

3D HPLC spectra (30-100% ACN in water, 0.1%TFA)

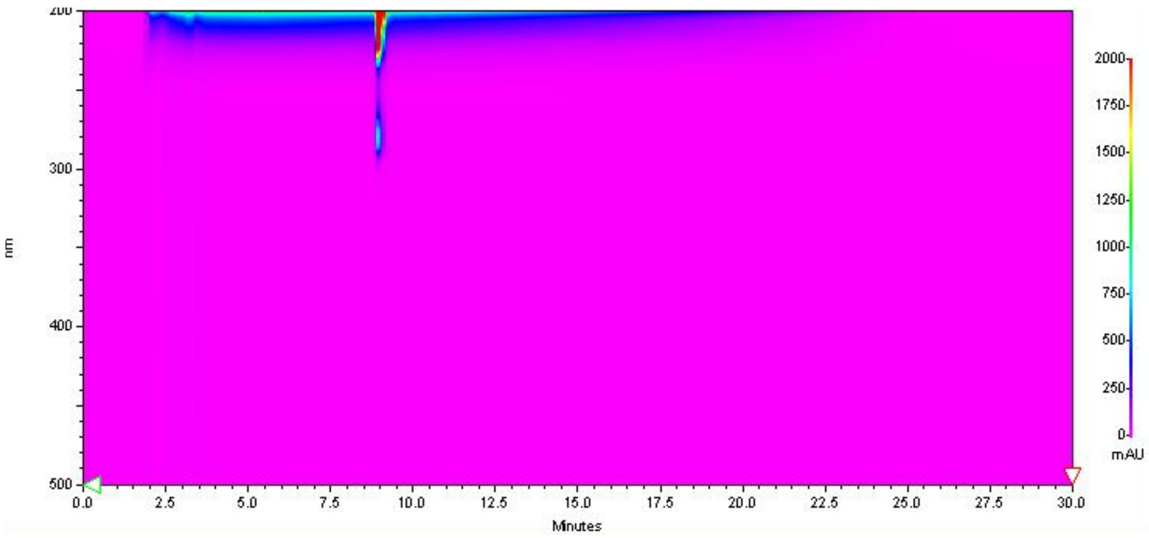

2D HPLC spectra (Absorbance measured at 277nm)

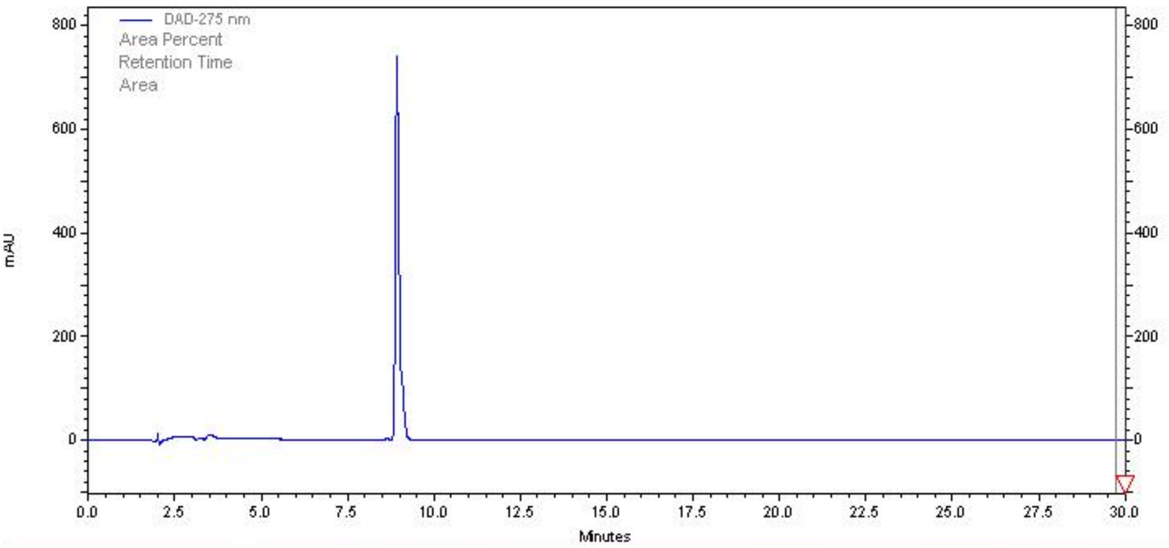

Mass spectra

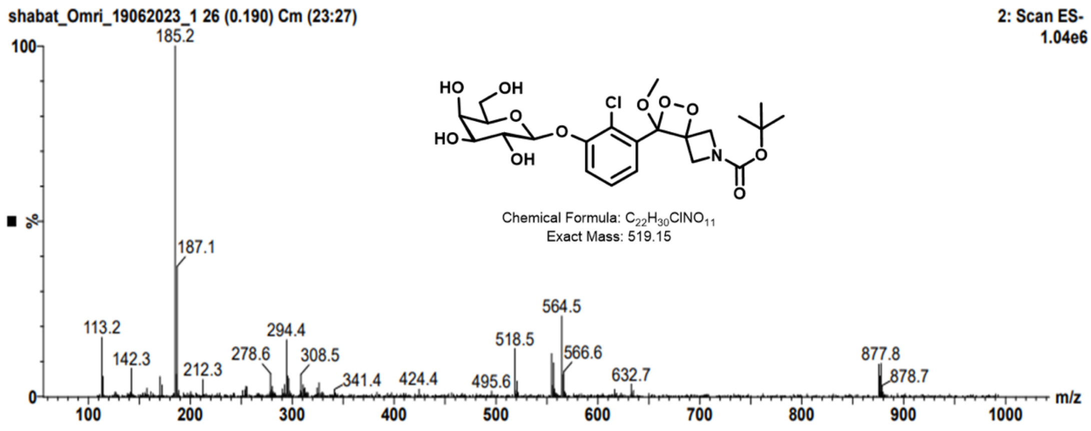

Compound 7d

^{1}H-NMR

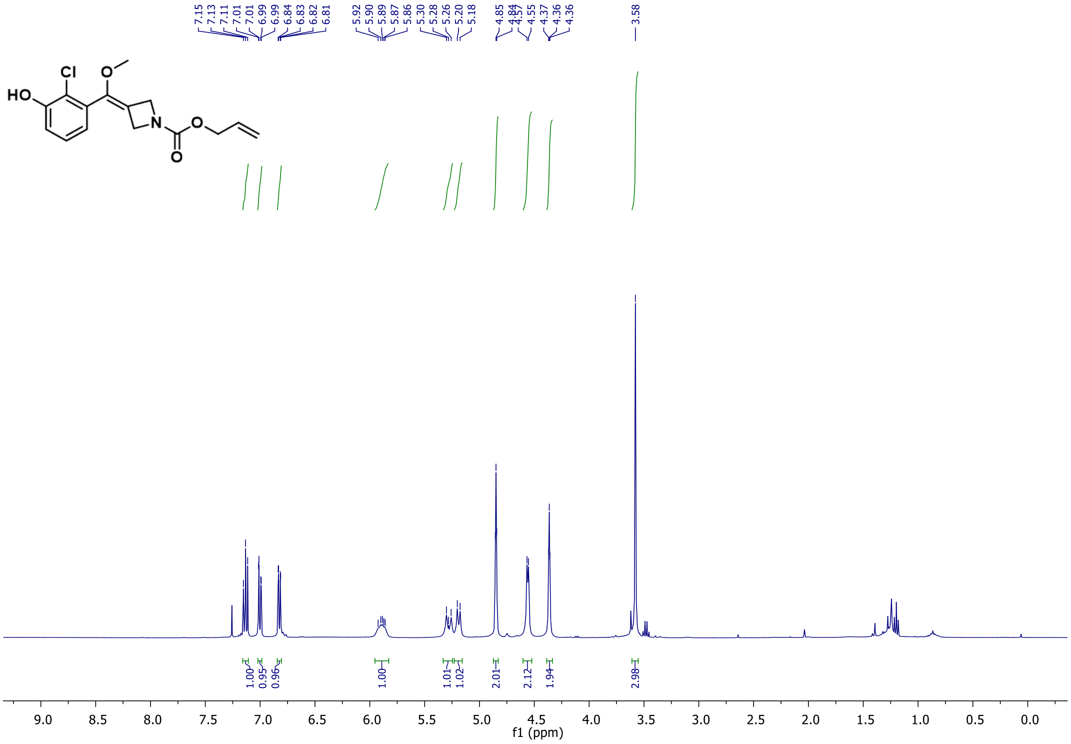

^{13}C-NMR

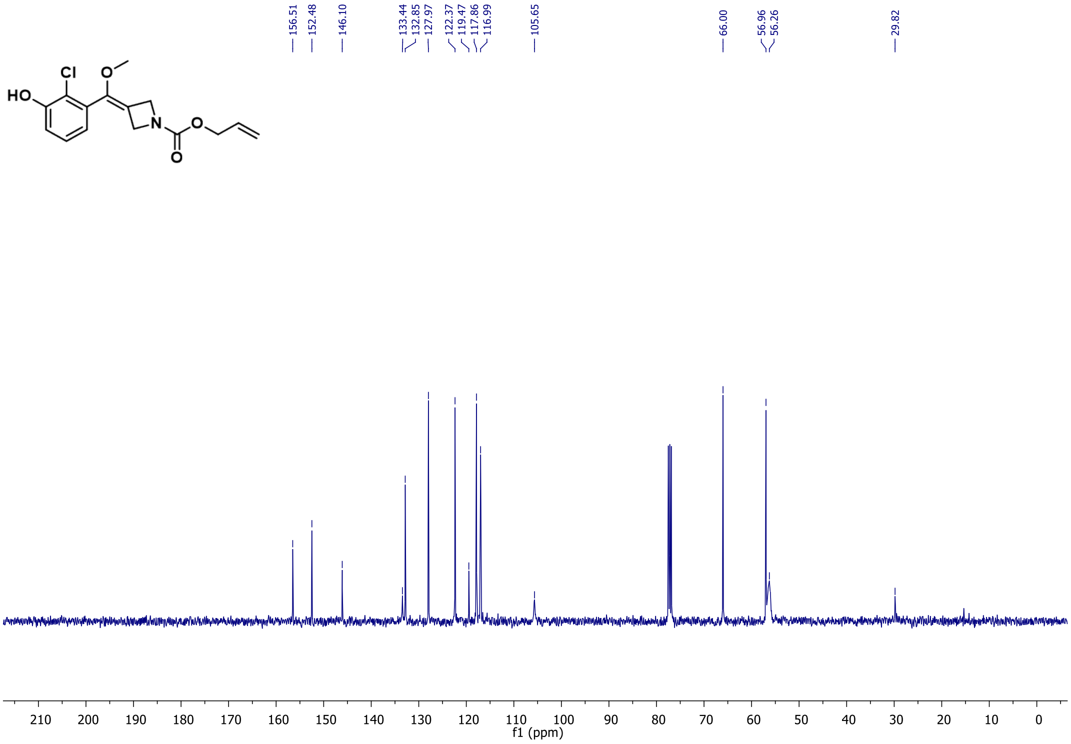

Mass spectra

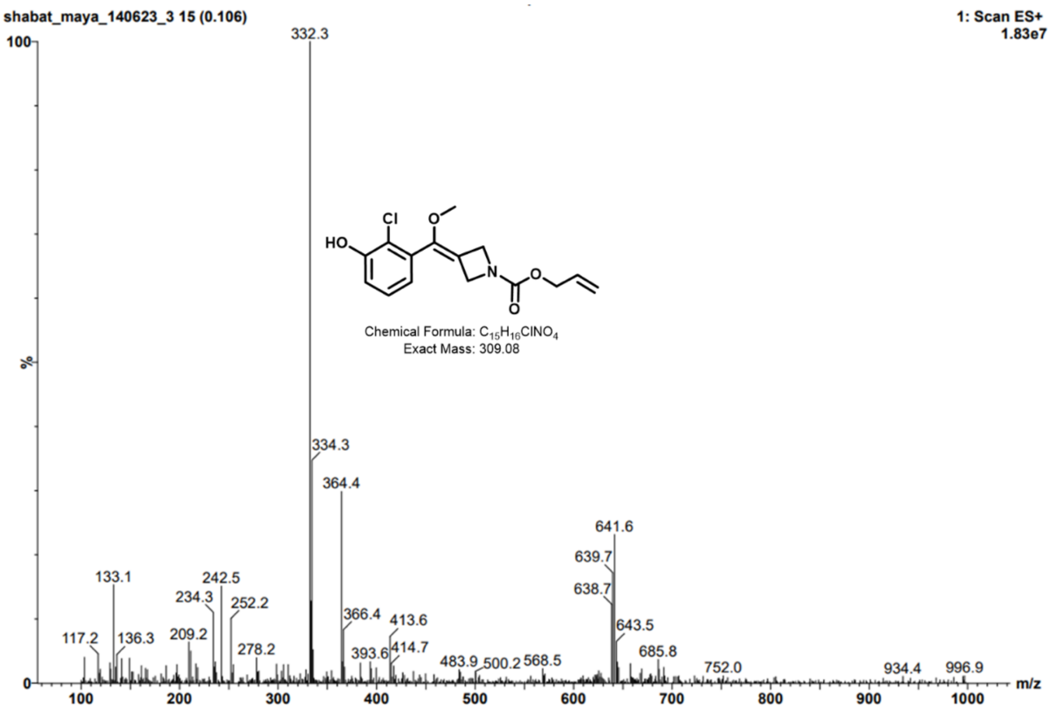

Compound 7e

^{1}H-NMR

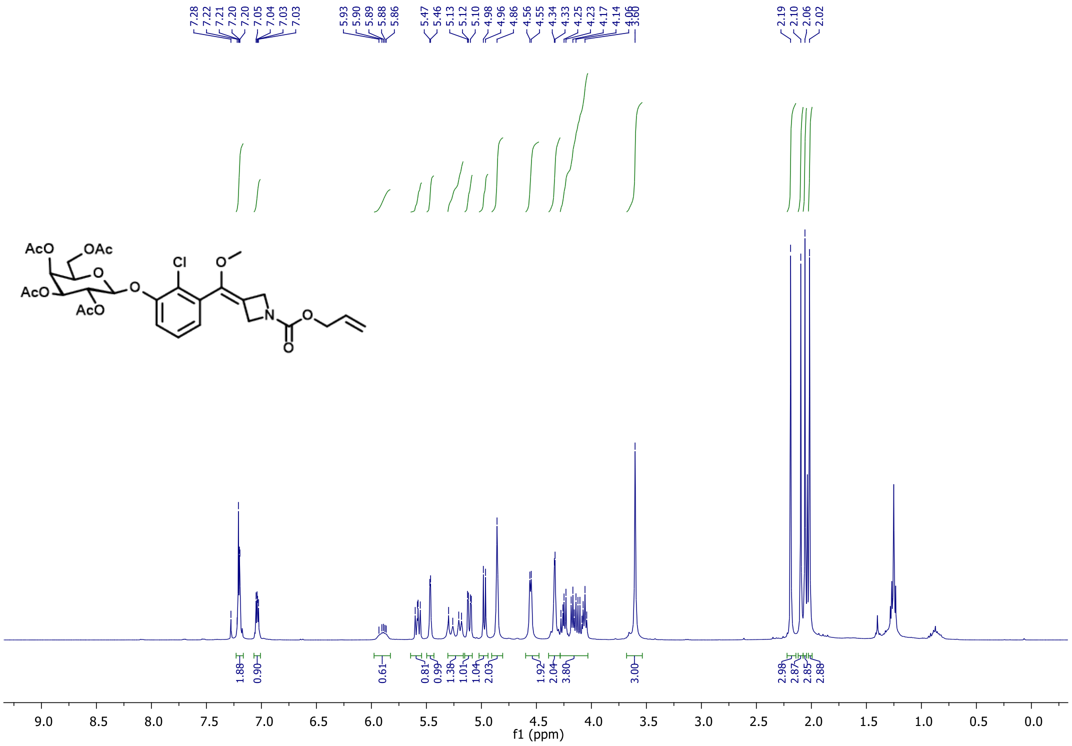

^{13}C-NMR

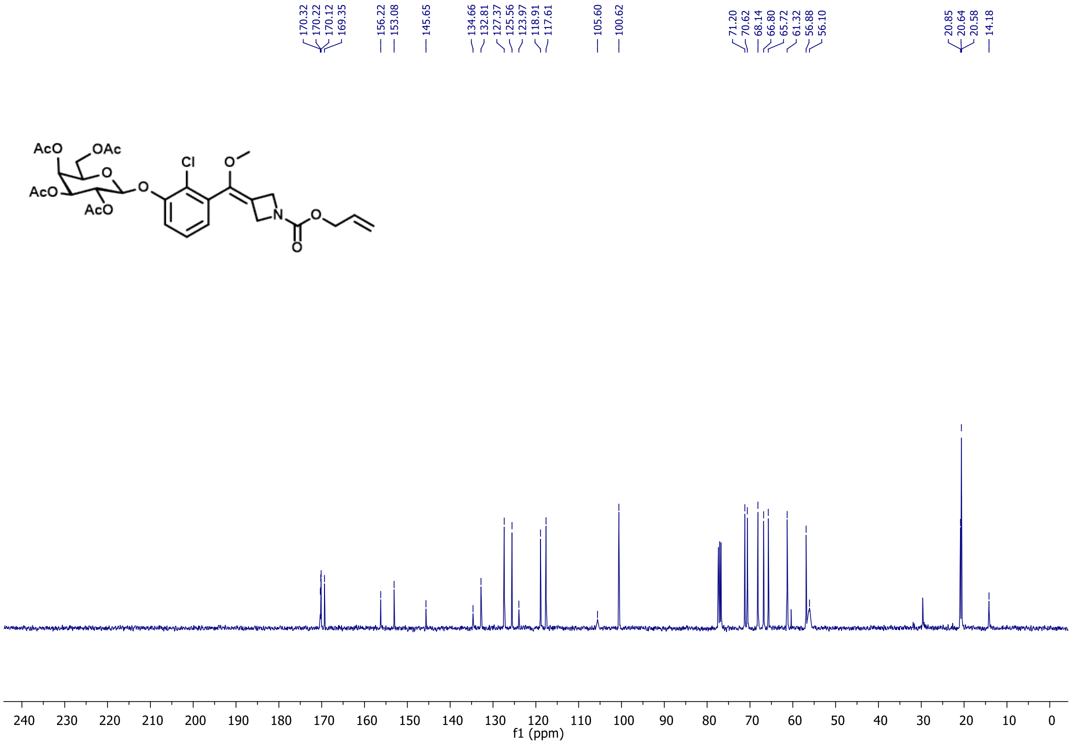

Mass spectra

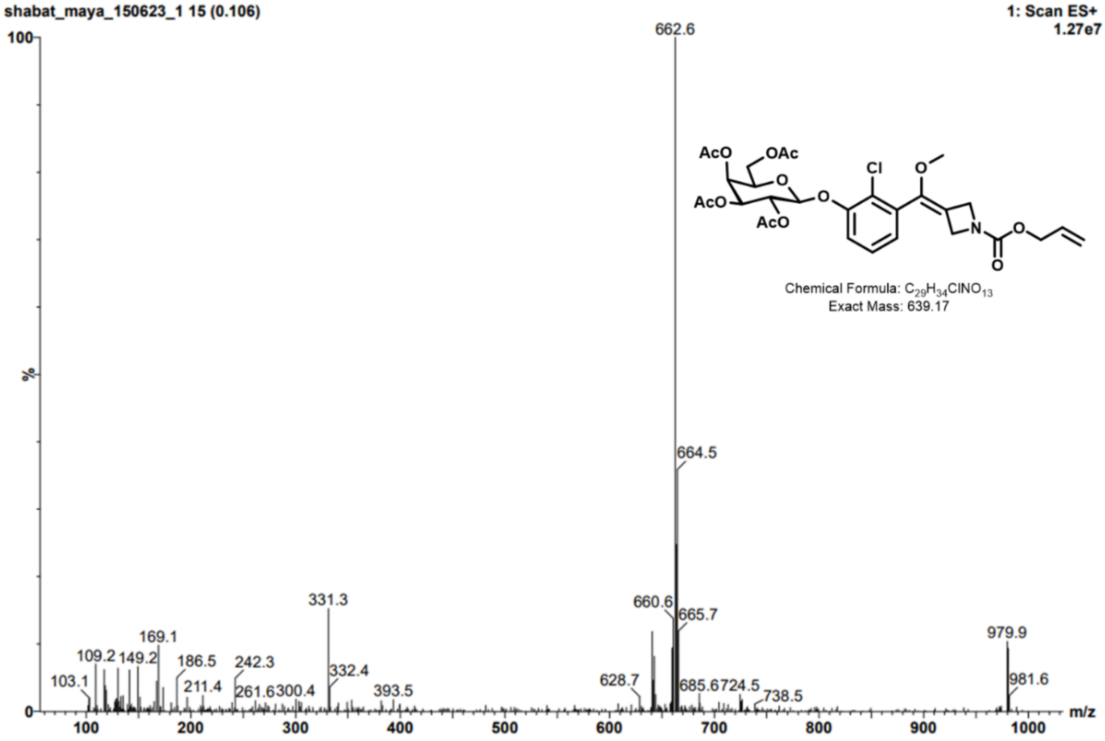

Compound 7f

^{1}H-NMR

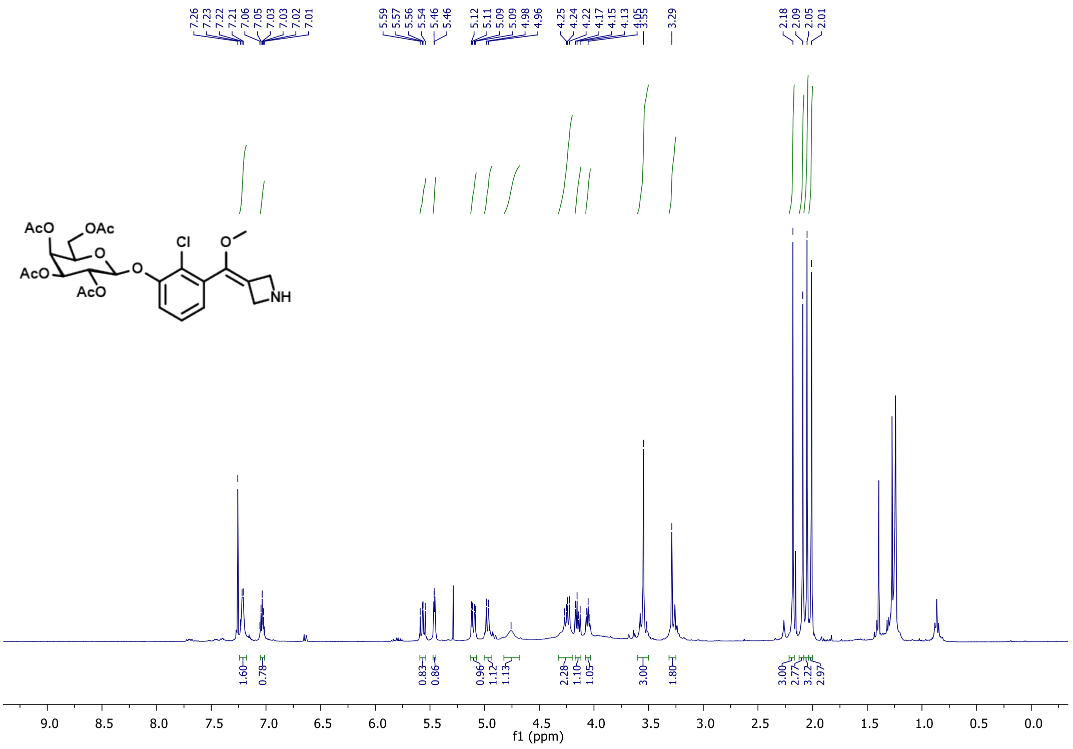

^{13}C-NMR

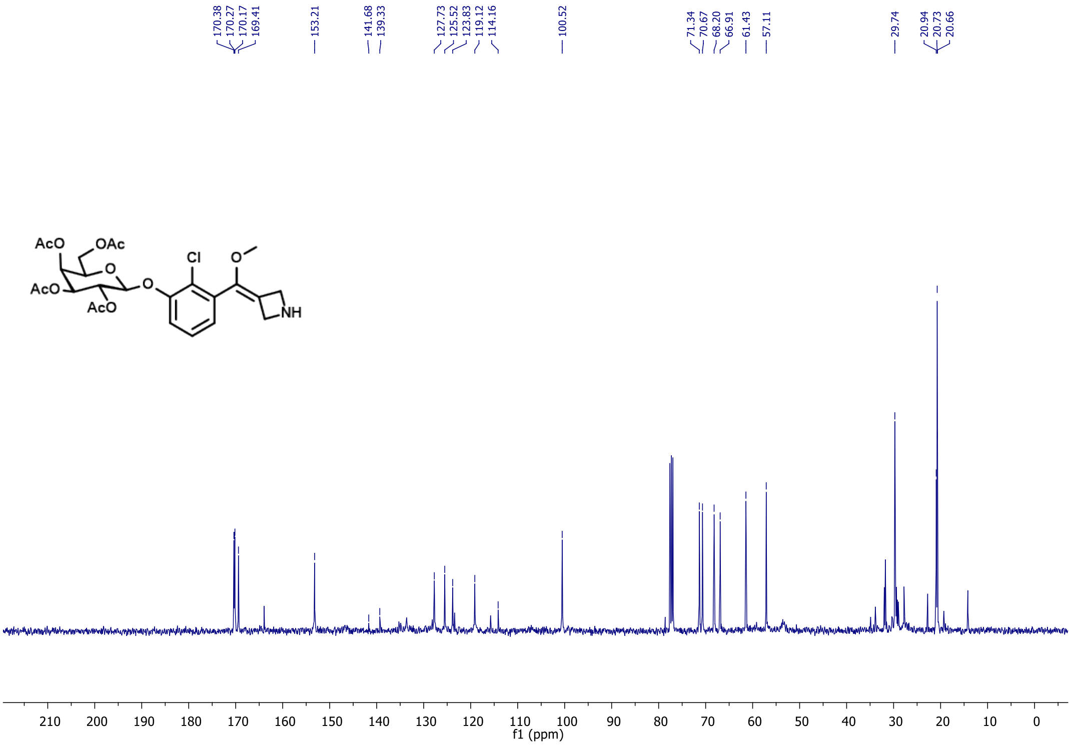

Mass spectra

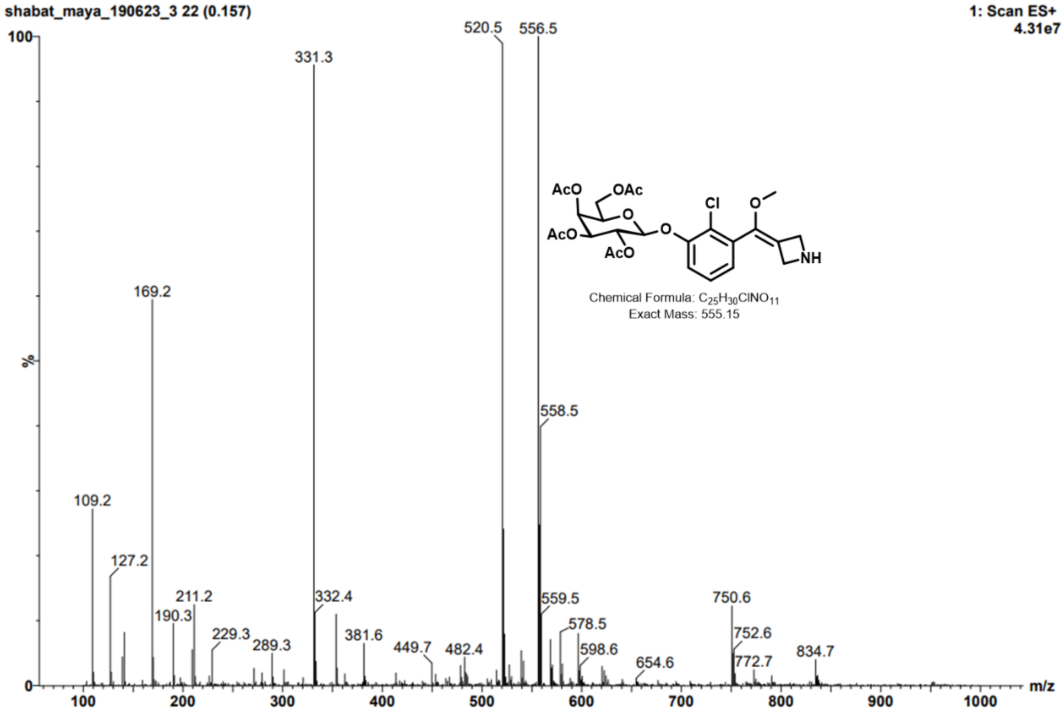

Compound 7g

^{1}H-NMR

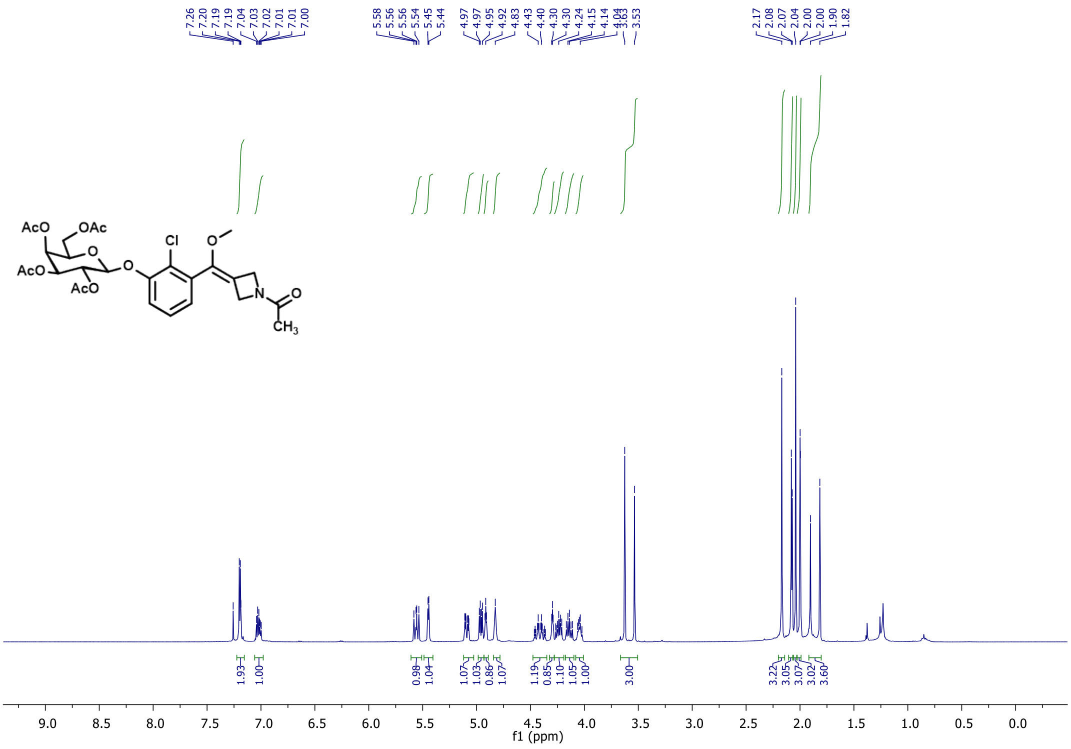

^{13}C-NMR

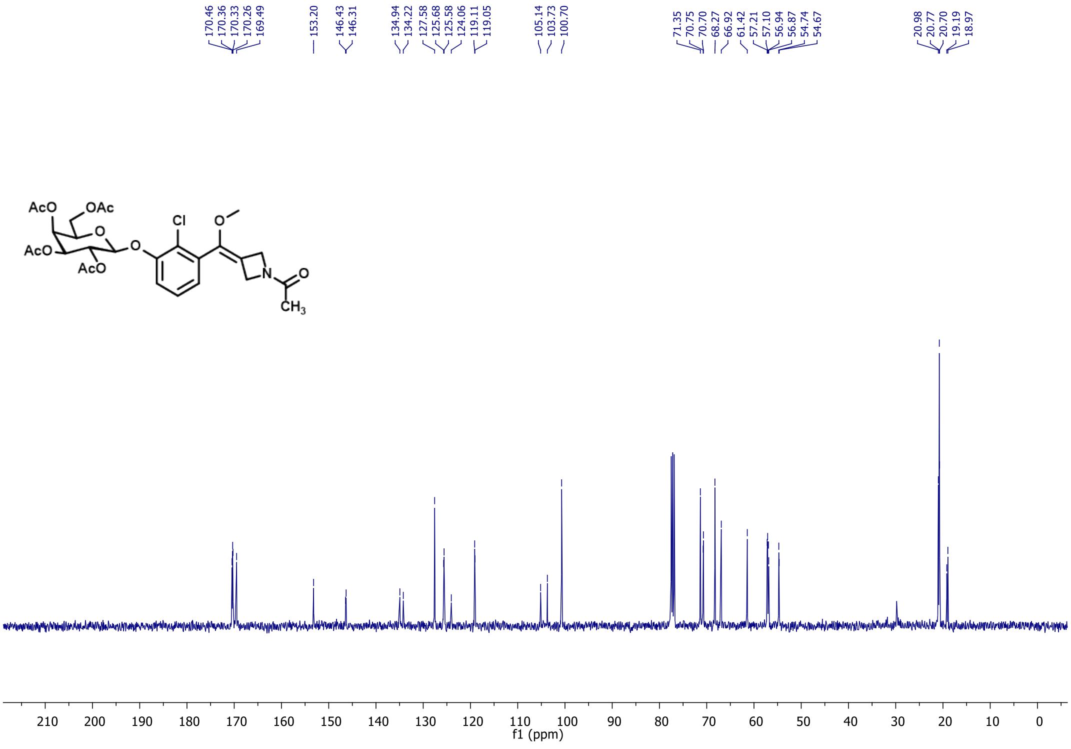

Mass spectra

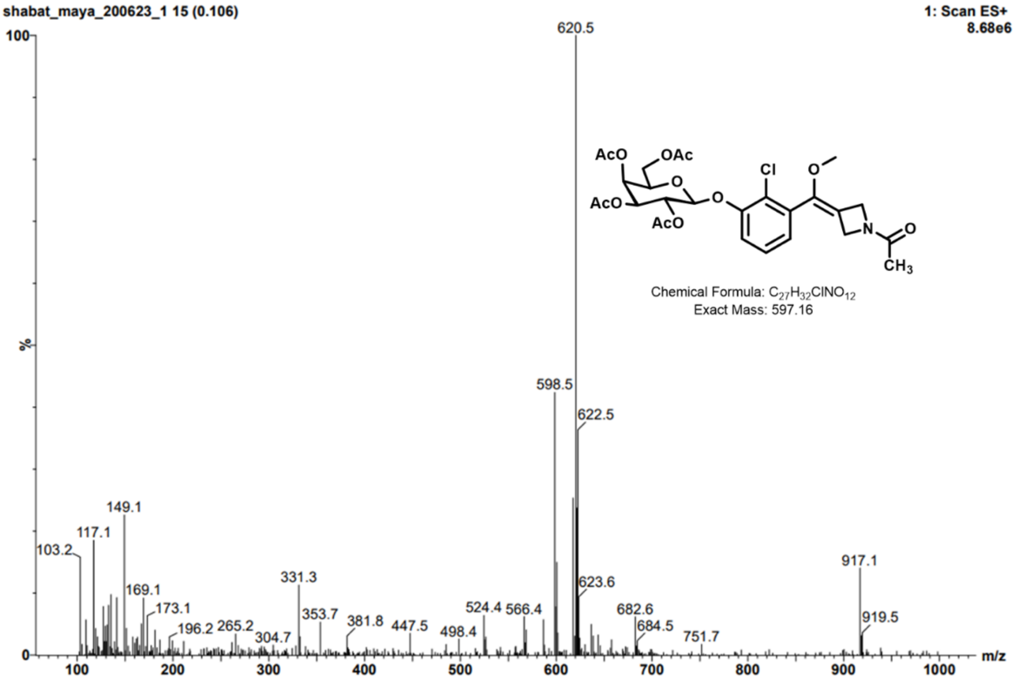

Probe β-gal N-Ac intermediate

Mass spectra

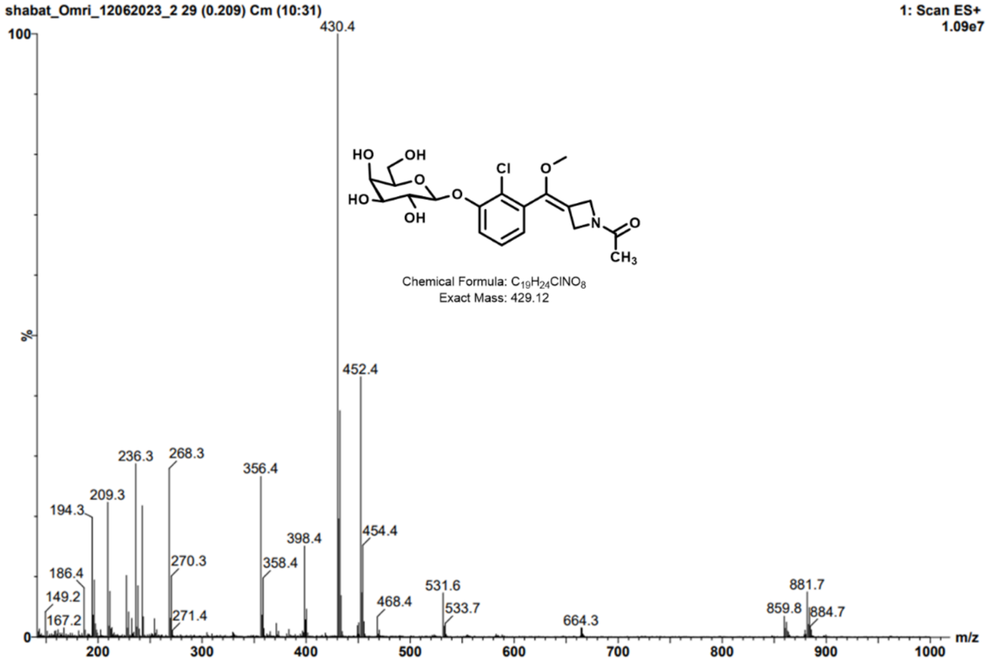

Probe β-gal N-Ac

^{1}H-NMR

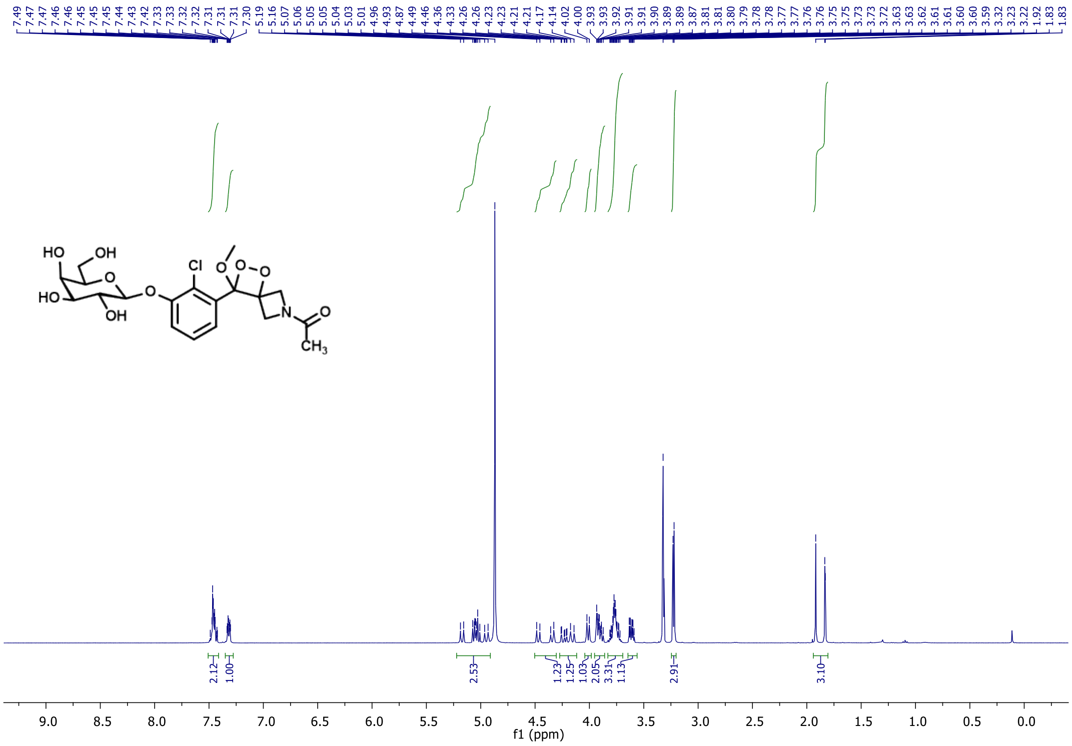

^{13}C-NMR

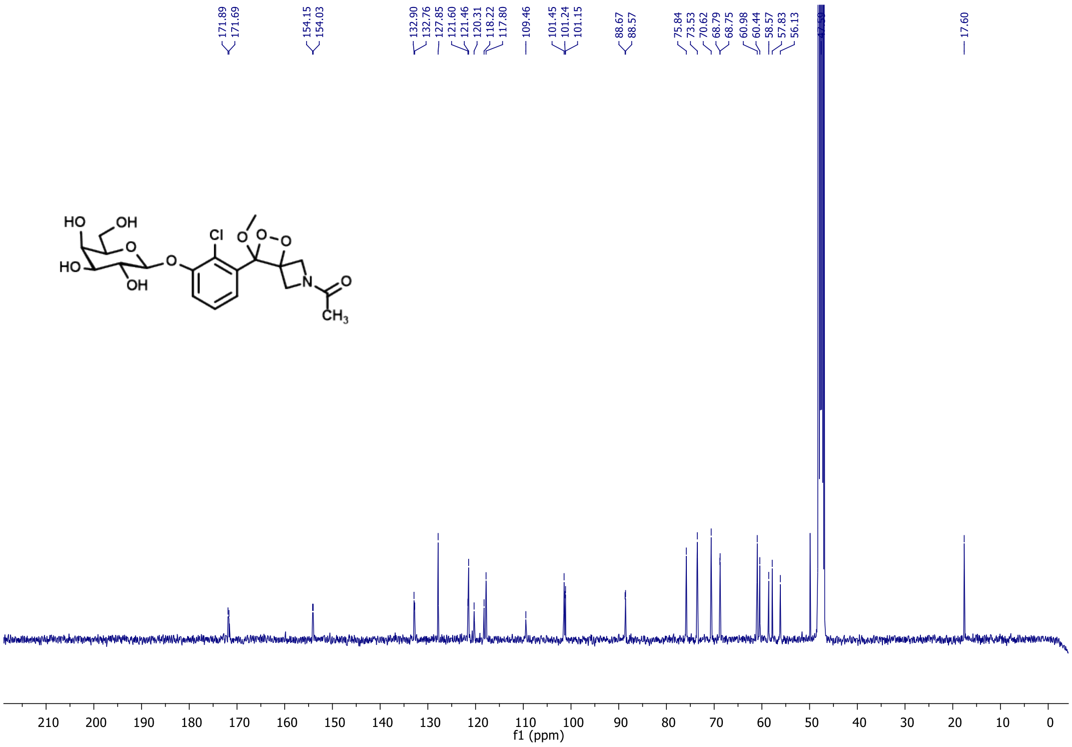

3D HPLC spectra (30-100% ACN in water, 0.1%TFA)

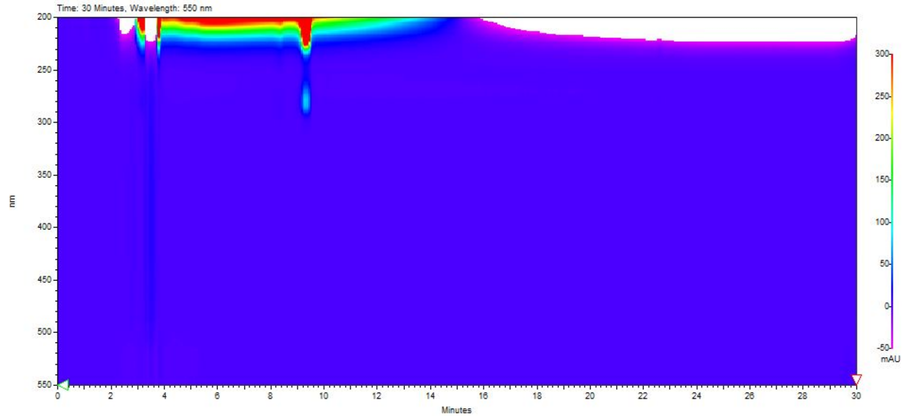

2D HPLC spectra (Absorbance measured at 277nm)

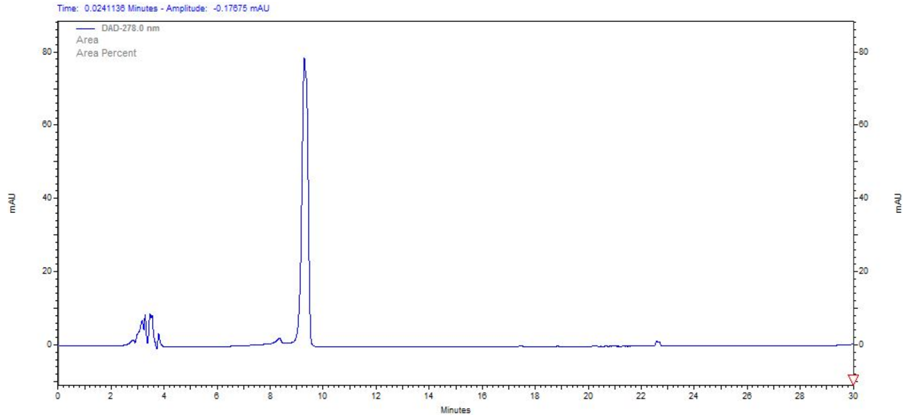

Mass spectra

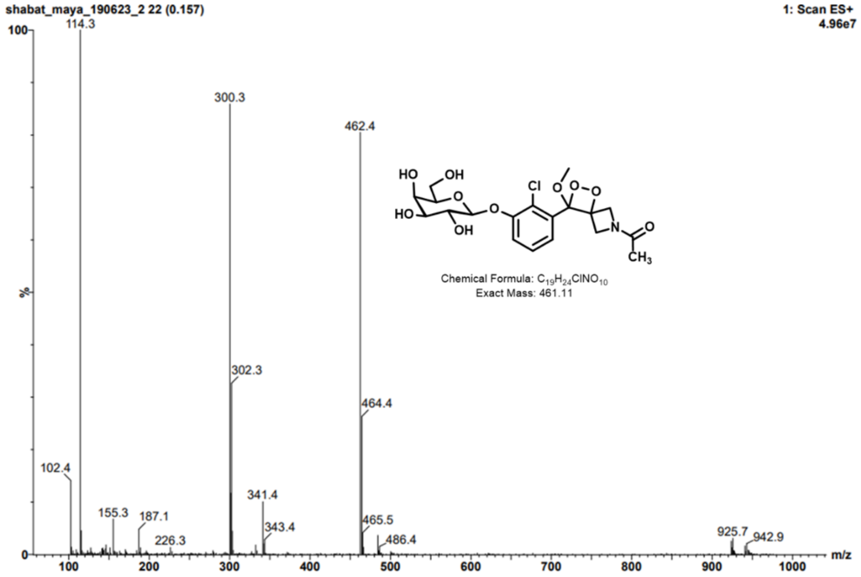

β-gal-masked-acrylate 1,2-dioxetane

Compound 1h

^{1}H-NMR

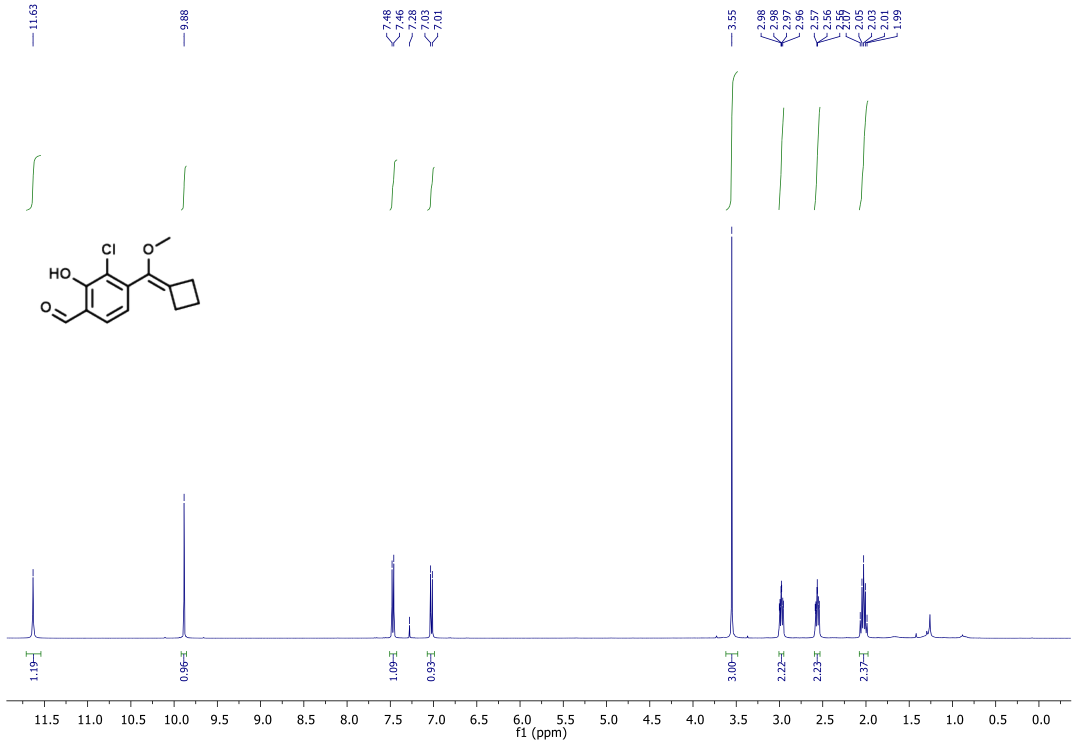

^{13}C-NMR

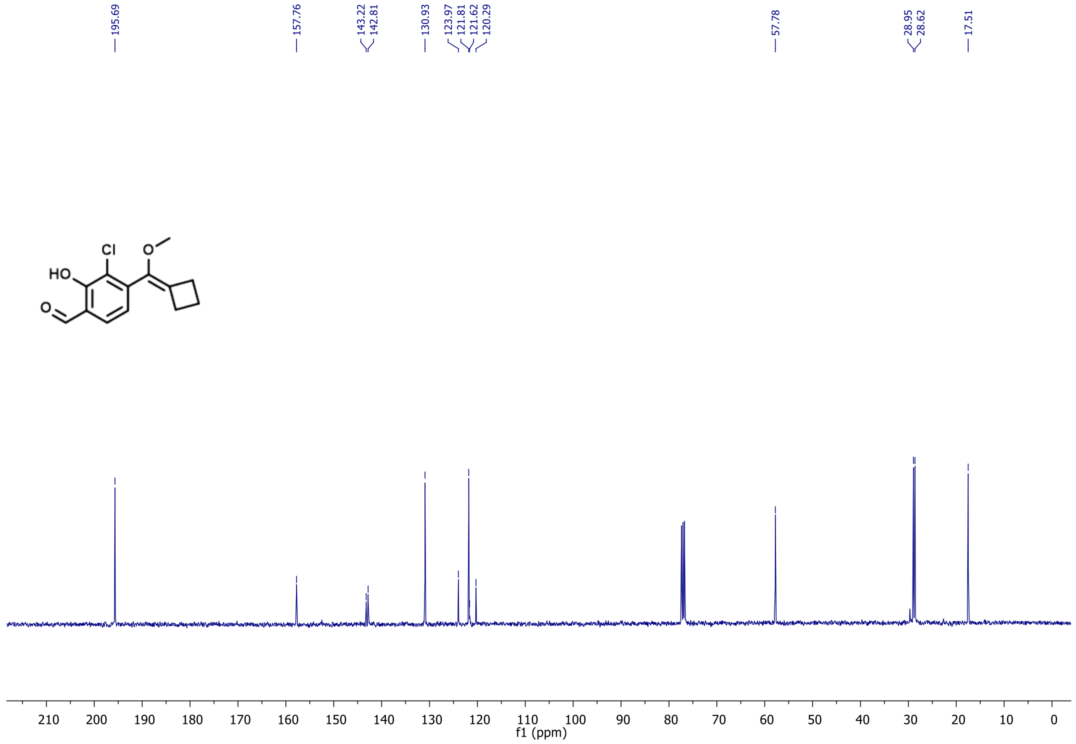

Mass spectra

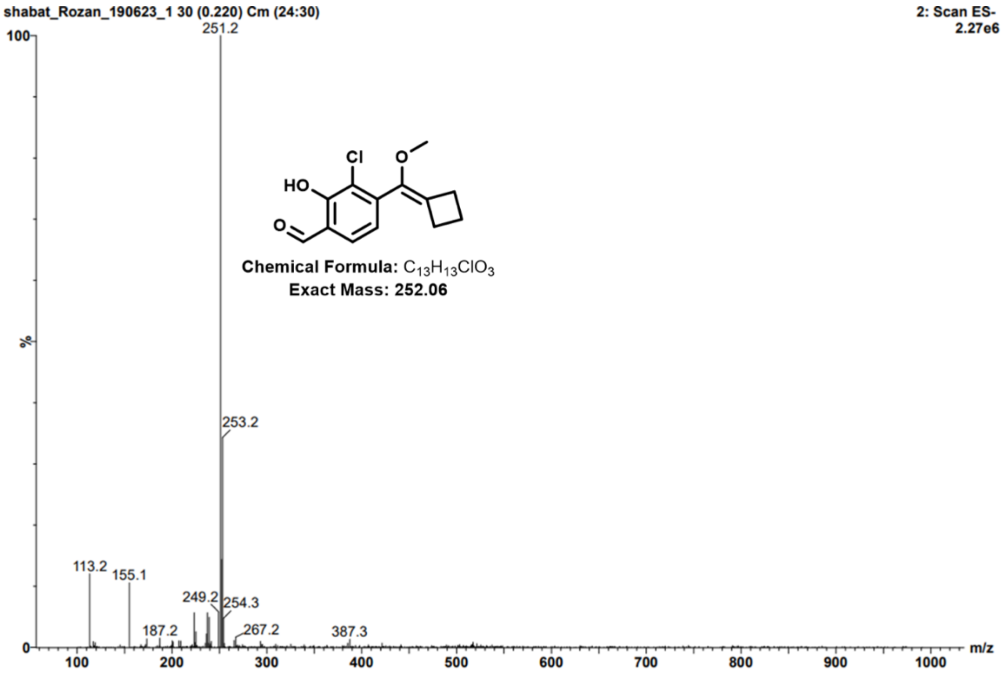

Compound 1i

^{1}H-NMR

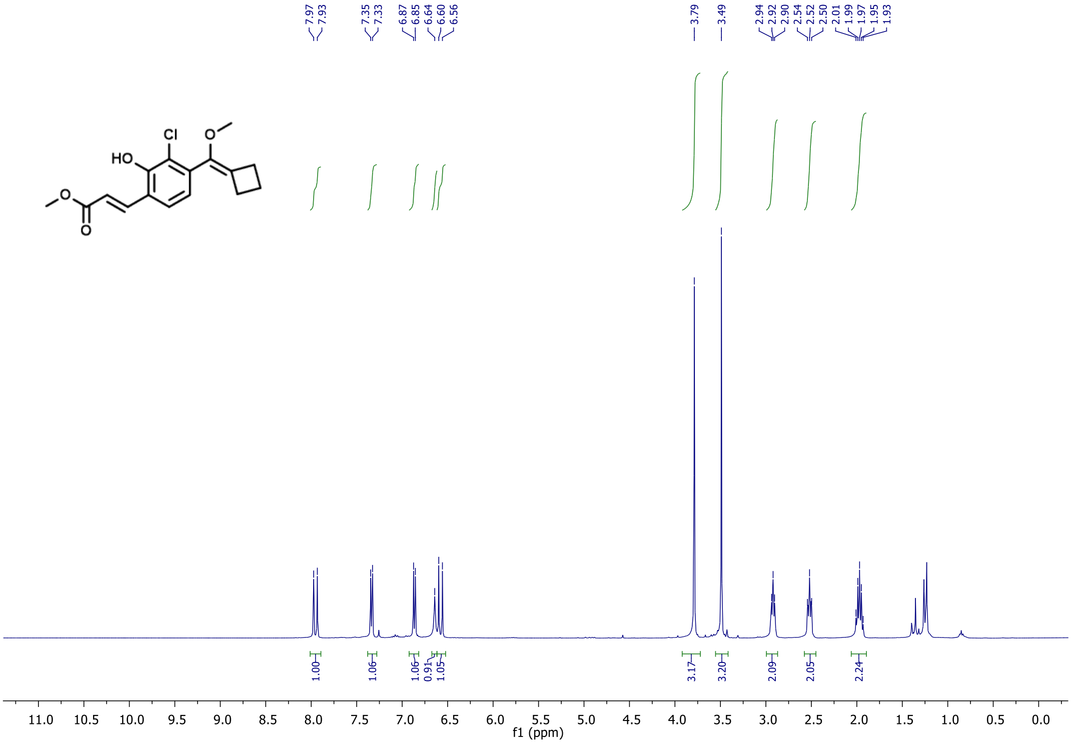

^{13}C-NMR

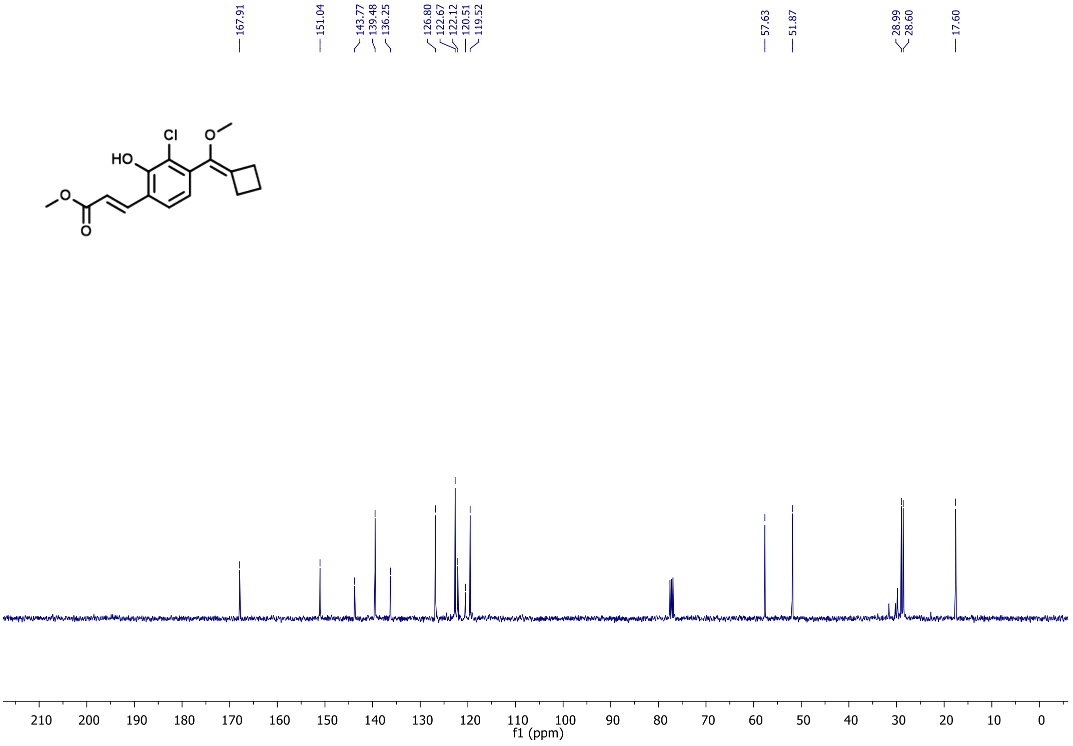

3D HPLC spectra (50-100% ACN in water, 0.1%TFA)

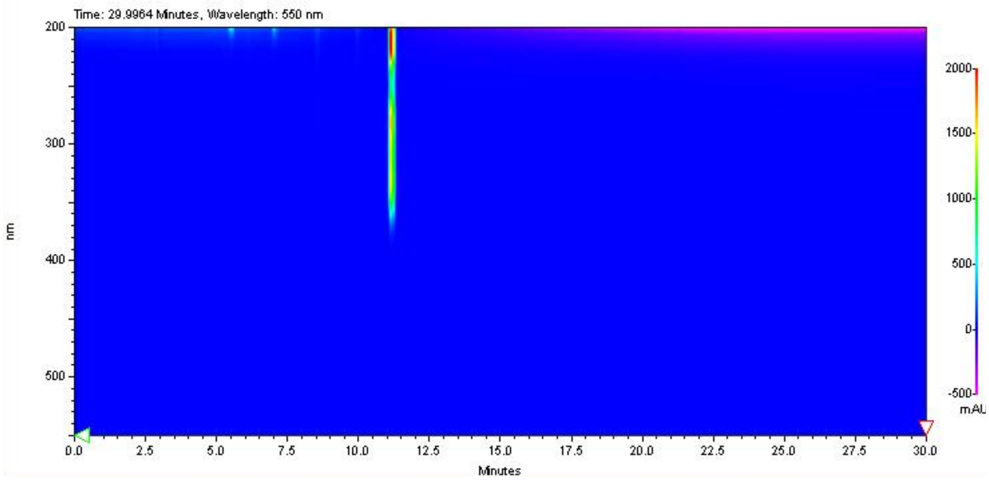

2D HPLC spectra (Absorbance measured at 325nm)

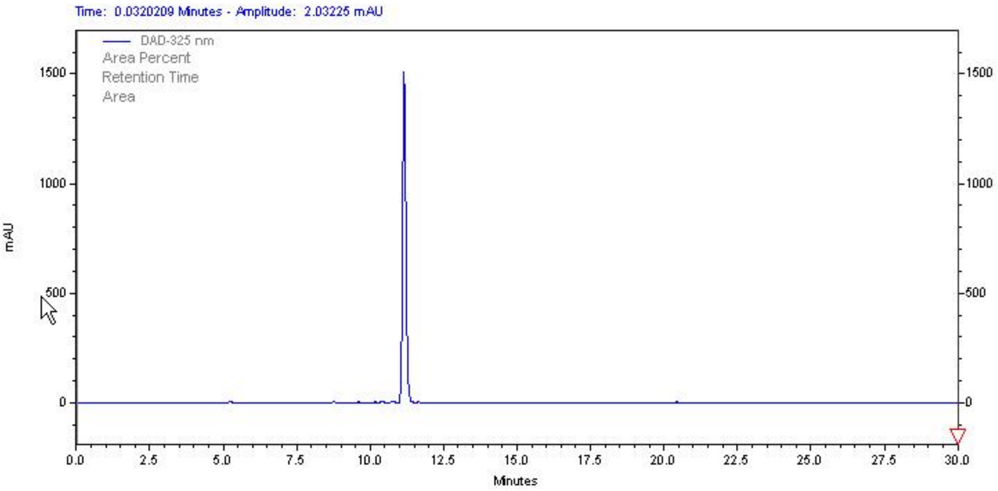

Mass spectra

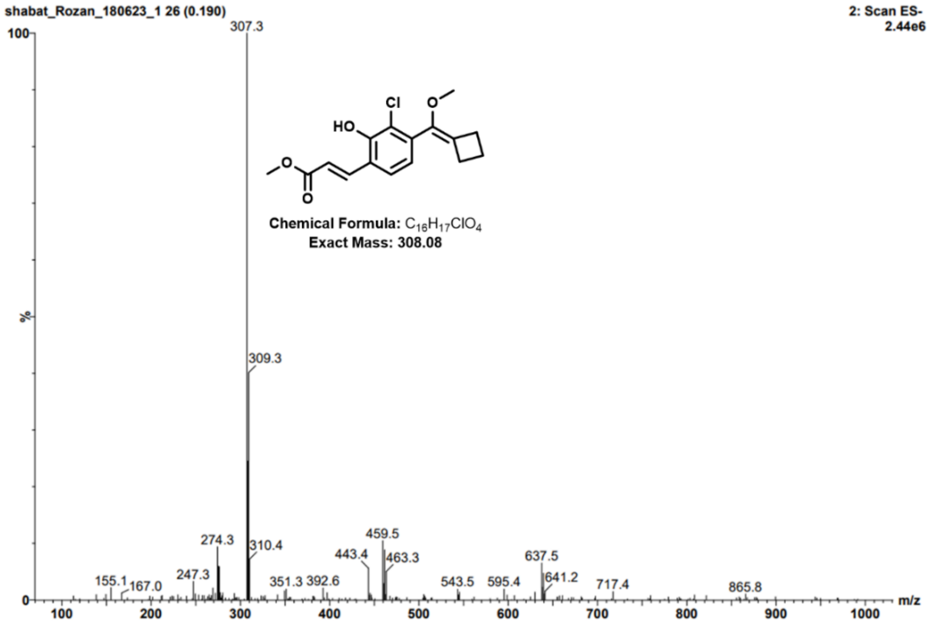

Compound 1j

^{1}H-NMR

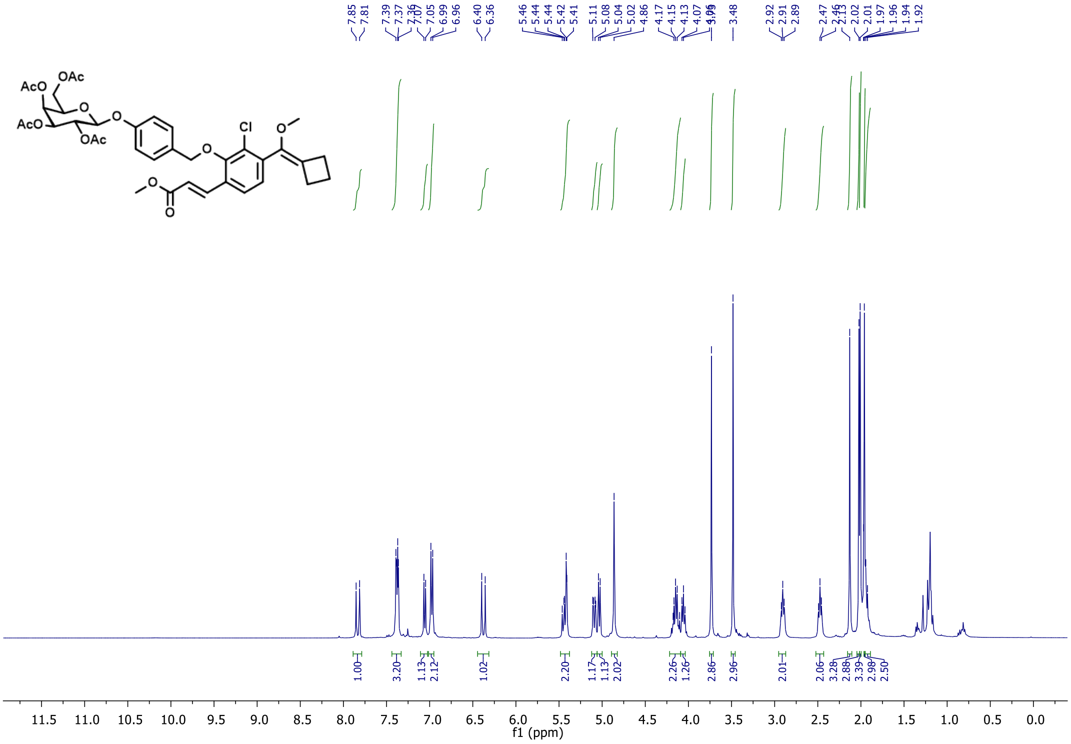

^{13}C-NMR

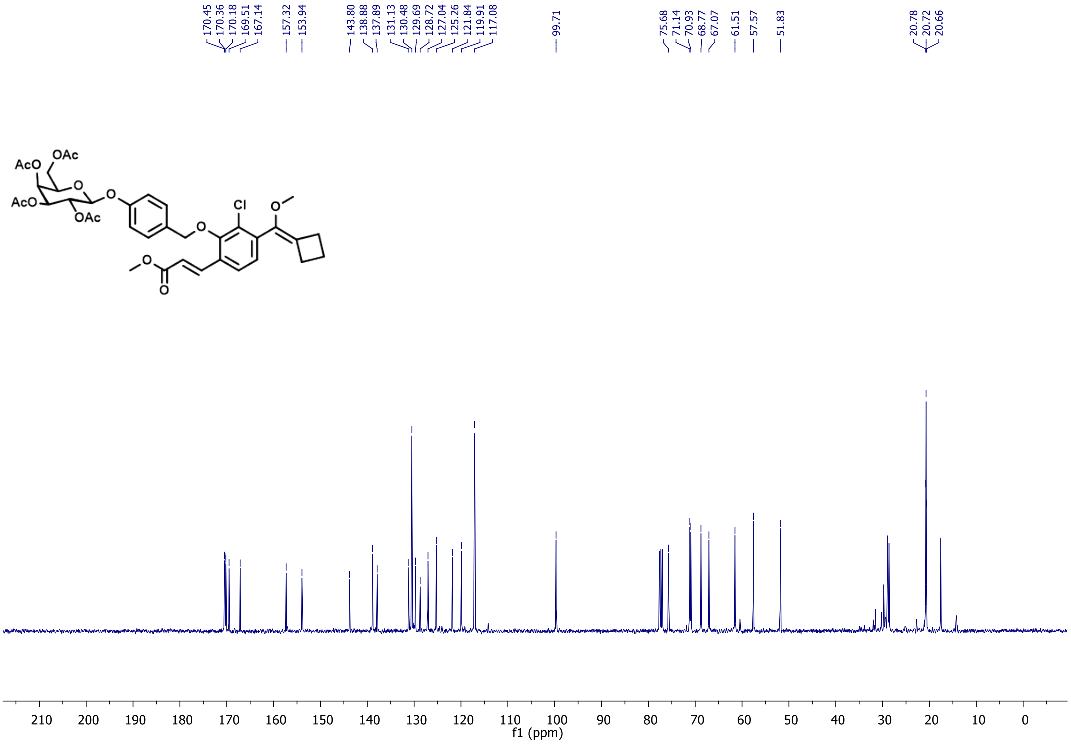

Mass spectra

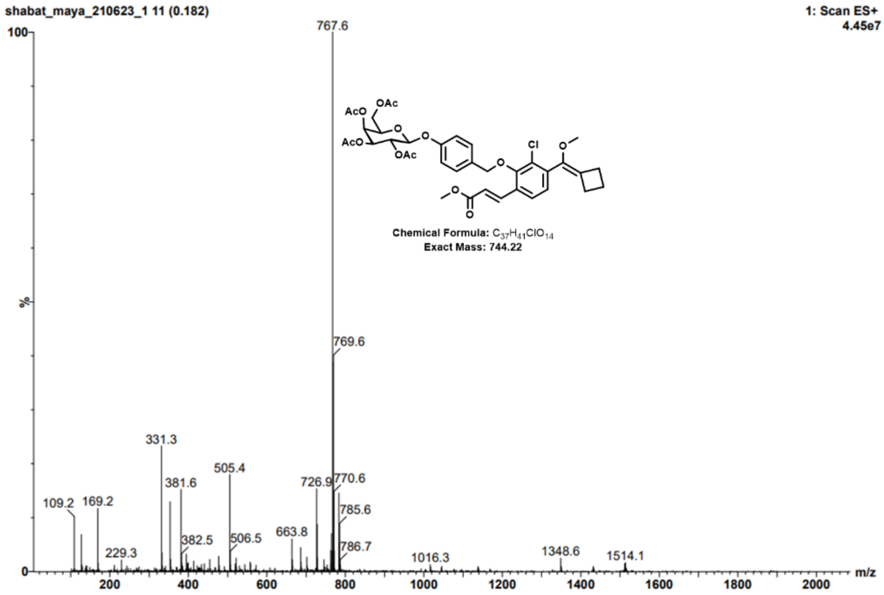

Probe MA-β-gal-CB intermediate

Mass spectra

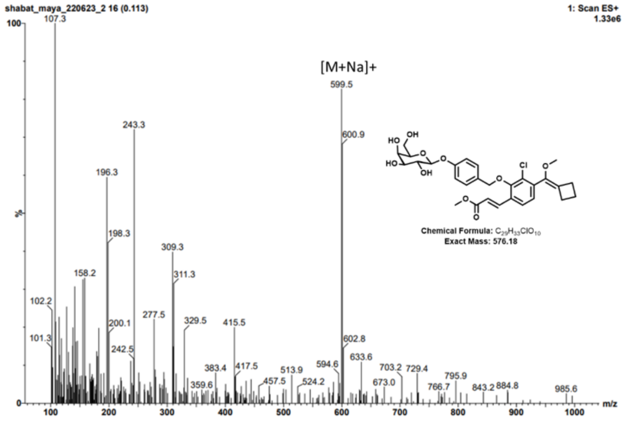

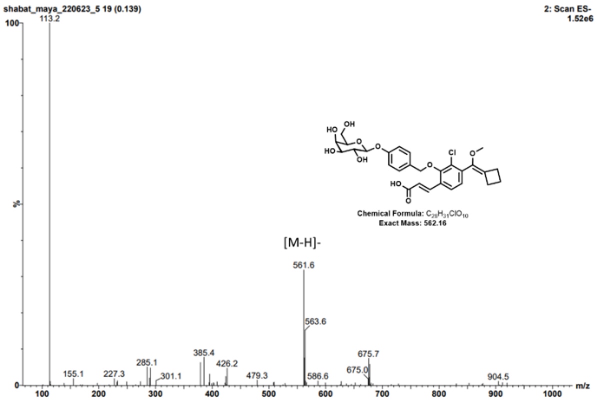

Probe MA-β-gal-CB

^{1}H-NMR

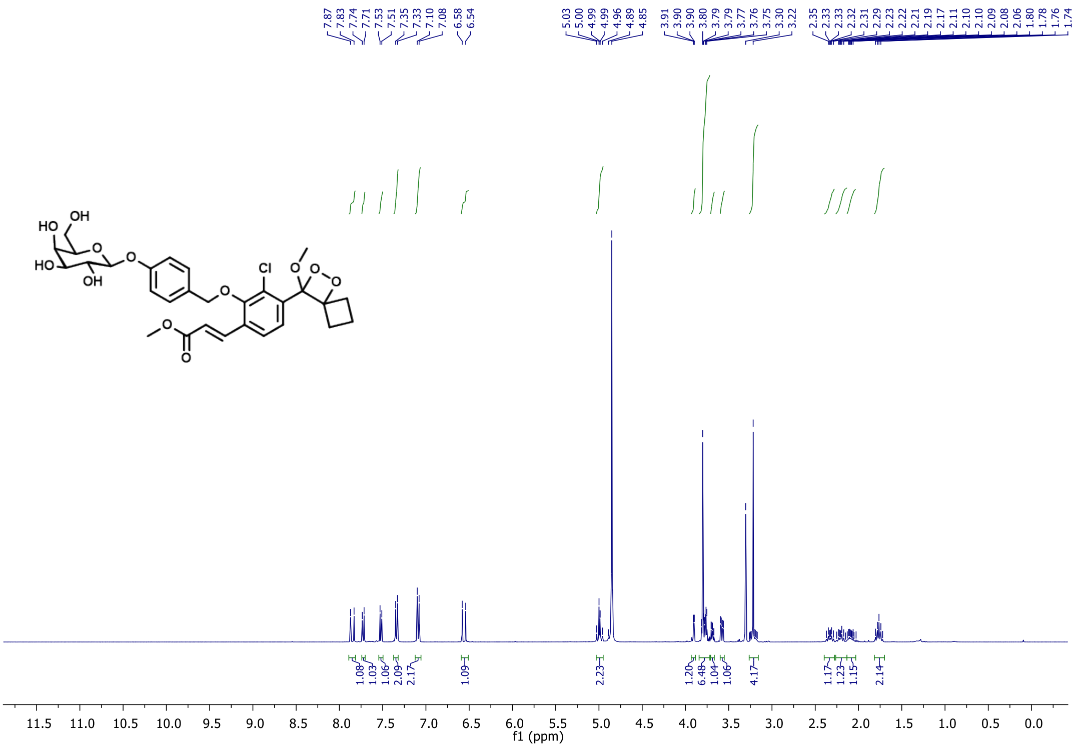

^{13}C-NMR

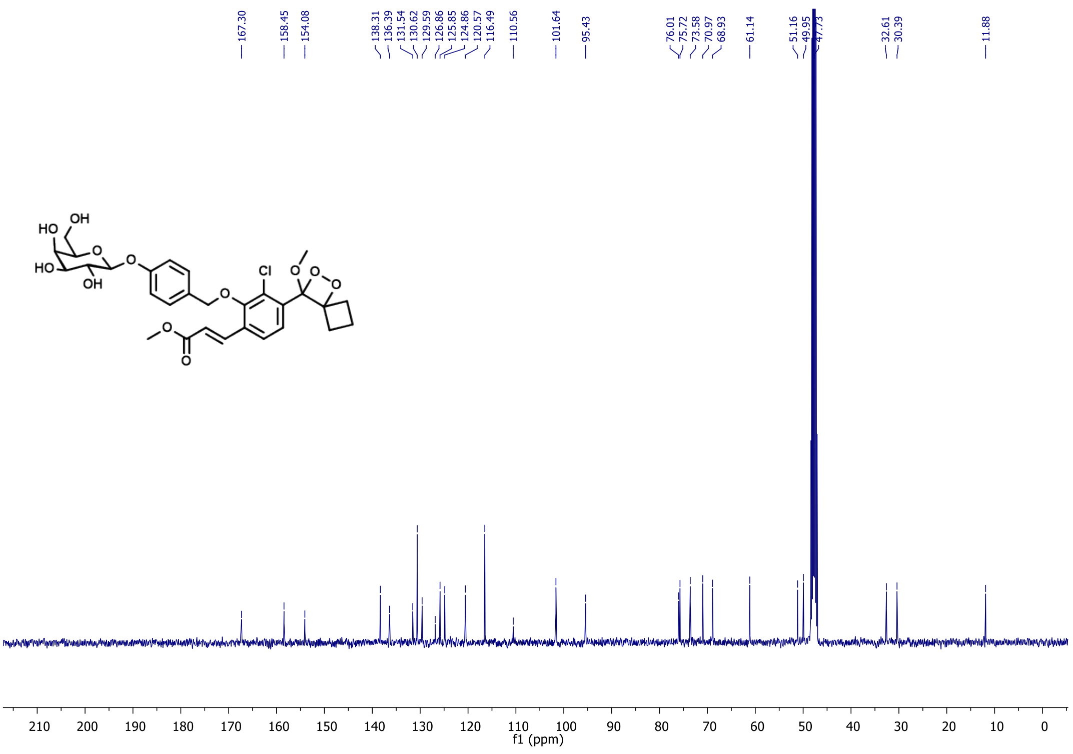

3D HPLC spectra (30-100% ACN in water, 0.1%TFA)

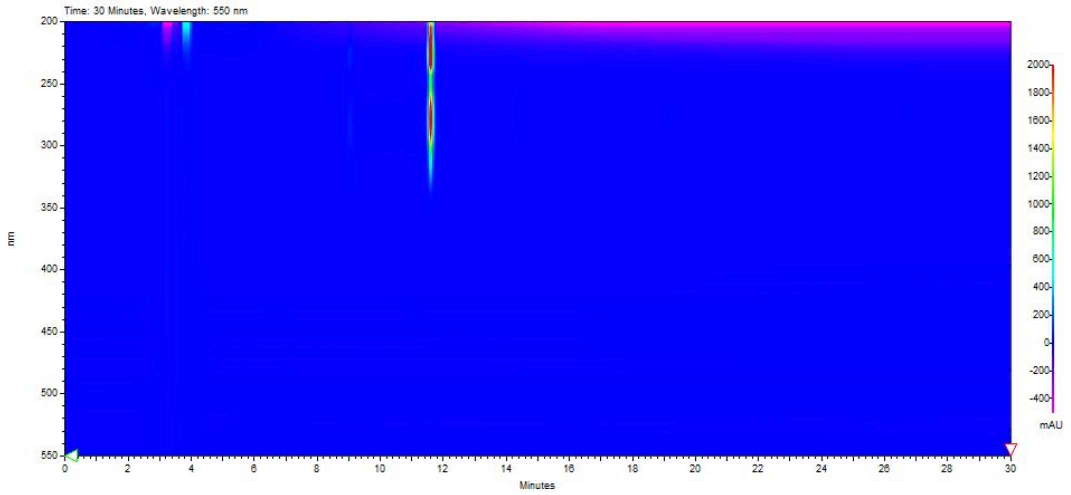

2D HPLC spectra (Absorbance measured at 280nm)

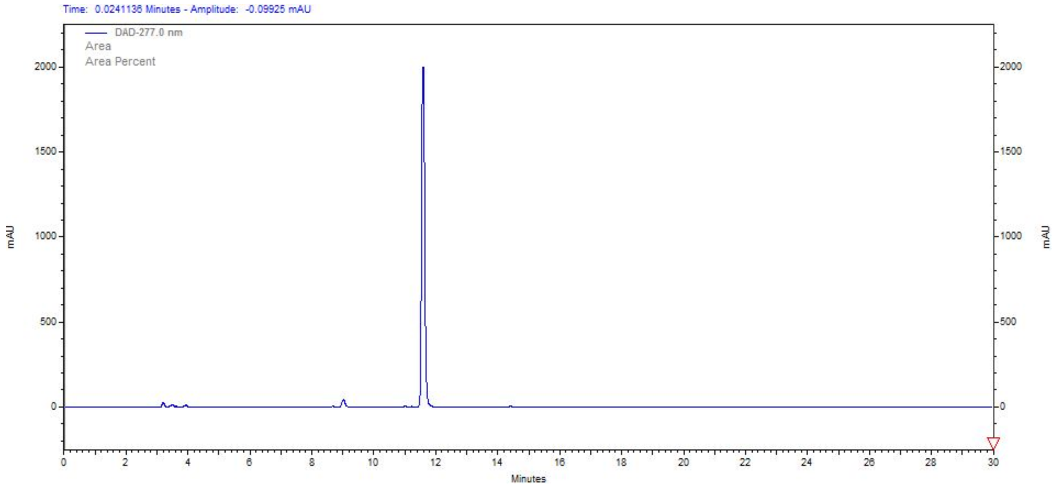

Mass spectra

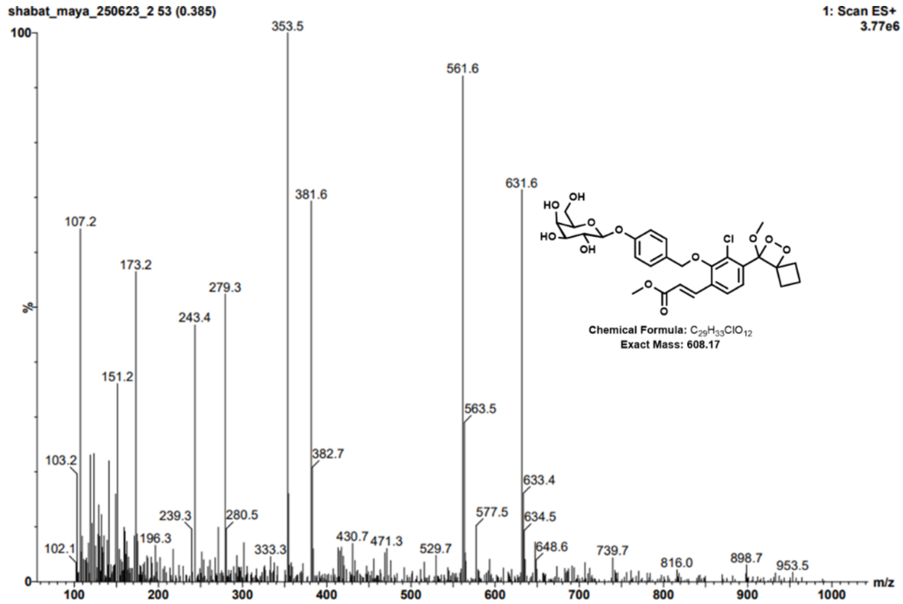

Probe AA- β-gal-CB

^{1}H-NMR

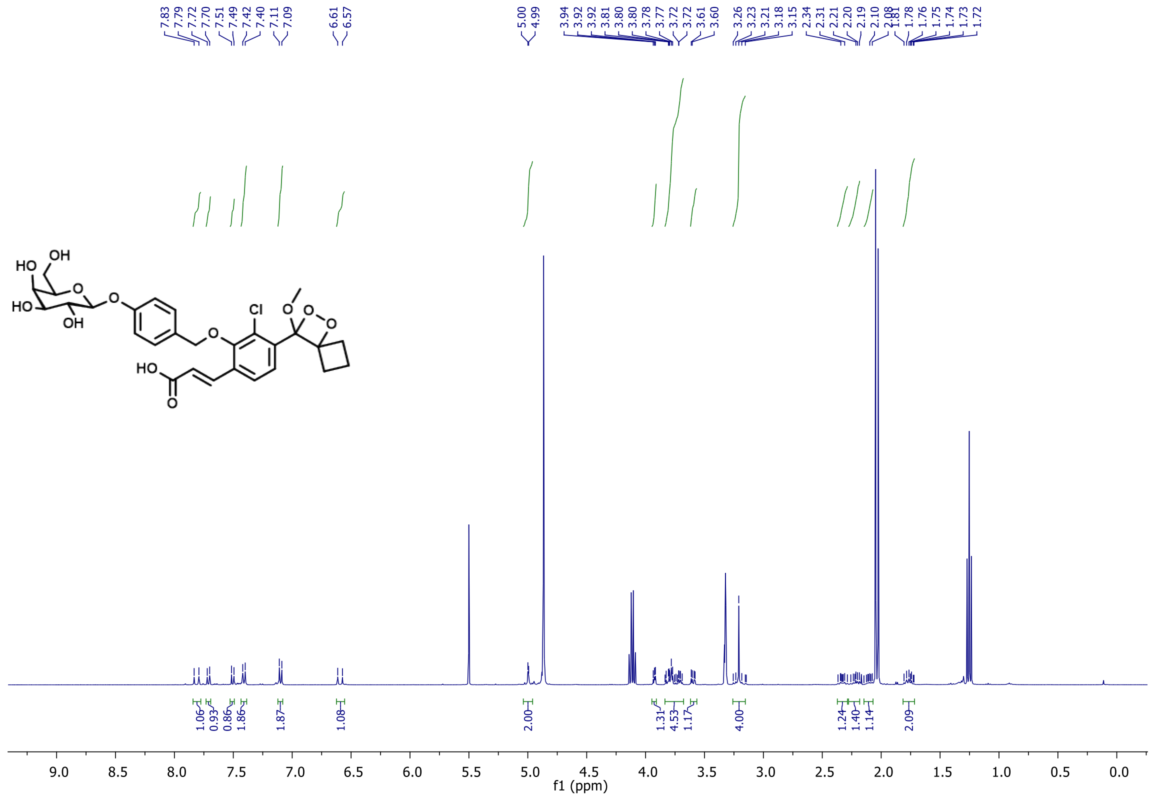

^{13}C-NMR

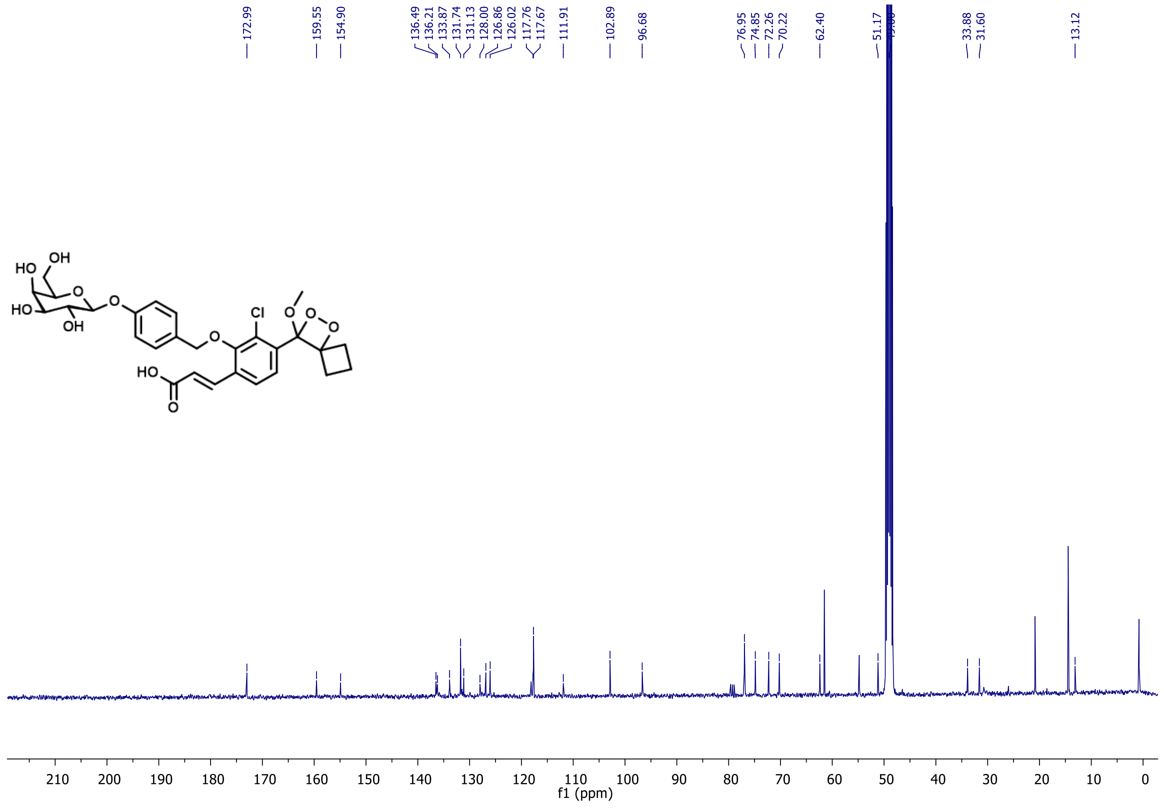

3D HPLC spectra (30-100% ACN in water, 0.1%TFA)

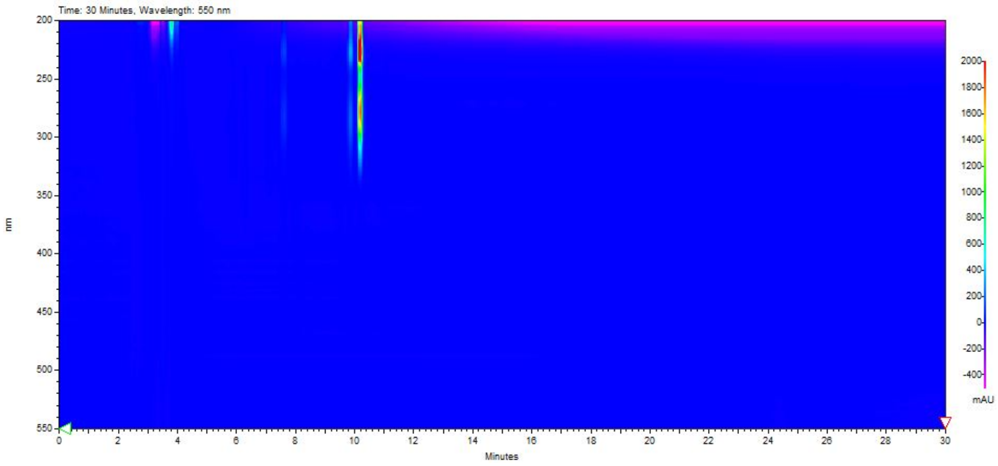

2D HPLC spectra (Absorbance measured at 280nm)

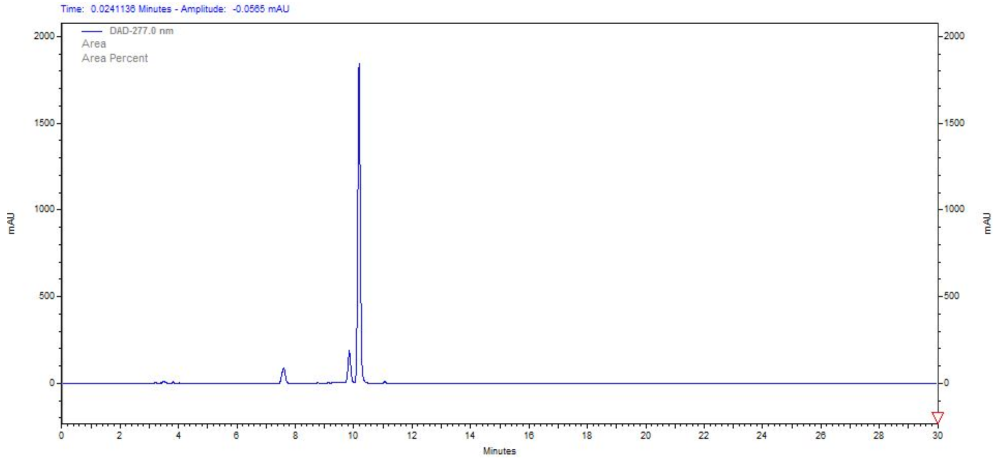

Mass spectra

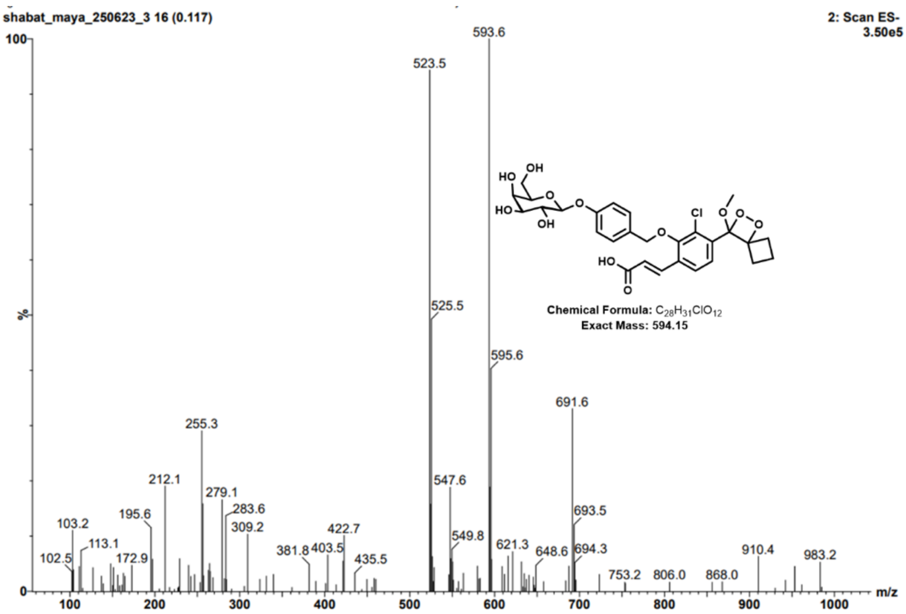

Compound 2i

^{1}H-NMR

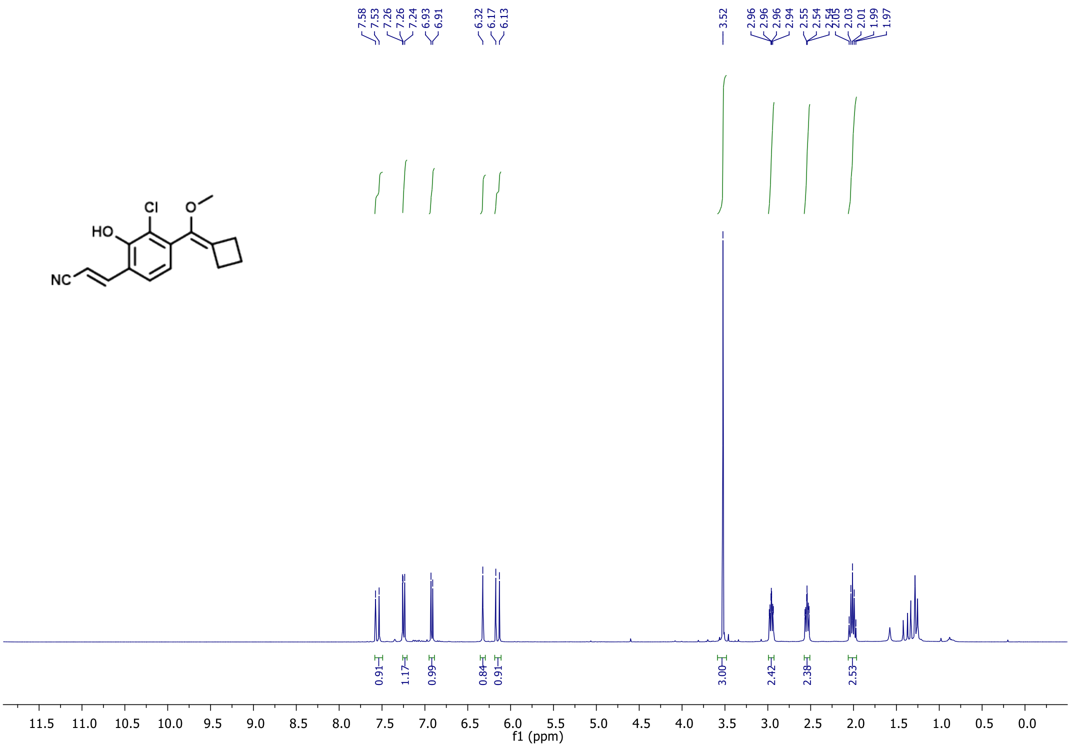

^{13}C-NMR

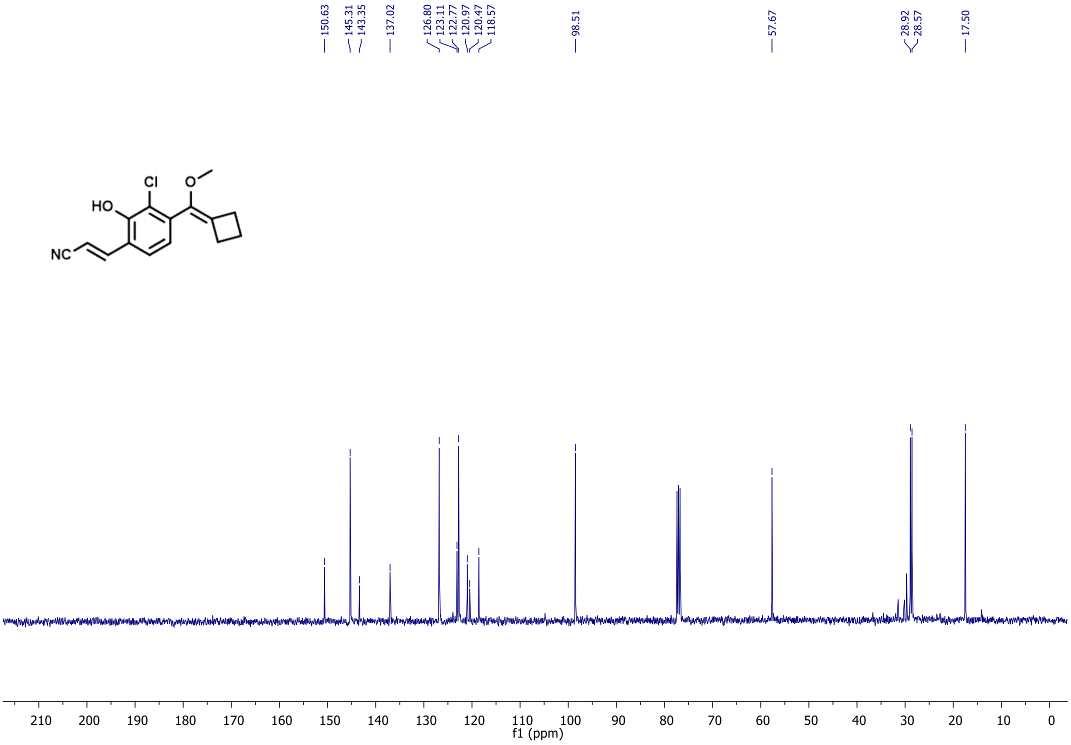

3D HPLC spectra (50-100% ACN in water, 0.1%TFA)

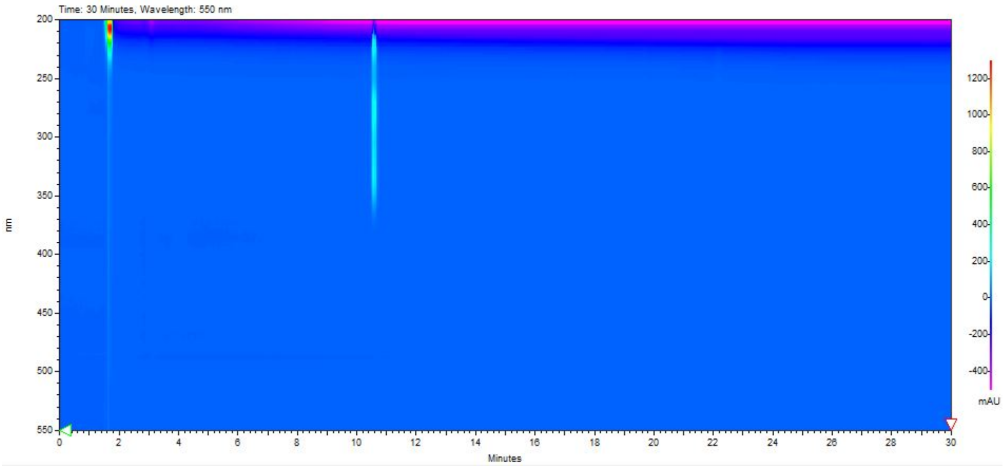

2D HPLC spectra (Absorbance measured at 330nm)

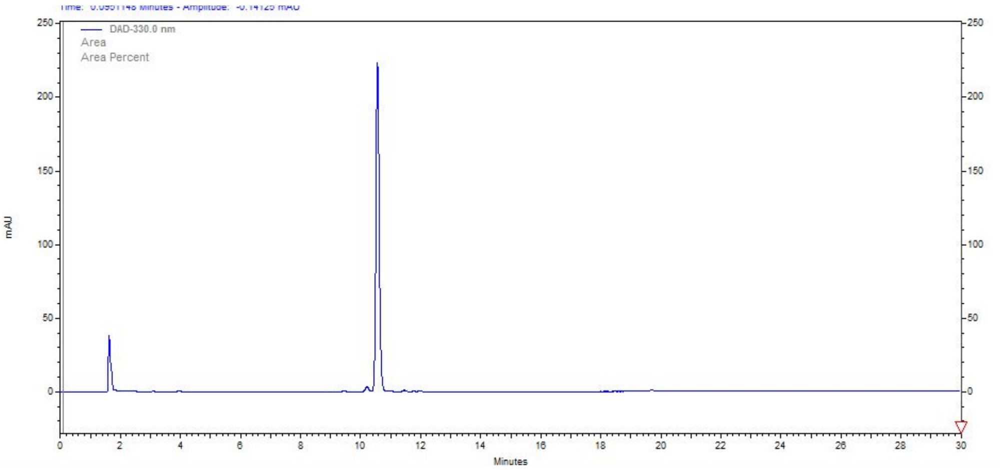

Mass spectra

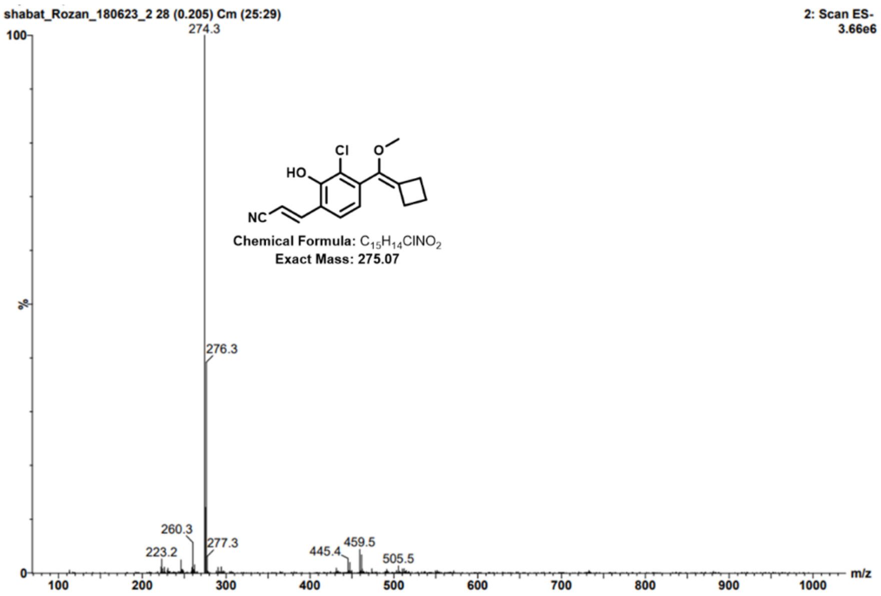

Compound 2j

^{1}H-NMR

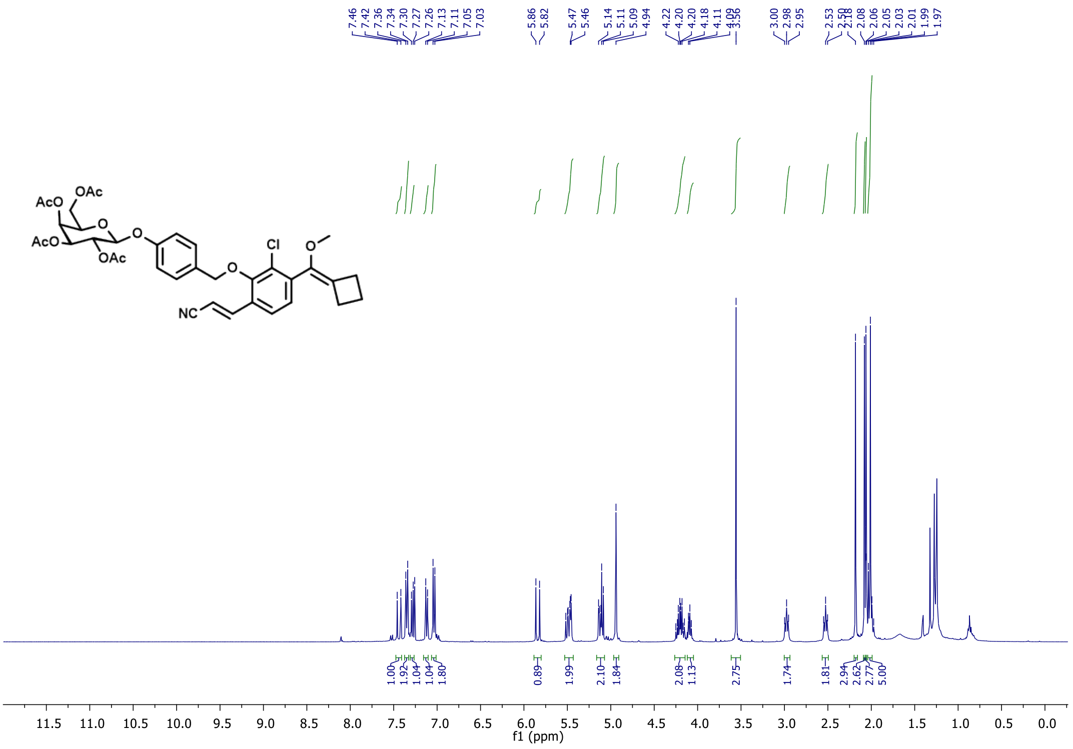

^{13}C-NMR

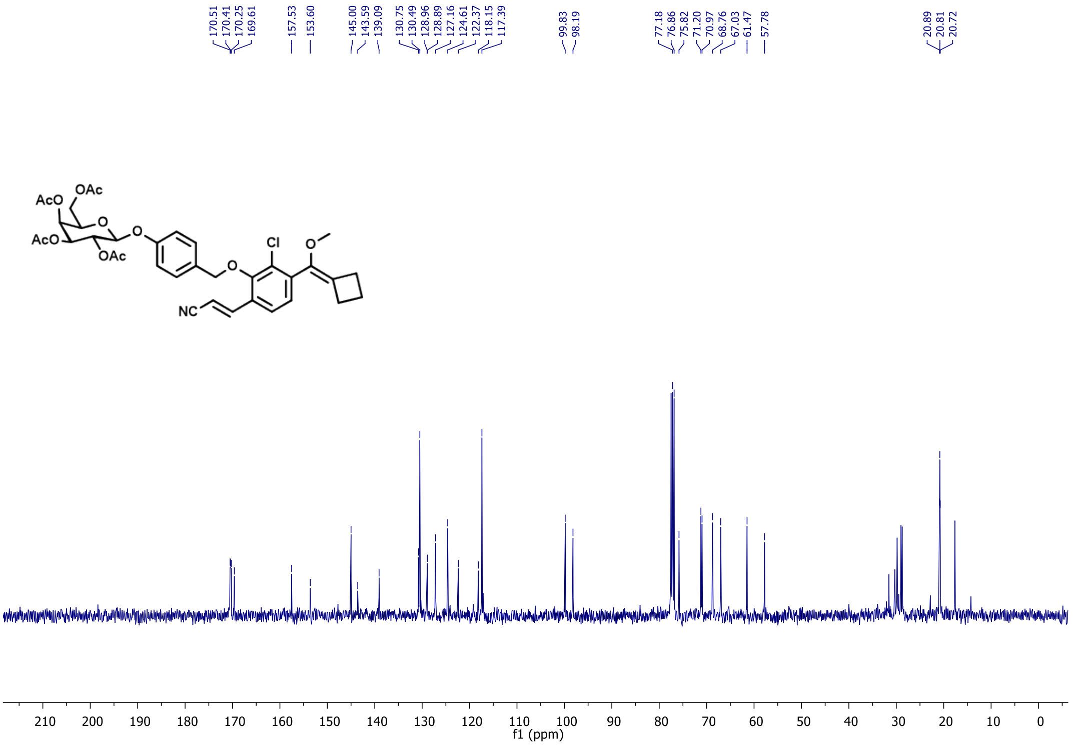

Mass spectra

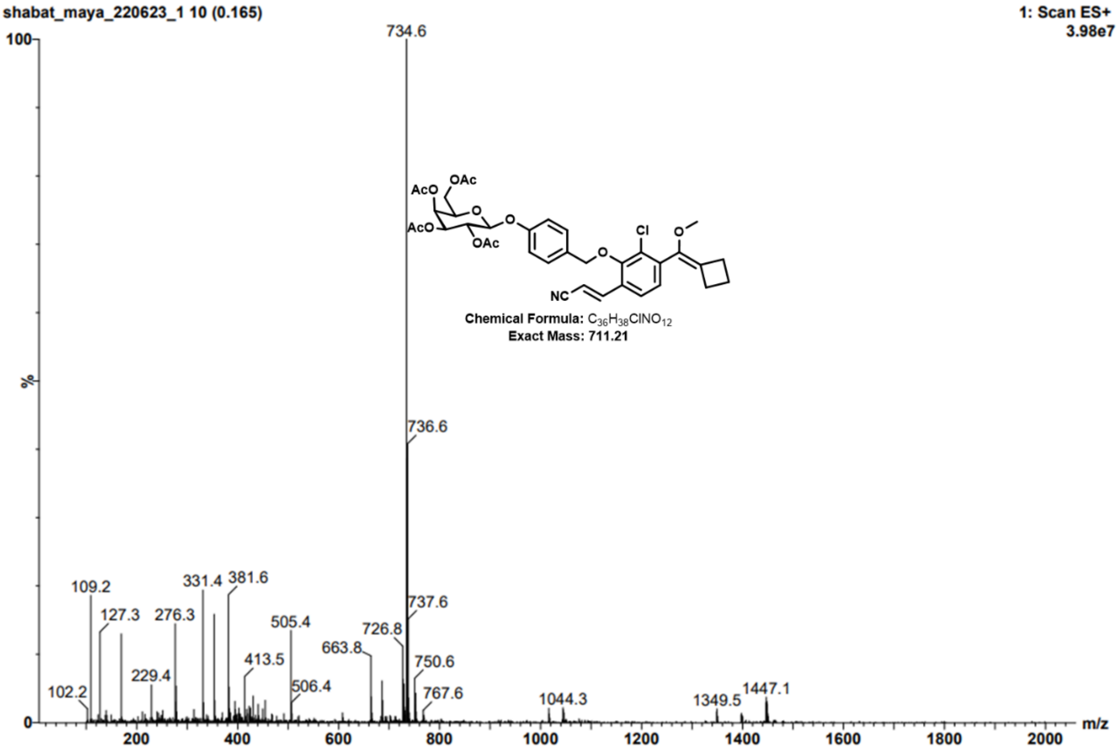

Probe CN- β-gal-CB intermediate

Mass spectra

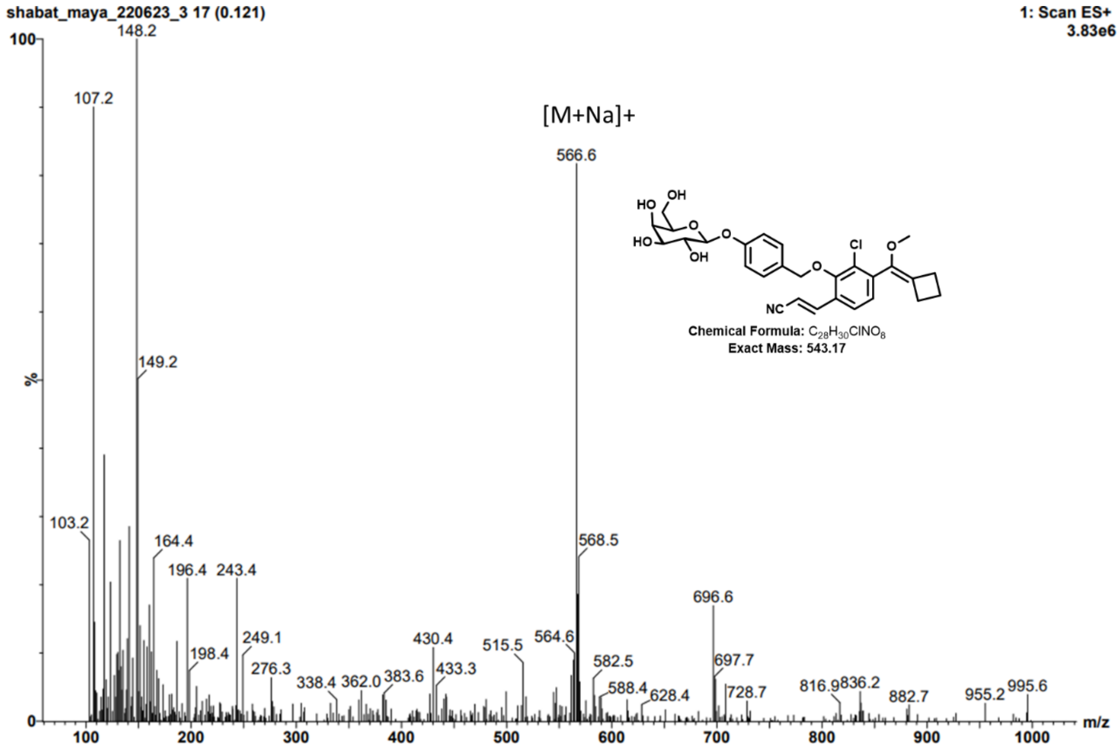

Probe CN- β-gal-CB

^{1}H-NMR

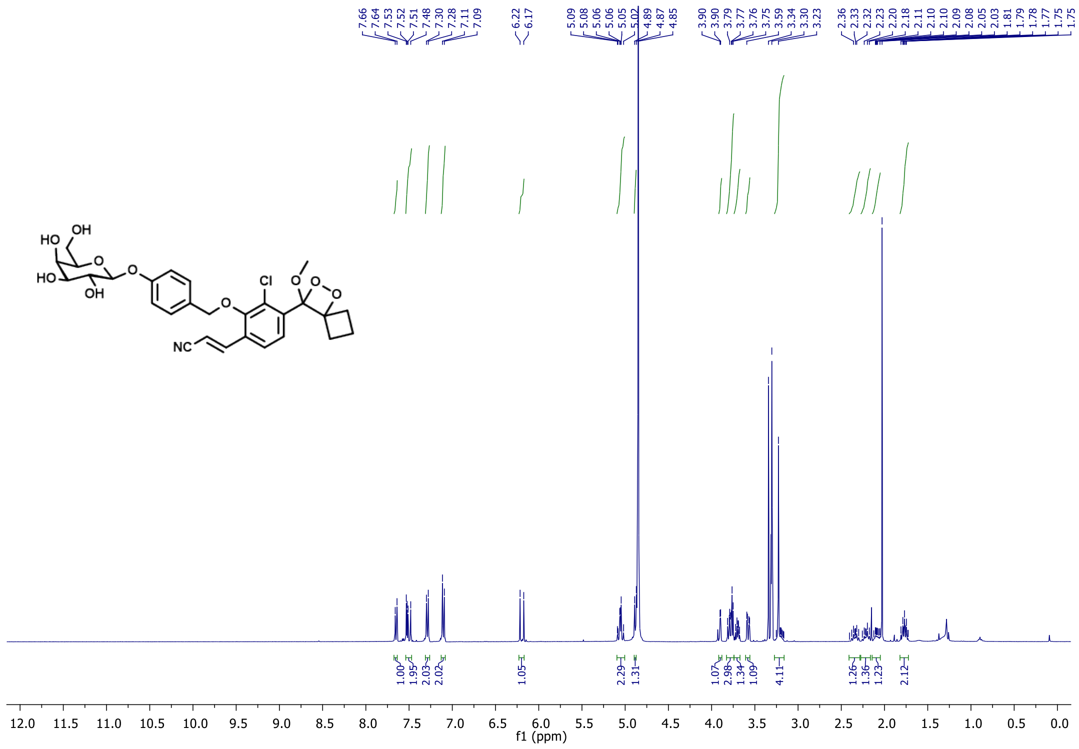

^{13}C-NMR

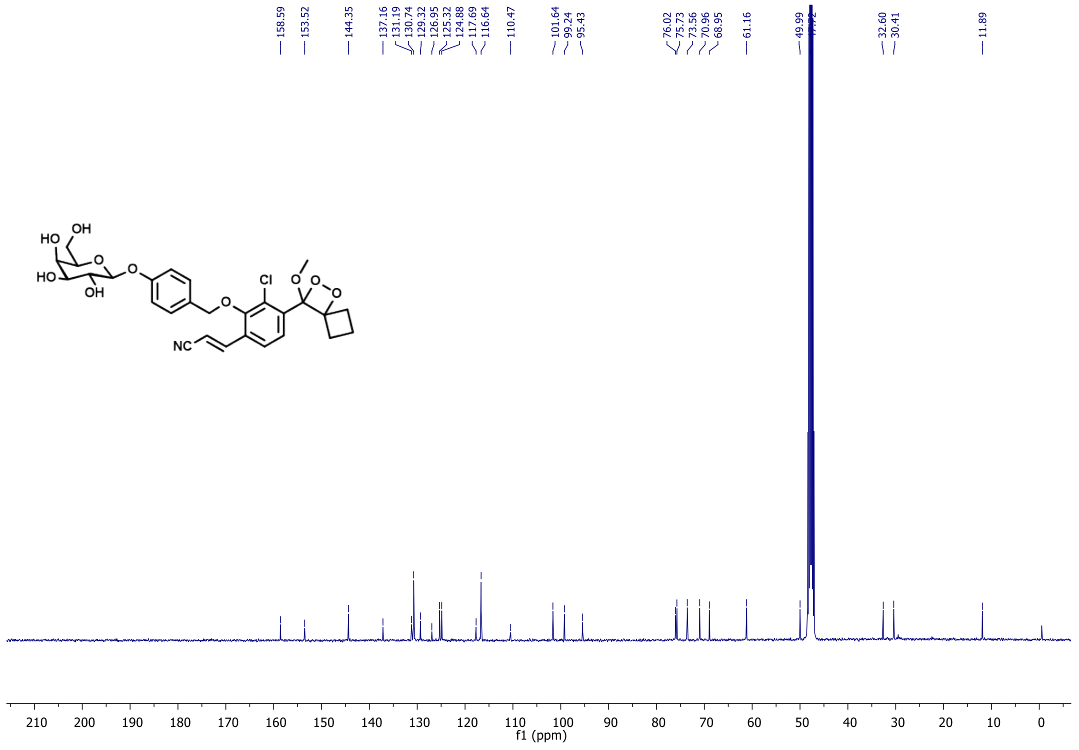

3D HPLC spectra (30-100% ACN in water, 0.1%TFA)

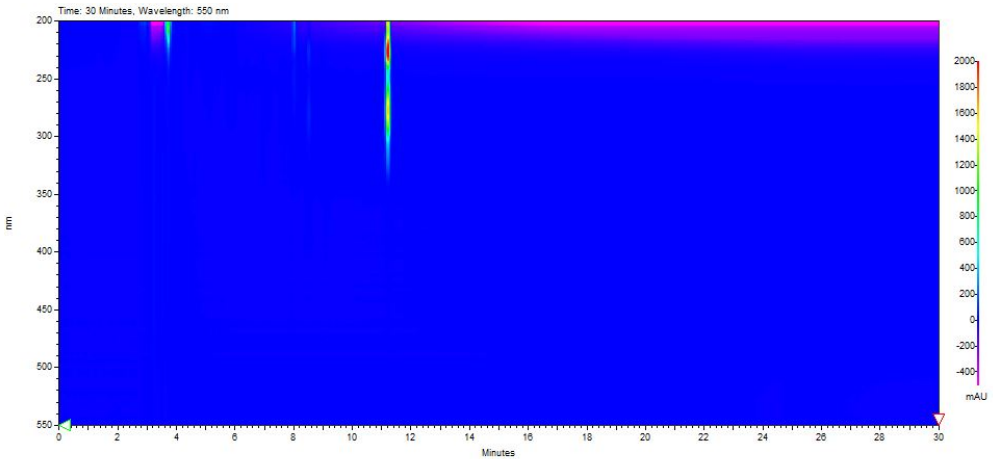

2D HPLC spectra (Absorbance measured at 330nm)

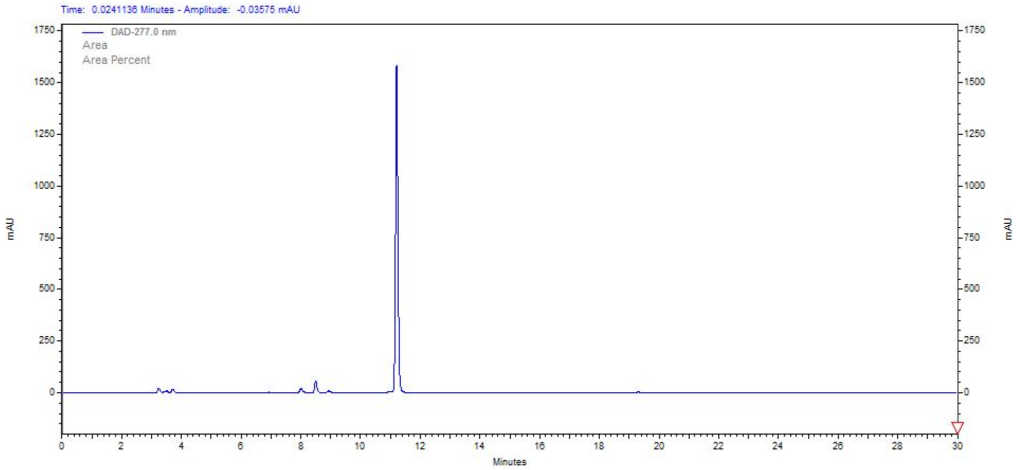

Mass spectra

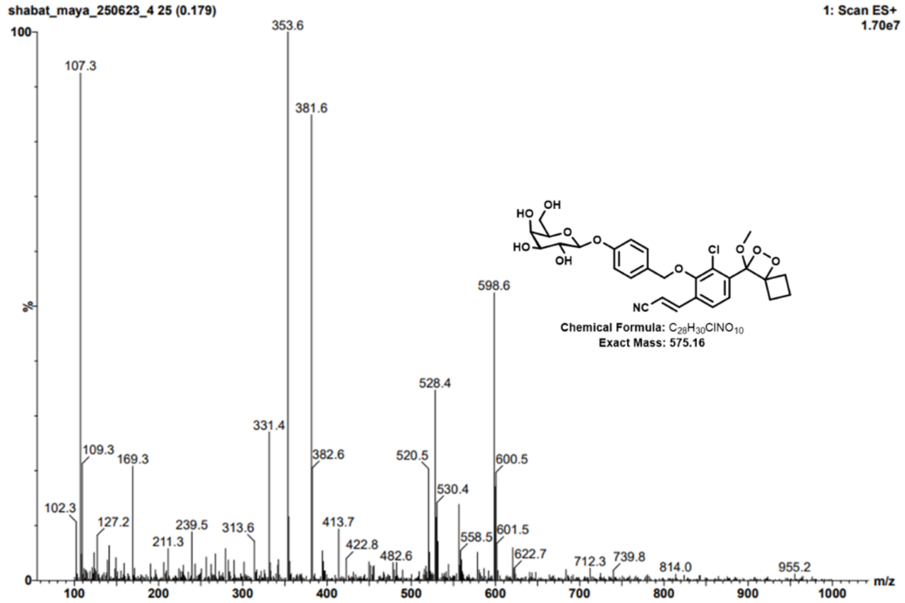

References

(1) Langhals, E.; Langhals, H. Alkylation of Ketones by Use of Solid Koh in Dimethyl-Sulfoxide. *Tetrahedron Lett.* **1990**, *31*, 859-862.

(2) Green, O.; Eilon, T.; Hananya, N.; Gutkin, S.; Bauer, C. R.; Shabat, D. Opening a Gateway for Chemiluminescence Cell Imaging: Distinctive Methodology for Design of Bright Chemiluminescent Dioxetane Probes. *ACS Cent. Sci.* **2017**, *3*, 349-358.

(3) Hananya, N.; Press, O.; Das, A.; Scomparin, A.; Satchi-Fainaro, R.; Sagi, I.; Shabat, D. Persistent Chemiluminescent Glow of Phenoxy-dioxetane Luminophore Enables Unique CRET-Based Detection of Proteases. *Chem. Eur. J.* **2019**, *25*, 14679-14687.

(4) Hananya, N.; Boock, A. E.; Bauer, C. R.; Satchi-Fainaro, R.; Shabat, D. Remarkable Enhancement of Chemiluminescent Signal by Dioxetane-Fluorophore Conjugates: Turn-ON Chemiluminescence Probes with Color Modulation for Sensing and Imaging. *J. Am. Chem. Soc.* **2016**, *138*, 13438-13446.

(5) Wang, Y.; Xu, N.; Li, W. W.; Li, J. J.; Huo, Y. J.; Zhu, W. R.; Liu, Q. Synthesis of 1,3-diselenyl-dihydroisobenzofurans via electrochemical radical selenylation with substituted o-divinylbenzenes and diselenides. *Org. Biomol. Chem.* **2022**, *20*, 2813-2817.

(6) *Gaussian 16, Revision C.01, Frisch, M. J.; Trucks, G. W.; Schlegel, H. B.; Scuseria, G. E.; Robb, M. A.; Cheeseman, J. R.; Scalmani, G.; Barone, V.; Petersson, G. A.; Nakatsuji, H.; et al. Gaussian, Inc., Wallingford CT,* 2016. (accessed 2023-07-07).

(7) Chai, J. D.; Head-Gordon, M. Long-range corrected hybrid density functionals with damped atom-atom dispersion corrections. *Phys. Chem. Chem. Phys.* **2008**, *10*, 6615-6620.

(8) Krishnan, R.; Binkley, J. S.; Seeger, R.; Pople, J. A. Self-Consistent Molecular-Orbital Methods .20. Basis Set for Correlated Wave-Functions. *J. Chem. Phys.* **1980**, *72*, 650-654.

(9) Clark, T.; Chandrasekhar, J.; Sp itznagel, G. W.; Schleyer, P. V. Efficient Diffuse Function-Augmented Basis Sets for Anion Calculations. Iii. The 3-21+G Basis Set for First-Row Elements, Li-F. *J. Comput. Chem.* **1983**, *4*, 294-301.

(10) Marenich, A. V.; Cramer, C. J.; Truhlar, D. G. Universal Solvation Model Based on Solute Electron Density and on a Continuum Model of the Solvent Defined by the Bulk Dielectric Constant and Atomic Surface Tensions. *J. Phys. Chem. B* **2009**, *113*, 6378-6396.

(11) Yanai, T.; Tew, D. P.; Handy, N. C. A new hybrid exchange-correlation functional using the Coulomb-attenuating method (CAM-B3LYP). *Chem. Phys. Lett.* **2004**, *393*, 51-57.

(12) Grimme, S.; Antony, J.; Ehrlich, S.; Krieg, H. A consistent and accurate ab initio parametrization of density functional dispersion correction (DFT-D) for the 94 elements H-Pu. *J. Chem. Phys.* **2010**, *132*, 154104.

(13) Grimme, S.; Ehrlich, S.; Goerigk, L. Effect of the Damping Function in Dispersion Corrected Density Functional Theory. *J. Comput. Chem.* **2011**, *32*, 1456-1465.

(14) Grimme, S. Supramolecular Binding Thermodynamics by Dispersion-Corrected Density Functional Theory. *Chem. Eur. J.* **2012**, *18*, 9955-9964.

(15) Luchini, G.; Alegre-Requena, J. V.; Funes-Ardoiz, I.; Paton, R. S. GoodVibes: automated thermochemistry for heterogeneous computational chemistry data. *F1000research* **2020**, *9*, 291.

(16) Shao, Y. H.; Head-Gordon, M.; Krylov, A. I. The spin-flip approach within time-dependent density functional theory: Theory and applications to diradicals. *J. Chem. Phys.* **2003**, *118*, 4807-4818.

(17) Wang, F.; Ziegler, T. Time-dependent density functional theory based on a noncollinear formulation of the exchange-correlation potential. *J. Chem. Phys.* **2004**, *121*, 12191-12196.

(18) Neese, F.; Wennmohs, F.; Becker, U.; Riplinger, C. The ORCA quantum chemistry program package. *J. Chem. Phys.* **2020**, *152,* 224108.

(19) Becke, A. D. A New Mixing of Hartree-Fock and Local Density-Functional Theories. *J. Chem. Phys.* **1993**, *98*, 1372-1377.

(20) Weigend, F.; Ahlrichs, R. Balanced basis sets of split valence, triple zeta valence and quadruple zeta valence quality for H to Rn: Design and assessment of accuracy. *Phys. Chem. Chem. Phys.* **2005**, *7*, 3297-3305.
